# Bioinformatic Approaches Including Predictive Metagenomic Profiling Reveal Characteristics of Bacterial Response to Petroleum Hydrocarbon Contamination in Diverse Environments

**DOI:** 10.1038/s41598-017-01126-3

**Published:** 2017-04-24

**Authors:** Arghya Mukherjee, Bobby Chettri, James S. Langpoklakpam, Pijush Basak, Aravind Prasad, Ashis K. Mukherjee, Maitree Bhattacharyya, Arvind K. Singh, Dhrubajyoti Chattopadhyay

**Affiliations:** 1 Department of Biotechnology, University of Calcutta, Kolkata, West Bengal India; 2 Department of Biochemistry, North-Eastern Hill University, Shillong, India; 3 Department of Biochemistry, University of Calcutta, Kolkata, West Bengal India; 4 Dr. D.Y.Patil Biotechnology and Bioinformatics Institute, Pune, India; 5 Department of Molecular Biology and Biotechnology, Tezpur University, Tezpur, India; 6 Jagadis Bose National Science Talent Search, Kolkata, West Bengal India; 7 Department of Biotechnology, Amity University, Rajarhat, New Town, Kolkata, West Bengal India

## Abstract

Microbial remediation of oil polluted habitats remains one of the foremost methods for restoration of petroleum hydrocarbon contaminated environments. The development of effective bioremediation strategies however, require an extensive understanding of the resident microbiome of these habitats. Recent developments such as high-throughput sequencing has greatly facilitated the advancement of microbial ecological studies in oil polluted habitats. However, effective interpretation of biological characteristics from these large datasets remain a considerable challenge. In this study, we have implemented recently developed bioinformatic tools for analyzing 65 16S rRNA datasets from 12 diverse hydrocarbon polluted habitats to decipher metagenomic characteristics of the resident bacterial communities. Using metagenomes predicted from 16S rRNA gene sequences through PICRUSt, we have comprehensively described phylogenetic and functional compositions of these habitats and additionally inferred a multitude of metagenomic features including 255 taxa and 414 functional modules which can be used as biomarkers for effective distinction between the 12 oil polluted sites. Additionally, we show that significantly over-represented taxa often contribute to either or both, hydrocarbon degradation and additional important functions. Our findings reveal significant differences between hydrocarbon contaminated sites and establishes the importance of endemic factors in addition to petroleum hydrocarbons as driving factors for sculpting hydrocarbon contaminated bacteriomes.

## Introduction

Anthropogenic activities and agents leading to contamination of the environment is one of the major issues that developing and developed industrial societies face today. Petroleum hydrocarbons are the most widespread of these anthropogenic agents and frequently contaminate aquatic and terrestrial ecosystems through releases of hydrocarbon during production, operational use, and transportation. The development, effectiveness and availability of technologies and strategies pose a significant challenge for the remediation, rehabilitation and restoration of these contaminated environments. Many of the technologies developed and in use for the restoration of oil contaminated environments exploit the potential of biological systems, in particular microbial systems, to use these toxic compounds as substrates for growth. Hence, much of the research conducted on bioremediation has concentrated on the capabilities of a single or couple of microbes exhibiting robust and effective growth using petroleum hydrocarbons. However, in the environment, bioremediation is often a complex process involving co-metabolism, cross-induction, inhibition and non-interaction among microbes^1–3^, possibly as petroleum hydrocarbons are a mixture of organic pollutants and therefore are used differently by different microbes. These findings, along with others, established bioremediation as a process mediated by a consortium of microbes rather than a few. Thus, characterization of microbial communities of oil contaminated environments could potentially provide guidelines for effective remediation and restoration of such environments.

Until recently, it was only possible to study a handful of microorganisms of interest isolated from source materials (as blood, soil, water or air), given the restrictions of the composition of culture media which cannot reflect and mimic the dynamic nutrient fluxes of the source environment. Indeed, only 1% of microorganisms were found to be cultivable using a set of media from the highly characterized soil rhizosphere^4^. The advent of high throughput massively-parallel sequencing methods has however, allowed us to investigate the entire complement of organisms inhabiting a certain environment. These next-generation sequencing methods (NGS) include a variety of procedures to holistically study any biological system such as amplicon sequencing (for variant identification and phylogenetic surveys), whole genome shotgun sequencing (for single organism genome and metagenomes) and RNA-Seq (for transcriptomes, metatranscriptomes and identification of non-regular RNAs). These powerful methods have ushered in rapid advances in bioinformatic approaches leading to development of software capable of handling huge amounts of data and offering meaningful biological interpretations of the same. Although a technological breakthrough in modern science, several NGS methods such as metagenomic and transcriptomic/metatranscriptomic sequencing are still expensive and hence, most studies on ecological processes like bioremediation report marker surveys as 16S rRNA gene amplicon sequencing when dealing with multiple samples. Thus, in general, most of these studies concentrated on interpretations from microbial community composition but inferred poorly regarding functional and metabolic properties of the same.

Recently, with the implementation of the Human Microbiome Project (HMP), bioinformatic advancements have been furthered through the development of powerful new computational tools for effective interpretation and visualization of taxonomic and functional composition of microbial communities^5, 6^. These tools have obvious applications for the analysis of huge amounts of microbial genomic/amplicon/transcriptomic data collected from other sources such as soil, water and so on. Some particularly interesting computational tools allow us to explain the complex mutual interactions and heterogeneity inherent in microbial communities through network-based correlation analyses^7^, prediction of metagenomic biomarkers^8^ and prediction of metagenomes from 16S rRNA data^9, 10^. While metagenomic shotgun sequencing has certain advantages over 16S rRNA gene surveys for studying microbial communities, it has some major disadvantages. These are: (i) Metagenomic shotgun sequencing can be 5–15 fold more expensive than 16S rRNA gene sequencing, which can be a limiting factor for microbial ecology research in smaller laboratories, (ii) Analysis of metagenomic shotgun sequencing datasets is manifold computationally more intensive than 16S rRNA gene analysis and requires much more advanced and powerful hardware, further escalating the operational costs, (iii) Metagenomic shotgun sequencing provides a much lower taxonomic resolution than 16S rRNA data^11^ which can lead to loss of the rare biosphere, large number of sequences being binned as unclassified and loss of information on lower level taxa (viz. genera, species) which can be projected as potential biomarkers in certain cases, and (iv) Unlike analysis of 16S rRNA gene sequences no consensus exists on the analysis of metagenomes and bioinformatic tools use diverse strategies for taxonomic and functional classification, relying on a variety of reference databases which bias the results depending on the approach taken. The availability of bioinformatic tools for prediction of functions i.e. metagenomes, from 16S rRNA gene sequences is therefore particularly attractive to microbial ecologists as it allows them to study the metabolomes of complex microbial communities with reasonable precision and confidence at a high taxonomic resolution while being able to construct robust hypotheses for further work at a much lower operational cost compared to metagenomic sequencing.

It is well understood that depending on the environment, the method of bioremediation will vary. Essential information required for the development of bioremediation technologies include the response of microbes to petroleum hydrocarbons and their dynamics with the immediate environment. Unfortunately, despite the large amount of work done on microbial community composition across a myriad of oil contaminated environments, mainly through 16S rRNA amplicon sequencing, no attempt has been made to find differential metagenomic signatures among these studies. In the present study, we have aimed to investigate the taxonomic and functional characteristics of diverse oil contaminated environments using recent bioinformatics tools through an evolving pipeline to process metagenomic data. In this bioinformatic pipeline we have employed tools that allow the analysis and interpretation of high-resolution taxonomic data generated from 16S rRNA gene surveys along with metagenome prediction tools that allow investigation of the functional dynamics of these microbial communities. We used 61 publicly available 16S rRNA datasets and 4 from this study as inputs for our analysis. Consequently, metagenomic level characteristics of bacterial composition and metabolic potential were comprehensively deduced for 12 petroleum hydrocarbon contaminated habitats. We also inferred an array of differentially abundant taxonomic and functional features which may be used as biomarkers for successful distinction of different oil contaminated habitats as well as for monitoring of bioremediation efforts in the same. Additionally, correlation between enriched taxa and functional orthologs was also evaluated along with estimation of metagenomic contributions by various taxa to hydrocarbonoclastic capabilities. Furthermore, a network of bacterial interaction patterns was inferred to deduce complex co-occurrence and co-exclusion relationships in these environments. We found that phylogenetic and functional composition of oil contaminated bacteriomes were significantly different to each other and greatly influenced by immediate environmental factors along with petroleum hydrocarbon contamination. Besides providing a robust bioinformatic pipeline for microbial ecology studies in the future, our investigation provides novel and valuable insights into the differential nature of various oil polluted habitats and hopefully improves upon previous understanding of these environments.

## Materials and Methods

### Collection and quality filtering of 16S rRNA gene sequence datasets from diverse oil contaminated environments

Sixty-one publicly available 16S rRNA datasets on oil degradation studies from 11 different environments collected along with four samples from this study were used for the present study (Table 1, Supplementary Table S1). These included four datasets representing upper soil layers of the Tundra biome (Tu), four from subsurface layers of the Tundra biome (Tb), four from the permafrost layers of the Tundra (Tp), nine from surface soil of Chinese oil refineries (C), twelve representing different regions of the arctic biome (A), four from surface soils of Indian oil refineries (I), three from mangroves (M), seven from surficial marine sediments (DWH), seven from oil sands cores (OSC), four from surface waters of oil sands tailings ponds (OSTPu), three from oil sands tailings pond waters at median depth (OSTPm) and four from deep oil sands tailings pond waters (OSTPd). We deliberately kept the taiga and OSTP samples separate even though we expected high amounts of similarity between them in certain aspects when compared to other samples, due to evidence of ample distinctive characteristics in the said samples in their parent studies^12, 13^. Oil contaminated soil samples representing Indian oil refineries (I) were collected from Noonmati Oil Refinery in Guwahati and Barhola oilfields, both in Assam, India. Soil samples were collected in both sites from the surface (0–10 cm) and beneath (20–40 cm) (Table 1, Supplementary Table S1). All the 16S rRNA datasets used can be downloaded through the list of accession numbers provided in Supplementary Table S1. All datasets used in the study presented, were sequenced in either Roche 454, Illumina or ABI Ion Torrent platforms. The 16S rRNA datasets are described in greater detail in Table 1. The downloaded 16S rRNA datasets were checked for quality using FastQC^14^ and filtered for high quality sequences in mothur^15^ using the following criteria: minimum sequence length of 100 bp, sequences trimmed when average quality drops below 20 in a sliding window of 15 bp, and a maximum of 2 mismatches in the barcode-key-template region of the reads.

**Table 1:** Summary of datasets used in the study. (For additional details, refer to Supplementary Data Table S1).

Biome Type	ID	Sequencing Platform	Location	Depth of sample collection (cm below surface)	Source material for sequencing	Predominant contaminant/hydrocarbon	Reference
Urban	I1-I4	454 GS Junior	Assam, India	0–10 and 20–30	*In situ* soil	Crude oil	This study
Arctic	A1-A12	Ion Torrent PGM	USA, Canada, Norway, Russia, and Greenland	0–15	Treated microcosm sediment	Diesel oil	Bell *et al*.^84^
Urban	C1-C9	Illumina Miseq	Changqing and Daqing, China	2–10	*In situ* soil	Crude oil	Sun *et al*.^85^
Mangrove	M1-M3	454 GS FLX	Restinga da Marambaia, Rio de Janeiro, Brazil	0–20	Treated microcosm sediment	Crude oil	dos Santos *et al*.^53^
Marine sediment	DWH1-DWH7	Illumina	Gulf of Mexico	0–1^#^	*In situ* soil	Crude oil	Mason *et al*.^54^
Taiga	Tu1-Tu4	454 GS FLX	Walagan, Walagan North, Taiyuan, and Jiagedaqi, China	20–30	Treated microcosm sediment	Crude oil	Yang *et al*.^13^
Taiga	Tb1-Tb4	454 GS FLX	Walagan, Walagan North, Taiyuan, and Jiagedaqi, China	70–80	Treated microcosm sediment	Crude oil	Yang *et al*.^13^
Taiga	Tp1-Tp4	454 GS FLX	Walagan, Walagan North, Taiyuan, and Jiagedaqi, China	140–150	Treated microcosm sediment	Crude oil	Yang *et al*.^13^
Arctic	OSC1, OSC3-5, OSC7, OSC9, OSC12	454 GS FLX	Alberta, Canada	2,985–2,990	*In situ* soil	Oil sands bitumen	An *et al*.^12^
Arctic	OSTPu1-OSTPu4	454 GS FLX	Alberta, Canada	100–240	*In situ* soil	Bitumen and various other hydrocarbons	An *et al*.^12^
Arctic	OSTPm2, OSTPm4, OSTPm6	454 GS FLX	Alberta, Canada	610–750	*In situ* soil	Bitumen and various other hydrocarbons	An *et al*.^12^
Arctic	OSTPd1-OSTPd4	454 GS FLX	Alberta, Canada	1220–1370	*In situ* soil	Bitumen and various other hydrocarbons	An *et al*.^12^

^#^All samples collected at an average of ~1500 metres below sea level, depth given is from the surface of the ocean floor.

### Analysis of microbial community structure and composition in 16S rRNA datasets

mothur^15^ was used to estimate abundances of bacterial taxa in the 16S rRNA datasets collected from diverse oil contaminated habitats. Filtered high quality sequences obtained from the quality control step were aligned to the mothur implementation of the SILVA database and trimmed for the alignment region. Chimeric sequences were then removed from the datasets using the mothur implementation of Uchime^16^. Filtered sequences were then taxonomically classified using the May 2013 release of the Greengenes database^17^ and contaminating archaeal, eukaryal, mitochondrial and chloroplast sequences or sequences classified as unknown were removed from further analysis. Finally, OTUs were predicted from these high-quality sequences. OTUs were again mapped to the sequence taxonomy file generated previously in mothur and converted to number of sequences to generate comparative taxonomy data for the datasets. We also assessed the compositional similarity between the soil samples from different sites. For doing this, we compared the pairwise taxonomic abundances from each site against each other and within the datasets as well, using Bray-Curtis measure for estimation of beta diversity^18^. The permutation-based multivariate analysis of variance (PERMANOVA) was used to test the homogeneity of taxonomic dispersion across samples along with concomitant estimation of 2D stress. Computation of Bray-Curtis distances and PERMANOVA tests were carried out in PAST v3.11^19^. The resulting Bray-Curtis similarity distance matrix was used as input for ordination of the oil contaminated samples through non-metric multidimensional scaling (NMDS) in PAST v3.11^19^.

### Metagenome prediction and metabolic reconstruction of 16S rRNA datasets

Metagenomes were predicted from 16S rRNA data using PICRUSt^9^. OTU data generated in mothur for all 16S rRNA datasets was used to prepare BIOM^20^ files formatted as input for PICRUSt v1.1.0^9^ with the *make.biom* script available in mothur. PICRUSt requires OTU abundances mapped to Greengenes OTU IDs as input for prediction of corresponding metagenomes. PICRUSt databases for 16S rRNA gene copy number normalization and KEGG ortholog prediction were updated using publicly available information listed in Integrated Microbial Genomes (IMG)^21^ as on 4^th^ April, 2016, according to the instructions provided for the Genome Prediction Tutorial for PICRUSt (http://picrust.github.io/picrust/tutorials/genome_prediction.html#genome-prediction-tutorial) using default settings. The update involved the inclusion of 16S rRNA gene copy number information and KEGG ortholog (KO) annotation data as per KEGG v77.1^22^ for ~34,000 bacterial and archaeal genomes available in IMG. 16S rRNA gene copy numbers for 16S rRNA datasets were normalized using the *normalize_by_copy_number.py* script. Metagenomes were predicted from the copy number normalized 16S rRNA data in PICRUSt using the *predict_metagenomes.py* script against the updated and PICRUSt-formatted, characterized-protein, functional database of KEGG Orthology. Contributions of various taxa to different KOs were computed with the script metagenome_contributions.py and visualized with the script *plot_metagenome_contributions*.*R* (https://groups.google.com/forum/#!topic/picrust-users/Hq9_G23J9W4) and ggplot2^23^ in R (http://www.R-project.org). Predicted metagenomes were then used as inputs in HUMAnN2^24^ for metabolic reconstruction of oil contaminated habitats using KEGG Pathways and/or KEGG modules. KEGG ortholog (KO) information derived from PICRUSt was used by MinPath^25^ implemented in HUMAnN2 to infer coverage and relative abundances of KEGG modules, which are manually defined tight, functional units. KEGG Pathways and KEGG modules (KEGG v77.1) data for HUMAnN2 were updated according to publicly available information in IMG^21^ and KEGG^22^. Coverages of a subset of KEGG modules were represented through heat maps generated in STAMP^26^.

### Identification of metagenomic biomarkers

We furthered our study through detection of taxonomic clades, KEGG orthologs and KEGG modules that are significantly over/under-represented (or differentially abundant) in the individual oil contaminated environments through statistical analyses carried out on the inferred relative abundances. To this end, the procedure of linear discriminant analysis (LDA) effect size was employed through LEfSe v1.0^8^ to identify differentially abundant features that can be used as potential metagenomic biomarkers. For this analysis, the alpha parameter significance threshold for the Krushkal-Wallis (KW) test implemented among classes in LEfSe was set to 0.01 and the logarithmic LDA score cut-off was set to 2.0, due to the relatively small sample size under consideration. All analysis carried out through LEfSe was performed through the Galaxy server^27^. Estimated biomarkers were represented using circular cladograms generated through the standalone graphical tool GraPhlAn v0.95^28^.

### Detection of associations between metagenomic gene families and taxa

Additionally, to estimate the relationship between taxonomic and functional enrichments in each oil polluted environment, we carried out tests of correlation between abundances for KEGG orthologs (metagenomic gene families) and taxonomic clades using a non-parametric test of Spearman’s rank correlation. Detection of significant relationships, defined as a correlation >0.7 with a p-value < 0.001 and reaching a Benjamini-Hochberg false discovery rate < 0.01 was carried out through the function corr.test implemented in the R package, *psych* ^29^. Correlations were only computed for oil polluted sites represented by at least 6 samples. A subset of the resultant correlation network was visualized using the interactive platform, Cytoscape v3.4.0^30^.

### Detection of bacterial interactions

Bacterial interactions in oil contaminated environments was investigated in the present study through non-random bacterial co-occurrence and co-exclusion relationships within individual soil sites. Only polluted sites consisting of more than 4 samples were subjected to deductions of bacterial interactions. mothur implementation of the Sparse Correlations for Compositional data algorithm (SparCC)^7^, a tool capable of computing significant correlations from compositional data while correcting for the effects of the same, was used to detect significant co-occurrence and co-exclusion patterns. SparCC was run on absolute count OTU tables generated by mothur for each sample, using the command *sparcc* with default settings except a single non-default parameter of permutations = 10,000. OTU associations having an absolute SparCC correlation value above 0.6 with *p*-values < 0.01 were considered statistically significant and incorporated into subsequent network construction. The final network of significant SparCC correlations was built in Cytoscape 3.4.0^30^. The nodes in the reconstructed networks represent OTUs participating in robust, statistically significant relationships (both positive and negative), which are in turn portrayed by edges i.e. connections between the nodes.

### Data Availability

16S rRNA amplicon sequencing data generated in this study was deposited in the NCBI Sequence Read Archive (SRA) under accession numbers SRR3168574-SRR3168577. The amplicon sequence data is bundled under NCBI BioProject number PRJNA306989.

## Results

### General characterization of bacterial community composition in petroleum hydrocarbon polluted habitats

**Figure 1:**
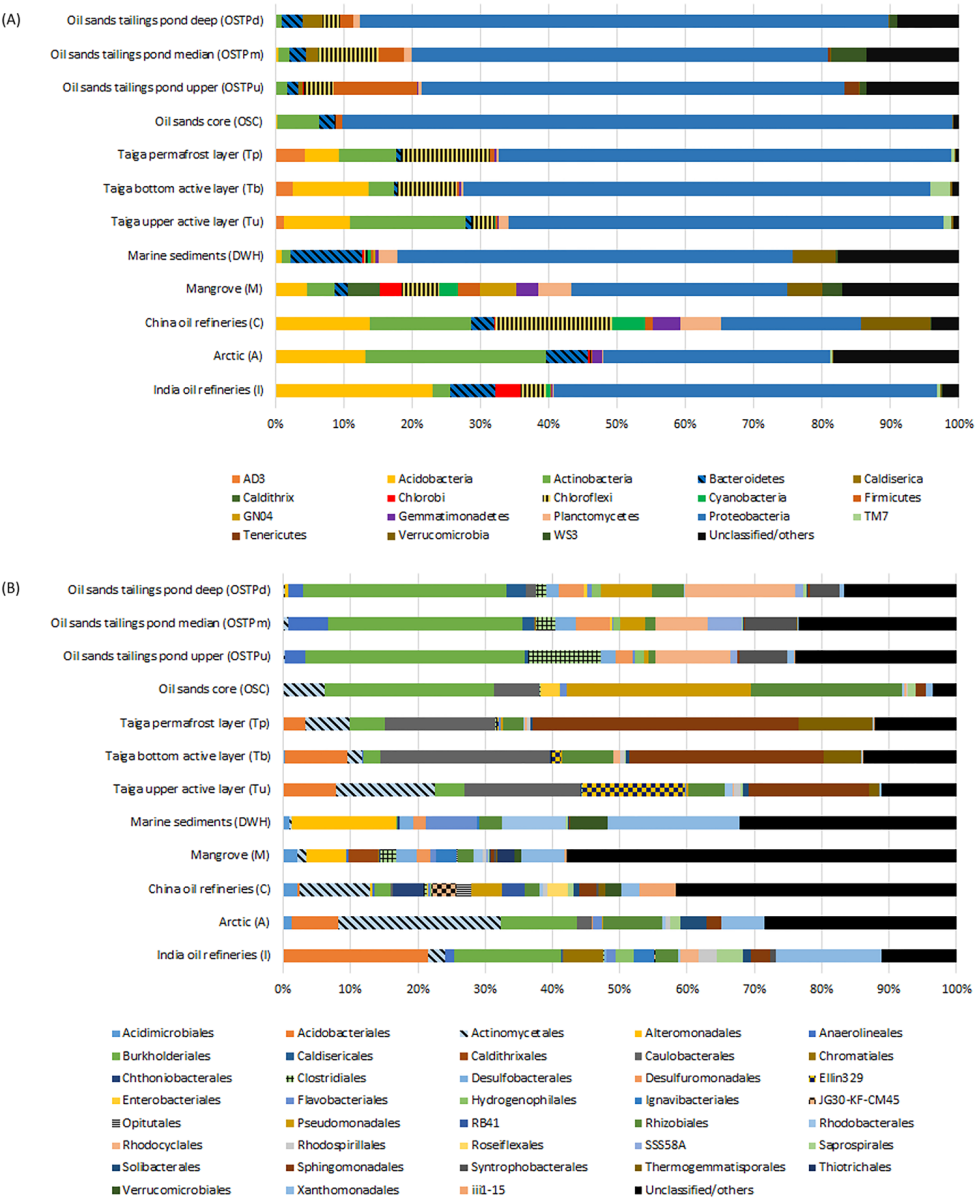
Taxonomic distribution of bacterial communities in oil contaminated environments. Taxonomic clades detected at an average relative abundance ≥2% in at least one of 12 oil contaminated habitats, (**A**) at the phylum level, and (**B**) at the order level.

Comprehensive characterization of bacterial community composition in hydrocarbon polluted environments was carried out using 61 publicly available and previously validated/published 16S rRNA amplicon sequencing datasets distributed over 11 different habitats (Table 1, Supplementary Table S1) along with 4 datasets generated in this study. mothur analysis of all datasets led to the identification of 18 phyla, 38 orders and 39 families at ≥2% average relative abundance in at least one habitat (Fig. 1A,B, Supplementary Tables S2 and S9). *Proteobacteria* dominated the bacterial community composition at the phylum level with mean relative abundances ranging from 20–77% across samples (Fig. 1A, Supplementary Table S2). *Acidobacteria* was detected in large numbers in all samples with notably decreased mean relative abundances in the OSC, OSTPu, OSTPm and OSTPd samples (Fig. 1A, Supplementary Table S2). *Actinobacteria* and *Chloroflexi* were consistently identified in all samples with significant increase in A samples, while *Bacteroidetes* showed higher average relative abundance in DWH and I samples (Fig. 1A, Supplementary Table S2). Similar to our findings, an increase in abundance for the *Actinobacteria* was reported by Yergeau *et al*. in diesel contaminated arctic soil biopiles^31^. Additionally, *Chlorobi* was detected in high mean relative abundance only in M and I samples with increased *Gemmatimonadetes* abundance identified in A, C and M samples (Fig. 1A, Supplementary Table S2). *Verrucomicrobia* contribution in microbial community composition was higher in DWH, M and C, while mean relative abundances of *Firmicutes* and *Cyanobacteria* were elevated in OSTP and C samples respectively (Fig. 1A, Supplementary Table S2). Order level taxonomic clades with average relative abundances detected at ≥2% in at least one habitat, tended to be more specific to certain samples. For instance, *Acidobacterales* had a 21% mean relative abundance in I, while *Burkholderiales* had an average relative abundance of 30% across OSTP samples and *Caulobacerales* had an average relative abundance of ~19% in taiga samples (Fig. 1B, Supplementary Table S2). Additionally, *Xanthomonadales* showed increased mean relative abundance (15–20%) in I and DWH samples and *Actinomycetales* dominated A samples with an average relative abundance of 24% (Fig. 1B, Supplementary Table S2). In addition, *Alteromonadales* (15%) was found in greater abundance in DWH samples, *Ellin329* (15%) abundance was highly elevated in Taiga upper active layer (Tu), and *Burkholderiales* (25%), *Pseudomonadales* (27%), *Rhizobiales* (22%) were enriched in the OSC (Fig. 1B, Supplementary Table S2). Bacterial families detected at ≥2% average relative abundance in a habitat also exhibited preferential sequestration to certain samples (Supplementary Table S9). While *Caulobacteraceae* and *Sphingomonadaceae* were highly enriched in the taiga samples with an average relative abundance of ~19% and ~29% respectively, *Comamonadaceae* exhibited a highly elevated mean relative abundance of 30% in the OSTP samples (Supplementary Table S9). Additionally, *Comamonadaceae* also dominated the I samples bacteriome with a mean relative abundance of 15% and contributed 10% of the bacteriome in A samples (Supplementary Table S9). Other highly specific increases in average relative abundance at the family level as compared to other samples included *Microbacteriaceae* (19%) for A samples, *Alteromonadaceae* (14%), *Xanthomonadaceae* (20%) for DWH samples, and *Moraxellaceae* (26%) for OSC samples (Supplementary Table S9).

### Similarity in bacterial community structure and detection of taxonomic biomarkers of oil polluted environments

**Figure 2:**
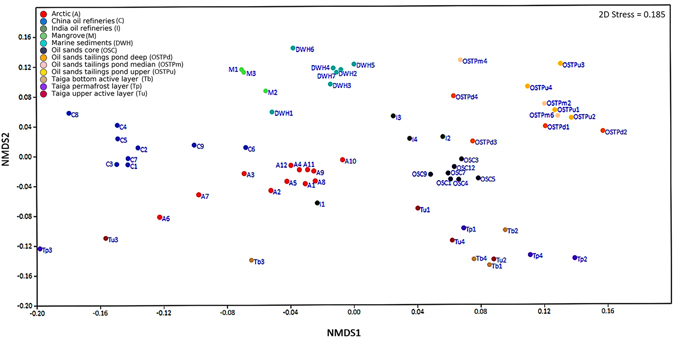
Non-metric multidimensional scaling (NMDS) plot of taxonomic composition of all oil contaminated samples of all habitats. NMDS ordination of 65 oil contaminated samples across 12 habitats was carried out based on Bray-Curtis similarity distances calculated from pairwise taxonomic profile comparisons between all samples. Taxonomic clades present in at least one sample at a relative abundance ≥0.5% were used as input. A shorter linear distance between two samples denote greater similarity between the corresponding samples. Samples from 12 environments are depicted by different colors (see legend).

Bray-Curtis similarity scores were inferred from taxonomic data generated by mothur in PAST v3.11 (Table 2) and consequently reduced to a two-dimensional space using NMDS (Fig. 2) for estimation of structural similarity of bacteriomes from petroleum hydrocarbon polluted environments. PERMANOVA tests carried out in PAST showed that taxonomic composition of bacterial communities in the oil polluted environments were significantly varied (*p* = 0.05) (Supplementary Table S7). However, there were some exceptions. The PERMANOVA results demonstrated that the taiga samples and OSTP samples were not significantly different among themselves (*p* = 0.2–0.9) (Supplementary Table S7) and that bacteriomes at these sites although separated by depth shared substantial similarity. These observations indicated that unlike large distance spatial separation i.e. geographical isolation, depth or local spatial separation is not a major defining factor for effecting substantial dissimilarity in bacterial community structure. This is well supported by the Bray-Curtis indices (Table 2) and NMDS plots of the same (Fig. 2) wherein all these samples cluster fairly closely. Additionally, polluted mangrove sediments, OSTPm and Tp samples showed similarity among themselves (*p* = 0.054–0.09) (Supplementary Table S7). Given the very low *p* values, these may be aberrations and may have occurred due to preferences, assumptions, and thresholds set in our analysis pipeline. Additionally, our observations using Bray-Curtis distances and PERMANOVA tests show that habitats showing significant similarities to OSTPm and Tp i.e. OSTPu, OSTPd and Tb, Tu respectively (Table 2, Supplementary Table S7), exhibit significantly different bacterial community structure when compared to polluted mangrove sediments (*p* = 0.0296–0.0312) (Supplementary Table S7) and with each other i.e OSTPu, OSTPd-Tb, Tu (*p* = 0.0262–0.0316) (Supplementary Table S7) thus furthering the conclusion of an aberration. All habitats showed considerable conservation of taxonomic composition within respective samples as described in Table 2. Among these intra-group interactions, OSC samples were indeed clustered in very close proximity (Fig. 2) and exhibited a Bray-Curtis similarity score of 0.85 ± 0.09, which was the highest among all inter and intra-group comparisons (Table 2). Intra-group comparisons of taiga samples showed lowest similarities (Bray-Curtis similarity score 0.45–0.57 ± 0.15) among all habitats, probably due to the sampling of source soil from 4 different regions of the China-Russia crude oil pipeline (Table 1, Supplementary Table S1, Table 2). Among the inter group comparisons, lowest similarity was observed among M and Tp samples (Bray-Curtis similarity score 0.31 ± 0.02) while the highest similarity was recorded between the relatively related environments of M and DWH (Bray-Curtis similarity score 0.54 ± 0.05) (Table 2). The taiga and OSTP samples, exhibited an inter-group Bray-Curtis similarity score similar to intra-group scores when compared within themselves (Table 2), i.e. (Tb, Tu, Tp and OSTPu, OSTPm, OSTPd). This showed that the taiga and OSTP samples were less homogenous for each habitat, while again underlining the inherent similarities in the bacterial community structure of taiga and OSTP habitats.

**Table 2:** Similarities of bacterial community structure within a habitat and between pairs of habitats expressed as Bray-Curtis distances.

**Habitat**	India oil refineries	Arctic	China oil refineries	Mangrove	Marine sediments	Taiga upper active layer	Taiga bottom active layer	Taiga permafrost layer	Oil sands core	Oil sands tailings pond upper	Oil sands tailings pond median	Oil sands tailings pond deep
India oil refineries (I)	**0**.**63 ± 0**.**06**	0.51 ± 0.07	0.39 ± 0.03	0.44 ± 0.04	0.47 ± 0.04	0.41 ± 0.06	0.41 ± 0.04	0.36 ± 0.05	0.48 ± 0.04	0.46 ± 0.08	0.46 ± 0.08	0.48 ± 0.08
Arctic (A)	0.51 ± 0.07	**0**.**72 ± 0**.**07**	0.48 ± 0.05	0.46 ± 0.03	0.42 ± 0.06	0.45 ± 0.07	0.41 ± 0.05	0.39 ± 0.05	0.49 ± 0.05	0.39 ± 0.05	0.39 ± 0.04	0.41 ± 0.06
China oil refineries (C)	0.39 ± 0.03	0.48 ± 0.05	**0**.**69 ± 0**.**08**	0.48 ± 0.02	0.40 ± 0.05	0.38 ± 0.06	0.35 ± 0.05	0.34 ± 0.06	0.37 ± 0.06	0.32 ± 0.02	0.35 ± 0.04	0.34 ± 0.05
Mangrove (M)	0.44 ± 0.04	0.46 ± 0.03	0.48 ± 0.02	**0**.**83 ± 0**.**02**	0.54 ± 0.05	0.34 ± 0.03	0.34 ± 0.02	0.31 ± 0.02	0.36 ± 0.02	0.41 ± 0.02	0.43 ± 0.04	0.41 ± 0.05
Marine sediments (DWH)	0.47 ± 0.04	0.42 ± 0.06	0.40 ± 0.05	0.54 ± 0.05	**0**.**77 ± 0**.**09**	0.35 ± 0.05	0.35 ± 0.02	0.33 ± 0.05	0.43 ± 0.02	0.38 ± 0.03	0.39 ± 0.03	0.42 ± 0.04
Taiga upper active layer (Tu)	0.41 ± 0.06	0.45 ± 0.07	0.38 ± 0.06	0.34 ± 0.03	0.35 ± 0.05	**0**.**52 ± 0**.**18**	0.59 ± 0.17	0.52 ± 0.18	0.45 ± 0.09	0.34 ± 0.06	0.35 ± 0.05	0.37 ± 0.07
Taiga bottom active layer (Tb)	0.41 ± 0.04	0.41 ± 0.05	0.35 ± 0.05	0.34 ± 0.02	0.35 ± 0.02	0.59 ± 0.17	**0**.**57 ± 0**.**12**	0.55 ± 0.20	0.45 ± 0.05	0.33 ± 0.04	0.34 ± 0.04	0.37 ± 0.06
Taiga permafrost layer (Tp)	0.36 ± 0.05	0.39 ± 0.05	0.34 ± 0.06	0.31 ± 0.02	0.33 ± 0.05	0.52 ± 0.18	0.55 ± 0.20	**0**.**45 ± 0**.**22**	0.42 ± 0.10	0.32 ± 0.06	0.33 ± 0.05	0.36 ± 0.08
Oil sands core (OSC)	0.48 ± 0.04	0.49 ± 0.05	0.37 ± 0.06	0.36 ± 0.02	0.43 ± 0.02	0.45 ± 0.09	0.45 ± 0.05	0.42 ± 0.10	**0**.**85 ± 0**.**09**	0.45 ± 0.05	0.45 ± 0.04	0.56 ± 0.10
Oil sands tailings pond upper (OSTPu)	0.46 ± 0.08	0.39 ± 0.05	0.32 ± 0.02	0.41 ± 0.02	0.38 ± 0.03	0.34 ± 0.06	0.33 ± 0.04	0.32 ± 0.06	0.45 ± 0.05	**0**.**67 ± 0**.**12**	0.64 ± 0.09	0.63 ± 0.13
Oil sands tailings pond median (OSTPm)	0.46 ± 0.08	0.39 ± 0.04	0.35 ± 0.04	0.43 ± 0.04	0.39 ± 0.03	0.35 ± 0.05	0.34 ± 0.04	0.33 ± 0.05	0.45 ± 0.04	0.64 ± 0.09	**0**.**56 ± 0**.**05**	0.61 ± 0.12
Oil sands tailings pond deep (OSTPd)	0.48 ± 0.08	0.41 ± 0.06	0.34 ± 0.05	0.41 ± 0.05	0.42 ± 0.04	0.37 ± 0.07	0.37 ± 0.06	0.36 ± 0.08	0.56 ± 0.10	0.63 ± 0.13	0.61 ± 0.12	**0**.**61 ± 0**.**15**

To further investigate taxonomic apportionment and detect differentially abundant clades in various oil polluted environments, we compared the abundances of clades detected at an abundance of ≥0.5% in at least 5 samples, at each taxonomic level. The consequent taxonomic profile inferred for all samples (from domain to species level) was then used by LEfSe to detect metagenomic biomarkers. In all, LEfSe detected 255 differentially abundant taxa including 66 families, 47 genera and 11 species level biomarkers across all habitats (Table 3, Supplementary Figure S2, Supplementary Tables S2 and S5). The largest number of taxonomic biomarkers were detected for the C samples (68) while the lowest were recorded for both OSTPd and Tu (7) (Supplementary Table S5). The very low number of detected taxonomic biomarkers for OSTPd and Tu may be a fallout of the comparatively higher bacterial community structure similarity between taiga and OSTP samples than others leading to smaller tally of unique and significantly differential clades. Taxonomic biomarkers detected at the family level are listed in Table 3. At the genus level, *Phenylobacterium* and *Novosphingobium* were detected as biomarkers for Tp samples, while genera such as *Geobacter*, *Syntrophus*, *Microbacterium*, *Mycobacterium*, *HB2.32.21*, *Candidatus Koribacter*, *Methylobacterium*, *Caulobacter*, and *Rhodococcus* were attributed as biomarkers for OSTPu, OSTPm, A, C, DWH, I, OSC, Tb, and Tu samples respectively (Supplementary Fig. S2, Supplementary Tables S2 and S5). Pathogenic microorganisms are known to be important degraders of petroleum hydrocarbons. Several strains of infectious microbes such as *Burkholderia* ^32^, *Stenotrophomonas* ^33, 34^ and *Mycobacterium* ^35^ have been shown to harbor impressive capabilities for degradation of a variety of petroleum hydrocarbons. In the present study, pathogens such as *Mycobacterium* and *Burkholderia*, were identified as genus level biomarkers for C samples and Tb samples respectively. Interestingly, LEfSe detected 19 phylum level biomarkers which indicate that preferential proliferation of bacterial lineages emanating from particular higher level taxa, probably driven by hydrocarbon stress, is possible and may lead to definitive compositional differences between oil polluted habitats (Supplementary Fig. S2, Supplementary Tables S2 and S5). Moreover, candidate phyla such as AC1, WS3 and WS6 were identified as biomarkers for OSTP samples which also underline the uniqueness of these environments (Supplementary Fig. S2, Supplementary Tables S2 and S5). However, further investigations are required to gather information on possibly distinctive roles played by these phyla in these habitats.

**Table 3:** Summary table showing differentially abundant bacterial clades at the Family level detected by LEfSe.

**Differentially abundant Taxa**	**Habitat** ^**†**^	**Taxonomy** ^**‡**^
*Iamiaceae*	A	Bacteria|Actinobacteria|Acidimicrobiia|Acidimicrobiales|Iamiaceae
*Microbacteriaceae*	A	Bacteria|Actinobacteria|Actinobacteria|Actinomycetales|Microbacteriaceae
*mb2424*	C	Bacteria|Acidobacteria|Acidobacteria 6|iii1 15|mb2424
*Ellin6075*	C	Bacteria|Acidobacteria|Chloracidobacteria|RB41|Ellin6075
*Dietziaceae*	C	Bacteria|Actinobacteria|Actinobacteria|Actinomycetales|Dietziaceae
*Geodermatophilaceae*	C	Bacteria|Actinobacteria|Actinobacteria|Actinomycetales|Geodermatophilaceae
*Micromonosporaceae*	C	Bacteria|Actinobacteria|Actinobacteria|Actinomycetales|Micromonosporaceae
*Mycobacteriaceae*	C	Bacteria|Actinobacteria|Actinobacteria|Actinomycetales|Mycobacteriaceae
*Nocardiaceae*	C	Bacteria|Actinobacteria|Actinobacteria|Actinomycetales|Nocardiaceae
*Solirubrobacteraceae*	C	Bacteria|Actinobacteria|Thermoleophilia|Solirubrobacterales|Solirubrobacteraceae
*Cytophagaceae*	C	Bacteria|Bacteroidetes|Cytophagia|Cytophagales|Cytophagaceae
*Kouleothrixaceae*	C	Bacteria|Chloroflexi|Chloroflexi|Roseiflexales|Kouleothrixaceae
*Dolo 23*	C	Bacteria|Chloroflexi|TK10|AKYG885|Dolo 23
*Gemmataceae*	C	Bacteria|Planctomycetes|Planctomycetia|Gemmatales|Gemmataceae
*Pirellulaceae*	C	Bacteria|Planctomycetes|Planctomycetia|Pirellulales|Pirellulaceae
*Planctomycetaceae*	C	Bacteria|Planctomycetes|Planctomycetia|Planctomycetales|Planctomycetaceae
*Myxococcaceae*	C	Bacteria|Proteobacteria|Deltaproteobacteria|Myxococcales|Myxococcaceae
*Opitutaceae*	C	Bacteria|Verrucomicrobia|Opitutae|Opitutales|Opitutaceae
*Chthoniobacteraceae*	C	Bacteria|Verrucomicrobia|Spartobacteria|Chthoniobacterales|Chthoniobacteraceae
*Flavobacteriaceae*	DWH	Bacteria|Bacteroidetes|Flavobacteriia|Flavobacteriales|Flavobacteriaceae
*Weeksellaceae*	DWH	Bacteria|Bacteroidetes|Flavobacteriia|Flavobacteriales|Weeksellaceae
*Rhodobacteraceae*	DWH	Bacteria|Proteobacteria|Alphaproteobacteria|Rhodobacterales|Rhodobacteraceae
*Alteromonadaceae*	DWH	Bacteria|Proteobacteria|Gammaproteobacteria|Alteromonadales|Alteromonadaceae
*Colwelliaceae*	DWH	Bacteria|Proteobacteria|Gammaproteobacteria|Alteromonadales|Colwelliaceae
*Marinicellaceae*	DWH	Bacteria|Proteobacteria|Gammaproteobacteria|Marinicellales|Marinicellaceae
*Xanthomonadaceae*	DWH	Bacteria|Proteobacteria|Gammaproteobacteria|Xanthomonadales|Xanthomonadaceae
*Verrucomicrobiaceae*	DWH	Bacteria|Verrucomicrobia|Verrucomicrobiae|Verrucomicrobiales|Verrucomicrobiaceae
*Acidobacteriaceae*	I	Bacteria|Acidobacteria|Acidobacteriia|Acidobacteriales|Acidobacteriaceae
*Koribacteraceae*	I	Bacteria|Acidobacteria|Acidobacteriia|Acidobacteriales|Koribacteraceae
*Chitinophagaceae*	I	Bacteria|Bacteroidetes|Saprospirae|Saprospirales|Chitinophagaceae
*Ignavibacteriaceae*	I	Bacteria|Chlorobi|Ignavibacteria|Ignavibacteriales|Ignavibacteriaceae
*Acetobacteraceae*	I	Bacteria|Proteobacteria|Alphaproteobacteria|Rhodospirillales|Acetobacteraceae
*Rhodospirillaceae*	I	Bacteria|Proteobacteria|Alphaproteobacteria|Rhodospirillales|Rhodospirillaceae
*Hydrogenophilaceae*	I	Bacteria|Proteobacteria|Betaproteobacteria|Hydrogenophilales|Hydrogenophilaceae
*Sinobacteraceae*	I	Bacteria|Proteobacteria|Gammaproteobacteria|Xanthomonadales|Sinobacteraceae
*Phycisphaeraceae*	M	Bacteria|Planctomycetes|Phycisphaerae|Phycisphaerales|Phycisphaeraceae
*Erythrobacteraceae*	M	Bacteria|Proteobacteria|Alphaproteobacteria|Sphingomonadales|Erythrobacteraceae
*Desulfuromonadaceae*	M	Bacteria|Proteobacteria|Deltaproteobacteria|Desulfuromonadales|Desulfuromonadaceae
*Spirochaetaceae*	M	Bacteria|Spirochaetes|Spirochaetes|Spirochaetales|Spirochaetaceae
*Propionibacteriaceae*	OSC	Bacteria|Actinobacteria|Actinobacteria|Actinomycetales|Propionibacteriaceae
*Brucellaceae*	OSC	Bacteria|Proteobacteria|Alphaproteobacteria|Rhizobiales|Brucellaceae
*Methylobacteriaceae*	OSC	Bacteria|Proteobacteria|Alphaproteobacteria|Rhizobiales|Methylobacteriaceae
*Oxalobacteraceae*	OSC	Bacteria|Proteobacteria|Betaproteobacteria|Burkholderiales|Oxalobacteraceae
*Enterobacteriaceae*	OSC	Bacteria|Proteobacteria|Gammaproteobacteria|Enterobacteriales|Enterobacteriaceae
*Moraxellaceae*	OSC	Bacteria|Proteobacteria|Gammaproteobacteria|Pseudomonadales|Moraxellaceae
*Rhodocyclaceae*	OSTPd	Bacteria|Proteobacteria|Betaproteobacteria|Rhodocyclales|Rhodocyclaceae
*Anaerolinaceae*	OSTPm	Bacteria|Chloroflexi|Anaerolineae|Anaerolineales|Anaerolinaceae
*Desulfobulbaceae*	OSTPm	Bacteria|Proteobacteria|Deltaproteobacteria|Desulfobacterales|Desulfobulbaceae
*Syntrophaceae*	OSTPm	Bacteria|Proteobacteria|Deltaproteobacteria|Syntrophobacterales|Syntrophaceae
*Pseudomonadaceae*	OSTPm	Bacteria|Proteobacteria|Gammaproteobacteria|Pseudomonadales|Pseudomonadaceae
*Peptococcaceae*	OSTPu	Bacteria|Firmicutes|Clostridia|Clostridiales|Peptococcaceae
*Comamonadaceae*	OSTPu	Bacteria|Proteobacteria|Betaproteobacteria|Burkholderiales|Comamonadaceae
*Geobacteraceae*	OSTPu	Bacteria|Proteobacteria|Deltaproteobacteria|Desulfuromonadales|Geobacteraceae
*Syntrophorhabdaceae*	OSTPu	Bacteria|Proteobacteria|Deltaproteobacteria|Syntrophobacterales|Syntrophorhabdaceae
*Gaiellaceae*	Tb	Bacteria|Actinobacteria|Thermoleophilia|Gaiellales|Gaiellaceae
*Caulobacteraceae*	Tb	Bacteria|Proteobacteria|Alphaproteobacteria|Caulobacterales|Caulobacteraceae
*Bradyrhizobiaceae*	Tb	Bacteria|Proteobacteria|Alphaproteobacteria|Rhizobiales|Bradyrhizobiaceae
*Hyphomicrobiaceae*	Tb	Bacteria|Proteobacteria|Alphaproteobacteria|Rhizobiales|Hyphomicrobiaceae
*Sporichthyaceae*	Tp	Bacteria|Actinobacteria|Actinobacteria|Actinomycetales|Sporichthyaceae
*Thermogemmatisporaceae*	Tp	Bacteria|Chloroflexi|Ktedonobacteria|Thermogemmatisporales|Thermogemmatisporaceae
*Sphingomonadaceae*	Tp	Bacteria|Proteobacteria|Alphaproteobacteria|Sphingomonadales|Sphingomonadaceae
*Alcaligenaceae*	Tp	Bacteria|Proteobacteria|Betaproteobacteria|Burkholderiales|Alcaligenaceae
*Intrasporangiaceae*	Tu	Bacteria|Actinobacteria|Actinobacteria|Actinomycetales|Intrasporangiaceae
*Micrococcaceae*	Tu	Bacteria|Actinobacteria|Actinobacteria|Actinomycetales|Micrococcaceae
*Nocardioidaceae*	Tu	Bacteria|Actinobacteria|Actinobacteria|Actinomycetales|Nocardioidaceae
*Burkholderiaceae*	Tu	Bacteria|Proteobacteria|Betaproteobacteria|Burkholderiales|Burkholderiaceae

^†^Column labelled “Habitat” represents the petroleum contaminated environment in which the corresponding taxa (as presented in column labelled “Differentially abundant Taxa”), was found to be significantly differentially abundant by LEfSe using the one class, non-strict test (Please refer to Materials and methods, and Supplementary Table S2 for details). Acronyms represent the following habitats: A: Arctic, C: China oil refineries, I: India oil refineries, M: Mangrove, DWH: Marine sediments, OSC: Oil sands core, OSTPu: Oil sands tailings pond upper, OSTPm: Oil sands tailings pond median, OSTPd: Oil sands tailings pond deep, Tb: Taiga bottom active layer, Tu: Taiga upper active layer, Tp: Taiga permafrost layer.

^‡^Taxonomy is described using the following hierarchy: Kingdom|Phylum|Class|Order|Family|Genus|species.

**Figure 3:**
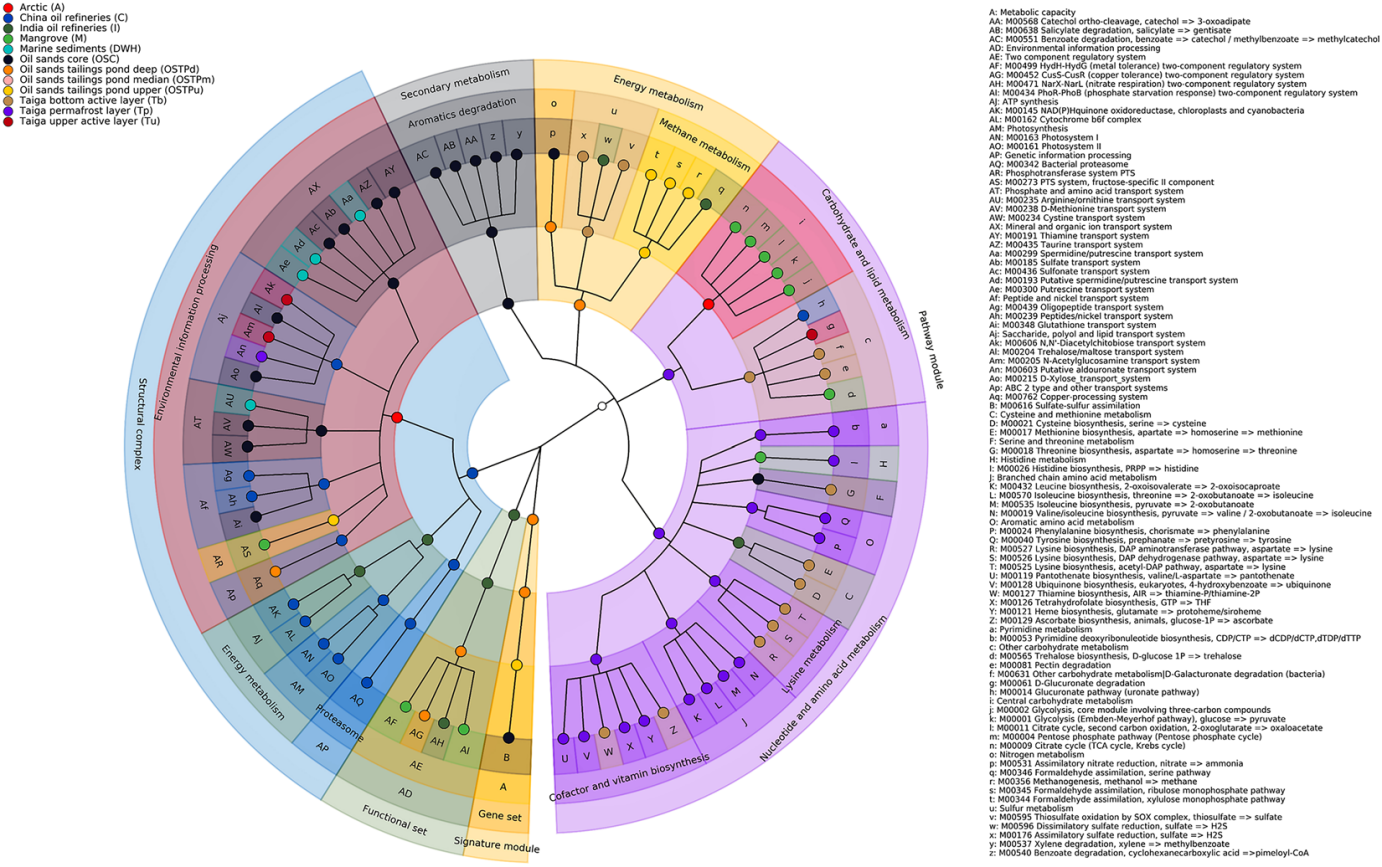
Metabolic reconstruction and functional biomarkers of metagenomes from oil polluted habitats. Cladogram showing a subset of the 4-level KEGG BRITE hierarchical structure denoted by four rings, as inferred against KEGG metabolic modules detected by HUMAnN2 from metagenomic gene family abundance data produced by PICRUSt for all oil contaminated samples. The outermost ring represents KEGG functional modules that have been detected in at least one of the 65 PICRUSt predicted metagenomes as reconstructed by HUMAnN2, while the innermost ring represents the Level 1 KEGG BRITE clades. Differentially abundant KEGG metabolic modules inferred by LEfSe using KEGG module abundance data generated by HUMAnN2 are colored corresponding to the oil contaminated habitat they have been identified to be differentially abundant in (see legend). Circles not differentially abundant in any habitat are colorless. Brackets represent a single KEGG BRITE clade at that Level from which daughter clades originate. KEGG BRITE clades with a single daughter clade are joined using regular branches. Annotations for the KEGG BRITE hierarchy follow an outside-in pattern, wherein Level 1 KEGG BRITE clades are annotated in the outermost section of the cladogram with lower clades annotated further inside ending at the outermost circle in that section of the cladogram. More information on this style of representation can be found elsewhere^28, 36, 83^.

### Metabolic characterization and functional biomarkers of oil contaminated environments

For understanding the metabolic potential of oil polluted environments and identifying differentially abundant functional features, metagenomes were predicted by PICRUSt using 16S rRNA gene amplicon data analyzed in mothur. Predicted proteins were classified as KEGG orthologs (KOs) resulting in the identification of 7020 KOs across all samples. Metabolic reconstruction of metagenomes predicted by PICRUSt was carried out in HUMAnN2, which detected 585 KEGG modules across all samples. Among these functional modules, 19 functional modules were present across all samples at a coverage of >90% and were identified as core modules (Table 4, Supplementary Fig. S4, Supplementary Table S4). Most of the core modules identified are essential for sustenance of prokaryotic life in the environment, such as translation (M00178), central carbon metabolism (M00149), ATP synthesis (M00153, M00157) and nucleotide and amino acid metabolism (M00005, M00020). Rest of the core modules identified were found to be involved in various kinds of transport systems for cations, nutrients and peptides including iron, phosphate, nickel, and amino acids (M00188, M00222, M00223, M00236, M00237, M00239, M00240, M00250, M00254, M00255, M00256, M00258, M00320) (Table 4, Supplementary Fig. S4, Supplementary Table S4). This is important, since these resources are generally present in limiting quantities in nature and often determine the survival and proliferation of microbes in the environment. Additionally, transport systems for lipopolysaccharide (LPS), a principal component of the gram-negative bacterial cell wall, were also understandably identified as core modules and included KEGG functional modules for export of LPS across both cytoplasmic (M00250) and outer membranes (M00320) (Table 4, Supplementary Fig. S4, Supplementary Table S4). Furthermore, 56 differently covered functional modules were detected across all oil contaminated samples (Supplementary Fig. S4, Supplementary Table S4). Among these, five modules were completely covered in only one sample while being absent in all others (Supplementary Fig. S4, Supplementary Table S4). These included structural complexes for Manganese/Iron transport (M00243), bacterial proteasomes (M00342) and putative aldouronate transport (M00603), all of which were completely covered only in the C samples (Supplementary Fig. S4, Supplementary Table S4). This indicates that bacteria in the C site are better equipped for transport of metallic cations, peptide utilization and uptake of plant derived aldouronates than other sites. Furthermore, the presence of a complete complement of D-Xylose transport system (M00215) in the C site also indicates possible bacterial access to hemicellulosic plant material at this site (Supplementary Fig. S4, Supplementary Table S4). Additionally, glutamate transport system (M00233) was completely covered in only the A site, and RstB-RstA stress response two component system (M00446) at the OSC site (Supplementary Fig. S4, Supplementary Table S4). The bacteria at A site, thus are possibly more capable of utilizing glutamate for growth, while resident bacteria in the OSC are conceivably better furnished with stress response mechanisms critical in environmental adaptation and survival.

**Table 4:** Core modules shared between habitats as detected by HUMAnN2.

Module ID	Definition of modules in KEGG
M00005	PRPP biosynthesis, ribose 5 P = > PRPP
M00020	Serine biosynthesis, glycerate-3P = > serine
M00149	Succinate dehydrogenase, prokaryotes
M00153	Cytochrome d ubiquinol oxidase
M00157	F-type ATPase, prokaryotes and chloroplasts
M00178	Ribosome, bacteria
M00188	NitT/TauT family transport system
M00222	Phosphate transport system
M00223	Phosphonate transport system
M00236	Putative polar amino acid transport system
M00237	Branched-chain amino acid transport system
M00239	Peptides/nickel transport system
M00240	Iron complex transport system
M00250	Lipopolysaccharide transport system
M00254	ABC-2 type transport system
M00255	Lipoprotein-releasing system
M00256	Cell division transport system
M00258	Putative ABC transport system
M00320	Lipopolysaccharide export system

In addition to differently covered functional modules, 414 KEGG modules were detected to be differentially abundant in at least one of the 12 contaminated environments (Fig. 3, Supplementary Fig. S3, Supplementary Tables S3 and S6). The largest number of differentially abundant modules were attributed to the OSC samples (70) while the least (8) were attributed to the OSTPm samples (Supplementary Fig. S3, Supplementary Tables S3 and S6). The detection of a higher number of differentially abundant modules in the OSC samples is possibly due to its highly extreme environment as compared to other samples, leading to sequestration of several convenient functions to optimize the use of available resources and counteract distinct environmental stress conditions. On the contrary, similar to the result for taxonomic biomarkers, the least number of differential functional modules were detected in an OSTP sample (OSTPm), with the penultimate spot occupied by Tu samples (13) (Supplementary Fig. S3, Supplementary Tables S3 and S6). As explained before, this is not surprising since both taiga and OSTP samples share comparatively greater similarity between their habitats leading to an overlap of functional capabilities and hence, fewer unique and over-represented functional modules. Most of the modules for metabolism of aromatic hydrocarbons such as xylene degradation (M00537), toluene degradation (M00539), benzoate degradation (M00540 and M00551), salicylate degradation (M00638) and catechol ortho-cleavage (M00568) were significantly associated with the OSC samples (Fig. 3, Supplementary Fig. S3, Supplementary Tables S3 and S6). A number of structural complexes implicated in photosynthesis were found to be differentially abundant in C samples, which included Photosystems I and II (M00163, M00161), the cytochrome b6f complex (M00162) and NADP(H): Quinone oxidoreductase for chloroplasts and cyanobacteria (M00145) (Fig. 3, Supplementary Fig. S3, Supplementary Tables S3 and S6). Additionally, a plethora of amino acid biosynthesis modules were detected as functional biomarkers in the taiga samples. For example, three different KEGG modules for lysine biosynthesis (M00525-M00527), and one each for threonine, methionine and cysteine biosynthesis (M00018, M00017, M00021) were significantly abundant in Tb samples while KEGG modules for valine/isoleucine, phenylalanine, tyrosine, leucine and isoleucine biosynthesis (M00019, M00024, M00026, M00040, M00432, M00535, M00570) were over-represented in Tp samples (Fig. 3, Supplementary Fig. S3, Supplementary Tables S3 and S6). The taiga samples also exhibited an over-representation for modules involved in the biosynthesis of vitamins and cofactors such as heme, pantothenate, ubiquinone, tetrahydrofolate, thiamine and ascorbate (M00127, M00129, M00121, M00119, M00128, M00126) (Fig. 3, Supplementary Fig. S3, Supplementary Tables S3 and S6).

**Figure 4:**
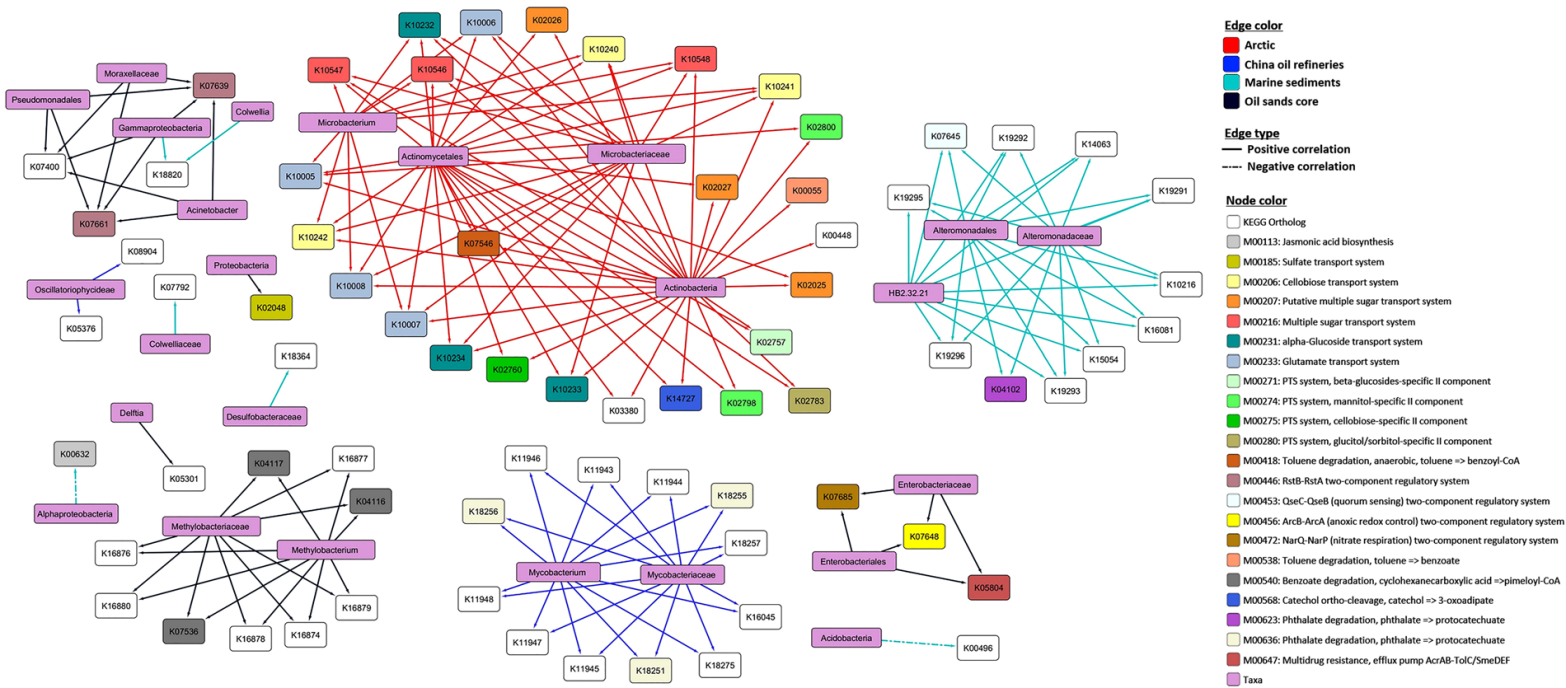
Subset of significant correlations exhibited between KEGG orthologous gene families and bacterial clade abundances. Spearman correlations were calculated between KEGG orthologous gene families and phylotypes at any taxonomic level from phylum to OTU within 4 oil polluted habitats (habitats with six or more samples). A subset of significant associations with correlation >0.7 and p-value < 0.001 reaching a Benjamini-Hochberg false discovery rate < 0.01 are shown here. Taxonomic clades are represented in rectangles with a light purple background and KEGG orthologs are depicted in rectangles with white background (see legend). KEGG orthologs are colored according to corresponding KEGG modules, wherever applicable (see legend). Correlations for each habitat is depicted using different colors (red, Arctic; blue, China oil refineries; turquoise, Marine sediments; midnight blue, Oil sands core) with positive and negative associations represented by continuous and broken arrow lines respectively (see legend).

Overall, all the sites were found to harbor a variety of differentially abundant modules dedicated to the transport of saccharides, polyols, peptides, metallic cations, vitamins, amino acids, mineral ions, organic ions, lipids and phosphate, underlining the large genetic investment of resident bacteria in the processing of environmental information specific to the said site (Fig. 3, Supplementary Fig. S3, Supplementary Tables S3 and S6). However, while differentially over-represented transport systems for saccharides, polyols and lipids were almost ubiquitously detected, significantly associated transport systems for other substrates as phosphates, amino acids, peptides and organic ions were restricted to certain sites. This may indicate differential availability of these nutrients resulting in preferential dependence on certain substrates acquired from the environment and may contribute to the characteristically different nature of the bacteriomes under consideration. Several differentially abundant biosynthetic pathways for sugars, amino acids and vitamins were also detected along with a great diversity of two component systems catering to a range of functions such as stress and redox response, quorum sensing, chemotaxis and heavy metal tolerance across all sites (Fig. 3, Supplementary Fig. S3, Supplementary Tables S3 and S6). Additionally, some modules for atypical energy metabolism as denitrification, dissimilatory nitrate reduction and dissimilatory sulfate reduction were also detected to be differentially abundant and may be important biomarkers for the corresponding sites due to their contribution in bacterial respiration (Fig. 3, Supplementary Fig. S3, Supplementary Tables S3 and S6). Finally, several modules describing microbial resistance to antibiotics and antimicrobial peptides were detected to be over-represented at all sites (Supplementary Tables S3 and S6). This is probably due to the method of ancestral state reconstruction used by PICRUSt for genome prediction, that leads to these genes being predicted for consequent metagenomes if input 16S rRNA data includes hits from bacteria known to have antibiotic resistance genes. The possession and even expression of these genes probably will not have a significant selective advantage in environments already undergoing natural selection due to oil pollution. However, these genes can be contributed by potential pathogens, some of which are known to be prolific degraders of petroleum hydrocarbons and therefore warrants precautions to be taken for further *ex*-*silico* studies.

### Associations between bacterial taxa and metagenomic gene families

Correlations between bacterial abundance and functions enriched at different sites were evaluated following a statistical strategy similar to the approach described by Segata *et al*.^36^ (see Materials and Methods: Detection of associations between metagenomic gene families and taxa). The results indicated strong and significant associations between a number taxonomic clades and metagenomic gene families predicted by PICRUSt (Fig. 4). A subset of these significant correlations included strong associations between previously detected taxonomic biomarkers and over-represented KOs for each site, which further confirmed the identified taxonomic biomarkers. For example, photosynthetic structural complex genes *cpeA* (K05376) and *psb28-2* (K08904), found to be differentially abundant in C samples exhibited strong positive association with an over-represented cyanobacterial order, *Oscillatoriophycideae* (Spearman correlation >0.7, *P*-value < 0.001) (Fig. 4). Additionally, an array of genes related to polycyclic aromatic hydrocarbon degradation such as *nidABD*, *phdFGIEK*, and *phtAaBC* (K11943-48, K18251, K18255-57, K18275) were differentially abundant in C samples and also significantly positively correlated to known polyaromatic hydrocarbon degrader and taxonomic biomarker *Mycobacterium* (Spearman correlation >0.7, *P*-value < 0.001)^35^ (Fig. 4). In other observations, taxonomic biomarkers *Microbacterium* and *Microbacteriaceae* showed positive correlation with several genes associated with the transport of sugars, saccharides and amino acids such as *ggtB*-*D* (K10232-34), *cebE*-*G* (K10240-42), *chvE* (K10546), *gguA*-*B* (K10547-48), and *gluA*-*D* (K10005-08) in arctic samples (Spearman correlation >0.7, *P*-value < 0.001) (Fig. 4). Hydrocarbon degradation genes like *pcaG* (K00448), *bbsH* (K07546) and *pcaL* (K14727) were significantly correlated to class *Actinobacteria* in a positive manner in the same samples (Spearman correlation >0.7, *P*-value < 0.001) (Fig. 4). In the DWH samples, *Colwelliaceae*/*Colwellia* exhibited positive correlations with both anaerobic C4-dicarboxylate transporter (*dcuB*; K07792) and 2-oxopent-4-enoate/cis-2-oxohex-4-enoate hydratase (*bphH*, *xylJ*, *tesE*; K18820), an enzyme implicated in oligosaccharide metabolism (Spearman correlation >0.7, *P*-value < 0.001) (Fig. 4). Additionally, genus *HB2.32.21*, associated positively with several genes involved in alginate production (*alg44*, *algJXKFE*; K19291-3, K19295-6, K16081), flagellar synthesis/chemotaxis (*qseC*; K07645) and aminobenzoate metabolism gene regulation (*feaR*; K14063) (Spearman correlation >0.7, *P*-value < 0.001) (Fig. 4). *Acidobacteria* however, was found to be negatively correlated with the *alkB1-2* gene (K00496) coding for alkane-1-monooxygenase (Spearman correlation <−0.7, *P*-value < 0.001) (Fig. 4) in the DWH samples. This can be due to a possible negative effect of crude oil contamination on the abundance of *Acidobacteria* at DWH sites. This observation is also corroborated by a conspicuous absence of any taxonomic biomarker from this phylum for DWH samples (Supplementary Fig. S2, Supplementary Tables S2 and S5) and an absent contribution for hydrocarbon degradation capabilities (Supplementary Fig. S5). In OSC samples, positive correlations were detected between *Methylobacterium* and genes involved in furfural degradation (*hmfABCDEF*; K16874-80) and benzoate degradation (*aliAB*, *badI*; K04116-17, K07536) (Spearman correlation >0.7, *P*-value < 0.001) (Fig. 4). *Methylobacterium*, although an aerobe^37^, has been shown to possess anaerobic benzene degradation genes in the genome annotation for *Methylobacterium extorquens* PA1 in the KEGG (http://www.genome.jp/kegg-bin/show_pathway?mex01220). Furthermore, several two-component systems (TCS) showed strong positive association with *Acinetobacter* and *﻿Enterobacteriaceae* in the OSC samples. *Acinetobacter* was positively correlated with the enrichment of RstA/RstB stress response TCS (K07639, K07661), while *Enterobacteriaceae* showed affirmative relationships with the aerobic stress response sensor kinase ArcB (K07648) and nitrate/nitrite response regulator NarP (K07685) (Fig. 4).

To further understand the association of bacterial clades with gene families specifically with respect to hydrocarbon degradation, we categorized all taxa contributing to the abundance of genes known to be involved in hydrocarbon degradation at the family and genus level (Supplementary Fig. S5). The results showed that significant differences existed between major contributors to the abundance of hydrocarbonoclastic genes at different sites. For example, abundance for alkane-1-monooxygenase (K00496) was contributed mainly by *Alteromonadaceae* in DWH samples, *Comammonadaceae* in I, *Mycobacteriaceae* and *Nocardiaceae* in C, *Propionibacteriaceae* in OSC, and a mixture of *Acetobacteraceae*, *Mycobacteriaceae*, *Nocardiaceae* and *Rhodospirillaceae* in the taiga samples (Supplementary Fig. S5). Similarly, for protocatechuate-4,5-dioxygenase (K04100-01), *Alteromonadaceae* was again the major contributor for DWH samples, *Comamonadaceae* and *Methylobacteriaceae* for OSC, *Rhodocyclaceae* for I, *Rhodocyclaceae* and *Comamonadaceae* in OSTP, and *Comamonadaceae* and *Bradyrhizobiaceae* for taiga samples (Supplementary Fig. S5). These differences in patterns observed at the family level, were even more stark at higher resolutions i.e. genus level, thus effectively differentiating such metagenomic contributors from site to site. This was best demonstrated for the hydrocarbonoclastic gene catechol-1,2-dioxygenase (K03381), for which *Alteromonadaceae* was found to be the most dominant contributor in both DWH and M samples (Supplementary Fig. S5). However, at the genus level, it was seen that while *HB2.32.21* was the dominant effector organism in DWH samples, *Marinobacter* was the largest metagenomic contributor for K03381 in the M samples (Supplementary Fig. S5).

### Bacterial interactions in oil polluted environments

**Figure 5:**
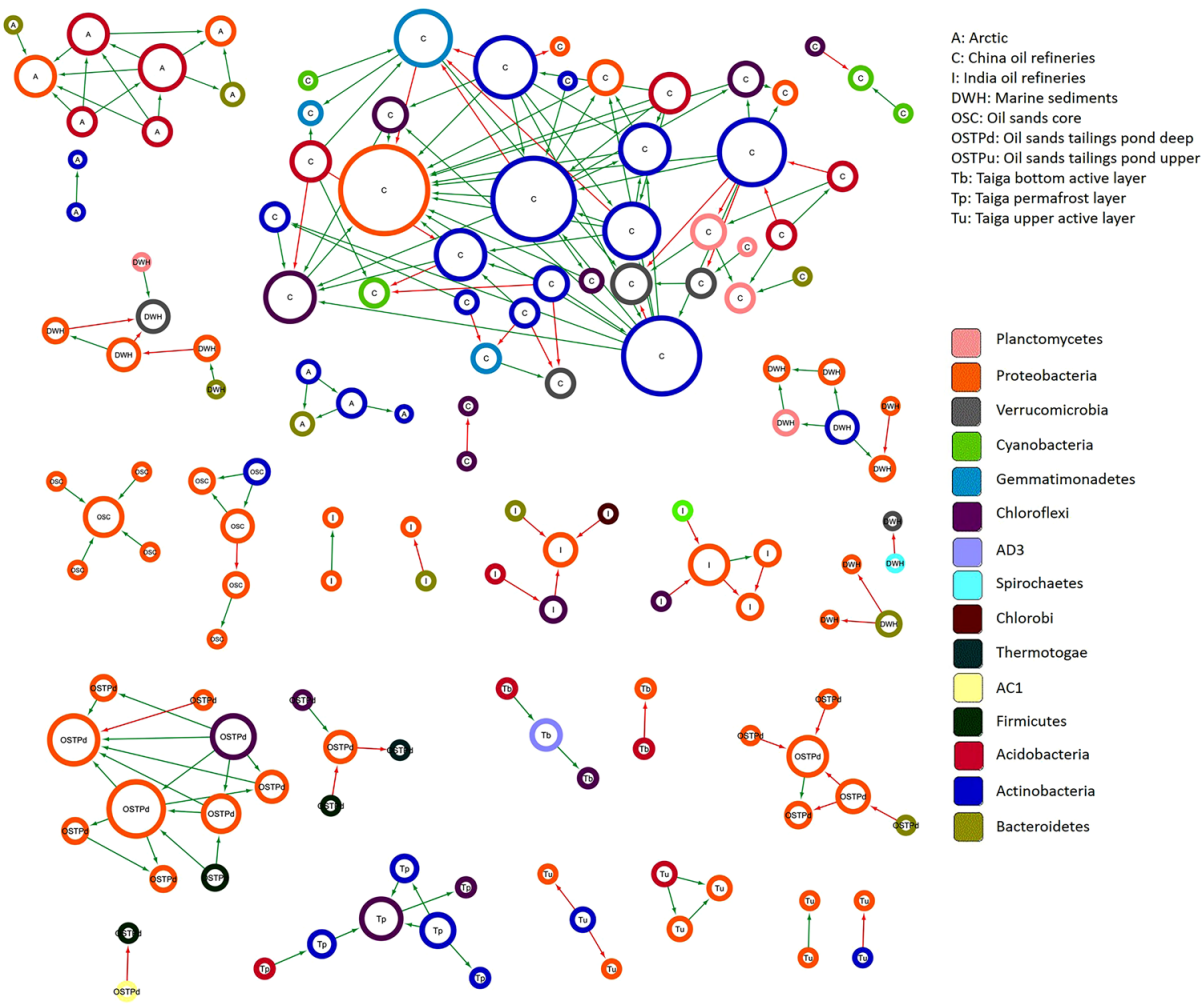
SparCC network plot of global bacterial interactions in individual oil polluted habitats. Significant bacterial associations captured by SparCC (*p*-value < 0.01) with an absolute correlation magnitude of ≥0.6 are presented. Nodes represent detected phylotypes (OTU clustered at 97% similarity) involved in either significant co-occurrence (green edges) or co-exclusion (red edges) relationships. Border coloration depicts taxonomic affiliation of nodes at the phylum level (see legend). Node size is proportional to the connectivity of the node (both positive and negative relationships).

To further understand complex ecological relationships in oil polluted environments, bacterial association networks were deduced from estimated taxonomic profiles. For our study, we concentrated on individual oil polluted habitats with 4 or more samples, i.e. arctic, China oil refineries, oil sands core and so on. The resulting bacterial correlation networks, inferred at or above the species level, constituted 186 significant relationships among 115 phylotypes (OTUs clustered at 97% similarity) (*P* < 0.01) (Fig. 5, Supplementary Table S8). Among the associations deduced to be significant, 72.58% were detected to share positive correlations while the rest shared antagonistic relationships. Almost half of the co-occurrence patterns identified (46%) were observed between bacteria of the same phyla while more than three-quarters of all negative correlations (78%) were detected between bacteria belonging to distinct phyla (Fig. 5, Supplementary Table S8). Interestingly, bacterial taxa affiliated to phylum *Actinobacteria* were involved in more same phylum, co-occurrence interactions (*i* = 25) than bacteria from phylum *Proteobacteria* (*i* = 23), even though the mean relative abundance of *Proteobacteria* was much higher than *Actinobacteria* (Fig. 1,﻿ Fig. 5, Supplementary Table S8) across all habitats. When computed at the class level, nearly 20% of all positive correlations were observed among bacteria belonging to the same class while almost all co-exclusion patterns observed (94%) were between dissimilar classes (Fig. 5, Supplementary Table S8). Thus, our results from the inferred bacterial correlation networks indicated that, co-occurrence of phylotypes was closely related to sharing of evolutionary lineage. For example, in the OSC habitat, phylotypes belonging to proteobacterial family *Oxalobacteraceae* shared positive pairwise correlations with *Moraxellaceae* and *Enterobacteriaceae* phylotypes, both of which belong to phylum *Proteobacteria* (Fig. 5, Supplementary Table S8). Additionally, similar co-occurrence patterns were observed between phylotypes attributed to families belonging to the order *Actinomycetales* in the C samples. Positive pairwise associations were observed in C samples between phylotypes from families *Micrococcaceae* and *Nocardioidaceae*, *Intrasporangiaceae* and *Mycobacteriaceae* with *Solirubrobacteraceae*, and *Gaiellaceae* and *Geodermatophilaceae* with *Microbacteriaceae*, all of which belong to order *Actinomycetales* (Fig. 5, Supplementary Table S8). Furthermore, genera *Arthrospira* and *Phenylobacterium*, both of which belong to family *Caulobacteraceae*, co-occurred in the Tu samples (Fig. 5, Supplementary Table S8). Conversely, bacteria without evolutionary commonalities tended to be negatively correlated. For example, in DWH samples, antagonistic relationships were observed between phylotypes belonging to family *Flavobacteriaceae* from phylum *Bacteroidetes* and proteobacterial families *Desulfuromonadaceae* and *Desulfobulbaceae* (Fig. 5, Supplementary Table S8). Similarly, mutual exclusion was observed between phylotypes belonging to family *Weeksellaceae* of phylum *Bacteroidetes* and *Xanthomonadaceae* of phylum *Proteobacteria* in I samples. Additionally, negatively correlated associations were observed between phylotypes belonging to genera *Dietzia* and *Chthoniobacter* in C samples, the former of which belongs to phylum *Actinobacteria* and the latter to phylum *Verrucomicrobia* (Fig. 5, Supplementary Table S8).

Most phylotype interactions observed in microbial association networks lack any empirical evidence to support the natural presence of the same. However, the nature of some interactions may be predicted based on the biological proclivities of the taxa involved. For example, *Cupriavidus* shared a significantly negative correlation with *Herminiimonas* in OSC samples (Fig. 5, Supplementary Table S8). This antagonistic activity can be an outcome of the non-obligate predatory nature of *Cupriavidus* ^38^, which can be preying on *Herminiimonas*. Additionally, *Candidatus Koribacter* and *Devosia* were involved in a significantly positive interaction in Tb samples (Fig. 5, Supplementary Table S8). It can be speculated that both species, which are known degraders of plant polymers^39, 40^ may be involved in a mutually beneficial relationship for achieving completion of such an objective. Such a possibility is supported by the detection of differentially abundant KEGG modules for degradation of pectin (M00081) and D-galacturonate (M00631) (Supplementary Fig. S3, Supplementary Tables S3 and S6) in Tb samples. An opposite, significantly antagonistic relationship was observed between the chemoorganotrophic *Microbacterium* and *Chthoniobacteraceae*, both of which are well known degraders of plant polymers^41, 42^, in the C samples. It can be argued that both taxa may be competing for similar resources in the environment and therefore are engaged in a competitive relationship (Fig. 5, Supplementary Table S8). Incidentally, a KEGG module for degradation of plant-polymer component glucuronate (M00014) was also identified to be differentially abundant in these samples (Supplementary Fig. S3, Supplementary Tables S3 and S6). *Pseudonocardina* was found to share significantly negative associations with both *Novosphingobium* and *Bradyrhizobiaceae* in Tu samples (Fig. 5, Supplementary Table S8). Such an interaction can be theorized to happen due to an antimicrobial activity that *Pseudonocardina* is known to have^43^. These interpretations show that SparCC computed taxonomic correlations can therefore provide reasonably relevant targets for hypothesis building and evaluation of co-operative and competitive interactions in the environment.

## Discussion

The advent of next-generation sequencing (NGS) technologies has revolutionized investigative approaches into microbial processes. This has led to re-exploration of well-known microbial processes as the nitrogen cycle^44^, methane metabolism^45^, sulfur cycle^46^, heavy metal remediation and petroleum bioremediation^47^ along with examination of exotic and extreme environments such as deep-sea hydrothermal vents^48^, cold deserts like Antarctica^49^ and remote cave systems^50^. As a result, a large body of work has accumulated over the years on the microbiological study of hydrocarbon degradation using NGS technologies^51^. Most of these studies employed 16S rRNA based amplicon sequencing while some used metagenomic shotgun sequencing for their enquiries. Although some of these studies have concentrated on prediction of potential biomarkers for oil pollution in certain environments^52, 53^, no investigative effort has been undertaken to use the large amounts of data generated in oil pollution studies across the world to review, validate and further these studies. In the present study, we describe taxonomic and functional characteristics of oil polluted environments across the world to understand the differences and similarities that exist between them. Additionally, we infer several potential biomarkers, both taxonomic and functional, along with correlation networks, which provide new insights into the process of oil bioremediation through identification of important taxa and metabolic pathways in different oil polluted ecosystems. To this end, we have used 65 16S rRNA datasets from different studies across the world (Table 1, Supplementary Table S1), including 4 datasets generated in this study, and carried out robust *in-silico* analysis with recently developed bioinformatics tools to compare and contrast the same. The principal features and findings of our study are discussed below.

### Validation of bioinformatic pipeline

To our knowledge this is the only study that has congregated existing 16S rRNA gene NGS data generated during experiments on hydrocarbon pollution in different habitats around the world to deduce possible biomarkers and associated bacterial characteristics and interactions. The bioinformatics pipeline we designed to analyze this data employed PICRUSt, which is a recently developed tool that uses 16S rRNA data to predict metagenomes along with LEfSe which predicts potential biomarkers and HUMAnN2 for metabolic reconstruction of PICRUSt predicted metagenomes. It is to be noted however, that KEGG orthologs and KEGG module databases for PICRUSt and HUMAnN2 were meticulously updated (previously PICRUSt KEGG databases included KEGG orthologs only up to K15039 and HUMAnN had a KEGG module database represented only up to M00378) to include currently available definitions of KEGG functional modules and represent the metabolic terrain of environmental habitats in totality, especially with respect to hydrocarbon degradation (several KEGG modules for hydrocarbon degradation were absent in the original database). Prediction of metagenomes for petroleum hydrocarbon contaminated habitats will therefore be incomplete without the use of the database developed in this study and may constitute a gross misrepresentation of the said environments.

It must be noted that although promising, studying of environmental systems and processes through prediction of metagenomes from 16S rRNA data is bound by certain limitations. The main disadvantages of this method include: (i) The requirement of an updated database for prediction of metagenomes from 16S rRNA data and consequent estimation of metabolic pathways present. An obsolete database will lead to identification of a partial metagenome and huge loss of information leading to a steep drop in the quality of conclusions drawn. To elaborate in brief, the metagenome prediction relies on sequenced genomes for creation of a prediction database and the availability of sequenced genomes can therefore dictate the quality of the prediction database created. Thus, non-updated prediction tools will only be able to predict a more complete metagenome for highly studied environments like the human gut as opposed to scarcely studied habitats such as hypersaline mats. Fortunately, focus on environmental research and large depositions of environmental sequences in public databases in recent years has largely bridged this divide. However, caution must be taken considering the type of habitats being analyzed, (ii) Due to the inherently predictive nature of the process, some caution must be exercised while interpreting results. This can be done by concentrating primarily on identification of patterns rather than on single points of conclusion i.e. relying on several proteins or pathways belonging to the same metabolic grouping to derive a conclusion rather than on the presence of one. Additional *ex silico* work is advised in the latter case. However, for obvious reasons, conclusions can be drawn with much more confidence from 16S rRNA gene datasets that have already been published and reviewed and when using an updated prediction database, (iii) Although the process is robust and has been proven to be much more than a hypotheses building exercise in a number of studies^24^, conclusions drawn from such bioinformatic pipelines require *ex silico* confirmation, similar to any other kind of sequencing based experiment.

To confidently interpret and infer results obtained in this study, we validated our findings in both taxonomic and functional aspects. For example, a complete convergence of conclusion was observed when comparing our inferred taxonomic compositions and biomarkers with the findings of Mason *et al*.^54^ for the marine sediment samples. Our analysis of the marine sediment samples identified a highly dominant Gammaproteobacterial genus, *HB2-32-21* (Greengenes OTU ID 248394) belonging to the family *Alteromonadaceae* (Supplementary Table S2) as a taxonomic biomarker (Supplementary Fig. S2, Supplementary Tables S2 and S5) and as a significant contributor of hydrocarbon degradation capabilities for the habitat (Supplementary Fig. S5). Additionally, *Colwelliaceae* and *Rhodobacteraceae* were also detected as over-represented taxonomic biomarkers at the Macondo oil contaminated DWH sample sites (Supplementary Fig. S2, Supplementary Tables S2 and S5) with the latter contributing heavily to the abundance of the hydrocarbonoclastic enzyme, alkane-1-monooxygenase (Supplementary Fig. S5). Understandably, all aforementioned taxa were also identified by Mason *et al*. as exceptionally abundant in oil contaminated samples as compared to uncontaminated marine sediment samples. To further this validation, we compared the relative abundances of all taxonomic biomarkers identified in this study for DWH samples with relative abundances inferred by Mason *et al*. To achieve this, we subjected the DWH data to 16S rRNA sequence analysis as described previously^54^ and plotted relative abundances for each study using boxplots generated in R by ggplot2 (Supplementary Fig. S6). Our results show that, relative abundances inferred in both studies, across all taxonomic biomarkers were exceptionally similar. The only departures from this observation were constituted by the taxa *Chryseobacterium*, *Xanthomnadales*, *Xanthomonadaceae* and *Weeksellaceae* (Supplementary Fig. S6). This can be explained by the differences in an updated 16S rRNA SILVA reference database used in the present study against the then Greengenes October 2012 release used by Mason *et al*., where larger number of representative sequences for these taxa are present in current databases thereby allowing appropriate recognition of the same. Indeed, the highly abundant *Xanthomonadales* and *Xanthomonadaceae* were not identified as important taxonomic indicators by Mason *et al*. Additionally, we compared mean relative abundances for 110 KEGG orthologs implicated in xenobiotic degradation as defined by KEGG Pathways^22^ and a subset of the same in terms of counts per million for both studies (Supplementary Fig. S6). Our observations show that quantitative dispositions of KOs for PICRUSt predicted metagenomes generated in this study and shotgun sequenced metagenomes produced by Mason *et al*. were largely in agreement and reasonably comparable (Supplementary Fig. S6). The observed consistencies of results obtained in this study with those by Mason *et al*. thus provided appropriate validation of the employed bioinformatic pipeline besides furthering their study by providing new insights.

Important similarities were also discovered between conclusions inferred by An *et al*.^12^ and our study, regarding the OSC datasets. In the original study by An *et al*.^12^, the oil sands core was deduced as an aerobic environment with limited oxygen ingress in specific regions leading to regional anaerobiasis. This theory of intermittent oxygen infusion in sections of the oil sands core was strongly supported by the detection of both aerobic and anaerobic pathways of hydrocarbon degradation in the oil sands core. For example, in the OSC samples we detected differentially abundant KEGG modules for aerobic degradation of different hydrocarbons such as xylene, benzoate, toluene and cumate including metabolism of corresponding intermediates such as salicylate and catechol (M0537-40, M00568, M00638) (Fig. 3, Supplementary Fig. S3, Supplementary Tables S3 and S6)^55^ alongside a module implicated in anaerobic degradation of benzoate (M00551) (Fig. 3, Supplementary Fig. S3, Supplementary Tables S3 and S6)^56^. This further validation of our bioinformatic pipeline through uniformity of results obtained, indicated the robustness and reliability of the applied computational approaches for interpretation of environmental 16S rRNA sequence datasets.

### Metabolic reconstruction of oil polluted metagenomes reveals important functional pathways in petroleum hydrocarbon contaminated habitats

PICRUSt was used to predict metagenomes from 16S rRNA data and KEGG metabolic modules were detected using HUMAnN2 in order to elaborate the functional landscape of each oil polluted environment. We identified 19 core modules which were present across all habitats with a coverage of >90%. Most of these are involved in processes central to survival of bacteria in the environment. Furthermore, to identify preferential genetic investments among resident bacteria at each habitat, differentially abundant KOs and KEGG modules were detected through LEfSe. Consequently, we analyzed over-represented KOs and KEGG modules across all habitats to identify broad metabolic signatures that may be indicative of important areas of genetic expenditure, especially outside hydrocarbon degradation.

We identified several differential functional pathways dedicated to the transport of certain sugars or lipids, biosynthesis of biomolecules, stress response, quorum sensing, metabolism of polysaccharides, assimilation and respiration of sulphur and/or nitrogen compounds besides hydrocarbon degradation, across all sites. For example, a collection of putrescine transport complexes (M00193, M00299, M00300) and a transport system for arginine/ornithine (M00235) were detected to be differentially abundant for the DWH samples (Fig. 3, Supplementary Fig. S3, Supplementary Tables S3 and S6). This sequestration of putrescine transporters along with transporters for ornithine, which is readily converted by ornithine decarboxylase to putrescine indicates a significant dependence of marine bacteria at oil polluted DWH sites on putrescine. This can be explained by the crucial role putrescine plays in bacteria as an osmoprotectant^57^, and therefore its prevalence in a marine oil polluted environment. Similarly, availability and possible use of carbon sources besides hydrocarbons was apparent in the C samples. The differential presence of a complete complement of D-xylose transport system (M00215) and a putative aldouronate transport system (M00603) along with the over-representation of KEGG module M00014 (Glucuronate pathway), strongly indicated that besides petroleum hydrocarbons, plant wastes may be available as possible sources of energy for resident soil bacteria at the China oil refineries site (Fig. 3, Supplementary Fig. S3, Supplementary Tables S3, S4 and S6). Bacteria are known to extracellularly depolymerize methylglucuronoxylan, a polysaccharide made of xylose that constitutes the hemicellulosic component of terrestrial plants^58^ leading to the production of aldouronates and xylooligosaccharides. These compounds are taken up and normally converted intracellularly to fermentable xylose, leading to generation of energy along with ethanol. Alternatively, D-xylose can also be directly taken up from the environment. Also, two structural complexes for transport of peptides/oligopeptides (M00239 & M00439) were detected to be differentially abundant in the C samples along with bacterial proteasomes (M00342) (Fig. 3, Supplementary Fig. S3, Supplementary Tables S3 and S6). This indicates that acquisition of environmental peptides and consequent proteasomal degradation of the same, may be a dominant mechanism for obtaining amino acids for assimilatory purposes in the C samples.

Interestingly, the DesK-DesR two-component system (M00479), implicated in regulation of the *des* gene coding for a desaturase that helps control the saturation state of membrane lipids at low temperatures^59^ was detected to be differentially abundant in the arctic samples (Fig. 3, Supplementary Fig. S3, Supplementary Tables S3 and S6). Furthermore, the FitF-FitH two component system (M00771), responsible for insecticidal toxin regulation^60^, was over-represented in the urban site of the Indian oil refinery samples (Fig. 3, Supplementary Fig. S3, Supplementary Tables S3 and S6). This makes sense, since it has previously been shown that relatively higher amount of heat generation in cities compared to rural areas leads to sequestration of insects in urban areas^61^. Sulfur assimilation in bacteria (M00616) was detected to be differentially abundant in OSC samples along with a number of modules dedicated to transfer of sulfur compounds (M00185, M00234, M00238, M00348, M00435-36) indicating a conceivably large genetic investment in scavenging and metabolism of sulfur compounds in this site (Fig. 3, Supplementary Fig. S3, Supplementary Tables S3 and S6). Differential presence of a transport module for thiamine (M00191), which is required for assimilation of sulfonate compounds, adds further credence to this notion (Fig. 3, Supplementary Fig. S3, Supplementary Tables S3 and S6). Additionally, differential detection of assimilatory nitrate reduction module (M00531) also indicates the capability of the OSC bacteriome to use such compounds for their proliferation (Fig. 3, Supplementary Fig. S3, Supplementary Tables S3 and S6). Sulfate and nitrate ions are also important molecules in anaerobic respiration, and therefore may play crucial roles in bacterial survival in the anaerobic regions of the OSC. Interestingly, reduction of nitrate has been reported to be closely linked to anaerobic degradation of benzene and concomitant growth^62^, functional modules for both of which have been differentially detected in OSC samples (Fig. 3, Supplementary Fig. S3, Supplementary Tables S3 and S6). Unlike other oil polluted sites, several hydrocarbonoclastic modules were differentially detected in the OSC samples (see previous section), whereas only two transport systems for small sugars (M00204, M00215) and no major polysaccharide metabolism and/or transport pathways were detected to be over-represented (Fig. 3, Supplementary Fig. S3, Supplementary Tables S3 and S6). This indicates large adaptations in the bacteriome of the OSC directed at the utmost utilization of available petroleum hydrocarbons against a possibly restricted supply of other carbon sources leading to the large clustering of differentially abundant hydrocarbon degradation pathways in the OSC samples.

Multiple functional modules related to methane metabolism, both methanogenic and methanotrophic, were detected to be over-represented for the OSTP samples. For example, methanogenesis (M00356) was over-represented in OSTPu, along with methane assimilation modules M00344-45 and M00608 detected to be differentially abundant in OSTPu and OSTPm respectively (Fig. 3, Supplementary Fig. S3, Supplementary Tables S3 and S6). Oil sands tailings ponds are known to be important sources of methanogenesis and of methylotrophy^12^, where deeper regions tend to be highly anaerobic. Additionally, modules for copper processing (M00762) and copper tolerance sensor (M00452) were detected in OSTPd (Fig. 3, Supplementary Fig. S3, Supplementary Tables S3 and S6). This is important, since copper is an essential component of the particulate methane monooxygenase (pMMO), and its availability can therefore determine the survivability of methanotrophs^63^ along with the ratio of soluble and particulate MMO in the environment.

Several KEGG modules dedicated to the biosynthesis of amino acids, vitamins and co-factors were detected to be over-represented in the taiga samples (see above) (Fig. 3, Supplementary Fig. S3, Supplementary Tables S3 and S6). This is indicative of the metabolic versatility of the taiga bacteriomes regarding amino acid, vitamin and co-factor metabolism. Steep enrichment of these KEGG modules in the taiga samples may be due to characteristic environmental conditions of the taiga and requires further studies to decipher the specific reasons behind such an adaptation. The identification and importance of differentially abundant modules for sulfur containing amino acid biosynthesis (M00017, M00021) is further supported by the detection of over-represented sulfur assimilation modules (M00176, M00595) which involve biosynthesis of cysteine and methionine as final/supplementary steps^64, 65^ (Fig. 3, Supplementary Fig. S3, Supplementary Tables S3 and S6). Additionally, capabilities of the taiga bacteriome to degrade complex plant polymers and a possible presence of alternative carbon sources such as pectin and component sugars of other plant polysaccharides can be inferred through the presence of differentially abundant functional modules for pectin degradation (M00081), and uptake and metabolism of other sugar and sugar derivatives such as N-Acetylglucosamine, N, N’-Diacetylchitobiose, D-glucuronate, aldouronates, ﻿and D-galactouronate (M00606, M00205, M00061, M00603, M00631) (Fig. 3, Supplementary Fig. S3, Supplementary Tables S3 and S6).

In the M samples, two component systems for starvation of phosphate (M00434), a limiting nutrient for mangroves^34^ and metal tolerance (M00499) were detected as differentially abundant (Fig. 3, Supplementary Fig. S3, Supplementary Tables S3 and S6). Genetic investment in metal tolerance should be important in M samples as mangroves in Brazil are routinely subjected to pollution from factory effluents^52^. Furthermore, the clustering of differentially abundant central carbohydrate metabolism pathways (M00001-2, M00004, M00009, M00011) along with transport systems for sugars like fructose (M00273) in M samples, indicate the possible availability of simple sugars as carbon sources besides hydrocarbons (Fig. 3, Supplementary Fig. S3, Supplementary Tables S3 and S6) and a concurrent ability to use the same. This observation is also supported by the detection of a differentially abundant module for the synthesis of trehalose (M00565), a known carbohydrate energy storage compound and anti-desiccation agent^66^, from glucose (Fig. 3, Supplementary Fig. S3, Supplementary Tables S3 and S6).

All functional modules for degradation of aromatic hydrocarbons were detected to be differentially abundant in OSC, DWH, taiga and OSTP (in that order) samples (Supplementary Fig. S3, Supplementary Tables S3 and S6). This is probably because these environments tend to be more extreme than other sites described in this study and coupled with oil pollution, the bacterial metabolic pathways in these environments have been further sculpted to rely greatly only on petroleum hydrocarbons for growth. Additionally, sulfate and nitrate utilization modules have been identified in most of these sites, which indicates the ability of these bacteriomes to possibly couple atypical metabolic pathways to anaerobic alkane degradation, as has been previously described^67^. Our results thus indicate that for all habitats, genetic composition of the bacteriome is representative of the immediate environment especially in terms of substrate usage, nutrient availability, energy metabolism, biosynthesis of compounds, and survival strategies including quorum sensing, chemotaxis, and stress response. Our findings reveal pathways differentially important in these oil polluted environments, especially those not related to hydrocarbon degradation and can therefore be used for differentiation between habitats of interest. Further empirical studies will however be required to strengthen these observations and pinpoint functional biomarkers absolutely exclusive to oil polluted environments in specific biomes.

### Taxonomic biomarkers make important contributions to hydrocarbonoclastic and additional functional capabilities in oil polluted environments

Taxonomic clades that are differentially abundant in oil polluted sites used in the present study were inferred using taxonomic profiles generated through analysis of 16S rRNA data in mothur by LEfSe. Additionally, to decipher functional associations of taxonomic clades, direct correlations between KOs and taxa were determined along with metagenomic contributions to hydrocarbonoclastic genes. Furthermore, bacterial co-occurrence and co-exclusion networks were deduced to understand important bacterial interactions in oil polluted sites. Our findings suggest that, taxonomic biomarkers inferred in our study contribute significantly to important functions in the oil polluted metabolic landscape and are often determined by their oil degradation capabilities. For example, biomarkers for DWH samples *HB2*.*32*.*21* and *Alteromonadaceae* (Table 3, Supplementary Fig. S2, Supplementary Tables S2 and S5), were associated with over-represented KOs implicated in alginate biosynthesis (Fig. 4). Moreover, a two-component pathway involved in the regulation of alginate production (M00505) was also differentially abundant in DWH samples (Supplementary Tables S3 and S6). Interestingly, previous studies have shown that alginates provide increased mechanical stability to bacterial biofilms^68^, and can therefore be instrumental in aiding anchorage or adhesion of DWH *Alteromonadaceae*. *HB*.*32*.*21* and *Alteromonadaceae* were found to be important contributors in hydrocarbonoclastic properties of the DWH bacteriome (Supplementary Fig. S5) and the former also exhibited strong associations with regulation of genes for aminobenzoate metabolism through *feaR* (see Results). Furthermore, another taxonomic biomarker identified for DWH samples, *Colwelliaceae*, was closely associated to the anaerobic C4-dicarboxylate transporter DcuB (see Results), which is responsible for transport of molecules as fumarate, succinate and malate^69^. This is important, as it may help the facultatively aerobic *Colwelliaceae* to degrade alkanes anaerobically by addition of fumarates in marine sediments^67^. Similarly, *Mycobacterium* was detected as a biomarker for C samples (Supplementary Fig. S2, Supplementary Tables S2 and S5) and correlated strongly with KOs implicated in degradation of hydrocarbons such as naphthalene, benzoate and phthalate (Fig. 4). Additionally, *Mycobacterium* also made major contributions to the aliphatic hydrocarbonoclastic capabilities of the habitat through the gene alkane-1-monooxygenase (K00496) (Supplementary Fig. S5). Hence, it can be conceived that *Mycobacterium* is one of the most important hydrocarbon degraders in these samples and may have made significant genetic investments for utilization of contaminating crude oil in C samples. *Mycobacterium*, a known human pathogen, is a well-known degrader of petroleum hydrocarbons and has previously been shown to harbor the ability to degrade a variety of aromatic hydrocarbons such as naphthalene, anthracene, phenanthrene, pyrene and so on^35^. Phylum *Cyanobacteria*, a biomarker for C samples (Supplementary Fig. S2, Supplementary Tables S2 and S5), strongly correlated with differentially abundant photosynthetic proteins *cpeA* (K05376) and *psb28-2* (K08904) through an over-represented cyanobacterial order for C samples, *Oscillatoriophycideae* (Fig. 4, Supplementary Fig. S2, Supplementary Tables S2 and S5). Additionally, KEGG modules for photosynthesis such as Photosystems I and II (M00163, M00161), cytochrome b6f complex (M00162) and NADP(H):quinone oxidoreductase for chloroplasts and cyanobacteria (M00145) were also found to be over-represented in C samples (Fig. 3, Supplementary Fig. S3, Supplementary Tables S3 and S6). These observations indicated important, differential and extra-hydrocarbonoclastic contributions of *Cyanobacteria* in C samples.

*Microbacterium*, which is known to be a stringent chemoorganotroph^70^, was identified to be differentially abundant in A samples (Supplementary Fig. S2, Supplementary Tables S2 and S5). *Microbacterium* was found to share close associations with KOs involved in over-represented transport systems dedicated to acquisition of organic compounds such as cellobiose (M00206), alpha-glucosides (M00201), glutamate (M00227, M00233) and multiple sugars (M00207, M00216, M00221) (Fig. 4), which can be used as possible sources of carbon and energy by it and indicates a probable availability of the same in the environment. Phylum *Actinobacteria* and class *Actinobacteria*, which were detected as biomarkers in the A samples, exhibited significant correlations with almost all differentially abundant KOs for A samples including hydrocarbon degrading genes as *pcaG*, phenol-2-monooxygenase, *bbsH*, and *pcaL* (data not shown).

In OSC samples, over-represented taxa *Methylobacterium* was associated with genes involved in degradation of furfural and other hydrocarbons (Supplementary Fig. S2, Supplementary Tables Ss and S5). The presence of the strongly aerobic *Methylobacterium* ^37^ once again reinforces the finding of ample availability of oxygen in the OSC. Interestingly, differentially abundant taxa *Enterobacteriaceae* and *Acinetobacter* were detected to be associated with several KOs implicated in stress response which included two-component system KOs for aerobic/anaerobic survival as ArcB and NarP and transcriptional regulation of the *mar-sox-rob* regulon (Supplementary Fig. S2, Supplementary Tables S2 and S5). The *mar-sox-rob* regulon has been reported in coordinating survival against various environmental stresses activated by inducers as paraquat, decanoate and intriguingly, salicylate^71^, functional modules for which is differentially abundant in OSC samples (Supplementary Fig. S3, Supplementary Tables S3 and S6). Additionally, *Acinetobacter* was correlated with the stress response serine protease DegS and iron starvation Fe/S biogenesis protein NfuA (Fig. 4). Thus, these biomarkers seem to contribute to important stress response pathways rather than hydrocarbon degrading capabilities. Additionally, over-represented taxa such as *Oxalobacteraceae*, *Cupriavidus*, *Brucellaceae*, and *Ochrobactrum* (Supplementary Fig. S2, Supplementary Tables S2 and S5) were found to differentially contribute to the abundance of several hydrocarbonoclastic genes (K00446, K00448-51, K03381) (Supplementary Fig. S5) in the OSC samples.

In taiga samples, several detected biomarkers such as *Phenylobacterium*, *Caulobacteraceae*, *Sphingomonadaceae*, *Novosphingobium*, *Rhodococcus*, and *Burkholderiaceae* (Table 3, Supplementary Fig. S2, Supplementary Tables S2 and S5) were found to contribute heavily but differently to the abundance of a plethora of hydrocarbonoclastic genes (Supplementary Fig. S5). Additionally, identification of differentially abundant functional modules for the assimilation of sulphate, transformation of thiosulphate to sulphate and regulation of the SOX complex responsible for thiosulphate transformation (M00176, M00595, M00523) underline the preferential sulphur usage in this site (Fig. 3, Supplementary Fig. S3, Supplementary Tables S3 and S6). This is well supported by the identification of *Bradyrhizobium*, *Caulobacter*, and *Burkholderia* as biomarkers (Supplementary Fig. S2, Supplementary Tables S2 and S5), all which are known to be involved in sulfur metabolism^72, 73^ and house homologous genes for the same. The pathogenic *Burkholderia*, a taxonomic biomarker for Tb samples (Supplementary Fig. S2, Supplementary Tables S2 and S5), although known for its hydrocarbon degradation capabilities^32^ was found to make only minor contributions to the abundance of a few hydrocarbonoclastic genes (K03381, K00448-449, K00451) (Supplementary Fig. S5) and therefore may not play a major remedial role in the taiga samples but contribute differently to the habitat. Importantly, a number of biomarkers identified here for the taiga samples such as *Phenylobacterium*, *Sphingomonadaceae*, *Novosphingobium* and *Rhodococcus* were detected as “habitat specialists” in oil contaminated taiga samples recently by Yang *et al*.^74^.

Anaerobic, photoautotrophic *Ignavibacteriaceae*, was identified as a biomarker in I samples (Table 3, Supplementary Fig. S2, Supplementary Tables S2 and S5). Additionally, KEGG modules such as dissimilatory sulfate reduction, sulfate = >H2S (M00596) and NarX-NarL (nitrate respiration) two-component regulatory system (M00471; also found to be present with complete coverage) were found to be differentially abundant in these samples (Fig. 3, Table 4, Supplementary Fig. S3 and S4, Supplementary Tables S3, S4 and S6). This indicated that anaerobic processes and taxa play a major role in the oil contaminated I samples. Simultaneous identification of over-represented aerobic pathways such as formaldehyde assimilation, serine pathway (M00346) (Supplementary Fig. S3, Supplementary Tables S3 and S6) and differentially abundant aerobic taxa such as *Methylibium*, *Chitinophagaceae* and sulfate oxidizing *Thiobacillus* (Table 3, Supplementary Fig. S2, Supplementary Tables S2 and S5) however indicated that these environments may also have aerobic aspects. Microbial association network inferred for I samples showed a large proportion of significant interactions to be antagonistic in nature. Microbial relationships in the I samples showed intense competition among taxa which included both aerobic (*Parvibaculum*, *Chitinophagaceae*, *Acidocella*) and anaerobic bacteria (*Anaerolineales*, *Ignavibacteriaceae*) (Fig. 5, Supplementary Table S8). These observations of an intertwined network of anaerobic and aerobic bacteria along with findings stated above indicate co-existence of these taxa in relative proximity with competition for resources and a possibly microaerophilic or partially anaerobic oil polluted habitat. One of only two significantly positive correlations in I samples was found to be shared between *Methylibium* and *Parvibaculum* (Fig. 5, Sup﻿plementary Table S8). *Methylibium petroleiphilum* was also detected as a biomarker for I samples and contributed significantly to the hydrocarbon degradation capabilities at these sites (Supplementary Fig. S2 and S5, Supplementary Tables S2 and S5). *Methylibium petroleiphilum*, an aerobic bacterium, has previously been reported to degrade hydrocarbons such as methyl tert-butyl ether, a compound frequently used in oil refineries^75^. It can therefore be speculated that these two taxa may be involved in a mutualistic relationship, possibly concerning hydrocarbon degradation, wherein the metabolically better adapted *Methylibium* may provide *Parvibaculum* with a competitive edge and facilitate its enrichment in the I samples (Fig. 5, Supplementary Table S8).

Similar to the results of An *et al*.^12^, we encountered a significantly high proportion of anaerobic taxa in the OSTP samples, among which *Anaerolinaceae*, *Syntrophaceae*, *Desulfobulbaceae*, *Peptococcaceae*, *Geobacteraceae*, *Syntrophorhabdaceae* and the thermophilic *Caldiserica* ^76^ were detected as biomarkers (Table 3, Supplementary Fig. S2, Supplementary Tables S2 and S5). Detected taxonomic biomarkers such as *Anaerolinaceae* and *Comamonadaceae* (Table 3, Supplementary Fig. S2, Supplementary Tables S2 and S5) were found to make significant contributions to the abundance of hydrocarbon degradation genes (Supplementary Fig. S5) in OSTP samples. Other identified biomarkers such as *Geobacteraceae* and *Thauera* (Table 3, Supplementary Fig. S2, Supplementary Tables S2 and S5) are well known anaerobic hydrocarbon degraders^77, 78^. Additionally, another detected biomarker *Nitrospirales* (Supplementary Fig. S2, Supplementary Tables S2 and S5), which is known to be involved in nitrification^79^ may contribute to an over-represented module ammonia = > nitrite transformation (M00528) that was identified in OSTP samples (Supplementary Fig. S3, Supplementary Tables S3 and S6). Biomarkers of sulfate reducing bacteria such as *Desulfuromonadales* and *Desulfobulbaceae* (Table 3, Supplementary Fig. S2, Supplementary Tables S2 and S5), which is a known mesophilic/psychrophilic sulfate reducer^80^, may be involved in critical sulfur metabolism pathways known to be important in OSTPs^81^. Interestingly, obligate anaerobes such as *Anaerolinaceae* have previously been associated with sulfate reducing conditions in the OSTPs^82^. Lastly, major contributions for hydrocarbonoclastic capabilities in OSTP samples was also observed from biomarkers *Pseudomonas* (K00446, K00448, K00449, K00496, K03381) and *Rhodocyclaceae* (K04100-01) (Table 3, Supplementary Fig. S2 and S5, Supplementary Tables S2 and S5) furthering the hydrocarbon degradation capabilities of OSTPs.

We also investigated significant bacterial associations in oil polluted sites to decipher important co-occurrence and co-exclusion relationships. Our results showed that greater co-occurrence exists between phylotypes sharing an evolutionary lineage while more co-exclusions were observed between phylotypes from different ancestries. This observation has also been previously reported in microbial correlation studies in the environment^46, 83^. Interestingly, not a large proportion of taxonomic biomarkers were observed to be represented in these significant correlations. This can possibly happen due to separation of niches due to various environmental and even temporal factors. For example, in the bacterial association network for DWH samples, biomarker *Colwelliaceae* was detected to participate in a significantly positive relationship with *Desulfobulbaceae*, a strictly anaerobic sulfate utilizing bacterial family (Fig. 5﻿, Supplementary Table S8). The existence of this kind of a relationship, based on degradation of recalcitrant hydrocarbons, was inferred upon by the original authors too^54^. Strikingly however, the most abundant and robust hydrocarbon degrader predicted for DWH samples in our study, i.e. *HB2*.*32*.*21* (Supplementary Fig. S5) was not detected to be involved in any significant associations. This observation can be explained by a possible individual capacity of survival for *HB2*.*32*.*21* due to its hydrocarbonoclastic capacities without extensive interactions with other resident bacteria, therefore occupying a separate niche in the oil polluted marine sediment site. Thus, significant correlations (both positive and negative) may be driven by factors other than only oil pollution in oil contaminated sites with apparently benign taxa being involved in such interactions. This indicates that biomarkers and correlation networks must be studied in tandem to deduce meaningful conclusions.

Our results therefore show that, detected biomarkers may contribute differently to strictly hydrocarbonoclastic properties when compared across sites, but their close association with most differentially abundant KOs and as an extension, several over-represented functional pathways for each site underlines their significance in these oil contaminated sites. We find that although many of the taxonomic biomarkers contribute to hydrocarbonoclastic capabilities, some do not and can therefore contribute to other possibly important functions. These observations not only elucidate important taxa contributing functions more specific and essential to each site, but also shows that niches related to functions other than hydrocarbon degradation may significantly influence bacteriome structure in oil polluted sites, possibly more in sites with understandably lower degrees of contamination. This indicates clearly that while hydrocarbonoclastic capabilities may be a driving force for continued survival in these sites, other immediate factors including availability of different organic and inorganic compounds and environmental stress can heavily influence evolution of the bacteriome also. Thus, we see that a combination of oil degradation capabilities and environmental factors shape the landscape for bacterial petroleum degradation. As an extension, it therefore becomes imperative to examine oil bioremediation processes, especially aimed at empirical identification of biomarkers, in totality with due comparison to similar studies and not in isolation as it may lead to misleading conclusions. This is well illustrated in some previous studies that have focused on predicting microbial markers or proxies for oil pollution in certain environments^52, 53^. In the study on mangrove oil pollution and detection of microbial proxies by dos Santos *et al*.^53^, *Marinobacter*, belonging to family *Alteromonadaceae*, was identified as a possible biomarker for oil pollution in mangroves. However, in our study when compared to other sites, *Alteromonadaceae* was detected to be differentially abundant in the DWH samples and *Marinobacter* was not identified as over-represented in any of the oil polluted sites (Table 3, Supplementary Fig. S2, Supplementary Tables S2 and S5).

## Conclusion

Meta-omics approaches such as meta-genomics, transcriptomics or proteomics, integrated with an in-depth analysis of large and exhaustive datasets using state-of-the-art bioinformatic tools bound in efficient cohesion offer enhanced and possibly novel interpretations of microbial and trophic associations occurring in the environment. In this study, we have implemented an atypical, evolving computational pipeline, that employs contemporary bioinformatic contrivances to explore and decipher characteristics of bacterial response to oil contamination in diverse environments from 16S rRNA sequence datasets. Our study showed that significant taxonomic and functional differences exist between geographically and/or spatially isolated oil polluted sites and that oil pollution is not the sole driving factor in determination of the metagenomic fabric at these sites, even if maybe the most predominant one. We have successfully demonstrated that several important taxonomic clades and functional modules detected for these habitats are often involved in extra-hydrocarbonoclastic activities, thus underlining the importance of these apparently peripheral niches related to endemic environmental responses in the survival of oil contaminated ecosystems. In the process, we inferred robust taxonomic and functional biomarkers along with competitive and cooperative interactions among bacteria at diverse oil contaminated sites, that are representative of an entire oil polluted habitat and not only its hydrocarbonoclastic capabilities. To our knowledge, this is the first population genomics study carried out on petroleum hydrocarbon polluted habitats. The present study contributes novel insights into the complex ecological dynamics of oil polluted bacteriomes besides providing relevant analytical and visualization methods for studying the relation between soil biodiversity and ecosystem function from environmental 16S rRNA phylogenetic survey data.

## Electronic supplementary material

Supplementary Data
