# Supplementary material for: Bioinformatic Approaches Including Predictive Metagenomic Profiling Reveal Characteristics of Bacterial Response to Petroleum Hydrocarbon Contamination in Diverse Environments
Source: Sci Rep. 2017 Apr 24;7:1108. doi: 10.1038/s41598-017-01126-3 (PMC5430712; doi:10.1038/s41598-017-01126-3)
Supplement: Supplementary file 1 — Supplementary Data [file 41598_2017_1126_MOESM1_ESM.pdf]

Bioinformatic Approaches Including Predictive Metagenomic Profiling Reveal Characteristics of Bacterial Response to Petroleum Hydrocarbon Contamination in Diverse Environments

Arghya Mukherjee, Bobby Chettri, James S. Langpoklakpam, Pijush Basak, Aravind Prasad, Ashis K. Mukherjee, Maitree Bhattacharyya, Arvind K. Singh and Dhrubajyoti Chattopadhyay

---

Supplementary Data

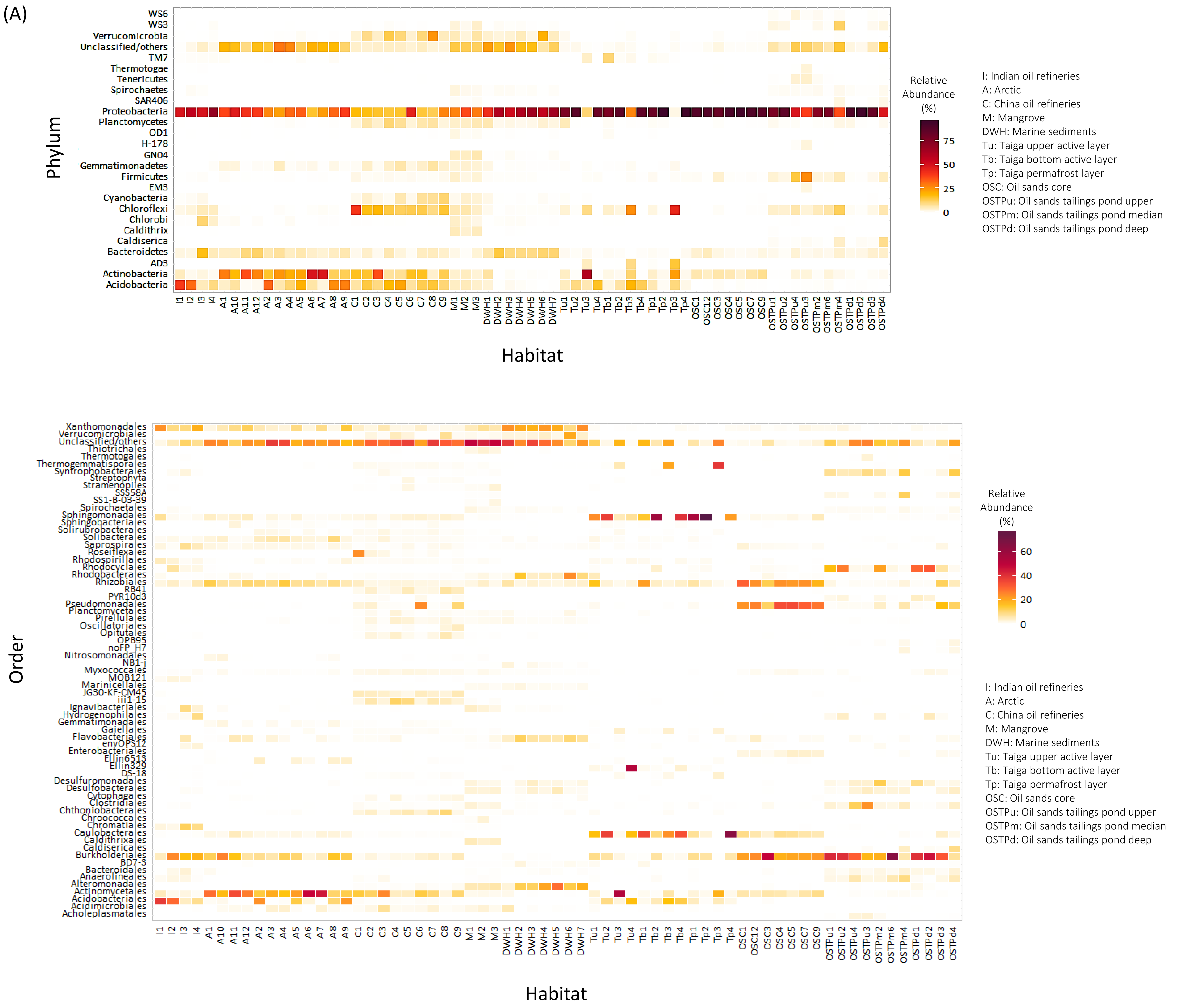

Supplementary data. Figure S1. Taxonomic distribution of bacterial communities in oil contaminated environments. Taxonomic clades detected at relative abundance ≥ 2% in at least one of 65 oil contaminated samples are represented. (A) Distribution at the phylum level, (B) Distribution at the order level.

Supplementary data. Figure S2. Taxonomic biomarkers of bacterial communities from oil polluted habitats. Cladogram showing all taxonomic clades detected at a relative abundance ≥ 0.5% in at least five samples across all habitats. Abundances for these taxa were used as inputs for LEfSe which detected taxonomic biomarkers for each taxonomic level. Seven rings of the cladogram represent phylum (innermost), class, order, family, genus and species (outermost), respectively and are rooted using kingdoms Bacteria and Archaea. Annotations for taxonomic clades follow the reverse pattern, wherein phyla are annotated in the outermost section of the cladogram and lower taxonomic levels annotated further inside ending at the outermost circle in that section of the cladogram. Circles representing differentially abundant taxa detected as taxonomic biomarkers are colored according to the individual soil habitat wherein they are over-represented among 12 oil polluted ecosystems (see legend). Circles not over-represented in any habitat are colorless. Brackets represent a single taxon at that taxonomic level from which daughter taxa originate. Taxa with a single daughter taxon are joined using regular branches. More information on this style of representation can be found elsewhere [28,36,83].

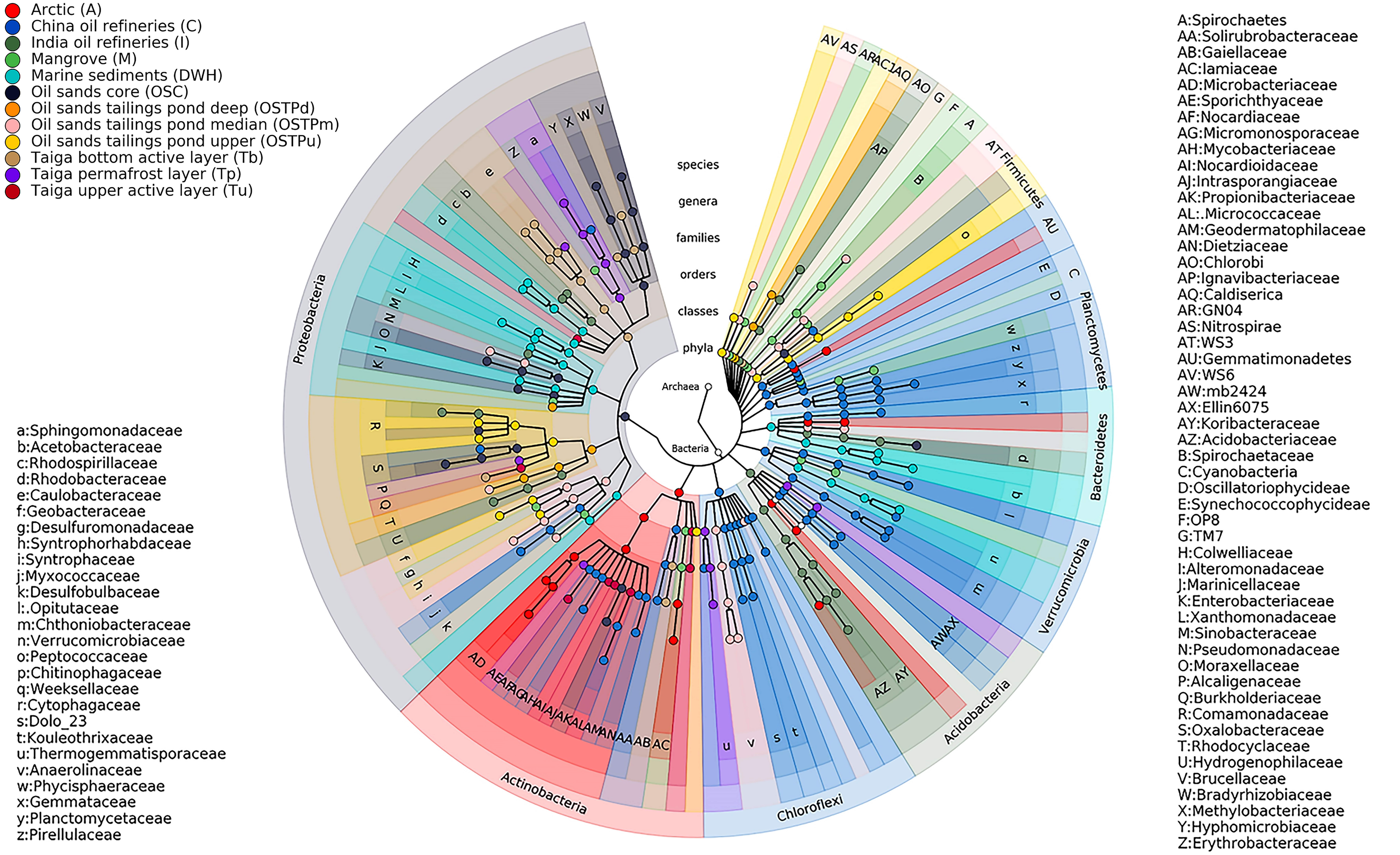

Arctic (A)
China oil refineries (C)
India oil refineries (I)
Mangrove (M)
Marine sediments (DWH)
Oil sands core (OSC)
Oil sands tailings pond deep (OSTPd)
Oil sands tailings pond median (OSTPm)
Oil sands tailings pond upper (OSTPu)
Taiga bottom active layer (Tb)
Taiga permafrost layer (Tp)
Taiga upper active layer (Tu)
species
genera
families
orders
classes
phyla
Archaea
Bacteria
Proteobacteria
Actinobacteria
Chloroflexi
Acidobacteria
Verrucomicrobia
Bacteroidetes
Planctomycetes
Firmicutes
a:Sphingomonadaceae
b:Acetobacteraceae
c:Rhodospirillaceae
d:Rhodobacteraceae
e:Caulobacteraceae
f:Geobacteraceae
g:Desulfuromonadaceae
h:Syntrophorhabdaceae
i:Syntrophaceae
j:Myxococcaceae
k:Desulfobulbaceae
l:.Opitutaceae
m:Chthoniobacteraceae
n:Verrucomicrobiaceae
o:Peptococcaceae
p:Chitinophagaceae
q:Weeksellaceae
r:Cytophagaceae
s:Dolo_23
t:Kouleothrixaceae
u:Thermogemmatisporaceae
v:Anaerolinaceae
w:Phycisphaeraceae
x:Gemmataceae
y:Planctomycetaceae
z:Pirellulaceae
A:Spirochaetes
AA:Solirubrobacteraceae
AB:Gaiellaceae
AC:Iamiaceae
AD:Microbacteriaceae
AE:Sporichthyaceae
AF:Nocardiaceae
AG:Micromonosporaceae
AH:Mycobacteriaceae
AI:Nocardioidaceae
AJ:Intrasporangiaceae
AK:Propionibacteriaceae
AL:.Micrococcaceae
AM:Geodermatophilaceae
AN:Dietziaceae
AO:Chlorobi
AP:Ignavibacteriaceae
AQ:Caldiserica
AR:GN04
AS:Nitrospirae
AT:WS3
AU:Gemmatimonadetes
AV:WS6
AW:mb2424
AX:Ellin6075
AY:Koribacteraceae
AZ:Acidobacteriaceae
B:Spirochaetaceae
C:Cyanobacteria
D:Oscillatoriophycideae
E:Synechococcophycideae
F:OP8
G:TM7
H:Colwelliaceae
I:Alteromonadaceae
J:Marinicellaceae
K:Enterobacteriaceae
L:Xanthomonadaceae
M:Sinobacteraceae
N:Pseudomonadaceae
O:Moraxellaceae
P:Alcaligenaceae
Q:Burkholderiaceae
R:Comamonadaceae
S:Oxalobacteraceae
T:Rhodocyclaceae
U:Hydrogenophilaceae
V:Brucellaceae
W:Bradyrhizobiaceae
X:Methylobacteriaceae
Y:Hyphomicrobiaceae
Z:Erythrobacteraceae

Supplementary data. Figure S3. High-resolution cladogram of metabolic reconstruction of metagenomes and functional biomarkers from oil polluted habitats. Cladogram showing the 4-level KEGG BRITE hierarchical structure denoted by four rings, as inferred against KEGG metabolic modules detected by HUMAnN2 from metagenomic gene family abundance data produced by PICRUSt for all oil contaminated samples. The outermost ring represents KEGG functional modules that have been detected in at least one of the 65 PICRUSt predicted metagenomes as reconstructed by HUMAnN2, while the innermost ring represents the Level 1 KEGG BRITE clades. Over-represented metabolic modules inferred from KEGG module abundance data generated by HUMAnN2 by LEfSe are colored corresponding to the oil contaminated habitat they have been identified to be differentially abundant in (see legend). Circles not over-represented in any habitat are colorless. Brackets represent a single KEGG BRITE clade at that Level from which daughter clades originate. KEGG BRITE clades with a single daughter clade are joined through regular branches. Annotations for the KEGG BRITE hierarchy follow an outside-in pattern, wherein Level 1 KEGG BRITE clades are annotated in the outermost section of the cladogram with lower clades annotated further inside ending at the outermost circle in that section of the cladogram. Core and Varied modules along with other differentially abundant KEGG BRITE clades are labelled. More information on this style of representation can be found elsewhere[28,36,83].

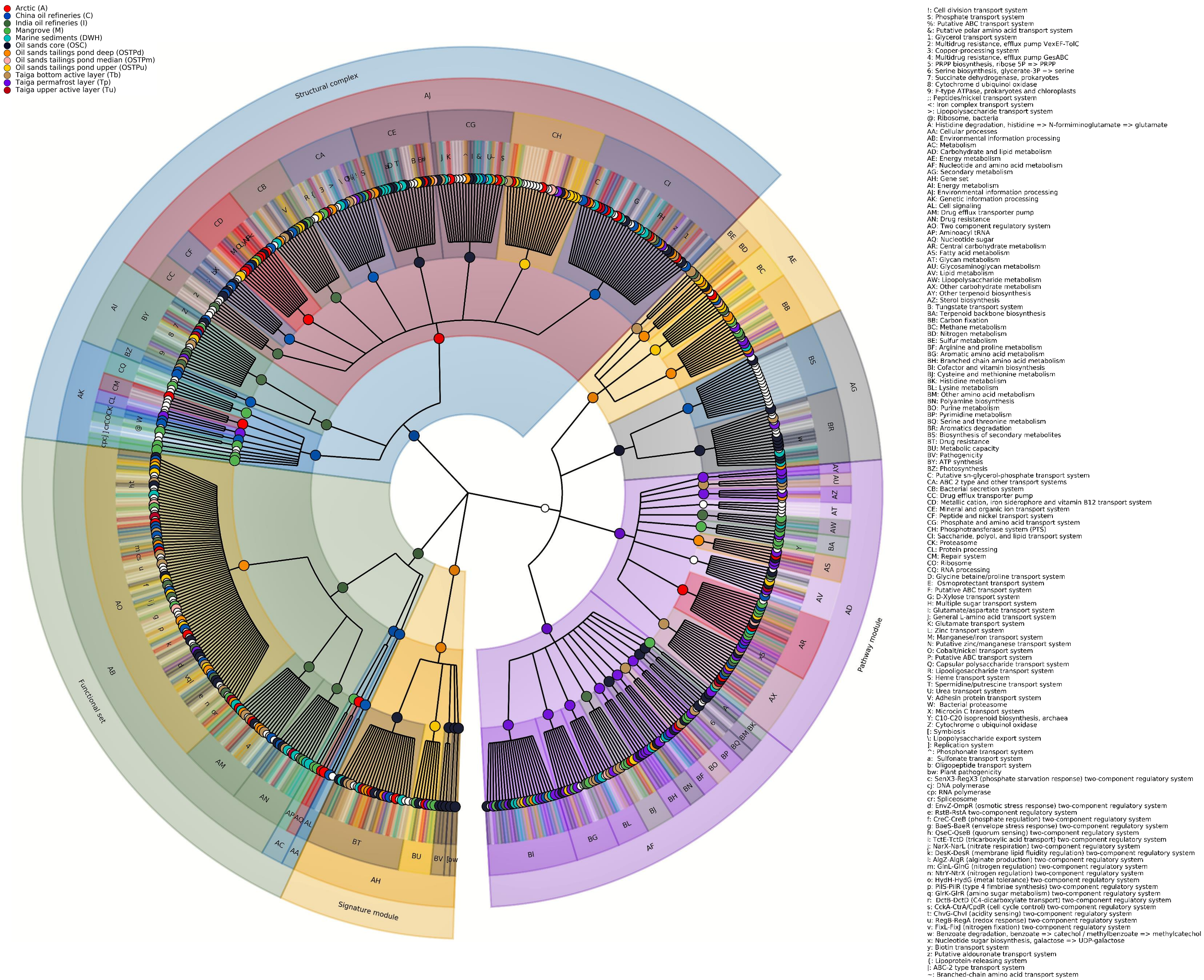
Arctic (A)
China oil refineries (C)
India oil refineries (I)
Mangrove (M)
Marine sediments (DWH)
Oil sands core (OSC)
Oil sands tailings pond deep (OSTPd)
Oil sands tailings pond median (OSTPm)
Oil sands tailings pond upper (OSTPu)
Taiga bottom active layer (Tb)
Taiga permafrost layer (Tp)
Taiga upper active layer (Tu)
Structural complex
Functional set
Signature module
Pathway module
!: Cell division transport system
$: Phosphate transport system
%: Putative ABC transport system
&: Putative polar amino acid transport system
1: Glycerol transport system
2: Multidrug resistance, efflux pump VexEF-TolC
3: Copper-processing system
4: Multidrug resistance, efflux pump GesABC
5: PRPP biosynthesis, ribose 5P => PRPP
6: Serine biosynthesis, glycerate-3P => serine
7: Succinate dehydrogenase, prokaryotes
8: Cytochrome d ubiquinol oxidase
9: F-type ATPase, prokaryotes and chloroplasts
;: Peptides/nickel transport system
<: Iron complex transport system
>: Lipopolysaccharide transport system
@: Ribosome, bacteria
A: Histidine degradation, histidine => N-formiminoglutamate => glutamate
AA: Cellular processes
AB: Environmental information processing
AC: Metabolism
AD: Carbohydrate and lipid metabolism
AE: Energy metabolism
AF: Nucleotide and amino acid metabolism
AG: Secondary metabolism
AH: Gene set
AI: Energy metabolism
AJ: Environmental information processing
AK: Genetic information processing
AL: Cell signaling
AM: Drug efflux transporter pump
AN: Drug resistance
AO: Two component regulatory system
AP: Aminoacyl tRNA
AQ: Nucleotide sugar
AR: Central carbohydrate metabolism
AS: Fatty acid metabolism
AT: Glycan metabolism
AU: Glycosaminoglycan metabolism
AV: Lipid metabolism
AW: Lipopolysaccharide metabolism
AX: Other carbohydrate metabolism
AY: Other terpenoid biosynthesis
AZ: Sterol biosynthesis
B: Tungstate transport system
BA: Terpenoid backbone biosynthesis
BB: Carbon fixation
BC: Methane metabolism
BD: Nitrogen metabolism
BE: Sulfur metabolism
BF: Arginine and proline metabolism
BG: Aromatic amino acid metabolism
BH: Branched chain amino acid metabolism
BI: Cofactor and vitamin biosynthesis
BJ: Cysteine and methionine metabolism
BK: Histidine metabolism
BL: Lysine metabolism
BM: Other amino acid metabolism
BN: Polyamine biosynthesis
BO: Purine metabolism
BP: Pyrimidine metabolism
BQ: Serine and threonine metabolism
BR: Aromatics degradation
BS: Biosynthesis of secondary metabolites
BT: Drug resistance
BU: Metabolic capacity
BV: Pathogenicity
BY: ATP synthesis
BZ: Photosynthesis
C: Putative sn-glycerol-phosphate transport system
CA: ABC 2 type and other transport systems
CB: Bacterial secretion system
CC: Drug efflux transporter pump
CD: Metallic cation, iron siderophore and vitamin B12 transport system
CE: Mineral and organic ion transport system
CF: Peptide and nickel transport system
CG: Phosphate and amino acid transport system
CH: Phosphotransferase system (PTS)
CI: Saccharide, polyol, and lipid transport system
CK: Proteasome
CL: Protein processing
CM: Repair system
CO: Ribosome
CQ: RNA processing
D: Glycine betaine/proline transport system
E: Osmoprotectant transport system
F: Putative ABC transport system
G: D-Xylose transport system
H: Multiple sugar transport system
I: Glutamate/aspartate transport system
J: General L-amino acid transport system
K: Glutamate transport system
L: Zinc transport system
M: Manganese/iron transport system
N: Putative zinc/manganese transport system
O: Cobalt/nickel transport system
P: Putative ABC transport system
Q: Capsular polysaccharide transport system
R: Lipooligosaccharide transport system
S: Heme transport system
T: Spermidine/putrescine transport system
U: Urea transport system
V: Adhesin protein transport system
W: Bacterial proteasome
X: Microcin C transport system
Y: C10-C20 isoprenoid biosynthesis, archaea
Z: Cytochrome o ubiquinol oxidase
[: Symbiosis
\: Lipopolysaccharide export system
]: Replication system
^: Phosphonate transport system
a: Sulfonate transport system
b: Oligopeptide transport system
bw: Plant pathogenicity
c: SenX3-RegX3 (phosphate starvation response) two-component regulatory system
cj: DNA polymerase
cp: RNA polymerase
cr: Spliceosome
d: EnvZ-OmpR (osmotic stress response) two-component regulatory system
e: RstB-RstA two-component regulatory system
f: CreC-CreB (phosphate regulation) two-component regulatory system
g: BaeS-BaeR (envelope stress response) two-component regulatory system
h: QseC-QseB (quorum sensing) two-component regulatory system
i: TctE-TctD (tricarboxylic acid transport) two-component regulatory system
j: NarX-NarL (nitrate respiration) two-component regulatory system
k: DesK-DesR (membrane lipid fluidity regulation) two-component regulatory system
l: AlgZ-AlgR (alginate production) two-component regulatory system
m: GlnL-GlnG (nitrogen regulation) two-component regulatory system
n: NtrY-NtrX (nitrogen regulation) two-component regulatory system
o: HydH-HydG (metal tolerance) two-component regulatory system
p: PilS-PilR (type 4 fimbriae synthesis) two-component regulatory system
q: GlrK-GlrR (amino sugar metabolism) two-component regulatory system
r: DctB-DctD (C4-dicarboxylate transport) two-component regulatory system
s: CckA-CtrA/CpdR (cell cycle control) two-component regulatory system
t: ChvG-ChvI (acidity sensing) two-component regulatory system
u: RegB-RegA (redox response) two-component regulatory system
v: FixL-FixJ (nitrogen fixation) two-component regulatory system
w: Benzoate degradation, benzoate => catechol / methylbenzoate => methylcatechol
x: Nucleotide sugar biosynthesis, galactose => UDP-galactose
y: Biotin transport system
z: Putative aldouronate transport system
{: Lipoprotein-releasing system
|: ABC-2 type transport system
~: Branched-chain amino acid transport system

Supplementary data. Figure S4. Core and varied modules of metagenomes from oil contaminated habitats. Heat-map showing a subset of KEGG modules detected as core modules (significant presence i.e. coverage of > 90% across all habitats) and varied modules (significant presence i.e. coverage of > 90% in some habitats and significant absence i.e. coverage of < 10% in others). PICRUSt predicted metagenomes were supplied to HUMAnN2, which calculated coverage for each detected KEGG module using MinPath. Most KEGG modules showed a coverage of 0.1-0.9 and were labelled as non-significant presence/absence and are not shown here. Darker regions of the heat-map represent greater coverage while lighter regions represent lower coverage of the corresponding KEGG module. Habitats are denoted by corresponding colours (see legend) at the top of the heatmap and labels at the bottom.

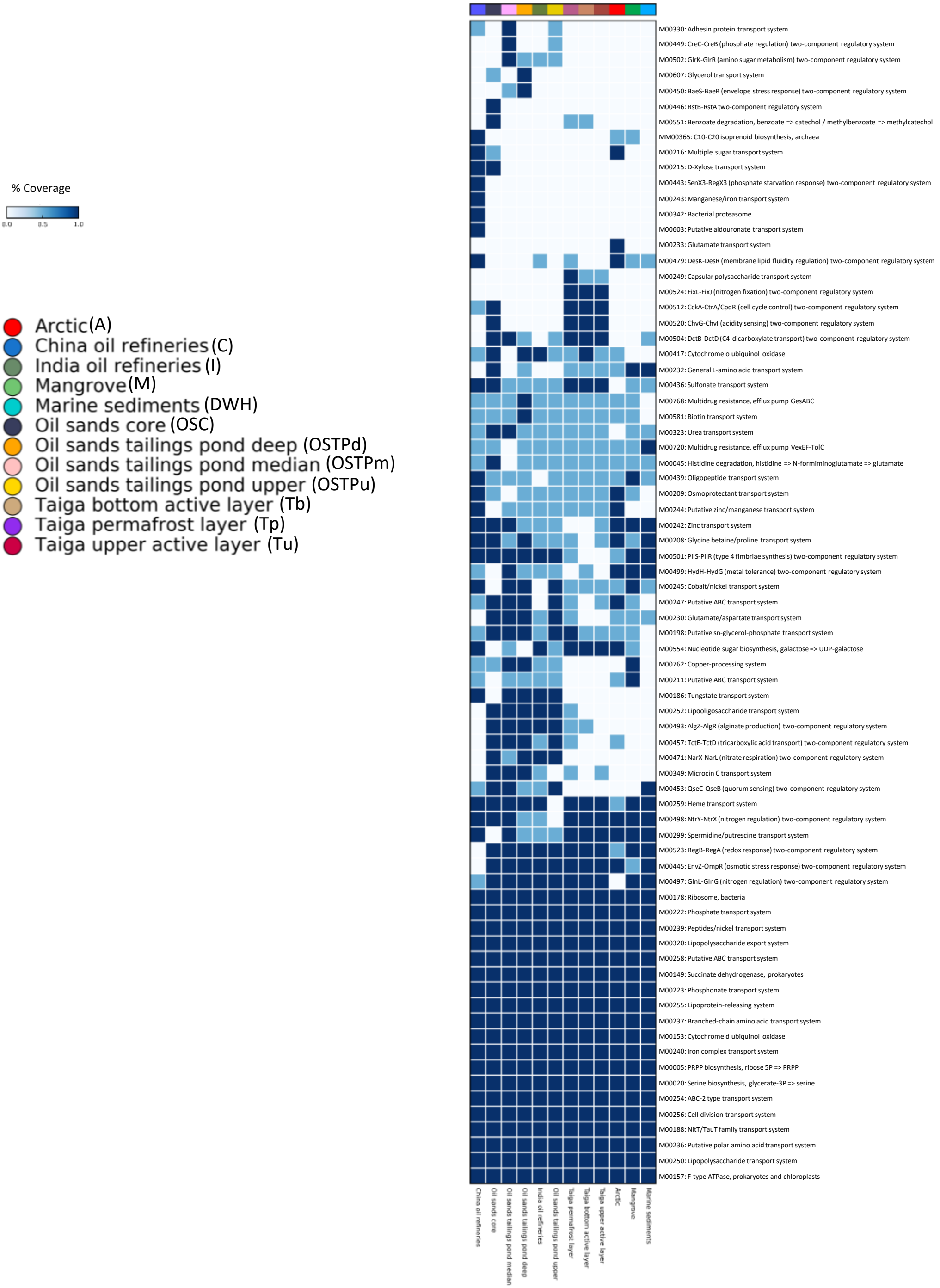
% Coverage
0.0
0.5
1.0
Arctic (A)
China oil refineries (C)
India oil refineries (I)
Mangrove (M)
Marine sediments (DWH)
Oil sands core (OSC)
Oil sands tailings pond deep (OSTPd)
Oil sands tailings pond median (OSTPm)
Oil sands tailings pond upper (OSTPu)
Taiga bottom active layer (Tb)
Taiga permafrost layer (Tp)
Taiga upper active layer (Tu)
M00330: Adhesin protein transport system
M00449: CreC-CreB (phosphate regulation) two-component regulatory system
M00502: GlrK-GlrR (amino sugar metabolism) two-component regulatory system
M00607: Glycerol transport system
M00450: BaeS-BaeR (envelope stress response) two-component regulatory system
M00446: RstB-RstA two-component regulatory system
M00551: Benzoate degradation, benzoate => catechol / methylbenzoate => methylcatechol
MM00365: C10-C20 isoprenoid biosynthesis, archaea
M00216: Multiple sugar transport system
M00215: D-Xylose transport system
M00443: SenX3-RegX3 (phosphate starvation response) two-component regulatory system
M00243: Manganese/iron transport system
M00342: Bacterial proteasome
M00603: Putative aldouronate transport system
M00233: Glutamate transport system
M00479: DesK-DesR (membrane lipid fluidity regulation) two-component regulatory system
M00249: Capsular polysaccharide transport system
M00524: FixL-FixJ (nitrogen fixation) two-component regulatory system
M00512: CckA-CtrA/CpdR (cell cycle control) two-component regulatory system
M00520: ChvG-ChvI (acidity sensing) two-component regulatory system
M00504: DctB-DctD (C4-dicarboxylate transport) two-component regulatory system
M00417: Cytochrome o ubiquinol oxidase
M00232: General L-amino acid transport system
M00436: Sulfonate transport system
M00768: Multidrug resistance, efflux pump GesABC
M00581: Biotin transport system
M00323: Urea transport system
M00720: Multidrug resistance, efflux pump VexEF-TolC
M00045: Histidine degradation, histidine => N-formiminoglutamate => glutamate
M00439: Oligopeptide transport system
M00209: Osmoprotectant transport system
M00244: Putative zinc/manganese transport system
M00242: Zinc transport system
M00208: Glycine betaine/proline transport system
M00501: PilS-PilR (type 4 fimbriae synthesis) two-component regulatory system
M00499: HydH-HydG (metal tolerance) two-component regulatory system
M00245: Cobalt/nickel transport system
M00247: Putative ABC transport system
M00230: Glutamate/aspartate transport system
M00198: Putative sn-glycerol-phosphate transport system
M00554: Nucleotide sugar biosynthesis, galactose => UDP-galactose
M00762: Copper-processing system
M00211: Putative ABC transport system
M00186: Tungstate transport system
M00252: Lipooligosaccharide transport system
M00493: AlgZ-AlgR (alginate production) two-component regulatory system
M00457: TctE-TctD (tricarboxylic acid transport) two-component regulatory system
M00471: NarX-NarL (nitrate respiration) two-component regulatory system
M00349: Microcin C transport system
M00453: QseC-QseB (quorum sensing) two-component regulatory system
M00259: Heme transport system
M00498: NtrY-NtrX (nitrogen regulation) two-component regulatory system
M00299: Spermidine/putrescine transport system
M00523: RegB-RegA (redox response) two-component regulatory system
M00445: EnvZ-OmpR (osmotic stress response) two-component regulatory system
M00497: GlnL-GlnG (nitrogen regulation) two-component regulatory system
M00178: Ribosome, bacteria
M00222: Phosphate transport system
M00239: Peptides/nickel transport system
M00320: Lipopolysaccharide export system
M00258: Putative ABC transport system
M00149: Succinate dehydrogenase, prokaryotes
M00223: Phosphonate transport system
M00255: Lipoprotein-releasing system
M00237: Branched-chain amino acid transport system
M00153: Cytochrome d ubiquinol oxidase
M00240: Iron complex transport system
M00005: PRPP biosynthesis, ribose 5P => PRPP
M00020: Serine biosynthesis, glycerate-3P => serine
M00254: ABC-2 type transport system
M00256: Cell division transport system
M00188: NitT/TauT family transport system
M00236: Putative polar amino acid transport system
M00250: Lipopolysaccharide transport system
M00157: F-type ATPase, prokaryotes and chloroplasts
China oil refineries
Oil sands core
Oil sands tailings pond median
Oil sands tailings pond deep
India oil refineries
Oil sands tailings pond upper
Taiga permafrost layer
Taiga bottom active layer
Taiga upper active layer
Arctic
Mangrove
Marine sediments

Supplementary data. Figure S5. Metagenome contributions of OTUs at family and genus level for select hydrocarbonoclastic genes as estimated by PICRUSt. Metagenome predicted by PICRUSt was used as input to *plot_metagenome_contributions.R* script. OTUs contributing ≥ 2% to the abundance of a particular hydrocarbonoclastic gene in a particular sample were retained for graphing. Graphs were generated in R with ggplot2. Taxa mentioned in text are marked with ‡.

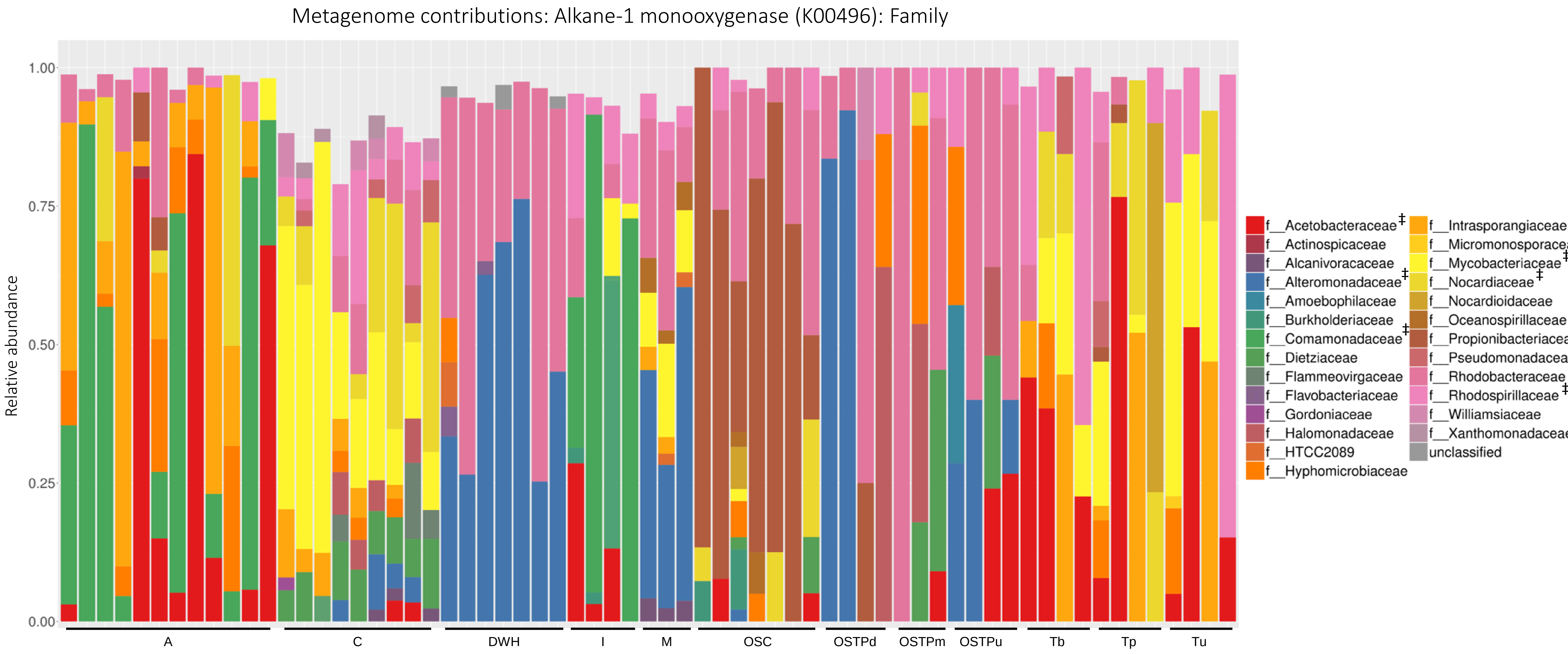

Metagenome contributions: Alkane-1 monooxygenase (K00496): Family
Relative abundance
0.00
0.25
0.50
0.75
1.00
A
C
DWH
I
M
OSC
OSTPd
OSTPm
OSTPu
Tb
Tp
Tu
f__Acetobacteraceae ‡
f__Actinospicaceae
f__Alcanivoracaceae
f__Alteromonadaceae ‡
f__Amoebophilaceae
f__Burkholderiaceae
f__Comamonadaceae ‡
f__Dietziaceae
f__Flammeovirgaceae
f__Flavobacteriaceae
f__Gordoniaceae
f__Halomonadaceae
f__HTCC2089
f__Hyphomicrobiaceae
f__Intrasporangiaceae
f__Mycobacteriaceae
f__Nocardiaceae ‡
f__Nocardioidaceae
f__Williamsiaceae
unclassified

Metagenome contributions: Alkane-1 monooxygenase (K00496): Genus

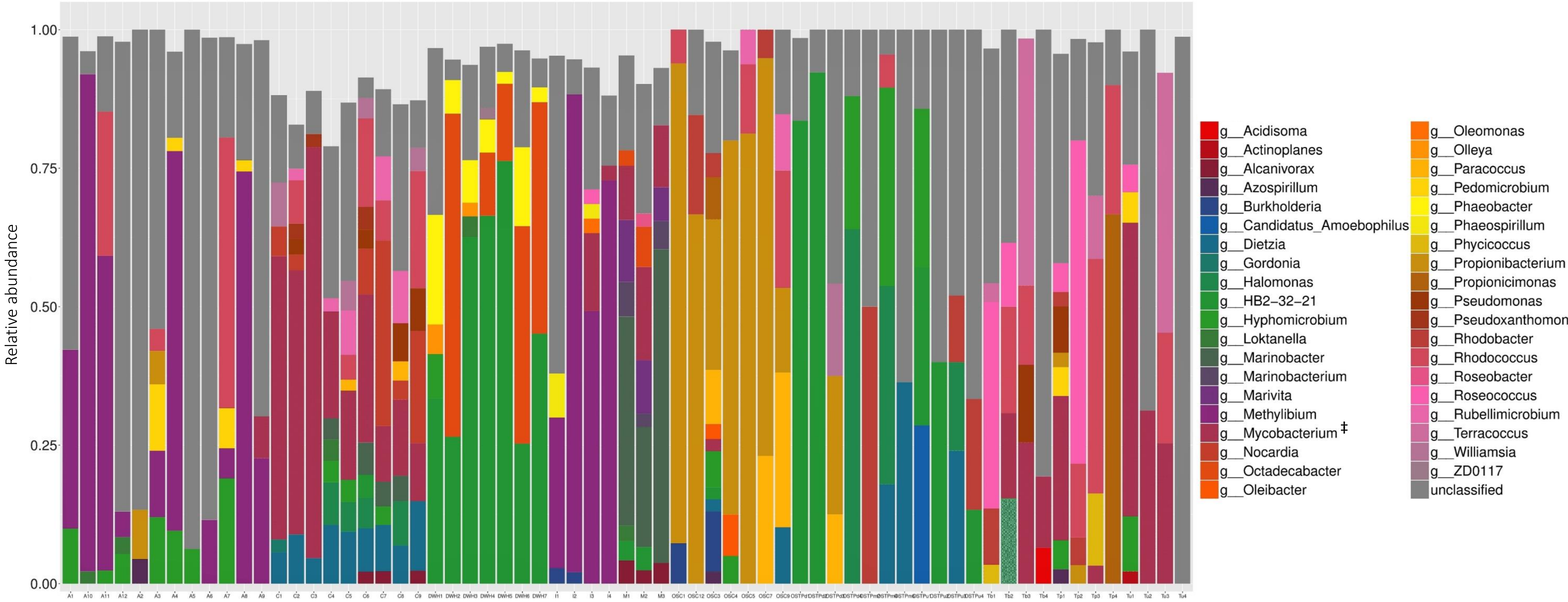

Metagenome contributions: Protocatechuate-4,5 dioxygenase α chain (K04100): Family

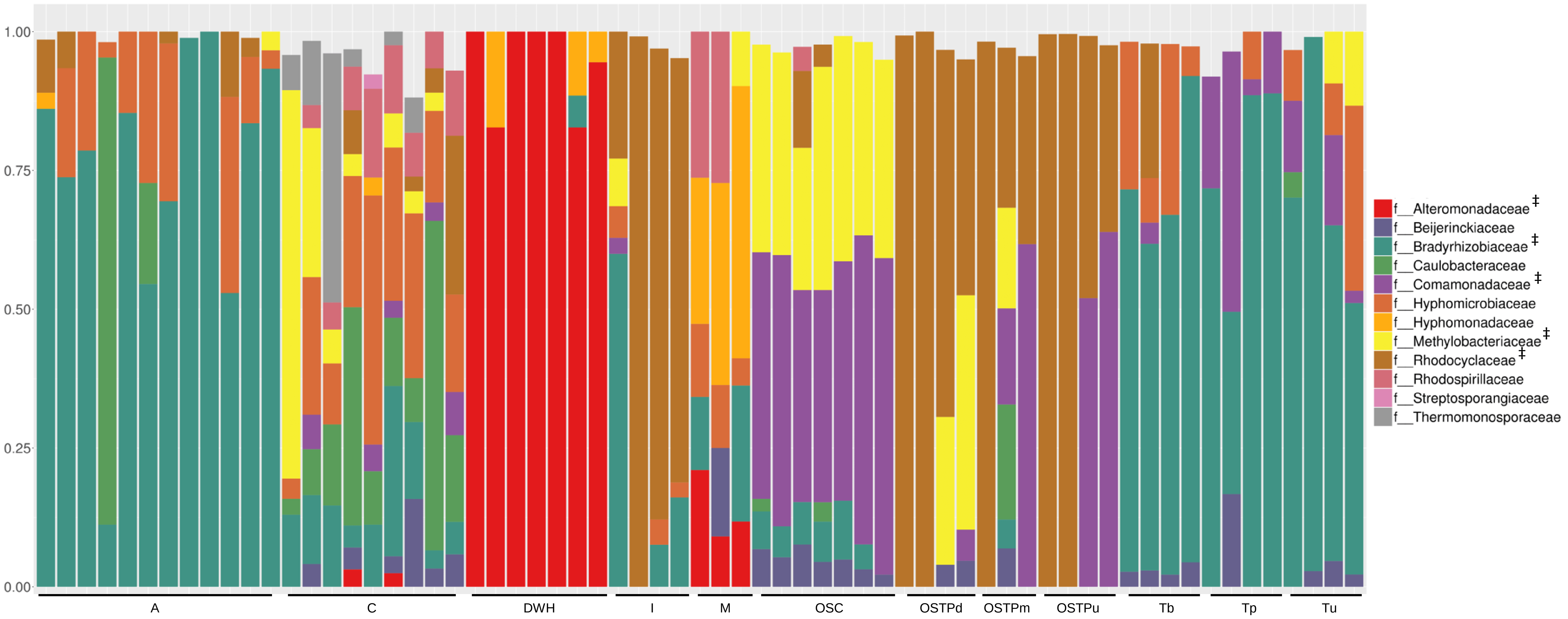

Metagenome contributions: Protocatechuate-4,5 dioxygenase α chain (K04100): Genus

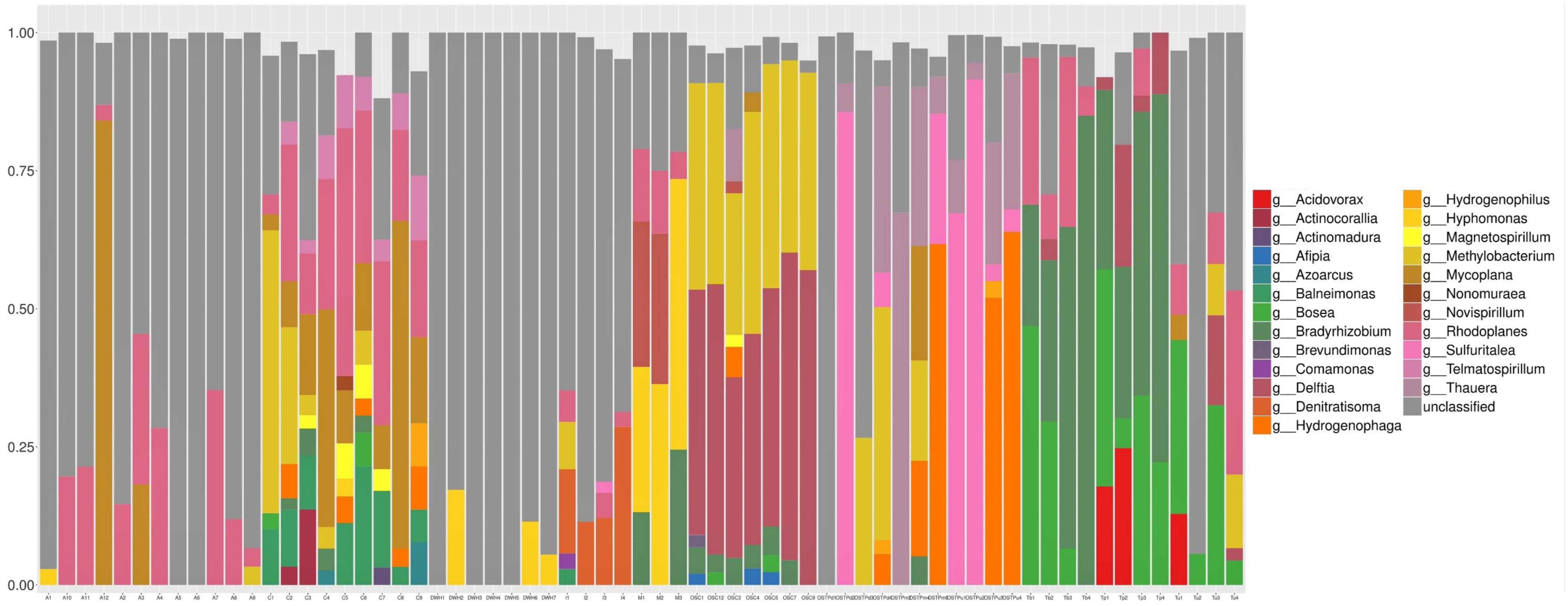

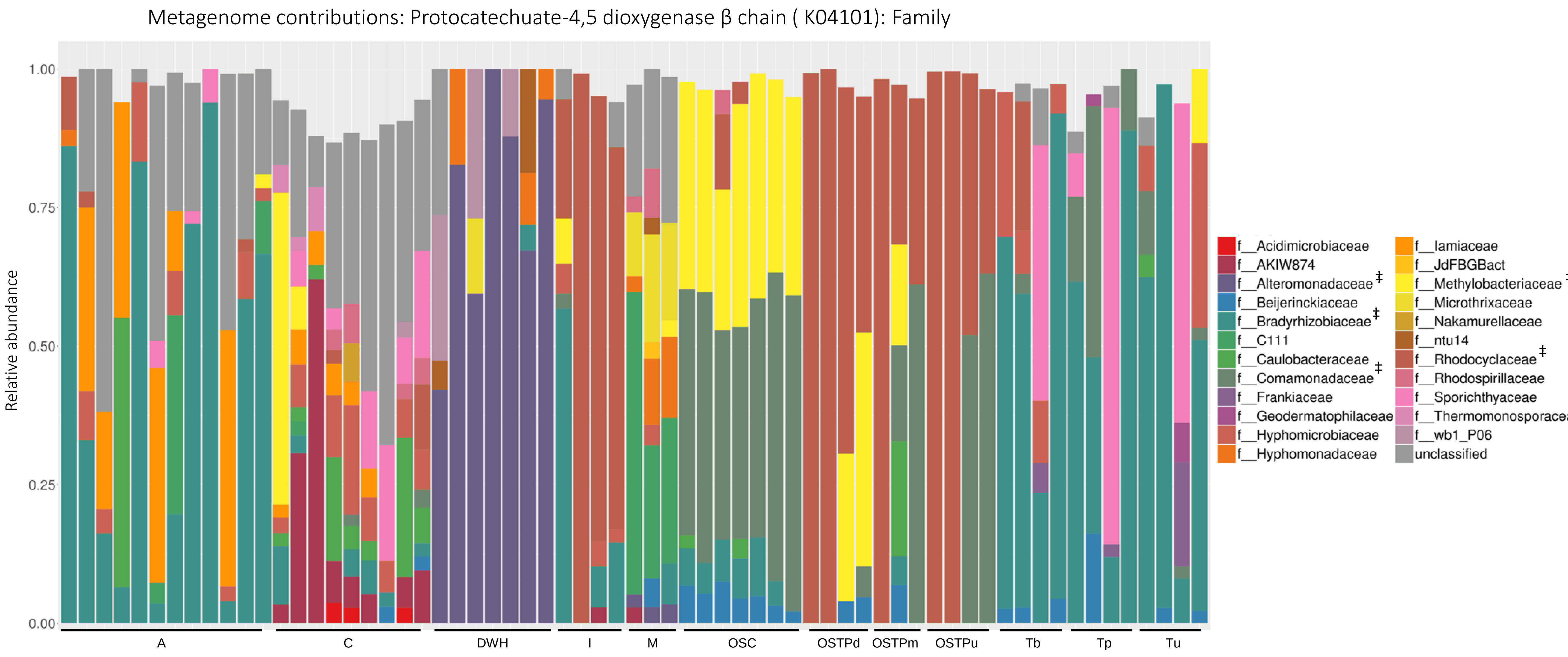

Metagenome contributions: Protocatechuate-4,5 dioxygenase β chain (K04101): Family
Relative abundance
1.00
0.75
0.50
0.25
0.00
A
C
DWH
I
M
OSC
OSTPd
OSTPm
OSTPu
Tb
Tp
Tu
f__Acidimicrobiaceae
f__AKIW874
f__Alteromonadaceae ‡
f__Beijerinckiaceae
f__Bradyrhizobiaceae ‡
f__C111
f__Caulobacteraceae ‡
f__Comamonadaceae
f__Frankiaceae
f__Geodermatophilaceae
f__Hyphomicrobiaceae
f__Hyphomonadaceae
f__Iamiaceae
f__JdFBGBact
f__Methylobacteriaceae
f__Microthrixaceae
f__Nakamurellaceae
f__ntu14
f__Rhodocyclaceae ‡
f__Rhodospirillaceae
f__Sporichthyaceae
f__wb1_P06
unclassified

Metagenome contributions: Protocatechuate-4,5 dioxygenase β chain (K04101): Genus

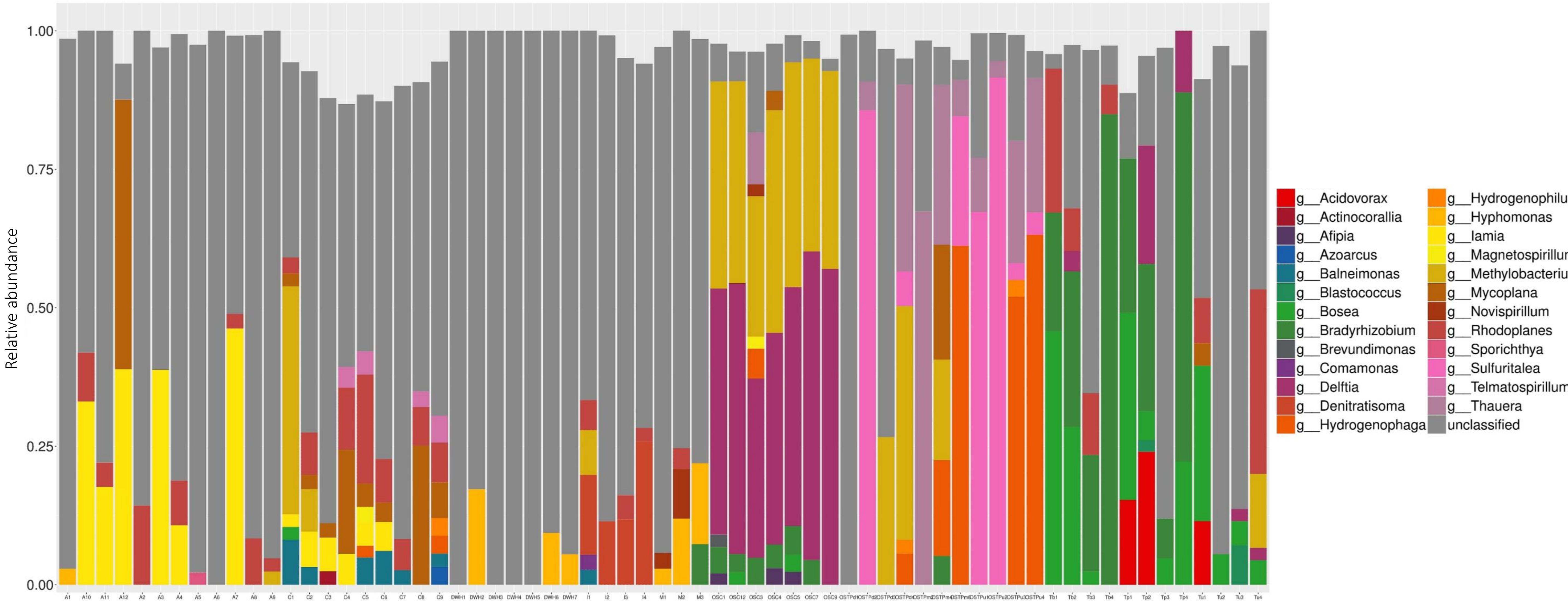

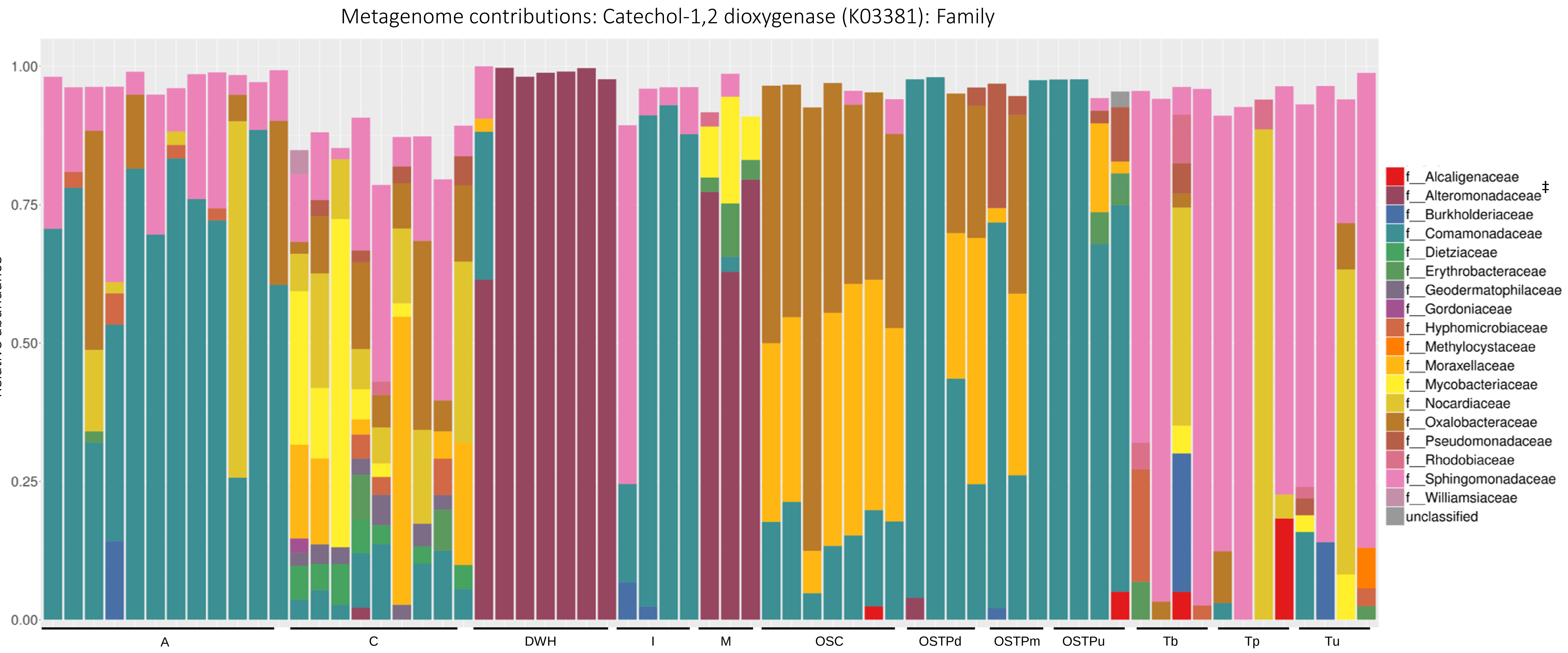
Metagenome contributions: Catechol-1,2 dioxygenase (K03381): Family
1.00
0.75
0.50
0.25
0.00
A
C
DWH
I
M
OSC
OSTPd
OSTPm
OSTPu
Tb
Tp
Tu
f__Alcaligenaceae
f__Alteromonadaceae
f__Burkholderiaceae
f__Comamonadaceae
f__Dietziaceae
f__Erythrobacteraceae
f__Geodermatophilaceae
f__Gordoniaceae
f__Hyphomicrobiaceae
f__Methylocystaceae
f__Moraxellaceae
f__Mycobacteriaceae
f__Nocardiaceae
f__Oxalobacteraceae
f__Pseudomonadaceae
f__Rhodobiaceae
f__Sphingomonadaceae
f__Williamsiaceae
unclassified

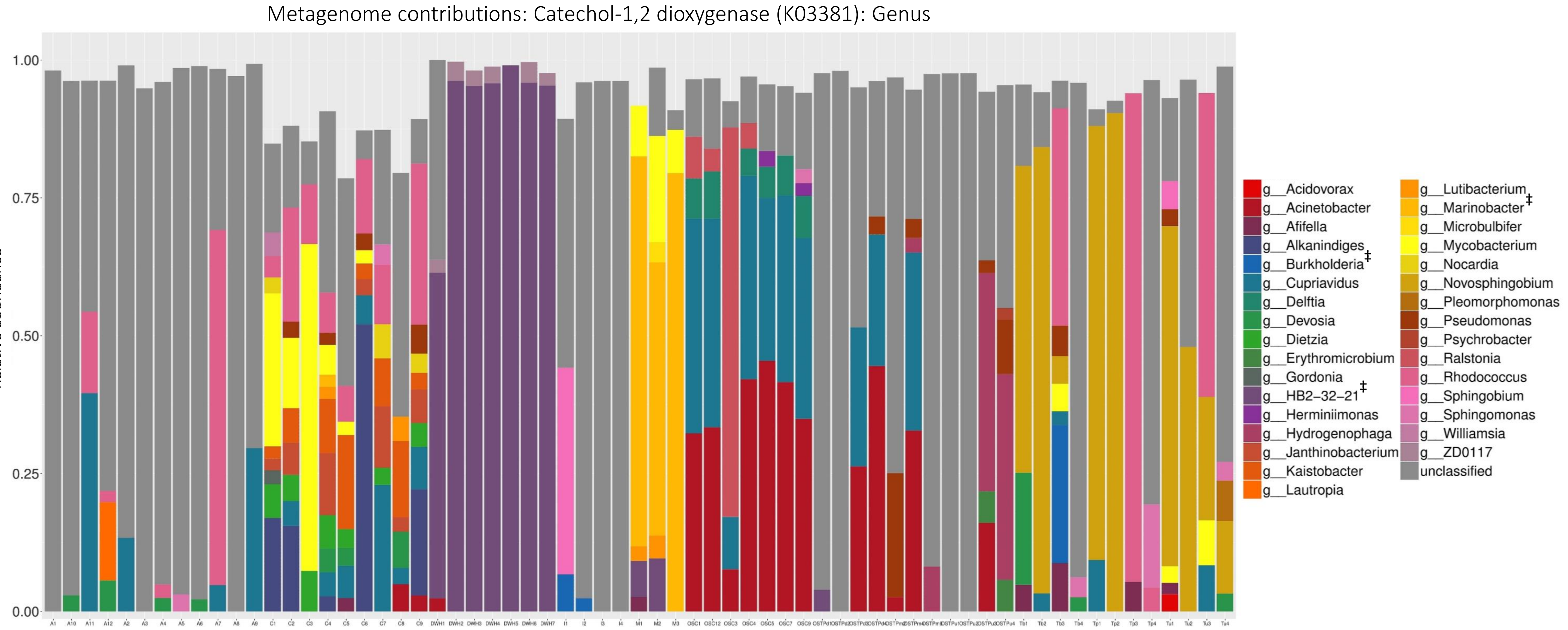

Metagenome contributions: Catechol-1,2 dioxygenase (K03381): Genus
1.00
0.75
0.50
0.25
0.00
A1
A10
A11
A12
A2
A3
A4
A5
A6
A7
A8
A9
C1
C2
C3
C4
C5
C6
C7
C8
C9
DWH1
DWH2
DWH3
DWH4
DWH5
DWH6
DWH7
I1
I2
I3
I4
M1
M2
M3
OSC1
OSC12
OSC3
OSC4
OSC5
OSC7
OSC9
Tb1
Tb2
Tb3
Tb4
Tp1
Tp2
Tp3
Tp4
Tu1
Tu2
Tu3
Tu4
g__Acidovorax
g__Acinetobacter
g__Afifella
g__Alkanindiges‡
g__Burkholderia
g__Cupriavidus
g__Delftia
g__Devosia
g__Dietzia
g__Erythromicrobium
g__Gordonia
g__HB2–32–21‡
g__Herminiimonas
g__Hydrogenophaga
g__Janthinobacterium
g__Kaistobacter
g__Lautropia
g__Lutibacterium‡
g__Marinobacter
g__Microbulbifer
g__Mycobacterium
g__Nocardia
g__Novosphingobium
g__Pleomorphomonas
g__Pseudomonas
g__Psychrobacter
g__Ralstonia
g__Rhodococcus
g__Sphingobium
g__Sphingomonas
g__Williamsia
g__ZD0117
unclassified

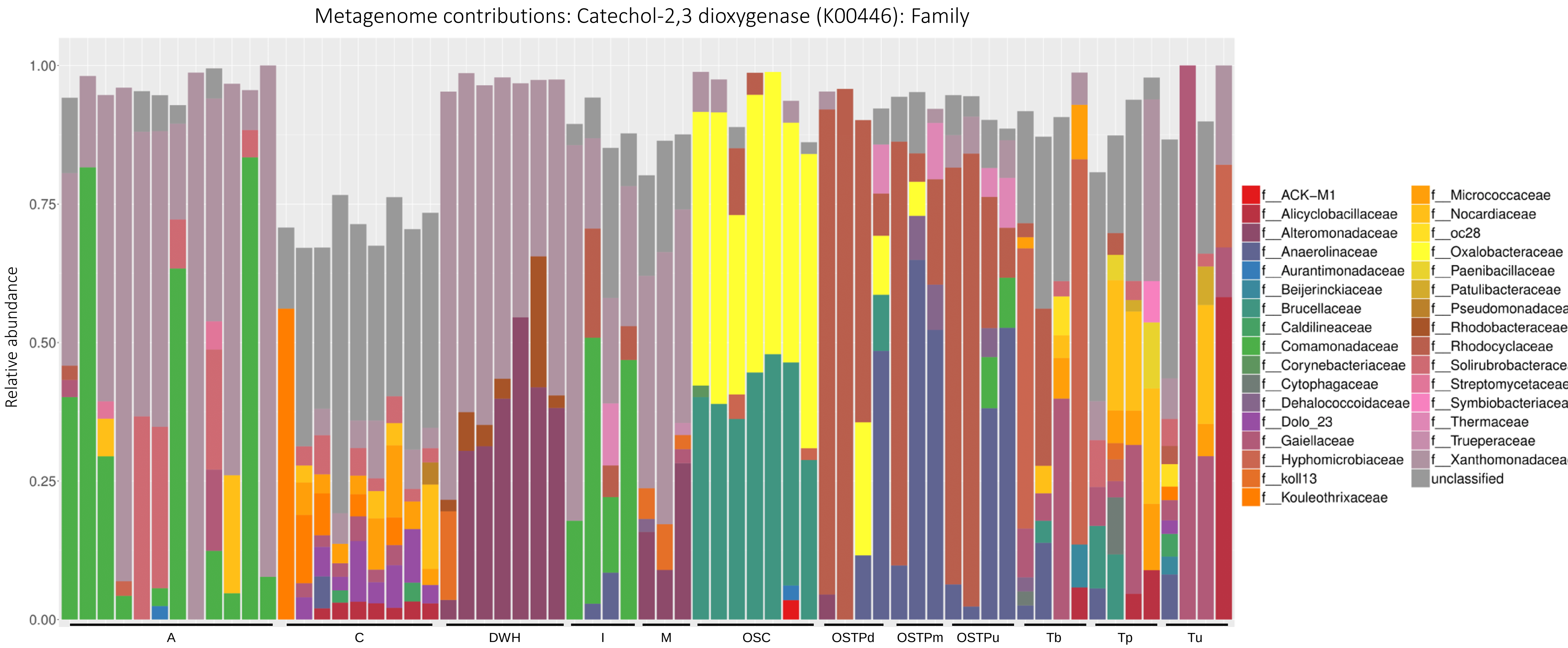

Metagenome contributions: Catechol-2,3 dioxygenase (K00446): Family
Relative abundance
1.00
0.75
0.50
0.25
0.00
A
C
DWH
I
M
OSC
OSTPd
OSTPm
OSTPu
Tb
Tp
Tu
f__ACK-M1
f__Alicyclobacillaceae
f__Alteromonadaceae
f__Anaerolinaceae
f__Aurantimonadaceae
f__Beijerinckiaceae
f__Brucellaceae
f__Caldilineaceae
f__Comamonadaceae
f__Corynebacteriaceae
f__Cytophagaceae
f__Dehalococcoidaceae
f__Dolo_23
f__Gaiellaceae
f__Hyphomicrobiaceae
f__koll13
f__Kouleothrixaceae
f__Micrococcaceae
f__Nocardiaceae
f__oc28
f__Oxalobacteraceae
f__Paenibacillaceae
f__Patulibacteraceae
f__Pseudomonadacea
f__Rhodobacteraceae
f__Rhodocyclaceae
f__Solirubrobacterace
f__Streptomycetaceae
f__Symbiobacteriacea
f__Thermaceae
f__Trueperaceae
f__Xanthomonadacea
unclassified

Metagenome contributions: Catechol-2,3 dioxygenase (K00446): Genus

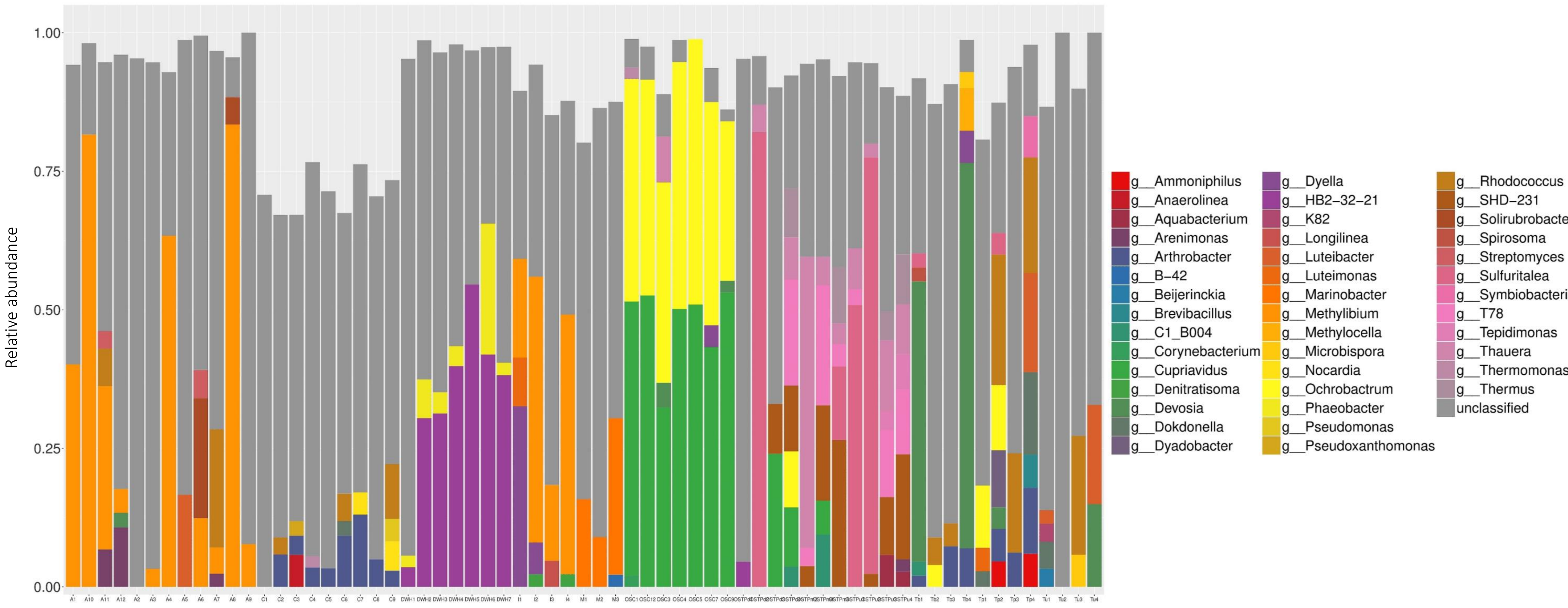

Metagenome contributions: Protocatechuate-3,4 dioxygenase α chain (K00448): Family

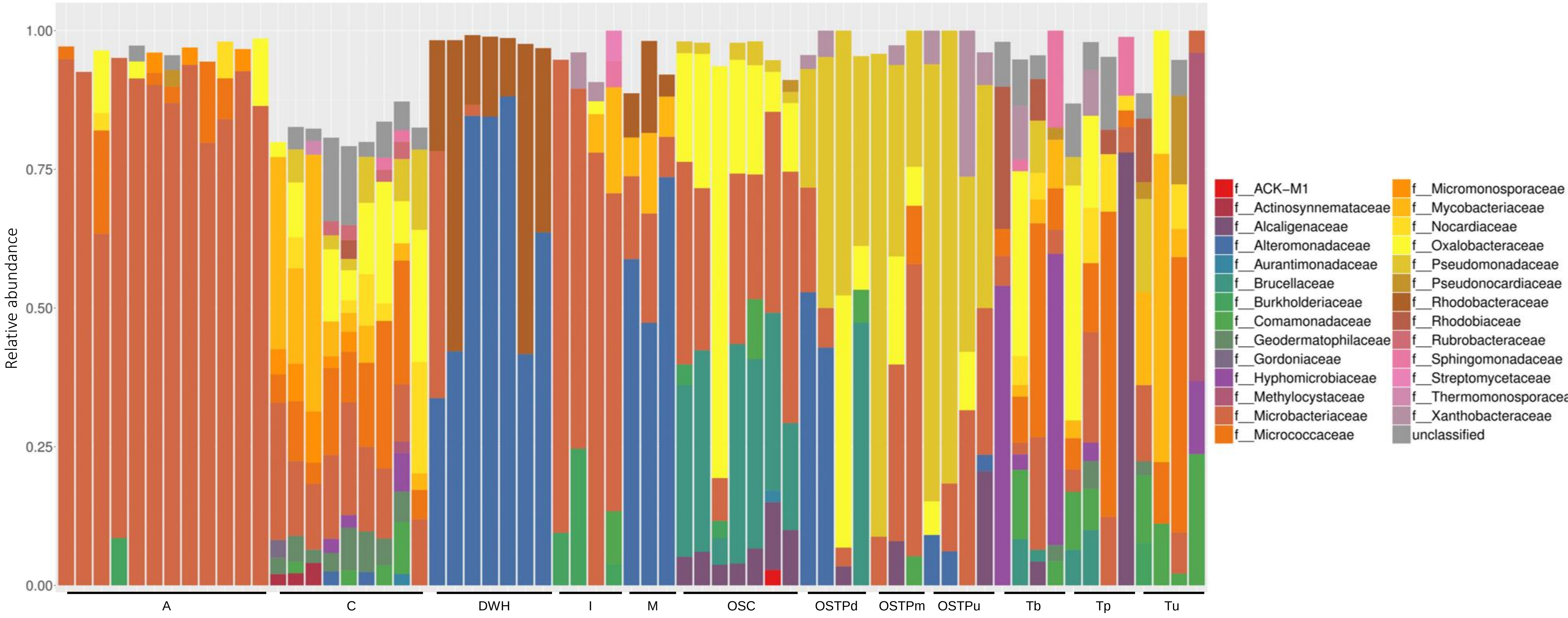

Metagenome contributions: Protocatechuate-3,4 dioxygenase α chain (K00448): Genus

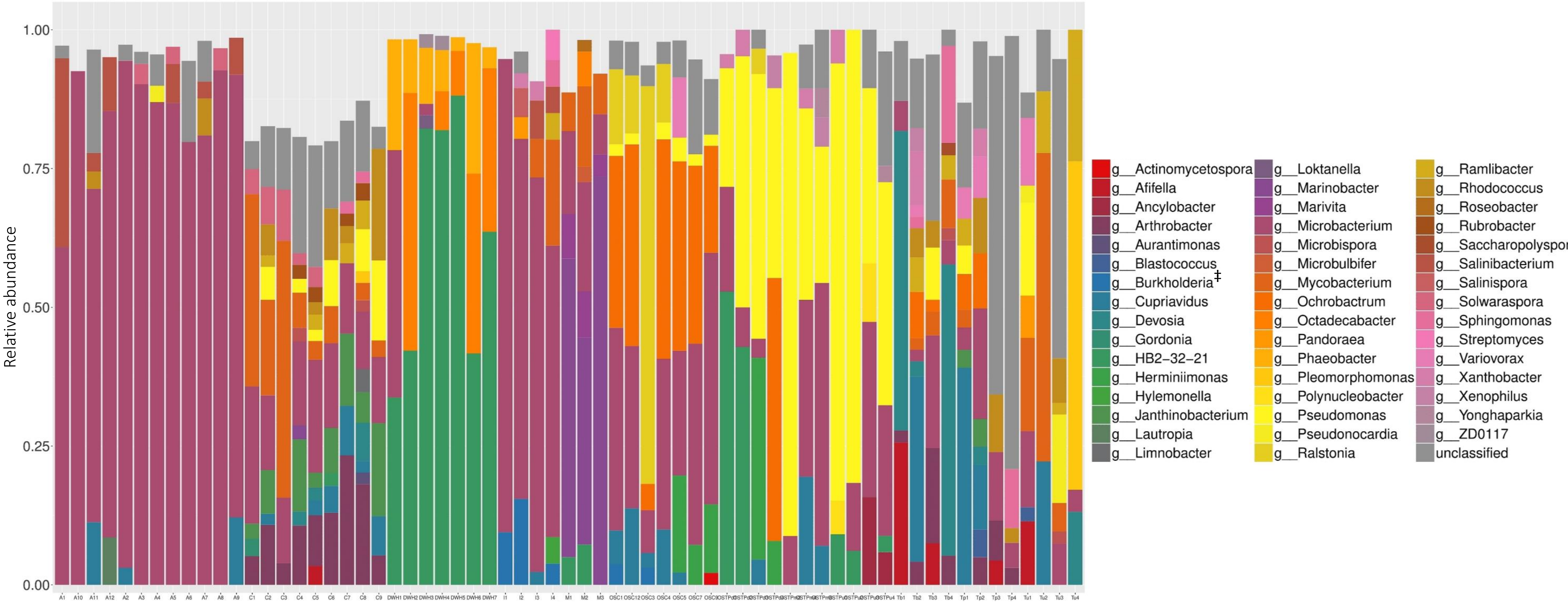

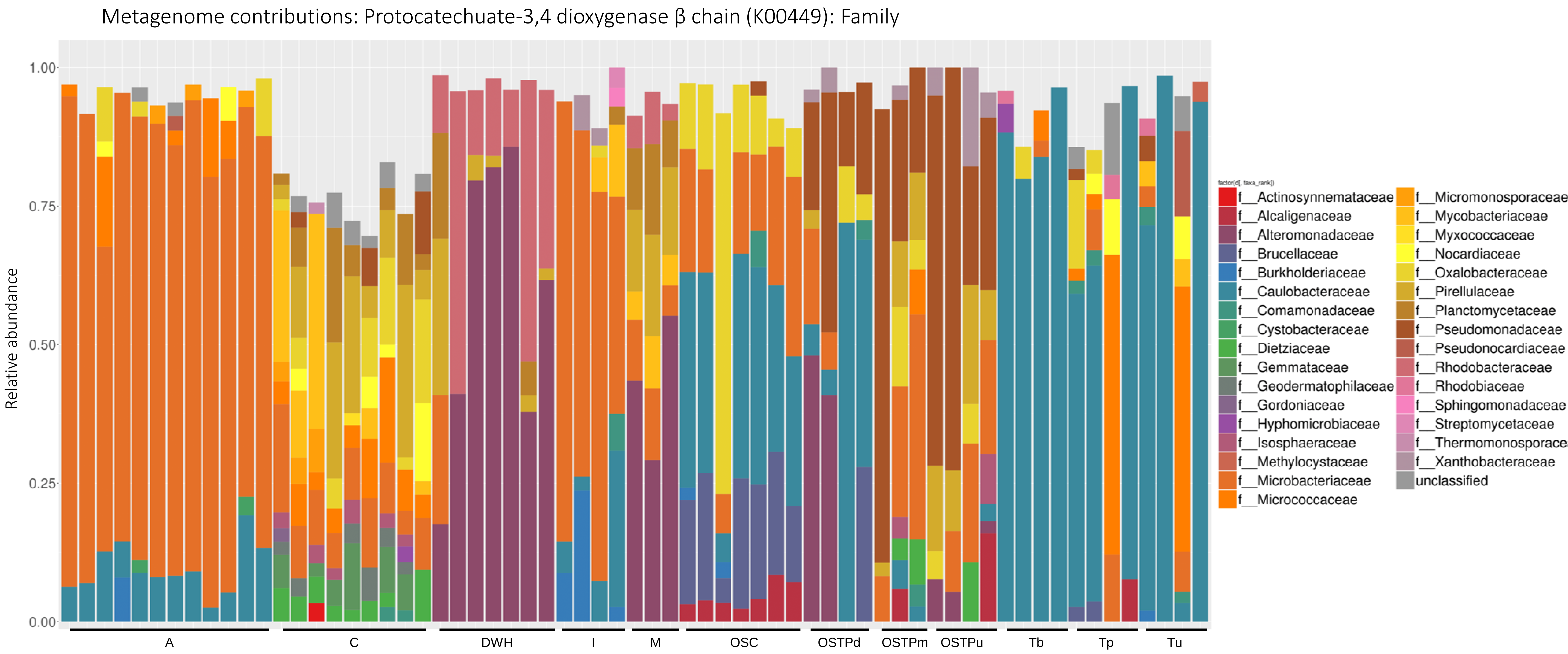
Metagenome contributions: Protocatechuate-3,4 dioxygenase β chain (K00449): Family
Relative abundance
1.00
0.75
0.50
0.25
0.00
A
C
DWH
I
M
OSC
OSTPd
OSTPm
OSTPu
Tb
Tp
Tu
factor(d[, taxa_rank])
f__Actinosynnemataceae
f__Alcaligenaceae
f__Alteromonadaceae
f__Brucellaceae
f__Burkholderiaceae
f__Caulobacteraceae
f__Comamonadaceae
f__Cystobacteraceae
f__Dietziaceae
f__Gemmataceae
f__Geodermatophilaceae
f__Gordoniaceae
f__Hyphomicrobiaceae
f__Isosphaeraceae
f__Methylocystaceae
f__Microbacteriaceae
f__Micrococcaceae
f__Micromonosporaceae
f__Mycobacteriaceae
f__Myxococcaceae
f__Nocardiaceae
f__Oxalobacteraceae
f__Pirellulaceae
f__Planctomycetaceae
f__Pseudomonadaceae
f__Pseudonocardiaceae
f__Rhodobacteraceae
f__Rhodobiaceae
f__Sphingomonadaceae
f__Streptomycetaceae
f__Thermomonosporace
f__Xanthobacteraceae
unclassified

Metagenome contributions: Protocatechuate-3,4 dioxygenase β chain (K00449): Genus

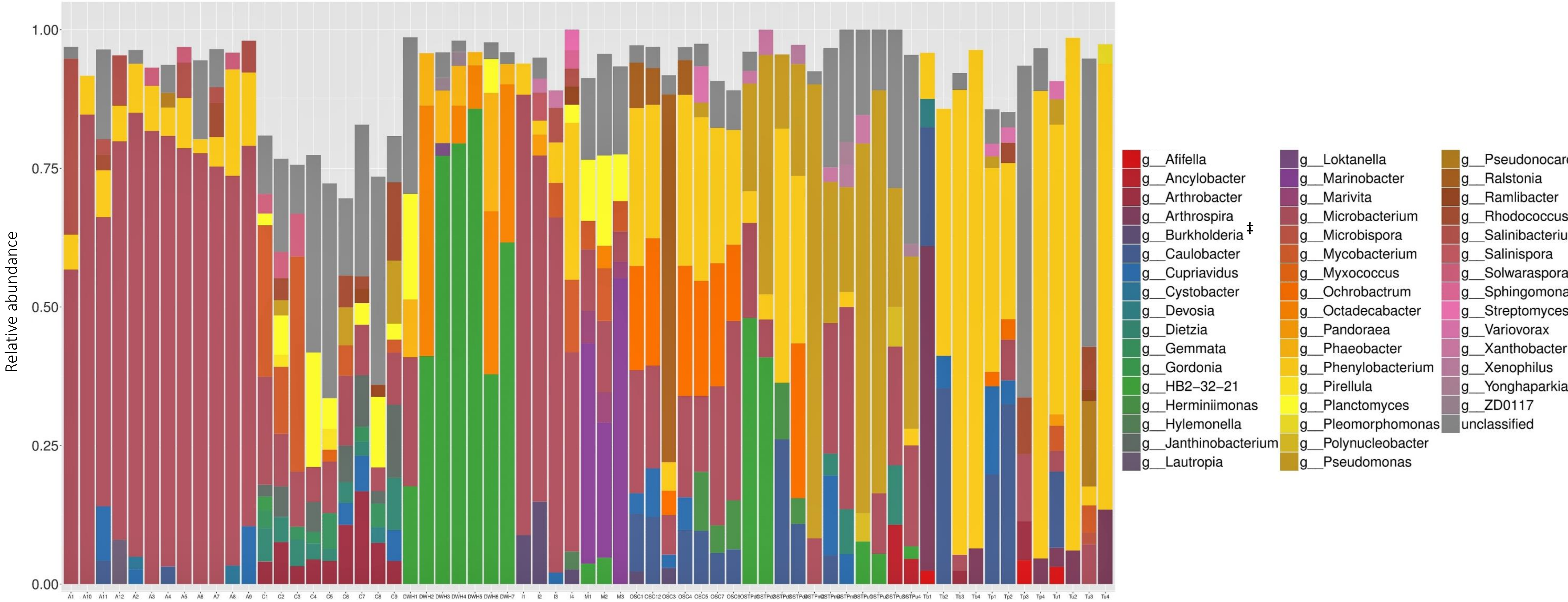

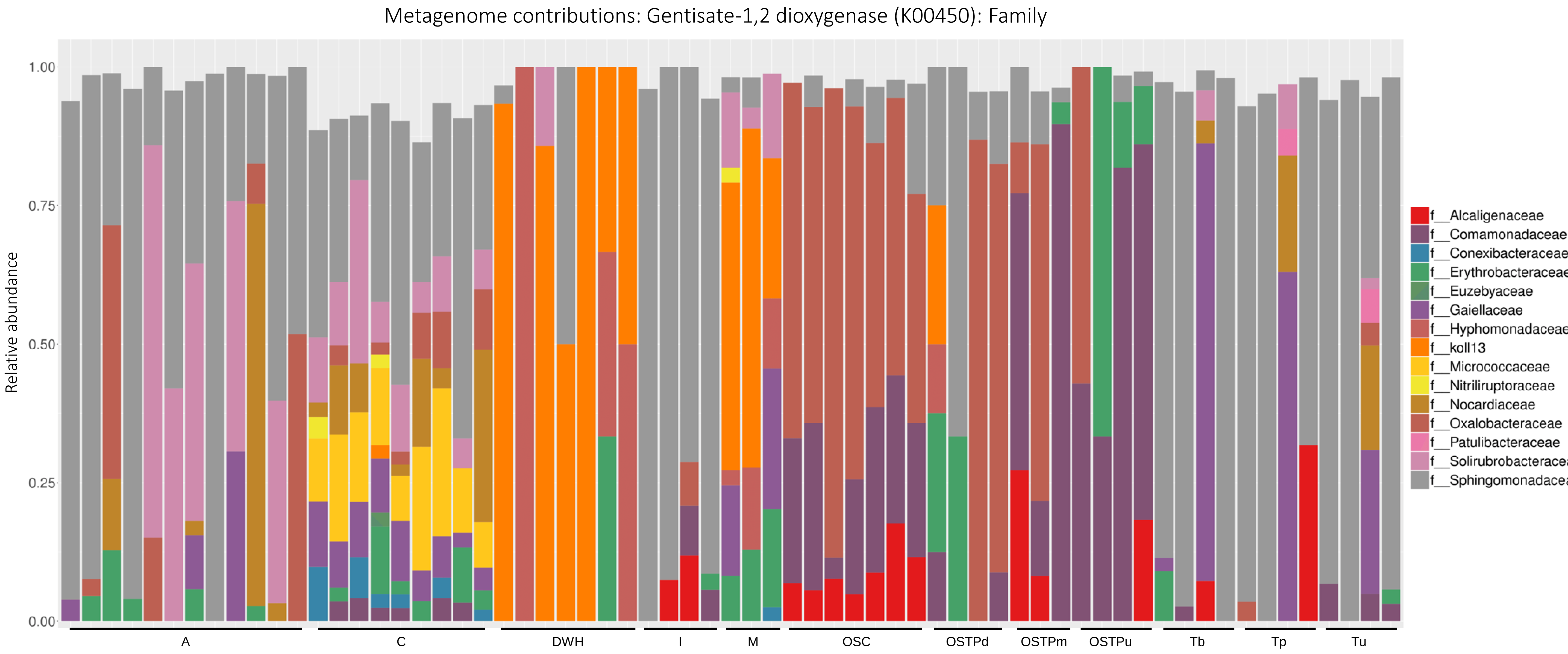
Metagenome contributions: Gentisate-1,2 dioxygenase (K00450): Family
Relative abundance
1.00
0.75
0.50
0.25
0.00
A
C
DWH
I
M
OSC
OSTPd
OSTPm
OSTPu
Tb
Tp
Tu
f__Alcaligenaceae
f__Comamonadaceae
f__Conexibacteraceae
f__Erythrobacteraceae
f__Euzebyaceae
f__Gaiellaceae
f__Hyphomonadaceae
f__koll13
f__Micrococcaceae
f__Nitriliruptoraceae
f__Nocardiaceae
f__Oxalobacteraceae
f__Patulibacteraceae
f__Solirubrobacterace
f__Sphingomonadacea

Metagenome contributions: Gentisate-1,2 dioxygenase (K00450): Genus

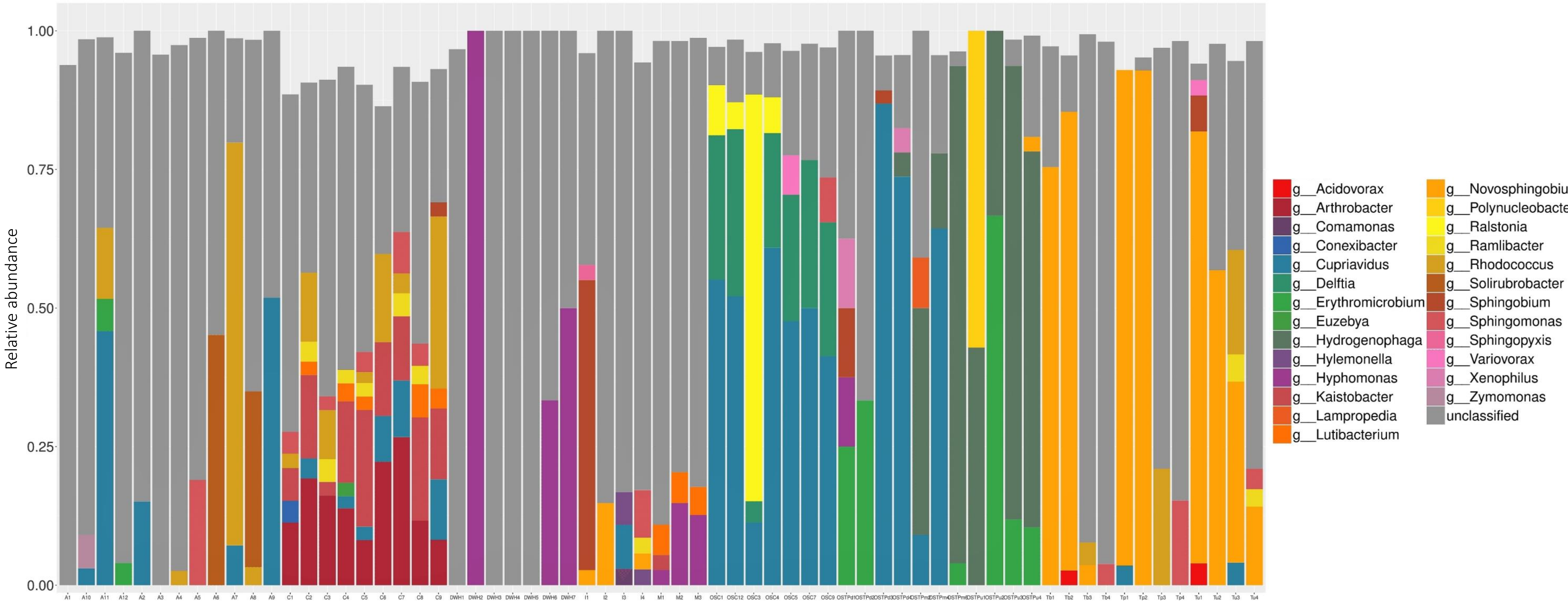

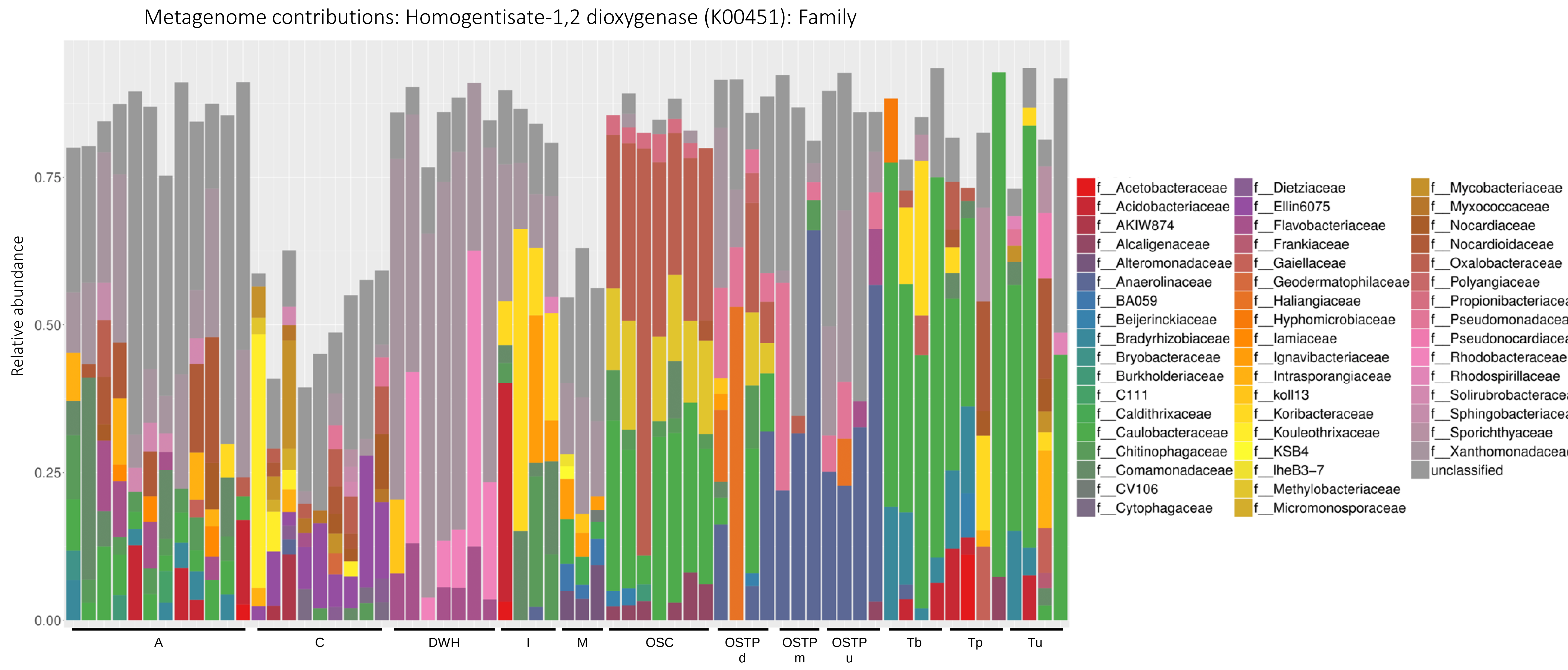
Metagenome contributions: Homogentisate-1,2 dioxygenase (K00451): Family
Relative abundance
0.00
0.25
0.50
0.75
A
C
DWH
I
M
OSC
OSTP d
OSTP m
OSTP u
Tb
Tp
Tu
f__Acetobacteraceae
f__Acidobacteriaceae
f__AKIW874
f__Alcaligenaceae
f__Alteromonadaceae
f__Anaerolinaceae
f__BA059
f__Beijerinckiaceae
f__Bradyrhizobiaceae
f__Bryobacteraceae
f__Burkholderiaceae
f__C111
f__Caldithrixaceae
f__Caulobacteraceae
f__Chitinophagaceae
f__Comamonadaceae
f__CV106
f__Cytophagaceae
f__Dietziaceae
f__Ellin6075
f__Flavobacteriaceae
f__Frankiaceae
f__Gaiellaceae
f__Geodermatophilaceae
f__Haliangiaceae
f__Hyphomicrobiaceae
f__Iamiaceae
f__Ignavibacteriaceae
f__Intrasporangiaceae
f__koll13
f__Koribacteraceae
f__Kouleothrixaceae
f__KSB4
f__lheB3−7
f__Methylobacteriaceae
f__Micromonosporaceae
f__Mycobacteriaceae
f__Myxococcaceae
f__Nocardiaceae
f__Nocardioidaceae
f__Oxalobacteraceae
f__Polyangiaceae
f__Propionibacterіace
f__Pseudomonadacea
f__Pseudonocardiacea
f__Rhodobacteraceae
f__Rhodospirillaceae
f__Solirubrobacterace
f__Sphingobacteriace
f__Sporichthyaceae
f__Xanthomonadacea
unclassified

Metagenome contributions: Homogentisate-1,2 dioxygenase (K00451): Genus

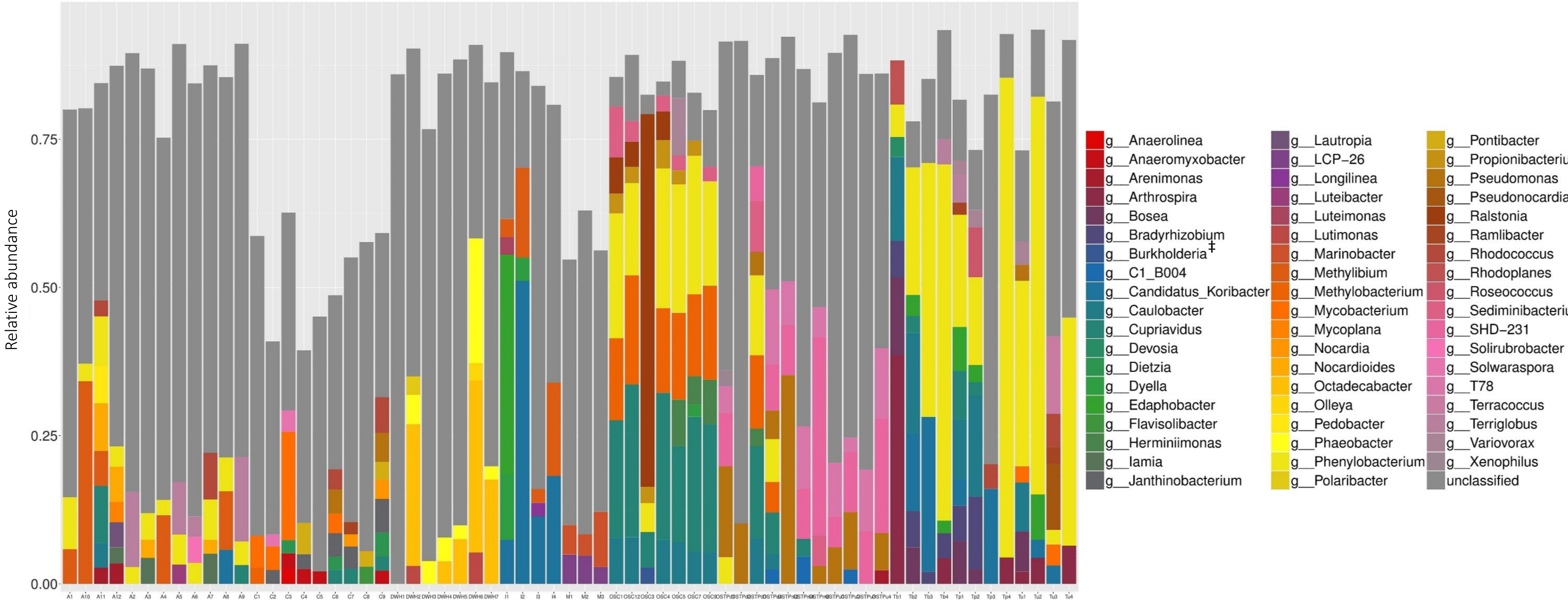

Supplementary data. Figure S6. Comparison between taxonomic and functional characterization of Marine sediments (DWH) carried out by Mason et al.[54] and in this study. (A) Relative abundances of taxonomic clades identified as biomarkers in this study were compared with relative abundances in the parent study by Mason et al.,(B) Relative abundances of 110 KEGG orthologs implicated in xenobiotics degradation as per the KEGG BRITE hierarchy were compared for shotgun sequencing metagenomes and PICRUSt predicted metagenomes from the study by Mason et al. and this study respectively, (C) Abundances of 8 select KEGG orthologs were compared for metagenomic and PICRUST predicted data from the study by Mason et al. and this study respectively using counts normalized for 1,000,000 reads. All boxplots were computed in R using the ggplot2 package.

(A)

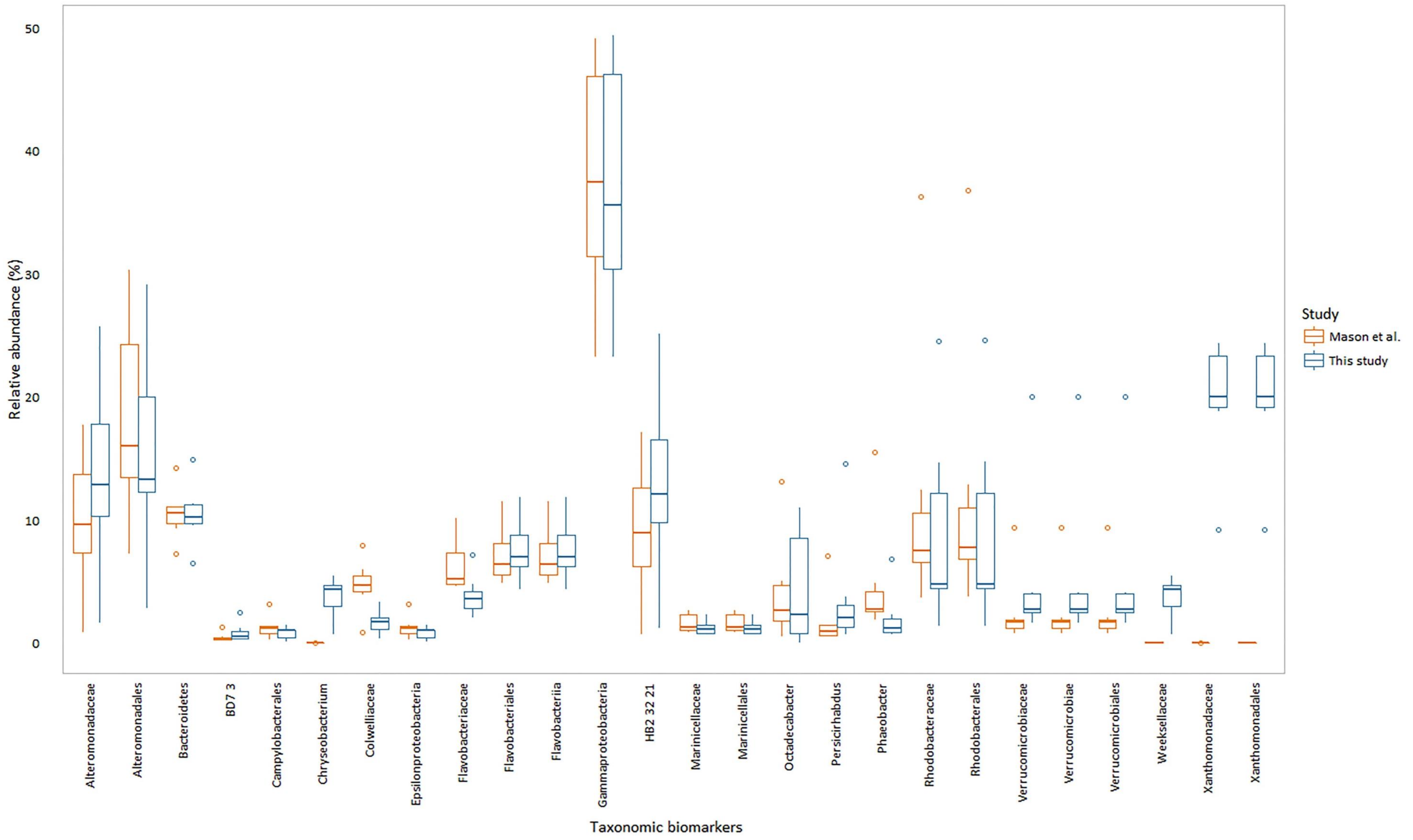

Relative abundance (%)
0
10
20
30
40
50
Study
Mason et al.
This study
Alteromonadaceae
Alteromonadales
Bacteroidetes
BD7 3
Campylobacterales
Chryseobacterium
Colwelliaceae
Epsilonproteobacteria
Flavobacteriaceae
Flavobacteriales
Flavobacteriia
Gammaproteobacteria
HB2 32 21
Marinicellaceae
Marinicellales
Octadecabacter
Persicirhabdus
Phaeobacter
Rhodobacteraceae
Rhodobacterales
Verrucomicrobiaceae
Verrucomicrobiae
Verrucomicrobiales
Weeksellaceae
Xanthomonadaceae
Xanthomonadales
Taxonomic biomarkers

(B) (C)

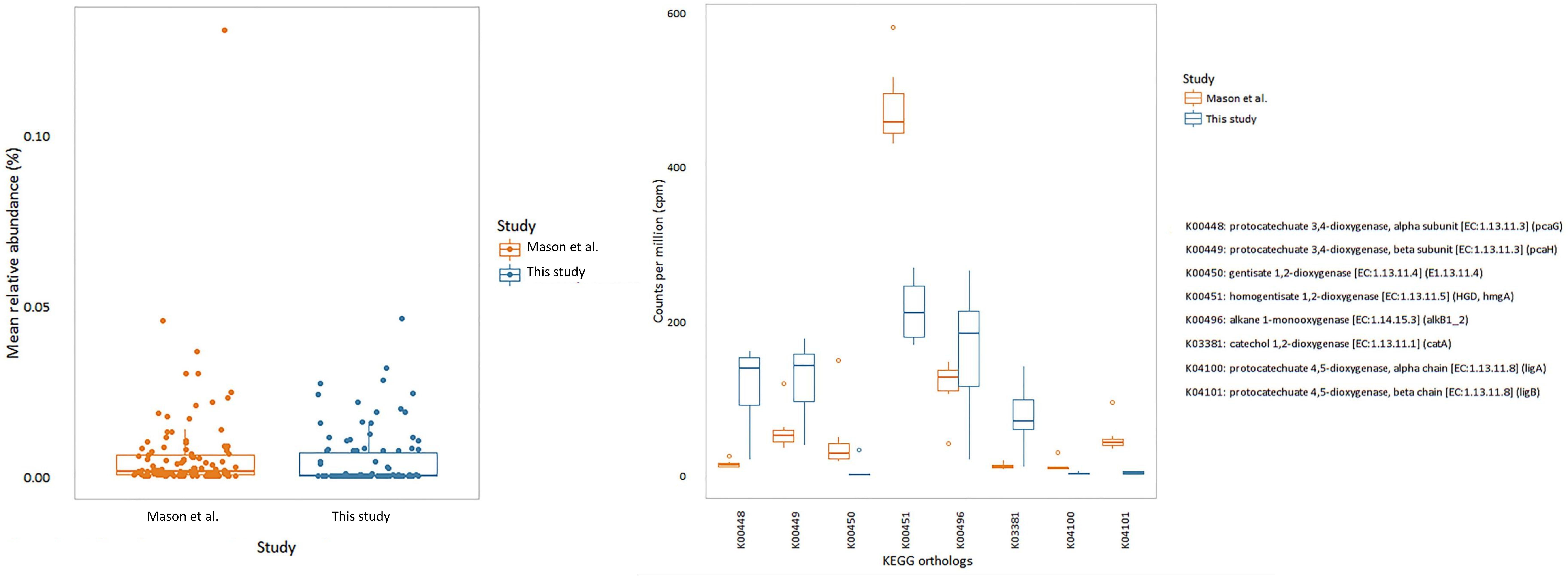

Mean relative abundance (%)
0.10
0.05
0.00
Mason et al.
This study
Study
Study
Mason et al.
This study
Counts per million (cpm)
600
400
200
0
K00448
K00449
K00450
K00451
K00496
K03381
K04100
K04101
KEGG orthologs
Study
Mason et al.
This study
K00448: protocatechuate 3,4-dioxygenase, alpha subunit [EC:1.13.11.3] (pcaG)
K00449: protocatechuate 3,4-dioxygenase, beta subunit [EC:1.13.11.3] (pcaH)
K00450: gentisate 1,2-dioxygenase [EC:1.13.11.4] (E1.13.11.4)
K00451: homogentisate 1,2-dioxygenase [EC:1.13.11.5] (HGD, hmgA)
K00496: alkane 1-monooxygenase [EC:1.14.15.3] (alkB1_2)
K03381: catechol 1,2-dioxygenase [EC:1.13.11.1] (catA)
K04100: protocatechuate 4,5-dioxygenase, alpha chain [EC:1.13.11.8] (ligA)
K04101: protocatechuate 4,5-dioxygenase, beta chain [EC:1.13.11.8] (ligB)

Supplementary data. Table S1. Complete description of 16S rRNA amplicon sequence datasets used in this study.

Biome type	ID	Original ID	Location	Depth of sample collection (cm below surface)	Accession numbers and/or web links for data download		Reference
					SRA Run ID	Web links	
Urban	I1	Noonmati_Surface	Guwahati, Assam, India	0-10	SRR3168574		This study
Urban	I2	Noonmati_Deep	Guwahati, Assam, India	20-30	SRR3168575		This study
Urban	I3	Barhola_Surface	Barhola, Assam, India	0-10	SRR3168576		This study
Urban	I4	Barhola_Deep	Barhola, Assam, India	20-30	SRR3168577		This study
Arctic	A1	AH1d1, AH1d2	Axel Heiburg, Nunavut, Canada	0-15	SRR640619-20		Bell et al. [84]
Arctic	A2	AK1d1, AK1d2, AK1d3	Toolik Lake, Alaska, USA	0-15	SRR640636-38		Bell et al. [84]
Arctic	A3	AL1d1, AL1d2, AL1d3	Alert, Nunavut, Canada	0-15	SRR640662-64		Bell et al. [84]
Arctic	A4	AVKd1, AVKd2, AVKd3	Akulivik, Quebec, Canada	0-15	SRR640689-91		Bell et al. [84]
Arctic	A5	BDEd1, BDEd2, BDEd3	Baie Deception, Quebec, Canada	0-15	SRR640698-700		Bell et al. [84]
Arctic	A6	BY1d1, BY1d2, BY1d3	Bylot Island, Nunavut, Canada	0-15	SRR640707-09		Bell et al. [84]
Arctic	A7	EBAd1, EBAd2, EBAd3	East Bay, Nunavut, Canada	0-15	SRR640725-27		Bell et al. [84]
Arctic	A8	IQAd1, IQAd2, IQAd3	Iqaluit, Nunavut, Canada	0-15	SRR640734-36		Bell et al. [84]
Arctic	A9	NORd1, NORd2, NORd3	Tromso, Norway	0-15	SRR640743-45		Bell et al. [84]
Arctic	A10	RANd1, RANd2, RANd3	Rankin Inlet, Nunavut, Canada	0-15	SRR640752-54		Bell et al. [84]
Arctic	A11	RUSd1, RUSd2, RUSd3	Yamal, Russia	0-15	SRR640761-63		Bell et al. [84]
Arctic	A12	THUd1, THUd2, THUd3	Thule, Greenland	0-15	SRR640770-72		Bell et al. [84]
Urban	C1	CQM1	Changqing, China	2-10	SRX731327		Sun et al. [85]
Urban	C2	CQM2	Changqing, China	2-10	SRX731327		Sun et al. [85]
Urban	C3	CQM3	Changqing, China	2-10	SRX731327		Sun et al. [85]
Urban	C4	DQM3	Daqing, China	2-10	SRX731327		Sun et al. [85]
Urban	C5	DQM4	Daqing, China	2-10	SRX731327		Sun et al. [85]
Urban	C6	DQM5	Daqing, China	2-10	SRX731327		Sun et al. [85]
Urban	C7	DQM12	Daqing, China	2-10	SRX731327		Sun et al. [85]
Urban	C8	DQM50	Daqing, China	2-10	SRX731327		Sun et al. [85]

Urban	C9	DQM200	Daqing, China	2-10	SRX731327		Sun et al. [85]
Mangrove	M1	T23 2% I, T23 2% II	Restinga da Marambaia, Rio de Janeiro, Brazil	0-20	HM602044–HM622061, HQ457546–HQ462469		dos Santos et al. [53]
Mangrove	M2	T66 2% I, T66 2% II	Restinga da Marambaia, Rio de Janeiro, Brazil	0-20	HM602044–HM622061, HQ457546–HQ462469		dos Santos et al. [53]
Mangrove	M3	T23 5% I, T23 5% II	Restinga da Marambaia, Rio de Janeiro, Brazil	0-20	HM602044–HM622061, HQ457546–HQ462469		dos Santos et al. [53]
Marine sediment	DWH1	SE-20101001-GY-LBNL1-BC-120	Gulf of Mexico	0-1[#]		http://mason.eoas.fsu.edu/DWH_sediment/16S_rRNA_gene_iTag_reads/	Mason et al. [54]
Marine sediment	DWH2	SE-20101001-GY-ALTNF001-BC-139	Gulf of Mexico	0-1[#]		http://mason.eoas.fsu.edu/DWH_sediment/16S_rRNA_gene_iTag_reads/	Mason et al. [54]
Marine sediment	DWH3	SE-20101001-GY-NF006MOD-BC-143	Gulf of Mexico	0-1[#]		http://mason.eoas.fsu.edu/DWH_sediment/16S_rRNA_gene_iTag_reads/	Mason et al. [54]
Marine sediment	DWH4	SE-20101017-GY-D031S-BC-278	Gulf of Mexico	0-1[#]		http://mason.eoas.fsu.edu/DWH_sediment/16S_rRNA_gene_iTag_reads/	Mason et al. [54]
Marine sediment	DWH5	SE-20101017-GY-D040S-BC-315	Gulf of Mexico	0-1[#]		http://mason.eoas.fsu.edu/DWH_sediment/16S_rRNA_gene_iTag_reads/	Mason et al. [54]
Marine sediment	DWH6	SE-20101017-GY-D038SW-BC-331	Gulf of Mexico	0-1[#]		http://mason.eoas.fsu.edu/DWH_sediment/16S_rRNA_gene_iTag_reads/	Mason et al. [54]
Marine sediment	DWH7	SE-20101017-GY-D042S-BC-350	Gulf of Mexico	0-1[#]		http://mason.eoas.fsu.edu/DWH_sediment/16S_rRNA_gene_iTag_reads/	Mason et al. [54]
Taiga	Tu1	1330	Walagan, China	20-30	SRR1055249		Yang et al. [13]
Taiga	Tu2	2330	Walagan North, China	20-30	SRR1055249		Yang et al. [13]
Taiga	Tu3	3330	Taiyuan, China	20-30	SRR1055249		Yang et al. [13]
Taiga	Tu4	4330	Jiagedaqi, China	20-30	SRR1055249		Yang et al. [13]
Taiga	Tb1	1530	Walagan, China	70-80	SRR1180575		Yang et al. [13]
Taiga	Tb2	2530	Walagan North, China	70-80	SRR1180576		Yang et al. [13]
Taiga	Tb3	3530	Taiyuan, China	70-80	SRR1180577		Yang et al. [13]
Taiga	Tb4	4530	Jiagedaqi, China	70-80	SRR1180578		Yang et al. [13]
Taiga	Tp1	1630	Walagan, China	140-150	SRR1180579		Yang et al. [13]
Taiga	Tp2	2630	Walagan North, China	140-150	SRR1180580		Yang et al. [13]

Taiga	Tp3	3630	Taiyuan, China	140-150	SRR1180581		Yang et al. [13]
Taiga	Tp4	4630	Jiagedaqi, China	140-150	SRR1180582		Yang et al. [13]
Arctic	OSC1	2010Suncor Oil Sands Core Run 11 Subsample 1	Alberta, Canada	2,985-2,990	SRR573815		An et al. [12]
Arctic	OSC3	2010Suncor Oil Sands Core Run 11 Subsample 3	Alberta, Canada	2,985-2,990	SRR573817		An et al. [12]
Arctic	OSC4	2010Suncor Oil Sands Core Run 11 Subsample 4	Alberta, Canada	2,985-2,990	SRR573818		An et al. [12]
Arctic	OSC5	2010Suncor Oil Sands Core Run 11 Subsample 5	Alberta, Canada	2,985-2,990	SRR573819		An et al. [12]
Arctic	OSC7	2010Suncor Oil Sands Core Run 11 Subsample 7	Alberta, Canada	2,985-2,990	SRR573821		An et al. [12]
Arctic	OSC9	2010Suncor Oil Sands Core Run 11 Subsample 9	Alberta, Canada	2,985-2,990	SRR573823		An et al. [12]
Arctic	OSC12	2010Suncor Oil Sands Core Run 11 Subsample 13	Alberta, Canada	2,985-2,990	SRR573826		An et al. [12]
Arctic	OSTPu1	2009Suncor Tailings Pond 5 (6.5 ft)	Alberta, Canada	200	SRR572718		An et al. [12]
Arctic	OSTPu2	2009Suncor Tailings Pond 5 (8.0 ft)	Alberta, Canada	240	SRR573715		An et al. [12]
Arctic	OSTPu3	2010Syncrude Tailings Pond MLSB M MFT - 1m	Alberta, Canada	100	SRR573789		An et al. [12]
Arctic	OSTPu4	2010Syncrude Tailings Pond MLSB N MFT - 1.1m	Alberta, Canada	110	SRR573780		An et al. [12]
Arctic	OSTPm2	2009Suncor Tailings Pond 5 (25 ft)	Alberta, Canada	750	SRR573718		An et al. [12]
Arctic	OSTPm4	011Suncor Tailings Pond 6 (7 m)	Alberta, Canada	700	SRR573772		An et al. [12]
Arctic	OSTPm6	2010Syncrude Tailings Pond MLSB N MFT - 6.1m	Alberta, Canada	610	SRR573782		An et al. [12]
Arctic	OSTPd1	2009Suncor Tailings Pond 5 (40 ft)	Alberta, Canada	1220	SRR573736		An et al. [12]
Arctic	OSTPd2	2009SuncorTailings Pond 5 (45 ft)	Alberta, Canada	1370	SRR573737		An et al. [12]
Arctic	OSTPd3	2010Suncor Tailings Pond 6 (12 m)	Alberta, Canada	1200	SRR573759		An et al. [12]
Arctic	OSTPd4	2011Suncor Tailings Pond 6 (13 m)	Alberta, Canada	1300	SRR573775		An et al. [12]

[#]All samples collected at an average of ~1500 metres below sea level, depth given is from surface of ocean floor marine sediment.

Supplementary data. Table S2. Taxonomic profile of oil polluted environments as estimated through mothur in the present study. Relative abundances for taxonomic clades were inferred from 16S rRNA data by mothur. Taxonomic classification was carried out using May 2013 release of Greengenes reference taxonomy. Detection of differentially abundant taxa was carried in LEfSe using the non-strict strategy along with a LDA cutoff of 2.0 and p-value < 0.01. Taxa were identified to be significantly differential upon fulfilling the given thresholds, whereby they were attributed to be taxonomic biomarkers for the oil contaminated site exhibiting the highest LDA score for pairwise comparisons. Bacterial clades are coloured by the habitat in which it has been identified to be differentially abundant in by LefSe. Coloring follows the legend give below.

- Arctic (A)
- China oil refineries (C)
- India oil refineries (I)
- Mangrove (M)
- Marine sediments (DWH)
- Oil sands core (OSC)
- Oil sands tailings pond deep (OSTPd)
- Oil sands tailings pond median (OSTPm)
- Oil sands tailings pond upper (OSTPu)
- Taiga bottom active layer (Tb)
- Taiga permafrost layer (Tp)
- Taiga upper active layer (Tu)

Bacterial clades	Habitat																																																																Differentially abundant class (one class test, non-strict)	p-value
	India oil refineries				Arctic												China oil refineries									Mangrove			Marine sediments							Taiga upper active layer				Taiga bottom active layer				Taiga permafrost layer				Oil sands core							Oil sands tailings pond upper				Oil sands tailings pond median			Oil sands tailings pond deep					
	I1	I2	I3	I4	A1	A10	A11	A12	A2	A3	A4	A5	A6	A7	A8	A9	C1	C2	C3	C4	C5	C6	C7	C8	C9	M1	M2	M3	DWH1	DWH2	DWH3	DWH4	DWH5	DWH6	DWH7	Tu1	Tu2	Tu3	Tu4	Tb1	Tb2	Tb3	Tb4	Tp1	Tp2	Tp3	Tp4	OSC1	OSC12	OSC3	OSC4	OSC5	OSC7	OSC9	OSTPu1	OSTPu2	OSTPu4	OSTPu3	OSTPm2	OSTPm6	OSTPm4	OSTPd1	OSTPd2	OSTPd3	OSTPd4		
Bacteria	100	100	100	100	100	100	100	100	100	100	100	100	100	100	100	100	100	100	100	100	100	100	100	100	100	100	100	100	100	100	100	100	100	100	100	100	100	100	100	100	100	100	100	100	100	100	100	100	100	100	100	100	100	100	100	100	100	100	100	100	100	100	100	100	100		
Bacteria|AC1	0	0	0	0	0	0	0	0	0	0	0	0	0	0	0	0	0	0	0	0	0	0	0	0	0	0.117015	0	0.236859	0	0	0	0	0	0	0	0	0	0	0	0	0	0	0	0	0	0	0	0	0	0	0	0	0	0	1.294498	0.385077	0.198926	0.554676	0.837966	0.212119	0.707477	0.055069	0	0.053478	0.540867	Oil sands tailings pond upper	5.91E-08
Bacteria|AC1|TA06	0	0	0	0	0	0	0	0	0	0	0	0	0	0	0	0	0	0	0	0	0	0	0	0	0	0	0	0	0	0	0	0	0	0	0	0	0	0	0	0	0	0	0	0	0	0	0	0	0	0	0	0	0	0	1.194922	0.385077	0.099463	0.554676	0.785593	0.106059	0.707477	0.055069	0	0.053478	0.450723		
Bacteria|Acidobacteria	39.73776	32.37967	5.345568	9.940515	4.284051	7.410186	0.785487	1.237133	32.97257	6.2996	11.13607	20.74643	6.867174	2.57416	26.70946	29.06042	7.101029	14.75239	9.840421	19.11443	22.79357	7.547663	13.58131	15.39242	10.80914	4.563585	5.44522	3.987123	3.198921	0.135591	1.277741	0.665371	0.286417	0.043244	0.216575	4.931358	9.849603	5.978875	16.643	2.260971	7.211158	19.84903	13.29147	5.991175	0.860088	10.18264	1.82685	0	0.103388	0.561575	0	0.406875	0.201815	0.206586	0	0.110022	0.198926	0.277338	0.314237	0.901504	0	0.220276	0.230357	0.106956	0	India oil refineries	1.35E-06
Bacteria|Acidobacteria|Acidobacteria-6	0	0.077463	0	0.042663	0	0	0.098186	0	0	0	0	0.043132	0	0.093606	0	0.041574	1.975121	6.516638	2.914116	11.74546	9.999067	2.217392	6.00943	5.050096	3.028243	0.7801	0.243816	0.592147	0.456989	0	0.047324	0	0	0.043244	0	0.047417	0	0.044288	0	0.051386	0.259084	0	0	0.043731	0	0.081137	0	0	0	0	0	0	0	0	0	0	0	0.092446	0	0	0	0	0	0	0	China oil refineries	3.15E-05
Bacteria|Acidobacteria|Acidobacteria-6|CCU21	0	0	0	0	0	0	0	0	0	0	0	0	0	0	0	0	0	0.439773	0.253401	0.336653	0.537584	0.042642	0.160251	0.103769	0	0.195025	0.20318	0.039476	0.091398	0	0	0	0	0	0	0	0	0.044288	0	0	0.086361	0	0	0	0	0.040568	0	0	0	0	0	0	0	0	0	0	0	0	0	0	0	0	0	0	0		
Bacteria|Acidobacteria|Acidobacteria-6|iii1-15	0	0.077463	0	0.042663	0	0	0.098186	0	0	0	0	0.043132	0	0.093606	0	0.041574	1.975121	6.076865	2.660715	11.40881	9.461483	2.132108	5.849179	4.946327	3.028243	0.429055	0	0.513194	0	0	0	0	0	0	0	0.047417	0	0	0	0.051386	0.172722	0	0	0.043731	0	0.040568	0	0	0	0	0	0	0	0	0	0	0	0.092446	0	0	0	0	0	0	0	China oil refineries	3.29E-05
Bacteria|Acidobacteria|Acidobacteria-6|iii1-15|mb2424	0	0	0	0	0	0	0	0	0	0	0	0	0	0	0	0	0.188107	0.919526	0.168934	1.795485	1.935303	0.213211	1.201886	0.726384	0.546766	0.039005	0	0	0	0	0	0	0	0	0	0	0	0	0	0	0	0	0	0	0	0	0	0	0	0	0	0	0	0	0	0	0	0	0	0	0	0	0	0	0	China oil refineries	9.82E-09
Bacteria|Acidobacteria|Acidobacteria-6|iii1-15|RB40	0	0	0	0	0	0	0	0	0	0	0	0	0	0	0	0	0.094053	0.519732	0.211168	1.271802	0.286712	0.127926	0.120189	0.103769	0.126177	0	0	0.039476	0	0	0	0	0	0	0	0	0	0	0	0	0.043181	0	0	0	0	0	0	0	0	0	0	0	0	0	0	0	0	0.092446	0	0	0	0	0	0	0		
Bacteria|Acidobacteria|Acidobacteriia	38.77722	29.66846	4.60675	8.361979	0.838184	1.447967	0	0	23.84732	0	0.552202	13.54341	2.838432	0	10.54982	22.86585	0.658374	0.319835	0.253401	0.523683	0.286712	0.682275	0.56088	0.207538	0.084118	0	0	0.078953	0	0	0	0	0	0	0	1.327673	9.153166	2.657278	16.60129	1.336028	5.354393	16.01552	13.29147	4.810432	0.430044	5.192743	1.82685	0	0	0.44926	0	0	0	0	0	0	0	0	0	0	0	0	0	0	0	India oil refineries	2.28E-06
Bacteria|Acidobacteria|Acidobacteriia|Acidobacteriales	38.77722	29.66846	4.60675	8.361979	0.838184	1.447967	0	0	23.84732	0	0.552202	13.54341	2.838432	0	10.54982	22.86585	0.658374	0.319835	0.253401	0.523683	0.286712	0.682275	0.56088	0.207538	0.084118	0	0	0.078953	0	0	0	0	0	0	0	1.327673	9.153166	2.657278	16.60129	1.336028	5.354393	16.01552	13.29147	4.810432	0.430044	5.192743	1.82685	0	0	0.44926	0	0	0	0	0	0	0	0	0	0	0	0	0	0	0	India oil refineries	2.28E-06
Bacteria|Acidobacteria|Acidobacteriia|Acidobacteriales|Acidobacteriaceae	25.18741	0.077463	0.17384	0	0.372526	0	0	0	6.489067	0	0.092034	3.881868	1.007185	0	0.334915	7.899113	0	0	0	0	0	0	0.080126	0	0	0	0	0	0	0	0	0	0	0	0	0.948338	3.979638	0.885759	0	1.336028	0.863612	0	3.346105	3.148647	0.258026	0.649093	0.967156	0	0	0	0	0	0	0	0	0	0	0	0	0	0	0	0	0	0	India oil refineries	0.000167
Bacteria|Acidobacteria|Acidobacteriia|Acidobacteriales|Acidobacteriaceae|Edaphobacter	25.11626	0.077463	0.08692	0	0	0	0	0	0.081113	0	0	0	0	0	0	0	0	0	0	0	0	0	0.080126	0	0	0	0	0	0	0	0	0	0	0	0	0.758671	3.979638	0.885759	0	0.822171	0.863612	0	1.115368	1.924173	0.258026	0.48682	0.967156	0	0	0	0	0	0	0	0	0	0	0	0	0	0	0	0	0	0	India oil refineries	9.35E-06
Bacteria|Acidobacteria|Acidobacteriia|Acidobacteriales|Acidobacteriaceae|Edaphobacter|modestum	8.2535	0	0.08692	0	0	0	0	0	0.081113	0	0	0	0	0	0	0	0	0	0	0	0	0	0.080126	0	0	0	0	0	0	0	0	0	0	0	0	0.758671	3.979638	0.885759	0	0.822171	0.863612	0	1.115368	1.924173	0.258026	0.48682	0.967156	0	0	0	0	0	0	0	0	0	0	0	0	0	0	0	0	0	0	India oil refineries	2.48E-05
Bacteria|Acidobacteria|Acidobacteriia|Acidobacteriales|Acidobacteriaceae|Terriglobus	0.071151	0	0.08692	0	0.372526	0	0	0	6.407954	0	0.092034	3.881868	1.007185	0	0.334915	7.899113	0	0	0	0	0	0	0	0	0	0	0	0	0	0	0	0	0	0	0	0.189668	0	0	0	0.513857	0	0	2.230737	1.224474	0	0.162273	0	0	0	0	0	0	0	0	0	0	0	0	0	0	0	0	0	0	0	Arctic	0.007654
Bacteria|Acidobacteria|Acidobacteriia|Acidobacteriales|Koribacteraceae	5.051711	26.56992	4.259071	8.106	0.093132	0.425873	0	0	0.648907	0	0.184067	0.086264	0.274687	0	2.679319	0.665188	0.470267	0.079959	0	0.074812	0.071678	0.255853	0.160251	0.138359	0	0	0	0.078953	0	0	0	0	0	0	0	0.379335	1.691346	1.594367	0.750812	0	3.195363	14.39693	0.278842	1.137011	0	4.05683	0	0	0	0.112315	0	0	0	0	0	0	0	0	0	0	0	0	0	0	0	India oil refineries	4.7E-05
Bacteria|Acidobacteria|Acidobacteriia|Acidobacteriales|Koribacteraceae|Candidatus_Koribacter	5.051711	26.56992	4.259071	8.106	0.093132	0.425873	0	0	0.648907	0	0.184067	0.086264	0.274687	0	2.679319	0.665188	0.470267	0.079959	0	0.074812	0.071678	0.255853	0.160251	0.138359	0	0	0	0.078953	0	0	0	0	0	0	0	0.379335	1.691346	1.594367	0.750812	0	3.195363	14.39693	0.278842	1.137011	0	4.05683	0	0	0	0.112315	0	0	0	0	0	0	0	0	0	0	0	0	0	0	0	India oil refineries	4.7E-05
Bacteria|Acidobacteria|Acidobacteriia|Acidobacteriales|Koribacteraceae|Candidatus_Koribacter|versatilis	0	0.387317	0	0	0	0	0	0	0	0	0	0	0	0	0	0	0.094053	0	0	0	0	0	0	0	0	0	0	0	0	0	0	0	0	0	0	0	0.099491	0	0.583965	0	0	0	0.092947	0	0	0	0	0	0	0	0	0	0	0	0	0	0	0	0	0	0	0	0	0	0		
Bacteria|Acidobacteria|BPC102	0	0	0.04346	0.042663	0	0	0	0	0	0	0	0	0	0	0	0	0.094053	0.079959	0.042234	0.224436	0.071678	0.085284	0	0.03459	0.042059	0.585075	0.528268	0.631623	0.182795	0	0	0.047527	0	0	0	0	0	0	0	0	0	0	0	0	0	0	0	0	0	0	0	0	0	0	0	0	0	0	0	0	0	0	0	0	0		
Bacteria|Acidobacteria|BPC102|B110	0	0	0	0	0	0	0	0	0	0	0	0	0	0	0	0	0.094053	0	0	0.18703	0	0	0	0	0	0.54607	0.487632	0.552671	0.182795	0	0	0.047527	0	0	0	0	0	0	0	0	0	0	0	0	0	0	0	0	0	0	0	0	0	0	0	0	0	0	0	0	0	0	0	0	0		
Bacteria|Acidobacteria|Chloracidobacteria	0	0	0	0	0.279395	0.085175	0	0	0.081113	0.094024	2.945076	0.172527	0.183125	0.093606	0.251186	0	3.197814	5.677071	2.95635	4.862771	10.3933	3.496657	5.288298	8.370707	6.308839	0.23403	0	0.236859	0	0	0	0	0	0	0	0.284501	0	0.177152	0	0	0.086361	0.340756	0	0.087462	0.086009	0	0	0	0	0	0	0	0	0.206586	0	0	0	0	0	0	0	0	0	0	0	China oil refineries	8.55E-06
Bacteria|Acidobacteria|Chloracidobacteria|11-24	0	0	0	0	0	0	0	0	0.081113	0	0.092034	0	0	0	0	0	0.56432	0	0.084467	0.149624	0.143356	0	0.080126	0.069179	0.168236	0.07801	0	0	0	0	0	0	0	0	0	0	0	0	0	0	0	0	0	0	0	0	0	0	0	0	0	0	0	0	0	0	0	0	0	0	0	0	0	0	0		
Bacteria|Acidobacteria|Chloracidobacteria|DS-100	0	0	0	0	0	0	0	0	0	0.094024	0	0	0	0	0	0	0.188107	0.319835	0.253401	0.448871	0.501745	0.085284	0.160251	0.276718	0.084118	0	0	0	0	0	0	0	0	0	0	0	0	0	0	0	0	0	0	0	0	0	0	0	0	0	0	0	0	0	0	0	0	0	0	0	0	0	0	0	0		
Bacteria|Acidobacteria|Chloracidobacteria|RB41	0	0	0	0	0	0	0	0	0	0	0	0	0	0	0	0	1.316747	3.678103	1.267007	2.169544	5.160809	1.790971	2.083269	6.987119	4.878835	0	0	0	0	0	0	0	0	0	0	0	0	0	0	0	0	0	0	0	0.086009	0	0	0	0	0	0	0	0	0.206586	0	0	0	0	0	0	0	0	0	0	0	China oil refineries	6.76E-08
Bacteria|Acidobacteria|Chloracidobacteria|RB41|Ellin6075	0	0	0	0	0	0	0	0	0	0	0	0	0	0	0	0	1.316747	3.678103	1.267007	2.169544	5.160809	1.790971	2.083269	6.987119	4.878835	0	0	0	0	0	0	0	0	0	0	0	0	0	0	0	0	0	0	0	0.086009	0	0	0	0	0	0	0	0	0.206586	0	0	0	0	0	0	0	0	0	0	0	China oil refineries	6.76E-08
Bacteria|Acidobacteria|DA052	0	0	0	0	0.279395	0.085175	0	0	4.299007	0	0	3.105494	1.19031	0	3.600335	3.824834	0.094053	0.079959	0.084467	0	0.071678	0	0.400629	0.138359	0.168236	0	0.081272	0	0	0	0	0	0	0	0	0	0.099491	0.531456	0	0	0.086361	0.85189	0	0	0.086009	0.730229	0	0	0.103388	0.112315	0	0.406875	0.201815	0	0	0	0	0	0	0	0	0	0	0.106956	0		
Bacteria|Acidobacteria|DA052|Ellin6513	0	0	0	0	0.279395	0.085175	0	0	4.299007	0	0	3.105494	1.19031	0	3.600335	3.824834	0.094053	0.079959	0.084467	0	0.071678	0	0.400629	0.138359	0.168236	0	0.081272	0	0	0	0	0	0	0	0	0	0.099491	0.531456	0	0	0.086361	0.85189	0	0	0.086009	0.730229	0	0	0.103388	0.112315	0	0.406875	0.201815	0	0	0	0	0	0	0	0	0	0	0.106956	0		
Bacteria|Acidobacteria|Holophagae	0	0.92956	0.04346	0	0	1.235031	0	0	0	0	0	0	0	0.046803	1.046609	0	0	0	0	0	0	0	0	0	0	0	0	0	0	0	0	0	0	0	0	1.280257	0	0	0	0	0	0	0	0.087462	0	0	0	0	0	0	0	0	0	0	0	0	0	0	0	0	0	0	0	0	0		
Bacteria|Acidobacteria|Holophagae|Holophagales	0	0.92956	0.04346	0	0	1.235031	0	0	0	0	0	0	0	0.046803	1.046609	0	0	0	0	0	0	0	0	0	0	0	0	0	0	0	0	0	0	0	0	1.280257	0	0	0	0	0	0	0	0.087462	0	0	0	0	0	0	0	0	0	0	0	0	0	0	0	0	0	0	0	0	0		
Bacteria|Acidobacteria|Holophagae|Holophagales|Holophagaceae	0	0.92956	0.04346	0	0	1.235031	0	0	0	0	0	0	0	0.046803	1.046609	0	0	0	0	0	0	0	0	0	0	0	0	0	0	0	0	0	0	0	0	1.280257	0	0	0	0	0	0	0	0.087462	0	0	0	0	0	0	0	0	0	0	0	0	0	0	0	0	0	0	0	0	0		
Bacteria|Acidobacteria|Holophagae|Holophagales|Holophagaceae|Geothrix	0	0.890828	0.04346	0	0	1.235031	0	0	0	0	0	0	0	0.046803	1.046609	0	0	0	0	0	0	0	0	0	0	0	0	0	0	0	0	0	0	0	0	1.280257	0	0	0	0	0	0	0	0.087462	0	0	0	0	0	0	0	0	0	0	0	0	0	0	0	0	0	0	0	0	0		
Bacteria|Acidobacteria|iii1-8	0.035575	0.077463	0.04346	0.127989	0.186263	0.723984	0	0.095164	0	0	0	0	0	0	0.837287	0	0.14108	0.399794	0.295635	0.635901	0.68094	0.085284	0.320503	0.726384	0.462648	0.07801	0.040636	0.039476	0	0	0.047324	0	0	0	0	0.094834	0	1.94867	0.041712	0.154157	0.820431	2.044535	0	0.087462	0.043004	4.178535	0	0	0	0	0	0	0	0	0	0	0	0	0	0	0	0	0	0	0	Taiga permafrost layer	0.000109
Bacteria|Acidobacteria|iii1-8|32-20	0	0	0	0	0	0	0	0	0	0	0	0	0	0	0	0	0.047027	0.039979	0	0.037406	0.215034	0.042642	0.080126	0.03459	0.084118	0	0	0	0	0	0	0	0	0	0	0.094834	0	0.664319	0	0	0.172722	0.298161	0	0.043731	0.043004	0.527388	0	0	0	0	0	0	0	0	0	0	0	0	0	0	0	0	0	0	0		
Bacteria|Acidobacteria|iii1-8|DS-18	0.035575	0.077463	0.04346	0.127989	0	0	0	0.047582	0	0	0	0	0	0	0	0	0.094053	0.359814	0.295635	0.598495	0.465906	0.042642	0.240377	0.691794	0.37853	0.07801	0.040636	0.039476	0	0	0.047324	0	0	0	0	0	0	1.284351	0.041712	0.051386	0.259084	1.746374	0	0	0	3.610579	0	0	0	0	0	0	0	0	0	0	0	0	0	0	0	0	0	0	0	Taiga permafrost layer	8.05E-06
Bacteria|Acidobacteria|iii1-8|SJA-36	0	0	0	0	0.186263	0.723984	0	0.047582	0	0	0	0	0	0	0.837287	0	0	0	0	0	0	0	0	0	0	0	0	0	0	0	0	0	0	0	0	0	0	0	0	0.102771	0.388625	0	0	0.043731	0	0.040568	0	0	0	0	0	0	0	0	0	0	0	0	0	0	0	0	0	0	0		
Bacteria|Acidobacteria|OS-K	0	0	0	0	0	0	0	0	0	0	0	0	0	0	0	0	0	0	0.084467	0	0	0	0	0	0	1.599205	3.33215	1.815917	0	0	0.047324	0.047527	0	0	0.043315	0	0	0	0	0	0	0	0	0	0	0	0	0	0	0	0	0	0	0	0	0	0.198926	0	0	0.795445	0	0	0	0	0		
Bacteria|Acidobacteria|RB25	0	0	0	0	0	0	0	0.047582	0.202783	0	0.13805	0	0	0	0.167457	0	0	0	0	0	0	0	0	0	0	0.663085	0.365724	0.236859	0.319892	0.045197	0.094647	0.14258	0	0	0	0	0	0	0	0	0	0	0	0	0.043004	0	0	0	0	0	0	0	0	0	0	0	0	0	0	0	0	0	0	0	0		
Bacteria|Acidobacteria|Solibacteres	0.924961	1.62673	0.608439	1.365221	2.607683	3.832855	0.687301	0.951641	4.542347	6.205577	6.902521	3.795604	2.563745	2.340146	9.963718	2.32816	0.752427	1.039464	2.702948	0.673307	0.788457	0.767559	0.881383	0.691794	0.588825	0.23403	0.243816	0.157906	0.913977	0.090394	0.662532	0.095053	0	0	0.17326	1.896676	0.596946	0.531456	0	0.7194	0.431806	0.511134	0	0.874624	0.172018	0	0	0	0	0	0	0	0	0	0	0.110022	0	0.184892	0.314237	0.106059	0	0.220276	0.230357	0	0	Arctic	1.43E-06
Bacteria|Acidobacteria|Solibacteres|Solibacterales	0.924961	1.62673	0.608439	1.365221	2.607683	3.832855	0.687301	0.951641	4.542347	6.205577	6.902521	3.795604	2.563745	2.340146	9.79626	2.245011	0.752427	1.039464	2.702948	0.673307	0.788457	0.767559	0.881383	0.691794	0.588825	0.23403	0.243816	0.157906	0.913977	0.090394	0.662532	0.095053	0	0	0.17326	1.896676	0.596946	0.531456	0	0.7194	0.345445	0.511134	0	0.874624	0.172018	0	0	0	0	0	0	0	0	0	0	0.110022	0	0.184892	0.314237	0.106059	0	0.220276	0.230357	0	0	Arctic	1.43E-06
Bacteria|Acidobacteria|Solibacteres|Solibacterales|Bryobacteraceae	0	0	0	0	1.024447	0.170349	0.098186	0.095164	0.081113	0.376096	0	0.172527	0	0.093606	0	0.083149	0	0	0	0	0	0	0	0	0	0	0	0	0	0	0	0	0	0	0	0	0	0	0	0.102771	0	0	0	0	0	0	0	0	0	0	0	0	0	0	0	0	0	0	0	0	0	0	0	0	0		
Bacteria|Acidobacteria|Sva0725	0	0	0	0	0.093132	0	0	0.047582	0	0	0.276101	0	0	0	0	0	0.094053	0.479753	0.380102	0.18703	0.286712	0.127926	0.040063	0.172948	0.084118	0.07801	0.284452	0.078953	0.82258	0	0.094647	0.095053	0.095472	0	0	0	0	0	0	0	0	0	0	0	0	0	0	0	0	0	0	0	0	0	0	0	0	0	0	0	0	0	0	0	0		
Bacteria|Acidobacteria|Sva0725|Sva0725	0	0	0	0	0.093132	0	0	0.047582	0	0	0.276101	0	0	0	0	0	0.094053	0.479753	0.380102	0.18703	0.286712	0.127926	0.040063	0.172948	0.084118	0.07801	0.284452	0.078953	0.82258	0	0.094647	0.095053	0.095472	0	0	0	0	0	0	0	0	0	0	0	0	0	0	0	0	0	0	0	0	0	0	0	0	0	0	0	0	0	0	0	0		
Bacteria|Actinobacteria	5.139803	1.342698	2.475145	1.058453	29.28987	18.62281	36.63737	28.23201	16.17342	25.18833	21.13486	22.80115	49.86658	48.95362	12.85236	13.24834	16.96163	15.94644	36.63179	7.743918	8.383072	17.05971	17.09483	3.897106	13.50772	3.776984	4.964361	3.197594	7.370574	0	0.709856	0.14258	0.381889	0.302709	0.08663	4.631954	0.447709	61.12034	0.236367	1.957795	1.137089	11.18815	0.303628	4.184451	1.411977	24.10047	1.414913	6.323894	4.583543	6.710826	5.9596	3.695783	6.693525	8.777431	0.199154	0.165033	2.619192	2.773382	0.576102	2.91663	1.444433	0.715898	0.115179	0.641734	1.171879	Arctic	5.45E-06
Bacteria|Actinobacteria|Acidimicrobiia	0.035575	0	0.04346	0.255979	0	1.064682	1.32551	1.237133	0.040557	3.431872	3.681345	0.646978	0	3.276204	1.046609	0.249446	0.752427	1.958989	7.728743	1.084772	1.146846	1.407191	3.205029	0.588025	0.967355	1.95025	3.169606	1.3422	5.666659	0	0.425914	0.047527	0.286417	0.129732	0.043315	0.521586	0	0.088576	0	0.154157	0.172722	0.553728	0	0.262387	0.043004	0.202842	0	0	0	0.056158	0	0	0	0	0	0	0.049731	0	0	0.05303	0	0.110138	0	0	0	Mangrove	9.27E-05
Bacteria|Actinobacteria|Acidimicrobiia|Acidimicrobiales	0.035575	0	0.04346	0.255979	0	1.064682	1.32551	1.237133	0.040557	3.431872	3.681345	0.646978	0	3.276204	1.046609	0.249446	0.752427	1.958989	7.728743	1.084772	1.146846	1.407191	3.205029	0.588025	0.967355	1.95025	3.169606	1.3422	5.666659	0	0.425914	0.047527	0.286417	0.129732	0.043315	0.521586	0	0.088576	0	0.154157	0.172722	0.553728	0	0.262387	0.043004	0.202842	0	0	0	0.056158	0	0	0	0	0	0	0.049731	0	0	0.05303	0	0.110138	0	0	0	Mangrove	9.27E-05
Bacteria|Actinobacteria|Acidimicrobiia|Acidimicrobiales|AKIW874	0	0	0.04346	0	0	0	0	0	0	0	0	0	0	0	0	0	0.14108	0.959505	6.0394	0.149624	0.143356	0.127926	0.040063	0.069179	0.252354	0.039005	0	0	0	0	0	0	0	0	0	0.047417	0	0	0	0	0	0	0	0	0	0	0	0	0	0	0	0	0	0	0	0	0	0	0	0	0	0	0	0	0		
Bacteria|Actinobacteria|Acidimicrobiia|Acidimicrobiales|C111	0	0	0	0.042663	0	0	0	0.047582	0	0.141036	1.840672	0	0	0	0	0.083149	0.047027	0.079959	0.084467	0.037406	0.035839	0.042642	0	0	0	0.741095	0.650176	0.355288	0	0	0	0	0	0	0	0	0	0.044288	0	0	0	0	0	0.043731	0	0	0	0	0	0.056158	0	0	0	0	0	0	0	0	0	0	0	0	0	0	0		
Bacteria|Actinobacteria|Acidimicrobiia|Acidimicrobiales|Iamiaceae	0	0	0	0	0	0.638809	0.294558	1.141969	0	1.504382	0.552202	0.043132	0	1.638102	0	0	0.094053	0.199897	0.59127	0.112218	0.107517	0.127926	0.040063	0	0	0	0	0	0	0	0	0	0	0	0	0	0	0	0	0	0	0	0	0	0	0	0	0	0	0	0	0	0	0	0	0	0	0	0	0	0	0	0	0	0	Arctic	0.000147
Bacteria|Actinobacteria|Acidimicrobiia|Acidimicrobiales|Iamiaceae|Iamia	0	0	0	0	0	0.638809	0.294558	1.141969	0	1.504382	0.552202	0.043132	0	1.638102	0	0	0.094053	0.199897	0.59127	0.112218	0.107517	0.127926	0.040063	0	0	0	0	0	0	0	0	0	0	0	0	0	0	0	0	0	0	0	0	0	0	0	0	0	0	0	0	0	0	0	0	0	0	0	0	0	0	0	0	0	0	Arctic	0.000147
Bacteria|Actinobacteria|Acidimicrobiia|Acidimicrobiales|koll13	0	0	0	0	0	0	0	0	0	0	0	0	0	0	0	0	0	0	0	0.112218	0	0	0	0	0	0.741095	1.340987	0.394765	5.163972	0	0.283942	0.047527	0.238681	0.043244	0.043315	0	0	0	0	0	0	0	0	0	0.043004	0	0	0	0	0	0	0	0	0	0	0	0	0	0	0	0	0.110138	0	0	0		
Bacteria|Actinobacteria|Acidimicrobiia|Acidimicrobiales|Microthrixaceae	0	0	0	0	0	0	0	0	0	0	0	0	0	0	0	0	0	0	0	0	0	0	0	0	0	0.15602	0.528268	0.236859	0	0	0.047324	0	0	0	0	0	0	0	0	0	0	0	0	0	0	0	0	0	0	0	0	0	0	0	0	0	0	0	0	0	0	0	0	0	0		
Bacteria|Actinobacteria|Actinobacteria	5.033077	1.342698	2.214386	0.802474	28.87078	17.17484	34.96821	26.99488	13.69946	19.87598	15.70488	22.06791	47.3944	45.53701	11.05219	12.99889	14.04597	11.74861	25.43989	4.676632	4.906694	13.60569	10.52452	2.444339	10.5636	1.358674	1.672848	1.184294	1.521119	0	0.236619	0.095053	0.095472	0.172977	0.043315	3.351698	0.348218	53.50281	0.152943	1.084238	0.48938	6.460162	0.303628	2.785053	1.23996	18.74545	1.414913	6.323894	4.583543	6.373881	5.9596	3.644924	6.693525	8.777431	0.049788	0.110022	0.530469	0.138669	0.36661	0.901504	0.736955	0.60576	0.057589	0.267389	0.090145	Arctic	5.98E-07
Bacteria|Actinobacteria|Actinobacteria|Actinomycetales	5.033077	1.342698	2.214386	0.75981	28.87078	17.17484	34.96821	26.99488	13.69946	19.87598	15.70488	22.06791	47.3944	45.53701	11.05219	12.99889	13.99895	11.62867	25.35542	4.564414	4.763338	13.60569	10.52452	2.409749	10.5636	1.319669	1.591576	1.184294	1.521119	0	0.236619	0.095053	0.095472	0.172977	0.043315	3.351698	0.348218	53.50281	0.152943	1.084238	0.48938	6.460162	0.303628	2.785053	1.23996	18.74545	1.414913	6.323894	4.583543	6.373881	5.9596	3.644924	6.592617	8.777431	0	0.110022	0.480738	0.138669	0.36661	0.848474	0.736955	0.60576	0.057589	0.267389	0.045072	Arctic	5.79E-07
Bacteria|Actinobacteria|Actinobacteria|Actinomycetales|Actinosynnemataceae	0	0	0	0	0	0.212936	0.049093	0	0	0.023506	0	0	0	0	0	0	0.21162	0.279856	0.886905	0	0.035839	0.106605	0.060094	0	0	0	0	0	0	0	0	0	0	0	0	0	0	0	0	0	0	0	0	0	0	0	0	0	0	0	0	0	0	0	0	0	0	0	0	0	0	0	0	0	0		
Bacteria|Actinobacteria|Actinobacteria|Actinomycetales|Dietziaceae	0	0	0	0	0	0	0	0	0	0	0	0	0	0	0	0	0.799454	0.759608	1.224773	0.411465	0.250873	0.724917	0.400629	0.138359	1.514121	0	0	0	0	0	0	0	0	0	0	0	0	0	0	0	0	0	0	0	0	0	0	0	0	0.112315	0	0	0	0.103293	0	0	0	0.046223	0	0.106059	0.088435	0	0	0	0	China oil refineries	3.24E-07
Bacteria|Actinobacteria|Actinobacteria|Actinomycetales|Dietziaceae|Dietzia	0	0	0	0	0	0	0	0	0	0	0	0	0	0	0	0	0.799454	0.759608	1.224773	0.411465	0.250873	0.724917	0.400629	0.138359	1.514121	0	0	0	0	0	0	0	0	0	0	0	0	0	0	0	0	0	0	0	0	0	0	0	0	0.112315	0	0	0	0.103293	0	0	0	0.046223	0	0.106059	0.088435	0	0	0	0	China oil refineries	3.24E-07
Bacteria|Actinobacteria|Actinobacteria|Actinomycetales|Frankiaceae	0	0	0	0	0	0	0	0	0	0	0	0	0	0	0	0	0	0.039979	0.422336	0	0	0	0	0	0.042059	0	0	0	0	0	0	0	0	0	0	0.047417	0	1.328639	0	0	0	0.298161	0	0	0	0.121705	0	0	0	0	0	0	0	0	0	0	0	0	0	0	0	0	0	0	0		
Bacteria|Actinobacteria|Actinobacteria|Actinomycetales|Geodermatophilaceae	0	0	0	0	0	0	0	0	0	0	0	0	0	0	0	0	0.376213	0.586364	0.534958	0.199498	0.549531	1.193981	0.614297	0.184478	0.196275	0	0	0	0	0	0	0	0	0	0	0.158056	0	0.560981	0	0	0	0.056793	0.030982	0	0.086009	0	0	0	0	0.037438	0	0	0.100907	0.137724	0	0	0	0	0	0	0	0	0	0	0	China oil refineries	3.07E-06
Bacteria|Actinobacteria|Actinobacteria|Actinomycetales|Geodermatophilaceae|Blastococcus	0	0	0	0	0	0	0	0	0	0	0	0	0	0	0	0	0	0	0.028156	0	0	0	0	0	0	0	0	0	0	0	0	0	0	0	0	0.158056	0	0.50193	0	0	0	0.056793	0	0	0.086009	0	0	0	0	0	0	0	0	0	0	0	0	0	0	0	0	0	0	0	0		
Bacteria|Actinobacteria|Actinobacteria|Actinomycetales|Geodermatophilaceae|Blastococcus|aggregatus	0	0	0	0	0	0	0	0	0	0	0	0	0	0	0	0	0	0	0.028156	0	0	0	0	0	0	0	0	0	0	0	0	0	0	0	0	0.158056	0	0.50193	0	0	0	0.056793	0	0	0.086009	0	0	0	0	0	0	0	0	0	0	0	0	0	0	0	0	0	0	0	0		
Bacteria|Actinobacteria|Actinobacteria|Actinomycetales|Intrasporangiaceae	0	0	0	0	1.676368	0.638809	0.392744	4.66304	0.040557	0.094024	0.460168	0.043132	2.334839	0.936058	0.502372	0.041574	1.739987	0.399794	2.111678	0.299247	0.179195	0.085284	0.160251	0.03459	0.084118	0.23403	0.20318	0.078953	0	0	0	0	0	0	0	0	0	7.17465	0	0.154157	0	0.596323	0	0.043731	0	1.298186	0	0.052699	0	0.056158	0	0	0	0	0	0	0	0	0	0	0	0	0	0	0	Taiga upper active layer	1.3E-05
Bacteria|Actinobacteria|Actinobacteria|Actinomycetales|Intrasporangiaceae|Terracoccus	0	0	0	0	0	0	0	0	0	0	0	0	0	0	0	0	0	0	0	0	0.035839	0	0	0	0	0	0	0	0	0	0	0	0	0	0	0	0	6.73177	0	0.051386	0	0.596323	0	0	0	0.283978	0	0	0	0	0	0	0	0	0	0	0	0	0	0	0	0	0	0	0		
Bacteria|Actinobacteria|Actinobacteria|Actinomycetales|Microbacteriaceae	4.695957	1.278145	1.955696	0.511958	25.3473	14.67842	22.01001	18.08117	13.01869	15.65498	13.62097	20.80394	33.17608	32.1068	9.79626	12.43071	2.586468	1.599175	2.618481	0.92268	1.099061	2.459031	1.442263	0.345897	1.514121	0.585075	0.772083	0.434241	1.508063	0	0.236619	0.095053	0.095472	0.172977	0.043315	0.853504	0	2.657278	0.041712	0.205543	0.043181	0.809295	0.046474	0.262387	0.344035	1.257617	0.322385	4.848319	3.101645	3.575363	3.76932	2.746408	5.280821	6.559095	0	0.110022	0.447583	0.092446	0.36661	0.530296	0.530608	0.60576	0.057589	0.053478	0.045072	Arctic	2.63E-07
Bacteria|Actinobacteria|Actinobacteria|Actinomycetales|Microbacteriaceae|Microbacterium	4.695957	1.278145	1.781856	0.469295	16.15832	14.43709	20.61904	16.08273	12.93758	15.46693	13.48292	19.19368	32.18415	30.93673	9.712531	11.4745	2.586468	1.599175	2.534014	0.897742	1.075168	2.430603	1.4022	0.345897	1.514121	0.585075	0.772083	0.434241	1.508063	0	0.236619	0.095053	0.095472	0.172977	0.043315	0.853504	0	2.657278	0.041712	0.205543	0.043181	0.809295	0.046474	0.262387	0.344035	1.257617	0.322385	4.848319	2.998257	3.481768	3.565573	2.746408	5.247185	6.559095	0	0.110022	0.397852	0.092446	0.36661	0.477267	0.530608	0.550691	0.057589	0.053478	0.045072	Arctic	2.46E-07
Bacteria|Actinobacteria|Actinobacteria|Actinomycetales|Microbacteriaceae|Salinibacterium	0	0	0.17384	0.042663	9.03376	0.212936	1.129138	1.998446	0.081113	0.188048	0.13805	1.552747	0.961404	1.170073	0.083729	0.956208	0	0	0	0	0	0	0	0	0	0	0	0	0	0	0	0	0	0	0	0	0	0	0	0	0	0	0	0	0	0	0	0	0	0	0	0	0	0	0	0	0	0	0	0	0	0	0	0	0	Arctic	1.56E-08
Bacteria|Actinobacteria|Actinobacteria|Actinomycetales|Microbacteriaceae|Salinibacterium|amurskyense	0	0	0	0	4.796275	0.127762	1.129138	1.998446	0.081113	0.188048	0.13805	1.552747	0.228906	1.170073	0	0.956208	0	0	0	0	0	0	0	0	0	0	0	0	0	0	0	0	0	0	0	0	0	0	0	0	0	0	0	0	0	0	0	0	0	0	0	0	0	0	0	0	0	0	0	0	0	0	0	0	0	Arctic	3.43E-08
Bacteria|Actinobacteria|Actinobacteria|Actinomycetales|Micrococcaceae	0	0	0	0	0.605355	0	6.382083	0.095164	0.12167	0.376096	0.460168	0.172527	5.905769	2.808175	0	0	0.728914	1.419268	1.18254	0.935148	0.659437	2.537209	3.064809	0.743679	0.988385	0	0	0.019738	0	0	0	0	0	0	0	0.094834	0.049745	17.75504	0	0.184989	0.194313	1.554698	0.079005	0.345476	0.236524	5.780983	0.29552	0	0.155082	0.393103	0	0	0.100907	0.025823	0	0	0	0	0	0.106059	0	0	0	0	0	Taiga upper active layer	1.45E-05
Bacteria|Actinobacteria|Actinobacteria|Actinomycetales|Micrococcaceae|Arthrobacter	0	0	0	0	0	0	0	0	0	0	0	0	0	0	0	0	0.540807	1.27934	0.823555	0.635901	0.483826	2.068145	2.584055	0.60532	0.672943	0	0	0.019738	0	0	0	0	0	0	0	0	0	0.017715	0	0.082217	0.086361	0.681512	0.055768	0.017492	0.086009	0.730229	0.214924	0	0	0	0	0	0	0	0	0	0	0	0	0	0	0	0	0	0	China oil refineries	1.42E-08
Bacteria|Actinobacteria|Actinobacteria|Actinomycetales|Micrococcaceae|Arthrobacter|psychrolactophilus	0	0	0	0	0	0	0	0	0	0	0	0	0	0	0	0	0.540807	1.27934	0.823555	0.635901	0.483826	2.068145	2.584055	0.60532	0.672943	0	0	0.019738	0	0	0	0	0	0	0	0	0	0	0	0	0	0	0	0	0	0	0	0	0	0	0	0	0	0	0	0	0	0	0	0	0	0	0	0	0	China oil refineries	9.82E-09
Bacteria|Actinobacteria|Actinobacteria|Actinomycetales|Micrococcaceae|Microbispora	0	0	0	0	0	0	0	0	0	0	0	0	0	0	0	0	0.094053	0.099948	0.147817	0.149624	0.089597	0.106605	0.120189	0.069179	0.084118	0	0	0	0	0	0	0	0	0	0	0	0	0.775039	0	0	0.02159	0.021297	0.023237	0.021866	0.021502	0.182557	0.026865	0	0	0	0	0	0	0.025823	0	0	0	0	0	0	0	0	0	0	0		
Bacteria|Actinobacteria|Actinobacteria|Actinomycetales|Micrococcaceae|Microbispora|rosea	0	0	0	0	0	0	0	0	0	0	0	0	0	0	0	0	0.094053	0.099948	0.147817	0.149624	0.089597	0.106605	0.120189	0.069179	0.084118	0	0	0	0	0	0	0	0	0	0	0	0	0.775039	0	0	0.02159	0.021297	0.023237	0.021866	0.021502	0.182557	0.026865	0	0	0	0	0	0	0.025823	0	0	0	0	0	0	0	0	0	0	0		
Bacteria|Actinobacteria|Actinobacteria|Actinomycetales|Micromonosporaceae	0	0	0.028973	0	0.310438	0.028392	0	0.063443	0.081113	0.626826	0.214745	0.69011	0.305208	0.187212	0.418644	0.027716	0.56432	0.906199	2.111678	0.174561	0.358389	0.397994	0.267086	0.04612	0.084118	0.07801	0	0.026318	0	0	0	0	0	0	0	0.126445	0	0	0	0	0	0	0	0.087462	0	0	0	0	0	0	0	0	0	0.068862	0	0	0	0	0	0	0	0	0	0	0	China oil refineries	2.11E-06
Bacteria|Actinobacteria|Actinobacteria|Actinomycetales|Micromonosporaceae|Solwaraspora	0	0	0.028973	0	0.310438	0.028392	0	0.063443	0.081113	0.626826	0.214745	0.69011	0.305208	0.187212	0.418644	0.027716	0.470267	0.799588	1.9709	0.124686	0.191141	0.312709	0.160251	0.04612	0.028039	0.07801	0	0.026318	0	0	0	0	0	0	0	0.031611	0	0	0	0	0	0	0	0.058308	0	0	0	0	0	0	0	0	0	0	0	0	0	0	0	0	0	0	0	0	0		
Bacteria|Actinobacteria|Actinobacteria|Actinomycetales|Mycobacteriaceae	0.071151	0	0.17384	0.170653	0	0.042587	0	0	0	0	0.046017	0	0	0	0	0.083149	3.621055	2.038948	9.882654	0.374059	0.215034	1.236623	0.240377	0.138359	0.630884	0.273035	0.568904	0.434241	0	0	0	0	0	0	0	1.138006	0.248727	1.815806	0	0	0.043181	0.170378	0.092947	0.218656	0	0.040568	0	0	0	0.056158	0	0	0	0	0	0	0	0	0	0	0	0	0	0	0	China oil refineries	2.83E-06
Bacteria|Actinobacteria|Actinobacteria|Actinomycetales|Mycobacteriaceae|Mycobacterium	0.071151	0	0.17384	0.170653	0	0.042587	0	0	0	0	0.046017	0	0	0	0	0.083149	3.621055	2.038948	9.882654	0.374059	0.215034	1.236623	0.240377	0.138359	0.630884	0.273035	0.568904	0.434241	0	0	0	0	0	0	0	1.138006	0.248727	1.815806	0	0	0.043181	0.170378	0.092947	0.218656	0	0.040568	0	0	0	0.056158	0	0	0	0	0	0	0	0	0	0	0	0	0	0	0	China oil refineries	2.83E-06
Bacteria|Actinobacteria|Actinobacteria|Actinomycetales|Mycobacteriaceae|Mycobacterium|vaccae	0	0	0	0	0	0	0	0	0	0	0	0	0	0	0	0	0.611347	0.439773	1.604875	0.149624	0.035839	0.170569	0.120189	0.03459	0.252354	0	0	0	0	0	0	0	0	0	0	0.047417	0	0	0	0	0	0	0	0.043731	0	0	0	0	0	0	0	0	0	0	0	0	0	0	0	0	0	0	0	0	0		
Bacteria|Actinobacteria|Actinobacteria|Actinomycetales|Nocardiaceae	0	0	0	0	0	0	1.080045	0.063443	0	0.031341	0.092034	0	0	2.527357	0.055819	0	0.56432	0.946179	0.689815	0.162092	0.143356	1.862041	1.175177	0.080709	3.813342	0	0	0	0	0	0	0	0	0	0	0.031611	0	3.0042	0	0.025693	0.107951	0.191675	0	0.021866	0.172018	1.054776	0.188058	0.070265	0	0	0	0.101719	0	0.215193	0	0	0	0	0	0	0.029478	0	0	0	0	China oil refineries	0.000112
Bacteria|Actinobacteria|Actinobacteria|Actinomycetales|Nocardiaceae|Nocardia	0	0	0	0	0	0	0	0	0	0	0	0	0	0	0	0	0.376213	0.119938	0.211168	0.037406	0	0.383779	0.801257	0.03459	1.219709	0	0	0	0	0	0	0	0	0	0	0	0	0	0	0	0	0	0	0	0	0	0	0	0	0	0	0	0	0	0	0	0	0	0	0	0	0	0	0	0		
Bacteria|Actinobacteria|Actinobacteria|Actinomycetales|Nocardiaceae|Rhodococcus	0	0	0	0	0	0	1.080045	0.063443	0	0.031341	0.092034	0	0	2.527357	0.055819	0	0.188107	0.82624	0.478647	0.124686	0.143356	1.478262	0.37392	0.04612	2.593634	0	0	0	0	0	0	0	0	0	0	0.031611	0	3.0042	0	0.025693	0.107951	0.191675	0	0.021866	0.172018	1.054776	0.188058	0.070265	0	0	0	0.101719	0	0.215193	0	0	0	0	0	0	0.029478	0	0	0	0	Taiga upper active layer	0.000167
Bacteria|Actinobacteria|Actinobacteria|Actinomycetales|Nocardiaceae|Rhodococcus|fascians	0	0	0	0	0	0	0	0.063443	0	0	0.030678	0	0	0	0	0	0.062702	0.666323	0.19709	0.074812	0.119463	1.108696	0.347212	0.02306	2.271182	0	0	0	0	0	0	0	0	0	0	0	0	0.029525	0	0	0	0	0	0	0	0	0	0.070265	0	0	0	0.101719	0	0	0	0	0	0	0	0	0.029478	0	0	0	0		
Bacteria|Actinobacteria|Actinobacteria|Actinomycetales|Nocardiaceae|Rhodococcus|globerulus	0	0	0	0	0	0	0	0	0	0	0	0	0	0	0	0	0	0	0	0	0	0	0	0	0.042059	0	0	0	0	0	0	0	0	0	0	0	0	0.11072	0	0.025693	0.107951	0.191675	0	0.021866	0.172018	1.054776	0.188058	0	0	0	0	0	0	0.07747	0	0	0	0	0	0	0	0	0	0	0		
Bacteria|Actinobacteria|Actinobacteria|Actinomycetales|Nocardioidaceae	0.189736	0.025821	0	0.028442	0.40357	1.504751	4.352908	3.96517	0.324453	2.726693	0.306779	0.258791	4.852803	6.864428	0.195367	0.36031	0.940534	1.119423	1.154384	0.473808	0.501745	1.50669	1.095052	0.368957	0.308432	0.130017	0.027091	0.046056	0	0	0	0	0	0	0	0.284501	0	8.90188	0	0.205543	0	0.227171	0.030982	0.816316	0.229357	4.760014	0.60895	0.035133	0.310165	0.430541	0	0.033906	0	0.826343	0	0	0.033154	0	0	0.106059	0	0	0	0	0	Taiga upper active layer	4.88E-06
Bacteria|Actinobacteria|Actinobacteria|Actinomycetales|Nocardioidaceae|Nocardioides	0	0	0	0	0	0.482656	3.207406	2.474266	0	1.065604	0	0.057509	0.427291	0.748847	0.02791	0.055432	0	0	0	0	0.023893	0	0	0	0	0	0	0	0	0	0	0	0	0	0	0	0	0	0	0	0	0	0	0	0	0	0	0	0	0	0	0	0	0	0	0	0	0	0	0	0	0	0	0	0		
Bacteria|Actinobacteria|Actinobacteria|Actinomycetales|Nocardioidaceae|Nocardioides|plantarum	0	0	0	0	0	0.482656	3.207406	2.474266	0	1.065604	0	0.057509	0.427291	0.748847	0.02791	0.055432	0	0	0	0	0.023893	0	0	0	0	0	0	0	0	0	0	0	0	0	0	0	0	0	0	0	0	0	0	0	0	0	0	0	0	0	0	0	0	0	0	0	0	0	0	0	0	0	0	0	0		
Bacteria|Actinobacteria|Actinobacteria|Actinomycetales|Nocardioidaceae|Pimelobacter	0	0	0	0	0	0.113566	0.556387	0	0	0.125365	0.030678	0	0.030521	0.031202	0	0	0	0	0.028156	0.024937	0	0	0	0	0	0.026003	0	0	0	0	0	0	0	0	0	0	0	0.147627	0	0.034257	0	0	0	0.058308	0.114678	0.054091	0	0	0	0	0	0	0	0	0	0	0	0	0	0	0	0	0	0	0		
Bacteria|Actinobacteria|Actinobacteria|Actinomycetales|Nocardioidaceae|Propionicimonas	0	0	0	0	0	0	0	0	0	0	0	0	0	0	0	0	0	0	0	0	0	0	0	0	0	0	0	0	0	0	0	0	0	0	0	0	0	0.044288	0	0	0	0	0	0	0	0	0.537309	0	0	0.393103	0	0	0	0	0	0	0	0	0	0	0	0	0	0	0		
Bacteria|Actinobacteria|Actinobacteria|Actinomycetales|Propionibacteriaceae	0	0	0	0	0.046566	0	0	0	0.081113	0.047012	0	0	0	0	0	0	0	0	0	0	0	0	0	0	0	0	0	0	0	0	0	0	0	0	0	0	0	0.132864	0	0	0	0	0	0.043731	0.043004	0.040568	0	1.001283	0.672023	1.403939	1.375292	0.762891	0.706352	0.154939	0	0	0	0	0	0	0	0	0	0.160434	0	Oil sands core	1.13E-05
Bacteria|Actinobacteria|Actinobacteria|Actinomycetales|Propionibacteriaceae|Propionibacterium	0	0	0	0	0.046566	0	0	0	0.081113	0.047012	0	0	0	0	0	0	0	0	0	0	0	0	0	0	0	0	0	0	0	0	0	0	0	0	0	0	0	0.132864	0	0	0	0	0	0.043731	0.043004	0.040568	0	1.001283	0.672023	1.403939	1.375292	0.661172	0.706352	0.154939	0	0	0	0	0	0	0	0	0	0.160434	0	Oil sands core	1.13E-05
Bacteria|Actinobacteria|Actinobacteria|Actinomycetales|Pseudonocardiaceae	0	0	0	0	0.186263	0	0	0	0	0.062683	0.490846	0	0.305208	0.093606	0.02791	0.055432	0.21162	0.193234	0.246362	0.074812	0.143356	0.440636	0.026709	0.04612	0.077108	0	0	0.026318	0	0	0	0	0	0	0	0.25289	0	5.698384	0	0	0.028787	0	0.023237	0.029154	0	0.027046	0	0	0	0.028079	0	0	0	0.309878	0	0	0	0	0	0	0	0	0	0	0		
Bacteria|Actinobacteria|Actinobacteria|Actinomycetales|Pseudonocardiaceae|Pseudonocardia	0	0	0	0	0.186263	0	0	0	0	0	0.460168	0	0.305208	0.093606	0.02791	0.055432	0.094053	0.026653	0.19709	0.024937	0.047785	0.369565	0	0.02306	0.056079	0	0	0.026318	0	0	0	0	0	0	0	0.189668	0	5.698384	0	0	0.028787	0	0	0.029154	0	0.027046	0	0	0	0	0	0	0	0	0	0	0	0	0	0	0	0	0	0	0		
Bacteria|Actinobacteria|Actinobacteria|Actinomycetales|Sporichthyaceae	0	0	0	0	0	0	0	0	0	0.188048	0	0.057509	0.091562	0	0	0	0.047027	0.199897	0.112623	0.074812	0.059732	0.341137	1.12176	0.126829	0.532746	0	0	0	0	0	0	0	0	0	0	0.094834	0	4.163068	0	0.274057	0.028787	2.47048	0	0.437312	0.043004	4.070353	0	0	0	0	0	0	0	0	0	0	0	0	0	0	0	0	0	0	0	Taiga permafrost layer	2.44E-05
Bacteria|Actinobacteria|Actinobacteria|Actinomycetales|Streptomycetaceae	0.040658	0	0.012417	0.048758	0	0.012168	0.504956	0	0.011588	0.013432	0.013148	0.024647	0.300848	0.013372	0	0	0.174671	0.159918	0.313735	0.032062	0.040959	0.085284	0.080126	0	0.060084	0	0	0	0.013057	0	0	0	0	0	0	0.013548	0	0	0	0	0	0	0	0.012495	0	0.023182	0	0	0	0	0	0	0	0.014756	0	0	0	0	0	0	0	0	0	0	0		
Bacteria|Actinobacteria|Actinobacteria|Actinomycetales|Streptomycetaceae|Streptomyces	0.040658	0	0.012417	0.048758	0	0.012168	0.504956	0	0.011588	0.013432	0.013148	0.024647	0.300848	0.013372	0	0	0.174671	0.159918	0.313735	0.032062	0.040959	0.085284	0.080126	0	0.060084	0	0	0	0.013057	0	0	0	0	0	0	0.013548	0	0	0	0	0	0	0	0.012495	0	0.023182	0	0	0	0	0	0	0	0.014756	0	0	0	0	0	0	0	0	0	0	0		
Bacteria|Actinobacteria|Actinobacteria|Actinomycetales|Thermomonosporaceae	0	0	0	0	0	0	0	0	0	0	0	0	0	0	0	0	0.225728	0.111942	0.810884	0.044887	0.014336	0.017057	0.096151	0	0	0	0	0	0	0	0	0	0	0	0	0.018967	0	0	0	0	0	0	0	0	0	0	0	0	0	0	0	0	0	0	0	0	0	0	0	0	0	0	0	0	0		
Bacteria|Actinobacteria|Actinobacteria|Actinomycetales|Williamsiaceae	0	0	0	0	0	0	0	0	0	0	0	0	0	0	0	0	0.56432	0.039979	0.253401	0.037406	0.071678	0.170569	0	0	0.252354	0	0	0	0	0	0	0	0	0	0	0	0	0	0	0	0	0	0	0	0	0	0	0	0	0	0	0	0	0	0	0	0	0	0	0	0	0	0	0.053478	0		
Bacteria|Actinobacteria|Actinobacteria|Actinomycetales|Williamsiaceae|Williamsia	0	0	0	0	0	0	0	0	0	0	0	0	0	0	0	0	0.56432	0.039979	0.253401	0.037406	0.071678	0.170569	0	0	0.252354	0	0	0	0	0	0	0	0	0	0	0	0	0	0	0	0	0	0	0	0	0	0	0	0	0	0	0	0	0	0	0	0	0	0	0	0	0	0	0.053478	0		

Table S2. I

Bacterial clades	Habitat																																																																Differentially abundant class (one class test, non-strict)	p-value
	India oil refineries				Arctic												China oil refineries									Mangrove			Marine sediments							Taiga upper active layer				Taiga bottom active layer				Taiga permafrost layer				Oil sands core							Oil sands tailings pond upper				Oil sands tailings pond median			Oil sands tailings pond deep					
	I1	I2	I3	I4	A1	A10	A11	A12	A2	A3	A4	A5	A6	A7	A8	A9	C1	C2	C3	C4	C5	C6	C7	C8	C9	M1	M2	M3	DWH1	DWH2	DWH3	DWH4	DWH5	DWH6	DWH7	Tu1	Tu2	Tu3	Tu4	Tb1	Tb2	Tb3	Tb4	Tp1	Tp2	Tp3	Tp4	OSC1	OSC12	OSC3	OSC4	OSC5	OSC7	OSC9	OSTPu1	OSTPu2	OSTPu4	OSTPu3	OSTPm2	OSTPm6	OSTPm4	OSTPd1	OSTPd2	OSTPd3	OSTPd4		
Bacteria|Actinobacteria|MB-A2-108	0	0	0	0	0.139697	0.170349	0.294558	0	0.405567	0.188048	0.414151	0.086264	0.320468	0.140409	0.125593	0	0.047027	0.479753	0.464569	0.561089	0.501745	0.426422	0.721132	0.069179	0.504707	0	0	0	0	0	0	0	0	0	0	0.284501	0	2.302974	0	0.308314	0.172722	0.170378	0	0.524774	0.086009	1.338754	0	0	0	0	0	0	0	0	0	0	0	0	0	0	0	0	0	0	0	Taiga upper active layer	7.35E-06
Bacteria|Actinobacteria|MB-A2-108|0319-7L14	0	0	0	0	0.139697	0.170349	0.294558	0	0.405567	0.188048	0.414151	0.086264	0.320468	0.140409	0.125593	0	0.047027	0.479753	0.464569	0.561089	0.501745	0.426422	0.721132	0.069179	0.504707	0	0	0	0	0	0	0	0	0	0	0.284501	0	2.302974	0	0.308314	0.172722	0.170378	0	0.524774	0.086009	1.338754	0	0	0	0	0	0	0	0	0	0	0	0	0	0	0	0	0	0	0	Taiga upper active layer	7.35E-06
Bacteria|Actinobacteria|OPB41	0.071151	0	0.2173	0	0	0.170349	0	0	0	0	0	0	0	0	0	0	0	0	0	0	0.035839	0	0	0	0	0	0	0	0	0	0	0	0	0	0	0.094834	0	0	0	0.051386	0.086361	0.042594	0	0.043731	0	0	0	0	0	0.280788	0	0.050859	0	0	0.149365	0.055011	2.038991	2.634713	0.209491	1.962096	0.707477	0	0.057589	0.374345	1.081735	Oil sands tailings pond upper	0.000172
Bacteria|Actinobacteria|Thermoleophilia	0	0	0	0	0.279395	0.042587	0.049093	0	2.027833	1.69243	1.334487	0	2.151714	0	0.627965	0	1.787014	1.479237	2.618481	1.00996	1.612753	1.364549	2.243521	0.588025	1.135591	0.429055	0.121908	0.6711	0.182795	0	0.047324	0	0	0	0	0.379335	0.099491	5.225979	0.083424	0.3597	0.215903	3.961286	0	0.481043	0.043004	3.813421	0	0	0	0	0	0	0	0	0	0	0	0	0	0	0	0	0	0	0	China oil refineries	2.12E-05
Bacteria|Actinobacteria|Thermoleophilia|Gaiellales	0	0	0	0	0.186263	0	0	0	0	0	0.230084	0	0.869842	0	0	0	0.56432	0.559711	0.506803	0.448871	0.645101	0.511706	0.761194	0.138359	0.336471	0.23403	0.040636	0.394765	0.091398	0	0	0	0	0	0	0.142251	0.099491	3.941628	0.083424	0.3597	0.215903	3.705719	0	0.218656	0.043004	3.164328	0	0	0	0	0	0	0	0	0	0	0	0	0	0	0	0	0	0	0	Taiga bottom active layer	8.21E-06
Bacteria|Actinobacteria|Thermoleophilia|Gaiellales|Gaiellaceae	0	0	0	0	0.186263	0	0	0	0	0	0.230084	0	0.869842	0	0	0	0.56432	0.559711	0.506803	0.448871	0.645101	0.511706	0.721132	0.138359	0.336471	0.23403	0.040636	0.394765	0.091398	0	0	0	0	0	0	0.142251	0.099491	3.941628	0.083424	0.3597	0.215903	3.705719	0	0.218656	0.043004	3.164328	0	0	0	0	0	0	0	0	0	0	0	0	0	0	0	0	0	0	0	Taiga bottom active layer	8.21E-06
Bacteria|Actinobacteria|Thermoleophilia|Solirubrobacterales	0	0	0	0	0.093132	0.042587	0.049093	0	2.027833	1.69243	1.104403	0	1.281872	0	0.627965	0	1.222694	0.919526	2.111678	0.561089	0.967652	0.852843	1.482326	0.449666	0.79912	0.195025	0.081272	0.276335	0.091398	0	0.047324	0	0	0	0	0.237085	0	1.284351	0	0	0	0.255567	0	0.262387	0	0.649093	0	0	0	0	0	0	0	0	0	0	0	0	0	0	0	0	0	0	0	China oil refineries	7.05E-05
Bacteria|Actinobacteria|Thermoleophilia|Solirubrobacterales|Patulibacteraceae	0	0	0	0	0	0	0	0	0	0	0	0	0	0	0	0	0.188107	0	0	0.037406	0.071678	0.085284	0.040063	0.03459	0	0	0	0	0	0	0	0	0	0	0	0.047417	0	0.974335	0	0	0	0	0	0	0	0.24341	0	0	0	0	0	0	0	0	0	0	0	0	0	0	0	0	0	0	0		
Bacteria|Actinobacteria|Thermoleophilia|Solirubrobacterales|Solirubrobacteraceae	0	0	0	0	0.093132	0.042587	0.049093	0	2.027833	1.69243	1.104403	0	1.281872	0	0.627965	0	0.56432	0.759608	1.689343	0.374059	0.716779	0.511706	0.961509	0.276718	0.588825	0.195025	0.081272	0.236859	0.045699	0	0.047324	0	0	0	0	0.189668	0	0.310016	0	0	0	0.255567	0	0.262387	0	0.405683	0	0	0	0	0	0	0	0	0	0	0	0	0	0	0	0	0	0	0	China oil refineries	6.01E-05
Bacteria|Actinobacteria|Thermoleophilia|Solirubrobacterales|Solirubrobacteraceae|Solirubrobacter	0	0	0	0	0.093132	0.042587	0	0	0	0	0	0	1.281872	0	0.544237	0	0	0	0	0.037406	0	0	0	0	0	0	0	0	0	0	0	0	0	0	0	0	0	0	0	0	0	0	0	0	0	0	0	0	0	0	0	0	0	0	0	0	0	0	0	0	0	0	0	0	0		
Bacteria|AD3	0	0	0	0	0	0	0	0	0	0	0	0	0	0	0	0	0.094053	0	0	0	0	0	0	0.03459	0	0	0	0	0	0	0	0	0	0	0	0	0	4.960252	0	0	0.086361	9.498568	0	0.437312	0	15.33482	0	0	0	0	0	0	0	0	0	0	0	0	0	0	0	0	0	0	0		
Bacteria|AD3|ABS-6	0	0	0	0	0	0	0	0	0	0	0	0	0	0	0	0	0	0	0	0	0	0	0	0	0	0	0	0	0	0	0	0	0	0	0	0	0	4.960252	0	0	0.086361	9.370785	0	0.437312	0	15.05084	0	0	0	0	0	0	0	0	0	0	0	0	0	0	0	0	0	0	0		
Bacteria|Armatimonadetes	0	0.038732	0.04346	0	0	0.042587	0	0	0	0	0	0	0	0.046803	0	0.083149	0.940534	0.199897	0.380102	0.299247	0.609262	0.127926	0.961509	0.484256	0.210295	0	0	0	0	0	0	0	0	0	0	0.474169	1.243637	0	0	0.565243	0.172722	0	0	0.174925	0	0	0	0	0	0.168473	0	0	0	0	0.049788	0	0	0.046223	0	0	0.044217	0	0	0	0		
Bacteria|Armatimonadetes|0319-6E2	0	0	0	0	0	0	0	0	0	0	0	0	0	0	0	0	0.376213	0.159918	0.253401	0.224436	0.322551	0.085284	0.761194	0.242128	0.084118	0	0	0	0	0	0	0	0	0	0	0.047417	0	0	0	0.411086	0.043181	0	0	0.043731	0	0	0	0	0	0	0	0	0	0	0	0	0	0	0	0	0	0	0	0	0		
Bacteria|Armatimonadetes|Armatimonadia	0	0	0	0	0	0	0	0	0	0	0	0	0	0	0	0	0.14108	0	0.042234	0	0.035839	0	0.040063	0.172948	0.042059	0	0	0	0	0	0	0	0	0	0	0.237085	1.193891	0	0	0	0.129542	0	0	0.087462	0	0	0	0	0	0	0	0	0	0	0	0	0	0	0	0	0	0	0	0	0		
Bacteria|Armatimonadetes|Armatimonadia|FW68	0	0	0	0	0	0	0	0	0	0	0	0	0	0	0	0	0	0	0	0	0	0	0	0	0	0	0	0	0	0	0	0	0	0	0	0.142251	1.144146	0	0	0	0.086361	0	0	0.087462	0	0	0	0	0	0	0	0	0	0	0	0	0	0	0	0	0	0	0	0	0		
Bacteria|Bacteroidetes	2.395412	2.168973	17.69687	5.404	4.540163	4.712992	10.25224	11.82414	6.225449	7.913677	4.670706	6.239743	4.138617	3.307406	4.772537	6.263858	1.515827	4.10988	1.244482	6.032329	2.745264	0.884114	4.836923	5.129652	4.039059	1.911245	0.521495	3.12522	6.420691	14.87737	10.22193	9.885518	9.483575	11.32276	11.10308	1.085847	0.663273	1.439359	0.278078	0.77935	0.532561	0.099387	0.464737	0.893574	0.480216	0.081137	1.146259	3.618673	5.203872	2.203248	1.25644	0.81375	1.177253	2.23629	1.634719	0.623458	3.224258	1.263429	2.025084	1.405285	3.706885	1.916405	1.971474	3.814753	4.149654	Marine sediments	1.61E-06
Bacteria|Bacteroidetes|Bacteroidia	0.071151	1.575088	3.158086	0.255979	1.024447	0.085175	0	0	0	0	0	0	0	0	0.02791	0	0.062702	0.079959	0.112623	0.024937	0.023893	0	0.080126	0	0.084118	0.31204	0.054181	0.289494	0.060932	0.090394	0.347041	0.665371	0.350065	0	0.375397	0.126445	0	0.044288	0.111231	0	0.115148	0	0	0.221571	0	0.027046	0	0.105398	0	0.46798	0.033958	0.101719	0	0	1.526844	0.586784	1.98926	0.893645	1.920338	0.883827	2.918343	1.835637	1.842858	0.677386	2.914674	Oil sands tailings pond median	7.09E-05
Bacteria|Bacteroidetes|Bacteroidia|Bacteroidales	0.071151	1.575088	3.158086	0.255979	1.024447	0.085175	0	0	0	0	0	0	0	0	0.02791	0	0.062702	0.079959	0.112623	0.024937	0.023893	0	0.080126	0	0.084118	0.31204	0.054181	0.289494	0.060932	0.090394	0.347041	0.665371	0.350065	0	0.375397	0.126445	0	0.044288	0.111231	0	0.115148	0	0	0.221571	0	0.027046	0	0.105398	0	0.46798	0.033958	0.101719	0	0	1.526844	0.586784	1.98926	0.893645	1.920338	0.883827	2.918343	1.835637	1.842858	0.677386	2.914674	Oil sands tailings pond median	7.09E-05
Bacteria|Bacteroidetes|Bacteroidia|Bacteroidales|Porphyromonadaceae	0.023717	1.213592	1.187904	0	0.962359	0	0	0	0	0	0	0	0	0	0	0	0	0	0	0	0	0	0	0	0	0	0	0.157906	0	0	0	0	0	0	0	0.063223	0	0.029525	0	0	0.057574	0	0	0.058308	0	0	0	0	0	0.037438	0	0	0	0	0.099577	0	0.033154	0	0.034915	0	0.117913	0	0	0	0.030048		
Bacteria|Bacteroidetes|Bacteroidia|Bacteroidales|Porphyromonadaceae|Dysgonomonas	0.023717	0.903739	1.072011	0	0	0	0	0	0	0	0	0	0	0	0	0	0	0	0	0	0	0	0	0	0	0	0	0	0	0	0	0	0	0	0	0	0	0	0	0	0	0	0	0	0	0	0	0	0	0	0	0	0	0	0.033192	0	0	0	0	0	0.029478	0	0	0	0		
Bacteria|Bacteroidetes|Bacteroidia|Bacteroidales|Porphyromonadaceae|Paludibacter	0	0.309853	0.115893	0	0.962359	0	0	0	0	0	0	0	0	0	0	0	0	0	0	0	0	0	0	0	0	0	0	0	0	0	0	0	0	0	0	0.063223	0	0	0	0	0.057574	0	0	0.058308	0	0	0	0	0	0.037438	0	0	0	0	0.066385	0	0.033154	0	0.034915	0	0.088435	0	0	0	0		
Bacteria|Bacteroidetes|Bacteroidia|Bacteroidales|Rikenellaceae	0	0	0.434599	0	0	0	0	0	0	0	0	0	0	0	0	0	0.031351	0.079959	0.056311	0	0	0	0.026709	0	0.056079	0	0	0	0	0	0	0	0	0	0	0	0	0	0	0	0	0	0	0	0	0	0	0	0	0	0	0	0	0	0.132769	0	0.132617	0.061631	0	0	0.530608	0	0	0	1.862987		
Bacteria|Bacteroidetes|Bacteroidia|Bacteroidales|Rikenellaceae|Blvii28	0	0	0.434599	0	0	0	0	0	0	0	0	0	0	0	0	0	0.031351	0.079959	0.056311	0	0	0	0.026709	0	0.056079	0	0	0	0	0	0	0	0	0	0	0	0	0	0	0	0	0	0	0	0	0	0	0	0	0	0	0	0	0	0.132769	0	0.132617	0.061631	0	0	0.530608	0	0	0	1.862987		
Bacteria|Bacteroidetes|Cytophagia	0	0	0	0	0.069849	0	0.270011	0.198258	0.047316	0.047012	0.069025	0	0	0	0	0	0.133242	1.412605	0.070389	2.587243	0.238926	0.397994	1.095052	1.804429	2.264172	0.039005	0	0.078953	0.068548	0.316379	0.094647	0.031684	0.143208	0.043244	0.895177	0	0	0	0	0.128464	0.02159	0	0	0	0.172018	0	0	0	0	0.028079	0	0	0	0.232409	0.074683	0	0	0	0	0.026515	0.022109	0	0.028795	0	0.045072	China oil refineries	0.000215
Bacteria|Bacteroidetes|Cytophagia|Cytophagales	0	0	0	0	0.069849	0	0.270011	0.198258	0.047316	0.047012	0.069025	0	0	0	0	0	0.133242	1.412605	0.070389	2.587243	0.238926	0.397994	1.095052	1.804429	2.264172	0.039005	0	0.078953	0.068548	0.316379	0.094647	0.031684	0.143208	0.043244	0.895177	0	0	0	0	0.128464	0.02159	0	0	0	0.172018	0	0	0	0	0.028079	0	0	0	0.232409	0.074683	0	0	0	0	0.026515	0.022109	0	0.028795	0	0.045072	China oil refineries	0.000215
Bacteria|Bacteroidetes|Cytophagia|Cytophagales|Cytophagaceae	0	0	0	0	0.069849	0	0.245465	0.071373	0.027038	0.047012	0.069025	0	0	0	0	0	0.086216	1.172728	0.070389	2.25059	0.203087	0.270067	1.014926	1.423943	1.54917	0	0	0	0.022849	0	0	0.031684	0	0	0	0	0	0	0	0.128464	0.02159	0	0	0	0.172018	0	0	0	0	0	0	0	0	0.232409	0	0	0	0	0	0	0	0	0	0	0	China oil refineries	7.22E-05
Bacteria|Bacteroidetes|Cytophagia|Cytophagales|Cytophagaceae|Adhaeribacter	0	0	0	0	0	0	0	0	0	0	0	0	0	0	0	0	0.062702	0.319835	0.028156	0.598495	0.095571	0.056856	0.694423	0.507316	0.252354	0	0	0	0	0	0	0	0	0	0	0	0	0	0	0	0	0	0	0	0	0	0	0	0	0	0	0	0	0	0	0	0	0	0	0	0	0	0	0	0		
Bacteria|Bacteroidetes|Cytophagia|Cytophagales|Cytophagaceae|Pontibacter	0	0	0	0	0	0	0	0	0	0	0	0	0	0	0	0	0	0.772935	0	1.595986	0.071678	0.170569	0.240377	0.830153	1.149611	0	0	0	0	0	0	0	0	0	0	0	0	0	0	0	0	0	0	0	0	0	0	0	0	0	0	0	0	0	0	0	0	0	0	0	0	0	0	0	0		
Bacteria|Bacteroidetes|Cytophagia|Cytophagales|Flammeovirgaceae	0	0	0	0	0	0	0	0.031721	0	0	0	0	0	0	0	0	0.047027	0.159918	0	0.18703	0.035839	0.085284	0.040063	0.276718	0.630884	0.039005	0	0.078953	0.045699	0.316379	0.094647	0	0.143208	0.043244	0.851862	0	0	0	0	0	0	0	0	0	0	0	0	0	0	0	0	0	0	0	0	0	0	0	0	0	0	0	0	0	0		
Bacteria|Bacteroidetes|Flavobacteriia	0	0	5.875779	0.085326	0.186263	0.397481	5.302039	4.631318	0.216302	2.758034	1.135081	0	0.079354	1.653703	0.586101	0.027716	0.614482	0.618348	0.681368	1.687007	0.105128	0.017057	0.096151	0.21215	0.218706	0.611078	0.196407	1.625115	4.295693	11.81903	6.940814	6.416081	5.967014	10.21282	7.233605	0.042675	0	0.420736	0	0	0.02159	0.042594	0	0.380461	0.021502	0	1.074618	0.421593	4.032139	0.471723	0.407494	0	0.908167	0.86766	0.033192	0	1.102381	0.277338	0.034915	0.070706	0.648521	0.044055	0.023036	1.105209	1.099764	Marine sediments	0.002332
Bacteria|Bacteroidetes|Flavobacteriia|Flavobacteriales	0	0	5.875779	0.085326	0.186263	0.397481	5.302039	4.631318	0.216302	2.758034	1.135081	0	0.079354	1.653703	0.586101	0.027716	0.614482	0.618348	0.681368	1.687007	0.105128	0.017057	0.096151	0.21215	0.218706	0.611078	0.196407	1.625115	4.295693	11.81903	6.940814	6.416081	5.967014	10.21282	7.233605	0.042675	0	0.420736	0	0	0.02159	0.042594	0	0.380461	0.021502	0	1.074618	0.421593	4.032139	0.471723	0.407494	0	0.908167	0.86766	0.033192	0	1.102381	0.277338	0.034915	0.070706	0.648521	0.044055	0.023036	1.105209	1.099764	Marine sediments	0.002332
Bacteria|Bacteroidetes|Flavobacteriia|Flavobacteriales|Flavobacteriaceae	0	0	0.052152	0	0.186263	0.227132	5.105667	3.96517	0.135189	2.695351	1.043048	0	0.079354	1.27928	0.586101	0.027716	0.018811	0.45843	0.14641	1.238136	0.081235	0.017057	0.096151	0.18909	0.218706	0.611078	0.196407	1.361938	3.564512	7.118537	2.019146	3.501121	2.816431	4.706403	2.728845	0.042675	0	0.420736	0	0	0.02159	0.042594	0	0.292999	0.021502	0	0	0	0	0.134778	0	0	0	0.041317	0.033192	0	1.102381	0.277338	0.034915	0.070706	0.029478	0.044055	0.023036	0.035652	0.018029	Marine sediments	7.32E-05
Bacteria|Bacteroidetes|Flavobacteriia|Flavobacteriales|Flavobacteriaceae|Lutimonas	0	0	0	0	0	0	0	0	0	0	0	0	0	0	0	0	0	0	0	0	0	0	0	0	0	0	0	0.236859	0	1.386043	0.599434	0.570318	0.286417	1.700936	0.17326	0	0	0	0	0	0	0	0	0	0	0	0	0	0	0	0	0	0	0	0	0	0	0	0	0	0	0	0	0	0		
Bacteria|Bacteroidetes|Flavobacteriia|Flavobacteriales|Flavobacteriaceae|Olleya	0	0	0	0	0	0	0	0	0	0	0	0	0	0	0	0	0	0	0	0	0	0	0	0	0	0	0	0	0.182795	0.632759	0.37859	0.126737	0.254593	0.951371	0.375397	0	0	0	0	0	0	0	0	0	0	0	0	0	0	0	0	0	0	0	0	0	0	0	0	0	0	0	0	0	0		
Bacteria|Bacteroidetes|Flavobacteriia|Flavobacteriales|Flavobacteriaceae|Polaribacter	0	0	0	0	0	0	0	0	0	0	0	0	0	0	0	0	0	0	0	0	0	0	0	0	0	0	0	0	0.091398	1.416175	0.504786	0.602003	0.541009	0.461271	0.288767	0	0	0	0	0	0	0	0	0	0	0	0	0	0	0	0	0	0	0	0	0	0	0	0	0	0	0	0	0	0		
Bacteria|Bacteroidetes|Flavobacteriia|Flavobacteriales|Weeksellaceae	0	0	5.823627	0.085326	0	0.170349	0.196372	0.570984	0.081113	0	0.092034	0	0	0.374423	0	0	0.56432	0.159918	0.506803	0.448871	0	0	0	0	0	0	0	0.236859	0.639784	4.429312	4.82702	2.756539	3.055111	5.448761	4.3315	0	0	0	0	0	0	0	0	0.087462	0	0	1.074618	0.421593	4.032139	0.336945	0.407494	0	0.908167	0.826343	0	0	0	0	0	0	0.619043	0	0	1.069557	1.081735	Marine sediments	0.001047
Bacteria|Bacteroidetes|Flavobacteriia|Flavobacteriales|Weeksellaceae|Chryseobacterium	0	0	5.823627	0.085326	0	0.170349	0.196372	0.570984	0.081113	0	0.092034	0	0	0.374423	0	0	0.56432	0.159918	0.506803	0.448871	0	0	0	0	0	0	0	0.236859	0.639784	4.429312	4.82702	2.756539	3.055111	5.448761	4.3315	0	0	0	0	0	0	0	0	0.087462	0	0	0.967156	0.421593	4.032139	0.336945	0.407494	0	0.908167	0.826343	0	0	0	0	0	0	0.619043	0	0	1.069557	1.081735	Marine sediments	0.001035
Bacteria|Bacteroidetes|Rhodothermi	0	0	0	0	0	0	0	0.047582	0.040557	0	0	0	0	0	0	0	0.047027	0	0.042234	0.18703	0.107517	0.042642	0.040063	0	0.126177	0.663085	0.081272	0.631623	0.045699	0	0	0.047527	0	0	0	0	0	0	0	0	0	0	0	0	0	0	0	0	0	0	0	0	0	0	0	0	0	0	0	0	0	0	0	0	0		
Bacteria|Bacteroidetes|Rhodothermi|Rhodothermales	0	0	0	0	0	0	0	0.047582	0.040557	0	0	0	0	0	0	0	0.047027	0	0.042234	0.18703	0.107517	0.042642	0.040063	0	0.126177	0.663085	0.081272	0.631623	0.045699	0	0	0.047527	0	0	0	0	0	0	0	0	0	0	0	0	0	0	0	0	0	0	0	0	0	0	0	0	0	0	0	0	0	0	0	0	0		
Bacteria|Bacteroidetes|Rhodothermi|Rhodothermales|Rhodothermaceae	0	0	0	0	0	0	0	0.047582	0.040557	0	0	0	0	0	0	0	0	0	0	0.18703	0.035839	0	0	0	0.084118	0.54607	0.081272	0.434241	0.045699	0	0	0.047527	0	0	0	0	0	0	0	0	0	0	0	0	0	0	0	0	0	0	0	0	0	0	0	0	0	0	0	0	0	0	0	0	0		
Bacteria|Bacteroidetes|Rhodothermi|Rhodothermales|Rhodothermaceae|Rubricoccus	0	0	0	0	0	0	0	0.047582	0.040557	0	0	0	0	0	0	0	0	0	0	0.18703	0.035839	0	0	0	0.084118	0.54607	0.081272	0.434241	0.045699	0	0	0.047527	0	0	0	0	0	0	0	0	0	0	0	0	0	0	0	0	0	0	0	0	0	0	0	0	0	0	0	0	0	0	0	0	0		
Bacteria|Bacteroidetes|Saprospirae	2.300545	0.568064	8.344302	5.034252	2.328289	1.817057	0.916402	1.776396	1.892645	1.661089	1.227115	1.782784	1.648122	0.624039	2.037399	1.330377	0.250809	0.719629	0.140779	0.473808	1.481343	0.170569	1.175177	1.637246	0.644904	0.130017	0.054181	0.289494	0.243727	0.512233	0.189295	0.158422	0.254593	0.432441	0.635287	0.853504	0	0.50193	0.139039	0.4796	0.374232	0	0	0.204079	0.172018	0.027046	0.071641	3.091681	1.171733	0.973397	0.814988	0.678125	0.235451	0.964066	0	0.036674	0.099463	0.061631	0	0.424237	0.117913	0.036713	0.038393	2.032158	0.090145	India oil refineries	1.69E-05
Bacteria|Bacteroidetes|Saprospirae|Saprospirales	2.300545	0.568064	8.344302	5.034252	2.328289	1.817057	0.916402	1.776396	1.892645	1.661089	1.227115	1.782784	1.648122	0.624039	2.037399	1.330377	0.250809	0.719629	0.140779	0.473808	1.481343	0.170569	1.175177	1.637246	0.644904	0.130017	0.054181	0.289494	0.243727	0.512233	0.189295	0.158422	0.254593	0.432441	0.635287	0.853504	0	0.50193	0.139039	0.4796	0.374232	0	0	0.204079	0.172018	0.027046	0.071641	3.091681	1.171733	0.973397	0.814988	0.678125	0.235451	0.964066	0	0.036674	0.099463	0.061631	0	0.424237	0.117913	0.036713	0.038393	2.032158	0.090145	India oil refineries	1.69E-05
Bacteria|Bacteroidetes|Saprospirae|Saprospirales|Chitinophagaceae	2.300545	0.568064	8.286355	5.00581	2.266201	1.618317	0.687301	1.332297	1.838569	1.598406	1.104403	1.782784	1.617601	0.499231	1.98158	1.330377	0.250809	0.63967	0.140779	0.324185	1.36188	0.170569	1.175177	1.614186	0.644904	0.052007	0	0	0	0	0	0	0.031824	0	0	0.853504	0	0.50193	0.139039	0.411086	0.374232	0	0	0.174925	0.172018	0.027046	0.071641	3.091681	1.171733	0.973397	0.814988	0.678125	0.235451	0.964066	0	0.036674	0.099463	0.061631	0	0.424237	0.117913	0.036713	0.038393	2.032158	0.090145	India oil refineries	2.63E-06
Bacteria|Bacteroidetes|Saprospirae|Saprospirales|Chitinophagaceae|Flavisolibacter	0	0	0	0	0	0	0	0	0	0	0	0	0	0	0	0	0.156756	0.213223	0.028156	0.149624	0.549531	0	0.614297	0.899332	0.420589	0	0	0	0	0	0	0	0	0	0	0	0	0	0	0	0.287871	0	0	0	0.114678	0	0	0	0	0	0	0	0	0	0	0	0	0	0	0	0	0	0	0	0		
Bacteria|Bacteroidetes|Saprospirae|Saprospirales|Chitinophagaceae|Sediminibacterium	0	0	0.028973	0	0	0	0	0	0	0	0	0	0	0	0	0	0	0	0	0	0.023893	0	0	0	0	0.052007	0	0	0	0	0	0	0	0	0	0.094834	0	0	0	0.068514	0	0	0	0	0	0	0	2.529558	0.861568	0.67389	0.679157	0.678125	0.235451	0.585326	0	0	0.099463	0.061631	0	0.424237	0.058956	0	0	1.639987	0.090145	Oil sands core	6.24E-05
Bacteria|Bacteroidetes|Saprospirae|Saprospirales|Saprospiraceae	0	0	0	0	0	0.170349	0.098186	0.412378	0.054076	0	0.061356	0	0	0.093606	0.02791	0	0	0.079959	0	0.149624	0.119463	0	0	0.02306	0	0.07801	0.054181	0.289494	0.243727	0.482102	0.189295	0.158422	0.222769	0.432441	0.60641	0	0	0	0	0.034257	0	0	0	0.029154	0	0	0	0	0	0	0	0	0	0	0	0	0	0	0	0	0	0	0	0	0		
Bacteria|Bacteroidetes|Sphingobacteriia	0.023717	0	0	0	0.062088	0	2.716477	0.22205	0.162227	0.752191	0.276101	0.201282	0.579895	0.312019	0.02791	0.083149	0.156756	0.613017	0.084467	0.398997	0.215034	0.198997	1.095052	0.322837	0.168236	0	0	0	0.030466	0	0.063098	0.031684	0	0	0	0	0.663273	0.472405	0.027808	0.137029	0	0.056793	0.464737	0.058308	0.057339	0.027046	0	0	0	0.149753	0	0.033906	0	0.068862	0	0	0	0	0	0	0	0	0	0	0	Arctic	2.87E-05
Bacteria|Bacteroidetes|Sphingobacteriia|Sphingobacteriales	0.023717	0	0	0	0.062088	0	2.716477	0.22205	0.162227	0.752191	0.276101	0.201282	0.579895	0.312019	0.02791	0.083149	0.156756	0.613017	0.084467	0.398997	0.215034	0.198997	1.095052	0.322837	0.168236	0	0	0	0.030466	0	0.063098	0.031684	0	0	0	0	0.663273	0.472405	0.027808	0.137029	0	0.056793	0.464737	0.058308	0.057339	0.027046	0	0	0	0.149753	0	0.033906	0	0.068862	0	0	0	0	0	0	0	0	0	0	0	Arctic	2.87E-05
Bacteria|Bacteroidetes|Sphingobacteriia|Sphingobacteriales|Sphingobacteriaceae	0.023717	0	0	0	0.062088	0	2.585562	0.22205	0.162227	0.752191	0.276101	0.143773	0.579895	0.218414	0.02791	0.083149	0.156756	0.319835	0.084467	0.324185	0.095571	0.198997	1.095052	0.161419	0.168236	0	0	0	0	0	0	0	0	0	0	0	0.663273	0.472405	0.027808	0.137029	0	0.056793	0.464737	0.058308	0.057339	0.027046	0	0	0	0.149753	0	0.033906	0	0.068862	0	0	0	0	0	0	0	0	0	0	0		
Bacteria|Bacteroidetes|Sphingobacteriia|Sphingobacteriales|Sphingobacteriaceae|Pedobacter	0	0	0	0	0.062088	0	2.585562	0.190328	0.081113	0.752191	0.184067	0.115018	0	0.124808	0	0.083149	0	0.079959	0.028156	0.099749	0	0	0.106834	0.069179	0.056079	0	0	0	0	0	0	0	0	0	0	0	0	0	0	0	0	0	0	0	0	0	0	0	0	0	0	0	0	0.034431	0	0	0	0	0	0	0	0	0	0	0		
Bacteria|Bacteroidetes|Sphingobacteriia|Sphingobacteriales|Sphingobacteriaceae|Pedobacter|cryoconitis	0	0	0	0	0	0	2.520105	0.031721	0.081113	0.407437	0.184067	0.115018	0	0.031202	0	0.083149	0	0.026653	0	0	0	0	0.053417	0	0	0	0	0	0	0	0	0	0	0	0	0	0	0	0	0	0	0	0	0	0	0	0	0	0	0	0	0	0	0	0	0	0	0	0	0	0	0	0	0	0		
Bacteria|Caldiserica	0.071151	0	0	0	0	0	0	0	0	0	0	0	0	0	0	0	0	0	0	0	0	0	0	0	0	0	0	0	0	0	0	0	0	0	0	0	0	0	0	0	0	0	0	0	0	0	0	0	0	0	0	0	0	0	0.796614	0.165033	0.34812	0.878238	0.314237	0.106059	4.46595	0.110138	0.80625	0.534778	8.924311	Oil sands tailings pond deep	9.19E-09
Bacteria|Caldiserica|Caldisericia	0.071151	0	0	0	0	0	0	0	0	0	0	0	0	0	0	0	0	0	0	0	0	0	0	0	0	0	0	0	0	0	0	0	0	0	0	0	0	0	0	0	0	0	0	0	0	0	0	0	0	0	0	0	0	0	0.697038	0.110022	0.099463	0.647122	0.104746	0	4.421732	0.110138	0.80625	0.534778	8.924311	Oil sands tailings pond deep	7.55E-08
Bacteria|Caldiserica|Caldisericia|Caldisericales	0.071151	0	0	0	0	0	0	0	0	0	0	0	0	0	0	0	0	0	0	0	0	0	0	0	0	0	0	0	0	0	0	0	0	0	0	0	0	0	0	0	0	0	0	0	0	0	0	0	0	0	0	0	0	0	0.697038	0.110022	0.099463	0.647122	0.104746	0	4.421732	0.110138	0.80625	0.534778	8.924311	Oil sands tailings pond deep	7.55E-08
Bacteria|Caldithrix	0	0	0	0.085326	0	0	0	0	0	0	0	0	0	0	0	0	0	0	0	0	0	0	0	0	0	5.38269	3.738509	5.131941	0	0	0.189295	0.095053	0	0	0	0	0	0	0	0	0	0	0	0	0	0	0	0	0	0	0	0	0	0	0.29873	0	0	0	0	0	0.265304	0	0	0	0		
Bacteria|Caldithrix|Caldithrixae	0	0	0	0	0	0	0	0	0	0	0	0	0	0	0	0	0	0	0	0	0	0	0	0	0	5.14866	3.413422	4.342411	0	0	0	0	0	0	0	0	0	0	0	0	0	0	0	0	0	0	0	0	0	0	0	0	0	0	0	0	0	0	0	0	0	0	0	0	0		
Bacteria|Caldithrix|Caldithrixae|Caldithrixales	0	0	0	0	0	0	0	0	0	0	0	0	0	0	0	0	0	0	0	0	0	0	0	0	0	5.14866	3.413422	4.342411	0	0	0	0	0	0	0	0	0	0	0	0	0	0	0	0	0	0	0	0	0	0	0	0	0	0	0	0	0	0	0	0	0	0	0	0	0		
Bacteria|Caldithrix|Caldithrixae|Caldithrixales|BA059	0	0	0	0	0	0	0	0	0	0	0	0	0	0	0	0	0	0	0	0	0	0	0	0	0	1.95025	0.975263	2.131729	0	0	0	0	0	0	0	0	0	0	0	0	0	0	0	0	0	0	0	0	0	0	0	0	0	0	0	0	0	0	0	0	0	0	0	0	0		
Bacteria|Caldithrix|Caldithrixae|Caldithrixales|Caldithrixaceae	0	0	0	0	0	0	0	0	0	0	0	0	0	0	0	0	0	0	0	0	0	0	0	0	0	3.19841	2.438158	2.210682	0	0	0	0	0	0	0	0	0	0	0	0	0	0	0	0	0	0	0	0	0	0	0	0	0	0	0	0	0	0	0	0	0	0	0	0	0		
Bacteria|Caldithrix|Caldithrixae|Caldithrixales|Caldithrixaceae|LCP-26	0	0	0	0	0	0	0	0	0	0	0	0	0	0	0	0	0	0	0	0	0	0	0	0	0	2.10627	1.950527	1.3422	0	0	0	0	0	0	0	0	0	0	0	0	0	0	0	0	0	0	0	0	0	0	0	0	0	0	0	0	0	0	0	0	0	0	0	0	0		
Bacteria|Caldithrix|KSB1	0	0	0	0.085326	0	0	0	0	0	0	0	0	0	0	0	0	0	0	0	0	0	0	0	0	0	0.23403	0.325088	0.789529	0	0	0.189295	0.095053	0	0	0	0	0	0	0	0	0	0	0	0	0	0	0	0	0	0	0	0	0	0	0.29873	0	0	0	0	0	0.265304	0	0	0	0		
Bacteria|Chlamydiae	0	0	0	0	0	0	0	0	0.040557	0	0	0	0.045781	0	0	0.041574	0	0	0	0	0	0.042642	0	0	0	0.819105	1.666075	0.592147	0.913977	0	0.094647	0.047527	0.047736	0.086488	0.043315	0	0	0	0	0	0	0	0	0	0	0	0	0	0	0	0	0.050859	0	0	0	0	0	0	0	0	0	0	0	0	0		
Bacteria|Chlamydiae|Chlamydiia	0	0	0	0	0	0	0	0	0.040557	0	0	0	0.045781	0	0	0.041574	0	0	0	0	0	0.042642	0	0	0	0.819105	1.666075	0.592147	0.913977	0	0.094647	0.047527	0.047736	0.086488	0.043315	0	0	0	0	0	0	0	0	0	0	0	0	0	0	0	0	0.050859	0	0	0	0	0	0	0	0	0	0	0	0	0		
Bacteria|Chlamydiae|Chlamydiia|Chlamydiales	0	0	0	0	0	0	0	0	0.040557	0	0	0	0.045781	0	0	0.041574	0	0	0	0	0	0.042642	0	0	0	0.819105	1.666075	0.592147	0.913977	0	0.094647	0.047527	0.047736	0.086488	0.043315	0	0	0	0	0	0	0	0	0	0	0	0	0	0	0	0	0.050859	0	0	0	0	0	0	0	0	0	0	0	0	0		
Bacteria|Chlorobi	0.284603	0.309853	11.12574	4.095663	0.465658	0.93692	0	0.47582	0	0.094024	0.828303	0	0	0.187212	0.167457	0	0.188107	0.159918	0.084467	0.149624	0.143356	0	0	0.069179	0.084118	4.29055	3.088334	1.973823	0.091398	0.361576	0.662532	0.665371	0.190944	0.086488	0.17326	0	0	0	0	0.102771	0.086361	0	0	0	0	0	0	0	0	0	0	0	0	0	0	0.110022	0.198926	0	0	0	0	0.110138	0	0	0	India oil refineries	3.74E-05
Bacteria|Chlorobi|Ignavibacteria	0.213453	0.077463	9.30042	3.2424	0	0.681396	0	0	0	0	0.276101	0	0	0.093606	0.083729	0	0.094053	0	0	0.149624	0	0	0	0	0	4.21254	3.007062	1.89487	0.091398	0.361576	0.189295	0.475265	0.190944	0.086488	0	0	0	0	0	0	0	0	0	0	0	0	0	0	0	0	0	0	0	0	0	0.110022	0	0	0	0	0	0.110138	0	0	0	India oil refineries	7.48E-05
Bacteria|Chlorobi|Ignavibacteria|Ignavibacteriales	0.213453	0.077463	9.30042	3.2424	0	0.681396	0	0	0	0	0.276101	0	0	0.093606	0.083729	0	0.094053	0	0	0.149624	0	0	0	0	0	4.21254	3.007062	1.89487	0.091398	0.361576	0.189295	0.475265	0.190944	0.086488	0	0	0	0	0	0	0	0	0	0	0	0	0	0	0	0	0	0	0	0	0	0.110022	0	0	0	0	0	0.110138	0	0	0	India oil refineries	7.48E-05
Bacteria|Chlorobi|Ignavibacteria|Ignavibacteriales|Ignavibacteriaceae	0.213453	0.077463	9.30042	3.071747	0	0	0	0	0	0	0	0	0	0	0	0	0	0	0	0.074812	0	0	0	0	0	2.88637	1.625439	1.105341	0.091398	0	0	0	0.095472	0	0	0	0	0	0	0	0	0	0	0	0	0	0	0	0	0	0	0	0	0	0	0.110022	0	0	0	0	0	0.110138	0	0	0	India oil refineries	1.1E-05
Bacteria|Chlorobi|Ignavibacteria|Ignavibacteriales|IheB3-7	0	0	0	0.085326	0	0	0	0	0	0	0	0	0	0	0	0	0	0	0	0	0	0	0	0	0	0.85811	0.731448	0.631623	0	0.271182	0.189295	0.475265	0.095472	0.086488	0	0	0	0	0	0	0	0	0	0	0	0	0	0	0	0	0	0	0	0	0	0	0	0	0	0	0	0	0	0	0		
Bacteria|Chlorobi|Ignavibacteria|Ignavibacteriales|Melioribacteraceae	0	0	0	0.085326	0	0.681396	0	0	0	0	0.276101	0	0	0.093606	0.083729	0	0.094053	0	0	0.074812	0	0	0	0	0	0.46806	0.650176	0.157906	0	0.090394	0	0	0	0	0	0	0	0	0	0	0	0	0	0	0	0	0	0	0	0	0	0	0	0	0	0	0	0	0	0	0	0	0	0	0		
Bacteria|Chlorobi|OPB56	0.071151	0.23239	1.825316	0.853263	0	0	0	0.095164	0	0	0.092034	0	0	0	0	0	0	0.079959	0.084467	0	0.143356	0	0	0	0.084118	0	0.081272	0.078953	0	0	0.283942	0.190106	0	0	0.17326	0	0	0	0	0	0	0	0	0	0	0	0	0	0	0	0	0	0	0	0	0	0.198926	0	0	0	0	0	0	0	0		
Bacteria|Chloroflexi	1.909215	2.801591	5.475948	4.892042	0.232829	0.056783	0.049093	0	0	0.48579	0.13805	0	0.320468	0.748847	0.334915	0	40.6624	17.7775	19.72308	14.57584	12.65115	13.61706	14.04871	9.650526	15.67396	5.902757	6.542392	4.276617	1.325267	0.090394	0.473237	0.36437	0.334153	0.317124	0.649725	3.303378	0.348218	9.551437	0.041712	4.05947	2.518868	26.55056	0.046474	2.463524	0.344035	44.59809	0.071641	0	0	0.599014	0	0	0.235451	0.241017	4.016264	3.649061	6.647442	3.035312	5.35949	6.982235	12.89672	0.991244	0.345536	1.960854	6.280071	China oil refineries	3.61E-06
Bacteria|Chloroflexi|Anaerolineae	1.446734	2.685396	5.128269	4.294758	0	0.056783	0	0	0	0	0	0	0	0	0	0	3.041059	1.146075	4.730159	0.673307	0.430067	0.483278	0.454046	0.576495	0.813139	3.874497	4.551229	3.1318	0.639784	0	0.094647	0.126737	0.095472	0.144147	0.25989	1.612175	0	0.354304	0	0.445343	1.525714	1.135853	0	0.466466	0.172018	0.108182	0.071641	0	0	0.374384	0	0	0.033636	0.034431	2.223882	1.943721	6.299322	2.434413	4.050168	5.974672	10.64164	0.881106	0.345536	1.960854	5.919492	Oil sands tailings pond median	3.84E-07
Bacteria|Chloroflexi|Anaerolineae|Anaerolineales	0.996112	2.014047	1.941209	0.398189	0	0	0	0	0	0	0	0	0	0	0	0	0.595671	0.239876	1.435941	0.099749	0.047785	0.056856	0.053417	0.069179	0.028039	0.182023	0.40636	0.263176	0	0	0	0	0	0	0	0.316113	0	0.029525	0	0.102771	0.662102	0	0	0.204079	0.02867	0	0	0	0	0.037438	0	0	0	0	1.891959	1.723677	6.000933	2.095444	2.269491	5.656494	9.374072	0.697542	0.076786	1.853899	5.258432	Oil sands tailings pond median	1.44E-07
Bacteria|Chloroflexi|Anaerolineae|Anaerolineales|Anaerolinaceae	0.996112	2.014047	1.941209	0.398189	0	0	0	0	0	0	0	0	0	0	0	0	0.595671	0.239876	1.435941	0.099749	0.047785	0.056856	0.053417	0.069179	0.028039	0.182023	0.40636	0.263176	0	0	0	0	0	0	0	0.316113	0	0.029525	0	0.102771	0.662102	0	0	0.204079	0.02867	0	0	0	0	0.037438	0	0	0	0	1.891959	1.723677	6.000933	2.095444	2.269491	5.656494	9.374072	0.697542	0.076786	1.853899	5.258432	Oil sands tailings pond median	1.44E-07
Bacteria|Chloroflexi|Anaerolineae|Anaerolineales|Anaerolinaceae|Anaerolinea	0	0.051642	0.17384	0.199095	0	0	0	0	0	0	0	0	0	0	0	0	0.56432	0.213223	1.37963	0.099749	0.047785	0.056856	0.026709	0.069179	0.028039	0	0	0.026318	0	0	0	0	0	0	0	0	0	0	0	0	0	0	0	0	0	0	0	0	0	0	0	0	0	0	0	0	0	0	0	0	0	0	0	0.142608	0		
Bacteria|Chloroflexi|Anaerolineae|Anaerolineales|Anaerolinaceae|C1_B004	0	0	0	0.028442	0	0	0	0	0	0	0	0	0	0	0	0	0	0	0	0	0	0	0	0	0	0.026003	0.135453	0.052635	0	0	0	0	0	0	0	0	0	0	0	0.102771	0.028787	0	0	0	0	0	0	0	0	0	0	0	0	0	0.132769	0.18337	0.165772	0.061631	0	0.141412	1.355998	0.036713	0	0.106956	0.390626		
Bacteria|Chloroflexi|Anaerolineae|Anaerolineales|Anaerolinaceae|Longilinea	0.118585	0.413138	0.840225	0	0	0	0	0	0	0	0	0	0	0	0	0	0	0	0	0	0	0	0	0	0	0	0	0	0	0	0	0	0	0	0	0	0	0	0	0	0	0	0	0	0	0	0	0	0	0	0	0	0	0	0	0	0.033154	0	0	0	0	0	0	0	0		
Bacteria|Chloroflexi|Anaerolineae|Anaerolineales|Anaerolinaceae|SHD-231	0	0.025821	0	0.028442	0	0	0	0	0	0	0	0	0	0	0	0	0	0	0	0	0	0	0	0	0	0.026003	0	0.052635	0	0	0	0	0	0	0	0	0	0	0	0	0	0	0	0	0	0	0	0	0	0.037438	0	0	0	0	0.365115	0.770154	2.088723	0.554676	0.872881	2.757541	2.47617	0.367127	0.038393	1.105209	1.262024	Oil sands tailings pond median	9.97E-08
Bacteria|Chloroflexi|Anaerolineae|Anaerolineales|Anaerolinaceae|T78	0.450622	0.490601	0.202813	0	0	0	0	0	0	0	0	0	0	0	0	0	0	0	0	0	0	0	0	0	0	0.052007	0.243816	0.131588	0	0	0	0	0	0	0	0	0	0	0	0	0	0	0	0	0	0	0	0	0	0	0	0	0	0	0.630653	0.18337	1.293019	0.647122	0.768135	0.424237	3.124691	0.183564	0.038393	0.178259	2.043277	Oil sands tailings pond median	1.38E-08
Bacteria|Chloroflexi|Anaerolineae|DRC31	0	0	0.08692	0.056884	0	0	0	0	0	0	0	0	0	0	0	0	0	0	0	0	0	0	0	0	0	0.54607	0.650176	0.394765	0	0	0	0	0	0	0	0	0	0	0	0	0	0	0	0	0	0	0	0	0	0	0	0	0	0	0	0	0	0	0	0	0	0	0	0	0		
Bacteria|Chloroflexi|Anaerolineae|envOPS12	0	0.025821	1.854289	2.84421	0	0	0	0	0	0	0	0	0	0	0	0	0	0.026653	0.028156	0	0	0.028428	0.026709	0	0.028039	0.260033	0.162544	0.394765	0	0	0	0	0	0	0	0.284501	0	0.118101	0	0.068514	0.345445	0	0	0.029154	0.086009	0	0	0	0	0.22463	0	0	0	0	0	0.036674	0	0	1.047457	0	0	0	0	0	0		
Bacteria|Chloroflexi|Anaerolineae|GCA004	0	0.051642	0.347679	0.284421	0	0	0	0	0	0	0	0	0	0	0	0	0.470267	0.079959	0.366024	0.024937	0.047785	0	0.026709	0.02306	0.028039	0.754097	1.137807	0.684259	0.426523	0	0.063098	0	0.031824	0	0.057753	0	0	0	0	0	0.287871	0	0	0.058308	0	0	0	0	0	0	0	0	0	0	0.066385	0.036674	0.066309	0.092446	0.209491	0.141412	0.235826	0.073425	0.153571	0.035652	0.150241		
Bacteria|Chloroflexi|Anaerolineae|SBR1031	0	0	0.115893	0.199095	0	0	0	0	0	0	0	0	0	0	0	0	0.031351	0.079959	0.140779	0	0.023893	0.056856	0.026709	0.02306	0.028039	0.104013	0.027091	0.105271	0.030466	0	0	0	0	0	0	0.158056	0	0	0	0	0	0.653115	0	0.029154	0	0.027046	0	0	0	0.037438	0	0	0	0	0	0	0	0	0.104746	0.035353	0.029478	0	0	0	0.030048		
Bacteria|Chloroflexi|Anaerolineae|SBR1031|oc28	0	0	0	0	0	0	0	0	0	0	0	0	0	0	0	0	0	0.026653	0	0	0	0	0.026709	0	0	0	0	0	0	0	0	0	0	0	0	0.158056	0	0	0	0	0	0.653115	0	0.029154	0	0.027046	0	0	0	0	0	0	0	0	0	0	0	0	0	0	0	0	0	0	0		
Bacteria|Chloroflexi|Anaerolineae|SHA-20	0	0	0	0.028442	0	0	0	0	0	0	0	0	0	0	0	0	0	0	0.028156	0.024937	0.023893	0	0.026709	0	0.028039	0.598077	0.704357	0.631623	0	0	0	0	0	0.057659	0	0	0	0	0	0	0.028787	0	0	0	0	0	0	0	0	0	0	0	0	0	0.066385	0	0	0.061631	0.104746	0.070706	0.117913	0	0	0	0.060096		
Bacteria|Chloroflexi|C0119	0	0	0	0	0	0	0	0	0	0.047012	0.046017	0	0	0	0.041864	0	0.56432	0.079959	0.337869	0.037406	0.179195	0	0.040063	0.103769	0.126177	0	0	0	0	0	0	0	0	0	0	0.047417	0	0.088576	0	0.051386	0	0.212972	0	0.043731	0.043004	0.162273	0	0	0	0	0	0	0	0	0	0	0	0	0	0	0	0	0	0	0		
Bacteria|Chloroflexi|Chloroflexi	0	0	0	0	0	0	0	0	0	0.062683	0	0	0	0	0	0	25.30035	3.598144	2.871882	0.398997	1.36188	0.511706	1.655932	0.807093	0.897257	0	0	0	0	0	0	0	0	0	0	0.126445	0	0.029525	0	0.068514	0	0.028396	0	0.029154	0	0.027046	0	0	0	0	0	0	0	0	0	0	0	0	0	0	0	0	0	0	0	China oil refineries	1.18E-06
Bacteria|Chloroflexi|Chloroflexi|Chloroflexales	0	0	0	0	0	0	0	0	0	0	0	0	0	0	0	0	0.344862	0.346488	0.619426	0.024937	0.358389	0.113712	0.106834	0.184478	0.140196	0	0	0	0	0	0	0	0	0	0	0	0	0	0	0	0	0	0	0	0	0	0	0	0	0	0	0	0	0	0	0	0	0	0	0	0	0	0	0	0		
Bacteria|Chloroflexi|Chloroflexi|Roseiflexales	0	0	0	0	0	0	0	0	0	0	0	0	0	0	0	0	24.17171	2.691945	1.801965	0.224436	0.573423	0.255853	0.988217	0.092239	0.308432	0	0	0	0	0	0	0	0	0	0	0.094834	0	0.029525	0	0.068514	0	0.028396	0	0.029154	0	0.027046	0	0	0	0	0	0	0	0	0	0	0	0	0	0	0	0	0	0	0	China oil refineries	3.59E-07
Bacteria|Chloroflexi|Chloroflexi|Roseiflexales|Kouleothrixaceae	0	0	0	0	0	0	0	0	0	0	0	0	0	0	0	0	24.17171	2.691945	1.801965	0.224436	0.573423	0.255853	0.988217	0.092239	0.308432	0	0	0	0	0	0	0	0	0	0	0.094834	0	0.029525	0	0.068514	0	0.028396	0	0.029154	0	0.027046	0	0	0	0	0	0	0	0	0	0	0	0	0	0	0	0	0	0	0	China oil refineries	3.59E-07
Bacteria|Chloroflexi|Dehalococcoidetes	0.071151	0	0.260759	0	0	0	0	0	0	0	0	0	0	0	0.083729	0	0	0	0	0	0	0	0	0	0	0.62408	0.162544	0.236859	0	0	0	0	0.095472	0.086488	0.17326	0	0	0.088576	0	0.102771	0.086361	0	0	0	0	0	0	0	0	0.22463	0	0	0	0	1.792382	1.650329	0.298389	0.554676	1.152203	0.954533	2.210866	0.110138	0	0	0.360578	Oil sands tailings pond median	2.62E-05
Bacteria|Chloroflexi|Dehalococcoidetes|Dehalococcoidales	0.071151	0	0.17384	0	0	0	0	0	0	0	0	0	0	0	0.083729	0	0	0	0	0	0	0	0	0	0	0.31204	0.162544	0.236859	0	0	0	0	0.095472	0	0.17326	0	0	0.088576	0	0.102771	0.086361	0	0	0	0	0	0	0	0	0.112315	0	0	0	0	0.199154	0.440088	0.099463	0.277338	0.314237	0.848474	1.414954	0	0	0	0		
Bacteria|Chloroflexi|Dehalococcoidetes|Dehalococcoidales|Dehalococcoidaceae	0.071151	0	0.17384	0	0	0	0	0	0	0	0	0	0	0	0.083729	0	0	0	0	0	0	0	0	0	0	0.31204	0.162544	0.236859	0	0	0	0	0.095472	0	0.17326	0	0	0.088576	0	0.102771	0.086361	0	0	0	0	0	0	0	0	0.112315	0	0	0	0	0.199154	0.440088	0.099463	0.277338	0.314237	0.848474	1.414954	0	0	0	0		

Table S2. II

Bacterial clades	I1	I2	I3	I4	A1	A10	A11	A12	A2	A3	A4	A5	A6	A7	A8	A9	C1	C2	C3	C4	C5	C6	C7	C8	C9	M1	M2	M3	DWH1	DWH2	DWH3	DWH4	DWH5	DWH6	DWH7
(Habitat)	India oil refineries				Arctic												China oil refineries									Mangrove			Marine sediments						
Bacteria|Chloroflexi|Dehalococcoidetes|GIF9	0	0	0.08692	0	0	0	0	0	0	0	0	0	0	0	0	0	0	0	0	0	0	0	0	0	0	0.15602	0	0	0	0	0	0	0	0.086488	0
Bacteria|Chloroflexi|Ellin6529	0.035575	0	0	0	0	0	0	0	0	0	0	0	0	0	0	0	1.034587	1.559196	1.647109	1.645861	2.472888	1.151338	1.4022	0.622615	0.883237	1.17015	1.137807	0.631623	0	0	0	0.047527	0	0.043244	0
Bacteria|Chloroflexi|Gitt-GS-136	0	0	0	0.213316	0.139697	0	0	0	0	0.188048	0	0	0.091562	0.655241	0.041864	0	0.14108	0.159918	0.295635	0.336653	0.609262	0.170569	0.040063	0.138359	1.009414	0	0	0	0	0	0	0	0	0	0
Bacteria|Chloroflexi|Ktedonobacteria	0	0.038732	0	0.042663	0	0	0.049093	0	0	0	0	0	0.228906	0	0.083729	0	2.492414	2.038948	2.322846	1.496237	1.541075	2.473245	2.163395	1.383588	1.892652	0.23403	0.60954	0.118429	0.045699	0	0	0	0.047736	0.043244	0.043315
Bacteria|Chloroflexi|Ktedonobacteria|B12-WMSP1	0	0	0	0	0	0	0	0	0	0	0	0	0	0	0	0	0	0	0.042234	0.037406	0	0	0	0	0	0	0	0	0	0	0	0	0	0	0
Bacteria|Chloroflexi|Ktedonobacteria|JG30-KF-AS9	0	0	0	0	0	0	0	0	0	0	0	0	0	0	0	0	1.034587	0.919526	1.351474	0.82293	0.824296	1.407191	1.041635	1.003101	1.17765	0	0	0	0	0	0	0	0	0	0
Bacteria|Chloroflexi|Ktedonobacteria|Thermogemmatisporales	0	0.038732	0	0.042663	0	0	0.049093	0	0	0	0	0	0.228906	0	0.083729	0	1.363774	1.119423	0.886905	0.635901	0.716779	1.023412	1.12176	0.380487	0.715002	0.23403	0.60954	0.118429	0.045699	0	0	0	0.047736	0.043244	0.043315
Bacteria|Chloroflexi|Ktedonobacteria|Thermogemmatisporales|Thermogemmatisporaceae	0	0.038732	0	0.042663	0	0	0.049093	0	0	0	0	0	0.228906	0	0.083729	0	1.363774	1.119423	0.886905	0.635901	0.716779	1.023412	1.12176	0.380487	0.715002	0.23403	0.60954	0.118429	0.045699	0	0	0	0.047736	0.043244	0.043315
Bacteria|Chloroflexi|P2-11E	0	0	0	0	0	0	0	0	0	0	0	0	0	0	0	0	0	0	0	0	0	0	0	0.03459	0	0	0	0	0	0	0	0	0	0	0
Bacteria|Chloroflexi|S085	0	0	0	0.085326	0	0	0	0	0	0	0	0	0	0	0	0	0.28216	1.359299	0.675737	3.441345	1.146846	1.108696	0.801257	0.622615	1.17765	0	0	0	0.639784	0	0	0.095053	0	0	0
Bacteria|Chloroflexi|SHA-26	0	0	0	0	0	0	0	0	0	0	0	0	0	0	0	0	0.376213	0	0.168934	0	0	0	0	0	0	0	0	0	0	0	0	0	0	0	0
Bacteria|Chloroflexi|Thermomicrobia	0.355754	0.077463	0.08692	0.170653	0.093132	0	0	0	0	0.188048	0.092034	0	0	0.093606	0.083729	0	5.172935	4.957443	4.307824	3.628375	1.899464	5.33027	4.126475	3.009304	6.098544	0	0.081272	0.078953	0	0	0	0	0.095472	0	0
Bacteria|Chloroflexi|Thermomicrobia|AKYG1722	0	0	0	0	0	0	0	0	0	0	0	0	0	0	0	0	0	0.319835	0.084467	0.523683	0.071678	0.341137	0.400629	0.207538	0.336471	0	0.081272	0.078953	0	0	0	0	0.095472	0	0
Bacteria|Chloroflexi|Thermomicrobia|JG30-KF-CM45	0.355754	0.077463	0.08692	0.085326	0	0	0	0	0	0	0	0	0	0	0	0	5.078882	4.557649	3.885488	2.768039	1.648592	4.775922	3.685784	2.697997	5.551778	0	0	0	0	0	0	0	0	0	0
Bacteria|Chloroflexi|TK10	0	0	0	0	0	0	0	0	0	0	0	0	0	0	0	0	1.692961	1.439258	1.858277	0.748119	2.006981	1.108696	1.842892	1.314409	1.009414	0	0	0.078953	0	0	0	0	0	0	0
Bacteria|Chloroflexi|TK10|AKYG885	0	0	0	0	0	0	0	0	0	0	0	0	0	0	0	0	0.84648	0.879546	1.267007	0.448871	1.576914	0.852843	1.522389	1.17605	0.757061	0	0	0	0	0	0	0	0	0	0
Bacteria|Chloroflexi|TK10|AKYG885|Dolo_23	0	0	0	0	0	0	0	0	0	0	0	0	0	0	0	0	0.84648	0.879546	1.267007	0.448871	1.576914	0.852843	1.522389	1.17605	0.757061	0	0	0	0	0	0	0	0	0	0
Bacteria|Chloroflexi|TK10|B07_WMSP1	0	0	0	0	0	0	0	0	0	0	0	0	0	0	0	0	0.752427	0.319835	0.337869	0.149624	0.071678	0.085284	0.160251	0	0	0	0	0.078953	0	0	0	0	0	0	0
Bacteria|Chloroflexi|TK17	0	0	0	0.085326	0	0	0	0	0	0	0	0	0	0	0	0	0.56432	1.439258	0.506803	1.945108	0.860135	1.193981	1.522389	0.968512	1.682357	0	0	0	0	0	0	0	0	0	0
Bacteria|Chloroflexi|TK17|mle1-48	0	0	0	0.085326	0	0	0	0	0	0	0	0	0	0	0	0	0.56432	1.439258	0.506803	1.945108	0.860135	1.193981	1.522389	0.968512	1.682357	0	0	0	0	0	0	0	0	0	0
Bacteria|Cyanobacteria	0.326108	0.852097	1.738396	0.042663	0	0	0	0.031721	0	0	0	0	0	0	0	0	1.536205	3.931305	1.872355	0.985023	6.116514	1.492476	7.925771	8.174699	11.22973	1.313168	3.860417	3.263388	1.827955	0.135591	0.047324	0.269317	0.047736	0.850468	0.158822
Bacteria|Cyanobacteria|4C0d-2	0.284603	0.69717	1.738396	0.042663	0	0	0	0	0	0	0	0	0	0	0	0	0.047027	0.199897	0.126701	0.037406	0.071678	0.810201	0.040063	0.207538	0.042059	0	0	0	0	0	0	0	0	0	0
Bacteria|Cyanobacteria|4C0d-2|MLE1-12	0.284603	0.69717	1.608016	0.042663	0	0	0	0	0	0	0	0	0	0	0	0	0	0	0.042234	0	0.035839	0	0	0.069179	0	0	0	0	0	0	0	0	0	0	0
Bacteria|Cyanobacteria|4C0d-2|SM1D11	0	0	0.08692	0	0	0	0	0	0	0	0	0	0	0	0	0	0.047027	0.199897	0.084467	0.037406	0.035839	0.810201	0.040063	0.138359	0.042059	0	0	0	0	0	0	0	0	0	0
Bacteria|Cyanobacteria|Chloroplast	0	0.154927	0	0	0	0	0	0	0	0	0	0	0	0	0	0	0.188107	0.319835	0.084467	0.299247	4.372352	0.170569	1.041635	0.899332	1.093532	0.7801	0.731448	3.079164	0	0	0	0	0	0	0
Bacteria|Cyanobacteria|Chloroplast|Stramenopiles	0	0.154927	0	0	0	0	0	0	0	0	0	0	0	0	0	0	0.188107	0.159918	0	0.299247	2.006981	0.170569	0.160251	0.207538	0.252354	0.7801	0.731448	3.079164	0	0	0	0	0	0	0
Bacteria|Cyanobacteria|Chloroplast|Streptophyta	0	0	0	0	0	0	0	0	0	0	0	0	0	0	0	0	0	0.159918	0	0	2.078659	0	0.56088	0.622615	0.757061	0	0	0	0	0	0	0	0	0	0
Bacteria|Cyanobacteria|ML635J-21	0	0	0	0	0	0	0	0	0	0	0	0	0	0	0	0	0.094053	0.399794	0.042234	0.112218	0.071678	0.426422	1.522389	0.242128	0.630884	0	0	0	0	0	0	0.047527	0	0	0
Bacteria|Cyanobacteria|Oscillatoriophycideae	0	0	0	0	0	0	0	0	0	0	0	0	0	0	0	0	0.736751	1.172728	0.295635	0.162092	0.776511	0.056856	1.335429	3.101543	5.986387	0.533068	3.12897	0.184224	0	0	0	0	0	0	0
Bacteria|Cyanobacteria|Oscillatoriophycideae|Chroococcales	0	0	0	0	0	0	0	0	0	0	0	0	0	0	0	0	0.047027	0	0.056311	0	0	0.028428	0.053417	0.02306	0.168236	0.494063	3.088334	0.157906	0	0	0	0	0	0	0
Bacteria|Cyanobacteria|Oscillatoriophycideae|Oscillatoriales	0	0	0	0	0	0	0	0	0	0	0	0	0	0	0	0	0.28216	0.852893	0.154856	0.137155	0.68094	0.028428	0.106834	2.594227	4.808737	0.039005	0.040636	0	0	0	0	0	0	0	0
Bacteria|Cyanobacteria|Oscillatoriophycideae|Oscillatoriales|Phormidiaceae	0	0	0	0	0	0	0	0	0	0	0	0	0	0	0	0	0.28216	0.852893	0.154856	0.137155	0.68094	0.028428	0.106834	2.594227	4.808737	0.039005	0.040636	0	0	0	0	0	0	0	0
Bacteria|Cyanobacteria|Oscillatoriophycideae|Oscillatoriales|Phormidiaceae|Phormidium	0	0	0	0	0	0	0	0	0	0	0	0	0	0	0	0	0.28216	0.772935	0.112623	0.099749	0.645101	0.028428	0.106834	2.559638	4.514325	0	0	0	0	0	0	0	0	0	0
Bacteria|Cyanobacteria|Synechococcophycideae	0	0	0	0	0	0	0	0	0	0	0	0	0	0	0	0	0.376213	1.119423	1.013606	0.299247	0.752618	0	1.482326	1.452767	2.102946	0	0	0	1.827955	0.135591	0.047324	0.190106	0.047736	0.821639	0.129945
Bacteria|Cyanobacteria|Synechococcophycideae|Pseudanabaenales	0	0	0	0	0	0	0	0	0	0	0	0	0	0	0	0	0.376213	0.479753	0.844671	0.149624	0.573423	0	0.881383	0.207538	0.504707	0	0	0	0	0	0	0	0	0	0
Bacteria|Cyanobacteria|Synechococcophycideae|Pseudanabaenales|Pseudanabaenaceae	0	0	0	0	0	0	0	0	0	0	0	0	0	0	0	0	0.376213	0.479753	0.844671	0.149624	0.573423	0	0.881383	0.207538	0.504707	0	0	0	0	0	0	0	0	0	0
Bacteria|Cyanobacteria|Synechococcophycideae|Pseudanabaenales|Pseudanabaenaceae|Leptolyngbya	0	0	0	0	0	0	0	0	0	0	0	0	0	0	0	0	0.376213	0.479753	0.844671	0.149624	0.573423	0	0.881383	0.207538	0.504707	0	0	0	0	0	0	0	0	0	0
Bacteria|Deferribacteres	0	0	0.04346	0	0	0	0	0	0	0	0	0	0	0	0	0	0	0	0	0	0	0	0	0	0	0	0	0	0	0	0	0	0	0	0
Bacteria|Deferribacteres|Deferribacteres	0	0	0.04346	0	0	0	0	0	0	0	0	0	0	0	0	0	0	0	0	0	0	0	0	0	0	0	0	0	0	0	0	0	0	0	0
Bacteria|Deferribacteres|Deferribacteres|Deferribacterales	0	0	0.04346	0	0	0	0	0	0	0	0	0	0	0	0	0	0	0	0	0	0	0	0	0	0	0	0	0	0	0	0	0	0	0	0
Bacteria|Deferribacteres|Deferribacteres|Deferribacterales|Deferribacteraceae	0	0	0.04346	0	0	0	0	0	0	0	0	0	0	0	0	0	0	0	0	0	0	0	0	0	0	0	0	0	0	0	0	0	0	0	0
Bacteria|Deferribacteres|Deferribacteres|Deferribacterales|Deferribacteraceae|Deferribacter	0	0	0.04346	0	0	0	0	0	0	0	0	0	0	0	0	0	0	0	0	0	0	0	0	0	0	0	0	0	0	0	0	0	0	0	0
Bacteria|Elusimicrobia	0	0.69717	0.17384	0.085326	0.186263	0.255524	0	0.190328	0.24334	0.658167	0.092034	0.172527	0.366249	0.093606	0.586101	0.083149	0	0.159918	0.084467	0	0.143356	0	0.080126	0	0.084118	0	0	0	0.182795	0	0.236619	0	0	0	0
Bacteria|Elusimicrobia|Elusimicrobia	0	0.69717	0.17384	0.085326	0.186263	0.255524	0	0.190328	0.24334	0.658167	0.092034	0.172527	0.366249	0.093606	0.586101	0.083149	0	0.159918	0.084467	0	0.143356	0	0.080126	0	0.084118	0	0	0	0.182795	0	0.189295	0	0	0	0
Bacteria|Elusimicrobia|Elusimicrobia|Elusimicrobiales	0	0.619707	0	0	0	0	0	0	0	0	0	0	0	0	0	0	0	0	0	0	0	0	0	0	0.084118	0	0	0	0	0	0	0	0	0	0
Bacteria|Elusimicrobia|Elusimicrobia|FAC88	0	0	0	0.085326	0	0.085175	0	0	0.24334	0.188048	0	0	0.274687	0	0.502372	0.083149	0	0	0	0	0.071678	0	0	0	0	0	0	0	0	0	0	0	0	0	0
Bacteria|EM3	0	0	0	0	0	0	0	0	0	0	0	0	0	0	0	0	0	0	0	0	0	0	0	0	0	0	0	0	0	0	0	0	0	0	0
Bacteria|Firmicutes	0.063528	0.23239	0.481784	0	0.465658	0.149055	0.147279	0.080889	0.097336	0.211554	0.253092	0.021566	0.190755	0.304219	0.090706	0.041574	1.963476	0.737873	1.008946	1.271683	0.888635	1.153098	0.699415	0.705657	1.338375	4.014543	2.201889	3.149094	0.520314	0.338978	0.392787	0.380212	0.367568	0.090813	0.051978
Bacteria|Firmicutes|Bacilli	0.027952	0	0.034147	0	0.023283	0	0	0.057098	0.016223	0.023506	0	0	0.076302	0.046803	0.006977	0.020787	0.730033	0.299877	0.49068	0.695216	0.35344	0.710229	0.366893	0.339007	0.594533	0.037891	0.044119	0.037597	0.447196	0.022599	0.023662	0.009505	0.023868	0	0
Bacteria|Firmicutes|Bacilli|Bacillales	0.027952	0	0.034147	0	0.023283	0	0	0	0	0.023506	0	0	0.076302	0.046803	0.006977	0.020787	0.730033	0.299877	0.469564	0.558061	0.35344	0.676115	0.346862	0.339007	0.560886	0.022289	0.044119	0.011279	0.447196	0.022599	0.023662	0.009505	0.023868	0	0
Bacteria|Firmicutes|Bacilli|Lactobacillales	0	0	0	0	0	0	0	0.057098	0.016223	0	0	0	0	0	0	0	0	0	0.021117	0.112218	0	0.034114	0.020031	0	0.033647	0.015602	0	0	0	0	0	0	0	0	0
Bacteria|Firmicutes|Bacilli|Lactobacillales|Carnobacteriaceae	0	0	0	0	0	0	0	0	0	0	0	0	0	0	0	0	0	0	0.021117	0.037406	0	0	0.020031	0	0	0	0	0	0	0	0	0	0	0	0
Bacteria|Firmicutes|Bacilli|Lactobacillales|Carnobacteriaceae|Trichococcus	0	0	0	0	0	0	0	0	0	0	0	0	0	0	0	0	0	0	0	0	0	0	0	0	0	0	0	0	0	0	0	0	0	0	0
Bacteria|Firmicutes|Clostridia	0.035575	0.193658	0.425907	0	0	0	0.049093	0	0	0	0	0	0.045781	0	0	0	0.998309	0.358038	0.370449	0.40814	0.481437	0.314943	0.232365	0.262882	0.596636	3.17705	1.771728	2.243015	0.027419	0.271182	0.227154	0.038021	0.248228	0.069191	0.051978
Bacteria|Firmicutes|Clostridia|Clostridiales	0.035575	0.193658	0.425907	0	0	0	0.049093	0	0	0	0	0	0.045781	0	0	0	0.904256	0.358038	0.349332	0.314625	0.463517	0.25098	0.192302	0.214456	0.596636	3.17705	1.771728	2.211434	0.027419	0.271182	0.227154	0.038021	0.248228	0.069191	0.051978
Bacteria|Firmicutes|Clostridia|Clostridiales|Acidaminobacteraceae	0	0	0	0	0	0	0	0	0	0	0	0	0	0	0	0	0	0	0	0	0	0	0	0	0	0.561672	0.14629	1.105341	0.01828	0.271182	0.227154	0.038021	0.248228	0.069191	0.051978
Bacteria|Firmicutes|Clostridia|Clostridiales|Acidaminobacteraceae|Acidaminobacter	0	0	0	0	0	0	0	0	0	0	0	0	0	0	0	0	0	0	0	0	0	0	0	0	0	0	0	0	0	0	0	0	0	0	0
Bacteria|Firmicutes|Clostridia|Clostridiales|Acidaminobacteraceae|Fusibacter	0	0	0	0	0	0	0	0	0	0	0	0	0	0	0	0	0	0	0	0	0	0	0	0	0	0.499264	0.130035	0.931645	0	0.036158	0.018929	0	0.019094	0.017298	0
Bacteria|Firmicutes|Clostridia|Clostridiales|Clostridiaceae	0	0.116195	0.239029	0	0	0	0.049093	0	0	0	0	0	0.045781	0	0	0	0.319781	0.06219	0.084467	0.097671	0.101544	0.085284	0.060094	0	0.218706	1.32747	0.357597	0.434241	0.00914	0	0	0	0	0	0
Bacteria|Firmicutes|Clostridia|Clostridiales|Clostridiaceae|Clostridium	0	0.116195	0.04346	0	0	0	0.049093	0	0	0	0	0	0.045781	0	0	0	0.296268	0.039979	0.084467	0.081046	0.071678	0.085284	0.040063	0	0.133187	1.319669	0.357597	0.434241	0	0	0	0	0	0	0
Bacteria|Firmicutes|Clostridia|Clostridiales|Eubacteriaceae	0	0	0	0	0	0	0	0	0	0	0	0	0	0	0	0	0	0	0	0	0	0	0	0	0	0.031204	0.016254	0.015791	0	0	0	0	0	0	0
Bacteria|Firmicutes|Clostridia|Clostridiales|Eubacteriaceae|Acetobacterium	0	0	0	0	0	0	0	0	0	0	0	0	0	0	0	0	0	0	0	0	0	0	0	0	0	0	0	0	0	0	0	0	0	0	0
Bacteria|Firmicutes|Clostridia|Clostridiales|Mogibacteriaceae	0	0	0	0	0	0	0	0	0	0	0	0	0	0	0	0	0	0	0	0	0	0.012183	0	0	0.012017	0.011144	0	0.011279	0	0	0	0	0	0	0
Bacteria|Firmicutes|Clostridia|Clostridiales|Mogibacteriaceae|Anaerovorax	0	0	0	0	0	0	0	0	0	0	0	0	0	0	0	0	0	0	0	0	0	0.012183	0	0	0.012017	0	0	0	0	0	0	0	0	0	0
Bacteria|Firmicutes|Clostridia|Clostridiales|Peptococcaceae	0	0	0.04346	0	0	0	0	0	0	0	0	0	0	0	0	0	0.385619	0.215889	0.202721	0.104737	0.25804	0.11087	0.112176	0.179866	0.260765	0	0	0	0	0	0	0	0	0	0
Bacteria|Firmicutes|Clostridia|Clostridiales|Peptococcaceae|Desulfosporosinus	0	0	0	0	0	0	0	0	0	0	0	0	0	0	0	0	0.338592	0.175909	0.202721	0.104737	0.25804	0.068227	0.112176	0.110687	0.218706	0	0	0	0	0	0	0	0	0	0
Bacteria|Firmicutes|Clostridia|Clostridiales|Peptococcaceae|Desulfosporosinus|meridiei	0	0	0	0	0	0	0	0	0	0	0	0	0	0	0	0	0.338592	0.175909	0.202721	0.104737	0.25804	0.068227	0.112176	0.110687	0.218706	0	0	0	0	0	0	0	0	0	0
Bacteria|Firmicutes|Clostridia|Clostridiales|Peptococcaceae|Desulfotomaculum_Desulfovirgula	0	0	0	0	0	0	0	0	0	0	0	0	0	0	0	0	0	0	0	0	0	0	0	0	0	0	0	0	0	0	0	0	0	0	0
Bacteria|Firmicutes|Clostridia|Clostridiales|Peptococcaceae|Pelotomaculum	0	0	0	0	0	0	0	0	0	0	0	0	0	0	0	0	0	0.039979	0	0	0	0.042642	0	0	0	0	0	0	0	0	0	0	0	0	0
Bacteria|Firmicutes|Clostridia|Clostridiales|Peptostreptococcaceae	0	0	0	0	0	0	0	0	0	0	0	0	0	0	0	0	0.067181	0	0.024133	0.074812	0	0	0	0	0	1.17015	1.137807	0.552671	0	0	0	0	0	0	0
Bacteria|Firmicutes|Clostridia|Clostridiales|Tissierellaceae	0	0	0.034768	0	0	0	0	0	0	0	0	0	0	0	0	0	0	0	0	0	0	0	0	0	0	0.031204	0.016254	0.078953	0	0	0	0	0	0	0
Bacteria|Firmicutes|Clostridia|Clostridiales|Tissierellaceae|Tissierella_Soehngenia	0	0	0.017384	0	0	0	0	0	0	0	0	0	0	0	0	0	0	0	0	0	0	0	0	0	0	0	0	0	0	0	0	0	0	0	0
Bacteria|Firmicutes|Clostridia|Thermoanaerobacterales	0	0	0	0	0	0	0	0	0	0	0	0	0	0	0	0	0	0	0	0	0	0	0	0	0	0	0	0	0	0	0	0	0	0	0
Bacteria|Firmicutes|Erysipelotrichi	0	0.019366	0	0	0	0	0	0	0	0	0	0	0	0	0	0	0	0	0	0	0	0	0	0	0	0	0	0	0	0	0	0	0	0	0
Bacteria|Firmicutes|Erysipelotrichi|Erysipelotrichales	0	0.019366	0	0	0	0	0	0	0	0	0	0	0	0	0	0	0	0	0	0	0	0	0	0	0	0	0	0	0	0	0	0	0	0	0
Bacteria|Firmicutes|Erysipelotrichi|Erysipelotrichales|Erysipelotrichaceae	0	0.019366	0	0	0	0	0	0	0	0	0	0	0	0	0	0	0	0	0	0	0	0	0	0	0	0	0	0	0	0	0	0	0	0	0
Bacteria|Firmicutes|Erysipelotrichi|Erysipelotrichales|Erysipelotrichaceae|PSB-M-3	0	0	0	0	0	0	0	0	0	0	0	0	0	0	0	0	0	0	0	0	0	0	0	0	0	0	0	0	0	0	0	0	0	0	0
Bacteria|Gemmatimonadetes	0	0	0	0.511958	2.607683	2.640411	0.196372	0.761313	0.648907	4.983266	0.552202	1.207692	1.739684	0.280817	1.925761	0.58204	0.940534	4.317773	0.929138	4.339088	6.666045	2.132108	4.166538	7.194657	3.953539	3.04239	3.657238	3.23707	2.102148	0	0.37859	0.380212	0.190944	0.172977	0.17326
Bacteria|Gemmatimonadetes|Gemm-1	0	0	0	0.085326	0	0	0	0	0	0	0	0	0	0	0	0	0.188107	0.479753	0.253401	0.448871	1.218524	0.341137	0.320503	0.276718	0.336471	0.39005	0	0.157906	0.274193	0	0.189295	0.095053	0.095472	0	0
Bacteria|Gemmatimonadetes|Gemm-2	0	0	0	0	0	0	0	0	0	0	0	0	0	0	0	0	0	0.239876	0.084467	0.598495	0.358389	0	0.160251	0.138359	0	1.32617	1.219079	0.947435	0.274193	0	0.094647	0.190106	0.095472	0	0.08663
Bacteria|Gemmatimonadetes|Gemm-3	0	0	0	0	0	0	0	0	0	0	0	0	0	0	0	0	0.188107	1.359299	0.084467	0.897742	2.150337	0.511706	1.923018	2.905535	1.934711	0	0	0	0	0	0	0	0	0	0
Bacteria|Gemmatimonadetes|Gemm-4	0	0	0	0	0	0	0	0	0	0	0	0	0	0	0	0	0	0.079959	0	0.074812	0	0	0.080126	0	0	0.7801	2.113071	1.3422	1.553761	0	0.094647	0.095053	0	0.172977	0.08663
Bacteria|Gemmatimonadetes|Gemm-5	0	0	0	0	0	0	0	0	0	0	0	0	0	0	0	0	0.094053	0.63967	0.168934	1.645861	0.860135	0.085284	0	1.591126	0.757061	0.23403	0.325088	0.394765	0	0	0	0	0	0	0
Bacteria|Gemmatimonadetes|Gemmatimonadetes	0	0	0	0	1.024447	2.214538	0	0.761313	0	1.128287	0	1.035165	0.549374	0.280817	1.842032	0.249446	0.28216	0.719629	0.168934	0.374059	1.36188	0.767559	1.12176	0.415076	0.084118	0	0	0.157906	0	0	0	0	0	0	0
Bacteria|Gemmatimonadetes|Gemmatimonadetes|Gemmatimonadales	0	0	0	0	1.024447	2.214538	0	0.761313	0	1.128287	0	1.035165	0.549374	0.187212	1.842032	0.249446	0.28216	0.239876	0.084467	0	0.716779	0.59699	0.320503	0.345897	0.084118	0	0	0.078953	0	0	0	0	0	0	0
Bacteria|Gemmatimonadetes|Gemmatimonadetes|Gemmatimonadales|Gemmatimonadaceae	0	0	0	0	0	0	0	0	0	0	0	0	0	0	0	0	0.094053	0.159918	0	0	0.501745	0.341137	0.160251	0.207538	0	0	0	0	0	0	0	0	0	0	0
Bacteria|Gemmatimonadetes|Gemmatimonadetes|Gemmatimonadales|Gemmatimonadaceae|Gemmatimonas	0	0	0	0	0	0	0	0	0	0	0	0	0	0	0	0	0.094053	0.159918	0	0	0.501745	0.341137	0.160251	0.207538	0	0	0	0	0	0	0	0	0	0	0
Bacteria|Gemmatimonadetes|Gemmatimonadetes|KD8-87	0	0	0	0	0	0	0	0	0	0	0	0	0	0	0	0	0	0.239876	0	0.149624	0.143356	0	0.641006	0	0	0	0	0	0	0	0	0	0	0	0
Bacteria|Gemmatimonadetes|Gemmatimonadetes|N1423WL	0	0	0	0	0	0	0	0	0	0	0	0	0	0.093606	0	0	0	0.239876	0	0.149624	0.215034	0.085284	0.160251	0.069179	0	0	0	0	0	0	0	0	0	0	0
Bacteria|GN02	0	0	0	0	0	0	0	0	0	0	0	0	0	0	0	0	0	0.039979	0	0.074812	0.071678	0	0	0.03459	0	0.15602	0.040636	0	0.319892	0.361576	0.473237	0.570318	0.095472	0.216221	0.216575
Bacteria|GN02|BD1-5	0	0	0	0	0	0	0	0	0	0	0	0	0	0	0	0	0	0	0	0	0	0	0	0	0	0	0	0	0.091398	0.361576	0.473237	0.522792	0.095472	0.172977	0.216575
Bacteria|GN04	0	0	0	0.085326	0	0	0	0	0	0	0.092034	0	0	0	0	0	0	0	0	0	0	0	0	0	0	4.91463	4.713773	6.158329	0.182795	0.271182	0.946475	0.285159	0.190944	0.086488	0.77967
Bacteria|GN04|GN15	0	0	0	0.085326	0	0	0	0	0	0	0.092034	0	0	0	0	0	0	0	0	0	0	0	0	0	0	4.83662	4.632501	6.158329	0.182795	0.271182	0.851827	0.285159	0.190944	0.086488	0.77967
Bacteria|H-178	0	0	0	0	0	0	0	0	0	0	0	0	0	0	0	0	0	0	0	0	0	0	0	0	0	0	0	0	0	0	0	0	0	0	0.08663
Bacteria|Nitrospirae	0	0	0.17384	0.170653	0.558789	0.511047	0	0	0.324453	1.128287	0.184067	0	0	1.029664	0.586101	0	0.376213	0.799588	0.337869	1.047366	1.146846	0.085284	0.56088	0.138359	0.252354	0.23403	0.325088	0.236859	1.188171	0	0.473237	0.190106	0.095472	0	0
Bacteria|Nitrospirae|Nitrospira	0	0	0.17384	0.170653	0.558789	0.511047	0	0	0.324453	1.128287	0.184067	0	0	1.029664	0.586101	0	0.376213	0.799588	0.337869	1.047366	1.146846	0.085284	0.56088	0.138359	0.252354	0.23403	0.325088	0.236859	1.188171	0	0.473237	0.190106	0.095472	0	0

Bacterial clades	Tu1	Tu2	Tu3	Tu4	Tb1	Tb2	Tb3	Tb4	Tp1	Tp2	Tp3	Tp4	OSC1	OSC12	OSC3	OSC4	OSC5	OSC7	OSC9	OSTPu1	OSTPu2	OSTPu4	OSTPu3	OSTPm2	OSTPm6	OSTPm4	OSTPd1	OSTPd2	OSTPd3	OSTPd4	Differentially abundant class (one class test, non-strict)	p-value
(Habitat)	Taiga upper active layer				Taiga bottom active layer				Taiga permafrost layer				Oil sands core							Oil sands tailings pond upper				Oil sands tailings pond median			Oil sands tailings pond deep					
Bacteria|Chloroflexi|Dehalococcoidetes|GIF9	0	0	0	0	0	0	0	0	0	0	0	0	0	0	0.112315	0	0	0	0	1.593229	1.210241	0.198926	0.277338	0.837966	0.106059	0.795912	0.110138	0	0	0.360578		
Bacteria|Chloroflexi|Ellin6529	0.853504	0	1.151487	0.041712	2.826214	0.345445	0.724106	0.046474	0.655968	0	1.176481	0	0	0	0	0	0	0	0	0	0	0	0	0	0	0	0	0	0	0	China oil refineries	4.97E-07
Bacteria|Chloroflexi|Gitt-GS-136	0.047417	0	0.487168	0	0.256929	0.043181	0.38335	0	0.087462	0	0.162273	0	0	0	0	0	0	0	0	0	0	0	0	0	0	0	0	0	0	0		
Bacteria|Chloroflexi|Ktedonobacteria	0.047417	0.348218	5.624571	0	0	0.086361	21.29724	0	0.043731	0	39.6758	0	0	0	0	0	0	0	0	0	0.055011	0.049731	0.046223	0.157119	0.05303	0.044217	0	0	0	0	Taiga permafrost layer	8.28E-05
Bacteria|Chloroflexi|Ktedonobacteria|B12-WMSP1	0	0	0	0	0	0	0.766701	0	0	0	0.324546	0	0	0	0	0	0	0	0	0	0	0	0	0	0	0	0	0	0	0		
Bacteria|Chloroflexi|Ktedonobacteria|JG30-KF-AS9	0	0	0	0	0	0	0	0	0	0	0	0	0	0	0	0	0	0	0	0	0	0	0	0	0	0	0	0	0	0	China oil refineries	2.03E-09
Bacteria|Chloroflexi|Ktedonobacteria|Thermogemmatisporales	0.047417	0.348218	5.580283	0	0	0.086361	20.48794	0	0.043731	0	39.35125	0	0	0	0	0	0	0	0	0	0.055011	0.049731	0.046223	0.157119	0.05303	0.044217	0	0	0	0	Taiga permafrost layer	8.43E-05
Bacteria|Chloroflexi|Ktedonobacteria|Thermogemmatisporales|Thermogemmatisporaceae	0.047417	0.348218	5.580283	0	0	0.086361	20.48794	0	0.043731	0	39.35125	0	0	0	0	0	0	0	0	0	0.055011	0.049731	0.046223	0.157119	0.05303	0.044217	0	0	0	0	Taiga permafrost layer	8.43E-05
Bacteria|Chloroflexi|P2-11E	0	0	0.664319	0	0	0	0.127783	0	0.524774	0.043004	0.527388	0	0	0	0	0	0	0	0	0	0	0	0	0	0	0	0	0	0	0		
Bacteria|Chloroflexi|S085	0.379335	0	0.885759	0	0.102771	0	1.363023	0	0.524774	0.086009	1.054776	0	0	0	0	0	0	0	0	0	0	0	0	0	0	0	0	0	0	0	China oil refineries	8.69E-06
Bacteria|Chloroflexi|SHA-26	0	0	0.088576	0	0.102771	0.431806	0.937078	0	0	0	1.460459	0	0	0	0	0	0	0	0	0	0	0	0	0	0	0	0	0	0	0		
Bacteria|Chloroflexi|Thermomicrobia	0.094834	0	0	0	0.102771	0	0.085189	0	0	0	0.081137	0	0	0	0	0	0	0.201815	0.206586	0	0	0	0	0	0	0	0	0	0	0	China oil refineries	5.8E-05
Bacteria|Chloroflexi|Thermomicrobia|AKYG1722	0	0	0	0	0	0	0	0	0	0	0	0	0	0	0	0	0	0	0	0	0	0	0	0	0	0	0	0	0	0		
Bacteria|Chloroflexi|Thermomicrobia|JG30-KF-CM45	0.094834	0	0	0	0.102771	0	0	0	0	0	0.081137	0	0	0	0	0	0	0	0.206586	0	0	0	0	0	0	0	0	0	0	0	China oil refineries	3.13E-07
Bacteria|Chloroflexi|TK10	0.094834	0	0.088576	0	0	0	0.255567	0	0	0	0.162273	0	0	0	0	0	0	0	0	0	0	0	0	0	0	0	0	0	0	0	China oil refineries	6.06E-07
Bacteria|Chloroflexi|TK10|AKYG885	0.094834	0	0	0	0	0	0	0	0	0	0	0	0	0	0	0	0	0	0	0	0	0	0	0	0	0	0	0	0	0	China oil refineries	1.2E-08
Bacteria|Chloroflexi|TK10|AKYG885|Dolo_23	0.094834	0	0	0	0	0	0	0	0	0	0	0	0	0	0	0	0	0	0	0	0	0	0	0	0	0	0	0	0	0	China oil refineries	1.2E-08
Bacteria|Chloroflexi|TK10|B07_WMSP1	0	0	0	0	0	0	0.255567	0	0	0	0.081137	0	0	0	0	0	0	0	0	0	0	0	0	0	0	0	0	0	0	0		
Bacteria|Chloroflexi|TK17	0	0	0	0	0	0	0	0	0.087462	0	0	0	0	0	0	0	0	0	0	0	0	0	0	0	0	0	0	0	0	0	China oil refineries	5.31E-08
Bacteria|Chloroflexi|TK17|mle1-48	0	0	0	0	0	0	0	0	0.087462	0	0	0	0	0	0	0	0	0	0	0	0	0	0	0	0	0	0	0	0	0	China oil refineries	5.31E-08
Bacteria|Cyanobacteria	0.426752	0	0.265728	0	0	0	0	0	0	0	0	0	0.035133	0	0	0	0	0.050454	0.154939	0	0	0	0	0.104746	0	0.044217	0	0	0.213911	0	China oil refineries	2.62E-07
Bacteria|Cyanobacteria|4C0d-2	0.047417	0	0	0	0	0	0	0	0	0	0	0	0	0	0	0	0	0.050454	0.051646	0	0	0	0	0.104746	0	0	0	0	0	0		
Bacteria|Cyanobacteria|4C0d-2|MLE1-12	0	0	0	0	0	0	0	0	0	0	0	0	0	0	0	0	0	0.050454	0.051646	0	0	0	0	0.052373	0	0	0	0	0	0		
Bacteria|Cyanobacteria|4C0d-2|SM1D11	0.047417	0	0	0	0	0	0	0	0	0	0	0	0	0	0	0	0	0	0	0	0	0	0	0	0	0	0	0	0	0		
Bacteria|Cyanobacteria|Chloroplast	0.379335	0	0.265728	0	0	0	0	0	0	0	0	0	0	0	0	0	0	0	0	0	0	0	0	0	0	0	0	0	0	0	Mangrove	6.91E-08
Bacteria|Cyanobacteria|Chloroplast|Stramenopiles	0.189668	0	0	0	0	0	0	0	0	0	0	0	0	0	0	0	0	0	0	0	0	0	0	0	0	0	0	0	0	0		
Bacteria|Cyanobacteria|Chloroplast|Streptophyta	0	0	0.265728	0	0	0	0	0	0	0	0	0	0	0	0	0	0	0	0	0	0	0	0	0	0	0	0	0	0	0		
Bacteria|Cyanobacteria|ML635J-21	0	0	0	0	0	0	0	0	0	0	0	0	0	0	0	0	0	0	0	0	0	0	0	0	0	0	0	0	0	0		
Bacteria|Cyanobacteria|Oscillatoriophycideae	0	0	0	0	0	0	0	0	0	0	0	0	0	0	0	0	0	0	0	0	0	0	0	0	0	0.044217	0	0	0	0	China oil refineries	7.49E-09
Bacteria|Cyanobacteria|Oscillatoriophycideae|Chroococcales	0	0	0	0	0	0	0	0	0	0	0	0	0	0	0	0	0	0	0	0	0	0	0	0	0	0	0	0	0	0		
Bacteria|Cyanobacteria|Oscillatoriophycideae|Oscillatoriales	0	0	0	0	0	0	0	0	0	0	0	0	0	0	0	0	0	0	0	0	0	0	0	0	0	0.044217	0	0	0	0		
Bacteria|Cyanobacteria|Oscillatoriophycideae|Oscillatoriales|Phormidiaceae	0	0	0	0	0	0	0	0	0	0	0	0	0	0	0	0	0	0	0	0	0	0	0	0	0	0.044217	0	0	0	0		
Bacteria|Cyanobacteria|Oscillatoriophycideae|Oscillatoriales|Phormidiaceae|Phormidium	0	0	0	0	0	0	0	0	0	0	0	0	0	0	0	0	0	0	0	0	0	0	0	0	0	0	0	0	0	0		
Bacteria|Cyanobacteria|Synechococcophycideae	0	0	0	0	0	0	0	0	0	0	0	0	0	0	0	0	0	0	0	0	0	0	0	0	0	0	0	0	0.213911	0	China oil refineries	1.08E-07
Bacteria|Cyanobacteria|Synechococcophycideae|Pseudanabaenales	0	0	0	0	0	0	0	0	0	0	0	0	0	0	0	0	0	0	0	0	0	0	0	0	0	0	0	0	0	0		
Bacteria|Cyanobacteria|Synechococcophycideae|Pseudanabaenales|Pseudanabaenaceae	0	0	0	0	0	0	0	0	0	0	0	0	0	0	0	0	0	0	0	0	0	0	0	0	0	0	0	0	0	0		
Bacteria|Cyanobacteria|Synechococcophycideae|Pseudanabaenales|Pseudanabaenaceae|Leptolyngbya	0	0	0	0	0	0	0	0	0	0	0	0	0	0	0	0	0	0	0	0	0	0	0	0	0	0	0	0	0	0		
Bacteria|Deferribacteres	0	0	0	0	0	0	0	0	0	0	0	0	0	0	0.280788	0	0	0	0	0.049788	0.055011	1.34275	0.323561	0.052373	0.212119	0.088435	0	0	0.213911	0.045072		
Bacteria|Deferribacteres|Deferribacteres	0	0	0	0	0	0	0	0	0	0	0	0	0	0	0.280788	0	0	0	0	0.049788	0.055011	1.34275	0.323561	0.052373	0.212119	0.088435	0	0	0.213911	0.045072		
Bacteria|Deferribacteres|Deferribacteres|Deferribacterales	0	0	0	0	0	0	0	0	0	0	0	0	0	0	0.280788	0	0	0	0	0.049788	0.055011	1.34275	0.323561	0.052373	0.212119	0.088435	0	0	0.213911	0.045072		
Bacteria|Deferribacteres|Deferribacteres|Deferribacterales|Deferribacteraceae	0	0	0	0	0	0	0	0	0	0	0	0	0	0	0.280788	0	0	0	0	0.049788	0.055011	1.34275	0.323561	0.052373	0.212119	0.088435	0	0	0.213911	0.045072		
Bacteria|Deferribacteres|Deferribacteres|Deferribacterales|Deferribacteraceae|Deferribacter	0	0	0	0	0	0	0	0	0	0	0	0	0	0	0	0	0	0	0	0.049788	0.055011	1.143824	0.092446	0.052373	0.05303	0.088435	0	0	0.213911	0.045072		
Bacteria|Elusimicrobia	0.094834	0	0	0	0	0	0	0	0	0	0.081137	0	0	0	0	0	0	0	0	0	0	0.099463	1.109353	0	0	0.176869	0	0	0	0.180289		
Bacteria|Elusimicrobia|Elusimicrobia	0.094834	0	0	0	0	0	0	0	0	0	0.081137	0	0	0	0	0	0	0	0	0	0	0.099463	1.109353	0	0	0.176869	0	0	0	0.180289		
Bacteria|Elusimicrobia|Elusimicrobia|Elusimicrobiales	0.094834	0	0	0	0	0	0	0	0	0	0.081137	0	0	0	0	0	0	0	0	0	0	0.099463	1.109353	0	0	0.176869	0	0	0	0.180289		
Bacteria|Elusimicrobia|Elusimicrobia|FAC88	0	0	0	0	0	0	0	0	0	0	0	0	0	0	0	0	0	0	0	0	0	0	0	0	0	0	0	0	0	0		
Bacteria|EM3	0	0	0	0	0	0	0	0	0	0	0	0	0	0	0	0	0	0	0	0	0	0.149194	2.218705	0	0.05303	0	0	0	0	0		
Bacteria|Firmicutes	0.109059	0	0.415569	0.586944	0.006851	0.070049	0.520126	0.219459	1.087935	0.093654	0.575007	0.705324	0.842851	0.146959	3.716827	0.376932	0.310242	0.480551	0.29586	3.385611	2.073914	14.81825	27.11091	2.586471	2.793147	5.519164	0.418525	0.27067	1.393989	4.859936	Oil sands tailings pond upper	7.06E-07
Bacteria|Firmicutes|Bacilli	0	0	0.182319	0.503521	0	0.070049	0.135356	0.172985	0.036929	0.093654	0.399211	0.544132	0.668944	0.126281	2.403543	0.325995	0.198352	0.359463	0.29586	0	0	0.110514	0.06009	0	0.047727	0.419433	0	0	0.187172	0.581504	Oil sands core	0.000453
Bacteria|Firmicutes|Bacilli|Bacillales	0	0	0.182319	0.503521	0	0.070049	0.118318	0.172985	0.036929	0.093654	0.399211	0.544132	0.647865	0.084926	0.769358	0.025468	0.127149	0.097103	0.073781	0	0	0.011051	0.023112	0	0.047727	0.101068	0	0	0.187172	0.157825	China oil refineries	0.000321
Bacteria|Firmicutes|Bacilli|Lactobacillales	0	0	0	0	0	0	0.017038	0	0	0	0	0	0.02108	0.041355	1.634184	0.300527	0.071203	0.262359	0.22208	0	0	0.099463	0.036978	0	0	0.318365	0	0	0	0.423679		
Bacteria|Firmicutes|Bacilli|Lactobacillales|Carnobacteriaceae	0	0	0	0	0	0	0	0	0	0	0	0	0	0	1.432017	0.076405	0.050859	0.201815	0.180762	0	0	0.099463	0	0	0	0	0	0	0	0.022536		
Bacteria|Firmicutes|Bacilli|Lactobacillales|Carnobacteriaceae|Trichococcus	0	0	0	0	0	0	0	0	0	0	0	0	0	0	1.403939	0	0	0.201815	0.180762	0	0	0	0	0	0	0	0	0	0	0.022536		
Bacteria|Firmicutes|Clostridia	0.109059	0	0.23325	0.083424	0.006851	0	0.299581	0.046474	0.679291	0	0.155512	0.134327	0.042159	0.020678	1.17289	0.050937	0.111891	0.121089	0	3.286034	1.963892	12.37035	24.48544	2.167488	1.97649	4.768101	0.280852	0.21308	1.1266	4.210824	Oil sands tailings pond upper	1.76E-06
Bacteria|Firmicutes|Clostridia|Clostridiales	0.109059	0	0.23325	0.083424	0.006851	0	0.278284	0.046474	0.679291	0	0.155512	0.134327	0.042159	0	1.088654	0.050937	0.061031	0.121089	0	3.186458	1.936386	12.08025	23.71505	2.167488	1.870431	4.687036	0.280852	0.21308	1.1266	4.068095	Oil sands tailings pond upper	2.27E-06
Bacteria|Firmicutes|Clostridia|Clostridiales|Acidaminobacteraceae	0	0	0	0	0	0	0	0	0	0	0	0	0	0	0.044926	0	0	0	0	0.019915	0	0.377959	1.275756	0	0.042424	0.053061	0	0	0	0.090145		
Bacteria|Firmicutes|Clostridia|Clostridiales|Acidaminobacteraceae|Acidaminobacter	0	0	0	0	0	0	0	0	0	0	0	0	0	0	0	0	0	0	0	0.019915	0	0.218819	1.238777	0	0.042424	0.053061	0	0	0	0.090145		
Bacteria|Firmicutes|Clostridia|Clostridiales|Acidaminobacteraceae|Fusibacter	0	0	0	0	0	0	0	0	0	0	0	0	0	0	0.044926	0	0	0	0	0	0	0.159141	0.036978	0	0	0	0	0	0	0		
Bacteria|Firmicutes|Clostridia|Clostridiales|Clostridiaceae	0.061642	0	0.056098	0.041712	0.006851	0	0.218652	0.046474	0.043731	0	0.119	0	0	0	0.162857	0	0	0.040363	0	0.069704	0.19804	1.236656	1.491023	0.104746	0.248482	0.421538	0.044055	0.046071	0.021391	0.287175		
Bacteria|Firmicutes|Clostridia|Clostridiales|Clostridiaceae|Clostridium	0.061642	0	0.056098	0.041712	0.006851	0	0.218652	0.046474	0.043731	0	0.086546	0	0	0	0	0	0	0	0	0.049788	0.110022	1.057623	1.06313	0	0.212119	0.050113	0	0	0	0.135217		
Bacteria|Firmicutes|Clostridia|Clostridiales|Eubacteriaceae	0	0	0	0	0	0	0	0	0	0	0	0	0	0	0.494186	0	0.040688	0	0	0	0	0.437637	1.701007	0.104746	0.148483	0	0	0	0.021391	0.036058		
Bacteria|Firmicutes|Clostridia|Clostridiales|Eubacteriaceae|Acetobacterium	0	0	0	0	0	0	0	0	0	0	0	0	0	0	0.494186	0	0.040688	0	0	0	0	0.437637	1.682518	0.104746	0.148483	0	0	0	0.021391	0.036058		
Bacteria|Firmicutes|Clostridia|Clostridiales|Mogibacteriaceae	0	0	0	0	0	0	0	0	0	0	0	0	0	0	0.016045	0	0	0	0	0	0	0.156299	0.924461	0.014964	0.121211	0	0	0	0	0.012878		
Bacteria|Firmicutes|Clostridia|Clostridiales|Mogibacteriaceae|Anaerovorax	0	0	0	0	0	0	0	0	0	0	0	0	0	0	0	0	0	0	0	0	0	0.14209	0.911254	0.014964	0.090908	0	0	0	0	0		
Bacteria|Firmicutes|Clostridia|Clostridiales|Peptococcaceae	0	0	0	0	0	0	0.017038	0	0	0	0.016227	0	0	0	0.078621	0	0	0	0	2.758277	1.320263	6.514825	10.61281	1.277898	0.795445	3.059839	0.055069	0.057589	1.026775	3.037871	Oil sands tailings pond upper	3.96E-08
Bacteria|Firmicutes|Clostridia|Clostridiales|Peptococcaceae|Desulfosporosinus	0	0	0	0	0	0	0.017038	0	0	0	0.016227	0	0	0	0.022463	0	0	0	0	0	0	0.099463	0.517698	0.020949	0	0.035374	0	0	0.064173	0		
Bacteria|Firmicutes|Clostridia|Clostridiales|Peptococcaceae|Desulfosporosinus|meridiei	0	0	0	0	0	0	0.017038	0	0	0	0.016227	0	0	0	0.022463	0	0	0	0	0	0	0.099463	0.517698	0.020949	0	0.035374	0	0	0.064173	0		
Bacteria|Firmicutes|Clostridia|Clostridiales|Peptococcaceae|Desulfotomaculum_Desulfovirgula	0	0	0	0	0	0	0	0	0	0	0	0	0	0	0.056158	0	0	0	0	0.049788	0.055011	5.768853	9.383275	0.36661	0.265148	0.707477	0	0	0.320867	0.360578		
Bacteria|Firmicutes|Clostridia|Clostridiales|Peptococcaceae|Pelotomaculum	0	0	0	0	0	0	0	0	0	0	0	0	0	0	0	0	0	0	0	2.539208	1.10022	0.497315	0.369784	0.628474	0.265148	1.989779	0.055069	0.057589	0.641734	2.433903	Oil sands tailings pond upper	3.45E-08
Bacteria|Firmicutes|Clostridia|Clostridiales|Peptostreptococcaceae	0	0	0	0	0	0	0	0	0	0	0	0	0	0	0	0	0	0	0	0	0	6.008994	0	0	0.106059	0	0	0	0	0		
Bacteria|Firmicutes|Clostridia|Clostridiales|Tissierellaceae	0	0	0.177152	0.041712	0	0	0.042594	0	0	0	0	0	0	0	0.078621	0.050937	0.020344	0	0	0.019915	0.022004	2.6855	1.035396	0.083797	0.381813	0.141495	0.022028	0.023036	0.021391	0.16226		
Bacteria|Firmicutes|Clostridia|Clostridiales|Tissierellaceae|Tissierella_Soehngenia	0	0	0	0	0	0	0	0	0	0	0	0	0	0	0	0	0.020344	0	0	0.019915	0.022004	2.52636	0.758058	0.083797	0.33939	0.141495	0.022028	0.023036	0.021391	0.16226		
Bacteria|Firmicutes|Clostridia|Thermoanaerobacterales	0	0	0	0	0	0	0	0	0	0	0	0	0	0	0.028079	0	0.050859	0	0	0.099577	0.027505	0.165772	0.701049	0	0.05303	0.081065	0	0	0	0.142729		
Bacteria|Firmicutes|Erysipelotrichi	0	0	0	0	0	0	0	0	0.34985	0	0.020284	0	0	0	0	0	0	0	0	0.024894	0.055011	1.34275	0.970684	0.130932	0.344693	0.154761	0.110138	0	0	0		
Bacteria|Firmicutes|Erysipelotrichi|Erysipelotrichales	0	0	0	0	0	0	0	0	0.34985	0	0.020284	0	0	0	0	0	0	0	0	0.024894	0.055011	1.34275	0.970684	0.130932	0.344693	0.154761	0.110138	0	0	0		
Bacteria|Firmicutes|Erysipelotrichi|Erysipelotrichales|Erysipelotrichaceae	0	0	0	0	0	0	0	0	0.34985	0	0.020284	0	0	0	0	0	0	0	0	0.024894	0.055011	1.34275	0.970684	0.130932	0.344693	0.154761	0.110138	0	0	0		
Bacteria|Firmicutes|Erysipelotrichi|Erysipelotrichales|Erysipelotrichaceae|PSB-M-3	0	0	0	0	0	0	0	0	0	0	0	0	0	0	0	0	0	0	0	0.024894	0	1.317884	0.924461	0.130932	0.344693	0.154761	0.110138	0	0	0		
Bacteria|Gemmatimonadetes	0.474169	0	0.44288	0	0.205543	0.259084	1.022267	0	0	0.172018	1.460459	0	0	0	0	0	0	0	0	0	0	0.099463	0	0	0	0	0	0	0	0	China oil refineries	7.19E-07
Bacteria|Gemmatimonadetes|Gemm-1	0.094834	0	0	0	0	0	0	0	0	0	0.24341	0	0	0	0	0	0	0	0	0	0	0	0	0	0	0	0	0	0	0		
Bacteria|Gemmatimonadetes|Gemm-2	0	0	0	0	0	0	0	0	0	0	0	0	0	0	0	0	0	0	0	0	0	0.099463	0	0	0	0	0	0	0	0		
Bacteria|Gemmatimonadetes|Gemm-3	0	0	0	0	0	0	0	0	0	0	0	0	0	0	0	0	0	0	0	0	0	0	0	0	0	0	0	0	0	0	China oil refineries	2.03E-09
Bacteria|Gemmatimonadetes|Gemm-4	0	0	0	0	0	0	0	0	0	0	0	0	0	0	0	0	0	0	0	0	0	0	0	0	0	0	0	0	0	0		
Bacteria|Gemmatimonadetes|Gemm-5	0	0	0	0	0	0	0	0	0	0	0	0	0	0	0	0	0	0	0	0	0	0	0	0	0	0	0	0	0	0	China oil refineries	3.32E-08
Bacteria|Gemmatimonadetes|Gemmatimonadetes	0.379335	0	0.44288	0	0.205543	0.259084	1.022267	0	0	0.086009	1.135913	0	0	0	0	0	0	0	0	0	0	0	0	0	0	0	0	0	0	0	Arctic	5.58E-05
Bacteria|Gemmatimonadetes|Gemmatimonadetes|Gemmatimonadales	0.189668	0	0	0	0	0	0	0	0	0.086009	0	0	0	0	0	0	0	0	0	0	0	0	0	0	0	0	0	0	0	0	Arctic	2.94E-05
Bacteria|Gemmatimonadetes|Gemmatimonadetes|Gemmatimonadales|Gemmatimonadaceae	0.189668	0	0	0	0	0	0	0	0	0	0	0	0	0	0	0	0	0	0	0	0	0	0	0	0	0	0	0	0	0		
Bacteria|Gemmatimonadetes|Gemmatimonadetes|Gemmatimonadales|Gemmatimonadaceae|Gemmatimonas	0.189668	0	0	0	0	0	0	0	0	0	0	0	0	0	0	0	0	0	0	0	0	0	0	0	0	0	0	0	0	0		
Bacteria|Gemmatimonadetes|Gemmatimonadetes|KD8-87	0	0	0	0	0	0	0	0	0	0	0	0	0	0	0	0	0	0	0	0	0	0	0	0	0	0	0	0	0	0		
Bacteria|Gemmatimonadetes|Gemmatimonadetes|N1423WL	0.094834	0	0.44288	0	0.205543	0.259084	1.022267	0	0	0	1.135913	0	0	0	0	0	0	0	0	0	0	0	0	0	0	0	0	0	0	0		
Bacteria|GN02	0	0	0	0	0	0	0	0	0	0	0	0	0	0	0	0	0	0	0	0	0	0	0	0	0	0	0	0	0	0		
Bacteria|GN02|BD1-5	0	0	0	0	0	0	0	0	0	0	0	0	0	0	0	0	0	0	0	0	0	0	0	0	0	0	0	0	0	0		
Bacteria|GN04	0	0	0	0	0	0	0	0	0	0	0	0	0	0	0	0	0	0	0	0	0	0	0	0	0	0	0	0	0	0	Mangrove	5.7E-08
Bacteria|GN04|GN15	0	0	0	0	0	0	0	0	0	0	0	0	0	0	0	0	0	0	0	0	0	0	0	0	0	0	0	0	0	0	Mangrove	5.7E-08
Bacteria|H-178	0	0	0	0	0	0	0	0	0	0	0	0	0	0	0	0	0	0	0	0	0	0.497315	2.680936	0	0.106059	0	0	0	0	0		
Bacteria|Nitrospirae	0	0.099491	0.088576	0	0	0.690889	0.425945	0	0	0	0.324546	0	0	0	0	0	0	0	0	0	0.110022	0.596778	0.277338	0.314237	1.696948	1.768693	0	0	0	0.270434	Oil sands tailings pond median	0.007361
Bacteria|Nitrospirae|Nitrospira	0	0.099491	0.088576	0	0	0.690889	0.425945	0	0	0	0.324546	0	0	0	0	0	0	0	0	0	0.110022	0.596778	0.277338	0.314237	1.696948	1.768693	0	0	0	0.270434	Oil sands tailings pond median	0.007361

Table S2. III

Bacterial clades	Habitat																												
	India oil refineries				Arctic												China oil refineries									Mangrove			Marine sediments
	I1	I2	I3	I4	A1	A10	A11	A12	A2	A3	A4	A5	A6	A7	A8	A9	C1	C2	C3	C4	C5	C6	C7	C8	C9	M1	M2	M3	DWH1
Bacteria|Nitrospirae|Nitrospira|Nitrospirales	0	0	0.17384	0.170653	0.558789	0.511047	0	0	0.324453	1.128287	0.184067	0	0	1.029664	0.586101	0	0.376213	0.799588	0.337869	1.047366	1.146846	0.085284	0.56088	0.138359	0.252354	0.23403	0.325088	0.236859	1.188171
Bacteria|Nitrospirae|Nitrospira|Nitrospirales|FW	0	0	0.08692	0.170653	0	0	0	0	0	0	0	0	0	0	0.167457	0	0	0	0	0	0	0	0	0	0	0	0	0	0
Bacteria|Nitrospirae|Nitrospira|Nitrospirales|FW|4-29	0	0	0.08692	0.170653	0	0	0	0	0	0	0	0	0	0	0.167457	0	0	0	0	0	0	0	0	0	0	0	0	0	0
Bacteria|Nitrospirae|Nitrospira|Nitrospirales|Nitrospiraceae	0	0	0	0	0.372526	0.425873	0	0	0.324453	1.128287	0.184067	0	0	1.029664	0.251186	0	0.094053	0.079959	0.084467	0.299247	0.501745	0	0.080126	0.069179	0.168236	0	0.325088	0.078953	0.913977
Bacteria|Nitrospirae|Nitrospira|Nitrospirales|Nitrospiraceae|Nitrospira	0	0	0	0	0.372526	0.425873	0	0	0.324453	1.034263	0.184067	0	0	1.029664	0.251186	0	0.094053	0	0	0.074812	0.286712	0	0	0.069179	0.084118	0	0	0	0
Bacteria|Nitrospirae|Nitrospira|Nitrospirales|Thermodesulfovibrionaceae	0	0	0.08692	0	0.186263	0.085175	0	0	0	0	0	0	0	0	0.083729	0	0	0	0	0	0.071678	0	0.080126	0.069179	0	0.15602	0	0.078953	0.274193
Bacteria|Nitrospirae|Nitrospira|Nitrospirales|Thermodesulfovibrionaceae|GOUTA19	0	0	0	0	0	0	0	0	0	0	0	0	0	0	0	0	0	0	0	0	0	0	0	0	0	0	0	0	0
Bacteria|Nitrospirae|Nitrospira|Nitrospirales|Thermodesulfovibrionaceae|LCP-6	0	0	0	0	0	0	0	0	0	0	0	0	0	0	0	0	0	0	0	0	0	0	0	0	0	0.15602	0	0	0
Bacteria|OD1	0	0.116195	0	0	0.046566	0	0.049093	0	0	0.047012	0	0	0	0	0.125593	0	0	0.079959	0	0.037406	0	0.085284	0	0.207538	0.126177	2.223285	0.60954	0.986912	0.137097
Bacteria|OD1|ZB2	0	0	0	0	0.046566	0	0.049093	0	0	0.047012	0	0	0	0	0.125593	0	0	0.039979	0	0	0	0.085284	0	0.138359	0.126177	2.067265	0.60954	0.986912	0.091398
Bacteria|OP1	0	0	0	0	0	0	0	0	0	0	0	0	0	0	0	0	0	0	0	0	0	0	0	0	0	0.026003	0.027091	0.026318	0
Bacteria|OP1|OPB14	0	0	0	0	0	0	0	0	0	0	0	0	0	0	0	0	0	0	0	0	0	0	0	0	0	0	0	0	0
Bacteria|OP11	0	0	0	0	0	0	0	0	0	0	0	0	0	0	0	0	0.047027	0	0	0	0	0	0	0.03459	0	0.07801	0.121908	0	0
Bacteria|OP11|OP11-2	0	0	0	0	0	0	0	0	0	0	0	0	0	0	0	0	0	0	0	0	0	0	0	0	0	0	0	0	0
Bacteria|OP11|OP11-2|KD3-145	0	0	0	0	0	0	0	0	0	0	0	0	0	0	0	0	0	0	0	0	0	0	0	0	0	0	0	0	0
Bacteria|OP8	0	0.038732	0	0	0	0	0	0	0	0	0	0	0	0	0	0	0	0	0.042234	0	0	0	0	0	0	1.79423	1.056535	1.460629	0
Bacteria|OP8|OP8_1	0	0.038732	0	0	0	0	0	0	0	0	0	0	0	0	0	0	0	0	0	0	0	0	0	0	0	1.09214	0.325088	0.434241	0
Bacteria|OP8|OP8_1|HMMVPog-54	0	0	0	0	0	0	0	0	0	0	0	0	0	0	0	0	0	0	0	0	0	0	0	0	0	1.01413	0.284452	0.434241	0
Bacteria|OP8|OP8_1|OPB95	0	0.038732	0	0	0	0	0	0	0	0	0	0	0	0	0	0	0	0	0	0	0	0	0	0	0	0.07801	0	0	0
Bacteria|OP8|SAW1_B6	0	0	0	0	0	0	0	0	0	0	0	0	0	0	0	0	0	0	0	0	0	0	0	0	0	0.62408	0.487632	0.789529	0
Bacteria|Planctomycetes	0.071151	0	0.04346	0.127989	0.046566	0.042587	0.098186	0	0.081113	0.282072	0.506185	0.215659	0	0.046803	0.251186	0.083149	2.774574	6.796494	3.843254	10.95994	8.171281	3.070236	6.490184	7.298426	2.691771	4.05652	4.510593	5.92147	6.077949
Bacteria|Planctomycetes|BD7-11	0	0	0	0	0	0	0	0	0	0	0	0	0	0	0	0	0.094053	0.079959	0.084467	0.261841	0.107517	0.042642	0	0.207538	0.042059	0.039005	0.040636	0.039476	0
Bacteria|Planctomycetes|Brocadiae	0	0	0	0	0	0	0	0	0	0	0	0	0	0	0	0	0	0	0	0	0	0	0	0	0	0	0	0	1.005375
Bacteria|Planctomycetes|Brocadiae|Brocadiales	0	0	0	0	0	0	0	0	0	0	0	0	0	0	0	0	0	0	0	0	0	0	0	0	0	0	0	0	1.005375
Bacteria|Planctomycetes|Brocadiae|Brocadiales|Brocadiaceae	0	0	0	0	0	0	0	0	0	0	0	0	0	0	0	0	0	0	0	0	0	0	0	0	0	0	0	0	1.005375
Bacteria|Planctomycetes|Brocadiae|Brocadiales|Brocadiaceae|Candidatus_Scalindua	0	0	0	0	0	0	0	0	0	0	0	0	0	0	0	0	0	0	0	0	0	0	0	0	0	0	0	0	1.005375
Bacteria|Planctomycetes|C6	0	0	0	0	0	0	0	0	0	0	0.046017	0	0	0	0	0	0	0.039979	0	0	0.035839	0	0	0	0	0.93612	0.528268	1.144818	0.091398
Bacteria|Planctomycetes|C6|d113	0	0	0	0	0	0	0	0	0	0	0.046017	0	0	0	0	0	0	0	0	0	0	0	0	0	0	0.54607	0.365724	0.513194	0.045699
Bacteria|Planctomycetes|OM190	0	0	0	0	0	0	0	0	0	0	0	0	0	0	0	0	0	0.279856	0.168934	0.897742	0.143356	0	0.040063	0	0	0.351045	0.528268	0.829006	0.228494
Bacteria|Planctomycetes|OM190|agg27	0	0	0	0	0	0	0	0	0	0	0	0	0	0	0	0	0	0.279856	0.084467	0.710713	0.107517	0	0.040063	0	0	0.15602	0.162544	0.394765	0.091398
Bacteria|Planctomycetes|OM190|CL500-15	0	0	0	0	0	0	0	0	0	0	0	0	0	0	0	0	0	0	0.084467	0.18703	0.035839	0	0	0	0	0.195025	0.365724	0.434241	0.137097
Bacteria|Planctomycetes|Phycisphaerae	0	0	0	0.085326	0	0	0	0	0	0.141036	0	0.129396	0	0	0.083729	0	0.893507	2.238845	1.055839	1.982514	2.222015	0.895485	2.604086	2.628817	0.841178	1.01413	1.178443	1.460629	1.553761
Bacteria|Planctomycetes|Phycisphaerae|MSBL9	0	0	0	0.042663	0	0	0	0	0	0	0	0	0	0	0	0	0	0	0.042234	0.074812	0.035839	0.085284	0.040063	0	0	0.195025	0.081272	0.513194	0
Bacteria|Planctomycetes|Phycisphaerae|Phycisphaerales	0	0	0	0	0	0	0	0	0	0	0	0	0	0	0	0	0.047027	0.399794	0.042234	0.935148	0.107517	0	0	0.103769	0	0.507065	0.731448	0.552671	0.731182
Bacteria|Planctomycetes|Phycisphaerae|Phycisphaerales|Phycisphaeraceae	0	0	0	0	0	0	0	0	0	0	0	0	0	0	0	0	0.047027	0.399794	0.042234	0.935148	0.107517	0	0	0.103769	0	0.507065	0.731448	0.552671	0.731182
Bacteria|Planctomycetes|Pla3	0	0	0	0	0	0	0	0	0	0	0	0	0	0	0	0	0	0.039979	0	0.074812	0	0	0.040063	0	0	0.07801	0.081272	0.039476	0.137097
Bacteria|Planctomycetes|Planctomycetia	0.071151	0	0.04346	0.042663	0.046566	0.042587	0.098186	0	0.040557	0.141036	0.414151	0.086264	0	0.046803	0.167457	0.083149	1.787014	4.117876	2.534014	7.743027	5.662554	2.132108	3.805972	4.392892	1.766475	1.365175	2.072435	1.934347	3.061824
Bacteria|Planctomycetes|Planctomycetia|Gemmatales	0.035575	0	0	0	0	0	0.098186	0	0.040557	0	0	0	0	0	0.041864	0	1.175667	0.599691	1.90051	0.972554	1.899464	0.852843	1.68264	0.691794	0.336471	0	0	0	0
Bacteria|Planctomycetes|Planctomycetia|Gemmatales|Gemmataceae	0	0	0	0	0	0	0	0	0	0	0	0	0	0	0.041864	0	0.799454	0.479753	1.055839	0.673307	1.397719	0.511706	1.282012	0.518845	0.210295	0	0	0	0
Bacteria|Planctomycetes|Planctomycetia|Gemmatales|Gemmataceae|Gemmata	0	0	0	0	0	0	0	0	0	0	0	0	0	0	0.041864	0	0.42324	0.199897	0.59127	0.299247	0.752618	0.127926	0.400629	0.345897	0.084118	0	0	0	0
Bacteria|Planctomycetes|Planctomycetia|Gemmatales|Isosphaeraceae	0.035575	0	0	0	0	0	0.098186	0	0.040557	0	0	0	0	0	0	0	0.376213	0.119938	0.844671	0.299247	0.501745	0.341137	0.400629	0.172948	0.126177	0	0	0	0
Bacteria|Planctomycetes|Planctomycetia|Pirellulales	0	0	0.04346	0	0.046566	0.042587	0	0	0	0.141036	0.414151	0.086264	0	0.046803	0.125593	0.083149	0.329187	2.238845	0.464569	3.703187	3.010472	1.151338	1.442263	2.628817	0.967355	0.7801	1.097171	1.263247	1.827955
Bacteria|Planctomycetes|Planctomycetia|Pirellulales|Pirellulaceae	0	0	0.04346	0	0.046566	0.042587	0	0	0	0.141036	0.414151	0.086264	0	0.046803	0.125593	0.083149	0.329187	2.238845	0.464569	3.703187	3.010472	1.151338	1.442263	2.628817	0.967355	0.7801	1.097171	1.263247	1.827955
Bacteria|Planctomycetes|Planctomycetia|Planctomycetales	0.035575	0	0	0.042663	0	0	0	0	0	0	0	0	0	0	0	0	0.28216	1.199381	0.126701	2.955068	0.645101	0.127926	0.600943	1.037691	0.462648	0.585075	0.975263	0.6711	1.233869
Bacteria|Planctomycetes|Planctomycetia|Planctomycetales|Planctomycetaceae	0.035575	0	0	0.042663	0	0	0	0	0	0	0	0	0	0	0	0	0.28216	1.199381	0.126701	2.955068	0.645101	0.127926	0.600943	1.037691	0.462648	0.585075	0.975263	0.6711	1.233869
Bacteria|Planctomycetes|Planctomycetia|Planctomycetales|Planctomycetaceae|Planctomyces	0.035575	0	0	0.042663	0	0	0	0	0	0	0	0	0	0	0	0	0.28216	1.199381	0.126701	2.955068	0.645101	0.127926	0.600943	1.037691	0.462648	0.585075	0.975263	0.6711	1.233869
Bacteria|Proteobacteria	49.30162	56.64635	50.00352	70.14392	37.53201	47.61258	42.01538	39.68818	27.80836	23.71753	32.71028	34.39047	16.1226	24.25171	32.88445	39.03825	18.80843	17.91076	16.31201	15.50796	16.23345	45.96493	15.30478	13.43233	28.31687	28.38979	37.02701	29.6488	42.80765
Bacteria|Proteobacteria|Alphaproteobacteria	16.64337	8.404772	3.1436	9.129915	19.88359	11.76828	17.07616	19.73862	13.11332	13.35923	16.94185	14.07537	10.87302	10.22644	13.78035	12.04268	8.206156	5.470511	3.744709	6.620849	7.466448	6.782947	5.615479	6.27803	5.194277	4.13453	7.915887	3.889748	8.605097
Bacteria|Proteobacteria|Alphaproteobacteria|BD7-3	0.071151	0	0.08692	0	0	0	0	0.285492	0	0	0	0	0	0	0.083729	0	0	0	0.084467	0.074812	0	0	0	0	0	0.15602	0.162544	0.157906	0.274193
Bacteria|Proteobacteria|Alphaproteobacteria|Caulobacterales	0.332037	0.077463	0.231786	0.369747	1.831587	1.192444	5.040209	2.854922	1.595229	1.582735	1.44186	2.214102	1.068227	2.776973	2.707229	2.217295	0.313511	0.159918	0.450491	0.523683	0.250873	0.397994	0.18696	0.472726	0.252354	0	0	0.052635	0
Bacteria|Proteobacteria|Alphaproteobacteria|Caulobacterales|Caulobacteraceae	0.332037	0.077463	0.231786	0.369747	1.831587	1.192444	5.040209	2.854922	1.595229	1.582735	1.44186	2.214102	1.068227	2.776973	2.707229	2.217295	0.313511	0.159918	0.450491	0.523683	0.250873	0.397994	0.18696	0.472726	0.252354	0	0	0.052635	0
Bacteria|Proteobacteria|Alphaproteobacteria|Caulobacterales|Caulobacteraceae|Arthrospira	0	0	0	0	0	0	0	0	0	0	0	0	0	0	0	0	0	0	0	0	0	0	0	0	0	0	0	0	0
Bacteria|Proteobacteria|Alphaproteobacteria|Caulobacterales|Caulobacteraceae|Arthrospira|fusiformis	0	0	0	0	0	0	0	0	0	0	0	0	0	0	0	0	0	0	0	0	0	0	0	0	0	0	0	0	0
Bacteria|Proteobacteria|Alphaproteobacteria|Caulobacterales|Caulobacteraceae|Caulobacter	0	0.025821	0.028973	0	0	0	1.701889	0	0	0	0.552202	0	0	0.592837	0.055819	0	0.062702	0	0.056311	0	0	0.056856	0.026709	0.02306	0	0	0	0	0
Bacteria|Proteobacteria|Alphaproteobacteria|Caulobacterales|Caulobacteraceae|Caulobacter|henricii	0	0	0	0	0	0	0.032729	0	0	0	0.552202	0	0	0	0.02791	0	0.062702	0	0.056311	0	0	0.056856	0.026709	0.02306	0	0	0	0	0
Bacteria|Proteobacteria|Alphaproteobacteria|Caulobacterales|Caulobacteraceae|Mycoplana	0	0	0	0	0	0	0	1.427461	0	0.047012	0	0	0	0	0	0	0.094053	0.079959	0.253401	0.374059	0.107517	0.085284	0.080126	0.311307	0.168236	0	0	0	0
Bacteria|Proteobacteria|Alphaproteobacteria|Caulobacterales|Caulobacteraceae|Phenylobacterium	0.332037	0.051642	0.202813	0.369747	1.800543	1.192444	3.338321	1.427461	1.433002	1.535723	0.889658	2.214102	1.037706	2.184136	2.651409	2.217295	0.156756	0.079959	0.140779	0.149624	0.143356	0.227425	0.080126	0.138359	0.084118	0	0	0.052635	0
Bacteria|Proteobacteria|Alphaproteobacteria|Ellin329	0.071151	0.038732	0.08692	0.127989	0	0	0	0	0	0	0	0.086264	0	0	0.167457	0	0.047027	0.039979	0	0	0	0.042642	0	0	0	0	0	0	0.137097
Bacteria|Proteobacteria|Alphaproteobacteria|Kiloniellales	0	0	0	0.042663	0	0	0	0	0	0	0	0	0	0	0	0	0	0	0	0	0.035839	0	0	0	0	0.39005	0.081272	0.473718	0
Bacteria|Proteobacteria|Alphaproteobacteria|Rhizobiales	2.300545	4.189475	1.383474	5.496437	12.922	7.46697	6.512998	10.62666	10.24732	8.767727	13.83572	7.878754	8.591597	5.975172	8.958973	5.654102	4.459697	1.872367	1.689343	2.581009	2.61027	2.10368	1.936372	1.568066	1.198679	1.989255	3.941689	1.500106	6.626336
Bacteria|Proteobacteria|Alphaproteobacteria|Rhizobiales|Beijerinckiaceae	0	0	0.028973	0	0	0	0	0	0	0	0	0	0	0	0	0	0.023513	0.039979	0	0.037406	0.017919	0.021321	0.160251	0.017295	0.063088	0	0.142226	0	0
Bacteria|Proteobacteria|Alphaproteobacteria|Rhizobiales|Bradyrhizobiaceae	0.373542	0.038732	0.10865	0.383968	1.396973	0.638809	0.270011	0.190328	1.419483	0.141036	1.01237	1.876236	1.419216	0.140409	2.051354	0.58204	0.42324	0.119938	0.253401	0.037406	0.143356	0.213211	0.160251	0.017295	0.063088	0.019503	0	0.098691	0
Bacteria|Proteobacteria|Alphaproteobacteria|Rhizobiales|Bradyrhizobiaceae|Bosea	0	0	0	0	0	0	0	0	0	0	0	0	0	0	0	0	0.094053	0	0	0	0	0.042642	0	0	0	0	0	0	0
Bacteria|Proteobacteria|Alphaproteobacteria|Rhizobiales|Bradyrhizobiaceae|Bosea|genosp.	0	0	0	0	0	0	0	0	0	0	0	0	0	0	0	0	0.094053	0	0	0	0	0.042642	0	0	0	0	0	0	0
Bacteria|Proteobacteria|Alphaproteobacteria|Rhizobiales|Bradyrhizobiaceae|Bradyrhizobium	0	0	0	0	0	0	0	0	0	0	0	0	0	0	0	0	0	0.01999	0.084467	0.037406	0.017919	0.021321	0.020031	0	0	0.019503	0	0.098691	0
Bacteria|Proteobacteria|Alphaproteobacteria|Rhizobiales|Bradyrhizobiaceae|Bradyrhizobium|elkanii	0	0	0	0	0	0	0	0	0	0	0	0	0	0	0	0	0	0.01999	0.084467	0.037406	0.017919	0.021321	0.020031	0	0	0	0	0	0
Bacteria|Proteobacteria|Alphaproteobacteria|Rhizobiales|Brucellaceae	0	0	0	0	0	0	0	0	0	0	0	0	0	0	0	0	0	0	0	0	0	0	0	0	0	0	0	0	0
Bacteria|Proteobacteria|Alphaproteobacteria|Rhizobiales|Brucellaceae|Ochrobactrum	0	0	0	0	0	0	0	0	0	0	0	0	0	0	0	0	0	0	0	0	0	0	0	0	0	0	0	0	0
Bacteria|Proteobacteria|Alphaproteobacteria|Rhizobiales|Brucellaceae|Ochrobactrum|intermedium	0	0	0	0	0	0	0	0	0	0	0	0	0	0	0	0	0	0	0	0	0	0	0	0	0	0	0	0	0
Bacteria|Proteobacteria|Alphaproteobacteria|Rhizobiales|Hyphomicrobiaceae	0.39133	0.135561	0.282489	3.946342	0.395809	0.695592	0.400926	0.729591	0.270378	0.258566	1.273132	0.043132	0.076302	1.482092	0.460508	0.020787	0.321349	0.493079	0.387141	0.561089	0.764564	0.504599	0.51414	0.351662	0.315442	0.019503	0.10159	0.019738	0.274193
Bacteria|Proteobacteria|Alphaproteobacteria|Rhizobiales|Hyphomicrobiaceae|Devosia	0	0	0	0	0	0.227132	0.2291	0.348935	0.027038	0	0.122711	0	0.030521	0.031202	0	0	0.062702	0.133265	0.112623	0.149624	0.119463	0.142141	0.053417	0.230598	0.084118	0	0	0	0
Bacteria|Proteobacteria|Alphaproteobacteria|Rhizobiales|Hyphomicrobiaceae|Hyphomicrobium	0	0.116195	0.04346	0.127989	0.372526	0.298111	0.098186	0.333074	0	0.094024	0.552202	0.043132	0.045781	0.982861	0.041864	0	0.047027	0.119938	0.084467	0.149624	0.107517	0.085284	0.160251	0.03459	0.042059	0	0	0	0.274193
Bacteria|Proteobacteria|Alphaproteobacteria|Rhizobiales|Hyphomicrobiaceae|Parvibaculum	0.355754	0	0.17384	3.754358	0	0	0	0	0	0	0	0	0	0	0	0	0.094053	0	0	0	0	0.085284	0	0	0	0	0	0	0
Bacteria|Proteobacteria|Alphaproteobacteria|Rhizobiales|Hyphomicrobiaceae|Rhodoplanes	0.035575	0.019366	0.06519	0.063995	0.023283	0.170349	0.073639	0.047582	0.24334	0.070518	0.414151	0	0	0.093606	0.293051	0.020787	0.117567	0.239876	0.190051	0.224436	0.501745	0.19189	0.300472	0.086474	0.189265	0.019503	0.10159	0.019738	0
Bacteria|Proteobacteria|Alphaproteobacteria|Rhizobiales|Methylobacteriaceae	0.053363	0	0.02173	0	0	0	0	0	0	0	0	0.021566	0	0	0	0.020787	2.327821	0.259866	0.105584	0.037406	0.035839	0.042642	0.060094	0.017295	0.042059	0	0	0.039476	0
Bacteria|Proteobacteria|Alphaproteobacteria|Rhizobiales|Methylobacteriaceae|Methylobacterium	0.053363	0	0.02173	0	0	0	0	0	0	0	0	0.021566	0	0	0	0.020787	1.716474	0.239876	0.06335	0.037406	0.017919	0.042642	0.020031	0	0.021029	0	0	0	0
Bacteria|Proteobacteria|Alphaproteobacteria|Rhizobiales|Methylobacteriaceae|Methylobacterium|adhaesivum	0	0	0	0	0	0	0	0	0	0	0	0	0	0	0	0	0.023513	0	0	0	0	0.021321	0	0	0	0	0	0	0
Bacteria|Proteobacteria|Alphaproteobacteria|Rhizobiales|Methylobacteriaceae|Methylobacterium|hispanicum	0	0	0	0	0	0	0	0	0	0	0	0	0	0	0	0	1.528367	0.239876	0.06335	0.037406	0	0.021321	0.020031	0	0.021029	0	0	0	0
Bacteria|Proteobacteria|Alphaproteobacteria|Rhizobiales|Methylocystaceae	0	0.051642	0.072433	0.056884	0	0	0	0	0	0	0	0	0	0	0	0	0.047027	0	0.042234	0.037406	0.035839	0.042642	0	0.069179	0.042059	0	0	0	0
Bacteria|Proteobacteria|Alphaproteobacteria|Rhizobiales|Methylocystaceae|Pleomorphomonas	0	0	0.04346	0	0	0	0	0	0	0	0	0	0	0	0	0	0.047027	0	0.042234	0.037406	0.035839	0.042642	0	0.069179	0.042059	0	0	0	0
Bacteria|Proteobacteria|Alphaproteobacteria|Rhizobiales|Methylocystaceae|Pleomorphomonas|oryzae	0	0	0.04346	0	0	0	0	0	0	0	0	0	0	0	0	0	0.047027	0	0.042234	0.037406	0.035839	0.042642	0	0.069179	0.042059	0	0	0	0
Bacteria|Proteobacteria|Alphaproteobacteria|Rhizobiales|Rhizobiaceae	0.39133	0.077463	0	0	0.465658	0	0.343651	0.428238	0.446123	0.470119	0.092034	0.69011	0.824061	0.046803	0.209322	0.498891	0.470267	0.239876	0.211168	0.037406	0.071678	0.213211	0.080126	0.207538	0.042059	0	0	0	0
Bacteria|Proteobacteria|Alphaproteobacteria|Rhizobiales|Rhizobiaceae|Kaistia	0.106726	0.077463	0	0	0	0	0	0	0	0.470119	0	0	0	0.046803	0	0	0.047027	0.039979	0	0	0.035839	0	0	0	0	0	0	0	0
Bacteria|Proteobacteria|Alphaproteobacteria|Rhizobiales|Rhodobiaceae	0	0	0	0	0	0	0	0	0	0	0	0	0	0	0	0	0	0.039979	0	0.037406	0.179195	0.042642	0.160251	0.03459	0	0.07801	0	0	0
Bacteria|Proteobacteria|Alphaproteobacteria|Rhizobiales|Rhodobiaceae|Afifella	0	0	0	0	0	0	0	0	0	0	0	0	0	0	0	0	0	0.039979	0	0.037406	0.179195	0.042642	0.160251	0.03459	0	0.07801	0	0	0
Bacteria|Proteobacteria|Alphaproteobacteria|Rhodobacterales	1.102838	0.096829	0.15211	0.191984	0.419092	0.340698	2.037357	1.141969	0.040557	0.587649	0.414151	0.021566	0	0.748847	0.020932	0	0.329187	0.579701	0.253401	0.542386	0.537584	0.315552	0.480754	0.449666	0.315442	1.053135	2.064307	0.797425	1.361826
Bacteria|Proteobacteria|Alphaproteobacteria|Rhodobacterales|Hyphomonadaceae	0	0	0	0	0.046566	0.042587	0	0.047582	0	0	0	0	0	0	0	0	0.047027	0.079959	0.042234	0.074812	0.143356	0.042642	0	0.03459	0	0.07801	0.325088	0.197382	0
Bacteria|Proteobacteria|Alphaproteobacteria|Rhodobacterales|Rhodobacteraceae	1.102838	0.096829	0.15211	0.191984	0.372526	0.298111	2.037357	1.094387	0.040557	0.587649	0.414151	0.021566	0	0.748847	0.020932	0	0.28216	0.499742	0.211168	0.467574	0.394228	0.27291	0.480754	0.415076	0.315442	0.975125	1.73922	0.600042	1.361826
Bacteria|Proteobacteria|Alphaproteobacteria|Rhodobacterales|Rhodobacteraceae|Amaricoccus	0	0	0	0	0.046566	0	1.865532	0.380656	0.040557	0.376096	0.276101	0	0	0.702044	0	0	0	0.079959	0.042234	0	0	0	0	0	0	0.039005	0.040636	0	0
Bacteria|Proteobacteria|Alphaproteobacteria|Rhodobacterales|Rhodobacteraceae|Octadecabacter	0	0	0	0	0	0	0	0	0	0	0	0	0	0	0	0	0	0	0	0	0	0	0	0	0	0.07801	0.243816	0	0
Bacteria|Proteobacteria|Alphaproteobacteria|Rhodobacterales|Rhodobacteraceae|Paracoccus	0	0	0	0	0	0.021294	0	0	0	0	0	0	0	0	0.020932	0	0.047027	0.039979	0.021117	0.074812	0.053758	0.021321	0.020031	0.086474	0.042059	0	0	0	0
Bacteria|Proteobacteria|Alphaproteobacteria|Rhodobacterales|Rhodobacteraceae|Paracoccus|marcusii	0	0	0	0	0	0.021294	0	0	0	0	0	0	0	0	0.020932	0	0.023513	0.039979	0.021117	0.056109	0	0	0.020031	0.017295	0.021029	0	0	0	0
Bacteria|Proteobacteria|Alphaproteobacteria|Rhodobacterales|Rhodobacteraceae|Phaeobacter	0	0	0	0	0	0	0	0	0	0	0	0	0	0	0	0	0	0	0	0	0	0.017057	0	0	0	0	0.032509	0.047372	0.676343
Bacteria|Proteobacteria|Alphaproteobacteria|Rhodospirillales	5.010207	3.033981	0.680872	1.663863	0.333721	0.085175	0.049093	0.071373	0.824652	0.11753	0.329787	0.726053	0.434921	0.023401	0.837287	2.466741	1.081614	0.419783	0.633503	0.654604	0.878054	0.59699	0.60762	0.294012	0.77809	0.31204	0.772083	0.315812	0.022849
Bacteria|Proteobacteria|Alphaproteobacteria|Rhodospirillales|Acetobacteraceae	3.249223	2.530469	0.398382	0.4053	0.333721	0.085175	0.049093	0.071373	0.784096	0.11753	0.329787	0.726053	0.366249	0.023401	0.355847	2.466741	0.517294	0.059969	0.105584	0.074812	0.053758	0.170569	0.247054	0.086474	0.231324	0.019503	0.020318	0.019738	0
Bacteria|Proteobacteria|Alphaproteobacteria|Rhodospirillales|Acetobacteraceae|Acidocella	0.972395	2.143152	0.028973	0	0.217307	0	0	0	0.054076	0	0.030678	0.143773	0	0	0	0.970067	0	0	0	0	0	0	0	0	0	0	0	0	0

Bacterial clades	Habitat																																				Differentially abundant class (one class test, non-strict)	p-value
	Marine sediments						Taiga upper active layer				Taiga bottom active layer				Taiga permafrost layer				Oil sands core							Oil sands tailings pond upper				Oil sands tailings pond median			Oil sands tailings pond deep					
	DWH2	DWH3	DWH4	DWH5	DWH6	DWH7	Tu1	Tu2	Tu3	Tu4	Tb1	Tb2	Tb3	Tb4	Tp1	Tp2	Tp3	Tp4	OSC1	OSC12	OSC3	OSC4	OSC5	OSC7	OSC9	OSTPu1	OSTPu2	OSTPu4	OSTPu3	OSTPm2	OSTPm6	OSTPm4	OSTPd1	OSTPd2	OSTPd3	OSTPd4		
Bacteria|Nitrospirae|Nitrospira|Nitrospirales	0	0.473237	0.190106	0.095472	0	0	0	0.099491	0.088576	0	0	0.690889	0.425945	0	0	0	0.324546	0	0	0	0	0	0	0	0	0	0.110022	0.596778	0.277338	0.314237	1.696948	1.768693	0	0	0	0.270434	Oil sands tailings pond median	0.007361
Bacteria|Nitrospirae|Nitrospira|Nitrospirales|FW	0	0	0	0	0	0	0	0	0	0	0	0.604528	0	0	0	0	0	0	0	0	0	0	0	0	0	0	0	0	0	0	0	0	0	0	0	0		
Bacteria|Nitrospirae|Nitrospira|Nitrospirales|FW|4-29	0	0	0	0	0	0	0	0	0	0	0	0.604528	0	0	0	0	0	0	0	0	0	0	0	0	0	0	0	0	0	0	0	0	0	0	0	0		
Bacteria|Nitrospirae|Nitrospira|Nitrospirales|Nitrospiraceae	0	0.37859	0.190106	0	0	0	0	0.099491	0.088576	0	0	0	0.170378	0	0	0	0.162273	0	0	0	0	0	0	0	0	0	0	0	0	0	0	0	0	0	0	0		
Bacteria|Nitrospirae|Nitrospira|Nitrospirales|Nitrospiraceae|Nitrospira	0	0.094647	0	0	0	0	0	0.099491	0	0	0	0	0	0	0	0	0	0	0	0	0	0	0	0	0	0	0	0	0	0	0	0	0	0	0	0		
Bacteria|Nitrospirae|Nitrospira|Nitrospirales|Thermodesulfovibrionaceae	0	0.094647	0	0.095472	0	0	0	0	0	0	0	0.086361	0	0	0	0	0	0	0	0	0	0	0	0	0	0	0.110022	0.596778	0.277338	0.314237	1.696948	1.768693	0	0	0	0.270434		
Bacteria|Nitrospirae|Nitrospira|Nitrospirales|Thermodesulfovibrionaceae|GOUTA19	0	0	0	0	0	0	0	0	0	0	0	0.086361	0	0	0	0	0	0	0	0	0	0	0	0	0	0	0	0.099463	0	0	1.166652	0	0	0	0	0		
Bacteria|Nitrospirae|Nitrospira|Nitrospirales|Thermodesulfovibrionaceae|LCP-6	0	0	0	0	0	0	0	0	0	0	0	0	0	0	0	0	0	0	0	0	0	0	0	0	0	0	0.110022	0	0	0.209491	0	1.414954	0	0	0	0.270434		
Bacteria|OD1	0.090394	0.283942	0.047527	0	0.043244	0.25989	0	0	0.044288	0.041712	0.924943	0	0	0	0.174925	0	0	0	0	0	0	0	0	0	0	0	0	0	0	0	0	0	0	0	0	0		
Bacteria|OD1|ZB2	0.045197	0.189295	0.047527	0	0.043244	0.216575	0	0	0.044288	0.041712	0.924943	0	0	0	0.087462	0	0	0	0	0	0	0	0	0	0	0	0	0	0	0	0	0	0	0	0	0		
Bacteria|OP1	0	0	0	0	0	0	0	0	0	0	0	0	0	0	0	0	0	0	0	0	0	0	0	0	0	0	0	0	0	0.034915	0	0.589564	0	0	0	1.65265		
Bacteria|OP1|OPB14	0	0	0	0	0	0	0	0	0	0	0	0	0	0	0	0	0	0	0	0	0	0	0	0	0	0	0	0	0	0.034915	0	0.589564	0	0	0	1.65265		
Bacteria|OP11	0	0	0.047527	0	0	0	0	0	0	0	0	0	0	0	0	0	0	0	0	0	0.617733	0	0	0	0	0.697038	0.275055	0.596778	0.231115	0.418983	0.106059	0.221087	0	0	0.481301	0		
Bacteria|OP11|OP11-2	0	0	0	0	0	0	0	0	0	0	0	0	0	0	0	0	0	0	0	0	0.393103	0	0	0	0	0.647249	0.275055	0.596778	0.184892	0.418983	0.106059	0.221087	0	0	0.481301	0		
Bacteria|OP11|OP11-2|KD3-145	0	0	0	0	0	0	0	0	0	0	0	0	0	0	0	0	0	0	0	0	0.393103	0	0	0	0	0.248942	0.110022	0.547046	0.138669	0.418983	0.106059	0.221087	0	0	0	0		
Bacteria|OP8	0.090394	0	0.047527	0	0.043244	0.129945	0	0	0	0	0	0	0	0	0	0	0	0	0	0	0	0	0	0	0	0.099577	0.055011	0.298389	0.138669	0.471356	0.106059	1.81291	0.110138	0	0.320867	2.659264	Mangrove	9.45E-07
Bacteria|OP8|OP8_1	0	0	0	0	0	0.043315	0	0	0	0	0	0	0	0	0	0	0	0	0	0	0	0	0	0	0	0.099577	0.055011	0.198926	0	0.471356	0.05303	1.503389	0.110138	0	0.320867	2.208542		
Bacteria|OP8|OP8_1|HMMVPog-54	0	0	0	0	0	0.043315	0	0	0	0	0	0	0	0	0	0	0	0	0	0	0	0	0	0	0	0	0	0	0	0	0	0	0	0	0	0		
Bacteria|OP8|OP8_1|OPB95	0	0	0	0	0	0	0	0	0	0	0	0	0	0	0	0	0	0	0	0	0	0	0	0	0	0.049788	0.055011	0.149194	0	0.471356	0.05303	1.503389	0.110138	0	0.320867	2.208542		
Bacteria|OP8|SAW1_B6	0	0	0	0	0	0	0	0	0	0	0	0	0	0	0	0	0	0	0	0	0	0	0	0	0	0	0	0	0	0	0	0	0	0	0	0		
Bacteria|Planctomycetes	1.401109	2.082244	1.473322	1.670764	3.546019	2.33901	5.500361	0.149236	0.088576	0	0.822171	0.129542	0.298161	0.185895	0.699699	0.473048	0.081137	0	0	0	0.393103	0	0	0.151361	0.206586	1.24471	0.385077	0.646509	0.092446	0.36661	0.318178	2.078214	1.927419	0.230357	0.053478	1.171879	China oil refineries	3.99E-06
Bacteria|Planctomycetes|BD7-11	0	0	0.047527	0	0	0	0	0	0	0	0	0	0	0	0	0	0	0	0	0	0	0	0	0	0	0.896191	0.165033	0	0	0	0	0	1.597004	0.057589	0	0		
Bacteria|Planctomycetes|Brocadiae	0	0.141971	0	0.190944	0	0.08663	0	0	0	0	0	0	0	0	0	0	0	0	0	0	0	0	0	0	0	0	0	0	0	0	0	0	0	0	0	0		
Bacteria|Planctomycetes|Brocadiae|Brocadiales	0	0.141971	0	0.190944	0	0.08663	0	0	0	0	0	0	0	0	0	0	0	0	0	0	0	0	0	0	0	0	0	0	0	0	0	0	0	0	0	0		
Bacteria|Planctomycetes|Brocadiae|Brocadiales|Brocadiaceae	0	0.141971	0	0.190944	0	0.08663	0	0	0	0	0	0	0	0	0	0	0	0	0	0	0	0	0	0	0	0	0	0	0	0	0	0	0	0	0	0		
Bacteria|Planctomycetes|Brocadiae|Brocadiales|Brocadiaceae|Candidatus_Scalindua	0	0.141971	0	0.190944	0	0.08663	0	0	0	0	0	0	0	0	0	0	0	0	0	0	0	0	0	0	0	0	0	0	0	0	0	0	0	0	0	0		
Bacteria|Planctomycetes|C6	0	0.094647	0.047527	0	0.043244	0.08663	0	0	0	0	0	0	0	0	0	0	0	0	0	0	0	0	0	0	0	0	0	0	0	0	0	0	0	0	0	0		
Bacteria|Planctomycetes|C6|d113	0	0.047324	0	0	0.043244	0	0	0	0	0	0	0	0	0	0	0	0	0	0	0	0	0	0	0	0	0	0	0	0	0	0	0	0	0	0	0		
Bacteria|Planctomycetes|OM190	0.406774	0.236619	0.14258	0.190944	0.129732	0.736355	0	0	0	0	0	0	0	0	0	0	0	0	0	0	0	0	0	0	0	0	0	0	0	0	0	0	0	0	0	0		
Bacteria|Planctomycetes|OM190|agg27	0.090394	0	0.047527	0.047736	0.086488	0.17326	0	0	0	0	0	0	0	0	0	0	0	0	0	0	0	0	0	0	0	0	0	0	0	0	0	0	0	0	0	0		
Bacteria|Planctomycetes|OM190|CL500-15	0.316379	0.236619	0.095053	0.143208	0.043244	0.563095	0	0	0	0	0	0	0	0	0	0	0	0	0	0	0	0	0	0	0	0	0	0	0	0	0	0	0	0	0	0		
Bacteria|Planctomycetes|Phycisphaerae	0.406774	0.567885	0.522792	0.477361	0.475685	0.43315	4.741691	0.049745	0.088576	0	0.051386	0	0	0	0.568506	0.387039	0	0	0	0	0	0	0	0	0.051646	0.248942	0.110022	0.248657	0	0.052373	0.159089	0.530608	0.220276	0.172768	0.053478	0.180289	China oil refineries	1.45E-06
Bacteria|Planctomycetes|Phycisphaerae|MSBL9	0	0	0	0	0	0	0	0	0	0	0	0	0	0	0	0	0	0	0	0	0	0	0	0	0	0.199154	0.110022	0.149194	0	0.052373	0.159089	0.486391	0.220276	0.115179	0	0.180289		
Bacteria|Planctomycetes|Phycisphaerae|Phycisphaerales	0.406774	0.236619	0.237633	0.429625	0.475685	0.216575	0	0	0	0	0	0	0	0	0	0	0	0	0	0	0	0	0	0	0	0	0	0	0	0	0	0	0	0	0	0	Mangrove	1.04E-07
Bacteria|Planctomycetes|Phycisphaerae|Phycisphaerales|Phycisphaeraceae	0.406774	0.236619	0.237633	0.429625	0.475685	0.216575	0	0	0	0	0	0	0	0	0	0	0	0	0	0	0	0	0	0	0	0	0	0	0	0	0	0	0	0	0	0	Mangrove	1.04E-07
Bacteria|Planctomycetes|Pla3	0	0.189295	0.047527	0.047736	0	0.129945	0	0	0	0	0	0	0	0	0	0	0	0	0	0	0	0	0	0	0	0	0	0	0	0.157119	0	1.193868	0	0	0	0.99159		
Bacteria|Planctomycetes|Planctomycetia	0.587562	0.709856	0.617845	0.763778	2.897357	0.822985	0.758671	0.099491	0	0	0.770786	0.129542	0.298161	0.185895	0.131194	0.086009	0.081137	0	0	0	0.393103	0	0	0.151361	0.154939	0.099577	0.110022	0.397852	0.092446	0.157119	0.159089	0.353739	0.110138	0	0	0	China oil refineries	1.63E-06
Bacteria|Planctomycetes|Planctomycetia|Gemmatales	0	0	0	0	0	0	0.284501	0.099491	0	0	0.205543	0.129542	0.298161	0.185895	0.043731	0.043004	0.081137	0	0	0	0.280788	0	0	0.151361	0.154939	0	0	0.198926	0	0.052373	0	0.088435	0	0	0	0	China oil refineries	9.46E-06
Bacteria|Planctomycetes|Planctomycetia|Gemmatales|Gemmataceae	0	0	0	0	0	0	0.284501	0.049745	0	0	0.205543	0	0.042594	0	0	0.043004	0	0	0	0	0	0	0	0.151361	0	0	0	0	0	0	0	0	0	0	0	0	China oil refineries	2.82E-06
Bacteria|Planctomycetes|Planctomycetia|Gemmatales|Gemmataceae|Gemmata	0	0	0	0	0	0	0.142251	0.049745	0	0	0.205543	0	0	0	0	0	0	0	0	0	0	0	0	0.151361	0	0	0	0	0	0	0	0	0	0	0	0		
Bacteria|Planctomycetes|Planctomycetia|Gemmatales|Isosphaeraceae	0	0	0	0	0	0	0	0.049745	0	0	0	0.129542	0.255567	0.185895	0.043731	0	0.081137	0	0	0	0.280788	0	0	0	0.154939	0	0	0.198926	0	0.052373	0	0.088435	0	0	0	0		
Bacteria|Planctomycetes|Planctomycetia|Pirellulales	0.451971	0.567885	0.427739	0.572833	0.951371	0.563095	0.142251	0	0	0	0.051386	0	0	0	0	0.043004	0	0	0	0	0	0	0	0	0	0.099577	0.110022	0.198926	0.092446	0.104746	0.159089	0.265304	0.110138	0	0	0	China oil refineries	2.17E-07
Bacteria|Planctomycetes|Planctomycetia|Pirellulales|Pirellulaceae	0.451971	0.567885	0.427739	0.572833	0.951371	0.563095	0.142251	0	0	0	0.051386	0	0	0	0	0.043004	0	0	0	0	0	0	0	0	0	0.099577	0.110022	0.198926	0.092446	0.104746	0.159089	0.265304	0.110138	0	0	0	China oil refineries	2.17E-07
Bacteria|Planctomycetes|Planctomycetia|Planctomycetales	0.135591	0.141971	0.190106	0.190944	1.945986	0.25989	0.331918	0	0	0	0.513857	0	0	0	0.087462	0	0	0	0	0	0.112315	0	0	0	0	0	0	0	0	0	0	0	0	0	0	0	China oil refineries	4.96E-07
Bacteria|Planctomycetes|Planctomycetia|Planctomycetales|Planctomycetaceae	0.135591	0.141971	0.190106	0.190944	1.945986	0.25989	0.331918	0	0	0	0.513857	0	0	0	0.087462	0	0	0	0	0	0.112315	0	0	0	0	0	0	0	0	0	0	0	0	0	0	0	China oil refineries	4.96E-07
Bacteria|Planctomycetes|Planctomycetia|Planctomycetales|Planctomycetaceae|Planctomyces	0.135591	0.141971	0.190106	0.190944	1.945986	0.25989	0.331918	0	0	0	0.513857	0	0	0	0.087462	0	0	0	0	0	0.112315	0	0	0	0	0	0	0	0	0	0	0	0	0	0	0	China oil refineries	4.96E-07
Bacteria|Proteobacteria	61.17573	52.25193	61.47183	66.20124	55.4326	65.55591	77.2295	86.99985	11.0277	81.96363	77.23271	85.86747	27.89276	85.36441	82.58054	95.47689	2.085211	94.65591	88.90717	88.97144	82.8765	91.8637	94.29018	90.18551	86.90001	77.37117	85.95203	49.39166	38.99991	73.8632	77.15281	36.48982	90.34088	94.2833	83.24004	48.78773	Oil sands core	2.95E-05
Bacteria|Proteobacteria|Alphaproteobacteria	19.83699	8.343174	7.608997	7.508891	29.24601	15.02597	65.42743	80.10181	8.119459	81.27539	76.53044	73.75964	26.56476	83.37378	71.7629	87.06237	1.406368	87.62612	39.92836	32.05034	19.43987	33.73711	32.48221	31.23925	29.55896	2.099411	0.440088	1.765468	1.001499	0.899068	1.546698	3.832168	1.31248	0.796652	17.15747	7.504534	Taiga bottom active layer	3.3E-06
Bacteria|Proteobacteria|Alphaproteobacteria|BD7-3	2.440641	0.37859	0.285159	0.477361	1.124347	0.60641	0.948338	0	0	0	1.541571	0	0	0	0.087462	0	0	0	0	0	0	0	0	0	0	0	0	0.099463	0.092446	0	0.106059	0.088435	0	0	0	0.270434	Marine sediments	0.000605
Bacteria|Proteobacteria|Alphaproteobacteria|Caulobacterales	0	0	0	0	0	0	16.37464	37.27594	1.269588	16.54567	35.79871	9.658058	23.85291	33.86382	7.784154	2.866959	0.108182	64.97855	8.888584	6.186059	3.163541	8.370606	7.917114	6.390802	5.595028	0.049788	0.055011	0.066309	0	0.06983	0.035353	0.677999	0.183564	0.038393	4.028664	1.592554	Taiga bottom active layer	9.52E-08
Bacteria|Proteobacteria|Alphaproteobacteria|Caulobacterales|Caulobacteraceae	0	0	0	0	0	0	16.37464	37.27594	1.269588	16.54567	35.79871	9.658058	23.85291	33.86382	7.784154	2.866959	0.108182	64.97855	8.888584	6.186059	3.163541	8.370606	7.917114	6.390802	5.595028	0.049788	0.055011	0.066309	0	0.06983	0.035353	0.677999	0.183564	0.038393	4.028664	1.592554	Taiga bottom active layer	9.52E-08
Bacteria|Proteobacteria|Alphaproteobacteria|Caulobacterales|Caulobacteraceae|Arthrospira	0	0	0	0	0	0	0.790282	2.288292	0	2.363667	23.63742	0.172722	0.255567	2.261719	0.204079	0.02867	0	3.331315	0	0	0	0	0	0	0	0	0	0	0	0	0	0	0	0	0	0	Taiga bottom active layer	2.49E-07
Bacteria|Proteobacteria|Alphaproteobacteria|Caulobacterales|Caulobacteraceae|Arthrospira|fusiformis	0	0	0	0	0	0	0.790282	2.288292	0	2.363667	23.63742	0.172722	0.255567	2.261719	0.204079	0.02867	0	3.331315	0	0	0	0	0	0	0	0	0	0	0	0	0	0	0	0	0	0	Taiga bottom active layer	2.49E-07
Bacteria|Proteobacteria|Alphaproteobacteria|Caulobacterales|Caulobacteraceae|Caulobacter	0	0	0	0	0	0	3.192738	0	0	0.027808	8.701312	4.174123	0	0.030982	2.653026	1.519488	0	0.859694	2.353894	2.033301	0.599014	1.901639	1.932657	1.177253	1.342807	0	0	0	0	0.034915	0	0.117913	0	0	1.461728	0.420675	Taiga bottom active layer	0.00168
Bacteria|Proteobacteria|Alphaproteobacteria|Caulobacterales|Caulobacteraceae|Caulobacter|henricii	0	0	0	0	0	0	3.161127	0	0	0.027808	8.667055	4.174123	0	0.030982	2.653026	1.519488	0	0.859694	2.283628	1.964375	0.561575	1.901639	1.932657	1.177253	1.273945	0	0	0	0	0.034915	0	0.117913	0	0	1.461728	0.420675	Taiga bottom active layer	0.000326
Bacteria|Proteobacteria|Alphaproteobacteria|Caulobacterales|Caulobacteraceae|Mycoplana	0	0	0	0	0	0	0.379335	0	0	0	0	0	0	0	0	0	0	0	0.052699	0.155082	0.056158	0.356557	0	0.050454	0.051646	0.049788	0.055011	0	0	0	0	0.530608	0	0	0	0		
Bacteria|Proteobacteria|Alphaproteobacteria|Caulobacterales|Caulobacteraceae|Phenylobacterium	0	0	0	0	0	0	12.01228	34.98765	1.269588	14.15419	3.357199	5.268031	23.59734	31.57112	4.927049	1.318801	0.108182	60.78754	6.218496	3.894288	2.50837	6.010537	5.933597	5.112642	4.200575	0	0	0.066309	0	0.034915	0.035353	0.029478	0.183564	0.038393	2.566936	1.171879	Taiga permafrost layer	5.6E-08
Bacteria|Proteobacteria|Alphaproteobacteria|Ellin329	0	0.047324	0	0	0	0	1.801842	1.0944	0	52.97395	1.181871	0.604528	0.042594	3.439052	0.306118	0.516053	0	0	0	0	0	0	0	0	0	0	0	0	0	0	0	0	0	0	0	0	Taiga upper active layer	0.000301
Bacteria|Proteobacteria|Alphaproteobacteria|Kiloniellales	0.542365	0.189295	0.047527	0.143208	0.259465	0.996245	0	0	0	0	0	0	0	0	0	0	0	0	0	0	0	0	0	0	0	0	0	0	0	0	0	0	0	0	0	0		
Bacteria|Proteobacteria|Alphaproteobacteria|Rhizobiales	1.988671	3.54928	2.471379	2.100389	3.264932	3.681775	15.50533	2.959855	1.313876	2.474898	21.92457	4.361239	2.477579	3.872807	6.778336	3.347174	1.257617	0.537309	30.67088	24.02914	12.89752	23.8384	22.63243	23.91506	18.41195	2.024728	0.247549	0.779127	0.469934	0.724491	0.954533	2.64567	0.550691	0.556697	12.11273	5.078143	Oil sands core	1.44E-05
Bacteria|Proteobacteria|Alphaproteobacteria|Rhizobiales|Beijerinckiaceae	0	0	0	0	0	0	0.268696	0.074618	0.073813	0.020856	0.462471	0.151132	0.042594	0.178149	0.065597	0.695238	0	0	0.790487	0.723717	0.393103	0.458431	0.483164	0.302722	0.232409	0	0	0	0	0	0	0.176869	0	0	0.401084	0.112681		
Bacteria|Proteobacteria|Alphaproteobacteria|Rhizobiales|Bradyrhizobiaceae	0	0	0	0	0.021622	0	5.83228	2.561892	0.575743	0.458829	11.94718	3.022641	1.277834	2.346921	3.454765	1.311634	0.628809	0.214924	0.974934	0.827105	0.44926	0.840456	1.042618	0.454083	0.309878	0	0	0	0	0.026186	0.026515	0.176869	0	0	0.160434	0.045072	Taiga bottom active layer	2.88E-07
Bacteria|Proteobacteria|Alphaproteobacteria|Rhizobiales|Bradyrhizobiaceae|Bosea	0	0	0	0	0	0	2.60793	0.149236	0.310016	0.041712	8.016169	1.511321	0.127783	0.046474	1.880442	0.215022	0.24341	0.053731	0	0.310165	0	0.101874	0.305156	0	0	0	0	0	0	0	0	0	0	0	0	0		
Bacteria|Proteobacteria|Alphaproteobacteria|Rhizobiales|Bradyrhizobiaceae|Bosea|genosp.	0	0	0	0	0	0	2.60793	0.149236	0.310016	0.041712	8.016169	1.511321	0.127783	0.046474	1.880442	0.215022	0.24341	0.053731	0	0.310165	0	0.101874	0.305156	0	0	0	0	0	0	0	0	0	0	0	0	0		
Bacteria|Proteobacteria|Alphaproteobacteria|Rhizobiales|Bradyrhizobiaceae|Bradyrhizobium	0	0	0	0	0	0	0	0	0	0	3.751156	1.48973	1.128754	2.230737	1.552458	1.096612	0.365115	0.161193	0.553341	0.4394	0.252709	0.432962	0.508594	0.428856	0.206586	0	0	0	0	0.026186	0.026515	0.132652	0	0	0	0	Taiga bottom active layer	2.6E-08
Bacteria|Proteobacteria|Alphaproteobacteria|Rhizobiales|Bradyrhizobiaceae|Bradyrhizobium|elkanii	0	0	0	0	0	0	0	0	0	0	0	0	0	0	0	0	0	0	0.553341	0.4394	0.252709	0.432962	0.508594	0.428856	0.206586	0	0	0	0	0.026186	0.026515	0.132652	0	0	0	0		
Bacteria|Proteobacteria|Alphaproteobacteria|Rhizobiales|Brucellaceae	0	0	0	0	0	0	0	0	0	0	0	0.172722	0.085189	0	0.34985	0.172018	0.081137	0	4.110531	3.721974	2.133987	4.584308	4.170471	4.843555	2.788906	0	0	0	0	0	0	0	0	0	0	1.081735	Oil sands core	3.54E-07
Bacteria|Proteobacteria|Alphaproteobacteria|Rhizobiales|Brucellaceae|Ochrobactrum	0	0	0	0	0	0	0	0	0	0	0	0.172722	0.085189	0	0.34985	0.172018	0.081137	0	4.110531	3.721974	2.133987	4.584308	4.170471	4.843555	2.788906	0	0	0	0	0	0	0	0	0	0	1.081735	Oil sands core	3.54E-07
Bacteria|Proteobacteria|Alphaproteobacteria|Rhizobiales|Brucellaceae|Ochrobactrum|intermedium	0	0	0	0	0	0	0	0	0	0	0	0	0	0	0	0	0	0	4.110531	3.721974	2.133987	4.584308	4.170471	4.641741	2.788906	0	0	0	0	0	0	0	0	0	0	1.081735	Oil sands core	1.88E-08
Bacteria|Proteobacteria|Alphaproteobacteria|Rhizobiales|Hyphomicrobiaceae	0	0	0	0	0.043244	0	1.501535	0.024873	0.088576	0.451878	6.603063	0.554151	0.624719	0.697105	0.320695	0.121846	0.060852	0	0	0.068925	0.627093	0.101874	0	0	0.206586	0.049788	0.027505	0	0.023112	0.052373	0.318178	0.176869	0	0	0	0.045072	Taiga bottom active layer	6.93E-05
Bacteria|Proteobacteria|Alphaproteobacteria|Rhizobiales|Hyphomicrobiaceae|Devosia	0	0	0	0	0	0	0.031611	0	0	0.139039	2.055428	0.057574	0.028396	0.557684	0.058308	0.057339	0	0	0	0.068925	0.262069	0	0	0	0.206586	0	0	0	0	0	0	0	0	0	0	0		
Bacteria|Proteobacteria|Alphaproteobacteria|Rhizobiales|Hyphomicrobiaceae|Hyphomicrobium	0	0	0	0	0.043244	0	0.474169	0	0	0	0	0.086361	0	0	0.087462	0	0	0	0	0	0.336945	0.101874	0	0	0	0.049788	0	0	0	0	0	0.176869	0	0	0	0.045072		
Bacteria|Proteobacteria|Alphaproteobacteria|Rhizobiales|Hyphomicrobiaceae|Parvibaculum	0	0	0	0	0	0	0	0	0	0	0	0	0	0	0	0	0	0	0	0	0	0	0	0	0	0	0	0	0	0	0.212119	0	0	0	0	0		
Bacteria|Proteobacteria|Alphaproteobacteria|Rhizobiales|Hyphomicrobiaceae|Rhodoplanes	0	0	0	0	0	0	0.758671	0.024873	0.088576	0.312838	4.547635	0.410216	0.596323	0.139421	0.087462	0.064507	0.060852	0	0	0	0.028079	0	0	0	0	0	0.027505	0	0.023112	0.052373	0.106059	0	0	0	0	0		
Bacteria|Proteobacteria|Alphaproteobacteria|Rhizobiales|Methylobacteriaceae	0	0	0	0	0	0	0	0	0.088576	0.125135	0	0	0.021297	0	0.021866	0	0	0	4.347677	4.936786	1.319702	4.100408	4.017893	3.329944	3.770188	0	0	0	0	0.052373	0.026515	0.464282	0	0	2.72737	1.059199	Oil sands core	5.24E-06
Bacteria|Proteobacteria|Alphaproteobacteria|Rhizobiales|Methylobacteriaceae|Methylobacterium	0	0	0	0	0	0	0	0	0.088576	0.125135	0	0	0.021297	0	0	0	0	0	4.347677	4.936786	1.319702	4.100408	4.017893	3.329944	3.770188	0	0	0	0	0.052373	0.026515	0.464282	0	0	2.72737	1.059199	Oil sands core	7.4E-06
Bacteria|Proteobacteria|Alphaproteobacteria|Rhizobiales|Methylobacteriaceae|Methylobacterium|adhaesivum	0	0	0	0	0	0	0	0	0	0	0	0	0	0	0	0	0	0	1.449226	1.188964	0.393103	0.891393	1.144337	0.580218	0.929635	0	0	0	0	0.026186	0	0.132652	0	0	0.347606	0.180289	Oil sands core	1.11E-06
Bacteria|Proteobacteria|Alphaproteobacteria|Rhizobiales|Methylobacteriaceae|Methylobacterium|hispanicum	0	0	0	0	0	0	0	0	0	0	0	0	0	0	0	0	0	0	0	0	0	0	0	0	0	0	0	0	0	0	0	0	0	0	0	0		
Bacteria|Proteobacteria|Alphaproteobacteria|Rhizobiales|Methylocystaceae	0	0	0	0	0	0	0	0	0	0.625677	0	0.043181	0	0	0.043731	0	0	0	0	0	0.056158	0	0	0	0	0	0	0	0	0	0	0	0.146851	0	0	0		
Bacteria|Proteobacteria|Alphaproteobacteria|Rhizobiales|Methylocystaceae|Pleomorphomonas	0	0	0	0	0	0	0	0	0	0.625677	0	0	0	0	0.043731	0	0	0	0	0	0.056158	0	0	0	0	0	0	0	0	0	0	0	0	0	0	0		
Bacteria|Proteobacteria|Alphaproteobacteria|Rhizobiales|Methylocystaceae|Pleomorphomonas|oryzae	0	0	0	0	0	0	0	0	0	0.625677	0	0	0	0	0.043731	0	0	0	0	0	0.056158	0	0	0	0	0	0	0	0	0	0	0	0	0	0	0		
Bacteria|Proteobacteria|Alphaproteobacteria|Rhizobiales|Rhizobiaceae	0	0	0	0	0	0	0.995755	0	0.044288	0	0.051386	0	0	0.278842	0.087462	0.043004	0	0.053731	0.263496	0.516941	0	0.458431	0.203438	0.454083	0.103293	0.049788	0	0	0	0	0	0	0	0	0	0		
Bacteria|Proteobacteria|Alphaproteobacteria|Rhizobiales|Rhizobiaceae|Kaistia	0	0	0	0	0	0	0.806087	0	0.044288	0	0	0	0	0	0.043731	0.043004	0	0	0	0	0	0	0	0	0	0	0	0	0	0	0	0	0	0	0	0		
Bacteria|Proteobacteria|Alphaproteobacteria|Rhizobiales|Rhodobiaceae	0	0	0	0	0	0	0.711254	0	0.132864	0	0.976328	0	0.298161	0	0.087462	0	0.446251	0	0	0	0	0	0	0	0	0	0	0	0	0	0	0	0	0	0	0		
Bacteria|Proteobacteria|Alphaproteobacteria|Rhizobiales|Rhodobiaceae|Afifella	0	0	0	0	0	0	0.711254	0	0.132864	0	0.976328	0	0.298161	0	0.087462	0	0.446251	0	0	0	0	0	0	0	0	0	0	0	0	0	0	0	0	0	0	0		
Bacteria|Proteobacteria|Alphaproteobacteria|Rhodobacterales	14.68452	4.178685	4.686116	4.764064	24.5324	9.654914	5.097318	0	0.022144	0.041712	0.154157	0	0.021297	0	0.54664	0.064507	0	0	0	0.180929	1.853199	0.382026	0.050859	0.277495	0.413171	0	0.082516	0.198926	0.069335	0.052373	0.132574	0	0.330415	0.028795	0.374345	0	Marine sediments	0.000689
Bacteria|Proteobacteria|Alphaproteobacteria|Rhodobacterales|Hyphomonadaceae	0.045197	0	0	0.047736	0.043244	0.043315	5.073609	0	0	0	0	0	0	0	0.043731	0	0	0	0	0	0	0	0	0	0	0	0	0	0	0	0	0	0.055069	0	0	0		
Bacteria|Proteobacteria|Alphaproteobacteria|Rhodobacterales|Rhodobacteraceae	14.63933	4.178685	4.686116	4.716328	24.48915	9.611599	0.023708	0	0.022144	0.041712	0.154157	0	0.021297	0	0.502909	0.064507	0	0	0	0.180929	1.853199	0.382026	0.050859	0.277495	0.413171	0	0.082516	0.198926	0.069335	0.052373	0.132574	0	0.275346	0.028795	0.374345	0	Marine sediments	0.000182
Bacteria|Proteobacteria|Alphaproteobacteria|Rhodobacterales|Rhodobacteraceae|Amaricoccus	0	0	0	0	0	0	0	0	0	0.041712	0	0	0	0	0	0	0	0	0	0	0	0	0	0	0	0	0	0	0	0	0	0	0	0	0	0		
Bacteria|Proteobacteria|Alphaproteobacteria|Rhodobacterales|Rhodobacteraceae|Octadecabacter	10.93769	0	1.425796	2.291334	9.340733	7.53681	0	0	0	0	0	0	0	0	0	0	0	0	0	0	0	0	0	0	0	0	0	0	0	0	0	0	0	0	0	0	Marine sediments	1.27E-05
Bacteria|Proteobacteria|Alphaproteobacteria|Rhodobacterales|Rhodobacteraceae|Paracoccus	0	0	0	0	0	0.021658	0	0	0.022144	0	0	0	0	0	0.021866	0	0	0	0	0	0.589654	0.050937	0	0.227042	0.284055	0	0	0	0	0	0	0	0	0	0.080217	0		
Bacteria|Proteobacteria|Alphaproteobacteria|Rhodobacterales|Rhodobacteraceae|Paracoccus|marcusii	0	0	0	0	0	0	0	0	0	0	0	0	0	0	0	0	0	0	0	0	0.505418	0	0	0	0	0	0	0	0	0	0	0	0	0	0	0		
Bacteria|Proteobacteria|Alphaproteobacteria|Rhodobacterales|Rhodobacteraceae|Phaeobacter	2.277932	1.173629	1.501838	0.706494	6.78068	0.970256	0	0	0	0	0	0	0	0	0	0	0	0	0	0	0	0	0	0	0	0	0	0	0	0	0	0	0	0	0	0	Marine sediments	8.66E-08
Bacteria|Proteobacteria|Alphaproteobacteria|Rhodospirillales	0	0	0.07129	0.023868	0.021622	0	1.138006	1.114299	0.243584	1.647615	1.181871	0.280674	0	1.254789	0.284253	1.010603	0	0.080596	0	0.155082	0.514777	0.025468	0	0	0.129116	0.024894	0	0.124329	0.046223	0	0.05303	0.022109	0.027535	0	0	0.022536	India oil refineries	4.09E-05
Bacteria|Proteobacteria|Alphaproteobacteria|Rhodospirillales|Acetobacteraceae	0	0	0.07129	0.023868	0.021622	0	0.260793	0.865571	0.243584	0.271127	0.668014	0.215903	0	0.325316	0.131194	1.010603	0	0	0	0.077541	0.149753	0	0	0	0.051646	0	0	0.099463	0.046223	0	0.026515	0	0.027535	0	0	0	India oil refineries	6.04E-05
Bacteria|Proteobacteria|Alphaproteobacteria|Rhodospirillales|Acetobacteraceae|Acidocella	0	0	0	0	0	0	0	0	0	0	0	0	0	0	0	0	0	0	0	0	0.149753	0	0	0	0	0	0	0	0	0	0	0	0	0	0	0		

Table S2. IV

Bacterial clades	India oil refineries				Arctic												China oil refineries									Mangrove		
	I1	I2	I3	I4	A1	A10	A11	A12	A2	A3	A4	A5	A6	A7	A8	A9	C1	C2	C3	C4	C5	C6	C7	C8	C9	M1	M2	M3
Bacteria|Proteobacteria|Alphaproteobacteria|Rhodospirillales|Acetobacteraceae|Roseococcus	0	0	0.06519	0.149321	0	0	0	0	0	0	0	0	0	0	0	0	0.023513	0	0	0	0	0.021321	0	0	0	0.019503	0	0
Bacteria|Proteobacteria|Alphaproteobacteria|Rhodospirillales|Rhodospirillaceae	1.760984	0.503512	0.282489	1.258563	0	0	0	0	0.040557	0	0	0	0.068672	0	0.48144	0	0.56432	0.359814	0.52792	0.579792	0.824296	0.426422	0.360566	0.207538	0.546766	0.292538	0.751765	0.296074
Bacteria|Proteobacteria|Alphaproteobacteria|Rhodospirillales|Rhodospirillaceae|Phaeospirillum	0.604782	0.154927	0.06519	0.042663	0	0	0	0	0	0	0	0	0	0	0	0	0.023513	0	0.021117	0	0	0	0	0	0	0	0	0
Bacteria|Proteobacteria|Alphaproteobacteria|Rhodospirillales|Rhodospirillaceae|Phaeospirillum|fulvum	0.604782	0.154927	0.06519	0.042663	0	0	0	0	0	0	0	0	0	0	0	0	0.023513	0	0.021117	0	0	0	0	0	0	0	0	0
Bacteria|Proteobacteria|Alphaproteobacteria|Sphingomonadales	7.755444	0.968292	0.521519	1.237232	4.377183	2.682999	3.436506	4.66304	0.405567	2.303585	0.920336	2.976099	0.686717	0.702044	1.004745	1.621397	1.975121	2.398763	0.633503	2.244356	3.153828	3.326089	2.403772	3.49356	2.649712	0.23403	0.568904	0.592147
Bacteria|Proteobacteria|Alphaproteobacteria|Sphingomonadales|Erythrobacteraceae	0.106726	0	0	0.042663	0.093132	0.127762	1.080045	0.23791	0	0.047012	0.13805	0	0	0.093606	0	0	0.14108	0.279856	0.126701	0.561089	0.286712	0.383779	0.120189	0.588025	0.37853	0.195025	0.446996	0.592147
Bacteria|Proteobacteria|Alphaproteobacteria|Sphingomonadales|Sphingomonadaceae	7.648718	0.968292	0.521519	1.194568	4.284051	2.555237	2.356462	4.42513	0.405567	2.256573	0.782286	2.976099	0.686717	0.608438	1.004745	1.621397	1.834041	2.118907	0.506803	1.683267	2.867116	2.942309	2.283583	2.905535	2.271182	0.039005	0.121908	0
Bacteria|Proteobacteria|Alphaproteobacteria|Sphingomonadales|Sphingomonadaceae|Kaistobacter	0.035575	0	0	0	0	0	0.049093	0	0	0.047012	0	0.043132	0	0	0	0	0.28216	0.999484	0.126701	0.673307	1.254363	1.236623	1.12176	0.968512	1.051473	0.039005	0	0
Bacteria|Proteobacteria|Alphaproteobacteria|Sphingomonadales|Sphingomonadaceae|Novosphingobium	0.213453	0.154927	0	0.042663	0	0	0	0	0	0	0	0	0	0	0	0	0	0	0	0.037406	0	0.170569	0.080126	0	0	0	0	0
Bacteria|Proteobacteria|Alphaproteobacteria|Sphingomonadales|Sphingomonadaceae|Novosphingobium|capsulatum	0	0	0	0.042663	0	0	0	0	0	0	0	0	0	0	0	0	0	0	0	0	0	0	0	0	0	0	0	0
Bacteria|Proteobacteria|Alphaproteobacteria|Sphingomonadales|Sphingomonadaceae|Novosphingobium|resinovorum	0	0	0	0	0	0	0	0	0	0	0	0	0	0	0	0	0	0	0	0	0	0	0	0	0	0	0	0
Bacteria|Proteobacteria|Alphaproteobacteria|Sphingomonadales|Sphingomonadaceae|Sphingobium	4.091175	0	0	0	0	0	0	0	0	0	0	0	0	0	0	0	0	0.079959	0	0	0.035839	0.298495	0	0	0.210295	0	0	0
Bacteria|Proteobacteria|Alphaproteobacteria|Sphingomonadales|Sphingomonadaceae|Sphingobium|yanoikuyae	2.561431	0	0	0	0	0	0	0	0	0	0	0	0	0	0	0	0	0	0	0	0	0	0	0	0	0	0	0
Bacteria|Proteobacteria|Alphaproteobacteria|Sphingomonadales|Sphingomonadaceae|Sphingomonas	0	0	0	0.042663	0	0	0	0	0	0	0	0.215659	0	0	0	0	0.235133	0	0.042234	0	0.215034	0.085284	0.240377	0.069179	0	0	0	0
Bacteria|Proteobacteria|Alphaproteobacteria|Sphingomonadales|Sphingomonadaceae|Sphingomonas|asaccharolytica	0	0	0	0	0	0	0	0	0	0	0	0	0	0	0	0	0.047027	0	0	0	0.107517	0.042642	0	0	0	0	0	0
Bacteria|Proteobacteria|Alphaproteobacteria|Sphingomonadales|Sphingomonadaceae|Sphingomonas|azotifigens	0	0	0	0	0	0	0	0	0	0	0	0	0	0	0	0	0.188107	0	0	0	0.035839	0.042642	0	0	0	0	0	0
Bacteria|Proteobacteria|Alphaproteobacteria|Sphingomonadales|Sphingomonadaceae|Sphingomonas|echinoides	0	0	0	0	0	0	0	0	0	0	0	0	0	0	0	0	0	0	0	0	0	0	0	0	0	0	0	0
Bacteria|Proteobacteria|Alphaproteobacteria|Sphingomonadales|Sphingomonadaceae|Sphingomonas|wittichii	0	0	0	0.042663	0	0	0	0	0	0	0	0.215659	0	0	0	0	0	0	0.042234	0	0.071678	0	0.240377	0.069179	0	0	0	0
Bacteria|Proteobacteria|Betaproteobacteria	6.047824	35.76999	21.76907	30.90803	14.90105	28.81739	15.70974	7.375216	8.570976	6.111553	12.54725	10.6967	2.563745	5.039114	16.048	12.94346	1.293234	3.138381	1.210696	2.705695	3.096485	3.915972	4.70071	2.115737	6.171446	0.23663	0.568904	0.144747
Bacteria|Proteobacteria|Betaproteobacteria|Burkholderiales	4.8264	25.1446	16.58285	18.74904	12.29336	26.09181	15.57883	6.931117	8.354674	6.04887	12.36318	10.61044	2.38062	4.602287	14.98744	12.94346	0.91702	2.458732	0.929138	1.795485	1.612753	3.290554	4.179892	1.550772	5.215307	0.07801	0.081272	0.105271
Bacteria|Proteobacteria|Betaproteobacteria|Burkholderiales|Alcaligenaceae	0	0.038732	0.04346	0	0	0	0	0	0	0	0	0	0	0	0	0	0	0	0.042234	0	0	0	0	0	0	0.026003	0	0
Bacteria|Proteobacteria|Betaproteobacteria|Burkholderiales|Burkholderiaceae	0.717438	0.524169	0.017384	0.034131	0	0	0	1.760535	0	0	0	0	0	0.046803	0	0	0.023513	0	0	0.037406	0.017919	0.028428	0	0	0	0	0	0
Bacteria|Proteobacteria|Betaproteobacteria|Burkholderiales|Burkholderiaceae|Burkholderia	0.539561	0.304689	0.017384	0.034131	0	0	0	0	0	0	0	0	0	0	0	0	0	0	0	0	0	0.028428	0	0	0	0	0	0
Bacteria|Proteobacteria|Betaproteobacteria|Burkholderiales|Burkholderiaceae|Burkholderia|bryophila	0.017788	0	0	0	0	0	0	0	0	0	0	0	0	0	0	0	0	0	0	0	0	0	0	0	0	0	0	0
Bacteria|Proteobacteria|Betaproteobacteria|Burkholderiales|Burkholderiaceae|Burkholderia|gladioli	0.213453	0.201405	0.017384	0.034131	0	0	0	0	0	0	0	0	0	0	0	0	0	0	0	0	0	0	0	0	0	0	0	0
Bacteria|Proteobacteria|Betaproteobacteria|Burkholderiales|Burkholderiaceae|Burkholderia|glathei	0	0	0	0	0	0	0	0	0	0	0	0	0	0	0	0	0	0	0	0	0	0	0	0	0	0	0	0
Bacteria|Proteobacteria|Betaproteobacteria|Burkholderiales|Burkholderiaceae|Burkholderia|tuberum	0.30832	0.103284	0	0	0	0	0	0	0	0	0	0	0	0	0	0	0	0	0	0	0	0.028428	0	0	0	0	0	0
Bacteria|Proteobacteria|Betaproteobacteria|Burkholderiales|Burkholderiaceae|Lautropia	0	0	0	0	0	0	0	1.760535	0	0	0	0	0	0.046803	0	0	0	0	0	0	0	0	0	0	0	0	0	0
Bacteria|Proteobacteria|Betaproteobacteria|Burkholderiales|Comamonadaceae	4.061529	24.5817	16.46406	18.7149	12.23128	26.00663	11.71685	5.13886	7.922069	6.04887	12.3325	10.61044	2.38062	4.305868	14.93162	11.19734	0.548645	1.286003	0.661659	0.860336	1.308122	1.186874	1.749412	1.297114	2.187064	0.052007	0.081272	0.105271
Bacteria|Proteobacteria|Betaproteobacteria|Burkholderiales|Comamonadaceae|Acidovorax	0	0	0	0.021332	0	0	0	0	0	0	0	0	0	0	0	0	0	0	0	0	0	0	0	0	0	0	0	0
Bacteria|Proteobacteria|Betaproteobacteria|Burkholderiales|Comamonadaceae|Acidovorax|delafieldii	0	0	0	0.021332	0	0	0	0	0	0	0	0	0	0	0	0	0	0	0	0	0	0	0	0	0	0	0	0
Bacteria|Proteobacteria|Betaproteobacteria|Burkholderiales|Comamonadaceae|Acidovorax|facilis	0	0	0	0	0	0	0	0	0	0	0	0	0	0	0	0	0	0	0	0	0	0	0	0	0	0	0	0
Bacteria|Proteobacteria|Betaproteobacteria|Burkholderiales|Comamonadaceae|Delftia	0	0	0	0	0	0	0	0	0	0	0	0	0	0	0	0	0	0	0	0	0	0	0	0	0	0	0	0
Bacteria|Proteobacteria|Betaproteobacteria|Burkholderiales|Comamonadaceae|Hydrogenophaga	0	0	0	0.042663	0	0	0	0	0	0	0	0	0	0	0	0	0	0.059969	0	0	0.053758	0.021321	0	0.017295	0.084118	0	0	0
Bacteria|Proteobacteria|Betaproteobacteria|Burkholderiales|Comamonadaceae|Hydrogenophaga|palleronii	0	0	0	0.021332	0	0	0	0	0	0	0	0	0	0	0	0	0	0	0	0	0	0	0	0	0	0	0	0
Bacteria|Proteobacteria|Betaproteobacteria|Burkholderiales|Comamonadaceae|Limnohabitans	0	0	0	0	0	0	0	0	0	0	0	0	0	0	0	0	0	0	0	0	0	0	0	0	0	0	0	0
Bacteria|Proteobacteria|Betaproteobacteria|Burkholderiales|Comamonadaceae|Methylibium	2.063375	8.443503	1.216877	7.082084	1.21071	13.79828	2.356462	0.285492	0	0.094024	3.957445	0	0.366249	0.280817	4.60508	0.498891	0	0	0	0	0	0	0	0	0	0	0	0
Bacteria|Proteobacteria|Betaproteobacteria|Burkholderiales|Comamonadaceae|Methylibium|petroleiphilum	2.063375	0.69717	0.869198	6.996758	0	0	2.356462	0	0	0.094024	0	0	0	0.280817	0	0	0	0	0	0	0	0	0	0	0	0	0	0
Bacteria|Proteobacteria|Betaproteobacteria|Burkholderiales|Comamonadaceae|Polaromonas	0	0	0	0	0	0	0	0	0	0	0	0	0	0	0	0	0	0	0	0	0.035839	0	0	0	0	0	0	0
Bacteria|Proteobacteria|Betaproteobacteria|Burkholderiales|Comamonadaceae|Ramlibacter	0	0	0	0.042663	0	0	0	0	0	0	0	0	0	0	0	0	0	0.239876	0.211168	0.112218	0.143356	0.170569	0.400629	0.172948	0.042059	0	0	0
Bacteria|Proteobacteria|Betaproteobacteria|Burkholderiales|Comamonadaceae|Rhodoferax	0	0	0	0	0	0	0	0	0	0	0	0	0	0	0	0	0	0	0	0	0	0.127926	0	0.03459	0	0	0	0
Bacteria|Proteobacteria|Betaproteobacteria|Burkholderiales|Comamonadaceae|Tepidimonas	0	0	0	0	0	0	0	0	0	0	0	0	0	0	0	0	0	0	0	0	0	0	0	0.02306	0	0	0	0
Bacteria|Proteobacteria|Betaproteobacteria|Burkholderiales|Comamonadaceae|Thiomonas	0.035575	1.16195	0	0	0	0	0	0	0	0	0	0	0	0	0	0	0	0	0	0	0	0	0	0	0	0	0	0
Bacteria|Proteobacteria|Betaproteobacteria|Burkholderiales|Comamonadaceae|Variovorax	0	0	0	0	0	0	0	0	0	0	0	0	0	0	0	0	0	0.039979	0	0	0	0	0.040063	0.03459	0	0	0	0
Bacteria|Proteobacteria|Betaproteobacteria|Burkholderiales|Comamonadaceae|Variovorax|paradoxus	0	0	0	0	0	0	0	0	0	0	0	0	0	0	0	0	0	0.039979	0	0	0	0	0.040063	0.03459	0	0	0	0
Bacteria|Proteobacteria|Betaproteobacteria|Burkholderiales|Oxalobacteraceae	0.047434	0	0.057947	0	0.062088	0.085175	3.861979	0.031721	0.432604	0	0.030678	0	0	0.249616	0.055819	1.74612	0.344862	1.172728	0.225246	0.897742	0.286712	2.075252	2.430481	0.253658	3.028243	0	0	0
Bacteria|Proteobacteria|Betaproteobacteria|Burkholderiales|Oxalobacteraceae|Cupriavidus	0.047434	0	0.057947	0	0.062088	0.085175	3.861979	0.031721	0.432604	0	0.030678	0	0	0.249616	0.02791	1.74612	0.062702	0.239876	0.084467	0.099749	0.143356	0.767559	0.988217	0.069179	0.897257	0	0	0
Bacteria|Proteobacteria|Betaproteobacteria|Burkholderiales|Oxalobacteraceae|Cupriavidus|gilardii	0	0	0	0	0	0	0	0	0	0	0	0	0	0	0	0	0	0	0	0	0	0	0	0	0	0	0	0
Bacteria|Proteobacteria|Betaproteobacteria|Burkholderiales|Oxalobacteraceae|Herminiimonas	0	0	0	0	0	0	0	0	0	0	0	0	0	0	0	0	0	0	0	0	0	0	0	0	0	0	0	0
Bacteria|Proteobacteria|Betaproteobacteria|Burkholderiales|Oxalobacteraceae|Janthinobacterium	0	0	0	0	0	0	0	0	0	0	0	0	0	0	0	0	0.28216	0.932852	0.140779	0.773056	0.143356	1.279265	1.442263	0.184478	2.130986	0	0	0
Bacteria|Proteobacteria|Betaproteobacteria|Burkholderiales|Oxalobacteraceae|Ralstonia	0	0	0	0	0	0	0	0	0	0	0	0	0	0	0	0	0	0	0	0.024937	0	0.028428	0	0	0	0	0	0
Bacteria|Proteobacteria|Betaproteobacteria|Ellin6067	0.023717	0	0	0.142211	0.682965	0.028392	0.032729	0.063443	0	0	0.030678	0	0	0.031202	0.02791	0	0.031351	0.133265	0.084467	0.124686	0.597316	0.198997	0.133543	0.253658	0.028039	0.026003	0.027091	0
Bacteria|Proteobacteria|Betaproteobacteria|Hydrogenophilales	0.166019	0.903739	0.985091	9.129915	0	0	0	0	0	0	0	0	0	0	0	0	0.031351	0.026653	0	0	0	0.028428	0	0.02306	0	0	0.027091	0
Bacteria|Proteobacteria|Betaproteobacteria|Hydrogenophilales|Hydrogenophilaceae	0.166019	0.903739	0.985091	9.129915	0	0	0	0	0	0	0	0	0	0	0	0	0.031351	0.026653	0	0	0	0.028428	0	0.02306	0	0	0.027091	0
Bacteria|Proteobacteria|Betaproteobacteria|Hydrogenophilales|Hydrogenophilaceae|Thiobacillus	0.166019	0.903739	0.985091	9.129915	0	0	0	0	0	0	0	0	0	0	0	0	0.031351	0.026653	0	0	0	0.028428	0	0.02306	0	0	0.027091	0
Bacteria|Proteobacteria|Betaproteobacteria|MND1	0	0.025821	0.028973	0.028442	0	0	0	0.190328	0.108151	0	0	0	0	0	0	0	0.031351	0.293182	0.112623	0.573558	0.262819	0.056856	0.106834	0.092239	0.140196	0	0.027091	0
Bacteria|Proteobacteria|Betaproteobacteria|MOB121	0.889386	2.595022	2.216455	0.127989	0	0	0	0	0	0	0	0	0	0	0	0	0.14108	0.079959	0.042234	0.112218	0.035839	0.170569	0.080126	0	0.168236	0	0	0
Bacteria|Proteobacteria|Betaproteobacteria|Nitrosomonadales	0	0	0	0	1.769499	2.640411	0.098186	0.095164	0	0	0	0	0	0.374423	0.921016	0	0	0	0	0	0.143356	0	0.080126	0.069179	0.252354	0.07801	0.40636	0
Bacteria|Proteobacteria|Betaproteobacteria|Nitrosomonadales|Nitrosomonadaceae	0	0	0	0	1.769499	2.640411	0.098186	0.095164	0	0	0	0	0	0.374423	0.921016	0	0	0	0	0	0.143356	0	0.080126	0.069179	0.252354	0.07801	0.40636	0
Bacteria|Proteobacteria|Betaproteobacteria|Rhodocyclales	0.142302	7.023342	1.694936	1.848737	0.155219	0.056783	0	0.031721	0	0	0.030678	0	0	0.031202	0.111638	0	0	0	0	0.074812	0	0	0.026709	0.02306	0.308432	0.039005	0	0
Bacteria|Proteobacteria|Betaproteobacteria|Rhodocyclales|Rhodocyclaceae	0.142302	7.023342	1.694936	1.848737	0.155219	0.056783	0	0.031721	0	0	0.030678	0	0	0.031202	0.111638	0	0	0	0	0.074812	0	0	0.026709	0.02306	0.308432	0.039005	0	0
Bacteria|Proteobacteria|Betaproteobacteria|Rhodocyclales|Rhodocyclaceae|Azospira	0	0	0.391139	0	0	0	0	0	0	0	0	0	0	0	0	0	0	0	0	0	0	0	0	0	0	0	0	0
Bacteria|Proteobacteria|Betaproteobacteria|Rhodocyclales|Rhodocyclaceae|Azovibrio	0	0.077463	0.08692	0	0	0	0	0	0	0	0	0	0	0	0	0	0	0	0	0	0	0	0	0	0	0	0	0
Bacteria|Proteobacteria|Betaproteobacteria|Rhodocyclales|Rhodocyclaceae|Dechloromonas	0	0	0	0	0	0	0	0	0	0	0	0	0	0	0	0	0	0	0	0	0	0	0	0	0	0.039005	0	0
Bacteria|Proteobacteria|Betaproteobacteria|Rhodocyclales|Rhodocyclaceae|Denitratisoma	0.094868	0.800454	0.17384	0.68261	0	0	0	0	0	0	0	0	0	0	0.02791	0	0	0	0	0	0	0	0	0	0	0	0	0
Bacteria|Proteobacteria|Betaproteobacteria|Rhodocyclales|Rhodocyclaceae|Sulfuritalea	0	0	0.028973	0.028442	0	0	0	0	0	0	0	0	0	0	0	0	0	0	0	0	0	0	0	0	0	0	0	0
Bacteria|Proteobacteria|Betaproteobacteria|Rhodocyclales|Rhodocyclaceae|Thauera	0	0	0	0	0	0	0	0	0	0	0	0	0	0	0	0	0	0	0	0	0	0	0	0	0	0	0	0
Bacteria|Proteobacteria|Betaproteobacteria|SBla14	0	0	0	0.725274	0	0	0	0	0	0	0	0	0	0	0	0	0	0	0	0	0	0	0	0	0	0	0	0
Bacteria|Proteobacteria|Deltaproteobacteria	0.213453	1.329787	3.317439	1.834516	0.279395	1.320206	0.294558	0.190328	0.527237	0.517131	0.552202	0.301923	0.183125	0.280817	1.255931	0.083149	1.881067	2.318804	4.040344	2.431385	2.867116	2.757527	2.630795	2.098442	2.313241	8.561598	8.045923	7.993985
Bacteria|Proteobacteria|Deltaproteobacteria|Bdellovibrionales	0	0	0	0.042663	0	0	0	0	0.081113	0	0.046017	0	0	0	0	0	0.14108	0.079959	0.126701	0.074812	0.107517	0.170569	0.360566	0.138359	0.126177	0.31204	0.040636	0.315812
Bacteria|Proteobacteria|Deltaproteobacteria|Bdellovibrionales|Bacteriovoracaceae	0	0	0	0.042663	0	0	0	0	0	0	0	0	0	0	0	0	0	0.039979	0	0	0	0.042642	0.120189	0.069179	0.084118	0.273035	0.040636	0.157906
Bacteria|Proteobacteria|Deltaproteobacteria|Bdellovibrionales|Bacteriovoracaceae|Bacteriovorax	0	0	0	0	0	0	0	0	0	0	0	0	0	0	0	0	0	0	0	0	0	0	0	0.03459	0	0.23403	0.040636	0.078953
Bacteria|Proteobacteria|Deltaproteobacteria|Desulfarculales	0	0	0	0.028442	0	0	0	0	0	0	0	0	0	0	0	0	0	0	0.028156	0	0	0	0	0	0	0.728093	0.975263	0.500035
Bacteria|Proteobacteria|Deltaproteobacteria|Desulfarculales|Desulfarculaceae	0	0	0	0.028442	0	0	0	0	0	0	0	0	0	0	0	0	0	0	0.028156	0	0	0	0	0	0	0.728093	0.975263	0.500035
Bacteria|Proteobacteria|Deltaproteobacteria|Desulfobacterales	0	0.025821	0.579465	0.483516	0	0	0	0	0	0.047012	0	0	0	0	0	0	0	0	0.084467	0	0	0.028428	0	0.02306	0	3.055392	2.72261	3.316023
Bacteria|Proteobacteria|Deltaproteobacteria|Desulfobacterales|Desulfobacteraceae	0	0	0	0	0	0	0	0	0	0	0	0	0	0	0	0	0	0	0.028156	0	0	0	0	0	0	1.742223	1.720256	1.539582
Bacteria|Proteobacteria|Deltaproteobacteria|Desulfobacterales|Desulfobacteraceae|Desulfobacter	0	0	0	0	0	0	0	0	0	0	0	0	0	0	0	0	0	0	0	0	0	0	0	0	0	1.17015	1.137807	0.6711
Bacteria|Proteobacteria|Deltaproteobacteria|Desulfobacterales|Desulfobacteraceae|Desulfosarcina	0	0	0	0	0	0	0	0	0	0	0	0	0	0	0	0	0	0	0.028156	0	0	0	0	0	0	0.520067	0.541813	0.868482
Bacteria|Proteobacteria|Deltaproteobacteria|Desulfobacterales|Desulfobulbaceae	0	0.025821	0.579465	0.483516	0	0	0	0	0	0.047012	0	0	0	0	0	0	0	0	0.056311	0	0	0.028428	0	0.02306	0	1.313168	1.002354	1.776441
Bacteria|Proteobacteria|Deltaproteobacteria|Desulfobacterales|Desulfobulbaceae|Desulfobulbus	0	0	0	0	0	0	0	0	0	0.047012	0	0	0	0	0	0	0	0	0	0	0	0	0	0	0	0.429055	0.325088	0.986912
Bacteria|Proteobacteria|Deltaproteobacteria|Desulfobacterales|Desulfobulbaceae|Desulfocapsa	0	0	0	0	0	0	0	0	0	0	0	0	0	0	0	0	0	0	0.028156	0	0	0	0	0	0	0	0	0
Bacteria|Proteobacteria|Deltaproteobacteria|Desulfobacterales|Desulfobulbaceae|Desulfotalea	0	0	0	0	0	0	0	0	0	0	0	0	0	0	0	0	0	0	0	0	0	0	0	0.02306	0	0	0	0
Bacteria|Proteobacteria|Deltaproteobacteria|Desulfuromonadales	0	0.038732	0	0.127989	0	0.127762	0.196372	0	0	0	0	0	0	0	0.376779	0	0.047027	0.079959	0.084467	0.074812	0.107517	0.127926	0.040063	0.138359	0.042059	2.691345	1.503531	1.539582
Bacteria|Proteobacteria|Deltaproteobacteria|Desulfuromonadales|Desulfuromonadaceae	0	0.038732	0	0.042663	0	0	0	0	0	0	0	0	0	0	0	0	0	0	0	0	0	0	0	0.03459	0	2.65234	1.462895	1.421153
Bacteria|Proteobacteria|Deltaproteobacteria|Desulfuromonadales|Geobacteraceae	0	0	0	0.085326	0	0.127762	0.196372	0	0	0	0	0	0	0	0.376779	0	0.047027	0.079959	0.084467	0.074812	0.071678	0.127926	0.040063	0.069179	0.042059	0.039005	0.040636	0.118429
Bacteria|Proteobacteria|Deltaproteobacteria|Desulfuromonadales|Geobacteraceae|Geobacter	0	0	0	0.085326	0	0.127762	0.196372	0	0	0	0	0	0	0	0.376779	0	0.047027	0.079959	0.084467	0.074812	0.071678	0.127926	0.040063	0.069179	0.042059	0.039005	0.040636	0.118429
Bacteria|Proteobacteria|Deltaproteobacteria|Desulfuromonadales|Geobacteraceae|Geobacter|lovleyi	0	0	0	0	0	0	0	0	0	0	0	0	0	0	0	0	0	0	0	0	0	0	0	0	0	0	0	0
Bacteria|Proteobacteria|Deltaproteobacteria|Desulfuromonadales|Pelobacteraceae	0	0	0	0	0	0	0	0	0	0	0	0	0	0	0	0	0	0	0	0	0.035839	0	0	0.03459	0	0	0	0
Bacteria|Proteobacteria|Deltaproteobacteria|Desulfuromonadales|Pelobacteraceae|Pelobacter	0	0	0	0	0	0	0	0	0	0	0	0	0	0	0	0	0	0	0	0	0	0	0	0.03459	0	0	0	0
Bacteria|Proteobacteria|Deltaproteobacteria|Myxococcales	0.177877	0.387317	0.2173	0.895926	0.139697	1.192444	0.098186	0.142746	0.36501	0.470119	0.460168	0.301923	0.183125	0.280817	0.837287	0.083149	1.4108	1.719113	2.196145	1.496237	2.293693	2.089466	1.722703	1.487357	1.430003	0.507065	1.462895	0.829006
Bacteria|Proteobacteria|Deltaproteobacteria|Myxococcales|0319-6G20	0	0	0	0	0	0.085175	0	0	0	0	0	0	0.091562	0	0.041864	0	0	0	0	0	0	0	0	0	0	0.039005	0	0
Bacteria|Proteobacteria|Deltaproteobacteria|Myxococcales|Haliangiaceae	0	0	0	0.298642	0	0	0	0	0	0	0	0.043132	0	0	0	0.041574	0.094053	0.439773	0.126701	0.112218	0.68094	0.383779	0.320503	0.207538	0.084118	0.07801	0.081272	0.078953
Bacteria|Proteobacteria|Deltaproteobacteria|Myxococcales|Myxococcaceae	0.106726	0.387317	0	0.042663	0.046566	0.127762	0	0	0	0	0	0	0	0	0	0	0.7054	0.63967	1.562642	0.785524	1.003491	0.938128	0.801257	0.553435	0.925296	0	0	0
Bacteria|Proteobacteria|Deltaproteobacteria|Myxococcales|Myxococcaceae|Anaeromyxobacter	0.106726	0.387317	0	0.042663	0.046566	0.127762	0	0	0	0	0	0	0	0	0	0	0.7054	0.519732	1.393708	0.748119	0.752618	0.639632	0.761194	0.449666	0.841178	0	0	0
Bacteria|Proteobacteria|Deltaproteobacteria|Myxococcales|Polyangiaceae	0	0	0	0	0	0.383286	0	0	0	0	0.230084	0.129396	0.091562	0.187212	0	0	0.047027	0.079959	0.168934	0	0.071678	0.298495	0.040063	0.03459	0.084118	0	0	0
Bacteria|Proteobacteria|Deltaproteobacteria|NB1-j	0	0.038732	0	0	0	0	0	0	0	0	0	0	0	0	0.041864	0	0	0	0	0.037406	0.035839	0	0	0	0	0.117015	0.081272	0.118429

Bacterial clades	Marine sediments							Taiga upper active layer				Taiga bottom active layer				Taiga permafrost layer				Oil sands core							Oil sands tailings pond upper				Oil sands tailings pond median			Oil sands tailings pond deep				Differentially abundant class (one class test, non-strict)	p-value
	DWH1	DWH2	DWH3	DWH4	DWH5	DWH6	DWH7	Tu1	Tu2	Tu3	Tu4	Tb1	Tb2	Tb3	Tb4	Tp1	Tp2	Tp3	Tp4	OSC1	OSC12	OSC3	OSC4	OSC5	OSC7	OSC9	OSTPu1	OSTPu2	OSTPu4	OSTPu3	OSTPm2	OSTPm6	OSTPm4	OSTPd1	OSTPd2	OSTPd3	OSTPd4		
Bacteria|Proteobacteria|Alphaproteobacteria|Rhodospirillales|Acetobacteraceae|Roseococcus	0	0	0	0	0	0	0	0.213376	0	0	0.020856	0.565243	0.064771	0	0	0.087462	0.752577	0	0	0	0	0	0	0	0	0	0	0	0	0	0	0	0	0	0	0	0		
Bacteria|Proteobacteria|Alphaproteobacteria|Rhodospirillales|Rhodospirillaceae	0.022849	0	0	0	0	0	0	0.877213	0.248727	0	1.376488	0.513857	0.064771	0	0.929474	0.153059	0	0	0.080596	0	0.077541	0.365024	0.025468	0	0	0.07747	0.024894	0	0.024866	0	0	0.026515	0.022109	0	0	0	0.022536	India oil refineries	6.09E-05
Bacteria|Proteobacteria|Alphaproteobacteria|Rhodospirillales|Rhodospirillaceae|Phaeospirillum	0	0	0	0	0	0	0	0	0	0	0	0	0	0	0	0	0	0	0	0	0	0	0	0	0	0	0	0	0	0	0	0	0	0	0	0	0		
Bacteria|Proteobacteria|Alphaproteobacteria|Rhodospirillales|Rhodospirillaceae|Phaeospirillum|fulvum	0	0	0	0	0	0	0	0	0	0	0	0	0	0	0	0	0	0	0	0	0	0	0	0	0	0	0	0	0	0	0	0	0	0	0	0	0		
Bacteria|Proteobacteria|Alphaproteobacteria|Sphingomonadales	0.182795	0.180788	0	0.047527	0	0.043244	0	24.56196	37.65732	5.270267	7.591542	14.7477	58.85514	0.170378	40.94331	55.97594	79.25708	0.040568	22.02966	0.368894	1.499129	1.010836	1.120609	1.881798	0.655898	5.009702	0	0.055011	0.497315	0.323561	0.052373	0.265148	0.397956	0.220276	0.172768	0.641734	0.540867	Taiga permafrost layer	2.3E-05
Bacteria|Proteobacteria|Alphaproteobacteria|Sphingomonadales|Erythrobacteraceae	0	0.180788	0	0	0	0.043244	0	0.142251	0	0	0.208559	1.4388	0.043181	0	0.139421	0	0	0	0.053731	0	0	0.056158	0	0	0	0	0	0.055011	0.397852	0.231115	0	0.159089	0.044217	0.110138	0.057589	0	0.045072	Mangrove	0.001862
Bacteria|Proteobacteria|Alphaproteobacteria|Sphingomonadales|Sphingomonadaceae	0.182795	0	0	0.047527	0	0	0	24.41971	37.65732	5.270267	7.382984	13.3089	58.81196	0.170378	40.80389	55.97594	79.25708	0.040568	21.97593	0.368894	1.499129	0.954678	1.120609	1.881798	0.655898	5.009702	0	0	0.099463	0.092446	0.052373	0.106059	0.353739	0.110138	0.115179	0.641734	0.495795	Taiga permafrost layer	4.99E-06
Bacteria|Proteobacteria|Alphaproteobacteria|Sphingomonadales|Sphingomonadaceae|Kaistobacter	0	0	0	0	0	0	0	0.284501	0	0	0	0	0	0	0	0.087462	0	0	0.161193	0	0	0	0	0	0	0	0	0	0	0	0	0	0	0	0	0	0	China oil refineries	1.01E-05
Bacteria|Proteobacteria|Alphaproteobacteria|Sphingomonadales|Sphingomonadaceae|Novosphingobium	0	0	0	0	0	0	0	21.57469	21.53979	5.048827	1.16793	11.61317	52.29169	0.170378	0.046474	54.70773	77.32188	0.040568	0	0	0.155082	0.056158	0.101874	0	0	0	0	0	0.099463	0	0	0	0.044217	0	0	0	0.045072	Taiga permafrost layer	9.84E-05
Bacteria|Proteobacteria|Alphaproteobacteria|Sphingomonadales|Sphingomonadaceae|Novosphingobium|capsulatum	0	0	0	0	0	0	0	2.750181	14.37644	0	0.7091	0	0.172722	0	0	6.996992	66.39877	0.040568	0	0	0	0	0	0	0	0	0	0	0	0	0	0	0	0	0	0	0	Taiga permafrost layer	9.22E-05
Bacteria|Proteobacteria|Alphaproteobacteria|Sphingomonadales|Sphingomonadaceae|Novosphingobium|resinovorum	0	0	0	0	0	0	0	0.426752	1.044655	0.088576	0.041712	0.308314	0.73407	0	0	0.787162	1.419145	0	0	0	0	0	0.050937	0	0	0	0	0	0	0	0	0	0	0	0	0	0		
Bacteria|Proteobacteria|Alphaproteobacteria|Sphingomonadales|Sphingomonadaceae|Sphingobium	0	0	0	0	0	0	0	1.754426	0	0.044288	0	0.102771	0	0	0	0.262387	0	0	0.214924	0	0.051694	0	0	0	0	0	0	0	0	0	0	0	0	0.055069	0	0.160434	0.045072		
Bacteria|Proteobacteria|Alphaproteobacteria|Sphingomonadales|Sphingomonadaceae|Sphingobium|yanoikuyae	0	0	0	0	0	0	0	0	0	0	0	0	0	0	0	0	0	0	0	0	0	0	0	0	0	0	0	0	0	0	0	0	0	0	0	0	0		
Bacteria|Proteobacteria|Alphaproteobacteria|Sphingomonadales|Sphingomonadaceae|Sphingomonas	0	0	0	0	0	0	0	0	0.646691	0	0.291982	0	0.215903	0	1.766	0	0.043004	0	3.814893	0	0	0	0.101874	0	0	2.01421	0	0	0	0	0	0	0	0	0	0.053478	0		
Bacteria|Proteobacteria|Alphaproteobacteria|Sphingomonadales|Sphingomonadaceae|Sphingomonas|asaccharolytica	0	0	0	0	0	0	0	0	0.646691	0	0.291982	0	0.172722	0	1.580105	0	0.043004	0	0.967156	0	0	0	0	0	0	0	0	0	0	0	0	0	0	0	0	0	0		
Bacteria|Proteobacteria|Alphaproteobacteria|Sphingomonadales|Sphingomonadaceae|Sphingomonas|azotifigens	0	0	0	0	0	0	0	0	0	0	0	0	0	0	0	0	0	0	2.095505	0	0	0	0	0	0	0	0	0	0	0	0	0	0	0	0	0	0		
Bacteria|Proteobacteria|Alphaproteobacteria|Sphingomonadales|Sphingomonadaceae|Sphingomonas|echinoides	0	0	0	0	0	0	0	0	0	0	0	0	0	0	0	0	0	0	0	0	0	0	0	0	0	2.01421	0	0	0	0	0	0	0	0	0	0	0		
Bacteria|Proteobacteria|Alphaproteobacteria|Sphingomonadales|Sphingomonadaceae|Sphingomonas|wittichii	0	0	0	0	0	0	0	0	0	0	0	0	0.043181	0	0.185895	0	0	0	0.752232	0	0	0	0	0	0	0	0	0	0	0	0	0	0	0	0	0	0		
Bacteria|Proteobacteria|Betaproteobacteria	0.51792	0.030131	0.189295	0.126737	0	0	0.028877	9.459673	6.848293	2.598227	0.396262	0.633757	11.11468	1.121655	1.169588	8.99405	7.769458	0.581479	6.205917	21.69096	25.47829	49.12849	19.27277	20.88796	21.24774	23.20646	60.63397	72.41933	33.09796	19.5015	43.37346	68.20495	7.251641	70.98409	83.38932	40.11016	8.557723	Oil sands tailings pond deep	3.88E-07
Bacteria|Proteobacteria|Betaproteobacteria|Burkholderiales	0.51792	0.030131	0.189295	0.126737	0	0	0.028877	8.780031	6.848293	2.052009	0.35455	0.325443	7.052829	1.121655	1.169588	8.221466	6.852031	0.297501	6.205917	21.65582	24.73734	48.28613	18.78038	20.5828	21.24774	23.01364	42.4944	41.37742	31.01587	17.91142	19.03754	65.76558	6.234642	40.78786	46.22502	32.32023	7.415892	Oil sands tailings pond upper	2.63E-07
Bacteria|Proteobacteria|Betaproteobacteria|Burkholderiales|Alcaligenaceae	0	0	0	0	0	0	0	0.094834	0	0.044288	0	0	0	0.170378	0	0.043731	0	0	5.534281	0.685088	0.620329	1.684726	0.458431	0.81375	1.76588	1.4461	0	0	0.34812	0	0.052373	0	0.132652	0	0	0.053478	0	Taiga permafrost layer	0.000179
Bacteria|Proteobacteria|Betaproteobacteria|Burkholderiales|Burkholderiaceae	0	0	0	0	0	0	0	0.829796	6.466911	0.155008	0.041712	0.025693	0.842021	0.894484	1.022421	1.137011	0.043004	0.101421	0.644771	0.628876	0.051694	2.330538	0.203747	0	0.176588	0.335702	0	0	0	0	0.340424	0.212119	0	0	0	0.080217	0	Taiga upper active layer	3.03E-05
Bacteria|Proteobacteria|Betaproteobacteria|Burkholderiales|Burkholderiaceae|Burkholderia	0	0	0	0	0	0	0	0.023708	0.174109	0.044288	0.041712	0	0.453396	0.873187	0.836526	0.109328	0	0.060852	0.29552	0.602527	0	1.984233	0	0	0	0	0	0	0	0	0	0	0	0	0	0	0	Taiga bottom active layer	1.85E-05
Bacteria|Proteobacteria|Betaproteobacteria|Burkholderiales|Burkholderiaceae|Burkholderia|bryophila	0	0	0	0	0	0	0	0.023708	0.049745	0	0	0	0.453396	0.85189	0	0.065597	0	0	0	0	0	0	0	0	0	0	0	0	0	0	0	0	0	0	0	0	0		
Bacteria|Proteobacteria|Betaproteobacteria|Burkholderiales|Burkholderiaceae|Burkholderia|gladioli	0	0	0	0	0	0	0	0	0	0	0	0	0	0	0	0	0	0	0	0.084319	0	0.561575	0	0	0	0	0	0	0	0	0	0	0	0	0	0	0		
Bacteria|Proteobacteria|Betaproteobacteria|Burkholderiales|Burkholderiaceae|Burkholderia|glathei	0	0	0	0	0	0	0	0	0.024873	0.022144	0	0	0	0	0.836526	0.043731	0	0	0.29552	0	0	0	0	0	0	0	0	0	0	0	0	0	0	0	0	0	0		
Bacteria|Proteobacteria|Betaproteobacteria|Burkholderiales|Burkholderiaceae|Burkholderia|tuberum	0	0	0	0	0	0	0	0	0	0	0	0	0	0	0	0	0	0	0	0.491858	0	1.422658	0	0	0	0	0	0	0	0	0	0	0	0	0	0	0		
Bacteria|Proteobacteria|Betaproteobacteria|Burkholderiales|Burkholderiaceae|Lautropia	0	0	0	0	0	0	0	0	0	0	0	0	0	0	0	0	0	0	0	0	0	0.056158	0	0	0	0	0	0	0	0	0	0	0	0	0	0	0		
Bacteria|Proteobacteria|Betaproteobacteria|Burkholderiales|Comamonadaceae	0.51792	0.030131	0.189295	0.126737	0	0	0.028877	7.760567	0.281891	1.203156	0.312838	0.29975	5.462344	0.028396	0.147167	4.679239	6.522331	0.141989	0.026865	12.57752	16.55934	8.517227	10.5524	12.79114	13.00865	14.09086	42.46121	41.37742	30.6346	17.88061	18.60982	65.48276	4.893384	40.78786	46.22502	28.52152	6.099781	Oil sands tailings pond upper	3.56E-07
Bacteria|Proteobacteria|Betaproteobacteria|Burkholderiales|Comamonadaceae|Acidovorax	0	0	0	0	0	0	0	1.161714	0	0	0	0.196979	1.640862	0	0	0.940221	0.989101	0	0	0	0	0	0	0.033906	0	0	0	0	0	0	0	0	0	0	0	0	0		
Bacteria|Proteobacteria|Betaproteobacteria|Burkholderiales|Comamonadaceae|Acidovorax|delafieldii	0	0	0	0	0	0	0	1.06688	0	0	0	0.025693	0	0	0	0.852758	0.989101	0	0	0	0	0	0	0	0	0	0	0	0	0	0	0	0	0	0	0	0		
Bacteria|Proteobacteria|Betaproteobacteria|Burkholderiales|Comamonadaceae|Acidovorax|facilis	0	0	0	0	0	0	0	0.063223	0	0	0	0.171286	1.640862	0	0	0.087462	0	0	0	0	0	0	0	0	0	0	0	0	0	0	0	0	0	0	0	0	0		
Bacteria|Proteobacteria|Betaproteobacteria|Burkholderiales|Comamonadaceae|Delftia	0	0	0	0	0	0	0	0.023708	0	0.155008	0.020856	0	0.194313	0	0.023237	0.109328	0.88159	0.020284	0.026865	5.164513	6.616843	1.684726	3.896661	4.24676	5.322866	6.016807	0	0	0	0	0	0	0	0	0	0	0	Oil sands core	4.34E-08
Bacteria|Proteobacteria|Betaproteobacteria|Burkholderiales|Comamonadaceae|Hydrogenophaga	0	0	0	0	0	0	0	0	0	0	0	0	0	0	0	0	0.021502	0	0	0	0	0.280788	0	0	0.050454	0	0.024894	0.027505	2.586037	1.594694	0.157119	3.63253	0.442173	0	0	0	0.135217		
Bacteria|Proteobacteria|Betaproteobacteria|Burkholderiales|Comamonadaceae|Hydrogenophaga|palleronii	0	0	0	0	0	0	0	0	0	0	0	0	0	0	0	0	0	0	0	0	0	0	0	0	0.050454	0	0	0.027505	1.566542	1.155576	0.157119	3.606015	0.442173	0	0	0	0.135217		
Bacteria|Proteobacteria|Betaproteobacteria|Burkholderiales|Comamonadaceae|Limnohabitans	0	0	0	0	0	0	0	0	0	0	0.041712	0	0	0	0	0	3.010307	0	0	0	0	0	0	0	0	0	0	0	0	0	0	0	0	0	0	0	0		
Bacteria|Proteobacteria|Betaproteobacteria|Burkholderiales|Comamonadaceae|Methylibium	0	0	0	0	0	0	0	0	0	0	0	0	0	0	0	0	0	0	0	0	0	0	0	0	0	0	0	0	0	0	0	0	0	0	0	0	0	India oil refineries	1.17E-07
Bacteria|Proteobacteria|Betaproteobacteria|Burkholderiales|Comamonadaceae|Methylibium|petroleiphilum	0	0	0	0	0	0	0	0	0	0	0	0	0	0	0	0	0	0	0	0	0	0	0	0	0	0	0	0	0	0	0	0	0	0	0	0	0	India oil refineries	1.49E-05
Bacteria|Proteobacteria|Betaproteobacteria|Burkholderiales|Comamonadaceae|Polaromonas	0	0	0	0	0	0	0	0.142251	0	0	0	0	0.086361	0	0.046474	0.043731	0	0	0	0	0	0	0	0	0	0	2.588997	3.080615	9.100863	1.756475	7.175082	0.05303	0.530608	3.854838	0	2.887803	0	Oil sands tailings pond upper	1.64E-05
Bacteria|Proteobacteria|Betaproteobacteria|Burkholderiales|Comamonadaceae|Ramlibacter	0	0	0	0	0	0	0	0	0.049745	0.752895	0.250271	0	0.129542	0	0.046474	0.262387	0	0	0	0	0	0	0	0	0	0	0	0	0	0	0	0	0	0	0	0	0		
Bacteria|Proteobacteria|Betaproteobacteria|Burkholderiales|Comamonadaceae|Rhodoferax	0	0	0	0	0	0	0	1.043172	0	0	0	0	2.97946	0	0	0.874624	0.946096	0	0	0	0	0	0	0	0	0	15.38462	0.275055	15.66542	12.89622	0	22.11336	0	0.055069	0.230357	0	0	Oil sands tailings pond upper	0.001127
Bacteria|Proteobacteria|Betaproteobacteria|Burkholderiales|Comamonadaceae|Tepidimonas	0	0	0	0	0	0	0	0	0	0	0	0	0	0	0	0	0	0	0	0	0	0	0	0.134543	0	0	0	0	0.696241	0.184892	0	0.035353	0	0	0	0	0		
Bacteria|Proteobacteria|Betaproteobacteria|Burkholderiales|Comamonadaceae|Thiomonas	0	0	0	0	0	0	0	0	0	0	0	0	0	0	0	0	0	0	0	0	0	0	0	0	0	0	0	0	0	0	0	0	0	0	0	0	0		
Bacteria|Proteobacteria|Betaproteobacteria|Burkholderiales|Comamonadaceae|Variovorax	0	0	0	0	0	0	0	0.758671	0	0.088576	0	0	0.043181	0	0	0.306118	0.129013	0.040568	0	0	0	0	0	1.322344	0	0.103293	0	0	0	0	0	0	0	0	0	0	0		
Bacteria|Proteobacteria|Betaproteobacteria|Burkholderiales|Comamonadaceae|Variovorax|paradoxus	0	0	0	0	0	0	0	0.758671	0	0.088576	0	0	0.043181	0	0	0.306118	0.129013	0.040568	0	0	0	0	0	1.322344	0	0.103293	0	0	0	0	0	0	0	0	0	0	0		
Bacteria|Proteobacteria|Betaproteobacteria|Burkholderiales|Oxalobacteraceae	0	0	0	0	0	0	0	0.094834	0.099491	0.649557	0	0	0.748463	0.028396	0	2.361485	0.286696	0.054091	0	7.764336	7.505982	35.75363	7.565805	6.97791	6.296622	7.140977	0.033192	0	0.033154	0.030815	0.034915	0.070706	1.208607	0	0	3.665015	1.31611	Oil sands core	3.22E-05
Bacteria|Proteobacteria|Betaproteobacteria|Burkholderiales|Oxalobacteraceae|Cupriavidus	0	0	0	0	0	0	0	0.031611	0.099491	0.620031	0	0	0.690889	0.028396	0	2.128252	0.200687	0.054091	0	5.867168	6.437637	3.069946	6.309366	4.70619	5.220276	5.212845	0	0	0	0	0.034915	0.070706	1.208607	0	0	3.023281	1.135821	Oil sands core	4.09E-05
Bacteria|Proteobacteria|Betaproteobacteria|Burkholderiales|Oxalobacteraceae|Cupriavidus|gilardii	0	0	0	0	0	0	0	0	0	0	0	0	0	0	0	0	0	0	0	5.059115	5.024665	1.909356	5.154799	4.43494	4.984826	5.143983	0	0	0	0	0	0	0.884346	0	0	2.951977	1.135821	Oil sands core	1.37E-07
Bacteria|Proteobacteria|Betaproteobacteria|Burkholderiales|Oxalobacteraceae|Herminiimonas	0	0	0	0	0	0	0	0	0	0.029525	0	0	0.028787	0	0	0.058308	0	0	0	0.105398	0	0	0.033958	2.136095	1.04271	1.790409	0	0	0.033154	0	0	0	0	0	0	0.57043	0.180289		
Bacteria|Proteobacteria|Betaproteobacteria|Burkholderiales|Oxalobacteraceae|Janthinobacterium	0	0	0	0	0	0	0	0	0	0	0	0	0.028787	0	0	0.174925	0.086009	0	0	0	0	0	0	0	0	0	0	0	0	0	0	0	0	0	0	0	0	China oil refineries	8.23E-08
Bacteria|Proteobacteria|Betaproteobacteria|Burkholderiales|Oxalobacteraceae|Ralstonia	0	0	0	0	0	0	0	0	0	0	0	0	0	0	0	0	0	0	0	1.79177	1.068345	32.30931	1.222482	0.135625	0.033636	0	0	0	0	0	0	0	0	0	0	0.071304	0		
Bacteria|Proteobacteria|Betaproteobacteria|Ellin6067	0	0	0	0	0	0	0	0.063223	0	0	0	0	0	0	0	0.029154	0	0	0	0	0	0	0	0	0	0	0	0	0	0	0.034915	0	0	0	0	0	0		
Bacteria|Proteobacteria|Betaproteobacteria|Hydrogenophilales	0	0	0	0	0	0	0	0	0	0	0	0	0	0	0	0.087462	0	0	0	0	0	0.074877	0	0	0	0	1.394075	4.180834	0.099463	0	2.93288	0	0.206348	0	4.991074	0.855645	0	India oil refineries	0.000337
Bacteria|Proteobacteria|Betaproteobacteria|Hydrogenophilales|Hydrogenophilaceae	0	0	0	0	0	0	0	0	0	0	0	0	0	0	0	0.087462	0	0	0	0	0	0.074877	0	0	0	0	1.394075	4.180834	0.099463	0	2.93288	0	0.206348	0	4.991074	0.855645	0	India oil refineries	0.000337
Bacteria|Proteobacteria|Betaproteobacteria|Hydrogenophilales|Hydrogenophilaceae|Thiobacillus	0	0	0	0	0	0	0	0	0	0	0	0	0	0	0	0.087462	0	0	0	0	0	0.074877	0	0	0	0	1.394075	4.180834	0.099463	0	2.93288	0	0.206348	0	4.991074	0.855645	0	India oil refineries	0.000337
Bacteria|Proteobacteria|Betaproteobacteria|MND1	0	0	0	0	0	0	0	0	0	0.029525	0	0	0	0	0	0	0	0	0	0	0	0	0	0	0	0	0	0	0	0	0	0	0	0	0	0	0		
Bacteria|Proteobacteria|Betaproteobacteria|MOB121	0	0	0	0	0	0	0	0	0	0	0	0	0	0	0	0	0	0	0	0	0	0	0	0	0	0	0	0	0.198926	0.046223	0	0	0	0	0	0	0		
Bacteria|Proteobacteria|Betaproteobacteria|Nitrosomonadales	0	0	0	0	0	0	0	0	0	0	0	0	0	0	0	0	0	0	0	0	0	0	0	0	0	0	0	0	0	0	0	0	0	0	0	0	0		
Bacteria|Proteobacteria|Betaproteobacteria|Nitrosomonadales|Nitrosomonadaceae	0	0	0	0	0	0	0	0	0	0	0	0	0	0	0	0	0	0	0	0	0	0	0	0	0	0	0	0	0	0	0	0	0	0	0	0	0		
Bacteria|Proteobacteria|Betaproteobacteria|Rhodocyclales	0	0	0	0	0	0	0	0.442558	0	0.044288	0	0.102771	3.742317	0	0	0.539351	0.917427	0	0	0.035133	0.740949	0.711329	0.441452	0.305156	0	0.172155	16.64592	26.84536	1.525099	1.448322	21.36813	2.103509	0.766434	30.01267	31.86609	6.845164	1.141831	Oil sands tailings pond deep	2.09E-05
Bacteria|Proteobacteria|Betaproteobacteria|Rhodocyclales|Rhodocyclaceae	0	0	0	0	0	0	0	0.442558	0	0.044288	0	0.102771	3.742317	0	0	0.539351	0.917427	0	0	0.035133	0.740949	0.711329	0.441452	0.305156	0	0.172155	16.64592	26.84536	1.525099	1.448322	21.36813	2.103509	0.766434	30.01267	31.86609	6.845164	1.141831	Oil sands tailings pond deep	2.09E-05
Bacteria|Proteobacteria|Betaproteobacteria|Rhodocyclales|Rhodocyclaceae|Azospira	0	0	0	0	0	0	0	0	0	0	0	0	0.777251	0	0	0	0.086009	0	0	0	0	0	0	0	0	0	0	0	0	0	0	0	0	0	0	0	0		
Bacteria|Proteobacteria|Betaproteobacteria|Rhodocyclales|Rhodocyclaceae|Azovibrio	0	0	0	0	0	0	0	0.189668	0	0	0	0	1.727223	0	0	0	0.774079	0	0	0	0	0	0	0	0	0	0	0	0	0	0	0	0	0	0	0	0.090145		
Bacteria|Proteobacteria|Betaproteobacteria|Rhodocyclales|Rhodocyclaceae|Dechloromonas	0	0	0	0	0	0	0	0	0	0	0	0	0	0	0	0	0	0	0	0	0.568635	0	0	0.305156	0	0	0	0	0.099463	0	3.561355	0.05303	0	0.055069	0	0	0		
Bacteria|Proteobacteria|Betaproteobacteria|Rhodocyclales|Rhodocyclaceae|Denitratisoma	0	0	0	0	0	0	0	0	0	0	0	0	0	0	0	0	0	0	0	0	0	0	0	0	0	0	0	0	0	0	0	0	0	0	0	0	0		
Bacteria|Proteobacteria|Betaproteobacteria|Rhodocyclales|Rhodocyclaceae|Sulfuritalea	0	0	0	0	0	0	0	0.031611	0	0	0	0.102771	0	0	0	0.058308	0.057339	0	0	0	0	0	0	0	0	0	11.21899	24.68159	0.165772	0.092446	0	1.37877	0	0.036713	27.29733	0.071304	0.150241		
Bacteria|Proteobacteria|Betaproteobacteria|Rhodocyclales|Rhodocyclaceae|Thauera	0	0	0	0	0	0	0	0	0	0	0	0	0	0	0	0	0	0	0	0	0	0.486699	0.033958	0	0	0	1.626421	0.806828	0.99463	0.677938	12.22034	0.388884	0.736955	0.110138	1.650894	0	0.811301	Oil sands tailings pond median	3.66E-07
Bacteria|Proteobacteria|Betaproteobacteria|SBla14	0	0	0	0	0	0	0	0	0	0	0	0	0	0	0	0	0	0	0	0	0	0	0	0	0	0	0	0	0	0	0	0	0	0	0	0	0		
Bacteria|Proteobacteria|Deltaproteobacteria	4.234762	4.361516	10.82925	6.273502	8.441336	2.911772	4.15824	0.316113	0	0.044288	0	0	0.863612	0	0	0.371715	0.458713	0	0	0.500642	1.13727	0.814284	1.298887	1.424063	0.63908	0.697227	11.08622	7.463156	12.36656	17.54164	23.3583	5.98351	18.37967	9.637095	6.046878	7.112553	19.81678	Oil sands tailings pond median	3.56E-08
Bacteria|Proteobacteria|Deltaproteobacteria|Bdellovibrionales	0.182795	0.723153	0.189295	0.807951	0.095472	0.345953	0.476465	0.047417	0	0	0	0	0.259084	0	0	0.131194	0.387039	0	0	0	0.051694	0	0	0	0	0	0	0	0	0	0	0	0	0	0	0	0		
Bacteria|Proteobacteria|Deltaproteobacteria|Bdellovibrionales|Bacteriovoracaceae	0.137097	0.723153	0.189295	0.807951	0.095472	0.345953	0.476465	0.047417	0	0	0	0	0.259084	0	0	0.131194	0.387039	0	0	0	0.051694	0	0	0	0	0	0	0	0	0	0	0	0	0	0	0	0		
Bacteria|Proteobacteria|Deltaproteobacteria|Bdellovibrionales|Bacteriovoracaceae|Bacteriovorax	0.091398	0.723153	0.094647	0.617845	0.047736	0.302709	0.389835	0	0	0	0	0	0	0	0	0	0	0	0	0	0	0	0	0	0	0	0	0	0	0	0	0	0	0	0	0	0		
Bacteria|Proteobacteria|Deltaproteobacteria|Desulfarculales	0	0	0	0.031684	0	0	0.028877	0	0	0	0	0	0	0	0	0	0	0	0	0	0	0	0	0	0	0	0.066385	0	0.033154	0	0.384068	0.070706	0.235826	0	0	0.142608	0.240385		
Bacteria|Proteobacteria|Deltaproteobacteria|Desulfarculales|Desulfarculaceae	0	0	0	0.031684	0	0	0.028877	0	0	0	0	0	0	0	0	0	0	0	0	0	0	0	0	0	0	0	0.066385	0	0.033154	0	0.384068	0.070706	0.235826	0	0	0.142608	0.240385		
Bacteria|Proteobacteria|Deltaproteobacteria|Desulfobacterales	0.213261	1.175124	3.423083	1.916903	5.171412	1.00903	0.924053	0	0	0	0	0	0	0	0	0	0.02867	0	0	0	0	0.112315	0	0	0	0	0.796614	0.513436	2.834695	4.529857	3.142372	2.792894	3.065734	0.055069	0.057589	2.424329	4.22177	Mangrove	2.88E-07
Bacteria|Proteobacteria|Deltaproteobacteria|Desulfobacterales|Desulfobacteraceae	0.030466	0.150657	0.063098	0.22179	0.445537	0	0.404273	0	0	0	0	0	0	0	0	0	0	0	0	0	0	0	0	0	0	0	0	0	0.099463	1.340468	0.244407	0	0.132652	0	0	0.053478	0.090145		
Bacteria|Proteobacteria|Deltaproteobacteria|Desulfobacterales|Desulfobacteraceae|Desulfobacter	0	0	0	0	0	0	0	0	0	0	0	0	0	0	0	0	0	0	0	0	0	0	0	0	0	0	0	0	0.099463	1.340468	0.209491	0	0.044217	0	0	0.053478	0.090145		
Bacteria|Proteobacteria|Deltaproteobacteria|Desulfobacterales|Desulfobacteraceae|Desulfosarcina	0	0	0	0	0	0	0	0	0	0	0	0	0	0	0	0	0	0	0	0	0	0	0	0	0	0	0	0	0	0	0.034915	0	0.088435	0	0	0	0		
Bacteria|Proteobacteria|Deltaproteobacteria|Desulfobacterales|Desulfobulbaceae	0.091398	1.024467	3.265337	1.695113	4.630403	1.00903	0.43315	0	0	0	0	0	0	0	0	0	0.02867	0	0	0	0	0.112315	0	0	0	0	0.796614	0.513436	2.735232	3.189389	2.897965	2.792894	2.933082	0.055069	0.057589	2.370851	4.131625	Oil sands tailings pond median	3.31E-07
Bacteria|Proteobacteria|Deltaproteobacteria|Desulfobacterales|Desulfobulbaceae|Desulfobulbus	0	0	0.047324	0.237633	0.047736	0	0	0	0	0	0	0	0	0	0	0	0	0	0	0	0	0.112315	0	0	0	0	0	0	0.248657	0.323561	0.73322	0	0.309521	0.055069	0.057589	0.160434	0.40565		
Bacteria|Proteobacteria|Deltaproteobacteria|Desulfobacterales|Desulfobulbaceae|Desulfocapsa	0.030466	0	3.028719	0.22179	0.763778	0.432441	0.028877	0	0	0	0	0	0	0	0	0	0	0	0	0	0	0	0	0	0	0	0	0	0	0	0	0	0	0	0	0	0		
Bacteria|Proteobacteria|Deltaproteobacteria|Desulfobacterales|Desulfobulbaceae|Desulfotalea	0.060932	0.934073	0	0.697056	0.350065	0.576588	0.144383	0	0	0	0	0	0	0	0	0	0	0	0	0	0	0	0	0	0	0	0	0	0	0	0	0	0	0	0	0	0		
Bacteria|Proteobacteria|Deltaproteobacteria|Desulfuromonadales	0.228494	1.898276	4.306459	1.853535	2.195861	1.124347	2.07912	0.047417	0	0	0	0	0.302264	0	0	0	0	0	0	0	0	0.22463	0	0	0	0	1.792382	0.990198	3.68013	3.420504	12.72661	1.856037	1.282302	8.866127	3.628127	0.855645	1.802891	Oil sands tailings pond median	5.92E-07
Bacteria|Proteobacteria|Deltaproteobacteria|Desulfuromonadales|Desulfuromonadaceae	0.137097	0.632759	3.265337	1.140637	0.572833	0.605418	1.169505	0	0	0	0	0	0	0	0	0	0	0	0	0	0	0	0	0	0	0	0.049788	0.055011	1.243287	0.416007	0.052373	0.477267	0.353739	0.055069	0.115179	0.106956	0.40565	Mangrove	1.18E-08
Bacteria|Proteobacteria|Deltaproteobacteria|Desulfuromonadales|Geobacteraceae	0.091398	1.265518	1.041122	0.712898	1.623028	0.51893	0.909615	0.047417	0	0	0	0	0.302264	0	0	0	0	0	0	0	0	0.22463	0	0	0	0	1.24471	0.880176	2.287649	2.773382	2.723389	1.325741	0.751694	0.991244	1.439733	0.74869	1.171879	Oil sands tailings pond upper	1.52E-06
Bacteria|Proteobacteria|Deltaproteobacteria|Desulfuromonadales|Geobacteraceae|Geobacter	0.091398	1.265518	1.041122	0.712898	1.623028	0.51893	0.909615	0.047417	0	0	0	0	0.302264	0	0	0	0	0	0	0	0	0.22463	0	0	0	0	1.24471	0.880176	2.287649	2.773382	2.723389	1.325741	0.751694	0.991244	1.439733	0.74869	1.171879	Oil sands tailings pond upper	1.52E-06
Bacteria|Proteobacteria|Deltaproteobacteria|Desulfuromonadales|Geobacteraceae|Geobacter|lovleyi	0	0	0	0	0	0	0	0	0	0	0	0	0	0	0	0	0	0	0	0	0	0.112315	0	0	0	0	0.149365	0	0.397852	1.06313	0.785593	0.106059	0.265304	0.055069	0.057589	0.481301	0.360578		
Bacteria|Proteobacteria|Deltaproteobacteria|Desulfuromonadales|Pelobacteraceae	0	0	0	0	0	0	0	0	0	0	0	0	0	0	0	0	0	0	0	0	0	0	0	0	0	0	0.497884	0.055011	0.149194	0.231115	9.950844	0.05303	0.176869	7.819814	2.073215	0	0.225361		
Bacteria|Proteobacteria|Deltaproteobacteria|Desulfuromonadales|Pelobacteraceae|Pelobacter	0	0	0	0	0	0	0	0	0	0	0	0	0	0	0	0	0	0	0	0	0	0	0	0	0	0	0.448096	0.055011	0.149194	0.231115	9.950844	0.05303	0.176869	7.819814	2.073215	0	0.225361		
Bacteria|Proteobacteria|Deltaproteobacteria|Myxococcales	0.319892	0.361576	0.615208	0.427739	0.238681	0.302709	0.17326	0.189668	0	0.044288	0	0	0.043181	0	0	0.174925	0	0	0	0.474292	1.085576	0.393103	1.273419	1.424063	0.605444	0.619757	0.099577	0.605121	0	0	0	0	0	0.495622	2.073215	1.49738	0.180289	China oil refineries	6.52E-07
Bacteria|Proteobacteria|Deltaproteobacteria|Myxococcales|0319-6G20	0	0	0	0	0	0	0	0	0	0	0	0	0	0	0	0	0	0	0	0.316195	0.206776	0.056158	0.560304	0.508594	0.252269	0.154939	0	0	0	0	0	0	0	0	0	0.427823	0.135217		
Bacteria|Proteobacteria|Deltaproteobacteria|Myxococcales|Haliangiaceae	0.045699	0.045197	0.141971	0	0.047736	0	0.043315	0.189668	0	0	0	0	0	0	0	0.087462	0	0	0	0	0	0	0	0	0	0	0.049788	0.605121	0	0	0	0	0	0.495622	1.900447	0	0		
Bacteria|Proteobacteria|Deltaproteobacteria|Myxococcales|Myxococcaceae	0	0	0	0	0	0	0	0	0	0	0	0	0.043181	0	0	0.043731	0	0	0	0	0	0	0	0	0	0	0	0	0	0	0	0	0	0	0	0	0	China oil refineries	1.1E-06
Bacteria|Proteobacteria|Deltaproteobacteria|Myxococcales|Myxococcaceae|Anaeromyxobacter	0	0	0	0	0	0	0	0	0	0	0	0	0.043181	0	0	0.043731	0	0	0	0	0	0	0	0	0	0	0	0	0	0	0	0	0	0	0	0	0	China oil refineries	1.1E-06
Bacteria|Proteobacteria|Deltaproteobacteria|Myxococcales|Polyangiaceae	0	0	0	0	0	0	0	0	0	0	0	0	0	0	0	0	0	0	0	0	0	0	0.101874	0	0	0.051646	0	0	0	0	0	0	0	0	0	0.962601	0		
Bacteria|Proteobacteria|Deltaproteobacteria|NB1-j	2.696233	0.045197	1.514359	0.665371	0.477361	0.086488	0.389835	0	0	0	0	0	0	0	0	0	0	0	0	0	0	0	0	0	0	0	0	0	0	0	0	0	0	0	0	0	0		

Table S2. V

Bacterial clades	Habitat															
	India oil refineries				Arctic											
	I1	I2	I3	I4	A1	A10	A11	A12	A2	A3	A4	A5	A6	A7	A8	A9
Bacteria|Proteobacteria|Deltaproteobacteria|NB1-j|NB1-i	0	0.038732	0	0	0	0	0	0	0	0	0	0	0	0	0	0
Bacteria|Proteobacteria|Deltaproteobacteria|Sva0853	0	0	0	0	0	0	0	0	0	0	0	0	0	0	0	0
Bacteria|Proteobacteria|Deltaproteobacteria|Sva0853|JTB36	0	0	0	0	0	0	0	0	0	0	0	0	0	0	0	0
Bacteria|Proteobacteria|Deltaproteobacteria|Syntrophobacterales	0.035575	0.800454	2.520674	0.255979	0	0	0	0	0	0	0	0	0	0	0	0
Bacteria|Proteobacteria|Deltaproteobacteria|Syntrophobacterales|Syntrophaceae	0	0.142016	2.086075	0	0	0	0	0	0	0	0	0	0	0	0	0
Bacteria|Proteobacteria|Deltaproteobacteria|Syntrophobacterales|Syntrophaceae|Syntrophus	0	0.077463	1.955696	0	0	0	0	0	0	0	0	0	0	0	0	0
Bacteria|Proteobacteria|Deltaproteobacteria|Syntrophobacterales|Syntrophobacteraceae	0.035575	0.658438	0.347679	0.255979	0	0	0	0	0	0	0	0	0	0	0	0
Bacteria|Proteobacteria|Deltaproteobacteria|Syntrophobacterales|Syntrophobacteraceae|Desulfacinum	0	0	0	0	0	0	0	0	0	0	0	0	0	0	0	0
Bacteria|Proteobacteria|Deltaproteobacteria|Syntrophobacterales|Syntrophobacteraceae|Syntrophobacter	0	0.542243	0.08692	0	0	0	0	0	0	0	0	0	0	0	0	0
Bacteria|Proteobacteria|Deltaproteobacteria|Syntrophobacterales|Syntrophorhabdaceae	0	0	0.08692	0	0	0	0	0	0	0	0	0	0	0	0	0
Bacteria|Proteobacteria|Epsilonproteobacteria	0.047434	0.103284	0.260759	0.199095	0	0	0	0	0	0	0	0	0	0	0	0
Bacteria|Proteobacteria|Epsilonproteobacteria|Campylobacterales	0.047434	0.103284	0.260759	0.199095	0	0	0	0	0	0	0	0	0	0	0	0
Bacteria|Proteobacteria|Epsilonproteobacteria|Campylobacterales|Campylobacteraceae	0	0	0.028973	0	0	0	0	0	0	0	0	0	0	0	0	0
Bacteria|Proteobacteria|Epsilonproteobacteria|Campylobacterales|Campylobacteraceae|Arcobacter	0	0	0	0	0	0	0	0	0	0	0	0	0	0	0	0
Bacteria|Proteobacteria|Epsilonproteobacteria|Campylobacterales|Helicobacteraceae	0.047434	0.103284	0.231786	0.199095	0	0	0	0	0	0	0	0	0	0	0	0
Bacteria|Proteobacteria|Gammaproteobacteria	26.34954	11.03853	21.51265	28.07236	2.467986	5.706695	8.934917	12.38402	5.59682	3.729614	2.668975	9.316483	2.502703	8.705342	1.800167	13.96896
Bacteria|Proteobacteria|Gammaproteobacteria|Alteromonadales	0	0	0	0	0	0	0	0.076131	0	0	0	0	0	0	0	0
Bacteria|Proteobacteria|Gammaproteobacteria|Alteromonadales|Alteromonadaceae	0	0	0	0	0	0	0	0	0	0	0	0	0	0	0	0
Bacteria|Proteobacteria|Gammaproteobacteria|Alteromonadales|Alteromonadaceae|Cellvibrio	0	0	0	0	0	0	0	0	0	0	0	0	0	0	0	0
Bacteria|Proteobacteria|Gammaproteobacteria|Alteromonadales|Alteromonadaceae|HB2-32-21	0	0	0	0	0	0	0	0	0	0	0	0	0	0	0	0
Bacteria|Proteobacteria|Gammaproteobacteria|Alteromonadales|Alteromonadaceae|Marinobacter	0	0	0	0	0	0	0	0	0	0	0	0	0	0	0	0
Bacteria|Proteobacteria|Gammaproteobacteria|Alteromonadales|Alteromonadaceae|ZD0117	0	0	0	0	0	0	0	0	0	0	0	0	0	0	0	0
Bacteria|Proteobacteria|Gammaproteobacteria|Alteromonadales|Colwelliaceae	0	0	0	0	0	0	0	0	0	0	0	0	0	0	0	0
Bacteria|Proteobacteria|Gammaproteobacteria|Alteromonadales|Colwelliaceae|Colwellia	0	0	0	0	0	0	0	0	0	0	0	0	0	0	0	0
Bacteria|Proteobacteria|Gammaproteobacteria|Alteromonadales|OM60	0	0	0	0	0	0	0	0	0	0	0	0	0	0	0	0
Bacteria|Proteobacteria|Gammaproteobacteria|Alteromonadales|OM60|Congregibacter	0	0	0	0	0	0	0	0	0	0	0	0	0	0	0	0
Bacteria|Proteobacteria|Gammaproteobacteria|Chromatiales	2.454705	1.665462	11.95147	8.831273	0	0	0	0	0	0	0	0	0	0	0	0
Bacteria|Proteobacteria|Gammaproteobacteria|Chromatiales|Ectothiorhodospiraceae	2.454705	1.62673	11.95147	8.831273	0	0	0	0	0	0	0	0	0	0	0	0
Bacteria|Proteobacteria|Gammaproteobacteria|Enterobacteriales	0	0	0	0	0.217307	0	0	0	0	0	0	0	0	0	0	0
Bacteria|Proteobacteria|Gammaproteobacteria|Enterobacteriales|Enterobacteriaceae	0	0	0	0	0.217307	0	0	0	0	0	0	0	0	0	0	0
Bacteria|Proteobacteria|Gammaproteobacteria|Enterobacteriales|Enterobacteriaceae|Serratia	0	0	0	0	0	0	0	0	0	0	0	0	0	0	0	0
Bacteria|Proteobacteria|Gammaproteobacteria|Enterobacteriales|Enterobacteriaceae|Serratia|symbiotica	0	0	0	0	0	0	0	0	0	0	0	0	0	0	0	0
Bacteria|Proteobacteria|Gammaproteobacteria|Legionellales	0	0	0	0	0.093132	0.085175	0	0.095164	0	0	0	0	0.091562	0	0.041864	0
Bacteria|Proteobacteria|Gammaproteobacteria|Legionellales|Legionellaceae	0	0	0	0	0.093132	0.085175	0	0.047582	0	0	0	0	0.045781	0	0.041864	0
Bacteria|Proteobacteria|Gammaproteobacteria|Legionellales|Legionellaceae|Legionella	0	0	0	0	0	0.042587	0	0.047582	0	0	0	0	0	0	0.041864	0
Bacteria|Proteobacteria|Gammaproteobacteria|Marinicellales	0	0	0	0	0	0	0	0	0	0	0	0	0	0	0	0
Bacteria|Proteobacteria|Gammaproteobacteria|Marinicellales|Marinicellaceae	0	0	0	0	0	0	0	0	0	0	0	0	0	0	0	0
Bacteria|Proteobacteria|Gammaproteobacteria|Marinicellales|Marinicellaceae|Marinicella	0	0	0	0	0	0	0	0	0	0	0	0	0	0	0	0
Bacteria|Proteobacteria|Gammaproteobacteria|Methylococcales	0	0	0	0	0	0	0	0	0	0	0	0	0	0	0	0
Bacteria|Proteobacteria|Gammaproteobacteria|Methylococcales|Crenotrichaceae	0	0	0	0	0	0	0	0	0	0	0	0	0	0	0	0
Bacteria|Proteobacteria|Gammaproteobacteria|Methylococcales|Crenotrichaceae|Crenothrix	0	0	0	0	0	0	0	0	0	0	0	0	0	0	0	0
Bacteria|Proteobacteria|Gammaproteobacteria|Oceanospirillales	0	0	0	0.170653	0	0	0	0	0	0	0	0	0	0	0	0
Bacteria|Proteobacteria|Gammaproteobacteria|Oceanospirillales|Halomonadaceae	0	0	0	0	0	0	0	0	0	0	0	0	0	0	0	0
Bacteria|Proteobacteria|Gammaproteobacteria|Oceanospirillales|Halomonadaceae|Halomonas	0	0	0	0	0	0	0	0	0	0	0	0	0	0	0	0
Bacteria|Proteobacteria|Gammaproteobacteria|Pseudomonadales	0	0	0	0	0.015522	0	0.098186	0.079303	0.040557	0.031341	0	0.129396	0.030521	0	0	0.041574
Bacteria|Proteobacteria|Gammaproteobacteria|Pseudomonadales|Moraxellaceae	0	0	0	0	0	0	0	0	0	0	0	0	0	0	0	0
Bacteria|Proteobacteria|Gammaproteobacteria|Pseudomonadales|Moraxellaceae|Acinetobacter	0	0	0	0	0	0	0	0	0	0	0	0	0	0	0	0
Bacteria|Proteobacteria|Gammaproteobacteria|Pseudomonadales|Moraxellaceae|Acinetobacter|rhizosphaerae	0	0	0	0	0	0	0	0	0	0	0	0	0	0	0	0
Bacteria|Proteobacteria|Gammaproteobacteria|Pseudomonadales|Moraxellaceae|Alkanindiges	0	0	0	0	0	0	0	0	0	0	0	0	0	0	0	0
Bacteria|Proteobacteria|Gammaproteobacteria|Pseudomonadales|Moraxellaceae|Perlucidibaca	0	0	0	0	0	0	0	0	0	0	0	0	0	0	0	0
Bacteria|Proteobacteria|Gammaproteobacteria|Pseudomonadales|Pseudomonadaceae	0	0	0	0	0.015522	0	0.098186	0.079303	0.040557	0.031341	0	0.129396	0.030521	0	0	0.041574
Bacteria|Proteobacteria|Gammaproteobacteria|Pseudomonadales|Pseudomonadaceae|Pseudomonas	0	0	0	0	0.015522	0	0.098186	0.079303	0.040557	0.031341	0	0.129396	0.030521	0	0	0.041574
Bacteria|Proteobacteria|Gammaproteobacteria|Pseudomonadales|Pseudomonadaceae|Pseudomonas|stutzeri	0	0	0	0	0	0	0	0	0	0	0	0	0	0	0	0
Bacteria|Proteobacteria|Gammaproteobacteria|Pseudomonadales|Pseudomonadaceae|Pseudomonas|viridiflava	0	0	0	0	0	0	0	0	0	0	0	0	0	0	0	0
Bacteria|Proteobacteria|Gammaproteobacteria|PYR10d3	0	0	0	0	0	0	0	0	0	0	0	0	0	0	0	0
Bacteria|Proteobacteria|Gammaproteobacteria|Thiotrichales	0	0	0	0	0	0	0	0	0	0	0	0	0	0	0	0
Bacteria|Proteobacteria|Gammaproteobacteria|Thiotrichales|Piscirickettsiaceae	0	0	0	0	0	0	0	0	0	0	0	0	0	0	0	0
Bacteria|Proteobacteria|Gammaproteobacteria|Thiotrichales|Thiotrichaceae	0	0	0	0	0	0	0	0	0	0	0	0	0	0	0	0
Bacteria|Proteobacteria|Gammaproteobacteria|Xanthomonadales	23.85926	9.373063	9.561179	19.07043	2.142026	5.621521	8.836731	12.13342	5.556264	3.698273	2.668975	9.187088	2.38062	8.705342	1.758303	13.92738
Bacteria|Proteobacteria|Gammaproteobacteria|Xanthomonadales|Sinobacteraceae	7.79102	3.563313	5.475948	10.49514	0	0	0	0.23791	2.717297	0.470119	0.368134	0.733242	0	0.280817	0.96288	1.995565
Bacteria|Proteobacteria|Gammaproteobacteria|Xanthomonadales|Sinobacteraceae|Hydrocarboniphaga	0	0	0	0	0	0	0	0	0	0	0	0	0	0	0	0
Bacteria|Proteobacteria|Gammaproteobacteria|Xanthomonadales|Sinobacteraceae|Hydrocarboniphaga|effusa	0	0	0	0	0	0	0	0	0	0	0	0	0	0	0	0
Bacteria|Proteobacteria|Gammaproteobacteria|Xanthomonadales|Sinobacteraceae|Nevskia	0.071151	0	0.13038	0.127989	0	0	0	0	0	0	0	0	0	0	0	0
Bacteria|Proteobacteria|Gammaproteobacteria|Xanthomonadales|Xanthomonadaceae	16.06824	5.80975	4.085231	8.575294	2.142026	5.621521	8.836731	11.89551	2.838967	3.228153	2.30084	8.453846	2.38062	8.424525	0.795423	11.93182
Bacteria|Proteobacteria|Gammaproteobacteria|Xanthomonadales|Xanthomonadaceae|Arenimonas	0	0	0.04346	0	0.046566	0.042587	1.080045	1.427461	0	0	0.13805	0	0	0.280817	0	0
Bacteria|Proteobacteria|Gammaproteobacteria|Xanthomonadales|Xanthomonadaceae|Dokdonella	0.035575	0	0.08692	0.085326	0	0	0	0.047582	0	0.094024	0	0	0	0.046803	0	0
Bacteria|Proteobacteria|Gammaproteobacteria|Xanthomonadales|Xanthomonadaceae|Dyella	7.541992	2.014047	0	0.554621	0	0	0	0	0	0	0	0	0	0	0	0
Bacteria|Proteobacteria|Gammaproteobacteria|Xanthomonadales|Xanthomonadaceae|Dyella|ginsengisoli	7.541992	2.014047	0	0.554621	0	0	0	0	0	0	0	0	0	0	0	0
Bacteria|Proteobacteria|Gammaproteobacteria|Xanthomonadales|Xanthomonadaceae|Luteibacter	0	0	0	0	0	0	0	0	0	0	0	1.423352	0	0	0	0
Bacteria|Proteobacteria|Gammaproteobacteria|Xanthomonadales|Xanthomonadaceae|Luteibacter|rhizovicinus	0	0	0	0	0	0	0	0	0	0	0	1.423352	0	0	0	0
Bacteria|Proteobacteria|Gammaproteobacteria|Xanthomonadales|Xanthomonadaceae|Luteimonas	2.0278	0	0.304219	0.255979	0	0	0	0	0	0	0	0	0	0	0	0
Bacteria|Proteobacteria|Gammaproteobacteria|Xanthomonadales|Xanthomonadaceae|Pseudoxanthomonas	0	0	0	0	0	0	0	0	0	0	0	0	0	0	0	0
Bacteria|Proteobacteria|Gammaproteobacteria|Xanthomonadales|Xanthomonadaceae|Pseudoxanthomonas|mexicana	0	0	0	0	0	0	0	0	0	0	0	0	0	0	0	0
Bacteria|SAR406	0	0	0	0	0	0	0	0	0	0	0	0	0	0	0	0
Bacteria|SAR406|AB16	0	0	0	0	0	0	0	0	0	0	0	0	0	0	0	0
Bacteria|SAR406|AB16|Arctic96B-7	0	0	0	0	0	0	0	0	0	0	0	0	0	0	0	0
Bacteria|SAR406|AB16|Arctic96B-7|A714017	0	0	0	0	0	0	0	0	0	0	0	0	0	0	0	0
Bacteria|SAR406|AB16|Arctic96B-7|A714017|so4B24	0	0	0	0	0	0	0	0	0	0	0	0	0	0	0	0
Bacteria|SAR406|AB16|noFP_H7	0	0	0	0	0	0	0	0	0	0	0	0	0	0	0	0
Bacteria|SBR1093	0	0	0	0	0	0	0	0	0	0	0	0	0	0	0	0
Bacteria|SBR1093|EC214	0	0	0	0	0	0	0	0	0	0	0	0	0	0	0	0
Bacteria|Spirochaetes	0	0.813365	0.325949	0	0	0	0	0	0	0	0	0	0	0	0	0
Bacteria|Spirochaetes|Spirochaetes	0	0.761723	0.304219	0	0	0	0	0	0	0	0	0	0	0	0	0
Bacteria|Spirochaetes|Spirochaetes|Spirochaetales	0	0.761723	0.304219	0	0	0	0	0	0	0	0	0	0	0	0	0
Bacteria|Spirochaetes|Spirochaetes|Spirochaetales|Spirochaetaceae	0	0.761723	0.304219	0	0	0	0	0	0	0	0	0	0	0	0	0
Bacteria|Spirochaetes|Spirochaetes|Spirochaetales|Spirochaetaceae|Treponema	0	0.722991	0.260759	0	0	0	0	0	0	0	0	0	0	0	0	0
Bacteria|Synergistetes	0	0.038732	0.08692	0	0	0	0	0	0	0	0	0	0	0	0	0
Bacteria|Synergistetes|Synergistia	0	0.038732	0.08692	0	0	0	0	0	0	0	0	0	0	0	0	0
Bacteria|Synergistetes|Synergistia|Synergistales	0	0.038732	0.08692	0	0	0	0	0	0	0	0	0	0	0	0	0
Bacteria|Synergistetes|Synergistia|Synergistales|Thermovirgaceae	0	0	0	0	0	0	0	0	0	0	0	0	0	0	0	0
Bacteria|Tenericutes	0	0	0	0	0	0	0	0	0	0	0	0	0	0	0	0
Bacteria|Tenericutes|Mollicutes	0	0	0	0	0	0	0	0	0	0	0	0	0	0	0	0
Bacteria|Tenericutes|Mollicutes|Acholeplasmatales	0	0	0	0	0	0	0	0	0	0	0	0	0	0	0	0
Bacteria|Tenericutes|Mollicutes|Acholeplasmatales|Acholeplasmataceae	0	0	0	0	0	0	0	0	0	0	0	0	0	0	0	0
Bacteria|Tenericutes|Mollicutes|Acholeplasmatales|Acholeplasmataceae|Acholeplasma	0	0	0	0	0	0	0	0	0	0	0	0	0	0	0	0
Bacteria|Tenericutes|RF3	0	0	0	0	0	0	0	0	0	0	0	0	0	0	0	0

Bacterial clades	China oil refineries									Mangrove			Marine sediments							Taiga upper active layer				Taiga bottom active layer			
	C1	C2	C3	C4	C5	C6	C7	C8	C9	M1	M2	M3	DWH1	DWH2	DWH3	DWH4	DWH5	DWH6	DWH7	Tu1	Tu2	Tu3	Tu4	Tb1	Tb2	Tb3	Tb4
Bacteria|Proteobacteria|Deltaproteobacteria|NB1-j|NB1-i	0	0	0	0	0	0	0	0	0	0	0.040636	0	2.284943	0	1.041122	0.475265	0.477361	0.086488	0.216575	0	0	0	0	0	0	0	0
Bacteria|Proteobacteria|Deltaproteobacteria|Sva0853	0	0	0	0	0	0	0	0	0	0.351045	0.528268	0.039476	0.456989	0.135591	0.520561	0.285159	0.143208	0	0.08663	0	0	0	0	0	0	0	0
Bacteria|Proteobacteria|Deltaproteobacteria|Sva0853|JTB36	0	0	0	0	0	0	0	0	0	0.351045	0.528268	0.039476	0.456989	0.135591	0.520561	0.285159	0.143208	0	0.08663	0	0	0	0	0	0	0	0
Bacteria|Proteobacteria|Deltaproteobacteria|Syntrophobacterales	0.094053	0.279856	1.393708	0.561089	0.215034	0.170569	0.227023	0.103769	0.252354	0.286037	0.135453	0.460559	0	0	0	0	0	0	0	0.031611	0	0	0	0	0.172722	0	0
Bacteria|Proteobacteria|Deltaproteobacteria|Syntrophobacterales|Syntrophaceae	0	0	0	0	0	0	0.066771	0	0	0.169022	0.135453	0.105271	0	0	0	0	0	0	0	0.031611	0	0	0	0	0	0	0
Bacteria|Proteobacteria|Deltaproteobacteria|Syntrophobacterales|Syntrophaceae|Syntrophus	0	0	0	0	0	0	0.040063	0	0	0	0	0	0	0	0	0	0	0	0	0	0	0	0	0	0	0	0
Bacteria|Proteobacteria|Deltaproteobacteria|Syntrophobacterales|Syntrophobacteraceae	0.094053	0.279856	1.393708	0.561089	0.215034	0.170569	0.160251	0.103769	0.252354	0.117015	0	0.355288	0	0	0	0	0	0	0	0	0	0	0	0	0.086361	0	0
Bacteria|Proteobacteria|Deltaproteobacteria|Syntrophobacterales|Syntrophobacteraceae|Desulfacinum	0	0	0	0	0	0	0	0	0	0	0	0	0	0	0	0	0	0	0	0	0	0	0	0	0	0	0
Bacteria|Proteobacteria|Deltaproteobacteria|Syntrophobacterales|Syntrophobacteraceae|Syntrophobacter	0	0.079959	1.351474	0	0	0	0	0	0	0	0	0	0	0	0	0	0	0	0	0	0	0	0	0	0	0	0
Bacteria|Proteobacteria|Deltaproteobacteria|Syntrophobacterales|Syntrophorhabdaceae	0	0	0	0	0	0	0	0	0	0	0	0	0	0	0	0	0	0	0	0	0	0	0	0	0.086361	0	0
Bacteria|Proteobacteria|Epsilonproteobacteria	0	0	0	0.024937	0	0.056856	0	0	0	0.279536	0.209953	0.743473	0.396057	1.416175	1.041122	0.982215	0.946766	0.086488	0.375397	0	0	0	0	0	0	0	0
Bacteria|Proteobacteria|Epsilonproteobacteria|Campylobacterales	0	0	0	0.024937	0	0.056856	0	0	0	0.279536	0.209953	0.743473	0.396057	1.416175	1.041122	0.982215	0.946766	0.086488	0.375397	0	0	0	0	0	0	0	0
Bacteria|Proteobacteria|Epsilonproteobacteria|Campylobacterales|Campylobacteraceae	0	0	0	0	0	0	0	0	0	0	0	0.131588	0.365591	1.175124	0.75718	0.697056	0.70013	0.086488	0.231013	0	0	0	0	0	0	0	0
Bacteria|Proteobacteria|Epsilonproteobacteria|Campylobacterales|Campylobacteraceae|Arcobacter	0	0	0	0	0	0	0	0	0	0	0	0.131588	0.335125	0.994335	0.725631	0.380212	0.636482	0.086488	0.231013	0	0	0	0	0	0	0	0
Bacteria|Proteobacteria|Epsilonproteobacteria|Campylobacterales|Helicobacteraceae	0	0	0	0.024937	0	0.056856	0	0	0	0.279536	0.209953	0.611885	0.030466	0.241051	0.283942	0.285159	0.246637	0	0.144383	0	0	0	0	0	0	0	0
Bacteria|Proteobacteria|Gammaproteobacteria	7.427976	6.943085	7.316261	3.650284	2.803402	32.45163	2.357795	2.870945	14.59585	15.06048	20.0019	16.75842	28.91672	35.53091	31.75445	46.43285	49.30425	23.18833	45.96743	2.026283	0.049745	0.265728	0.291982	0.068514	0.129542	0.206347	0.821035
Bacteria|Proteobacteria|Gammaproteobacteria|Alteromonadales	0.391889	0.426447	0.17175	0.478796	0.222998	1.138072	0.416654	0.219068	1.045865	5.533509	5.651109	6.376765	2.81505	12.64614	11.73088	20.76909	29.0522	13.25865	19.12295	0	0	0	0	0	0	0.018931	0
Bacteria|Proteobacteria|Gammaproteobacteria|Alteromonadales|Alteromonadaceae	0.391889	0.346488	0.157672	0.466327	0.119463	1.026255	0.336528	0.207538	0.9281	2.397507	1.977617	4.692436	1.553761	10.56707	10.05156	18.10761	25.69158	12.80891	17.46461	0	0	0	0	0	0	0	0
Bacteria|Proteobacteria|Gammaproteobacteria|Alteromonadales|Alteromonadaceae|Cellvibrio	0.062702	0.159918	0	0.174561	0.071678	0.085284	0.026709	0.115299	0.532746	0	0	0	0	0	0	0	0	0	0	0	0	0	0	0	0	0	0
Bacteria|Proteobacteria|Gammaproteobacteria|Alteromonadales|Alteromonadaceae|HB2-32-21	0.047027	0	0	0.037406	0	0.383779	0.080126	0	0.042059	0.195025	0.284452	0.078953	1.142472	9.943353	9.559394	16.63428	25.1092	12.02187	16.28644	0	0	0	0	0	0	0	0
Bacteria|Proteobacteria|Gammaproteobacteria|Alteromonadales|Alteromonadaceae|Marinobacter	0.28216	0.159918	0.140779	0.199498	0.047785	0.540134	0.213669	0.092239	0.336471	2.10627	1.462895	4.395047	0	0	0	0	0	0	0	0	0	0	0	0	0	0	0
Bacteria|Proteobacteria|Gammaproteobacteria|Alteromonadales|Alteromonadaceae|ZD0117	0	0	0	0	0	0	0	0	0	0.039005	0.040636	0.039476	0.045699	0.361576	0.283942	0.522792	0.238681	0.475685	0.389835	0	0	0	0	0	0	0	0
Bacteria|Proteobacteria|Gammaproteobacteria|Alteromonadales|Colwelliaceae	0	0	0	0	0	0	0	0	0	0	0.016254	0	0.767741	1.663252	1.646866	2.357316	3.303339	0.276762	1.438058	0	0	0	0	0	0	0	0
Bacteria|Proteobacteria|Gammaproteobacteria|Alteromonadales|Colwelliaceae|Colwellia	0	0	0	0	0	0	0	0	0	0	0	0	0.237634	0.542365	0.37859	1.197668	2.482278	0.069191	0.571758	0	0	0	0	0	0	0	0
Bacteria|Proteobacteria|Gammaproteobacteria|Alteromonadales|OM60	0	0.079959	0	0	0	0	0	0	0	2.65234	3.575966	1.3422	0	0	0	0	0	0	0	0	0	0	0	0	0	0	0
Bacteria|Proteobacteria|Gammaproteobacteria|Alteromonadales|OM60|Congregibacter	0	0	0	0	0	0	0	0	0	1.32617	2.031799	0.631623	0	0	0	0	0	0	0	0	0	0	0	0	0	0	0
Bacteria|Proteobacteria|Gammaproteobacteria|Chromatiales	0	0	0	0.037406	0	0	0	0	0.042059	0.208027	0.189635	0.171065	0	0	0	0	0	0	0	0	0	0	0	0	0	0	0
Bacteria|Proteobacteria|Gammaproteobacteria|Chromatiales|Ectothiorhodospiraceae	0	0	0	0.037406	0	0	0	0	0.042059	0	0	0.039476	0	0	0	0	0	0	0	0	0	0	0	0	0	0	0
Bacteria|Proteobacteria|Gammaproteobacteria|Enterobacteriales	0.076138	0	0.028156	0.035625	0.047785	0.028428	0.011447	0.069179	0.056079	0	0.027091	0	0	0	0	0	0	0	0	0	0	0	0	0	0	0	0.030982
Bacteria|Proteobacteria|Gammaproteobacteria|Enterobacteriales|Enterobacteriaceae	0.076138	0	0.028156	0.035625	0.047785	0.028428	0.011447	0.069179	0.056079	0	0.027091	0	0	0	0	0	0	0	0	0	0	0	0	0	0	0	0.030982
Bacteria|Proteobacteria|Gammaproteobacteria|Enterobacteriales|Enterobacteriaceae|Serratia	0	0	0	0	0	0	0	0	0	0	0	0	0	0	0	0	0	0	0	0	0	0	0	0	0	0	0
Bacteria|Proteobacteria|Gammaproteobacteria|Enterobacteriales|Enterobacteriaceae|Serratia|symbiotica	0	0	0	0	0	0	0	0	0	0	0	0	0	0	0	0	0	0	0	0	0	0	0	0	0	0	0
Bacteria|Proteobacteria|Gammaproteobacteria|Legionellales	0	0.079959	0.042234	0.074812	0.215034	0.170569	0.080126	0.03459	0.042059	0.31204	0.40636	0.078953	0.319892	0	0.047324	0	0	0.043244	0.043315	0.142251	0.049745	0	0	0	0.043181	0	0
Bacteria|Proteobacteria|Gammaproteobacteria|Legionellales|Legionellaceae	0	0	0	0	0.107517	0.170569	0.080126	0.03459	0	0.195025	0.243816	0	0.045699	0	0	0	0	0.043244	0	0.094834	0.049745	0	0	0	0	0	0
Bacteria|Proteobacteria|Gammaproteobacteria|Legionellales|Legionellaceae|Legionella	0	0	0	0	0.071678	0.127926	0	0.03459	0	0	0.040636	0	0	0	0	0	0	0	0	0.094834	0.049745	0	0	0	0	0	0
Bacteria|Proteobacteria|Gammaproteobacteria|Marinicellales	0	0.026653	0.028156	0.024937	0.047785	0.028428	0	0	0	1.118143	1.27326	0.684259	1.584227	2.229722	0.820278	1.108952	0.731954	0.663077	1.241697	0	0	0	0	0	0	0	0
Bacteria|Proteobacteria|Gammaproteobacteria|Marinicellales|Marinicellaceae	0	0.026653	0.028156	0.024937	0.047785	0.028428	0	0	0	1.118143	1.27326	0.684259	1.584227	2.229722	0.820278	1.108952	0.731954	0.663077	1.241697	0	0	0	0	0	0	0	0
Bacteria|Proteobacteria|Gammaproteobacteria|Marinicellales|Marinicellaceae|Marinicella	0	0	0	0	0	0	0	0	0	0.182023	0	0	0	1.898276	0.441688	0.697056	0.541009	0.374782	0.77967	0	0	0	0	0	0	0	0
Bacteria|Proteobacteria|Gammaproteobacteria|Methylococcales	0	0	0	0	0	0	0	0	0.042059	0	0	0.078953	0	0	0	0	0	0	0	0	0	0	0	0	0	0	0
Bacteria|Proteobacteria|Gammaproteobacteria|Methylococcales|Crenotrichaceae	0	0	0	0	0	0	0	0	0	0	0	0	0	0	0	0	0	0	0	0	0	0	0	0	0	0	0
Bacteria|Proteobacteria|Gammaproteobacteria|Methylococcales|Crenotrichaceae|Crenothrix	0	0	0	0	0	0	0	0	0	0	0	0	0	0	0	0	0	0	0	0	0	0	0	0	0	0	0
Bacteria|Proteobacteria|Gammaproteobacteria|Oceanospirillales	0.062702	0.18657	0.168934	0.367825	0.143356	0.739131	0.160251	0.196008	0.37853	0.62408	0.419905	0.723735	0.068548	0.564963	0.212957	1.354506	0.047736	0.086488	0.801328	0	0	0	0	0	0	0	0
Bacteria|Proteobacteria|Gammaproteobacteria|Oceanospirillales|Halomonadaceae	0	0.159918	0.084467	0.299247	0.143356	0.540134	0.053417	0.161419	0.077108	0	0.054181	0	0	0	0	0	0	0	0	0	0	0	0	0	0	0	0
Bacteria|Proteobacteria|Gammaproteobacteria|Oceanospirillales|Halomonadaceae|Halomonas	0	0.133265	0.084467	0.299247	0.143356	0.511706	0.053417	0.161419	0.056079	0	0.054181	0	0	0	0	0	0	0	0	0	0	0	0	0	0	0	0
Bacteria|Proteobacteria|Gammaproteobacteria|Pseudomonadales	2.570792	3.411573	0.499764	0.448871	0.298658	24.88881	0.367243	0.634144	10.28341	0.026003	0	0.107902	0.045699	0	0	0.031684	0	0	0.332082	1.125361	0	0.118101	0	0.017129	0	0.187416	0
Bacteria|Proteobacteria|Gammaproteobacteria|Pseudomonadales|Moraxellaceae	2.492414	2.678618	0.253401	0.224436	0.143356	23.23998	0.260409	0.380487	8.432814	0	0	0	0.045699	0	0	0	0	0	0	0	0	0	0	0	0	0	0
Bacteria|Proteobacteria|Gammaproteobacteria|Pseudomonadales|Moraxellaceae|Acinetobacter	0.235133	0.199897	0	0	0.035839	0.170569	0.080126	0.345897	1.051473	0	0	0	0.045699	0	0	0	0	0	0	0	0	0	0	0	0	0	0
Bacteria|Proteobacteria|Gammaproteobacteria|Pseudomonadales|Moraxellaceae|Acinetobacter|rhizosphaerae	0	0	0	0	0	0	0	0	0	0	0	0	0	0	0	0	0	0	0	0	0	0	0	0	0	0	0
Bacteria|Proteobacteria|Gammaproteobacteria|Pseudomonadales|Moraxellaceae|Alkanindiges	2.210254	2.478721	0.253401	0.18703	0.107517	22.81356	0.160251	0.03459	6.729428	0	0	0	0	0	0	0	0	0	0	0	0	0	0	0	0	0	0
Bacteria|Proteobacteria|Gammaproteobacteria|Pseudomonadales|Moraxellaceae|Perlucidibaca	0.047027	0	0	0	0	0.170569	0	0	0.630884	0	0	0	0	0	0	0	0	0	0	0	0	0	0	0	0	0	0
Bacteria|Proteobacteria|Gammaproteobacteria|Pseudomonadales|Pseudomonadaceae	0.078378	0.732955	0.246362	0.224436	0.155302	1.64883	0.106834	0.253658	1.850593	0.026003	0	0.107902	0	0	0	0.031684	0	0	0.332082	1.125361	0	0.118101	0	0.017129	0	0.187416	0
Bacteria|Proteobacteria|Gammaproteobacteria|Pseudomonadales|Pseudomonadaceae|Pseudomonas	0.078378	0.732955	0.246362	0.224436	0.155302	1.64883	0.106834	0.253658	1.836573	0.026003	0	0.107902	0	0	0	0.031684	0	0	0.332082	1.125361	0	0.118101	0	0.017129	0	0.187416	0
Bacteria|Proteobacteria|Gammaproteobacteria|Pseudomonadales|Pseudomonadaceae|Pseudomonas|stutzeri	0.031351	0.239876	0.168934	0.074812	0.047785	0.312709	0.026709	0.138359	0.925296	0	0	0	0	0	0	0	0	0	0	0	0	0	0	0	0	0	0
Bacteria|Proteobacteria|Gammaproteobacteria|Pseudomonadales|Pseudomonadaceae|Pseudomonas|viridiflava	0	0	0	0	0	0	0.013354	0	0	0	0	0	0	0	0	0	0	0	0	1.043172	0	0.014763	0	0.017129	0	0	0
Bacteria|Proteobacteria|Gammaproteobacteria|PYR10d3	0.14108	0.599691	0.633503	0	0	1.492476	0.080126	0.03459	0.79912	0.07801	0.040636	0	0	0	0	0	0	0	0	0	0	0	0	0	0	0	0
Bacteria|Proteobacteria|Gammaproteobacteria|Thiotrichales	0	0.026653	0	0.199498	0	0.042642	0.106834	0.02306	0.042059	1.898243	3.318604	2.197523	0	0.060263	0	0.316844	0	0	0	0	0	0	0	0	0	0	0
Bacteria|Proteobacteria|Gammaproteobacteria|Thiotrichales|Piscirickettsiaceae	0	0.026653	0	0.199498	0	0.042642	0.106834	0.02306	0.042059	1.040133	2.180797	1.368518	0	0.060263	0	0.316844	0	0	0	0	0	0	0	0	0	0	0
Bacteria|Proteobacteria|Gammaproteobacteria|Thiotrichales|Thiotrichaceae	0	0	0	0	0	0	0	0	0	0.85811	1.137807	0.829006	0	0	0	0	0	0	0	0	0	0	0	0	0	0	0
Bacteria|Proteobacteria|Gammaproteobacteria|Xanthomonadales	4.138348	2.132233	5.743765	1.982514	1.827786	3.923079	1.135115	1.625716	1.682357	5.109655	8.005287	6.000423	23.80911	19.9771	18.7402	22.71768	19.4286	9.124512	24.29972	0.758671	0	0.147627	0.291982	0.051386	0.086361	0	0.790053
Bacteria|Proteobacteria|Gammaproteobacteria|Xanthomonadales|Sinobacteraceae	3.150788	0.879546	4.350057	0.448871	0.537584	0.724917	0.440692	0.311307	0.37853	0	0	0	0	0	0	0	0	0	0	0.426752	0	0	0.125135	0	0	0	0.743579
Bacteria|Proteobacteria|Gammaproteobacteria|Xanthomonadales|Sinobacteraceae|Hydrocarboniphaga	0.517294	0.039979	0.295635	0	0	0	0	0.03459	0	0	0	0	0	0	0	0	0	0	0	0	0	0	0.083424	0	0	0	0.557684
Bacteria|Proteobacteria|Gammaproteobacteria|Xanthomonadales|Sinobacteraceae|Hydrocarboniphaga|effusa	0.517294	0.039979	0.295635	0	0	0	0	0.03459	0	0	0	0	0	0	0	0	0	0	0	0	0	0	0.083424	0	0	0	0.557684
Bacteria|Proteobacteria|Gammaproteobacteria|Xanthomonadales|Sinobacteraceae|Nevskia	0.047027	0.079959	0.084467	0.037406	0	0	0	0	0	0	0	0	0	0	0	0	0	0	0	0	0	0	0.041712	0	0	0	0.185895
Bacteria|Proteobacteria|Gammaproteobacteria|Xanthomonadales|Xanthomonadaceae	0.98756	1.252687	1.393708	1.533643	1.290202	3.198162	0.694423	1.314409	1.303827	5.109655	8.005287	6.000423	23.80911	19.9771	18.7402	22.71768	19.4286	9.124512	24.29972	0.331918	0	0.147627	0.166847	0.051386	0.086361	0	0.046474
Bacteria|Proteobacteria|Gammaproteobacteria|Xanthomonadales|Xanthomonadaceae|Arenimonas	0	0.079959	0	0	0.071678	0.127926	0	0.03459	0.126177	0	0	0	0	0	0	0	0	0	0	0	0	0	0	0	0	0	0
Bacteria|Proteobacteria|Gammaproteobacteria|Xanthomonadales|Xanthomonadaceae|Dokdonella	0	0.119938	0.084467	0	0.107517	0.59699	0	0.069179	0	0	0	0	0	0	0	0	0	0	0	0.189668	0	0	0	0	0	0	0
Bacteria|Proteobacteria|Gammaproteobacteria|Xanthomonadales|Xanthomonadaceae|Dyella	0	0	0	0	0	0	0	0	0	0	0	0	0	0	0	0	0	0	0	0.047417	0	0	0	0.051386	0	0	0.046474
Bacteria|Proteobacteria|Gammaproteobacteria|Xanthomonadales|Xanthomonadaceae|Dyella|ginsengisoli	0	0	0	0	0	0	0	0	0	0	0	0	0	0	0	0	0	0	0	0.047417	0	0	0	0.051386	0	0	0.046474
Bacteria|Proteobacteria|Gammaproteobacteria|Xanthomonadales|Xanthomonadaceae|Luteibacter	0	0	0	0	0	0	0	0	0	0	0	0	0	0	0	0	0	0	0	0.094834	0	0	0.166847	0	0	0	0
Bacteria|Proteobacteria|Gammaproteobacteria|Xanthomonadales|Xanthomonadaceae|Luteibacter|rhizovicinus	0	0	0	0	0	0	0	0	0	0	0	0	0	0	0	0	0	0	0	0.094834	0	0	0.166847	0	0	0	0
Bacteria|Proteobacteria|Gammaproteobacteria|Xanthomonadales|Xanthomonadaceae|Luteimonas	0.28216	0.119938	0.042234	0.299247	0	0	0	0.172948	0.084118	0	0	0	0	0	0	0	0	0	0	0	0	0	0	0	0.043181	0	0
Bacteria|Proteobacteria|Gammaproteobacteria|Xanthomonadales|Xanthomonadaceae|Pseudoxanthomonas	0.14108	0.239876	0.633503	0	0	0.383779	0	0.03459	0.042059	0	0	0	0	0	0	0	0	0	0	0	0	0	0	0	0	0	0
Bacteria|Proteobacteria|Gammaproteobacteria|Xanthomonadales|Xanthomonadaceae|Pseudoxanthomonas|mexicana	0.14108	0.239876	0.633503	0	0	0.383779	0	0.03459	0.042059	0	0	0	0	0	0	0	0	0	0	0	0	0	0	0	0	0	0
Bacteria|SAR406	0	0	0	0	0	0	0	0	0	0.62408	0.243816	0.789529	0	0	0.094647	0	0	0	0	0	0	0	0	0	0	0	0
Bacteria|SAR406|AB16	0	0	0	0	0	0	0	0	0	0.62408	0.243816	0.789529	0	0	0.094647	0	0	0	0	0	0	0	0	0	0	0	0
Bacteria|SAR406|AB16|Arctic96B-7	0	0	0	0	0	0	0	0	0	0.62408	0.162544	0.789529	0	0	0.094647	0	0	0	0	0	0	0	0	0	0	0	0
Bacteria|SAR406|AB16|Arctic96B-7|A714017	0	0	0	0	0	0	0	0	0	0.62408	0.162544	0.789529	0	0	0.094647	0	0	0	0	0	0	0	0	0	0	0	0
Bacteria|SAR406|AB16|Arctic96B-7|A714017|so4B24	0	0	0	0	0	0	0	0	0	0.62408	0.162544	0.789529	0	0	0.094647	0	0	0	0	0	0	0	0	0	0	0	0
Bacteria|SAR406|AB16|noFP_H7	0	0	0	0	0	0	0	0	0	0	0.081272	0	0	0	0	0	0	0	0	0	0	0	0	0	0	0	0
Bacteria|SBR1093	0	0	0.028156	0.049875	0	0	0	0	0	0.104013	0.270906	0	0.578852	0.030131	0.031549	0	0	0	0.028877	0	0	0	0	0	0	0	0
Bacteria|SBR1093|EC214	0	0	0	0	0	0	0	0	0	0	0	0	0.548386	0.030131	0.031549	0	0	0	0	0	0	0	0	0	0	0	0
Bacteria|Spirochaetes	0	0.026653	0	0	0	0	0.020031	0	0.028039	2.613335	1.401941	1.210612	0.274193	0.888875	0.804503	0.332686	0.620569	0.086488	0.51978	0	0	0	0	0	0	0	0
Bacteria|Spirochaetes|Spirochaetes	0	0.026653	0	0	0	0	0	0	0.028039	2.223285	1.178443	1.013229	0.274193	0.888875	0.804503	0.332686	0.620569	0.086488	0.476465	0	0	0	0	0	0	0	0
Bacteria|Spirochaetes|Spirochaetes|Spirochaetales	0	0.026653	0	0	0	0	0	0	0.028039	2.223285	1.178443	1.013229	0.274193	0.888875	0.804503	0.332686	0.620569	0.086488	0.476465	0	0	0	0	0	0	0	0
Bacteria|Spirochaetes|Spirochaetes|Spirochaetales|Spirochaetaceae	0	0.026653	0	0	0	0	0	0	0.028039	2.223285	1.178443	1.013229	0.274193	0.888875	0.804503	0.332686	0.620569	0.086488	0.476465	0	0	0	0	0	0	0	0
Bacteria|Spirochaetes|Spirochaetes|Spirochaetales|Spirochaetaceae|Treponema	0	0.026653	0	0	0	0	0	0	0.028039	0	0	0.026318	0	0.030131	0	0	0	0	0	0	0	0	0	0	0	0	0
Bacteria|Synergistetes	0.094053	0.039979	0.042234	0.074812	0	0	0.080126	0	0	0.351045	0.243816	0.315812	0	0	0	0	0	0	0	0	0	0	0	0	0	0	0
Bacteria|Synergistetes|Synergistia	0.094053	0.039979	0.042234	0.074812	0	0	0.080126	0	0	0.351045	0.243816	0.315812	0	0	0	0	0	0	0	0	0	0	0	0	0	0	0
Bacteria|Synergistetes|Synergistia|Synergistales	0.094053	0.039979	0.042234	0.074812	0	0	0.080126	0	0	0.351045	0.243816	0.315812	0	0	0	0	0	0	0	0	0	0	0	0	0	0	0
Bacteria|Synergistetes|Synergistia|Synergistales|Thermovirgaceae	0	0	0	0	0	0	0	0	0	0	0	0	0	0	0	0	0	0	0	0	0	0	0	0	0	0	0
Bacteria|Tenericutes	0	0	0.042234	0	0	0.042642	0.080126	0.03459	0.042059	0	0	0	0	0	0	0	0	0	0	0	0	0	0	0	0	0	0
Bacteria|Tenericutes|Mollicutes	0	0	0.042234	0	0	0.042642	0.080126	0.03459	0.042059	0	0	0	0	0	0	0	0	0	0	0	0	0	0	0	0	0	0
Bacteria|Tenericutes|Mollicutes|Acholeplasmatales	0	0	0	0	0	0	0	0	0	0	0	0	0	0	0	0	0	0	0	0	0	0	0	0	0	0	0
Bacteria|Tenericutes|Mollicutes|Acholeplasmatales|Acholeplasmataceae	0	0	0	0	0	0	0	0	0	0	0	0	0	0	0	0	0	0	0	0	0	0	0	0	0	0	0
Bacteria|Tenericutes|Mollicutes|Acholeplasmatales|Acholeplasmataceae|Acholeplasma	0	0	0	0	0	0	0	0	0	0	0	0	0	0	0	0	0	0	0	0	0	0	0	0	0	0	0
Bacteria|Tenericutes|RF3	0	0	0	0	0	0	0	0	0	0	0	0	0	0	0	0	0	0	0	0	0	0	0	0	0	0	0

Bacterial clades	Taiga permafrost layer				Oil sands core							Oil sands tailings pond upper				Oil sands tailings pond median			Oil sands tailings pond deep				Differentially abundant class (one class test, non-strict)	p-value
	Tp1	Tp2	Tp3	Tp4	OSC1	OSC12	OSC3	OSC4	OSC5	OSC7	OSC9	OSTPu1	OSTPu2	OSTPu4	OSTPu3	OSTPm2	OSTPm6	OSTPm4	OSTPd1	OSTPd2	OSTPd3	OSTPd4		
Bacteria|Proteobacteria|Deltaproteobacteria|NB1-j|NB1-i	0	0	0	0	0	0	0	0	0	0	0	0	0	0	0	0	0	0	0	0	0	0		
Bacteria|Proteobacteria|Deltaproteobacteria|Sva0853	0	0	0	0	0	0	0	0	0	0	0	0	0	0	0	0	0	0	0	0	0	0		
Bacteria|Proteobacteria|Deltaproteobacteria|Sva0853|JTB36	0	0	0	0	0	0	0	0	0	0	0	0	0	0	0	0	0	0	0	0	0	0		
Bacteria|Proteobacteria|Deltaproteobacteria|Syntrophobacterales	0	0	0	0	0	0	0	0	0	0	0	8.165297	5.354402	5.619658	9.15216	6.7561	1.166652	13.5305	0.073425	0.287947	2.139114	13.10101	Oil sands tailings pond median	1.81E-08
Bacteria|Proteobacteria|Deltaproteobacteria|Syntrophobacterales|Syntrophaceae	0	0	0	0	0	0	0	0	0	0	0	7.717202	4.914314	3.33201	5.176979	5.499151	1.060593	11.67337	0.073425	0.230357	1.657813	3.816119	Oil sands tailings pond median	9.48E-08
Bacteria|Proteobacteria|Deltaproteobacteria|Syntrophobacterales|Syntrophaceae|Syntrophus	0	0	0	0	0	0	0	0	0	0	0	7.46826	4.455889	2.884426	4.992087	4.870676	0.848474	11.14277	0	0.230357	1.657813	3.245204	Oil sands tailings pond median	4.3E-07
Bacteria|Proteobacteria|Deltaproteobacteria|Syntrophobacterales|Syntrophobacteraceae	0	0	0	0	0	0	0	0	0	0	0	0.049788	0.110022	0.298389	1.848921	0.523729	0	0.265304	0	0.057589	0.160434	7.662287		
Bacteria|Proteobacteria|Deltaproteobacteria|Syntrophobacterales|Syntrophobacteraceae|Desulfacinum	0	0	0	0	0	0	0	0	0	0	0	0	0	0	0	0	0	0.088435	0	0	0	7.211564		
Bacteria|Proteobacteria|Deltaproteobacteria|Syntrophobacterales|Syntrophobacteraceae|Syntrophobacter	0	0	0	0	0	0	0	0	0	0	0	0	0	0.298389	1.848921	0	0	0	0	0	0.106956	0		
Bacteria|Proteobacteria|Deltaproteobacteria|Syntrophobacterales|Syntrophorhabdaceae	0	0	0	0	0	0	0	0	0	0	0	0.398307	0.330066	1.98926	2.126259	0.73322	0.106059	1.591824	0	0	0.320867	1.622602	Oil sands tailings pond upper	1.03E-06
Bacteria|Proteobacteria|Epsilonproteobacteria	0	0	0	0	0	0	0	0	0	0	0	0.199154	0	0	0	0.034915	0	0	0	0	0	0.060096	Marine sediments	3.15E-07
Bacteria|Proteobacteria|Epsilonproteobacteria|Campylobacterales	0	0	0	0	0	0	0	0	0	0	0	0.199154	0	0	0	0.034915	0	0	0	0	0	0.060096	Marine sediments	3.15E-07
Bacteria|Proteobacteria|Epsilonproteobacteria|Campylobacterales|Campylobacteraceae	0	0	0	0	0	0	0	0	0	0	0	0.066385	0	0	0	0	0	0	0	0	0	0.030048		
Bacteria|Proteobacteria|Epsilonproteobacteria|Campylobacterales|Campylobacteraceae|Arcobacter	0	0	0	0	0	0	0	0	0	0	0	0.033192	0	0	0	0	0	0	0	0	0	0.030048		
Bacteria|Proteobacteria|Epsilonproteobacteria|Campylobacterales|Helicobacteraceae	0	0	0	0	0	0	0	0	0	0	0	0.132769	0	0	0	0.034915	0	0	0	0	0	0.030048		
Bacteria|Proteobacteria|Gammaproteobacteria	1.451876	0.186352	0.097364	0.823874	26.78721	30.30554	13.49385	37.55494	39.49596	37.05945	33.43736	3.352419	5.629457	2.161662	0.955276	6.197456	1.417659	7.026343	8.407218	4.050448	18.85985	12.8486	Marine sediments	1.51E-07
Bacteria|Proteobacteria|Gammaproteobacteria|Alteromonadales	0	0	0	0	0	0	0.336945	0	0	0.134543	0	0.049788	0.055011	0.18898	0	0	0.021212	0	1.541935	0.345536	0	0.018029	Marine sediments	2.03E-07
Bacteria|Proteobacteria|Gammaproteobacteria|Alteromonadales|Alteromonadaceae	0	0	0	0	0	0	0.22463	0	0	0.134543	0	0.049788	0.055011	0.049731	0	0	0	0	1.541935	0.345536	0	0	Marine sediments	7.28E-08
Bacteria|Proteobacteria|Gammaproteobacteria|Alteromonadales|Alteromonadaceae|Cellvibrio	0	0	0	0	0	0	0.037438	0	0	0.134543	0	0	0	0	0	0	0	0	0	0	0	0		
Bacteria|Proteobacteria|Gammaproteobacteria|Alteromonadales|Alteromonadaceae|HB2-32-21	0	0	0	0	0	0	0.112315	0	0	0	0	0.049788	0.055011	0.049731	0	0	0	0	1.541935	0.345536	0	0	Marine sediments	2.6E-06
Bacteria|Proteobacteria|Gammaproteobacteria|Alteromonadales|Alteromonadaceae|Marinobacter	0	0	0	0	0	0	0	0	0	0	0	0	0	0	0	0	0	0	0	0	0	0		
Bacteria|Proteobacteria|Gammaproteobacteria|Alteromonadales|Alteromonadaceae|ZD0117	0	0	0	0	0	0	0	0	0	0	0	0	0	0	0	0	0	0	0	0	0	0		
Bacteria|Proteobacteria|Gammaproteobacteria|Alteromonadales|Colwelliaceae	0	0	0	0	0	0	0	0	0	0	0	0	0	0	0	0	0	0	0	0	0	0	Marine sediments	1.46E-08
Bacteria|Proteobacteria|Gammaproteobacteria|Alteromonadales|Colwelliaceae|Colwellia	0	0	0	0	0	0	0	0	0	0	0	0	0	0	0	0	0	0	0	0	0	0		
Bacteria|Proteobacteria|Gammaproteobacteria|Alteromonadales|OM60	0	0	0	0	0	0	0	0	0	0	0	0	0	0	0	0	0	0	0	0	0	0		
Bacteria|Proteobacteria|Gammaproteobacteria|Alteromonadales|OM60|Congregibacter	0	0	0	0	0	0	0	0	0	0	0	0	0	0	0	0	0	0	0	0	0	0		
Bacteria|Proteobacteria|Gammaproteobacteria|Chromatiales	0	0	0	0	0	0	0	0	0	0.151361	0	0	0.055011	0.198926	0.092446	0	0.088383	0.029478	0	0	0	0		
Bacteria|Proteobacteria|Gammaproteobacteria|Chromatiales|Ectothiorhodospiraceae	0	0	0	0	0	0	0	0	0	0.151361	0	0	0	0	0	0	0.05303	0	0	0	0	0		
Bacteria|Proteobacteria|Gammaproteobacteria|Enterobacteriales	0	0.02867	0	0	2.278609	2.569935	0.914566	2.289728	4.063908	3.77682	3.172565	0.033192	0	0	0	0.087288	0	0.614831	0	0	1.283468	0.901446	Oil sands core	1.54E-05
Bacteria|Proteobacteria|Gammaproteobacteria|Enterobacteriales|Enterobacteriaceae	0	0.02867	0	0	2.278609	2.569935	0.914566	2.289728	4.063908	3.77682	3.172565	0.033192	0	0	0	0.087288	0	0.614831	0	0	1.283468	0.901446	Oil sands core	1.54E-05
Bacteria|Proteobacteria|Gammaproteobacteria|Enterobacteriales|Enterobacteriaceae|Serratia	0	0	0	0	0	0	0	0	0	0	0.516464	0	0	0	0	0	0	0	0	0	0	0		
Bacteria|Proteobacteria|Gammaproteobacteria|Enterobacteriales|Enterobacteriaceae|Serratia|symbiotica	0	0	0	0	0	0	0	0	0	0	0.516464	0	0	0	0	0	0	0	0	0	0	0		
Bacteria|Proteobacteria|Gammaproteobacteria|Legionellales	0.043731	0	0	0	0.158097	0	0	0.101874	0	0	0.051646	0.049788	0	0	0	0	0	0	0.715898	0	0.481301	0		
Bacteria|Proteobacteria|Gammaproteobacteria|Legionellales|Legionellaceae	0	0	0	0	0.158097	0	0	0.101874	0	0	0.051646	0	0	0	0	0	0	0	0.715898	0	0.481301	0		
Bacteria|Proteobacteria|Gammaproteobacteria|Legionellales|Legionellaceae|Legionella	0	0	0	0	0	0	0	0	0	0	0	0	0	0	0	0	0	0	0.715898	0	0.481301	0		
Bacteria|Proteobacteria|Gammaproteobacteria|Marinicellales	0	0	0	0	0	0	0	0	0	0	0	0	0	0	0	0	0	0	0	0	0	0	Marine sediments	8.09E-08
Bacteria|Proteobacteria|Gammaproteobacteria|Marinicellales|Marinicellaceae	0	0	0	0	0	0	0	0	0	0	0	0	0	0	0	0	0	0	0	0	0	0	Marine sediments	8.09E-08
Bacteria|Proteobacteria|Gammaproteobacteria|Marinicellales|Marinicellaceae|Marinicella	0	0	0	0	0	0	0	0	0	0	0	0	0	0	0	0	0	0	0	0	0	0		
Bacteria|Proteobacteria|Gammaproteobacteria|Methylococcales	0	0	0	0	0	0.155082	0.168473	0	0	0.050454	0.981282	0	0	0	0.046223	0	0.477267	0.044217	0	0	0	0		
Bacteria|Proteobacteria|Gammaproteobacteria|Methylococcales|Crenotrichaceae	0	0	0	0	0	0.155082	0.168473	0	0	0.050454	0.929635	0	0	0	0	0	0.477267	0.044217	0	0	0	0		
Bacteria|Proteobacteria|Gammaproteobacteria|Methylococcales|Crenotrichaceae|Crenothrix	0	0	0	0	0	0.155082	0.168473	0	0	0.050454	0.929635	0	0	0	0	0	0.477267	0.044217	0	0	0	0		
Bacteria|Proteobacteria|Gammaproteobacteria|Oceanospirillales	0	0	0	0	0	0	0.140394	0.15281	0	0	0	0	0	0	0.030815	0	0	0.176869	0	0	0	0.120193	Mangrove	1.09E-06
Bacteria|Proteobacteria|Gammaproteobacteria|Oceanospirillales|Halomonadaceae	0	0	0	0	0	0	0	0	0	0	0	0	0	0	0.030815	0	0	0.176869	0	0	0	0.120193		
Bacteria|Proteobacteria|Gammaproteobacteria|Oceanospirillales|Halomonadaceae|Halomonas	0	0	0	0	0	0	0	0	0	0	0	0	0	0	0.030815	0	0	0.176869	0	0	0	0.120193		
Bacteria|Proteobacteria|Gammaproteobacteria|Pseudomonadales	0.941679	0.100344	0.056796	0	23.38084	26.19167	11.55909	34.21252	35.04213	30.97857	27.90628	0.431499	0.788491	0.828858	0.739568	4.119999	0.300501	6.11673	0.642473	0.364732	16.45335	11.67372	Oil sands core	1.08E-06
Bacteria|Proteobacteria|Gammaproteobacteria|Pseudomonadales|Moraxellaceae	0.699699	0.086009	0.040568	0	23.09978	25.9849	10.81033	33.83898	34.51658	30.67585	27.61362	0	0.055011	0.149194	0.647122	0.471356	0.05303	5.527165	0	0	15.70466	10.86242	Oil sands core	2.11E-07
Bacteria|Proteobacteria|Gammaproteobacteria|Pseudomonadales|Moraxellaceae|Acinetobacter	0.087462	0.086009	0.040568	0	22.97681	25.95043	10.61378	33.56732	34.48268	30.67585	27.57919	0	0.055011	0	0.647122	0.471356	0.05303	5.527165	0	0	15.56205	10.59199	Oil sands core	2.39E-06
Bacteria|Proteobacteria|Gammaproteobacteria|Pseudomonadales|Moraxellaceae|Acinetobacter|rhizosphaerae	0	0	0	0	0	0	0.112315	0	0.050859	0	0.051646	0	0	0	0.647122	0.052373	0.05303	0.619043	0	0	0.053478	0		
Bacteria|Proteobacteria|Gammaproteobacteria|Pseudomonadales|Moraxellaceae|Alkanindiges	0.131194	0	0	0	0.052699	0	0.056158	0	0	0	0	0	0	0	0	0	0	0	0	0	0.106956	0		
Bacteria|Proteobacteria|Gammaproteobacteria|Pseudomonadales|Moraxellaceae|Perlucidibaca	0.481043	0	0	0	0	0	0	0	0	0	0	0	0	0	0	0	0	0	0	0	0	0		
Bacteria|Proteobacteria|Gammaproteobacteria|Pseudomonadales|Pseudomonadaceae	0.241979	0.014335	0.016227	0	0.281062	0.206776	0.748767	0.373536	0.525547	0.302722	0.292663	0.431499	0.73348	0.679664	0.092446	3.648643	0.247472	0.589564	0.642473	0.364732	0.74869	0.811301	Oil sands tailings pond median	2.21E-05
Bacteria|Proteobacteria|Gammaproteobacteria|Pseudomonadales|Pseudomonadaceae|Pseudomonas	0.241979	0.014335	0.016227	0	0.281062	0.206776	0.748767	0.373536	0.525547	0.302722	0.292663	0.431499	0.73348	0.679664	0.092446	3.631185	0.247472	0.574825	0.624117	0.364732	0.74869	0.781253	Oil sands tailings pond median	2.21E-05
Bacteria|Proteobacteria|Gammaproteobacteria|Pseudomonadales|Pseudomonadaceae|Pseudomonas|stutzeri	0	0	0	0	0	0	0	0	0	0	0	0	0	0	0	0	0	0	0	0	0	0		
Bacteria|Proteobacteria|Gammaproteobacteria|Pseudomonadales|Pseudomonadaceae|Pseudomonas|viridiflava	0.102039	0	0	0	0	0	0	0.016979	0	0	0	0	0	0	0	0	0	0	0	0	0	0		
Bacteria|Proteobacteria|Gammaproteobacteria|PYR10d3	0	0	0	0	0	0	0	0	0	0	0	1.493652	2.530505	0.198926	0	1.780677	0.159089	0.044217	4.405529	2.937055	0.106956	0.045072	Oil sands tailings pond deep	9.06E-07
Bacteria|Proteobacteria|Gammaproteobacteria|Thiotrichales	0	0.02867	0	0	0	0	0	0	0	0	0	0	0	0	0	0	0.106059	0	0	0	0	0		
Bacteria|Proteobacteria|Gammaproteobacteria|Thiotrichales|Piscirickettsiaceae	0	0.02867	0	0	0	0	0	0	0	0	0	0	0	0	0	0	0.106059	0	0	0	0	0		
Bacteria|Proteobacteria|Gammaproteobacteria|Thiotrichales|Thiotrichaceae	0	0	0	0	0	0	0	0	0	0	0	0	0	0	0	0	0	0	0	0	0	0		
Bacteria|Proteobacteria|Gammaproteobacteria|Xanthomonadales	0.466466	0.02867	0.040568	0.823874	0.895885	1.378509	0.355664	0.747072	0.356016	1.967694	1.325591	1.294498	2.200439	0.745972	0.046223	0.209491	0.265148	0	1.101382	0.403125	0.534778	0.090145	Marine sediments	5.94E-08
Bacteria|Proteobacteria|Gammaproteobacteria|Xanthomonadales|Sinobacteraceae	0.218656	0	0.040568	0.053731	0.158097	0.775411	0.168473	0.509368	0.356016	1.160435	0.929635	0	0	0	0	0	0	0	0	0	0.320867	0.045072	India oil refineries	1.2E-05
Bacteria|Proteobacteria|Gammaproteobacteria|Xanthomonadales|Sinobacteraceae|Hydrocarboniphaga	0	0	0	0.053731	0	0	0	0	0	0	0	0	0	0	0	0	0	0	0	0	0	0		
Bacteria|Proteobacteria|Gammaproteobacteria|Xanthomonadales|Sinobacteraceae|Hydrocarboniphaga|effusa	0	0	0	0.053731	0	0	0	0	0	0	0	0	0	0	0	0	0	0	0	0	0	0		
Bacteria|Proteobacteria|Gammaproteobacteria|Xanthomonadales|Sinobacteraceae|Nevskia	0.218656	0	0.040568	0	0	0.155082	0.056158	0.509368	0.101719	0.050454	0.413171	0	0	0	0	0	0	0	0	0	0.267389	0.045072		
Bacteria|Proteobacteria|Gammaproteobacteria|Xanthomonadales|Xanthomonadaceae	0.24781	0.02867	0	0.770143	0.737788	0.603098	0.187192	0.237705	0	0.807259	0.395956	1.294498	2.200439	0.745972	0.046223	0.209491	0.265148	0	1.101382	0.403125	0.213911	0.045072	Marine sediments	8.38E-08
Bacteria|Proteobacteria|Gammaproteobacteria|Xanthomonadales|Xanthomonadaceae|Arenimonas	0	0	0	0	0	0	0	0	0	0	0	0	0	0.248657	0	0	0	0	0	0.057589	0	0.045072		
Bacteria|Proteobacteria|Gammaproteobacteria|Xanthomonadales|Xanthomonadaceae|Dokdonella	0.087462	0	0	0.268654	0	0	0	0	0	0	0	0	0	0	0	0	0	0	0	0	0	0		
Bacteria|Proteobacteria|Gammaproteobacteria|Xanthomonadales|Xanthomonadaceae|Dyella	0	0	0	0	0	0	0	0	0	0.454083	0	0	0	0	0	0	0	0	0	0	0	0		
Bacteria|Proteobacteria|Gammaproteobacteria|Xanthomonadales|Xanthomonadaceae|Dyella|ginsengisoli	0	0	0	0	0	0	0	0	0	0.454083	0	0	0	0	0	0	0	0	0	0	0	0		
Bacteria|Proteobacteria|Gammaproteobacteria|Xanthomonadales|Xanthomonadaceae|Luteibacter	0	0	0	0.322385	0	0	0	0	0	0	0	0	0	0	0	0	0	0	0	0	0	0		
Bacteria|Proteobacteria|Gammaproteobacteria|Xanthomonadales|Xanthomonadaceae|Luteibacter|rhizovicinus	0	0	0	0.322385	0	0	0	0	0	0	0	0	0	0	0	0	0	0	0	0	0	0		
Bacteria|Proteobacteria|Gammaproteobacteria|Xanthomonadales|Xanthomonadaceae|Luteimonas	0.131194	0	0	0	0	0	0	0	0	0	0	0	0	0	0	0	0	0	0	0	0	0		
Bacteria|Proteobacteria|Gammaproteobacteria|Xanthomonadales|Xanthomonadaceae|Pseudoxanthomonas	0	0	0	0	0	0	0	0	0	0	0	0	0	0	0	0	0	0	0	0	0	0		
Bacteria|Proteobacteria|Gammaproteobacteria|Xanthomonadales|Xanthomonadaceae|Pseudoxanthomonas|mexicana	0	0	0	0	0	0	0	0	0	0	0	0	0	0	0	0	0	0	0	0	0	0		
Bacteria|SAR406	0	0	0	0	0	0	0	0	0	0	0	0.099577	0	0	0	0	0	2.564605	0	0	0.427823	1.893036		
Bacteria|SAR406|AB16	0	0	0	0	0	0	0	0	0	0	0	0.099577	0	0	0	0	0	2.564605	0	0	0.320867	1.802891		
Bacteria|SAR406|AB16|Arctic96B-7	0	0	0	0	0	0	0	0	0	0	0	0	0	0	0	0	0	0	0	0	0	0		
Bacteria|SAR406|AB16|Arctic96B-7|A714017	0	0	0	0	0	0	0	0	0	0	0	0	0	0	0	0	0	0	0	0	0	0		
Bacteria|SAR406|AB16|Arctic96B-7|A714017|so4B24	0	0	0	0	0	0	0	0	0	0	0	0	0	0	0	0	0	0	0	0	0	0		
Bacteria|SAR406|AB16|noFP_H7	0	0	0	0	0	0	0	0	0	0	0	0.099577	0	0	0	0	0	2.564605	0	0	0.320867	1.802891		
Bacteria|SBR1093	0	0	0	0	0	0	0	0	0	0	0	0	0	0	0	0	0	0	0	0	0	0		
Bacteria|SBR1093|EC214	0	0	0	0	0	0	0	0	0	0	0	0	0	0	0	0	0	0	0	0	0	0		
Bacteria|Spirochaetes	0	0	0	0	0.02635	0.025847	0	0	0	0	0	0.647249	0.91685	0.853724	0.824311	1.152203	0.176765	1.473911	0.587404	0.287947	0.249563	1.352168	Mangrove	1.71E-07
Bacteria|Spirochaetes|Spirochaetes	0	0	0	0	0	0	0	0	0	0	0	0.647249	0.91685	0.779127	0.793495	1.082373	0.176765	1.473911	0.587404	0.287947	0.249563	1.352168	Mangrove	5.74E-08
Bacteria|Spirochaetes|Spirochaetes|Spirochaetales	0	0	0	0	0	0	0	0	0	0	0	0.647249	0.91685	0.729395	0.770384	1.082373	0.176765	1.473911	0.587404	0.287947	0.142608	1.352168	Mangrove	6.01E-08
Bacteria|Spirochaetes|Spirochaetes|Spirochaetales|Spirochaetaceae	0	0	0	0	0	0	0	0	0	0	0	0.647249	0.91685	0.729395	0.770384	1.082373	0.176765	1.473911	0.587404	0.287947	0.142608	1.352168	Mangrove	6.01E-08
Bacteria|Spirochaetes|Spirochaetes|Spirochaetales|Spirochaetaceae|Treponema	0	0	0	0	0	0	0	0	0	0	0	0.29873	0.476762	0.530469	0.677938	0.349152	0.070706	1.297041	0.036713	0	0.142608	1.352168	Oil sands tailings pond median	1.28E-05
Bacteria|Synergistetes	0	0	0	0	0	0	0.168473	0	0	0	0	0.149365	0.165033	1.34275	0.832015	0.104746	0.265148	1.503389	0	0	0.374345	0.40565		
Bacteria|Synergistetes|Synergistia	0	0	0	0	0	0	0.168473	0	0	0	0	0.149365	0.165033	1.34275	0.832015	0.104746	0.265148	1.503389	0	0	0.374345	0.40565		
Bacteria|Synergistetes|Synergistia|Synergistales	0	0	0	0	0	0	0.168473	0	0	0	0	0.149365	0.165033	1.34275	0.832015	0.104746	0.265148	1.503389	0	0	0.374345	0.40565		
Bacteria|Synergistetes|Synergistia|Synergistales|Thermovirgaceae	0	0	0	0	0	0	0.112315	0	0	0	0	0.099577	0.165033	1.243287	0.369784	0.104746	0.265148	1.503389	0	0	0.374345	0.40565		
Bacteria|Tenericutes	0	0	0	0	0	0	0.056158	0	0	0	0	0.29873	0.055011	3.530936	4.067626	0.942712	0.212119	0.088435	0	0	0.053478	0.135217		
Bacteria|Tenericutes|Mollicutes	0	0	0	0	0	0	0.056158	0	0	0	0	0.248942	0.055011	2.735232	3.281835	0.942712	0.106059	0.088435	0	0	0.053478	0.135217		
Bacteria|Tenericutes|Mollicutes|Acholeplasmatales	0	0	0	0	0	0	0.056158	0	0	0	0	0.248942	0.055011	2.735232	3.235612	0.942712	0.106059	0.088435	0	0	0.053478	0.135217		
Bacteria|Tenericutes|Mollicutes|Acholeplasmatales|Acholeplasmataceae	0	0	0	0	0	0	0.056158	0	0	0	0	0.248942	0.055011	2.735232	3.235612	0.942712	0.106059	0.088435	0	0	0.053478	0.135217		
Bacteria|Tenericutes|Mollicutes|Acholeplasmatales|Acholeplasmataceae|Acholeplasma	0	0	0	0	0	0	0.056158	0	0	0	0	0.248942	0.055011	2.735232	3.235612	0.890339	0.106059	0.088435	0	0	0.053478	0.135217		
Bacteria|Tenericutes|RF3	0	0	0	0	0	0	0	0	0	0	0	0.049788	0	0.795704	0.785791	0	0.106059	0	0	0	0	0		

Table S2. VI

Bacterial clades	Habitat																																																																		Differentially abundant class (one class test, non-strict)	p-value
	India oil refineries				Arctic												China oil refineries									Mangrove			Marine sediments							Taiga upper active layer				Taiga bottom active layer				Taiga permafrost layer				Oil sands core							Oil sands tailings pond upper				Oil sands tailings pond median			Oil sands tailings pond deep					
	I1	I2	I3	I4	A1	A10	A11	A12	A2	A3	A4	A5	A6	A7	A8	A9	C1	C2	C3	C4	C5	C6	C7	C8	C9	M1	M2	M3	DWH1	DWH2	DWH3	DWH4	DWH5	DWH6	DWH7	Tu1	Tu2	Tu3	Tu4	Tb1	Tb2	Tb3	Tb4	Tp1	Tp2	Tp3	Tp4	OSC1	OSC12	OSC3	OSC4	OSC5	OSC7	OSC9	OSTPu1	OSTPu2	OSTPu4	OSTPu3	OSTPm2	OSTPm6	OSTPm4	OSTPd1	OSTPd2	OSTPd3	OSTPd4		
Bacteria|Tenericutes|RF3|ML615J-28	0	0	0	0	0	0	0	0	0	0	0	0	0	0	0	0	0	0	0	0	0	0	0	0	0	0	0	0	0	0	0	0	0	0	0	0	0	0	0	0	0	0	0	0	0	0	0	0	0	0	0	0	0	0	0.049788	0	0.795704	0.785791	0	0.106059	0	0	0	0	0		
Bacteria|Thermi	0	0	0.999578	0.085326	0	0	0	0	0	0	0	0	0	0	0	0	0.054864	0.193234	0.13374	0.143389	0.238926	0.312709	0.166929	0.230598	0.098137	0.07801	0.054181	0.342129	0	0	0	0	0	0	0	0	0	0	0	0	0	0	0	0	0	0	0	0	0	0	0	0	0	0	0	0.055011	0.99463	0.277338	0	1.060593	0.088435	0	0	0.08913	0.946518		
Bacteria|Thermi|Deinococci	0	0	0.999578	0.085326	0	0	0	0	0	0	0	0	0	0	0	0	0.054864	0.193234	0.13374	0.143389	0.238926	0.312709	0.166929	0.230598	0.098137	0.07801	0.054181	0.342129	0	0	0	0	0	0	0	0	0	0	0	0	0	0	0	0	0	0	0	0	0	0	0	0	0	0	0	0.055011	0.99463	0.277338	0	1.060593	0.088435	0	0	0.08913	0.946518		
Bacteria|Thermi|Deinococci|Thermales	0	0	0.999578	0.085326	0	0	0	0	0	0	0	0	0	0	0	0	0	0.039979	0	0	0.071678	0	0	0.069179	0.042059	0	0	0	0	0	0	0	0	0	0	0	0	0	0	0	0	0	0	0	0	0	0	0	0	0	0	0	0	0	0	0.055011	0.99463	0.277338	0	1.060593	0.088435	0	0	0.053478	0.946518		
Bacteria|Thermi|Deinococci|Thermales|Thermaceae	0	0	0.999578	0.085326	0	0	0	0	0	0	0	0	0	0	0	0	0	0.039979	0	0	0.071678	0	0	0.069179	0.042059	0	0	0	0	0	0	0	0	0	0	0	0	0	0	0	0	0	0	0	0	0	0	0	0	0	0	0	0	0	0	0.055011	0.99463	0.277338	0	1.060593	0.088435	0	0	0.053478	0.946518		
Bacteria|Thermi|Deinococci|Thermales|Thermaceae|Thermus	0	0	0	0	0	0	0	0	0	0	0	0	0	0	0	0	0	0.039979	0	0	0	0	0	0	0.042059	0	0	0	0	0	0	0	0	0	0	0	0	0	0	0	0	0	0	0	0	0	0	0	0	0	0	0	0	0	0	0.055011	0.99463	0.277338	0	1.060593	0.088435	0	0	0.053478	0.946518		
Bacteria|Thermotogae	0.035575	0.077463	0.2173	0	0	0	0	0	0	0	0	0	0	0	0	0	0	0	0	0.074812	0	0	0	0	0.042059	0	0	0	0	0	0	0	0	0	0	0	0	0	0	0	0	0	0	0	0	0	0	0	0	0.112315	0	0	0	0	0	0	0.149194	3.05072	0	0.05303	0	0	0	0	0.135217		
Bacteria|Thermotogae|Thermotogae	0.035575	0.077463	0.2173	0	0	0	0	0	0	0	0	0	0	0	0	0	0	0	0	0.074812	0	0	0	0	0.042059	0	0	0	0	0	0	0	0	0	0	0	0	0	0	0	0	0	0	0	0	0	0	0	0	0.112315	0	0	0	0	0	0	0.149194	3.05072	0	0.05303	0	0	0	0	0.135217		
Bacteria|Thermotogae|Thermotogae|Thermotogales	0.035575	0.077463	0.2173	0	0	0	0	0	0	0	0	0	0	0	0	0	0	0	0	0.074812	0	0	0	0	0.042059	0	0	0	0	0	0	0	0	0	0	0	0	0	0	0	0	0	0	0	0	0	0	0	0	0.112315	0	0	0	0	0	0	0.149194	3.05072	0	0.05303	0	0	0	0	0.135217		
Bacteria|Thermotogae|Thermotogae|Thermotogales|Thermotogaceae	0.035575	0.077463	0.2173	0	0	0	0	0	0	0	0	0	0	0	0	0	0	0	0	0.074812	0	0	0	0	0.042059	0	0	0	0	0	0	0	0	0	0	0	0	0	0	0	0	0	0	0	0	0	0	0	0	0.112315	0	0	0	0	0	0	0.149194	3.05072	0	0.05303	0	0	0	0	0.135217		
Bacteria|Thermotogae|Thermotogae|Thermotogales|Thermotogaceae|Fervidobacterium	0.035575	0.077463	0.13038	0	0	0	0	0	0	0	0	0	0	0	0	0	0	0	0	0	0	0	0	0	0.042059	0	0	0	0	0	0	0	0	0	0	0	0	0	0	0	0	0	0	0	0	0	0	0	0	0	0	0	0	0	0	0	0	0.693345	0	0	0	0	0	0	0.045072		
Bacteria|Thermotogae|Thermotogae|Thermotogales|Thermotogaceae|Thermotoga	0	0	0	0	0	0	0	0	0	0	0	0	0	0	0	0	0	0	0	0.074812	0	0	0	0	0	0	0	0	0	0	0	0	0	0	0	0	0	0	0	0	0	0	0	0	0	0	0	0	0	0	0	0	0	0	0	0	0	2.218705	0	0	0	0	0	0	0		
Bacteria|TM6	0	0.038732	0.04346	0.042663	0.046566	0	0	0	0	0	0	0	0	0	0	0	0.047027	0.039979	0	0.112218	0	0.042642	0	0	0	0.273035	0.121908	0.078953	0.594085	0	0	0	0	0	0.043315	0	0	0	0	0	0	0	0	0	0	0	0	0	0	0	0.305621	0	0	0	0	0	0.049731	0	0	0	0	0	0	0	0.135217		
Bacteria|TM6|SJA-4	0	0.038732	0.04346	0	0.046566	0	0	0	0	0	0	0	0	0	0	0	0.047027	0.039979	0	0.112218	0	0	0	0	0	0.117015	0.121908	0.078953	0.594085	0	0	0	0	0	0.043315	0	0	0	0	0	0	0	0	0	0	0	0	0	0	0	0.305621	0	0	0	0	0	0.049731	0	0	0	0	0	0	0	0.135217		
Bacteria|TM7	0.284603	0.46478	0.608439	0.725274	0.698487	0.127762	0.049093	0.047582	0.162227	0.094024	0.046017	0.517582	0.045781	0.046803	0.795423	0	0	0.039979	0.084467	0.037406	0	0.127926	0.040063	0.03459	0.084118	0	0.081272	0.039476	0	0	0	0	0	0	0	0.047417	0	4.34022	0.041712	10.94515	0.604528	1.533401	0	1.049549	0.602061	0.202842	0	0	0	0	0	0	0	0	0	0	0	0	0	0	0	0	0	0	0	Taiga bottom active layer	2.02E-05
Bacteria|TM7|SC3	0	0	0	0	0.698487	0.085175	0.049093	0.047582	0.162227	0.094024	0.046017	0.431319	0.045781	0	0.795423	0	0	0	0.042234	0	0	0	0	0	0	0	0	0	0	0	0	0	0	0	0	0.047417	0	0	0	0	0	1.192645	0	0.043731	0	0	0	0	0	0	0	0	0	0	0	0	0	0	0	0	0	0	0	0	0		
Bacteria|TM7|TM7-1	0.249028	0.348585	0	0.298642	0	0	0	0	0	0	0	0.086264	0	0.046803	0	0	0	0	0	0	0	0.042642	0.040063	0	0	0	0.081272	0.039476	0	0	0	0	0	0	0	0	0	4.34022	0	10.84238	0.431806	0.298161	0	0.043731	0	0.202842	0	0	0	0	0	0	0	0	0	0	0	0	0	0	0	0	0	0	0		
Bacteria|TM7|TM7-3	0.035575	0.116195	0.608439	0.426632	0	0.042587	0	0	0	0	0	0	0	0	0	0	0	0.039979	0.042234	0	0	0.085284	0	0.03459	0.084118	0	0	0	0	0	0	0	0	0	0	0	0	0	0.041712	0.102771	0.172722	0.042594	0	0.918355	0.602061	0	0	0	0	0	0	0	0	0	0	0	0	0	0	0	0	0	0	0	0		
Bacteria|TM7|TM7-3|EW055	0.035575	0	0.564979	0.255979	0	0	0	0	0	0	0	0	0	0	0	0	0	0.039979	0.042234	0	0	0.042642	0	0	0.042059	0	0	0	0	0	0	0	0	0	0	0	0	0	0	0.102771	0.043181	0	0	0.043731	0.559057	0	0	0	0	0	0	0	0	0	0	0	0	0	0	0	0	0	0	0	0		
Bacteria|Verrucomicrobia	0.071151	0	0.08692	0.341305	0	0.085175	0	0	0.081113	0.282072	0.184067	0.862637	0.183125	0	0.167457	0.083149	3.432948	9.435133	4.138889	14.06463	10.53665	3.581942	10.5766	25.66556	5.131189	5.53871	2.763246	7.105764	3.747307	2.711824	3.312661	5.322971	2.100389	20.6707	4.85128	1.138006	0	0	0	0	0.086361	0.681512	0	0	0	0.48682	0	0	0	0.561575	0	0	0.201815	0.309878	0.099577	0	0.099463	0.092446	0.104746	0	0.353739	0.110138	0	0.106956	0	China oil refineries	1.53E-05
Bacteria|Verrucomicrobia|Opitutae	0.071151	0	0.08692	0.255979	0	0	0	0	0	0	0	0	0	0	0	0	0.28216	2.478721	1.604875	3.890217	1.36188	0.426422	2.003143	5.603531	1.850593	1.48219	0.731448	1.026388	0	0.090394	0.37859	0.190106	0.095472	0	0.17326	0	0	0	0	0	0	0	0	0	0	0	0	0	0	0.561575	0	0	0.201815	0.309878	0	0	0	0	0	0	0	0	0	0	0	China oil refineries	3.48E-07
Bacteria|Verrucomicrobia|Opitutae|Opitutales	0.071151	0	0.08692	0.255979	0	0	0	0	0	0	0	0	0	0	0	0	0.28216	2.478721	1.604875	3.815405	1.36188	0.426422	1.923018	5.603531	1.850593	0.15602	0.081272	0.236859	0	0	0.189295	0	0	0	0.08663	0	0	0	0	0	0	0	0	0	0	0	0	0	0	0.112315	0	0	0.100907	0	0	0	0	0	0	0	0	0	0	0	0	China oil refineries	3.76E-07
Bacteria|Verrucomicrobia|Opitutae|Opitutales|Opitutaceae	0.071151	0	0.08692	0.255979	0	0	0	0	0	0	0	0	0	0	0	0	0.28216	2.478721	1.604875	3.815405	1.36188	0.426422	1.923018	5.603531	1.850593	0.15602	0.081272	0.236859	0	0	0.189295	0	0	0	0.08663	0	0	0	0	0	0	0	0	0	0	0	0	0	0	0.112315	0	0	0.100907	0	0	0	0	0	0	0	0	0	0	0	0	China oil refineries	3.76E-07
Bacteria|Verrucomicrobia|Opitutae|Opitutales|Opitutaceae|Opitutus	0	0	0.08692	0.255979	0	0	0	0	0	0	0	0	0	0	0	0	0.188107	2.398763	1.520408	3.665781	1.36188	0.426422	1.923018	5.465172	1.514121	0	0	0	0	0	0	0	0	0	0.08663	0	0	0	0	0	0	0	0	0	0	0	0	0	0	0.112315	0	0	0.100907	0	0	0	0	0	0	0	0	0	0	0	0	China oil refineries	7.33E-07
Bacteria|Verrucomicrobia|Opitutae|Puniceicoccales	0	0	0	0	0	0	0	0	0	0	0	0	0	0	0	0	0	0	0	0	0	0	0	0	0	1.17015	0.487632	0.789529	0	0.090394	0.189295	0.190106	0.095472	0	0.08663	0	0	0	0	0	0	0	0	0	0	0	0	0	0	0	0	0	0	0	0	0	0	0	0	0	0	0	0	0	0		
Bacteria|Verrucomicrobia|Opitutae|Puniceicoccales|Puniceicoccaceae	0	0	0	0	0	0	0	0	0	0	0	0	0	0	0	0	0	0	0	0	0	0	0	0	0	1.17015	0.487632	0.789529	0	0.090394	0.189295	0.190106	0.095472	0	0.08663	0	0	0	0	0	0	0	0	0	0	0	0	0	0	0	0	0	0	0	0	0	0	0	0	0	0	0	0	0	0		
Bacteria|Verrucomicrobia|Opitutae|Puniceicoccales|Puniceicoccaceae|Coraliomargarita	0	0	0	0	0	0	0	0	0	0	0	0	0	0	0	0	0	0	0	0	0	0	0	0	0	1.17015	0.487632	0.789529	0	0.090394	0.189295	0.190106	0.095472	0	0.08663	0	0	0	0	0	0	0	0	0	0	0	0	0	0	0	0	0	0	0	0	0	0	0	0	0	0	0	0	0	0		
Bacteria|Verrucomicrobia|Pedosphaerae	0	0	0	0.085326	0	0.085175	0	0	0	0.094024	0	0.258791	0.091562	0	0.083729	0.083149	0.094053	0.479753	0.760204	0.897742	0.860135	0.085284	1.842892	1.591126	0.672943	1.01413	0.731448	1.736965	0	0	0	0.095053	0.095472	0	0.51978	0.094834	0	0	0	0	0	0	0	0	0	0	0	0	0	0	0	0	0	0	0.099577	0	0	0	0	0	0.265304	0.110138	0	0	0	Mangrove	8.98E-05
Bacteria|Verrucomicrobia|Pedosphaerae|Pedosphaerales	0	0	0	0.085326	0	0.085175	0	0	0	0.094024	0	0.258791	0.091562	0	0.083729	0.083149	0.094053	0.479753	0.760204	0.897742	0.860135	0.085284	1.842892	1.591126	0.672943	1.01413	0.731448	1.736965	0	0	0	0.095053	0.095472	0	0.51978	0.094834	0	0	0	0	0	0	0	0	0	0	0	0	0	0	0	0	0	0	0.099577	0	0	0	0	0	0.265304	0.110138	0	0	0	Mangrove	8.98E-05
Bacteria|Verrucomicrobia|Pedosphaerae|Pedosphaerales|Ellin517	0	0	0	0	0	0	0	0	0	0	0	0	0	0	0	0	0	0.159918	0	0.224436	0.430067	0	1.201886	0.968512	0.252354	0	0	0	0	0	0	0	0	0	0	0	0	0	0	0	0	0	0	0	0	0	0	0	0	0	0	0	0	0	0	0	0	0	0	0	0	0	0	0	0		
Bacteria|Verrucomicrobia|Spartobacteria	0	0	0	0	0	0	0	0	0.081113	0.188048	0.184067	0.603846	0.091562	0	0.083729	0	2.539441	4.157855	1.098073	4.264276	5.662554	2.899667	6.490184	10.37691	1.682357	0.07801	0.162544	0.315812	0.913977	0.180788	0.189295	0.095053	0.095472	0.691906	0	0.758671	0	0	0	0	0.086361	0.681512	0	0	0	0.48682	0	0	0	0	0	0	0	0	0	0	0	0	0	0	0	0	0	0	0	China oil refineries	6.35E-06
Bacteria|Verrucomicrobia|Spartobacteria|Chthoniobacterales	0	0	0	0	0	0	0	0	0.081113	0.188048	0.184067	0.603846	0.091562	0	0.083729	0	2.539441	4.157855	1.098073	4.264276	5.662554	2.899667	6.490184	10.37691	1.682357	0.07801	0.162544	0.315812	0.913977	0.180788	0.189295	0.095053	0.095472	0.691906	0	0.758671	0	0	0	0	0.086361	0.681512	0	0	0	0.48682	0	0	0	0	0	0	0	0	0	0	0	0	0	0	0	0	0	0	0	China oil refineries	6.35E-06
Bacteria|Verrucomicrobia|Spartobacteria|Chthoniobacterales|Chthoniobacteraceae	0	0	0	0	0	0	0	0	0.081113	0.188048	0.184067	0.603846	0.091562	0	0.083729	0	2.539441	4.157855	1.098073	4.264276	5.662554	2.899667	6.490184	10.37691	1.682357	0.07801	0.162544	0.315812	0.913977	0.180788	0.189295	0.095053	0.095472	0.691906	0	0.758671	0	0	0	0	0.086361	0.681512	0	0	0	0.48682	0	0	0	0	0	0	0	0	0	0	0	0	0	0	0	0	0	0	0	China oil refineries	6.35E-06
Bacteria|Verrucomicrobia|Spartobacteria|Chthoniobacterales|Chthoniobacteraceae|Candidatus_Xiphinematobacter	0	0	0	0	0	0	0	0	0	0	0	0	0	0	0	0	0.188107	0.719629	0.253401	0.748119	0.645101	0.170569	0.480754	0.069179	0.168236	0	0	0	0.548386	0.090394	0	0.095053	0	0.345953	0	0.094834	0	0	0	0	0	0	0	0	0	0	0	0	0	0	0	0	0	0	0	0	0	0	0	0	0	0	0	0	0		
Bacteria|Verrucomicrobia|Spartobacteria|Chthoniobacterales|Chthoniobacteraceae|Chthoniobacter	0	0	0	0	0	0	0	0	0	0	0.092034	0	0	0	0	0	0.28216	1.119423	0.168934	0.523683	1.146846	0.852843	1.522389	3.113073	0.252354	0	0	0	0	0	0	0	0	0	0	0.474169	0	0	0	0	0.086361	0	0	0	0	0	0	0	0	0	0	0	0	0	0	0	0	0	0	0	0	0	0	0	0	China oil refineries	3.98E-07
Bacteria|Verrucomicrobia|Spartobacteria|Chthoniobacterales|Chthoniobacteraceae|DA101	0	0	0	0	0	0	0	0	0	0	0	0	0	0	0	0	1.316747	0.159918	0.168934	0.673307	1.290202	0.511706	2.003143	0.691794	0.168236	0	0	0.157906	0.091398	0	0	0	0	0	0	0.189668	0	0	0	0	0	0.681512	0	0	0	0.48682	0	0	0	0	0	0	0	0	0	0	0	0	0	0	0	0	0	0	0	China oil refineries	5.94E-06
Bacteria|Verrucomicrobia|Spartobacteria|Chthoniobacterales|Chthoniobacteraceae|Ellin506	0	0	0	0	0	0	0	0	0	0	0	0	0	0	0	0	0.28216	0.159918	0.168934	0.074812	0.215034	0.255853	0	0.830153	0.084118	0	0	0	0	0	0	0	0	0	0	0	0	0	0	0	0	0	0	0	0	0	0	0	0	0	0	0	0	0	0	0	0	0	0	0	0	0	0	0	0		
Bacteria|Verrucomicrobia|Verruco-5	0	0	0	0	0	0	0	0	0	0	0	0	0	0	0	0	0	0	0	0	0	0	0	0	0	1.63821	0.568904	2.763353	0.274193	0.090394	0.094647	0.855477	0.190944	0	0.34652	0	0	0	0	0	0	0	0	0	0	0	0	0	0	0	0	0	0	0	0	0	0.099463	0.092446	0.104746	0	0	0	0	0	0		
Bacteria|Verrucomicrobia|Verruco-5|LD1-PB3	0	0	0	0	0	0	0	0	0	0	0	0	0	0	0	0	0	0	0	0	0	0	0	0	0	0.62408	0	0.157906	0	0	0	0	0	0	0	0	0	0	0	0	0	0	0	0	0	0	0	0	0	0	0	0	0	0	0	0	0.099463	0.092446	0.104746	0	0	0	0	0	0		
Bacteria|Verrucomicrobia|Verruco-5|R76-B128	0	0	0	0	0	0	0	0	0	0	0	0	0	0	0	0	0	0	0	0	0	0	0	0	0	0.07801	0.081272	0.078953	0.274193	0.090394	0.094647	0.760424	0.095472	0	0.25989	0	0	0	0	0	0	0	0	0	0	0	0	0	0	0	0	0	0	0	0	0	0	0	0	0	0	0	0	0	0		
Bacteria|Verrucomicrobia|Verruco-5|SS1-B-03-39	0	0	0	0	0	0	0	0	0	0	0	0	0	0	0	0	0	0	0	0	0	0	0	0	0	0.54607	0.243816	2.210682	0	0	0	0	0	0	0	0	0	0	0	0	0	0	0	0	0	0	0	0	0	0	0	0	0	0	0	0	0	0	0	0	0	0	0	0	0		
Bacteria|Verrucomicrobia|Verrucomicrobiae	0	0	0	0	0	0	0	0	0	0	0	0	0	0	0	0	0.28216	2.318804	0.675737	5.012394	2.652082	0.170569	0.240377	8.09399	0.925296	1.32617	0.568904	1.263247	2.559137	2.350247	2.650129	4.087281	1.623028	19.97879	3.81172	0.189668	0	0	0	0	0	0	0	0	0	0	0	0	0	0	0	0	0	0	0	0	0	0	0	0	0.088435	0	0	0.106956	0	Marine sediments	2.57E-08
Bacteria|Verrucomicrobia|Verrucomicrobiae|Verrucomicrobiales	0	0	0	0	0	0	0	0	0	0	0	0	0	0	0	0	0.28216	2.318804	0.675737	5.012394	2.652082	0.170569	0.240377	8.09399	0.925296	1.32617	0.568904	1.263247	2.559137	2.350247	2.650129	4.087281	1.623028	19.97879	3.81172	0.189668	0	0	0	0	0	0	0	0	0	0	0	0	0	0	0	0	0	0	0	0	0	0	0	0	0.088435	0	0	0.106956	0	Marine sediments	2.57E-08
Bacteria|Verrucomicrobia|Verrucomicrobiae|Verrucomicrobiales|Verrucomicrobiaceae	0	0	0	0	0	0	0	0	0	0	0	0	0	0	0	0	0.28216	2.318804	0.675737	5.012394	2.652082	0.170569	0.240377	8.09399	0.925296	1.32617	0.568904	1.263247	2.559137	2.350247	2.650129	4.087281	1.623028	19.97879	3.81172	0.189668	0	0	0	0	0	0	0	0	0	0	0	0	0	0	0	0	0	0	0	0	0	0	0	0	0.088435	0	0	0.106956	0	Marine sediments	2.57E-08
Bacteria|Verrucomicrobia|Verrucomicrobiae|Verrucomicrobiales|Verrucomicrobiaceae|Luteolibacter	0	0	0	0	0	0	0	0	0	0	0	0	0	0	0	0	0.28216	2.158886	0.59127	4.4139	2.508726	0.085284	0.240377	7.609734	0.757061	1.01413	0.40636	0.631623	1.645159	0.813547	0.37859	0.285159	0.095472	4.49739	1.21282	0	0	0	0	0	0	0	0	0	0	0	0	0	0	0	0	0	0	0	0	0	0	0	0	0	0	0	0	0.106956	0	China oil refineries	1.14E-08
Bacteria|Verrucomicrobia|Verrucomicrobiae|Verrucomicrobiales|Verrucomicrobiaceae|MSBL3	0	0	0	0	0	0	0	0	0	0	0	0	0	0	0	0	0	0.079959	0	0.598495	0	0	0	0	0.084118	0	0	0.078953	0	0	0	0	0	0.259465	0	0	0	0	0	0	0	0	0	0	0	0	0	0	0	0	0	0	0	0	0	0	0	0	0	0	0	0	0	0	0		
Bacteria|Verrucomicrobia|Verrucomicrobiae|Verrucomicrobiales|Verrucomicrobiaceae|Persicirhabdus	0	0	0	0	0	0	0	0	0	0	0	0	0	0	0	0	0	0	0	0	0	0	0	0	0	0.23403	0.081272	0.552671	0.639784	1.084729	1.987597	3.707069	1.336611	14.53003	2.33901	0	0	0	0	0	0	0	0	0	0	0	0	0	0	0	0	0	0	0	0	0	0	0	0	0	0	0	0	0	0	Marine sediments	1.82E-09
Bacteria|Verrucomicrobia|Verrucomicrobiae|Verrucomicrobiales|Verrucomicrobiaceae|Verrucomicrobium	0	0	0	0	0	0	0	0	0	0	0	0	0	0	0	0	0	0	0	0	0	0.085284	0	0.138359	0	0.07801	0.081272	0	0.182795	0.361576	0.094647	0.095053	0.190944	0.605418	0.08663	0	0	0	0	0	0	0	0	0	0	0	0	0	0	0	0	0	0	0	0	0	0	0	0	0	0	0	0	0	0		
Bacteria|WPS-2	0	0	0	0	0	0	0	0	0	0	0	0	0	0	0	0	0.42324	0.159918	0.59127	0.336653	0.394228	0.59699	0.360566	0.345897	0.420589	0	0	0	0	0	0	0	0	0	0	0	0	0	0	0	0	0.042594	0	0	0	0	0	0	0	0	0	0	0	0	0	0	0.049731	0	0	0	0	0	0	0	0		
Bacteria|WS3	0	0.077463	0	0.597284	0	0	0	0	0	0	0.092034	0	0	0	0	0	0	0.239876	0.084467	0.523683	0.215034	0	0.160251	0	0	2.88637	1.219079	4.105553	0.091398	0.813547	0.37859	0.570318	0.190944	0.086488	0.34652	0	0	0	0	0	0.086361	0	0	0	0	0	0	0	0	0	0	0	0	0	2.389843	1.760351	0.099463	0.092446	1.571186	0.318178	12.11555	0.110138	0	1.176513	2.704337	Oil sands tailings pond median	3.68E-06
Bacteria|WS3|PRR-12	0	0.077463	0	0.597284	0	0	0	0	0	0	0.092034	0	0	0	0	0	0	0.239876	0.084467	0.523683	0.215034	0	0.160251	0	0	2.88637	1.219079	4.105553	0.091398	0.813547	0.37859	0.570318	0.190944	0.086488	0.34652	0	0	0	0	0	0.086361	0	0	0	0	0	0	0	0	0	0	0	0	0	2.389843	1.760351	0.099463	0.092446	1.571186	0.318178	12.11555	0.110138	0	1.176513	2.704337	Oil sands tailings pond median	3.68E-06
Bacteria|WS3|PRR-12|GN03	0	0	0	0.255979	0	0	0	0	0	0	0	0	0	0	0	0	0	0	0	0	0	0	0	0	0	0.93612	0.243816	0.868482	0	0.813547	0.189295	0.475265	0.095472	0.086488	0.25989	0	0	0	0	0	0	0	0	0	0	0	0	0	0	0	0	0	0	0	0	0	0	0	0	0	0	0	0	0	0		
Bacteria|WS3|PRR-12|GN03|KSB4	0	0	0	0.255979	0	0	0	0	0	0	0	0	0	0	0	0	0	0	0	0	0	0	0	0	0	0.93612	0.243816	0.868482	0	0.813547	0.189295	0.475265	0.095472	0.086488	0.25989	0	0	0	0	0	0	0	0	0	0	0	0	0	0	0	0	0	0	0	0	0	0	0	0	0	0	0	0	0	0		
Bacteria|WS3|PRR-12|Sediment-1	0	0.077463	0	0.341305	0	0	0	0	0	0	0.092034	0	0	0	0	0	0	0.239876	0.084467	0.448871	0.143356	0	0.160251	0	0	1.09214	0.40636	1.736965	0.091398	0	0.094647	0	0	0	0	0	0	0	0	0	0.086361	0	0	0	0	0	0	0	0	0	0	0	0	0	0.099577	0	0	0	0	0	0	0	0	0	0	Mangrove	0.002235
Bacteria|WS3|PRR-12|Sediment-1|CV106	0	0	0	0	0	0	0	0	0	0	0	0	0	0	0	0	0	0	0	0	0	0	0	0	0	0.62408	0.162544	0.947435	0	0	0.094647	0	0	0	0	0	0	0	0	0	0	0	0	0	0	0	0	0	0	0	0	0	0	0	0	0	0	0	0	0	0	0	0	0	0		
Bacteria|WS3|PRR-12|SSS58A	0	0	0	0	0	0	0	0	0	0	0	0	0	0	0	0	0	0	0	0	0	0	0	0	0	0.23403	0.243816	0.473718	0	0	0	0	0	0	0	0	0	0	0	0	0	0	0	0	0	0	0	0	0	0	0	0	0	0	2.091113	1.760351	0.099463	0.092446	1.571186	0.318178	12.02711	0.110138	0	1.176513	2.704337	Oil sands tailings pond median	1.83E-08
Bacteria|WS6	0	0	0	0	0	0	0	0	0	0	0	0	0	0	0	0	0	0	0	0	0	0	0	0	0	0.039005	0	0	0	0	0	0	0	0	0	0	0	0	0	0	0	0	0	0	0	0	0	0	0	0	0	0	0	0	0.697038	0.165033	2.735232	0.508453	1.09983	0.212119	0.751694	0	0	0.320867	0.270434	Oil sands tailings pond upper	1.9E-07
Bacteria|WS6|SC72	0	0	0	0	0	0	0	0	0	0	0	0	0	0	0	0	0	0	0	0	0	0	0	0	0	0	0	0	0	0	0	0	0	0	0	0	0	0	0	0	0	0	0	0	0	0	0	0	0	0	0	0	0	0	0.697038	0.165033	2.735232	0.508453	1.09983	0.212119	0.751694	0	0	0.320867	0.270434	Oil sands tailings pond upper	4.82E-08
Bacteria|WS6|SC72|A-2AF	0	0	0	0	0	0	0	0	0	0	0	0	0	0	0	0	0	0	0	0	0	0	0	0	0	0	0	0	0	0	0	0	0	0	0	0	0	0	0	0	0	0	0	0	0	0	0	0	0	0	0	0	0	0	0.248942	0.110022	1.939528	0.416007	0.73322	0.159089	0.088435	0	0	0.106956	0		
Bacteria|WS6|SC72|WCHB1-15	0	0	0	0	0	0	0	0	0	0	0	0	0	0	0	0	0	0	0	0	0	0	0	0	0	0	0	0	0	0	0	0	0	0	0	0	0	0	0	0	0	0	0	0	0	0	0	0	0	0	0	0	0	0	0.448096	0.055011	0.795704	0.092446	0.36661	0.05303	0.66326	0	0	0.213911	0.270434		
Bacteria|WWE1	0	0	0	0	0	0	0	0	0	0	0	0	0	0	0	0	0	0	0	0	0	0	0	0	0	0.039005	0	0	0	0.225985	0.094647	0.522792	0.143208	0	0.043315	0	0	0	0	0	0	0	0	0	0	0	0	0	0	0	0	0	0	0	0	0	0.298389	0.184892	1.152203	0	0	0.220276	0.057589	0	0		
Bacteria|WWE1|Cloacamonae	0	0	0	0	0	0	0	0	0	0	0	0	0	0	0	0	0	0	0	0	0	0	0	0	0	0.039005	0	0	0	0.225985	0.094647	0.522792	0.143208	0	0.043315	0	0	0	0	0	0	0	0	0	0	0	0	0	0	0	0	0	0	0	0	0	0.298389	0.184892	1.152203	0	0	0.220276	0.057589	0	0		
Bacteria|WWE1|Cloacamonae|Cloacamonales	0	0	0	0	0	0	0	0	0	0	0	0	0	0	0	0	0	0	0	0	0	0	0	0	0	0.039005	0	0	0	0.225985	0.094647	0.522792	0.143208	0	0.043315	0	0	0	0	0	0	0	0	0	0	0	0	0	0	0	0	0	0	0	0	0	0.298389	0.184892	1.152203	0	0	0.220276	0.057589	0	0		
Bacteria|WWE1|Cloacamonae|Cloacamonales|SHA-116	0	0	0	0	0	0	0	0	0	0	0	0	0	0	0	0	0	0	0	0	0	0	0	0	0	0	0	0	0	0.225985	0.094647	0.522792	0.143208	0	0.043315	0	0	0	0	0	0	0	0	0	0	0	0	0	0	0	0	0	0	0	0	0	0.248657	0.184892	1.152203	0	0	0.220276	0.057589	0	0		

Table S2. VII

Supplementary data. Table S3. Functional profile of oil contaminated environments expressed in terms of KEGG functional modules. Relative abundances of KEGG modules as estimated by HUMAnN2 are described. Differentially abundant functional modules were detected by LEfSe using the non-strict strategy with a LDA cutoff of 2.0 and p-value < 0.01. Functional modules are labeled according to the habitat exhibiting the highest LDA value for pairwise comparison among all oil polluted environments. KEGG Functional Modules are further colored according to the habitat in which they were detected to be differentially abundant in by LefSe. Coloring was according to the legend given below.

- Arctic (A)
- China oil refineries (C)
- India oil refineries (I)
- Mangrove (M)
- Marine sediments (DWH)
- Oil sands core (OSC)
- Oil sands tailings pond deep (OSTPd)
- Oil sands tailings pond median (OSTPm)
- Oil sands tailings pond upper (OSTPu)
- Taiga bottom active layer (Tb)
- Taiga permafrost layer (Tp)
- Taiga upper active layer (Tu)

KEGG_Module	Habitat: India oil refineries				Arctic												China oil refineries									Mangrove			Marine sediments							Taiga upper active layer				Taiga bottom active layer				Taiga permafrost layer				Oil sands core							Oil sands tailings pond upper				Oil sands tailings pond median			Oil sands tailings pond deep				One-class test (strict, p < 0.01)	p value
	I1	I2	I3	I4	A1	A10	A11	A12	A2	A3	A4	A5	A6	A7	A8	A9	C1	C2	C3	C4	C5	C6	C7	C8	C9	M1	M2	M3	DWH1	DWH2	DWH3	DWH4	DWH5	DWH6	DWH7	Tu1	Tu2	Tu3	Tu4	Tb1	Tb2	Tb3	Tb4	Tp1	Tp2	Tp3	Tp4	OSC1	OSC12	OSC3	OSC4	OSC5	OSC7	OSC9	OSTPu1	OSTPu2	OSTPu4	OSTPu3	OSTPm2	OSTPm6	OSTPm4	OSTPd1	OSTPd2	OSTPd3	OSTPd4		
M00001: Glycolysis_(Embden-Meyerhof_pathway),_glucose_=>_pyruvate	0.00159	0.001607	0.001788	0.00166	0.001674	0.001731	0.001741	0.001788	0.001711	0.001814	0.001776	0.00173	0.001948	0.001888	0.001716	0.001642	0.001885	0.001827	0.001838	0.001855	0.001874	0.001619	0.001843	0.001833	0.001728	0.001883	0.001823	0.001827	0.001749	0.001642	0.001732	0.001626	0.001628	0.001653	0.001599	0.001392	0.001284	0.001822	0.001407	0.001343	0.001353	0.001744	0.001303	0.001356	0.001307	0.002025	0.001208	0.001273	0.001294	0.001259	0.001282	0.001243	0.001282	0.001298	0.001556	0.001458	0.001937	0.001802	0.001708	0.001935	0.001428	0.001463	0.001394	0.001399	0.001877	Mangrove	0.000102
M00002: Glycolysis,_core_module_involving_three-carbon_compounds	0.002001	0.002037	0.002243	0.002178	0.002319	0.002298	0.002334	0.002362	0.002194	0.002447	0.002423	0.002194	0.002521	0.002471	0.00226	0.002107	0.002556	0.002381	0.002486	0.002361	0.002381	0.002207	0.00239	0.002312	0.002309	0.002383	0.002373	0.002369	0.002311	0.002319	0.002358	0.002236	0.002287	0.002292	0.002211	0.001898	0.001809	0.002456	0.001863	0.001837	0.001957	0.002182	0.001826	0.001951	0.002036	0.002544	0.001701	0.001811	0.001829	0.001858	0.001841	0.001777	0.001819	0.001823	0.002094	0.001968	0.002565	0.002462	0.002277	0.002505	0.001982	0.001994	0.001899	0.001952	0.002524	Mangrove	0.000158
M00003: Gluconeogenesis,_oxaloacetate_=>_fructose-6P	0.001482	0.001559	0.00181	0.001714	0.001836	0.001807	0.001812	0.001819	0.001633	0.001854	0.00184	0.00166	0.001967	0.001948	0.001699	0.001588	0.001992	0.001709	0.001855	0.001689	0.001671	0.001675	0.001695	0.001632	0.001665	0.001826	0.001784	0.001799	0.001776	0.001796	0.001841	0.001716	0.001739	0.001712	0.001684	0.001503	0.001437	0.001875	0.001663	0.001491	0.001527	0.001538	0.001453	0.001533	0.001623	0.001791	0.001435	0.001507	0.001488	0.001396	0.001544	0.001511	0.001534	0.00152	0.001689	0.001591	0.001987	0.001901	0.001801	0.00197	0.00164	0.001602	0.001542	0.001602	0.00196	Oil sands tailings pond median	0.000749
M00004: Pentose_phosphate_pathway_(Pentose_phosphate_cycle)	0.001969	0.001723	0.001906	0.001764	0.001757	0.00195	0.001971	0.00202	0.002031	0.002048	0.001965	0.001989	0.002207	0.002136	0.00187	0.001929	0.001929	0.002032	0.002074	0.002066	0.002049	0.00188	0.002085	0.002012	0.001964	0.002066	0.002026	0.002023	0.001953	0.001774	0.00185	0.001763	0.00173	0.001742	0.001762	0.001662	0.001641	0.002128	0.001724	0.001558	0.001741	0.001942	0.001688	0.001749	0.001795	0.002272	0.001507	0.001622	0.001681	0.001586	0.001667	0.001626	0.00167	0.001643	0.001561	0.001404	0.001951	0.001823	0.001654	0.001918	0.001499	0.001395	0.001337	0.00154	0.001887	Mangrove	4.96E-05
M00005: PRPP_biosynthesis,_ribose_5P_=>_PRPP	0.002024	0.001918	0.002701	0.002519	0.001963	0.001999	0.002312	0.002077	0.002035	0.002175	0.00205	0.002046	0.002129	0.002063	0.002013	0.001973	0.002706	0.002443	0.002389	0.002543	0.002342	0.002235	0.00235	0.002362	0.002316	0.002387	0.0023	0.002303	0.002152	0.002203	0.002103	0.002066	0.001998	0.001959	0.002061	0.002017	0.00181	0.002215	0.001877	0.001971	0.002012	0.002011	0.001849	0.001997	0.00204	0.002211	0.0018	0.001772	0.001789	0.001091	0.001783	0.001788	0.001821	0.001829	0.001963	0.001919	0.002345	0.002326	0.002051	0.002472	0.001904	0.001799	0.001836	0.001922	0.002385	China oil refineries	6.32E-05
M00006: Pentose_phosphate_pathway,_oxidative_phase,_glucose_6P_=>_ribulose_5P	0.00196	0.001628	0.001346	0.001342	0.00147	0.001911	0.001903	0.001971	0.002002	0.00189	0.001837	0.001965	0.002347	0.002281	0.001704	0.001882	0.001913	0.001864	0.002128	0.001814	0.001862	0.001562	0.001903	0.001773	0.001707	0.001557	0.001653	0.001532	0.001782	0.001462	0.001545	0.001493	0.00142	0.001562	0.001494	0.001457	0.001398	0.002256	0.001469	0.001214	0.001445	0.001999	0.001429	0.001457	0.00139	0.002339	0.001125	0.000984	0.00113	0.001189	0.001001	0.000916	0.001016	0.001033	0.000971	0.000823	0.000974	0.001093	0.00122	0.00121	0.000914	0.000855	0.000749	0.000888	0.001061	Arctic	1.82E-06
M00007: Pentose_phosphate_pathway,_non-oxidative_phase,_fructose_6P_=>_ribose_5P	0.002262	0.002023	0.002571	0.002213	0.002207	0.002116	0.002181	0.002195	0.00227	0.002327	0.00229	0.002181	0.00216	0.002109	0.002247	0.002192	0.002158	0.002425	0.002273	0.002512	0.002463	0.002445	0.002506	0.002485	0.002507	0.002708	0.002594	0.002628	0.002337	0.002307	0.002382	0.002248	0.00224	0.002181	0.002225	0.002058	0.001947	0.002259	0.002023	0.002032	0.002101	0.002285	0.001988	0.002105	0.002144	0.002954	0.001775	0.002219	0.002174	0.001919	0.002249	0.002262	0.002248	0.002211	0.002055	0.001832	0.00287	0.002518	0.002154	0.002609	0.001991	0.001843	0.001749	0.002132	0.002672		

KEGG_Module	I1	I2 – OSTPd2	OSTPd3	OSTPd4	One-class test (strict, p < 0.01)	p value
M00008: Entner-Doudoroff_pathway,_glucose-6P_=>_glyceraldehyde-3P_+_pyruvate	0.001898	[illegible]	0.000995	0.000859	Taiga permafrost layer	1.76E-07
M00009: Citrate_cycle_(TCA_cycle,_Krebs_cycle)	0.001677	[illegible]	0.001748	0.001971	Mangrove	0.002283
M00010: Citrate_cycle,_first_carbon_oxidation,_oxaloacetate_=>_2-oxoglutarate	0.002663	[illegible]	0.002589	0.00229	Oil sands core	5.33E-05
M00011: Citrate_cycle,_second_carbon_oxidation,_2-oxoglutarate_=>_oxaloacetate	0.001419	[illegible]	0.001562	0.001914	Mangrove	0.004024
M00012: Glyoxylate_cycle	0.001883	[illegible]	0.001913	0.001566	Oil sands core	0.000122
M00013: Malonate_semialdehyde_pathway,_propanoyl-CoA_=>_acetyl-CoA	0.001054	[illegible]	0.003047	0.001661	Oil sands core	0.000115
M00014: Glucuronate_pathway_(uronate_pathway)	0.000758	[illegible]	0.000359	0.000567	China oil refineries	1.57E-05
M00015: Proline_biosynthesis,_glutamate_=>_proline	0.001506	[illegible]	0.001775	0.002043		
M00016: Lysine_biosynthesis,_succinyl-DAP_pathway,_aspartate_=>_lysine	0.002198	[illegible]	0.00208	0.002279		
M00017: Methionine_biosynthesis,_apartate_=>_homoserine_=>_methionine	0.001939	[illegible]	0.001854	0.001871	Taiga bottom active layer	8.57E-05
M00018: Threonine_biosynthesis,_aspartate_=>_homoserine_=>_threonine	0.002016	[illegible]	0.001867	0.002002	Taiga bottom active layer	6.71E-07
M00019: Valine/isoleucine_biosynthesis,_pyruvate_=>_valine_/_2-oxobutanoate_=>_isoleucine	0.002962	[illegible]	0.003103	0.002813	Taiga permafrost layer	7.39E-08
M00020: Serine_biosynthesis,_glycerate-3P_=>_serine	0.002121	[illegible]	0.002246	0.001954		
M00021: Cysteine_biosynthesis,_serine_=>_cysteine	0.002439	[illegible]	0.002095	0.001962	Taiga bottom active layer	0.000132
M00022: Shikimate_pathway,_phosphoenolpyruvate_+_erythrose-4P_=>_chorismate	0.001906	[illegible]	0.001854	0.001974	Mangrove	0.001601
M00023: Tryptophan_biosynthesis,_chorismate_=>_tryptophan	0.001853	[illegible]	0.001648	0.001897	Mangrove	5.67E-05
M00024: Phenylalanine_biosynthesis,_chorismate_=>_phenylalanine	0.000523	[illegible]	0.000487	0.000455	Taiga permafrost layer	0.001381
M00025: Tyrosine_biosynthesis,_chorismate_=>_tyrosine	0.000567	[illegible]	0.000568	0.000543	Oil sands tailings pond upper	0.000178
M00026: Histidine_biosynthesis,_PRPP_=>_histidine	0.001866	[illegible]	0.00169	0.001798	Taiga permafrost layer	2.07E-05
M00027: GABA_(gamma-Aminobutyrate)_shunt	0.000976	[illegible]	0.000981	0.000671	Oil sands core	1.07E-06
M00028: Ornithine_biosynthesis,_glutamate_=>_ornithine	0.001636	[illegible]	0.001297	0.001394	Marine sediments	0.000556
M00029: Urea_cycle	0.001708	[illegible]	0.001461	0.002008	Taiga permafrost layer	3.97E-06
M00030: Lysine_biosynthesis,_AAA_pathway,_2-oxoglutarate_=>_2-aminoadipate_=>_lysine	5.40E-05	[illegible]	0	0	Marine sediments	9.13E-06
M00031: Lysine_biosynthesis,_mediated_by_LysW,_2-aminoadipate_=>_lysine	0.00029	[illegible]	4.89E-05	0.000206	China oil refineries	1.60E-06
M00032: Lysine_degradation,_lysine_=>_saccharopine_=>_acetoacetyl-CoA	0.00098	[illegible]	0.001049	0.000774	Taiga permafrost layer	7.52E-05
M00033: Ectoine_biosynthesis,_aspartate_=>_ectoine	0.0016	[illegible]	0.001423	0.001623		
M00034: Methionine_salvage_pathway	0.000762	[illegible]	0.000794	0.000985	Oil sands tailings pond upper	3.27E-07
M00035: Methionine_degradation	0.001388	[illegible]	0.001206	0.001489	Mangrove	9.36E-05
M00036: Leucine_degradation,_leucine_=>_acetoacetate_+_acetyl-CoA	0.001775	[illegible]	0.002951	0.00214	Oil sands core	6.05E-05
M00038: Tryptophan_metabolism,_tryptophan_=>_kynurenine_=>_2-aminomuconate	0.000555	[illegible]	0.000445	0.000235		
M00039: Monolignol_biosynthesis,_phenylalanine_/_tyrosine__=>_monolignol	0	[illegible]	0	0		
M00040: Tyrosine_biosynthesis,_prephanate_=>_pretyrosine_=>_tyrosine	0.000309	[illegible]	0.000426	0.000207	Taiga permafrost layer	0.000564
M00042: Catecholamine_biosynthesis,_tyrosine_=>_dopamine_=>_noradrenaline_=>_adrenaline	0.000285	[illegible]	2.64E-05	8.50E-05	India oil refineries	0.000817
M00044: Tyrosine_degradation,_tyrosine_=>_homogentisate	0.001281	[illegible]	0.001114	0.000629	Taiga permafrost layer	0.00182
M00045: Histidine_degradation,_histidine_=>_N-formiminoglutamate_=>_glutamate	0.000902	[illegible]	0.000807	0.001182	Oil sands core	2.38E-06
M00046: Pyrimidine_degradation,_uracil_=>_beta-alanine,_thymine_=>_3-aminoisobutanoate	4.14E-05	[illegible]	6.09E-05	4.18E-05	Oil sands core	0.000326
M00047: Creatine_pathway	2.67E-06	[illegible]	0	1.10E-05		
M00048: Inosine_monophosphate_biosynthesis,_PRPP_+_glutamine_=>_IMP	0.002111	[illegible]	0.001879	0.002097	Taiga permafrost layer	3.40E-05
M00049: Adenine_ribonucleotide_biosynthesis,_IMP_=>_ADP,ATP	0.001908	[illegible]	0.001857	0.00213	Taiga bottom active layer	5.33E-05
M00050: Guanine_ribonucleotide_biosynthesis_IMP_=>_GDP,GTP	0.002176	[illegible]	0.002031	0.002229	Arctic	0.000306
M00051: Uridine_monophosphate_biosynthesis,_glutamine_(+_PRPP)_=>_UMP	0.001797	[illegible]	0.001767	0.001874	Mangrove	0.001073
M00052: Pyrimidine_ribonucleotide_biosynthesis,_UMP_=>_UDP/UTP,CDP/CTP	0.001398	[illegible]	0.001198	0.001605	Mangrove	0.00085
M00053: Pyrimidine_deoxyribonuleotide_biosynthesis,_CDP/CTP_=>_dCDP/dCTP,dTDP/dTTP	0.001963	[illegible]	0.001782	0.001742	Taiga permafrost layer	8.21E-05
M00055: N-glycan_precursor_biosynthesis	1.91E-07	[illegible]	2.76E-07	1.99E-06		
M00060: Lipopolysaccharide_biosynthesis,_KDO2-lipid_A	0.001874	[illegible]	0.001944	0.001799	Marine sediments	2.86E-06
M00061: D-Glucuronate_degradation	0.000999	[illegible]	0.000449	0.00033	Taiga upper active layer	3.13E-06
M00063: CMP-KDO_biosynthesis	0.001745	[illegible]	0.001705	0.001567	India oil refineries	0.000598
M00064: ADP-L-glycero-D-manno-heptose_biosynthesis	0.002213	[illegible]	0.001255	0.001304	India oil refineries	0.000253
M00066: Lactosylceramide_biosynthesis	0.002196	[illegible]	0.000145	6.18E-05	India oil refineries	4.10E-06
M00072: N-glycosylation_by_oligosaccharyltransferase	1.67E-06	[illegible]	8.21E-06	5.38E-05		
M00073: N-glycan_precursor_trimming	0	[illegible]	0	0		
M00075: N-glycan_biosynthesis,_complex_type	1.14E-05	[illegible]	0	0		
M00076: Dermatan_sulfate_degradation	0	[illegible]	1.93E-06	0	China oil refineries	2.89E-06
M00077: Chondroitin_sulfate_degradation	5.88E-05	[illegible]	7.34E-05	4.08E-05	Taiga bottom active layer	4.11E-06
M00078: Heparan_sulfate_degradation	0.00017	[illegible]	3.09E-05	1.59E-05	Taiga bottom active layer	8.77E-06
M00080: Lipopolysaccharide_biosynthesis,_inner_core_=>_outer_core_=>_O-antigen	0.000659	[illegible]	0.000705	0.000925	Mangrove	2.82E-06
M00081: Pectin_degradation	0.000779	[illegible]	0.000146	8.77E-05	Taiga bottom active layer	8.76E-06
M00082: Fatty_acid_biosynthesis,_initiation	0.001786	[illegible]	0.001655	0.001819	Taiga permafrost layer	2.70E-06
M00083: Fatty_acid_biosynthesis,_elongation	0.002593	[illegible]	0.002452	0.002462	Taiga permafrost layer	1.97E-05
M00085: Fatty_acid_biosynthesis,_elongation,_mitochondria	0	[illegible]	7.08E-06	0		
M00086: beta-Oxidation,_acyl-CoA_synthesis	0.004257	[illegible]	0.006117	0.006087	Arctic	5.88E-06
M00087: beta-Oxidation	0.000923	[illegible]	0.001712	0.001188	Taiga permafrost layer	0.000485
M00088: Ketone_body_biosynthesis,_acetyl-CoA_=>_acetoacetate/3-hydroxybutyrate/acetone	0.002315	[illegible]	0.004772	0.003216	Oil sands core	0.000201
M00089: Triacylglycerol_biosynthesis	0.000735	[illegible]	0.000612	0.00077	China oil refineries	6.86E-06
M00090: Phosphatidylcholine_(PC)_biosynthesis,_choline_=>_PC	0	[illegible]	0	0		
M00091: Phosphatidylcholine_(PC)_biosynthesis,_PE_=>_PC	0.000168	[illegible]	6.38E-05	4.08E-05	Taiga upper active layer	0.000129
M00092: Phosphatidylethanolamine_(PE)_biosynthesis,_ethanolamine_=>_PE	0	[illegible]	0	0		
M00093: Phosphatidylethanolamine_(PE)_biosynthesis,_PA_=>_PS_=>_PE	0.002167	[illegible]	0.002129	0.002144	Oil sands tailings pond upper	1.16E-06
M00095: C5_isoprenoid_biosynthesis,_mevalonate_pathway	0.001257	[illegible]	0.002747	0.002076	Taiga permafrost layer	0.000268
M00096: C5_isoprenoid_biosynthesis,_non-mevalonate_pathway	0.001844	[illegible]	0.001707	0.002053		
M00097: beta-Carotene_biosynthesis,_GGAP_=>_beta-carotene	0.000996	[illegible]	0.000529	0.000337	Taiga permafrost layer	3.61E-06
M00098: Acylglycerol_degradation	2.29E-06	[illegible]	5.77E-05	4.38E-05	Oil sands core	3.43E-06
M00099: Sphingosine_biosynthesis	2.34E-06	[illegible]	5.80E-06	0		
M00101: Cholesterol_biosynthesis,_squalene_2,3-epoxide_=>_cholesterol	8.91E-07	[illegible]	5.80E-06	3.32E-07		
M00102: Ergocalciferol_biosynthesis	0	[illegible]	2.58E-06	0		
M00104: Bile_acid_biosynthesis,_cholesterol_=>_cholate	9.02E-05	[illegible]	0.000121	4.58E-05	Taiga permafrost layer	3.51E-05
M00107: Steroid_hormone_biosynthesis,_cholesterol_=>_prognenolone_=>_progesterone	2.07E-05	[illegible]	0.00026	7.87E-05	Oil sands tailings pond deep	3.01E-06
M00112: Tocopherol_biosynthesis	1.02E-05	[illegible]	1.35E-05	4.38E-05	China oil refineries	5.37E-07
M00114: Ascorbate_biosynthesis,_plants,_glucose-6P_=>_ascorbate	0.000684	[illegible]	0.00072	0.00091	Taiga permafrost layer	0.000467
M00115: NAD_biosynthesis,_aspartate_=>_NAD	0.00185	[illegible]	0.001587	0.001894	Mangrove	1.34E-05
M00116: Menaquinone_biosynthesis,_chorismate_=>_menaquinone	0.000637	[illegible]	0.000578	0.00078	China oil refineries	0.000276
M00117: Ubiquinone_biosynthesis,_prokaryotes,_chorismate_=>_ubiquinone	0.001488	[illegible]	0.001618	0.001424	Oil sands tailings pond deep	0.009116
M00118: Glutathione_biosynthesis,_glutamate_=>_glutathione	0.000952	[illegible]	0.001645	0.000734	Oil sands core	0.000277
M00119: Pantothenate_biosynthesis,_valine/L-aspartate_=>_pantothenate	0.001995	[illegible]	0.001917	0.002356	Taiga permafrost layer	5.90E-06
M00120: Coenzyme_A_biosynthesis,_pantothenate_=>_CoA	0.001253	[illegible]	0.0012	0.001458	Mangrove	0.002765
M00121: Heme_biosynthesis,_glutamate_=>_protoheme/siroheme	0.002029	[illegible]	0.001867	0.001752	Taiga permafrost layer	6.42E-05
M00122: Cobalamin_biosynthesis,_cobinamide_=>_cobalamin	0.000921	[illegible]	0.001512	0.001684	Oil sands tailings pond upper	1.21E-06
M00123: Biotin_biosynthesis,_pimeloyl-ACP/CoA_=>_biotin	0.000913	[illegible]	0.001497	0.001886	Marine sediments	0.000617
M00124: Pyridoxal_biosynthesis,_erythrose-4P_=>_pyridoxal-5P	0.001108	[illegible]	0.001718	0.001486	Oil sands core	0.001097
M00125: Riboflavin_biosynthesis,_GTP_=>_riboflavin/FMN/FAD	0.001743	[illegible]	0.001632	0.001778		

Table S4. I

KEGG_Module	Habitat																																																																One-class test (strict, p < 0.01)	p value
	India oil refineries				Arctic												China oil refineries									Mangrove			Marine sediments							Taiga upper active layer				Taiga bottom active layer				Taiga permafrost layer				Oil sands core							Oil sands tailings pond upper				Oil sands tailings pond median			Oil sands tailings pond deep					
	I1	I2	I3	I4	A1	A10	A11	A12	A2	A3	A4	A5	A6	A7	A8	A9	C1	C2	C3	C4	C5	C6	C7	C8	C9	M1	M2	M3	DWH1	DWH2	DWH3	DWH4	DWH5	DWH6	DWH7	Tu1	Tu2	Tu3	Tu4	Tb1	Tb2	Tb3	Tb4	Tp1	Tp2	Tp3	Tp4	OSC1	OSC12	OSC3	OSC4	OSC5	OSC7	OSC9	OSTPu1	OSTPu2	OSTPu4	OSTPu3	OSTPm2	OSTPm6	OSTPm4	OSTPd1	OSTPd2	OSTPd3	OSTPd4		
M00126: Tetrahydrofolate_biosynthesis,_GTP_=>_THF	0.001398	0.001378	0.001763	0.001675	0.001486	0.001558	0.001556	0.001409	0.00126	0.001423	0.001349	0.001374	0.001394	0.001463	0.001337	0.001354	0.001291	0.001503	0.001392	0.001571	0.001528	0.001598	0.001497	0.001557	0.00154	0.00161	0.001594	0.001676	0.001539	0.001499	0.001462	0.001529	0.001472	0.001407	0.001514	0.001679	0.001792	0.0015	0.001635	0.001643	0.001811	0.001501	0.00181	0.001776	0.001977	0.001457	0.001884	0.001483	0.001456	0.001304	0.001515	0.001494	0.001476	0.001499	0.001449	0.001449	0.001319	0.001339	0.00137	0.001459	0.001447	0.001448	0.001457	0.001488	0.001449	Taiga permafrost layer	0.006159
M00127: Thiamine_biosynthesis,_AIR_=>_thiamine-P/thiamine-2P	0.00208	0.001954	0.001782	0.001889	0.001818	0.00175	0.001739	0.001842	0.002015	0.001923	0.001939	0.001976	0.001742	0.001765	0.002027	0.002013	0.001585	0.001863	0.001769	0.001924	0.00197	0.001923	0.001903	0.001842	0.00189	0.001681	0.001718	0.001697	0.001824	0.001761	0.001846	0.001734	0.001754	0.001591	0.001706	0.002065	0.002269	0.0016	0.002318	0.002045	0.002343	0.00203	0.002291	0.002266	0.002477	0.001908	0.002084	0.001693	0.001673	0.001472	0.00174	0.001765	0.001716	0.001737	0.001912	0.001844	0.001961	0.001945	0.001888	0.001746	0.001929	0.001856	0.001849	0.001829	0.001861	Taiga bottom active layer	0.001388
M00128: Ubiquinone_biosynthesis,_eukaryotes,_4-hydroxybenzoate_=>_ubiquinone	0.000274	0.000289	0.000233	0.000364	0.00024	0.000245	0.00019	0.000167	0.000119	0.000103	0.000126	0.000181	5.36E-05	0.000128	0.000159	0.000222	7.85E-05	8.53E-05	6.42E-05	6.84E-05	7.23E-05	0.000255	7.02E-05	6.20E-05	0.000152	0.000136	0.000177	0.000138	0.000187	0.000242	0.000199	0.000271	0.000287	0.000146	0.000284	0.000474	0.000725	6.75E-05	0.00027	0.000534	0.000582	0.000313	0.000687	0.000534	0.000634	6.01E-06	0.000902	0.000391	0.000375	0.000349	0.000425	0.000438	0.000399	0.0004	0.000381	0.000432	0.000157	0.000248	0.000295	0.000108	0.000401	0.000425	0.000468	0.000399	0.000179	Taiga permafrost layer	0.000142
M00129: Ascorbate_biosynthesis,_animals,_glucose-1P_=>_ascorbate	0.001321	0.001422	0.001223	0.001218	0.001045	0.001191	0.001073	0.00112	0.001169	[illegible]	[illegible]	[illegible]	[illegible]	[illegible]	[illegible]	[illegible]	[illegible]	[illegible]	[illegible]	[illegible]	[illegible]	[illegible]	[illegible]	[illegible]	[illegible]	[illegible]	[illegible]	[illegible]	[illegible]	[illegible]	[illegible]	[illegible]	[illegible]	[illegible]	[illegible]	[illegible]	[illegible]	[illegible]	[illegible]	[illegible]	[illegible]	[illegible]	[illegible]	[illegible]	[illegible]	[illegible]	[illegible]	[illegible]	[illegible]	[illegible]	[illegible]	[illegible]	[illegible]	[illegible]	[illegible]	[illegible]	[illegible]	[illegible]	[illegible]	[illegible]	[illegible]	[illegible]	[illegible]	[illegible]	[illegible]	Taiga bottom active layer	[illegible]
M00130: Inositol_phosphate_metabolism,_PI=>_PIP2_=>_Ins(1,4,5)P3_=>_Ins(1,3,4,5)P4	0	0	0	0	0	0	0	0	0	[illegible]	[illegible]	[illegible]	[illegible]	[illegible]	[illegible]	[illegible]	[illegible]	[illegible]	[illegible]	[illegible]	[illegible]	[illegible]	[illegible]	[illegible]	[illegible]	[illegible]	[illegible]	[illegible]	[illegible]	[illegible]	[illegible]	[illegible]	[illegible]	[illegible]	[illegible]	[illegible]	[illegible]	[illegible]	[illegible]	[illegible]	[illegible]	[illegible]	[illegible]	[illegible]	[illegible]	[illegible]	[illegible]	[illegible]	[illegible]	[illegible]	[illegible]	[illegible]	[illegible]	[illegible]	[illegible]	[illegible]	[illegible]	[illegible]	[illegible]	[illegible]	[illegible]	[illegible]	[illegible]	[illegible]	[illegible]	[illegible]	[illegible]
M00131: Inositol_phosphate_metabolism,_Ins(1,3,4,5)P4_=>_Ins(1,3,4)P3_=>_myo-inositol	0.000646	0.0006	0.000661	0.000675	0.000647	0.0007	0.000768	0.000801	0.000686	[illegible]	[illegible]	[illegible]	[illegible]	[illegible]	[illegible]	[illegible]	[illegible]	[illegible]	[illegible]	[illegible]	[illegible]	[illegible]	[illegible]	[illegible]	[illegible]	[illegible]	[illegible]	[illegible]	[illegible]	[illegible]	[illegible]	[illegible]	[illegible]	[illegible]	[illegible]	[illegible]	[illegible]	[illegible]	[illegible]	[illegible]	[illegible]	[illegible]	[illegible]	[illegible]	[illegible]	[illegible]	[illegible]	[illegible]	[illegible]	[illegible]	[illegible]	[illegible]	[illegible]	[illegible]	[illegible]	[illegible]	[illegible]	[illegible]	[illegible]	[illegible]	[illegible]	[illegible]	[illegible]	[illegible]	[illegible]	[illegible]	[illegible]
M00133: Polyamine_biosynthesis,_arginine_=>_agmatine_=>_putrescine_=>_spermidine	0.000696	0.000925	0.001134	0.001122	0.001229	0.000956	0.000999	0.001141	0.000944	[illegible]	[illegible]	[illegible]	[illegible]	[illegible]	[illegible]	[illegible]	[illegible]	[illegible]	[illegible]	[illegible]	[illegible]	[illegible]	[illegible]	[illegible]	[illegible]	[illegible]	[illegible]	[illegible]	[illegible]	[illegible]	[illegible]	[illegible]	[illegible]	[illegible]	[illegible]	[illegible]	[illegible]	[illegible]	[illegible]	[illegible]	[illegible]	[illegible]	[illegible]	[illegible]	[illegible]	[illegible]	[illegible]	[illegible]	[illegible]	[illegible]	[illegible]	[illegible]	[illegible]	[illegible]	[illegible]	[illegible]	[illegible]	[illegible]	[illegible]	[illegible]	[illegible]	[illegible]	[illegible]	[illegible]	[illegible]	[illegible]	[illegible]
M00134: Polyamine_biosynthesis,_arginine_=>_ornithine_=>_putrescine	0.001338	0.001222	0.001171	0.001197	0.00117	0.001148	0.001082	0.00108	0.001317	[illegible]	[illegible]	[illegible]	[illegible]	[illegible]	[illegible]	[illegible]	[illegible]	[illegible]	[illegible]	[illegible]	[illegible]	[illegible]	[illegible]	[illegible]	[illegible]	[illegible]	[illegible]	[illegible]	[illegible]	[illegible]	[illegible]	[illegible]	[illegible]	[illegible]	[illegible]	[illegible]	[illegible]	[illegible]	[illegible]	[illegible]	[illegible]	[illegible]	[illegible]	[illegible]	[illegible]	[illegible]	[illegible]	[illegible]	[illegible]	[illegible]	[illegible]	[illegible]	[illegible]	[illegible]	[illegible]	[illegible]	[illegible]	[illegible]	[illegible]	[illegible]	[illegible]	[illegible]	[illegible]	[illegible]	[illegible]	[illegible]	[illegible]
M00135: GABA_biosynthesis,_eukaryotes,_putrescine_=>_GABA	0.001737	0.001135	0.001214	0.001023	0.001212	0.001675	0.001494	0.001527	0.001787	[illegible]	[illegible]	[illegible]	[illegible]	[illegible]	[illegible]	[illegible]	[illegible]	[illegible]	[illegible]	[illegible]	[illegible]	[illegible]	[illegible]	[illegible]	[illegible]	[illegible]	[illegible]	[illegible]	[illegible]	[illegible]	[illegible]	[illegible]	[illegible]	[illegible]	[illegible]	[illegible]	[illegible]	[illegible]	[illegible]	[illegible]	[illegible]	[illegible]	[illegible]	[illegible]	[illegible]	[illegible]	[illegible]	[illegible]	[illegible]	[illegible]	[illegible]	[illegible]	[illegible]	[illegible]	[illegible]	[illegible]	[illegible]	[illegible]	[illegible]	[illegible]	[illegible]	[illegible]	[illegible]	[illegible]	[illegible]	[illegible]	[illegible]
M00136: GABA_biosynthesis,_prokaryotes,_putrescine_=>_GABA	0.000478	0.000345	0.000149	0.000395	0.000442	0.000409	0.000418	0.000235	0.000187	[illegible]	[illegible]	[illegible]	[illegible]	[illegible]	[illegible]	[illegible]	[illegible]	[illegible]	[illegible]	[illegible]	[illegible]	[illegible]	[illegible]	[illegible]	[illegible]	[illegible]	[illegible]	[illegible]	[illegible]	[illegible]	[illegible]	[illegible]	[illegible]	[illegible]	[illegible]	[illegible]	[illegible]	[illegible]	[illegible]	[illegible]	[illegible]	[illegible]	[illegible]	[illegible]	[illegible]	[illegible]	[illegible]	[illegible]	[illegible]	[illegible]	[illegible]	[illegible]	[illegible]	[illegible]	[illegible]	[illegible]	[illegible]	[illegible]	[illegible]	[illegible]	[illegible]	[illegible]	[illegible]	[illegible]	[illegible]	[illegible]	[illegible]
[illegible]																																																																			

Table S4. II

KEGG_Module	I1	OSTPd3	OSTPd4	One-class test (strict, p < 0.01)	p value
M00242: Zinc_transport_system	0.000347	0.000795	0.001673	Mangrove	0.000771
M00243: Manganese/iron_transport_system	0.00013	4.06E-05	0.000149	China oil refineries	0.000103
M00244: Putative_zinc/manganese_transport_system	0.000331	0.000342	0.000239	Arctic	2.25E-05
M00245: Cobalt/nickel_transport_system	0.000152	0.000561	0.001503	Oil sands tailings pond upper	0.001534
M00246: Nickel_transport_system	0.000148	0.000543	0.001443	Oil sands tailings pond upper	0.001299
M00247: Putative_ABC_transport_system	7.81E-05	0.001261	0.001083	Oil sands core	3.17E-08
M00249: Capsular_polysaccharide_transport_system	0.000239	0.000135	6.58E-05	Taiga permafrost layer	6.14E-05
M00250: Lipopolysaccharide_transport_system	0.000851	0.001194	0.00159	Marine sediments	1.54E-05
M00251: Teichoic_acid_transport_system	6.68E-06	1.55E-05	5.98E-06	Taiga upper active layer	0.000132
M00252: Lipooligosaccharide_transport_system	0.000274	0.000788	0.000293	Oil sands tailings pond upper	6.53E-07
M00253: Sodium_transport_system	5.08E-05	4.25E-05	2.39E-05	China oil refineries	6.34E-06
M00254: ABC-2_type_transport_system	0.009622	0.006816	0.010086	China oil refineries	9.27E-06
M00255: Lipoprotein-releasing_system	0.002391	0.001891	0.001867	Mangrove	0.001304
M00256: Cell_division_transport_system	0.000995	0.000912	0.00153	Mangrove	0.00043
M00257: Hemin_transport_system	0	3.86E-06	0		
M00258: Putative_ABC_transport_system	0.014746	0.006198	0.007243	India oil refineries	4.34E-05
M00259: Heme_transport_system	0.001699	0.000962	0.000811	Taiga bottom active layer	4.85E-06
M00260: DNA_polymerase_III_complex,_bacteria	0.001964	0.002138	0.00238	Mangrove	0.000479
M00265: PTS_system,_glucose-specific_II_component	0	2.80E-05	3.19E-05	Oil sands tailings pond upper	3.25E-06
M00266: PTS_system,_maltose_and_glucose-specific_II_component	0	2.61E-05	2.99E-05	Oil sands tailings pond upper	1.07E-05
M00267: PTS_system,_N-acetylglucosamine-specific_II_component	1.20E-05	5.47E-05	4.28E-05	Taiga bottom active layer	3.82E-07
M00268: PTS_system,_arbutin-like_II_component	0	2.51E-05	3.29E-05		
M00269: PTS_system,_sucrose-specific_II_component	1.34E-06	1.64E-05	2.39E-05	Oil sands core	0.000147
M00270: PTS_system,_trehalose-specific_II_component	0	2.70E-05	3.39E-05		
M00271: PTS_system,_beta-glucosides-specific_II_component	4.41E-05	1.35E-05	2.49E-05	Arctic	6.70E-06
M00272: PTS_system,_arbutin-,_cellobiose-,_and_salicin-specific_II_component	0	2.51E-05	2.89E-05		
M00273: PTS_system,_fructose-specific_II_component	8.62E-05	7.73E-05	0.000179	Mangrove	8.34E-06
M00274: PTS_system,_mannitol-specific_II_component	8.88E-05	1.55E-05	3.59E-05	Arctic	2.77E-06
M00275: PTS_system,_cellobiose-specific_II_component	4.54E-05	5.76E-05	0.000197	Oil sands tailings pond upper	2.36E-06
M00276: PTS_system,_mannose-specific_II_component	0.000151	0.000194	0.000451	Taiga permafrost layer	0.004691
M00277: PTS_system,_N-acetylgalactosamine-specific_II_component	0	9.66E-07	0		
M00279: PTS_system,_galactitol-specific_II_component	0	0	5.18E-05	Oil sands tailings pond upper	1.35E-06
M00280: PTS_system,_glucitol/sorbitol-specific_II_component	8.55E-05	8.11E-05	0.000281	Oil sands tailings pond median	7.18E-06
M00281: PTS_system,_lactose-specific_II_component	0	0	1.99E-06		
M00282: PTS_system,_D-glucosamine-specific_II_component	0	5.80E-06	7.97E-06		
M00283: PTS_system,_ascorbate-specific_II_component	0.000305	0.000425	0.00035	Oil sands tailings pond deep	2.31E-05
M00287: PTS_system,_galactosamine-specific_II_component	0	0	4.98E-06		
M00288: RPA_complex	2.67E-06	7.73E-06	3.99E-06		
M00290: Holo-TFIIH_complex	2.91E-05	9.47E-05	3.35E-05	Arctic	4.54E-06
M00295: BRCA1-associated_genome_surveillance_complex_(BASC)	7.63E-07	2.21E-06	1.14E-06		
M00296: BER_complex	0	0	0		
M00298: Multidrug/hemolysin_transport_system	0	1.93E-06	1.99E-06		
M00299: Spermidine/putrescine_transport_system	0.000288	0.000377	0.001108	Marine sediments	0.000183
M00300: Putrescine_transport_system	0.000454	0.00016	0.000162	Marine sediments	0.000139
M00301: Mannopine_transport_system	0	0	2.39E-05		
M00302: 2-Aminoethylphosphonate_transport_system	1.20E-05	9.66E-07	0		
M00304: PTS_system,_fructose-specific_II_component	0	0	0		
M00305: PTS_system,_2-O-A-mannosyl-D-glycerate-specific_II_component	0	0	0		
M00306: PTS_system,_fructose-specific_II-like_component	0	1.16E-05	9.96E-06		
M00307: Pyruvate_oxidation,_pyruvate_=>_acetyl-CoA	0.001996	0.002208	0.002231	Taiga permafrost layer	7.89E-05
M00308: Semi-phosphorylative_Entner-Doudoroff_pathway,_gluconate_=>_glycerate-3P	0.001624	0.001378	0.001685	Marine sediments	1.26E-05
M00309: Non-phosphorylative_Entner-Doudoroff_pathway,_gluconate/galactonate_=>_glycerate	4.68E-05	0.000325	0.000766	Oil sands tailings pond upper	1.85E-07
M00314: Bacitracin_transport_system	0	0	0		
M00315: Uncharacterized_ABC_transport_system	0	1.93E-06	0		
M00316: Manganese_transport_system	1.34E-06	7.73E-06	0	Taiga upper active layer	4.04E-05
M00317: Manganese/iron_transport_system	2.54E-05	0.000184	8.57E-05	Oil sands core	7.04E-06
M00318: Iron/zinc/copper_transport_system	0	4.83E-06	3.99E-06		
M00319: Manganese/zinc/iron_transport_system	0.000152	7.63E-05	0.000141	China oil refineries	1.76E-05
M00320: Lipopolysaccharide_export_system	0.001455	0.001758	0.001718	Taiga bottom active layer	0.000202
M00321: Bicarbonate_transport_system	1.34E-06	0	0		
M00322: Neutral_amino_acid_transport_system	4.01E-06	0	2.66E-06	China oil refineries	1.09E-05
M00323: Urea_transport_system	0.000409	0.000433	0.000166	Marine sediments	0.001007
M00324: Dipeptide_transport_system	2.85E-05	0.000114	0.000104	Oil sands core	0.000898
M00325: alpha-Hemolysin/cyclolysin_transport_system	0.000894	0.001005	0.000638	Oil sands tailings pond deep	0.000623
M00326: RTX_toxin_transport_system	0	0	0		
M00327: S-Layer_protein_transport_system	0	0	0		
M00328: Hemophore/metalloprotease_transport_system	3.81E-05	9.08E-05	6.38E-05	Oil sands core	1.08E-05
M00329: Multiple_protein_transport_system	5.34E-06	0	0		
M00330: Adhesin_protein_transport_system	0.00014	0.000303	0.000106	Oil sands tailings pond upper	1.30E-05
M00331: Type_II_general_secretion_system	0.001206	0.00148	0.001408	Oil sands tailings pond upper	3.62E-07
M00332: Type_III_secretion_system	2.00E-05	9.19E-05	4.05E-05	Oil sands core	8.83E-05
M00333: Type_IV_secretion_system	0.002439	0.001492	0.000593	Taiga bottom active layer	0.000104
M00334: Type_VI_secretion_system	0.000566	0.000454	0.000286	Marine sediments	4.59E-07
M00335: Sec_(secretion)_system	0.001892	0.001806	0.002212		
M00336: Twin-arginine_translocation_(Tat)_system	0.002016	0.001893	0.002367	China oil refineries	0.000338
M00338: Cysteine_biosynthesis,_homocysteine_+_serine_=>_cysteine	0.000578	0.000127	0.000174	Mangrove	9.13E-07
M00339: RaxAB-RaxC_type_I_secretion_system	0.000943	0	0	India oil refineries	0.000843
M00342: Bacterial_proteasome	1.34E-05	2.32E-05	0.000207	China oil refineries	0.000326
M00343: Archaeal_proteasome	1.00E-05	2.03E-05	0.000204	China oil refineries	0.000403
M00344: Formaldehyde_assimilation,_xylulose_monophosphate_pathway	0.000921	0.001491	0.001287	Oil sands tailings pond upper	0.000114
M00345: Formaldehyde_assimilation,_ribulose_monophosphate_pathway	0.000745	0.00072	0.001022	Oil sands tailings pond upper	0.000162
M00346: Formaldehyde_assimilation,_serine_pathway	0.001556	0.001461	0.001538	India oil refineries	3.66E-06
M00348: Glutathione_transport_system	9.35E-06	9.18E-05	5.58E-05	Oil sands core	0.001918
M00349: Microcin_C_transport_system	0.00018	0.001152	0.000643	Oil sands core	6.53E-05
M00350: Capsaicin_biosynthesis,_L-Phenylalanine_=>_Capsaicin	2.23E-06	6.44E-06	6.64E-07		
M00355: Spliceosome,_35S_U5-snRNP	1.42E-06	1.21E-07	0		
M00356: Methanogenesis,_methanol_=>_methane	0	0	0.000988	Oil sands tailings pond upper	0.008015
M00357: Methanogenesis,_acetate_=>_methane	0	0	0		
M00358: Coenzyme_M_biosynthesis	6.86E-05	6.96E-05	0.000155	Arctic	2.11E-06
M00359: Aminoacyl-tRNA_biosynthesis,_eukaryotes	0.002076	0.001866	0.002336	Mangrove	0.004188
M00360: Aminoacyl-tRNA_biosynthesis,_prokaryotes	0.002015	0.001847	0.002297	Mangrove	0.004593
M00361: Nucleotide_sugar_biosynthesis,_eukaryotes	0.002394	0.002043	0.002379	Arctic	4.27E-06
M00362: Nucleotide_sugar_biosynthesis,_prokaryotes	0.001938	0.001737	0.00211	Arctic	0.000558
M00364: C10-C20_isoprenoid_biosynthesis,_bacteria	0.001287	0.000911	0.001023	Mangrove	0.008298
M00365: C10-C20_isoprenoid_biosynthesis,_archaea	0.000236	0.000149	0.000412	Mangrove	0.001001
M00366: C10-C20_isoprenoid_biosynthesis,_plants	0.000944	0.0003	0.000911	Mangrove	1.70E-05
M00368: Ethylene_biosynthesis,_methionine_=>_ethylene	0	0	0		

Table S4. III

KEGG_Module	Habitat																																																																		
	India oil refineries				Arctic												China oil refineries									Mangrove			Marine sediments							Taiga upper active layer				Taiga bottom active layer				Taiga permafrost layer				Oil sands core							Oil sands tailings pond upper				Oil sands tailings pond median			Oil sands tailings pond deep				One-class test (strict, p < 0.01)	p value
	I1	I2	I3	I4	A1	A10	A11	A12	A2	A3	A4	A5	A6	A7	A8	A9	C1	C2	C3	C4	C5	C6	C7	C8	C9	M1	M2	M3	DWH1	DWH2	DWH3	DWH4	DWH5	DWH6	DWH7	Tu1	Tu2	Tu3	Tu4	Tb1	Tb2	Tb3	Tb4	Tp1	Tp2	Tp3	Tp4	OSC1	OSC12	OSC3	OSC4	OSC5	OSC7	OSC9	OSTPu1	OSTPu2	OSTPu4	OSTPu3	OSTPm2	OSTPm6	OSTPm4	OSTPd1	OSTPd2	OSTPd3	OSTPd4		
M00372: Abscisic_acid_biosynthesis,_beta-carotene_=>_abscisic_acid	4.74E-05	7.66E-06	4.88E-05	3.15E-05	3.87E-05	6.85E-05	9.07E-05	4.05E-05	3.14E-05	6.75E-05	4.79E-05	4.63E-05	2.32E-05	3.83E-05	3.29E-05	3.83E-05	1.71E-05	4.07E-05	4.30E-05	4.60E-05	3.53E-05	2.74E-05	5.03E-05	4.62E-05	3.69E-05	1.22E-05	1.42E-05	1.79E-05	3.25E-05	5.03E-05	2.49E-05	2.92E-05	2.79E-05	2.69E-05	2.93E-05	0.000126	0.000173	2.93E-05	3.63E-05	7.01E-05	0.000289	4.06E-06	0.00019	0.000275	0.000403	1.64E-06	9.09E-05	1.27E-05	1.67E-05	1.06E-05	1.09E-05	1.16E-05	4.46E-06	2.83E-05	9.09E-07	4.72E-07	3.56E-06	9.26E-06	9.67E-07	6.74E-06	3.68E-06	1.39E-06	1.43E-06	1.21E-05	3.99E-06	Taiga permafrost layer	6.66E-05
M00373: Ethylmalonyl_pathway	0.001301	0.001661	0.001812	0.001674	0.001894	0.001806	0.001793	0.001845	0.001531	0.001804	0.001876	0.001652	0.001522	0.001661	0.001761	0.001597	0.001659	0.001316	0.001702	0.001118	0.00113	0.001413	0.001262	0.001071	0.001361	0.001327	0.001359	0.001295	0.001421	0.001625	0.001457	0.001385	0.001417	0.001574	0.001449	0.002411	0.002503	0.001769	0.002674	0.002364	0.002657	0.001556	0.002563	0.002629	0.003028	0.001186	0.002621	0.001915	0.001861	0.001888	0.001843	0.001783	0.001743	0.001835	0.00223	0.002431	0.001506	0.001675	0.001632	0.001643	0.002504	0.002353	0.00256	0.002225	0.001585	Taiga permafrost layer	0.00011
M00374: Dicarboxylate-hydroxybutyrate_cycle	0.001618	0.001791	0.001959	0.001859	0.002036	0.001982	0.002007	0.002019	0.001814	0.002088	0.002088	0.001863	0.001905	0.001959	0.001942	0.001831	0.001828	0.001791	0.001935	0.001724	0.001706	0.001959	0.001765	0.001641	0.00186	0.00177	0.001826	0.00176	0.001988	0.001939	0.002003	0.00191	0.001949	0.001735	0.001883	0.001992	0.002006	0.00206	0.001839	0.001969	0.002171	0.001776	0.001999	0.002158	0.002334	0.001832	0.001962	0.002001	0.001963	0.001863	0.002004	0.001981	0.00196	0.001964	0.002066	0.00208	0.001822	0.001877	0.001829	0.001947	0.002168	0.002012	0.002083	0.002144	0.002078	Taiga permafrost layer	0.004476
M00375: Hydroxypropionate-hydroxybutylate_cycle	0	0	0	0	0.00126	0.001107	0	0	0.001154	0	0	0.001123	0.001106	0	0	0.001108	0	0	0	0	0	0	0	0	0	0	0	0	0	0	0	0	0	0	0	0	0.001291	0	0	0	0	0	0.001312	0	0	0	0	0.001102	0	0	0	0	0	0.001069	0	0	0	0	0	0	0	0	0	0	0		
M00376: 3-Hydroxypropionate_bi-cycle	0.001587	0.001551	0.001772	0.001604	0.001395	0.001339	[illegible]	[illegible]	[illegible]	[illegible]	[illegible]	[illegible]	[illegible]	[illegible]	[illegible]	[illegible]	[illegible]	[illegible]	[illegible]	[illegible]	[illegible]	[illegible]	[illegible]	[illegible]	[illegible]	[illegible]	[illegible]	[illegible]	[illegible]	[illegible]	[illegible]	[illegible]	[illegible]	[illegible]	[illegible]	[illegible]	[illegible]	[illegible]	[illegible]	[illegible]	[illegible]	[illegible]	[illegible]	[illegible]	[illegible]	[illegible]	[illegible]	[illegible]	[illegible]	[illegible]	[illegible]	[illegible]	[illegible]	[illegible]	[illegible]	[illegible]	[illegible]	[illegible]	[illegible]	[illegible]	[illegible]	[illegible]	[illegible]	[illegible]	[illegible]	[illegible]	[illegible]
M00377: Reductive_acetyl-CoA_pathway_(Wood-Ljungdahl_pathway)	0.000756	0.000699	0.000976	0.000826	0.001173	0.000938	[illegible]	[illegible]	[illegible]	[illegible]	[illegible]	[illegible]	[illegible]	[illegible]	[illegible]	[illegible]	[illegible]	[illegible]	[illegible]	[illegible]	[illegible]	[illegible]	[illegible]	[illegible]	[illegible]	[illegible]	[illegible]	[illegible]	[illegible]	[illegible]	[illegible]	[illegible]	[illegible]	[illegible]	[illegible]	[illegible]	[illegible]	[illegible]	[illegible]	[illegible]	[illegible]	[illegible]	[illegible]	[illegible]	[illegible]	[illegible]	[illegible]	[illegible]	[illegible]	[illegible]	[illegible]	[illegible]	[illegible]	[illegible]	[illegible]	[illegible]	[illegible]	[illegible]	[illegible]	[illegible]	[illegible]	[illegible]	[illegible]	[illegible]	[illegible]	[illegible]	[illegible]
M00378: F420_biosynthesis	0.000141	8.08E-05	0.000164	0.000112	0.000338	0.000582	[illegible]	[illegible]	[illegible]	[illegible]	[illegible]	[illegible]	[illegible]	[illegible]	[illegible]	[illegible]	[illegible]	[illegible]	[illegible]	[illegible]	[illegible]	[illegible]	[illegible]	[illegible]	[illegible]	[illegible]	[illegible]	[illegible]	[illegible]	[illegible]	[illegible]	[illegible]	[illegible]	[illegible]	[illegible]	[illegible]	[illegible]	[illegible]	[illegible]	[illegible]	[illegible]	[illegible]	[illegible]	[illegible]	[illegible]	[illegible]	[illegible]	[illegible]	[illegible]	[illegible]	[illegible]	[illegible]	[illegible]	[illegible]	[illegible]	[illegible]	[illegible]	[illegible]	[illegible]	[illegible]	[illegible]	[illegible]	[illegible]	[illegible]	[illegible]	[illegible]	[illegible]
M00391: Exosome,_eukaryotes	0	0	3.65E-07	0	9.28E-07	1.87E-05	[illegible]	[illegible]	[illegible]	[illegible]	[illegible]	[illegible]	[illegible]	[illegible]	[illegible]	[illegible]	[illegible]	[illegible]	[illegible]	[illegible]	[illegible]	[illegible]	[illegible]	[illegible]	[illegible]	[illegible]	[illegible]	[illegible]	[illegible]	[illegible]	[illegible]	[illegible]	[illegible]	[illegible]	[illegible]	[illegible]	[illegible]	[illegible]	[illegible]	[illegible]	[illegible]	[illegible]	[illegible]	[illegible]	[illegible]	[illegible]	[illegible]	[illegible]	[illegible]	[illegible]	[illegible]	[illegible]	[illegible]	[illegible]	[illegible]	[illegible]	[illegible]	[illegible]	[illegible]	[illegible]	[illegible]	[illegible]	[illegible]	[illegible]	[illegible]	[illegible]	[illegible]
M00392: Ski_complex	0	0	0	0	0	0	[illegible]	[illegible]	[illegible]	[illegible]	[illegible]	[illegible]	[illegible]	[illegible]	[illegible]	[illegible]	[illegible]	[illegible]	[illegible]	[illegible]	[illegible]	[illegible]	[illegible]	[illegible]	[illegible]	[illegible]	[illegible]	[illegible]	[illegible]	[illegible]	[illegible]	[illegible]	[illegible]	[illegible]	[illegible]	[illegible]	[illegible]	[illegible]	[illegible]	[illegible]	[illegible]	[illegible]	[illegible]	[illegible]	[illegible]	[illegible]	[illegible]	[illegible]	[illegible]	[illegible]	[illegible]	[illegible]	[illegible]	[illegible]	[illegible]	[illegible]	[illegible]	[illegible]	[illegible]	[illegible]	[illegible]	[illegible]	[illegible]	[illegible]	[illegible]	[illegible]	[illegible]
M00394: RNA_degradosome	0.00188	0.00181	0.002103	0.001894	0.001833	0.001876	[illegible]	[illegible]	[illegible]	[illegible]	[illegible]	[illegible]	[illegible]	[illegible]	[illegible]	[illegible]	[illegible]	[illegible]	[illegible]	[illegible]	[illegible]	[illegible]	[illegible]	[illegible]	[illegible]	[illegible]	[illegible]	[illegible]	[illegible]	[illegible]	[illegible]	[illegible]	[illegible]	[illegible]	[illegible]	[illegible]	[illegible]	[illegible]	[illegible]	[illegible]	[illegible]	[illegible]	[illegible]	[illegible]	[illegible]	[illegible]	[illegible]	[illegible]	[illegible]	[illegible]	[illegible]	[illegible]	[illegible]	[illegible]	[illegible]	[illegible]	[illegible]	[illegible]	[illegible]	[illegible]	[illegible]	[illegible]	[illegible]	[illegible]	[illegible]	[illegible]	[illegible]
M00400: p97-Ufd1-Npl4_complex	8.08E-05	1.46E-05	1.64E-05	2.42E-05	3.56E-05	4.60E-05	[illegible]	[illegible]	[illegible]	[illegible]	[illegible]	[illegible]	[illegible]	[illegible]	[illegible]	[illegible]	[illegible]	[illegible]	[illegible]	[illegible]	[illegible]	[illegible]	[illegible]	[illegible]	[illegible]	[illegible]	[illegible]	[illegible]	[illegible]	[illegible]	[illegible]	[illegible]	[illegible]	[illegible]	[illegible]	[illegible]	[illegible]	[illegible]	[illegible]	[illegible]	[illegible]	[illegible]	[illegible]	[illegible]	[illegible]	[illegible]	[illegible]	[illegible]	[illegible]	[illegible]	[illegible]	[illegible]	[illegible]	[illegible]	[illegible]	[illegible]	[illegible]	[illegible]	[illegible]	[illegible]	[illegible]	[illegible]	[illegible]	[illegible]	[illegible]	[illegible]	[illegible]
M00403: HRD1/SEL1_ERAD_complex	0	0	0	0	0	0	[illegible]	[illegible]	[illegible]	[illegible]	[illegible]	[illegible]	[illegible]	[illegible]	[illegible]	[illegible]	[illegible]	[illegible]	[illegible]	[illegible]	[illegible]	[illegible]	[illegible]	[illegible]	[illegible]	[illegible]	[illegible]	[illegible]	[illegible]	[illegible]	[illegible]	[illegible]	[illegible]	[illegible]	[illegible]	[illegible]	[illegible]	[illegible]	[illegible]	[illegible]	[illegible]	[illegible]	[illegible]	[illegible]	[illegible]	[illegible]	[illegible]	[illegible]	[illegible]	[illegible]	[illegible]	[illegible]	[illegible]	[illegible]	[illegible]	[illegible]	[illegible]	[illegible]	[illegible]	[illegible]	[illegible]	[illegible]	[illegible]	[illegible]	[illegible]	[illegible]	[illegible]
M00404: COPII_complex	2.54E-05	[illegible]	[illegible]	[illegible]	[illegible]	[illegible]	[illegible]	[illegible]	[illegible]	[illegible]	[illegible]	[illegible]	[illegible]	[illegible]	[illegible]	[illegible]	[illegible]	[illegible]	[illegible]	[illegible]	[illegible]	[illegible]	[illegible]	[illegible]	[illegible]	[illegible]	[illegible]	[illegible]	[illegible]	[illegible]	[illegible]	[illegible]	[illegible]	[illegible]	[illegible]	[illegible]	[illegible]	[illegible]	[illegible]	[illegible]	[illegible]	[illegible]	[illegible]	[illegible]	[illegible]	[illegible]	[illegible]	[illegible]	[illegible]	[illegible]	[illegible]	[illegible]	[illegible]	[illegible]	[illegible]	[illegible]	[illegible]	[illegible]	[illegible]	[illegible]	[illegible]	[illegible]	[illegible]	[illegible]	[illegible]	[illegible]	[illegible]
M00413: FA_core_complex	7.75E-06	[illegible]	[illegible]	[illegible]	[illegible]	[illegible]	[illegible]	[illegible]	[illegible]	[illegible]	[illegible]	[illegible]	[illegible]	[illegible]	[illegible]	[illegible]	[illegible]	[illegible]	[illegible]	[illegible]	[illegible]	[illegible]	[illegible]	[illegible]	[illegible]	[illegible]	[illegible]	[illegible]	[illegible]	[illegible]	[illegible]	[illegible]	[illegible]	[illegible]	[illegible]	[illegible]	[illegible]	[illegible]	[illegible]	[illegible]	[illegible]	[illegible]	[illegible]	[illegible]	[illegible]	[illegible]	[illegible]	[illegible]	[illegible]	[illegible]	[illegible]	[illegible]	[illegible]	[illegible]	[illegible]	[illegible]	[illegible]	[illegible]	[illegible]	[illegible]	[illegible]	[illegible]	[illegible]	[illegible]	[illegible]	[illegible]	[illegible]
M00416: Cytochrome_aa3-600_menaquinol_oxidase	3.34E-06	[illegible]	[illegible]	[illegible]	[illegible]	[illegible]	[illegible]	[illegible]	[illegible]	[illegible]	[illegible]	[illegible]	[illegible]	[illegible]	[illegible]	[illegible]	[illegible]	[illegible]	[illegible]	[illegible]	[illegible]	[illegible]	[illegible]	[illegible]	[illegible]	[illegible]	[illegible]	[illegible]	[illegible]	[illegible]	[illegible]	[illegible]	[illegible]	[illegible]	[illegible]	[illegible]	[illegible]	[illegible]	[illegible]	[illegible]	[illegible]	[illegible]	[illegible]	[illegible]	[illegible]	[illegible]	[illegible]	[illegible]	[illegible]	[illegible]	[illegible]	[illegible]	[illegible]	[illegible]	[illegible]	[illegible]	[illegible]	[illegible]	[illegible]	[illegible]	[illegible]	[illegible]	[illegible]	[illegible]	[illegible]	[illegible]	[illegible]
M00417: Cytochrome_o_ubiquinol_oxidase	0.00082	[illegible]	[illegible]	[illegible]	[illegible]	[illegible]	[illegible]	[illegible]	[illegible]	[illegible]	[illegible]	[illegible]	[illegible]	[illegible]	[illegible]	[illegible]	[illegible]	[illegible]	[illegible]	[illegible]	[illegible]	[illegible]	[illegible]	[illegible]	[illegible]	[illegible]	[illegible]	[illegible]	[illegible]	[illegible]	[illegible]	[illegible]	[illegible]	[illegible]	[illegible]	[illegible]	[illegible]	[illegible]	[illegible]	[illegible]	[illegible]	[illegible]	[illegible]	[illegible]	[illegible]	[illegible]	[illegible]	[illegible]	[illegible]	[illegible]	[illegible]	[illegible]	[illegible]	[illegible]	[illegible]	[illegible]	[illegible]	[illegible]	[illegible]	[illegible]	[illegible]	[illegible]	[illegible]	[illegible]	[illegible]	[illegible]	[illegible]
M00418: Toluene_degradation,_anaerobic,_toluene_=>_benzoyl-CoA	1.76E-05	[illegible]	[illegible]	[illegible]	[illegible]	[illegible]	[illegible]	[illegible]	[illegible]	[illegible]	[illegible]	[illegible]	[illegible]	[illegible]	[illegible]	[illegible]	[illegible]	[illegible]	[illegible]	[illegible]	[illegible]	[illegible]	[illegible]	[illegible]	[illegible]	[illegible]	[illegible]	[illegible]	[illegible]	[illegible]	[illegible]	[illegible]	[illegible]	[illegible]	[illegible]	[illegible]	[illegible]	[illegible]	[illegible]	[illegible]	[illegible]	[illegible]	[illegible]	[illegible]	[illegible]	[illegible]	[illegible]	[illegible]	[illegible]	[illegible]	[illegible]	[illegible]	[illegible]	[illegible]	[illegible]	[illegible]	[illegible]	[illegible]	[illegible]	[illegible]	[illegible]	[illegible]	[illegible]	[illegible]	[illegible]	[illegible]	[illegible]
M00423: Molybdate/tungstate_transport_system	7.35E-06	[illegible]	[illegible]	[illegible]	[illegible]	[illegible]	[illegible]	[illegible]	[illegible]	[illegible]	[illegible]	[illegible]	[illegible]	[illegible]	[illegible]	[illegible]	[illegible]	[illegible]	[illegible]	[illegible]	[illegible]	[illegible]	[illegible]	[illegible]	[illegible]	[illegible]	[illegible]	[illegible]	[illegible]	[illegible]	[illegible]	[illegible]	[illegible]	[illegible]	[illegible]	[illegible]	[illegible]	[illegible]	[illegible]	[illegible]	[illegible]	[illegible]	[illegible]	[illegible]	[illegible]	[illegible]	[illegible]	[illegible]	[illegible]	[illegible]	[illegible]	[illegible]	[illegible]	[illegible]	[illegible]	[illegible]	[illegible]	[illegible]	[illegible]	[illegible]	[illegible]	[illegible]	[illegible]	[illegible]	[illegible]	[illegible]	[illegible]
M00427: Nuclear_pore_complex	0	[illegible]	[illegible]	[illegible]	[illegible]	[illegible]	[illegible]	[illegible]	[illegible]	[illegible]	[illegible]	[illegible]	[illegible]	[illegible]	[illegible]	[illegible]	[illegible]	[illegible]	[illegible]	[illegible]	[illegible]	[illegible]	[illegible]	[illegible]	[illegible]	[illegible]	[illegible]	[illegible]	[illegible]	[illegible]	[illegible]	[illegible]	[illegible]	[illegible]	[illegible]	[illegible]	[illegible]	[illegible]	[illegible]	[illegible]	[illegible]	[illegible]	[illegible]	[illegible]	[illegible]	[illegible]	[illegible]	[illegible]	[illegible]	[illegible]	[illegible]	[illegible]	[illegible]	[illegible]	[illegible]	[illegible]	[illegible]	[illegible]	[illegible]	[illegible]	[illegible]	[illegible]	[illegible]	[illegible]	[illegible]	[illegible]	[illegible]
M00428: eIF4F_complex	6.68E-07	[illegible]	[illegible]	[illegible]	[illegible]	[illegible]	[illegible]	[illegible]	[illegible]	[illegible]	[illegible]	[illegible]	[illegible]	[illegible]	[illegible]	[illegible]	[illegible]	[illegible]	[illegible]	[illegible]	[illegible]	[illegible]	[illegible]	[illegible]	[illegible]	[illegible]	[illegible]	[illegible]	[illegible]	[illegible]	[illegible]	[illegible]	[illegible]	[illegible]	[illegible]	[illegible]	[illegible]	[illegible]	[illegible]	[illegible]	[illegible]	[illegible]	[illegible]	[illegible]	[illegible]	[illegible]	[illegible]	[illegible]	[illegible]	[illegible]	[illegible]	[illegible]	[illegible]	[illegible]	[illegible]	[illegible]	[illegible]	[illegible]	[illegible]	[illegible]	[illegible]	[illegible]	[illegible]	[illegible]	[illegible]	[illegible]	[illegible]
M00429: Competence-related_DNA_transformation_transporter	0.000353	[illegible]	[illegible]	[illegible]	[illegible]	[illegible]	[illegible]	[illegible]	[illegible]	[illegible]	[illegible]	[illegible]	[illegible]	[illegible]	[illegible]	[illegible]	[illegible]	[illegible]	[illegible]	[illegible]	[illegible]	[illegible]	[illegible]	[illegible]	[illegible]	[illegible]	[illegible]	[illegible]	[illegible]	[illegible]	[illegible]	[illegible]	[illegible]	[illegible]	[illegible]	[illegible]	[illegible]	[illegible]	[illegible]	[illegible]	[illegible]	[illegible]	[illegible]	[illegible]	[illegible]	[illegible]	[illegible]	[illegible]	[illegible]	[illegible]	[illegible]	[illegible]	[illegible]	[illegible]	[illegible]	[illegible]	[illegible]	[illegible]	[illegible]	[illegible]	[illegible]	[illegible]	[illegible]	[illegible]	[illegible]	[illegible]	[illegible]
M00432: Leucine_biosynthesis,_2-oxoisovalerate_=>_2-oxoisocaproate	0.00195	[illegible]	[illegible]	[illegible]	[illegible]	[illegible]	[illegible]	[illegible]	[illegible]	[illegible]	[illegible]	[illegible]	[illegible]	[illegible]	[illegible]	[illegible]	[illegible]	[illegible]	[illegible]	[illegible]	[illegible]	[illegible]	[illegible]	[illegible]	[illegible]	[illegible]	[illegible]	[illegible]	[illegible]	[illegible]	[illegible]	[illegible]	[illegible]	[illegible]	[illegible]	[illegible]	[illegible]	[illegible]	[illegible]	[illegible]	[illegible]	[illegible]	[illegible]	[illegible]	[illegible]	[illegible]	[illegible]	[illegible]	[illegible]	[illegible]	[illegible]	[illegible]	[illegible]	[illegible]	[illegible]	[illegible]	[illegible]	[illegible]	[illegible]	[illegible]	[illegible]	[illegible]	[illegible]	[illegible]	[illegible]	[illegible]	[illegible]
M00433: Lysine_biosynthesis,_2-oxoglutarate_=>_2-oxoadipate	2.67E-05	[illegible]	[illegible]	[illegible]	[illegible]	[illegible]	[illegible]	[illegible]	[illegible]	[illegible]	[illegible]	[illegible]	[illegible]	[illegible]	[illegible]	[illegible]	[illegible]	[illegible]	[illegible]	[illegible]	[illegible]	[illegible]	[illegible]	[illegible]	[illegible]	[illegible]	[illegible]	[illegible]	[illegible]	[illegible]	[illegible]	[illegible]	[illegible]	[illegible]	[illegible]	[illegible]	[illegible]	[illegible]	[illegible]	[illegible]	[illegible]	[illegible]	[illegible]	[illegible]	[illegible]	[illegible]	[illegible]	[illegible]	[illegible]	[illegible]	[illegible]	[illegible]	[illegible]	[illegible]	[illegible]	[illegible]	[illegible]	[illegible]	[illegible]	[illegible]	[illegible]	[illegible]	[illegible]	[illegible]	[illegible]	[illegible]	[illegible]
M00434: PhoR-PhoB_(phosphate_starvation_response)_two-component_regulatory_system	0.001785	[illegible]	[illegible]	[illegible]	[illegible]	[illegible]	[illegible]	[illegible]	[illegible]	[illegible]	[illegible]	[illegible]	[illegible]	[illegible]	[illegible]	[illegible]	[illegible]	[illegible]	[illegible]	[illegible]	[illegible]	[illegible]	[illegible]	[illegible]	[illegible]	[illegible]	[illegible]	[illegible]	[illegible]	[illegible]	[illegible]	[illegible]	[illegible]	[illegible]	[illegible]	[illegible]	[illegible]	[illegible]	[illegible]	[illegible]	[illegible]	[illegible]	[illegible]	[illegible]	[illegible]	[illegible]	[illegible]	[illegible]	[illegible]	[illegible]	[illegible]	[illegible]	[illegible]	[illegible]	[illegible]	[illegible]	[illegible]	[illegible]	[illegible]	[illegible]	[illegible]	[illegible]	[illegible]	[illegible]	[illegible]	[illegible]	[illegible]
M00435: Taurine_transport_system	4.68E-05	[illegible]	[illegible]	[illegible]	[illegible]	[illegible]	[illegible]	[illegible]	[illegible]	[illegible]	[illegible]	[illegible]	[illegible]	[illegible]	[illegible]	[illegible]	[illegible]	[illegible]	[illegible]	[illegible]	[illegible]	[illegible]	[illegible]	[illegible]	[illegible]	[illegible]	[illegible]	[illegible]	[illegible]	[illegible]	[illegible]	[illegible]	[illegible]	[illegible]	[illegible]	[illegible]	[illegible]	[illegible]	[illegible]	[illegible]	[illegible]	[illegible]	[illegible]	[illegible]	[illegible]	[illegible]	[illegible]	[illegible]	[illegible]	[illegible]	[illegible]	[illegible]	[illegible]	[illegible]	[illegible]	[illegible]	[illegible]	[illegible]	[illegible]	[illegible]	[illegible]	[illegible]	[illegible]	[illegible]	[illegible]	[illegible]	[illegible]
M00436: Sulfonate_transport_system	0.000339	[illegible]	[illegible]	[illegible]	[illegible]	[illegible]	[illegible]	[illegible]	[illegible]	[illegible]	[illegible]	[illegible]	[illegible]	[illegible]	[illegible]	[illegible]	[illegible]	[illegible]	[illegible]	[illegible]	[illegible]	[illegible]	[illegible]	[illegible]	[illegible]	[illegible]	[illegible]	[illegible]	[illegible]	[illegible]	[illegible]	[illegible]	[illegible]	[illegible]	[illegible]	[illegible]	[illegible]	[illegible]	[illegible]	[illegible]	[illegible]	[illegible]	[illegible]	[illegible]	[illegible]	[illegible]	[illegible]	[illegible]	[illegible]	[illegible]	[illegible]	[illegible]	[illegible]	[illegible]	[illegible]	[illegible]	[illegible]	[illegible]	[illegible]	[illegible]	[illegible]	[illegible]	[illegible]	[illegible]	[illegible]	[illegible]	[illegible]
M00438: Nitrate/nitrite_transport_system	0.000441	[illegible]	[illegible]	[illegible]	[illegible]	[illegible]	[illegible]	[illegible]	[illegible]	[illegible]	[illegible]	[illegible]	[illegible]	[illegible]	[illegible]	[illegible]	[illegible]	[illegible]	[illegible]	[illegible]	[illegible]	[illegible]	[illegible]	[illegible]	[illegible]	[illegible]	[illegible]	[illegible]	[illegible]	[illegible]	[illegible]	[illegible]	[illegible]	[illegible]	[illegible]	[illegible]	[illegible]	[illegible]	[illegible]	[illegible]	[illegible]	[illegible]	[illegible]	[illegible]	[illegible]	[illegible]	[illegible]	[illegible]	[illegible]	[illegible]	[illegible]	[illegible]	[illegible]	[illegible]	[illegible]	[illegible]	[illegible]	[illegible]	[illegible]	[illegible]	[illegible]	[illegible]	[illegible]	[illegible]	[illegible]	[illegible]	[illegible]
M00439: Oligopeptide_transport_system	0.000328	[illegible]	[illegible]	[illegible]	[illegible]	[illegible]	[illegible]	[illegible]	[illegible]	[illegible]	[illegible]	[illegible]	[illegible]	[illegible]	[illegible]	[illegible]	[illegible]	[illegible]	[illegible]	[illegible]	[illegible]	[illegible]	[illegible]	[illegible]	[illegible]	[illegible]	[illegible]	[illegible]	[illegible]	[illegible]	[illegible]	[illegible]	[illegible]	[illegible]	[illegible]	[illegible]	[illegible]	[illegible]	[illegible]	[illegible]	[illegible]	[illegible]	[illegible]	[illegible]	[illegible]	[illegible]	[illegible]	[illegible]	[illegible]	[illegible]	[illegible]	[illegible]	[illegible]	[illegible]	[illegible]	[illegible]	[illegible]	[illegible]	[illegible]	[illegible]	[illegible]	[illegible]	[illegible]	[illegible]	[illegible]	[illegible]	[illegible]
M00440: Nickel_transport_system	0	[illegible]	[illegible]	[illegible]	[illegible]	[illegible]	[illegible]	[illegible]	[illegible]	[illegible]	[illegible]	[illegible]	[illegible]	[illegible]	[illegible]	[illegible]	[illegible]	[illegible]	[illegible]	[illegible]	[illegible]	[illegible]	[illegible]	[illegible]	[illegible]	[illegible]	[illegible]	[illegible]	[illegible]	[illegible]	[illegible]	[illegible]	[illegible]	[illegible]	[illegible]	[illegible]	[illegible]	[illegible]	[illegible]	[illegible]	[illegible]	[illegible]	[illegible]	[illegible]	[illegible]	[illegible]	[illegible]	[illegible]	[illegible]	[illegible]	[illegible]	[illegible]	[illegible]	[illegible]	[illegible]	[illegible]	[illegible]	[illegible]	[illegible]	[illegible]	[illegible]	[illegible]	[illegible]	[illegible]	[illegible]	[illegible]	[illegible]
M00442: Putative_hydroxymethylpyrimidine_transport_system	4.74E-05	[illegible]	[illegible]	[illegible]	[illegible]	[illegible]	[illegible]	[illegible]	[illegible]	[illegible]	[illegible]	[illegible]	[illegible]	[illegible]	[illegible]	[illegible]	[illegible]	[illegible]	[illegible]	[illegible]	[illegible]	[illegible]	[illegible]	[illegible]	[illegible]	[illegible]	[illegible]	[illegible]	[illegible]	[illegible]	[illegible]	[illegible]	[illegible]	[illegible]	[illegible]	[illegible]	[illegible]	[illegible]	[illegible]	[illegible]	[illegible]	[illegible]	[illegible]	[illegible]	[illegible]	[illegible]	[illegible]	[illegible]	[illegible]	[illegible]	[illegible]	[illegible]	[illegible]	[illegible]	[illegible]	[illegible]	[illegible]	[illegible]	[illegible]	[illegible]	[illegible]	[illegible]	[illegible]	[illegible]	[illegible]	[illegible]	[illegible]
M00443: SenX3-RegX3_(phosphate_starvation_response)_two-component_regulatory_system	9.75E-05	[illegible]	[illegible]	[illegible]	[illegible]	[illegible]	[illegible]	[illegible]	[illegible]	[illegible]	[illegible]	[illegible]	[illegible]	[illegible]	[illegible]	[illegible]	[illegible]	[illegible]	[illegible]	[illegible]	[illegible]	[illegible]	[illegible]	[illegible]	[illegible]	[illegible]	[illegible]	[illegible]	[illegible]	[illegible]	[illegible]	[illegible]	[illegible]	[illegible]	[illegible]	[illegible]	[illegible]	[illegible]	[illegible]	[illegible]	[illegible]	[illegible]	[illegible]	[illegible]	[illegible]	[illegible]	[illegible]	[illegible]	[illegible]	[illegible]	[illegible]	[illegible]	[illegible]	[illegible]	[illegible]	[illegible]	[illegible]	[illegible]	[illegible]	[illegible]	[illegible]	[illegible]	[illegible]	[illegible]	[illegible]	[illegible]	[illegible]
M00444: PhoQ-PhoP_(magnesium_transport)_two-component_regulatory_system	0.00103	[illegible]	[illegible]	[illegible]	[illegible]	[illegible]	[illegible]	[illegible]	[illegible]	[illegible]	[illegible]	[illegible]	[illegible]	[illegible]	[illegible]	[illegible]	[illegible]	[illegible]	[illegible]	[illegible]	[illegible]	[illegible]	[illegible]	[illegible]	[illegible]	[illegible]	[illegible]	[illegible]	[illegible]	[illegible]	[illegible]	[illegible]	[illegible]	[illegible]	[illegible]	[illegible]	[illegible]	[illegible]	[illegible]	[illegible]	[illegible]	[illegible]	[illegible]	[illegible]	[illegible]	[illegible]	[illegible]	[illegible]	[illegible]	[illegible]	[illegible]	[illegible]	[illegible]	[illegible]	[illegible]	[illegible]	[illegible]	[illegible]	[illegible]	[illegible]	[illegible]	[illegible]	[illegible]	[illegible]	[illegible]	[illegible]	[illegible]
M00445: EnvZ-OmpR_(osmotic_stress_response)_two-component_regulatory_system	0.000625	[illegible]	[illegible]	[illegible]	[illegible]	[illegible]	[illegible]	[illegible]	[illegible]	[illegible]	[illegible]	[illegible]	[illegible]	[illegible]	[illegible]	[illegible]	[illegible]	[illegible]	[illegible]	[illegible]	[illegible]	[illegible]	[illegible]	[illegible]	[illegible]	[illegible]	[illegible]	[illegible]	[illegible]	[illegible]	[illegible]	[illegible]	[illegible]	[illegible]	[illegible]	[illegible]	[illegible]	[illegible]	[illegible]	[illegible]	[illegible]	[illegible]	[illegible]	[illegible]	[illegible]	[illegible]	[illegible]	[illegible]	[illegible]	[illegible]	[illegible]	[illegible]	[illegible]	[illegible]	[illegible]	[illegible]	[illegible]	[illegible]	[illegible]	[illegible]	[illegible]	[illegible]	[illegible]	[illegible]	[illegible]	[illegible]	[illegible]
M00446: RstB-RstA_two-component_regulatory_system	0	[illegible]	[illegible]	[illegible]	[illegible]	[illegible]	[illegible]	[illegible]	[illegible]	[illegible]	[illegible]	[illegible]	[illegible]	[illegible]	[illegible]	[illegible]	[illegible]	[illegible]	[illegible]	[illegible]	[illegible]	[illegible]	[illegible]	[illegible]	[illegible]	[illegible]	[illegible]	[illegible]	[illegible]	[illegible]	[illegible]	[illegible]	[illegible]	[illegible]	[illegible]	[illegible]	[illegible]	[illegible]	[illegible]	[illegible]	[illegible]	[illegible]	[illegible]	[illegible]	[illegible]	[illegible]	[illegible]	[illegible]	[illegible]	[illegible]	[illegible]	[illegible]	[illegible]	[illegible]	[illegible]	[illegible]	[illegible]	[illegible]	[illegible]	[illegible]	[illegible]	[illegible]	[illegible]	[illegible]	[illegible]	[illegible]	[illegible]
M00447: CpxA-CpxR_(envelope_stress_response)_two-component_regulatory_system	0.000858	[illegible]	[illegible]	[illegible]	[illegible]	[illegible]	[illegible]	[illegible]	[illegible]	[illegible]	[illegible]	[illegible]	[illegible]	[illegible]	[illegible]	[illegible]	[illegible]	[illegible]	[illegible]	[illegible]	[illegible]	[illegible]	[illegible]	[illegible]	[illegible]	[illegible]	[illegible]	[illegible]	[illegible]	[illegible]	[illegible]	[illegible]	[illegible]	[illegible]	[illegible]	[illegible]	[illegible]	[illegible]	[illegible]	[illegible]	[illegible]	[illegible]	[illegible]	[illegible]	[illegible]	[illegible]	[illegible]	[illegible]	[illegible]	[illegible]	[illegible]	[illegible]	[illegible]	[illegible]	[illegible]	[illegible]	[illegible]	[illegible]	[illegible]	[illegible]	[illegible]	[illegible]	[illegible]	[illegible]	[illegible]	[illegible]	[illegible]
M00448: CssS-CssR_(secretion_stress_response)_two-component_regulatory_system	0	[illegible]	[illegible]	[illegible]	[illegible]	[illegible]	[illegible]	[illegible]	[illegible]	[illegible]	[illegible]	[illegible]	[illegible]	[illegible]	[illegible]	[illegible]	[illegible]	[illegible]	[illegible]	[illegible]	[illegible]	[illegible]	[illegible]	[illegible]	[illegible]	[illegible]	[illegible]	[illegible]	[illegible]	[illegible]	[illegible]	[illegible]	[illegible]	[illegible]	[illegible]	[illegible]	[illegible]	[illegible]	[illegible]	[illegible]	[illegible]	[illegible]	[illegible]	[illegible]	[illegible]	[illegible]	[illegible]	[illegible]	[illegible]	[illegible]	[illegible]	[illegible]	[illegible]	[illegible]	[illegible]	[illegible]	[illegible]	[illegible]	[illegible]	[illegible]	[illegible]	[illegible]	[illegible]	[illegible]	[illegible]	[illegible]	[illegible]
M00449: CreC-CreB_(phosphate_regulation)_two-component_regulatory_system	0.000345	[illegible]	[illegible]	[illegible]	[illegible]	[illegible]	[illegible]	[illegible]	[illegible]	[illegible]	[illegible]	[illegible]	[illegible]	[illegible]	[illegible]	[illegible]	[illegible]	[illegible]	[illegible]	[illegible]	[illegible]	[illegible]	[illegible]	[illegible]	[illegible]	[illegible]	[illegible]	[illegible]	[illegible]	[illegible]	[illegible]	[illegible]	[illegible]	[illegible]	[illegible]	[illegible]	[illegible]	[illegible]	[illegible]	[illegible]	[illegible]	[illegible]	[illegible]	[illegible]	[illegible]	[illegible]	[illegible]	[illegible]	[illegible]	[illegible]	[illegible]	[illegible]	[illegible]	[illegible]	[illegible]	[illegible]	[illegible]	[illegible]	[illegible]	[illegible]	[illegible]	[illegible]	[illegible]	[illegible]	[illegible]	[illegible]	[illegible]
M00450: BaeS-BaeR_(envelope_stress_response)_two-component_regulatory_system	0.0001	[illegible]	[illegible]	[illegible]	[illegible]	[illegible]	[illegible]	[illegible]	[illegible]	[illegible]	[illegible]	[illegible]	[illegible]	[illegible]	[illegible]	[illegible]	[illegible]	[illegible]	[illegible]	[illegible]	[illegible]	[illegible]	[illegible]	[illegible]	[illegible]	[illegible]	[illegible]	[illegible]	[illegible]	[illegible]	[illegible]	[illegible]	[illegible]	[illegible]	[illegible]	[illegible]	[illegible]	[illegible]	[illegible]	[illegible]	[illegible]	[illegible]	[illegible]	[illegible]	[illegible]	[illegible]	[illegible]	[illegible]	[illegible]	[illegible]	[illegible]	[illegible]	[illegible]	[illegible]	[illegible]	[illegible]	[illegible]	[illegible]	[illegible]	[illegible]	[illegible]	[illegible]	[illegible]	[illegible]	[illegible]	[illegible]	[illegible]
M00451: BasS-BasR_(antimicrobial_peptide_resistance)_two-component_regulatory_system	4.01E-06	[illegible]	[illegible]	[illegible]	[illegible]	[illegible]	[illegible]	[illegible]	[illegible]	[illegible]	[illegible]	[illegible]	[illegible]	[illegible]	[illegible]	[illegible]	[illegible]	[illegible]	[illegible]	[illegible]	[illegible]	[illegible]	[illegible]	[illegible]	[illegible]	[illegible]	[illegible]	[illegible]	[illegible]	[illegible]	[illegible]	[illegible]	[illegible]	[illegible]	[illegible]	[illegible]	[illegible]	[illegible]	[illegible]	[illegible]	[illegible]	[illegible]	[illegible]	[illegible]	[illegible]	[illegible]	[illegible]	[illegible]	[illegible]	[illegible]	[illegible]	[illegible]	[illegible]	[illegible]	[illegible]	[illegible]	[illegible]	[illegible]	[illegible]	[illegible]	[illegible]	[illegible]	[illegible]	[illegible]	[illegible]	[illegible]	[illegible]
M00452: CusS-CusR_(copper_tolerance)_two-component_regulatory_system	0.001877	[illegible]	[illegible]	[illegible]	[illegible]	[illegible]	[illegible]	[illegible]	[illegible]	[illegible]	[illegible]	[illegible]	[illegible]	[illegible]	[illegible]	[illegible]	[illegible]	[illegible]	[illegible]	[illegible]	[illegible]	[illegible]	[illegible]	[illegible]	[illegible]	[illegible]	[illegible]	[illegible]	[illegible]	[illegible]	[illegible]	[illegible]	[illegible]	[illegible]	[illegible]	[illegible]	[illegible]	[illegible]	[illegible]	[illegible]	[illegible]	[illegible]	[illegible]	[illegible]	[illegible]	[illegible]	[illegible]	[illegible]	[illegible]	[illegible]	[illegible]	[illegible]	[illegible]	[illegible]	[illegible]	[illegible]	[illegible]	[illegible]	[illegible]	[illegible]	[illegible]	[illegible]	[illegible]	[illegible]	[illegible]	[illegible]	[illegible]
M00453: QseC-QseB_(quorum_sensing)_two-component_regulatory_system	0.00039	[illegible]	[illegible]	[illegible]	[illegible]	[illegible]	[illegible]	[illegible]	[illegible]	[illegible]	[illegible]	[illegible]	[illegible]	[illegible]	[illegible]	[illegible]	[illegible]	[illegible]	[illegible]	[illegible]	[illegible]	[illegible]	[illegible]	[illegible]	[illegible]	[illegible]	[illegible]	[illegible]	[illegible]	[illegible]	[illegible]	[illegible]	[illegible]	[illegible]	[illegible]	[illegible]	[illegible]	[illegible]	[illegible]	[illegible]	[illegible]	[illegible]	[illegible]	[illegible]	[illegible]	[illegible]	[illegible]	[illegible]	[illegible]	[illegible]	[illegible]	[illegible]	[illegible]	[illegible]	[illegible]	[illegible]	[illegible]	[illegible]	[illegible]	[illegible]	[illegible]	[illegible]	[illegible]	[illegible]	[illegible]	[illegible]	[illegible]
M00454: KdpD-KdpE_(potassium_transport)_two-component_regulatory_system	0.002457	[illegible]	[illegible]	[illegible]	[illegible]	[illegible]	[illegible]	[illegible]	[illegible]	[illegible]	[illegible]	[illegible]	[illegible]	[illegible]	[illegible]	[illegible]	[illegible]	[illegible]	[illegible]	[illegible]	[illegible]	[illegible]	[illegible]	[illegible]	[illegible]	[illegible]	[illegible]	[illegible]	[illegible]	[illegible]	[illegible]	[illegible]	[illegible]	[illegible]	[illegible]	[illegible]	[illegible]	[illegible]	[illegible]	[illegible]	[illegible]	[illegible]	[illegible]	[illegible]	[illegible]	[illegible]	[illegible]	[illegible]	[illegible]	[illegible]	[illegible]	[illegible]	[illegible]	[illegible]	[illegible]	[illegible]	[illegible]	[illegible]	[illegible]	[illegible]	[illegible]	[illegible]	[illegible]	[illegible]	[illegible]	[illegible]	[illegible]
M00455: TorS-TorR_(TMAO_respiration)_two-component_regulatory_system	9.75E-05	[illegible]	[illegible]	[illegible]	[illegible]	[illegible]	[illegible]	[illegible]	[illegible]	[illegible]	[illegible]	[illegible]	[illegible]	[illegible]	[illegible]	[illegible]	[illegible]	[illegible]	[illegible]	[illegible]	[illegible]	[illegible]	[illegible]	[illegible]	[illegible]	[illegible]	[illegible]	[illegible]	[illegible]	[illegible]	[illegible]	[illegible]	[illegible]	[illegible]	[illegible]	[illegible]	[illegible]	[illegible]	[illegible]	[illegible]	[illegible]	[illegible]	[illegible]	[illegible]	[illegible]	[illegible]	[illegible]	[illegible]	[illegible]	[illegible]	[illegible]	[illegible]	[illegible]	[illegible]	[illegible]	[illegible]	[illegible]	[illegible]	[illegible]	[illegible]	[illegible]	[illegible]	[illegible]	[illegible]	[illegible]	[illegible]	[illegible]
M00456: ArcB-ArcA_(anoxic_redox_control)_two-component_regulatory_system	0	[illegible]	[illegible]	[illegible]	[illegible]	[illegible]	[illegible]	[illegible]	[illegible]	[illegible]	[illegible]	[illegible]	[illegible]	[illegible]	[illegible]	[illegible]	[illegible]	[illegible]	[illegible]	[illegible]	[illegible]	[illegible]	[illegible]	[illegible]	[illegible]	[illegible]	[illegible]	[illegible]	[illegible]	[illegible]	[illegible]	[illegible]	[illegible]	[illegible]	[illegible]	[illegible]	[illegible]	[illegible]	[illegible]	[illegible]	[illegible]	[illegible]	[illegible]	[illegible]	[illegible]	[illegible]	[illegible]	[illegible]	[illegible]	[illegible]	[illegible]	[illegible]	[illegible]	[illegible]	[illegible]	[illegible]	[illegible]	[illegible]	[illegible]	[illegible]	[illegible]	[illegible]	[illegible]	[illegible]	[illegible]	[illegible]	[illegible]
M00457: TctE-TctD_(tricarboxylic_acid_transport)_two-component_regulatory_system	0.000518	[illegible]	[illegible]	[illegible]	[illegible]	[illegible]	[illegible]	[illegible]	[illegible]	[illegible]	[illegible]	[illegible]	[illegible]	[illegible]	[illegible]	[illegible]	[illegible]	[illegible]	[illegible]	[illegible]	[illegible]	[illegible]	[illegible]	[illegible]	[illegible]	[illegible]	[illegible]	[illegible]	[illegible]	[illegible]	[illegible]	[illegible]	[illegible]	[illegible]	[illegible]	[illegible]	[illegible]	[illegible]	[illegible]	[illegible]	[illegible]	[illegible]	[illegible]	[illegible]	[illegible]	[illegible]	[illegible]	[illegible]	[illegible]	[illegible]	[illegible]	[illegible]	[illegible]	[illegible]	[illegible]	[illegible]	[illegible]	[illegible]	[illegible]	[illegible]	[illegible]	[illegible]	[illegible]	[illegible]	[illegible]	[illegible]	[illegible]
M00458: ResE-ResD_(aerobic_and_anaerobic_respiration)_two-component_regulatory_system	2.67E-06	[illegible]	[illegible]	[illegible]	[illegible]	[illegible]	[illegible]	[illegible]	[illegible]	[illegible]	[illegible]	[illegible]	[illegible]	[illegible]	[illegible]	[illegible]	[illegible]	[illegible]	[illegible]	[illegible]	[illegible]	[illegible]	[illegible]	[illegible]	[illegible]	[illegible]	[illegible]	[illegible]	[illegible]	[illegible]	[illegible]	[illegible]	[illegible]	[illegible]	[illegible]	[illegible]	[illegible]	[illegible]	[illegible]	[illegible]	[illegible]	[illegible]	[illegible]	[illegible]	[illegible]	[illegible]	[illegible]	[illegible]	[illegible]	[illegible]	[illegible]	[illegible]	[illegible]	[illegible]	[illegible]	[illegible]	[illegible]	[illegible]	[illegible]	[illegible]	[illegible]	[illegible]	[illegible]	[illegible]	[illegible]	[illegible]	[illegible]
M00459: VicK-VicR_(cell_wall_metabolism)_two-component_regulatory_system	4.27E-05	[illegible]	[illegible]	[illegible]	[illegible]	[illegible]	[illegible]	[illegible]	[illegible]	[illegible]	[illegible]	[illegible]	[illegible]	[illegible]	[illegible]	[illegible]	[illegible]	[illegible]	[illegible]	[illegible]	[illegible]	[illegible]	[illegible]	[illegible]	[illegible]	[illegible]	[illegible]	[illegible]	[illegible]	[illegible]	[illegible]	[illegible]	[illegible]	[illegible]	[illegible]	[illegible]	[illegible]	[illegible]	[illegible]	[illegible]	[illegible]	[illegible]	[illegible]	[illegible]	[illegible]	[illegible]	[illegible]	[illegible]	[illegible]	[illegible]	[illegible]	[illegible]	[illegible]	[illegible]	[illegible]	[illegible]	[illegible]	[illegible]	[illegible]	[illegible]	[illegible]	[illegible]	[illegible]	[illegible]	[illegible]	[illegible]	[illegible]
M00460: MprB-MprA_(maintenance_of_persistent_infection)_two-component_regulatory_system	0.000146	[illegible]	[illegible]	[illegible]	[illegible]	[illegible]	[illegible]	[illegible]	[illegible]	[illegible]	[illegible]	[illegible]	[illegible]	[illegible]	[illegible]	[illegible]	[illegible]	[illegible]	[illegible]	[illegible]	[illegible]	[illegible]	[illegible]	[illegible]	[illegible]	[illegible]	[illegible]	[illegible]	[illegible]	[illegible]	[illegible]	[illegible]	[illegible]	[illegible]	[illegible]	[illegible]	[illegible]	[illegible]	[illegible]	[illegible]	[illegible]	[illegible]	[illegible]	[illegible]	[illegible]	[illegible]	[illegible]	[illegible]	[illegible]	[illegible]	[illegible]	[illegible]	[illegible]	[illegible]	[illegible]	[illegible]	[illegible]	[illegible]	[illegible]	[illegible]	[illegible]	[illegible]	[illegible]	[illegible]	[illegible]	[illegible]	[illegible]
M00461: MtrB-MtrA_(osmotic_stress_response)_two-component_regulatory_system	0.000207	[illegible]	[illegible]	[illegible]	[illegible]	[illegible]	[illegible]	[illegible]	[illegible]	[illegible]	[illegible]	[illegible]	[illegible]	[illegible]	[illegible]	[illegible]	[illegible]	[illegible]	[illegible]	[illegible]	[illegible]	[illegible]	[illegible]	[illegible]	[illegible]	[illegible]	[illegible]	[illegible]	[illegible]	[illegible]	[illegible]	[illegible]	[illegible]	[illegible]	[illegible]	[illegible]	[illegible]	[illegible]	[illegible]	[illegible]	[illegible]	[illegible]	[illegible]	[illegible]	[illegible]	[illegible]	[illegible]	[illegible]	[illegible]	[illegible]	[illegible]	[illegible]	[illegible]	[illegible]	[illegible]	[illegible]	[illegible]	[illegible]	[illegible]	[illegible]	[illegible]	[illegible]	[illegible]	[illegible]	[illegible]	[illegible]	[illegible]
M00462: PrrB-PrrA_(intracellular_multiplication)_two-component_regulatory_system	2.67E-06	[illegible]	[illegible]	[illegible]	[illegible]	[illegible]	[illegible]	[illegible]	[illegible]	[illegible]	[illegible]	[illegible]	[illegible]	[illegible]	[illegible]	[illegible]	[illegible]	[illegible]	[illegible]	[illegible]	[illegible]	[illegible]	[illegible]	[illegible]	[illegible]	[illegible]	[illegible]	[illegible]	[illegible]	[illegible]	[illegible]	[illegible]	[illegible]	[illegible]	[illegible]	[illegible]	[illegible]	[illegible]	[illegible]	[illegible]	[illegible]	[illegible]	[illegible]	[illegible]	[illegible]	[illegible]	[illegible]	[illegible]	[illegible]	[illegible]	[illegible]	[illegible]	[illegible]	[illegible]	[illegible]	[illegible]	[illegible]	[illegible]	[illegible]	[illegible]	[illegible]	[illegible]	[illegible]	[illegible]	[illegible]	[illegible]	[illegible]
M00464: NrsS-NrsR_(nickel_tolerance)_two-component_regulatory_system	0	[illegible]	[illegible]	[illegible]	[illegible]	[illegible]	[illegible]	[illegible]	[illegible]	[illegible]	[illegible]	[illegible]	[illegible]	[illegible]	[illegible]	[illegible]	[illegible]	[illegible]	[illegible]	[illegible]	[illegible]	[illegible]	[illegible]	[illegible]	[illegible]	[illegible]	[illegible]	[illegible]	[illegible]	[illegible]	[illegible]	[illegible]	[illegible]	[illegible]	[illegible]	[illegible]	[illegible]	[illegible]	[illegible]	[illegible]	[illegible]	[illegible]	[illegible]	[illegible]	[illegible]	[illegible]	[illegible]	[illegible]	[illegible]	[illegible]	[illegible]	[illegible]	[illegible]	[illegible]	[illegible]	[illegible]	[illegible]	[illegible]	[illegible]	[illegible]	[illegible]	[illegible]	[illegible]	[illegible]	[illegible]	[illegible]	[illegible]
M00465: ManS-ManR_(manganese_homeostasis)_two-component_regulatory_system	1.34E-06	[illegible]	[illegible]	[illegible]	[illegible]	[illegible]	[illegible]	[illegible]	[illegible]	[illegible]	[illegible]	[illegible]	[illegible]	[illegible]	[illegible]	[illegible]	[illegible]	[illegible]	[illegible]	[illegible]	[illegible]	[illegible]	[illegible]	[illegible]	[illegible]	[illegible]	[illegible]	[illegible]	[illegible]	[illegible]	[illegible]	[illegible]	[illegible]	[illegible]	[illegible]	[illegible]	[illegible]	[illegible]	[illegible]	[illegible]	[illegible]	[illegible]	[illegible]	[illegible]	[illegible]	[illegible]	[illegible]	[illegible]	[illegible]	[illegible]	[illegible]	[illegible]	[illegible]	[illegible]	[illegible]	[illegible]	[illegible]	[illegible]	[illegible]	[illegible]	[illegible]	[illegible]	[illegible]	[illegible]	[illegible]	[illegible]	[illegible]
M00466: NblS-NblR_(photosynthesis)_two-component_regulatory_system	1.34E-06	[illegible]	[illegible]	[illegible]	[illegible]	[illegible]	[illegible]	[illegible]	[illegible]	[illegible]	[illegible]	[illegible]	[illegible]	[illegible]	[illegible]	[illegible]	[illegible]	[illegible]	[illegible]	[illegible]	[illegible]	[illegible]	[illegible]	[illegible]	[illegible]	[illegible]	[illegible]	[illegible]	[illegible]	[illegible]	[illegible]	[illegible]	[illegible]	[illegible]	[illegible]	[illegible]	[illegible]	[illegible]	[illegible]	[illegible]	[illegible]	[illegible]	[illegible]	[illegible]	[illegible]	[illegible]	[illegible]	[illegible]	[illegible]	[illegible]	[illegible]	[illegible]	[illegible]	[illegible]	[illegible]	[illegible]	[illegible]	[illegible]	[illegible]	[illegible]	[illegible]	[illegible]	[illegible]	[illegible]	[illegible]	[illegible]	[illegible]
M00467: SasA-RpaAB_(circadian_timing_mediating)_two-component_regulatory_system	1.47E-05	[illegible]	[illegible]	[illegible]	[illegible]	[illegible]	[illegible]	[illegible]	[illegible]	[illegible]	[illegible]	[illegible]	[illegible]	[illegible]	[illegible]	[illegible]	[illegible]	[illegible]	[illegible]	[illegible]	[illegible]	[illegible]	[illegible]	[illegible]	[illegible]	[illegible]	[illegible]	[illegible]	[illegible]	[illegible]	[illegible]	[illegible]	[illegible]	[illegible]	[illegible]	[illegible]	[illegible]	[illegible]	[illegible]	[illegible]	[illegible]	[illegible]	[illegible]	[illegible]	[illegible]	[illegible]	[illegible]	[illegible]	[illegible]	[illegible]	[illegible]	[illegible]	[illegible]	[illegible]	[illegible]	[illegible]	[illegible]	[illegible]	[illegible]	[illegible]	[illegible]	[illegible]	[illegible]	[illegible]	[illegible]	[illegible]	[illegible]
M00468: SaeS-SaeR_(staphylococcal_virulence_regulation)_two-component_regulatory_system	0	[illegible]	[illegible]	[illegible]	[illegible]	[illegible]	[illegible]	[illegible]	[illegible]	[illegible]	[illegible]	[illegible]	[illegible]	[illegible]	[illegible]	[illegible]	[illegible]	[illegible]	[illegible]	[illegible]	[illegible]	[illegible]	[illegible]	[illegible]	[illegible]	[illegible]	[illegible]	[illegible]	[illegible]	[illegible]	[illegible]	[illegible]	[illegible]	[illegible]	[illegible]	[illegible]	[illegible]	[illegible]	[illegible]	[illegible]	[illegible]	[illegible]	[illegible]	[illegible]	[illegible]	[illegible]	[illegible]	[illegible]	[illegible]	[illegible]	[illegible]	[illegible]	[illegible]	[illegible]	[illegible]	[illegible]	[illegible]	[illegible]	[illegible]	[illegible]	[illegible]	[illegible]	[illegible]	[illegible]	[illegible]	[illegible]	[illegible]
M00469: BceS-BceR_(bacitracin_transport)_two-component_regulatory_system	0	[illegible]	[illegible]	[illegible]	[illegible]	[illegible]	[illegible]	[illegible]	[illegible]	[illegible]	[illegible]	[illegible]	[illegible]	[illegible]	[illegible]	[illegible]	[illegible]	[illegible]	[illegible]	[illegible]	[illegible]	[illegible]	[illegible]	[illegible]	[illegible]	[illegible]	[illegible]	[illegible]	[illegible]	[illegible]	[illegible]	[illegible]	[illegible]	[illegible]	[illegible]	[illegible]	[illegible]	[illegible]	[illegible]	[illegible]	[illegible]	[illegible]	[illegible]	[illegible]	[illegible]	[illegible]	[illegible]	[illegible]	[illegible]	[illegible]	[illegible]	[illegible]	[illegible]	[illegible]	[illegible]	[illegible]	[illegible]	[illegible]	[illegible]	[illegible]	[illegible]	[illegible]	[illegible]	[illegible]	[illegible]	[illegible]	[illegible]
M00471: NarX-NarL_(nitrate_respiration)_two-component_regulatory_system	0.000377	[illegible]	[illegible]	[illegible]	[illegible]	[illegible]	[illegible]	[illegible]	[illegible]	[illegible]	[illegible]	[illegible]	[illegible]	[illegible]	[illegible]	[illegible]	[illegible]	[illegible]	[illegible]	[illegible]	[illegible]	[illegible]	[illegible]	[illegible]	[illegible]	[illegible]	[illegible]	[illegible]	[illegible]	[illegible]	[illegible]	[illegible]	[illegible]	[illegible]	[illegible]	[illegible]	[illegible]	[illegible]	[illegible]	[illegible]	[illegible]	[illegible]	[illegible]	[illegible]	[illegible]	[illegible]	[illegible]	[illegible]	[illegible]	[illegible]	[illegible]	[illegible]	[illegible]	[illegible]	[illegible]	[illegible]	[illegible]	[illegible]	[illegible]	[illegible]	[illegible]	[illegible]	[illegible]	[illegible]	[illegible]	[illegible]	[illegible]
M00472: NarQ-NarP_(nitrate_respiration)_two-component_regulatory_system	0	[illegible]	[illegible]	[illegible]	[illegible]	[illegible]	[illegible]	[illegible]	[illegible]	[illegible]	[illegible]	[illegible]	[illegible]	[illegible]	[illegible]	[illegible]	[illegible]	[illegible]	[illegible]	[illegible]	[illegible]	[illegible]	[illegible]	[illegible]	[illegible]	[illegible]	[illegible]	[illegible]	[illegible]	[illegible]	[illegible]	[illegible]	[illegible]	[illegible]	[illegible]	[illegible]	[illegible]	[illegible]	[illegible]	[illegible]	[illegible]	[illegible]	[illegible]	[illegible]	[illegible]	[illegible]	[illegible]	[illegible]	[illegible]	[illegible]	[illegible]	[illegible]	[illegible]	[illegible]	[illegible]	[illegible]	[illegible]	[illegible]	[illegible]	[illegible]	[illegible]	[illegible]	[illegible]	[illegible]	[illegible]	[illegible]	[illegible]
M00473: UhpB-UhpA_(hexose_phosphates_uptake)_two-component_regulatory_system	0.000473	[illegible]	[illegible]	[illegible]	[illegible]	[illegible]	[illegible]	[illegible]	[illegible]	[illegible]	[illegible]	[illegible]	[illegible]	[illegible]	[illegible]	[illegible]	[illegible]	[illegible]	[illegible]	[illegible]	[illegible]	[illegible]	[illegible]	[illegible]	[illegible]	[illegible]	[illegible]	[illegible]	[illegible]	[illegible]	[illegible]	[illegible]	[illegible]	[illegible]	[illegible]	[illegible]	[illegible]	[illegible]	[illegible]	[illegible]	[illegible]	[illegible]	[illegible]	[illegible]	[illegible]	[illegible]	[illegible]	[illegible]	[illegible]	[illegible]	[illegible]	[illegible]	[illegible]	[illegible]	[illegible]	[illegible]	[illegible]	[illegible]	[illegible]	[illegible]	[illegible]	[illegible]	[illegible]	[illegible]	[illegible]	[illegible]	[illegible]
M00474: RcsC-RcsD-RcsB_(capsule_synthesis)_two-component_regulatory_system	0.000179	[illegible]	[illegible]	[illegible]	[illegible]	[illegible]	[illegible]	[illegible]	[illegible]	[illegible]	[illegible]	[illegible]	[illegible]	[illegible]	[illegible]	[illegible]	[illegible]	[illegible]	[illegible]	[illegible]	[illegible]	[illegible]	[illegible]	[illegible]	[illegible]	[illegible]	[illegible]	[illegible]	[illegible]	[illegible]	[illegible]	[illegible]	[illegible]	[illegible]	[illegible]	[illegible]	[illegible]	[illegible]	[illegible]	[illegible]	[illegible]	[illegible]	[illegible]	[illegible]	[illegible]	[illegible]	[illegible]	[illegible]	[illegible]	[illegible]	[illegible]	[illegible]	[illegible]	[illegible]	[illegible]	[illegible]	[illegible]	[illegible]	[illegible]	[illegible]	[illegible]	[illegible]	[illegible]	[illegible]	[illegible]	[illegible]	[illegible]
M00475: BarA-UvrY_(central_carbon_metabolism)_two-component_regulatory_system	0.000208	[illegible]	[illegible]	[illegible]	[illegible]	[illegible]	[illegible]	[illegible]	[illegible]	[illegible]	[illegible]	[illegible]	[illegible]	[illegible]	[illegible]	[illegible]	[illegible]	[illegible]	[illegible]	[illegible]	[illegible]	[illegible]	[illegible]	[illegible]	[illegible]	[illegible]	[illegible]	[illegible]	[illegible]	[illegible]	[illegible]	[illegible]	[illegible]	[illegible]	[illegible]	[illegible]	[illegible]	[illegible]	[illegible]	[illegible]	[illegible]	[illegible]	[illegible]	[illegible]	[illegible]	[illegible]	[illegible]	[illegible]	[illegible]	[illegible]	[illegible]	[illegible]	[illegible]	[illegible]	[illegible]	[illegible]	[illegible]	[illegible]	[illegible]	[illegible]	[illegible]	[illegible]	[illegible]	[illegible]	[illegible]	[illegible]	[illegible]
M00476: ComP-ComA_(competence)_two-component_regulatory_system	0	[illegible]	[illegible]	[illegible]	[illegible]	[illegible]	[illegible]	[illegible]	[illegible]	[illegible]	[illegible]	[illegible]	[illegible]	[illegible]	[illegible]	[illegible]	[illegible]	[illegible]	[illegible]	[illegible]	[illegible]	[illegible]	[illegible]	[illegible]	[illegible]	[illegible]	[illegible]	[illegible]	[illegible]	[illegible]	[illegible]	[illegible]	[illegible]	[illegible]	[illegible]	[illegible]	[illegible]	[illegible]	[illegible]	[illegible]	[illegible]	[illegible]	[illegible]	[illegible]	[illegible]	[illegible]	[illegible]	[illegible]	[illegible]	[illegible]	[illegible]	[illegible]	[illegible]	[illegible]	[illegible]	[illegible]	[illegible]	[illegible]	[illegible]	[illegible]	[illegible]	[illegible]	[illegible]	[illegible]	[illegible]	[illegible]	[illegible]
M00477: EvgS-EvgA_(acid_and_drug_tolerance)_two-component_regulatory_system	8.95E-05	[illegible]	[illegible]	[illegible]	[illegible]	[illegible]	[illegible]	[illegible]	[illegible]	[illegible]	[illegible]	[illegible]	[illegible]	[illegible]	[illegible]	[illegible]	[illegible]	[illegible]	[illegible]	[illegible]	[illegible]	[illegible]	[illegible]	[illegible]	[illegible]	[illegible]	[illegible]	[illegible]	[illegible]	[illegible]	[illegible]	[illegible]	[illegible]	[illegible]	[illegible]	[illegible]	[illegible]	[illegible]	[illegible]	[illegible]	[illegible]	[illegible]	[illegible]	[illegible]	[illegible]	[illegible]	[illegible]	[illegible]	[illegible]	[illegible]	[illegible]	[illegible]	[illegible]	[illegible]	[illegible]	[illegible]	[illegible]	[illegible]	[illegible]	[illegible]	[illegible]	[illegible]	[illegible]	[illegible]	[illegible]	[illegible]	[illegible]
M00478: DegS-DegU_(multicellular_behavior_control)_two-component_regulatory_system	7.21E-05	[illegible]	[illegible]	[illegible]	[illegible]	[illegible]	[illegible]	[illegible]	[illegible]	[illegible]	[illegible]	[illegible]	[illegible]	[illegible]	[illegible]	[illegible]	[illegible]	[illegible]	[illegible]	[illegible]	[illegible]	[illegible]	[illegible]	[illegible]	[illegible]	[illegible]	[illegible]	[illegible]	[illegible]	[illegible]	[illegible]	[illegible]	[illegible]	[illegible]	[illegible]	[illegible]	[illegible]	[illegible]	[illegible]	[illegible]	[illegible]	[illegible]	[illegible]	[illegible]	[illegible]	[illegible]	[illegible]	[illegible]	[illegible]	[illegible]	[illegible]	[illegible]	[illegible]	[illegible]	[illegible]	[illegible]	[illegible]	[illegible]	[illegible]	[illegible]	[illegible]	[illegible]	[illegible]	[illegible]	[illegible]	[illegible]	[illegible]
M00479: DesK-DesR_(membrane_lipid_fluidity_regulation)_two-component_regulatory_system	0.001252	[illegible]	[illegible]	[illegible]	[illegible]	[illegible]	[illegible]	[illegible]	[illegible]	[illegible]	[illegible]	[illegible]	[illegible]	[illegible]	[illegible]	[illegible]	[illegible]	[illegible]	[illegible]	[illegible]	[illegible]	[illegible]	[illegible]	[illegible]	[illegible]	[illegible]	[illegible]	[illegible]	[illegible]	[illegible]	[illegible]	[illegible]	[illegible]	[illegible]	[illegible]	[illegible]	[illegible]	[illegible]	[illegible]	[illegible]	[illegible]	[illegible]	[illegible]	[illegible]	[illegible]	[illegible]	[illegible]	[illegible]	[illegible]	[illegible]	[illegible]	[illegible]	[illegible]	[illegible]	[illegible]	[illegible]	[illegible]	[illegible]	[illegible]	[illegible]	[illegible]	[illegible]	[illegible]	[illegible]	[illegible]	[illegible]	[illegible]
M00480: VraS-VraR_(cell-wall_peptidoglycan_synthesis)_two-component_regulatory_system	0	[illegible]	[illegible]	[illegible]	[illegible]	[illegible]	[illegible]	[illegible]	[illegible]	[illegible]	[illegible]	[illegible]	[illegible]	[illegible]	[illegible]	[illegible]	[illegible]	[illegible]	[illegible]	[illegible]	[illegible]	[illegible]	[illegible]	[illegible]	[illegible]	[illegible]	[illegible]	[illegible]	[illegible]	[illegible]	[illegible]	[illegible]	[illegible]	[illegible]	[illegible]	[illegible]	[illegible]	[illegible]	[illegible]	[illegible]	[illegible]	[illegible]	[illegible]	[illegible]	[illegible]	[illegible]	[illegible]	[illegible]	[illegible]	[illegible]	[illegible]	[illegible]	[illegible]	[illegible]	[illegible]	[illegible]	[illegible]	[illegible]	[illegible]	[illegible]	[illegible]	[illegible]	[illegible]	[illegible]	[illegible]	[illegible]	[illegible]
M00481: LiaS-LiaR_(cell_wall_stress_response)_two-component_regulatory_system	2.81E-05	[illegible]	[illegible]	[illegible]	[illegible]	[illegible]	[illegible]	[illegible]	[illegible]	[illegible]	[illegible]	[illegible]	[illegible]	[illegible]	[illegible]	[illegible]	[illegible]	[illegible]	[illegible]	[illegible]	[illegible]	[illegible]	[illegible]	[illegible]	[illegible]	[illegible]	[illegible]	[illegible]	[illegible]	[illegible]	[illegible]	[illegible]	[illegible]	[illegible]	[illegible]	[illegible]	[illegible]	[illegible]	[illegible]	[illegible]	[illegible]	[illegible]	[illegible]	[illegible]	[illegible]	[illegible]	[illegible]	[illegible]	[illegible]	[illegible]	[illegible]	[illegible]	[illegible]	[illegible]	[illegible]	[illegible]	[illegible]	[illegible]	[illegible]	[illegible]	[illegible]	[illegible]	[illegible]	[illegible]	[illegible]	[illegible]	[illegible]
M00482: DevS-DevR_(redox_response)_two-component_regulatory_system	0	[illegible]	[illegible]	[illegible]	[illegible]	[illegible]	[illegible]	[illegible]	[illegible]	[illegible]	[illegible]	[illegible]	[illegible]	[illegible]	[illegible]	[illegible]	[illegible]	[illegible]	[illegible]	[illegible]	[illegible]	[illegible]	[illegible]	[illegible]	[illegible]	[illegible]	[illegible]	[illegible]	[illegible]	[illegible]	[illegible]	[illegible]	[illegible]	[illegible]	[illegible]	[illegible]	[illegible]	[illegible]	[illegible]	[illegible]	[illegible]	[illegible]	[illegible]	[illegible]	[illegible]	[illegible]	[illegible]	[illegible]	[illegible]	[illegible]	[illegible]	[illegible]	[illegible]	[illegible]	[illegible]	[illegible]	[illegible]	[illegible]	[illegible]	[illegible]	[illegible]	[illegible]	[illegible]	[illegible]	[illegible]	[illegible]	[illegible]
M00483: NreB-NreC_(dissimilatory_nitrate/nitrite_reduction)_two-component_regulatory_system	2.81E-05	[illegible]	[illegible]	[illegible]	[illegible]	[illegible]	[illegible]	[illegible]	[illegible]	[illegible]	[illegible]	[illegible]	[illegible]	[illegible]	[illegible]	[illegible]	[illegible]	[illegible]	[illegible]	[illegible]	[illegible]	[illegible]	[illegible]	[illegible]	[illegible]	[illegible]	[illegible]	[illegible]	[illegible]	[illegible]	[illegible]	[illegible]	[illegible]	[illegible]	[illegible]	[illegible]	[illegible]	[illegible]	[illegible]	[illegible]	[illegible]	[illegible]	[illegible]	[illegible]	[illegible]	[illegible]	[illegible]	[illegible]	[illegible]	[illegible]	[illegible]	[illegible]	[illegible]	[illegible]	[illegible]	[illegible]	[illegible]	[illegible]	[illegible]	[illegible]	[illegible]	[illegible]	[illegible]	[illegible]	[illegible]	[illegible]	[illegible]
M00484: YdfH-YdfI_two-component_regulatory_system	0	[illegible]	[illegible]	[illegible]	[illegible]	[illegible]	[illegible]	[illegible]	[illegible]	[illegible]	[illegible]	[illegible]	[illegible]	[illegible]	[illegible]	[illegible]	[illegible]	[illegible]	[illegible]	[illegible]	[illegible]	[illegible]	[illegible]	[illegible]	[illegible]	[illegible]	[illegible]	[illegible]	[illegible]	[illegible]	[illegible]	[illegible]	[illegible]	[illegible]	[illegible]	[illegible]	[illegible]	[illegible]	[illegible]	[illegible]	[illegible]	[illegible]	[illegible]	[illegible]	[illegible]	[illegible]	[illegible]	[illegible]	[illegible]	[illegible]	[illegible]	[illegible]	[illegible]	[illegible]	[illegible]	[illegible]	[illegible]	[illegible]	[illegible]	[illegible]	[illegible]	[illegible]	[illegible]	[illegible]	[illegible]	[illegible]	[illegible]
M00485: KinABCDE-Spo0FA_(sporulation_control)_two-component_regulatory_system	2.04E-05	[illegible]	[illegible]	[illegible]	[illegible]	[illegible]	[illegible]	[illegible]	[illegible]	[illegible]	[illegible]	[illegible]	[illegible]	[illegible]	[illegible]	[illegible]	[illegible]	[illegible]	[illegible]	[illegible]	[illegible]	[illegible]	[illegible]	[illegible]	[illegible]	[illegible]	[illegible]	[illegible]	[illegible]	[illegible]	[illegible]	[illegible]	[illegible]	[illegible]	[illegible]	[illegible]	[illegible]	[illegible]	[illegible]	[illegible]	[illegible]	[illegible]	[illegible]	[illegible]	[illegible]	[illegible]	[illegible]	[illegible]	[illegible]	[illegible]	[illegible]	[illegible]	[illegible]	[illegible]	[illegible]	[illegible]	[illegible]	[illegible]	[illegible]	[illegible]	[illegible]	[illegible]	[illegible]	[illegible]	[illegible]	[illegible]	[illegible]
M00486: CitA-CitB_(citrate_fermentation)_two-component_regulatory_system	0	[illegible]	[illegible]	[illegible]	[illegible]	[illegible]	[illegible]	[illegible]	[illegible]	[illegible]	[illegible]	[illegible]	[illegible]	[illegible]	[illegible]	[illegible]	[illegible]	[illegible]	[illegible]	[illegible]	[illegible]	[illegible]	[illegible]	[illegible]	[illegible]	[illegible]	[illegible]	[illegible]	[illegible]	[illegible]	[illegible]	[illegible]	[illegible]	[illegible]	[illegible]	[illegible]	[illegible]	[illegible]	[illegible]	[illegible]	[illegible]	[illegible]	[illegible]	[illegible]	[illegible]	[illegible]	[illegible]	[illegible]	[illegible]	[illegible]	[illegible]	[illegible]	[illegible]	[illegible]	[illegible]	[illegible]	[illegible]	[illegible]	[illegible]	[illegible]	[illegible]	[illegible]	[illegible]	[illegible]	[illegible]	[illegible]	[illegible]
M00488: DcuS-DcuR_(C4-dicarboxylate_metabolism)_two-component_regulatory_system	0	[illegible]	[illegible]	[illegible]	[illegible]	[illegible]	[illegible]	[illegible]	[illegible]	[illegible]	[illegible]	[illegible]	[illegible]	[illegible]	[illegible]	[illegible]	[illegible]	[illegible]	[illegible]	[illegible]	[illegible]	[illegible]	[illegible]	[illegible]	[illegible]	[illegible]	[illegible]	[illegible]	[illegible]	[illegible]	[illegible]	[illegible]	[illegible]	[illegible]	[illegible]	[illegible]	[illegible]	[illegible]	[illegible]	[illegible]	[illegible]	[illegible]	[illegible]	[illegible]	[illegible]	[illegible]	[illegible]	[illegible]	[illegible]	[illegible]	[illegible]	[illegible]	[illegible]	[illegible]	[illegible]	[illegible]	[illegible]	[illegible]	[illegible]	[illegible]	[illegible]	[illegible]	[illegible]	[illegible]	[illegible]	[illegible]	[illegible]
M00489: DctS-DctR_(C4-dicarboxylate_transport)_two-component_regulatory_system	0	[illegible]	[illegible]	[illegible]	[illegible]	[illegible]	[illegible]	[illegible]	[illegible]	[illegible]	[illegible]	[illegible]	[illegible]	[illegible]	[illegible]	[illegible]	[illegible]	[illegible]	[illegible]	[illegible]	[illegible]	[illegible]	[illegible]	[illegible]	[illegible]	[illegible]	[illegible]	[illegible]	[illegible]	[illegible]	[illegible]	[illegible]	[illegible]	[illegible]	[illegible]	[illegible]	[illegible]	[illegible]	[illegible]	[illegible]	[illegible]	[illegible]	[illegible]	[illegible]	[illegible]	[illegible]	[illegible]	[illegible]	[illegible]	[illegible]	[illegible]	[illegible]	[illegible]	[illegible]	[illegible]	[illegible]	[illegible]	[illegible]	[illegible]	[illegible]	[illegible]	[illegible]	[illegible]	[illegible]	[illegible]	[illegible]	[illegible]
M00490: MalK-MalR_(malate_transport)_two-component_regulatory_system	0	[illegible]	[illegible]	[illegible]	[illegible]	[illegible]	[illegible]	[illegible]	[illegible]	[illegible]	[illegible]	[illegible]	[illegible]	[illegible]	[illegible]	[illegible]	[illegible]	[illegible]	[illegible]	[illegible]	[illegible]	[illegible]	[illegible]	[illegible]	[illegible]	[illegible]	[illegible]	[illegible]	[illegible]	[illegible]	[illegible]	[illegible]	[illegible]	[illegible]	[illegible]	[illegible]	[illegible]	[illegible]	[illegible]	[illegible]	[illegible]	[illegible]	[illegible]	[illegible]	[illegible]	[illegible]	[illegible]	[illegible]	[illegible]	[illegible]	[illegible]	[illegible]	[illegible]	[illegible]	[illegible]	[illegible]	[illegible]	[illegible]	[illegible]	[illegible]	[illegible]	[illegible]	[illegible]	[illegible]	[illegible]	[illegible]	[illegible]
M00491: arabinogalactan_oligomer/maltooligosaccharide_transport_system	0.000321	[illegible]	[illegible]	[illegible]	[illegible]	[illegible]	[illegible]	[illegible]	[illegible]	[illegible]	[illegible]	[illegible]	[illegible]	[illegible]	[illegible]	[illegible]	[illegible]	[illegible]	[illegible]	[illegible]	[illegible]	[illegible]	[illegible]	[illegible]	[illegible]	[illegible]	[illegible]	[illegible]	[illegible]	[illegible]	[illegible]	[illegible]	[illegible]	[illegible]	[illegible]	[illegible]	[illegible]	[illegible]	[illegible]	[illegible]	[illegible]	[illegible]	[illegible]	[illegible]	[illegible]	[illegible]	[illegible]	[illegible]	[illegible]	[illegible]	[illegible]	[illegible]	[illegible]	[illegible]	[illegible]	[illegible]	[illegible]	[illegible]	[illegible]	[illegible]	[illegible]	[illegible]	[illegible]	[illegible]	[illegible]	[illegible]	[illegible]
M00492: LytS-LytR_two-component_regulatory_system	0.000749	[illegible]	[illegible]	[illegible]	[illegible]	[illegible]	[illegible]	[illegible]	[illegible]	[illegible]	[illegible]	[illegible]	[illegible]	[illegible]	[illegible]	[illegible]	[illegible]	[illegible]	[illegible]	[illegible]	[illegible]	[illegible]	[illegible]	[illegible]	[illegible]	[illegible]	[illegible]	[illegible]	[illegible]	[illegible]	[illegible]	[illegible]	[illegible]	[illegible]	[illegible]	[illegible]	[illegible]	[illegible]	[illegible]	[illegible]	[illegible]	[illegible]	[illegible]	[illegible]	[illegible]	[illegible]	[illegible]	[illegible]	[illegible]	[illegible]	[illegible]	[illegible]	[illegible]	[illegible]	[illegible]	[illegible]	[illegible]	[illegible]	[illegible]	[illegible]	[illegible]	[illegible]	[illegible]	[illegible]	[illegible]	[illegible]	[illegible]
M00493: AlgZ-AlgR_(alginate_production)_two-component_regulatory_system	0.000692	[illegible]	[illegible]	[illegible]	[illegible]	[illegible]	[illegible]	[illegible]	[illegible]	[illegible]	[illegible]	[illegible]	[illegible]	[illegible]	[illegible]	[illegible]	[illegible]	[illegible]	[illegible]	[illegible]	[illegible]	[illegible]	[illegible]	[illegible]	[illegible]	[illegible]	[illegible]	[illegible]	[illegible]	[illegible]	[illegible]	[illegible]	[illegible]	[illegible]	[illegible]	[illegible]	[illegible]	[illegible]	[illegible]	[illegible]	[illegible]	[illegible]	[illegible]	[illegible]	[illegible]	[illegible]	[illegible]	[illegible]	[illegible]	[illegible]	[illegible]	[illegible]	[illegible]	[illegible]	[illegible]	[illegible]	[illegible]	[illegible]	[illegible]	[illegible]	[illegible]	[illegible]	[illegible]	[illegible]	[illegible]	[illegible]	[illegible]
M00495: AgrC-AgrA_(exoprotein_synthesis)_two-component_regulatory_system	0	[illegible]	[illegible]	[illegible]	[illegible]	[illegible]	[illegible]	[illegible]	[illegible]	[illegible]	[illegible]	[illegible]	[illegible]	[illegible]	[illegible]	[illegible]	[illegible]	[illegible]	[illegible]	[illegible]	[illegible]	[illegible]	[illegible]	[illegible]	[illegible]	[illegible]	[illegible]	[illegible]	[illegible]	[illegible]	[illegible]	[illegible]	[illegible]	[illegible]	[illegible]	[illegible]	[illegible]	[illegible]	[illegible]	[illegible]	[illegible]	[illegible]	[illegible]	[illegible]	[illegible]	[illegible]	[illegible]	[illegible]	[illegible]	[illegible]	[illegible]	[illegible]	[illegible]	[illegible]	[illegible]	[illegible]	[illegible]	[illegible]	[illegible]	[illegible]	[illegible]	[illegible]	[illegible]	[illegible]	[illegible]	[illegible]	[illegible]
M00497: GlnL-GlnG_(nitrogen_regulation)_two-component_regulatory_system	0.000903	[illegible]	[illegible]	[illegible]	[illegible]	[illegible]	[illegible]	[illegible]	[illegible]	[illegible]	[illegible]	[illegible]	[illegible]	[illegible]	[illegible]	[illegible]	[illegible]	[illegible]	[illegible]	[illegible]	[illegible]	[illegible]	[illegible]	[illegible]	[illegible]	[illegible]	[illegible]	[illegible]	[illegible]	[illegible]	[illegible]	[illegible]	[illegible]	[illegible]	[illegible]	[illegible]	[illegible]	[illegible]	[illegible]	[illegible]	[illegible]	[illegible]	[illegible]	[illegible]	[illegible]	[illegible]	[illegible]	[illegible]	[illegible]	[illegible]	[illegible]	[illegible]	[illegible]	[illegible]	[illegible]	[illegible]	[illegible]	[illegible]	[illegible]	[illegible]	[illegible]	[illegible]	[illegible]	[illegible]	[illegible]	[illegible]	[illegible]
M00498: NtrY-NtrX_(nitrogen_regulation)_two-component_regulatory_system	0.001066	[illegible]	[illegible]	[illegible]	[illegible]	[illegible]	[illegible]	[illegible]	[illegible]	[illegible]	[illegible]	[illegible]	[illegible]	[illegible]	[illegible]	[illegible]	[illegible]	[illegible]	[illegible]	[illegible]	[illegible]	[illegible]	[illegible]	[illegible]	[illegible]	[illegible]	[illegible]	[illegible]	[illegible]	[illegible]	[illegible]	[illegible]	[illegible]	[illegible]	[illegible]	[illegible]	[illegible]	[illegible]	[illegible]	[illegible]	[illegible]	[illegible]	[illegible]	[illegible]	[illegible]	[illegible]	[illegible]	[illegible]	[illegible]	[illegible]	[illegible]	[illegible]	[illegible]	[illegible]	[illegible]	[illegible]	[illegible]	[illegible]	[illegible]	[illegible]	[illegible]	[illegible]	[illegible]	[illegible]	[illegible]	[illegible]	[illegible]
M00499: HydH-HydG_(metal_tolerance)_two-component_regulatory_system	0.001109	[illegible]	[illegible]	[illegible]	[illegible]	[illegible]	[illegible]	[illegible]	[illegible]	[illegible]	[illegible]	[illegible]	[illegible]	[illegible]	[illegible]	[illegible]	[illegible]	[illegible]	[illegible]	[illegible]	[illegible]	[illegible]	[illegible]	[illegible]	[illegible]	[illegible]	[illegible]	[illegible]	[illegible]	[illegible]	[illegible]	[illegible]	[illegible]	[illegible]	[illegible]	[illegible]	[illegible]	[illegible]	[illegible]	[illegible]	[illegible]	[illegible]	[illegible]	[illegible]	[illegible]	[illegible]	[illegible]	[illegible]	[illegible]	[illegible]	[illegible]	[illegible]	[illegible]	[illegible]	[illegible]	[illegible]	[illegible]	[illegible]	[illegible]	[illegible]	[illegible]	[illegible]	[illegible]	[illegible]	[illegible]	[illegible]	[illegible]
M00500: AtoS-AtoC_(cPHB_biosynthesis)_two-component_regulatory_system	0.001019	[illegible]	[illegible]	[illegible]	[illegible]	[illegible]	[illegible]	[illegible]	[illegible]	[illegible]	[illegible]	[illegible]	[illegible]	[illegible]	[illegible]	[illegible]	[illegible]	[illegible]	[illegible]	[illegible]	[illegible]	[illegible]	[illegible]	[illegible]	[illegible]	[illegible]	[illegible]	[illegible]	[illegible]	[illegible]	[illegible]	[illegible]	[illegible]	[illegible]	[illegible]	[illegible]	[illegible]	[illegible]	[illegible]	[illegible]	[illegible]	[illegible]	[illegible]	[illegible]	[illegible]	[illegible]	[illegible]	[illegible]	[illegible]	[illegible]	[illegible]	[illegible]	[illegible]	[illegible]	[illegible]	[illegible]	[illegible]	[illegible]	[illegible]	[illegible]	[illegible]	[illegible]	[illegible]	[illegible]	[illegible]	[illegible]	[illegible]
M00501: PilS-PilR_(type_4_fimbriae_synthesis)_two-component_regulatory_system	0.000728	[illegible]	[illegible]	[illegible]	[illegible]	[illegible]	[illegible]	[illegible]	[illegible]	[illegible]	[illegible]	[illegible]	[illegible]	[illegible]	[illegible]	[illegible]	[illegible]	[illegible]	[illegible]	[illegible]	[illegible]	[illegible]	[illegible]	[illegible]	[illegible]	[illegible]	[illegible]	[illegible]	[illegible]	[illegible]	[illegible]	[illegible]	[illegible]	[illegible]	[illegible]	[illegible]	[illegible]	[illegible]	[illegible]	[illegible]	[illegible]	[illegible]	[illegible]	[illegible]	[illegible]	[illegible]	[illegible]	[illegible]	[illegible]	[illegible]	[illegible]	[illegible]	[illegible]	[illegible]	[illegible]	[illegible]	[illegible]	[illegible]	[illegible]	[illegible]	[illegible]	[illegible]	[illegible]	[illegible]	[illegible]	[illegible]	[illegible]
M00502: GlrK-GlrR_(amino_sugar_metabolism)_two-component_regulatory_system	0.000297	[illegible]	[illegible]	[illegible]	[illegible]	[illegible]	[illegible]	[illegible]	[illegible]	[illegible]	[illegible]	[illegible]	[illegible]	[illegible]	[illegible]	[illegible]	[illegible]	[illegible]	[illegible]	[illegible]	[illegible]	[illegible]	[illegible]	[illegible]	[illegible]	[illegible]	[illegible]	[illegible]	[illegible]	[illegible]	[illegible]	[illegible]	[illegible]	[illegible]	[illegible]	[illegible]	[illegible]	[illegible]	[illegible]	[illegible]	[illegible]	[illegible]	[illegible]	[illegible]	[illegible]	[illegible]	[illegible]	[illegible]	[illegible]	[illegible]	[illegible]	[illegible]	[illegible]	[illegible]	[illegible]	[illegible]	[illegible]	[illegible]	[illegible]	[illegible]	[illegible]	[illegible]	[illegible]	[illegible]	[illegible]	[illegible]	[illegible]
M00504: DctB-DctD_(C4-dicarboxylate_transport)_two-component_regulatory_system	8.15E-05	[illegible]	[illegible]	[illegible]	[illegible]	[illegible]	[illegible]	[illegible]	[illegible]	[illegible]	[illegible]	[illegible]	[illegible]	[illegible]	[illegible]	[illegible]	[illegible]	[illegible]	[illegible]	[illegible]	[illegible]	[illegible]	[illegible]	[illegible]	[illegible]	[illegible]	[illegible]	[illegible]	[illegible]	[illegible]	[illegible]	[illegible]	[illegible]	[illegible]	[illegible]	[illegible]	[illegible]	[illegible]	[illegible]	[illegible]	[illegible]	[illegible]	[illegible]	[illegible]	[illegible]	[illegible]	[illegible]	[illegible]	[illegible]	[illegible]	[illegible]	[illegible]	[illegible]	[illegible]	[illegible]	[illegible]	[illegible]	[illegible]	[illegible]	[illegible]	[illegible]	[illegible]	[illegible]	[illegible]	[illegible]	[illegible]	[illegible]
M00505: KinB-AlgB_(alginate_production)_two-component_regulatory_system	8.01E-06	[illegible]	[illegible]	[illegible]	[illegible]	[illegible]	[illegible]	[illegible]	[illegible]	[illegible]	[illegible]	[illegible]	[illegible]	[illegible]	[illegible]	[illegible]	[illegible]	[illegible]	[illegible]	[illegible]	[illegible]	[illegible]	[illegible]	[illegible]	[illegible]	[illegible]	[illegible]	[illegible]	[illegible]	[illegible]	[illegible]	[illegible]	[illegible]	[illegible]	[illegible]	[illegible]	[illegible]	[illegible]	[illegible]	[illegible]	[illegible]	[illegible]	[illegible]	[illegible]	[illegible]	[illegible]	[illegible]	[illegible]	[illegible]	[illegible]	[illegible]	[illegible]	[illegible]	[illegible]	[illegible]	[illegible]	[illegible]	[illegible]	[illegible]	[illegible]	[illegible]	[illegible]	[illegible]	[illegible]	[illegible]	[illegible]	[illegible]
M00506: CheA-CheYBV_(chemotaxis)_two-component_regulatory_system	0.002358	[illegible]	[illegible]	[illegible]	[illegible]	[illegible]	[illegible]	[illegible]	[illegible]	[illegible]	[illegible]	[illegible]	[illegible]	[illegible]	[illegible]	[illegible]	[illegible]	[illegible]	[illegible]	[illegible]	[illegible]	[illegible]	[illegible]	[illegible]	[illegible]	[illegible]	[illegible]	[illegible]	[illegible]	[illegible]	[illegible]	[illegible]	[illegible]	[illegible]	[illegible]	[illegible]	[illegible]	[illegible]	[illegible]	[illegible]	[illegible]	[illegible]	[illegible]	[illegible]	[illegible]	[illegible]	[illegible]	[illegible]	[illegible]	[illegible]	[illegible]	[illegible]	[illegible]	[illegible]	[illegible]	[illegible]	[illegible]	[illegible]	[illegible]	[illegible]	[illegible]	[illegible]	[illegible]	[illegible]	[illegible]	[illegible]	[illegible]
M00507: ChpA-ChpB/PilGH_(chemosensory)_two-component_regulatory_system	0.000653	[illegible]	[illegible]	[illegible]	[illegible]	[illegible]	[illegible]	[illegible]	[illegible]	[illegible]	[illegible]	[illegible]	[illegible]	[illegible]	[illegible]	[illegible]	[illegible]	[illegible]	[illegible]	[illegible]	[illegible]	[illegible]	[illegible]	[illegible]	[illegible]	[illegible]	[illegible]	[illegible]	[illegible]	[illegible]	[illegible]	[illegible]	[illegible]	[illegible]	[illegible]	[illegible]	[illegible]	[illegible]	[illegible]	[illegible]	[illegible]	[illegible]	[illegible]	[illegible]	[illegible]	[illegible]	[illegible]	[illegible]	[illegible]	[illegible]	[illegible]	[illegible]	[illegible]	[illegible]	[illegible]	[illegible]	[illegible]	[illegible]	[illegible]	[illegible]	[illegible]	[illegible]	[illegible]	[illegible]	[illegible]	[illegible]	[illegible]
M00508: PixL-PixGH_(positive_phototaxis)_two-component_regulatory_system	6.68E-07	[illegible]	[illegible]	[illegible]	[illegible]	[illegible]	[illegible]	[illegible]	[illegible]	[illegible]	[illegible]	[illegible]	[illegible]	[illegible]	[illegible]	[illegible]	[illegible]	[illegible]	[illegible]	[illegible]	[illegible]	[illegible]	[illegible]	[illegible]	[illegible]	[illegible]	[illegible]	[illegible]	[illegible]	[illegible]	[illegible]	[illegible]	[illegible]	[illegible]	[illegible]	[illegible]	[illegible]	[illegible]	[illegible]	[illegible]	[illegible]	[illegible]	[illegible]	[illegible]	[illegible]	[illegible]	[illegible]	[illegible]	[illegible]	[illegible]	[illegible]	[illegible]	[illegible]	[illegible]	[illegible]	[illegible]	[illegible]	[illegible]	[illegible]	[illegible]	[illegible]	[illegible]	[illegible]	[illegible]	[illegible]	[illegible]	[illegible]
M00509: WspE-WspRF_(chemosensory)_two-component_regulatory_system	1.34E-05	[illegible]	[illegible]	[illegible]	[illegible]	[illegible]	[illegible]	[illegible]	[illegible]	[illegible]	[illegible]	[illegible]	[illegible]	[illegible]	[illegible]	[illegible]	[illegible]	[illegible]	[illegible]	[illegible]	[illegible]	[illegible]	[illegible]	[illegible]	[illegible]	[illegible]	[illegible]	[illegible]	[illegible]	[illegible]	[illegible]	[illegible]	[illegible]	[illegible]	[illegible]	[illegible]	[illegible]	[illegible]	[illegible]	[illegible]	[illegible]	[illegible]	[illegible]	[illegible]	[illegible]	[illegible]	[illegible]	[illegible]	[illegible]	[illegible]	[illegible]	[illegible]	[illegible]	[illegible]	[illegible]	[illegible]	[illegible]	[illegible]	[illegible]	[illegible]	[illegible]	[illegible]	[illegible]	[illegible]	[illegible]	[illegible]	[illegible]
M00510: Cph1-Rcp1_(light_response)_two-component_regulatory_system	1.34E-06	[illegible]	[illegible]	[illegible]	[illegible]	[illegible]	[illegible]	[illegible]	[illegible]	[illegible]	[illegible]	[illegible]	[illegible]	[illegible]	[illegible]	[illegible]	[illegible]	[illegible]	[illegible]	[illegible]	[illegible]	[illegible]	[illegible]	[illegible]	[illegible]	[illegible]	[illegible]	[illegible]	[illegible]	[illegible]	[illegible]	[illegible]	[illegible]	[illegible]	[illegible]	[illegible]	[illegible]	[illegible]	[illegible]	[illegible]	[illegible]	[illegible]	[illegible]	[illegible]	[illegible]	[illegible]	[illegible]	[illegible]	[illegible]	[illegible]	[illegible]	[illegible]	[illegible]	[illegible]	[illegible]	[illegible]	[illegible]	[illegible]	[illegible]	[illegible]	[illegible]	[illegible]	[illegible]	[illegible]	[illegible]	[illegible]	[illegible]
M00511: PleC-PleD_(cell_fate_control)_two-component_regulatory_system	0.000197	[illegible]	[illegible]	[illegible]	[illegible]	[illegible]	[illegible]	[illegible]	[illegible]	[illegible]	[illegible]	[illegible]	[illegible]	[illegible]	[illegible]	[illegible]	[illegible]	[illegible]	[illegible]	[illegible]	[illegible]	[illegible]	[illegible]	[illegible]	[illegible]	[illegible]	[illegible]	[illegible]	[illegible]	[illegible]	[illegible]	[illegible]	[illegible]	[illegible]	[illegible]	[illegible]	[illegible]	[illegible]	[illegible]	[illegible]	[illegible]	[illegible]	[illegible]	[illegible]	[illegible]	[illegible]	[illegible]	[illegible]	[illegible]	[illegible]	[illegible]	[illegible]	[illegible]	[illegible]	[illegible]	[illegible]	[illegible]	[illegible]	[illegible]	[illegible]	[illegible]	[illegible]	[illegible]	[illegible]	[illegible]	[illegible]	[illegible]
M00512: CckA-CtrA/CpdR_(cell_cycle_control)_two-component_regulatory_system	0.00034	[illegible]	[illegible]	[illegible]	[illegible]	[illegible]	[illegible]	[illegible]	[illegible]	[illegible]	[illegible]	[illegible]	[illegible]	[illegible]	[illegible]	[illegible]	[illegible]	[illegible]	[illegible]	[illegible]	[illegible]	[illegible]	[illegible]	[illegible]	[illegible]	[illegible]	[illegible]	[illegible]	[illegible]	[illegible]	[illegible]	[illegible]	[illegible]	[illegible]	[illegible]	[illegible]	[illegible]	[illegible]	[illegible]	[illegible]	[illegible]	[illegible]	[illegible]	[illegible]	[illegible]	[illegible]	[illegible]	[illegible]	[illegible]	[illegible]	[illegible]	[illegible]	[illegible]	[illegible]	[illegible]	[illegible]	[illegible]	[illegible]	[illegible]	[illegible]	[illegible]	[illegible]	[illegible]	[illegible]	[illegible]	[illegible]	[illegible]
M00513: LuxQN/CqsS-LuxU-LuxO_(quorum_sensing)_two-component_regulatory_system	3.87E-05	[illegible]	[illegible]	[illegible]	[illegible]	[illegible]	[illegible]	[illegible]	[illegible]	[illegible]	[illegible]	[illegible]	[illegible]	[illegible]	[illegible]	[illegible]	[illegible]	[illegible]	[illegible]	[illegible]	[illegible]	[illegible]	[illegible]	[illegible]	[illegible]	[illegible]	[illegible]	[illegible]	[illegible]	[illegible]	[illegible]	[illegible]	[illegible]	[illegible]	[illegible]	[illegible]	[illegible]	[illegible]	[illegible]	[illegible]	[illegible]	[illegible]	[illegible]	[illegible]	[illegible]	[illegible]	[illegible]	[illegible]	[illegible]	[illegible]	[illegible]	[illegible]	[illegible]	[illegible]	[illegible]	[illegible]	[illegible]	[illegible]	[illegible]	[illegible]	[illegible]	[illegible]	[illegible]	[illegible]	[illegible]	[illegible]	[illegible]
M00514: TtrS-TtrR_(tetrathionate_respiration)_two-component_regulatory_system	4.01E-06	[illegible]	[illegible]	[illegible]	[illegible]	[illegible]	[illegible]	[illegible]	[illegible]	[illegible]	[illegible]	[illegible]	[illegible]	[illegible]	[illegible]	[illegible]	[illegible]	[illegible]	[illegible]	[illegible]	[illegible]	[illegible]	[illegible]	[illegible]	[illegible]	[illegible]	[illegible]	[illegible]	[illegible]	[illegible]	[illegible]	[illegible]	[illegible]	[illegible]	[illegible]	[illegible]	[illegible]	[illegible]	[illegible]	[illegible]	[illegible]	[illegible]	[illegible]	[illegible]	[illegible]	[illegible]	[illegible]	[illegible]	[illegible]	[illegible]	[illegible]	[illegible]	[illegible]	[illegible]	[illegible]	[illegible]	[illegible]	[illegible]	[illegible]	[illegible]	[illegible]	[illegible]	[illegible]	[illegible]	[illegible]	[illegible]	[illegible]
M00515: FlrB-FlrC_(polar_flagellar_synthesis)_two-component_regulatory_system	0.000349	[illegible]	[illegible]	[illegible]	[illegible]	[illegible]	[illegible]	[illegible]	[illegible]	[illegible]	[illegible]	[illegible]	[illegible]	[illegible]	[illegible]	[illegible]	[illegible]	[illegible]	[illegible]	[illegible]	[illegible]	[illegible]	[illegible]	[illegible]	[illegible]	[illegible]	[illegible]	[illegible]	[illegible]	[illegible]	[illegible]	[illegible]	[illegible]	[illegible]	[illegible]	[illegible]	[illegible]	[illegible]	[illegible]	[illegible]	[illegible]	[illegible]	[illegible]	[illegible]	[illegible]	[illegible]	[illegible]	[illegible]	[illegible]	[illegible]	[illegible]	[illegible]	[illegible]	[illegible]	[illegible]	[illegible]	[illegible]	[illegible]	[illegible]	[illegible]	[illegible]	[illegible]	[illegible]	[illegible]	[illegible]	[illegible]	[illegible]

Table S4. IV

KEGG_Module	Habitat																																																																One-class test (strict, p < 0.01)	p value
	India oil refineries				Arctic												China oil refineries									Mangrove			Marine sediments							Taiga upper active layer				Taiga bottom active layer				Taiga permafrost layer				Oil sands core							Oil sands tailings pond upper				Oil sands tailings pond median			Oil sands tailings pond deep					
	I1	I2	I3	I4	A1	A10	A11	A12	A2	A3	A4	A5	A6	A7	A8	A9	C1	C2	C3	C4	C5	C6	C7	C8	C9	M1	M2	M3	DWH1	DWH2	DWH3	DWH4	DWH5	DWH6	DWH7	Tu1	Tu2	Tu3	Tu4	Tb1	Tb2	Tb3	Tb4	Tp1	Tp2	Tp3	Tp4	OSC1	OSC12	OSC3	OSC4	OSC5	OSC7	OSC9	OSTPu1	OSTPu2	OSTPu4	OSTPu3	OSTPm2	OSTPm6	OSTPm4	OSTPd1	OSTPd2	OSTPd3	OSTPd4		
M00517: RpfC-RpfG_(cell-to-cell_signaling)_two-component_regulatory_system	0.000305	0.000121	0.000108	0.000353	0.000102	0.000162	0.000232	5.46E-05	5.43E-05	6.25E-05	4.44E-05	0.000162	4.55E-05	0.000157	1.58E-05	0.000225	2.11E-05	2.79E-05	2.84E-05	3.32E-05	3.85E-05	6.70E-05	1.56E-05	3.23E-05	2.59E-05	0.000108	0.000165	0.000124	0.00047	0.00039	0.000366	0.000424	0.000362	0.000166	0.000456	1.24E-05	0	3.17E-06	3.12E-06	3.66E-06	5.03E-06	0	0	8.45E-06	0	3.28E-06	1.28E-05	1.09E-05	1.30E-05	3.39E-06	3.62E-06	0	1.61E-05	7.20E-06	4.91E-05	0.00011	1.22E-05	3.91E-05	5.80E-05	3.85E-06	9.21E-06	1.86E-05	8.97E-05	2.13E-05	0	Marine sediments	2.13E-07
M00518: GlnK-GlnL_(glutamine_utilization)_two-component_regulatory_system	0	0	0	0	0	0	0	0	0	0	0	0	0	0	0	0	0	3.10E-06	1.58E-06	1.51E-06	0	1.63E-06	6.24E-06	0	1.62E-06	0	0	0	0	0	0	0	0	0	0	0	0	0	1.56E-06	0	1.68E-06	1.62E-06	0	0	1.74E-06	0	3.66E-06	0	0	1.69E-06	0	0	0	0	1.82E-06	0	1.02E-05	4.12E-06	0	1.54E-05	0	0	0	0	4.18E-05		
M00519: YesM-YesN_two-component_regulatory_system	1.34E-06	6.41E-05	6.75E-05	6.46E-06	2.78E-05	1.08E-05	3.68E-06	6.18E-05	5.43E-05	7.71E-05	5.68E-05	6.61E-06	5.59E-05	4.35E-05	3.80E-05	3.12E-06	0.000185	0.000158	0.000186	0.000211	0.000188	0.000139	0.000186	0.000132	0.000169	0.000524	0.000307	0.000375	0.000233	0.000113	0.00014	8.33E-05	9.40E-05	2.65E-05	7.82E-05	0.000109	2.70E-05	0.000122	9.68E-05	1.83E-05	6.54E-05	0.00012	6.85E-06	6.08E-05	2.61E-05	0.000141	6.58E-05	1.09E-05	2.60E-05	7.29E-05	9.06E-06	5.33E-06	1.61E-05	7.20E-06	9.64E-05	0.000108	0.000696	0.000478	0.000159	0.000318	0.000155	8.91E-05	3.82E-05	4.44E-05	0.000426	Mangrove	5.32E-06
M00520: ChvG-ChvI_(acidity_sensing)_two-component_regulatory_system	0.000387	0.000167	7.84E-05	0.000203	0.000215	0.000314	0.000375	0.000391	0.000265	0.000263	0.000332	0.000278	0.000213	0.000192	0.000276	0.000253	0.000174	0.000116	8.05E-05	0.000145	0.000167	0.000142	0.00012	0.000129	0.000115	0.000137	0.000197	0.000104	0.00017	0.000357	0.000172	0.000144	0.000142	0.000509	0.00028	0.001221	0.001467	0.000149	0.001552	0.001358	0.001437	0.000506	0.001558	0.00139	0.001781	2.79E-05	0.001491	0.000566	0.000704	0.000298	0.0006	0.000567	0.000553	0.000517	4.55E-05	1.51E-05	2.24E-05	4.32E-05	3.10E-05	8.66E-05	2.76E-05	3.16E-05	1.72E-05	0.000311	0.000159	Taiga bottom active layer	9.97E-06
M00521: CiaH-CiaR_two-component_regulatory_system	0	0	0	0	0	0	1.84E-06	0	0	0	0	0	0	0	0	0	7.03E-06	3.10E-06	3.16E-06	3.02E-06	5.71E-06	1.63E-06	1.56E-06	2.70E-06	4.87E-06	0	0	0	0	0	0	0	0	0	0	0	0	0	0	0	0	0	0	0	0	0	0	0	0	3.39E-06	3.62E-06	0	1.79E-06	0	0	0	1.22E-05	2.06E-06	0	5.78E-06	0	0	0	1.93E-06	1.99E-06		
M00522: SalK-SalR_two-component_regulatory_system	0	0	0	0	0	0	0	0	0	0	0	0	0	0	0	0	0	1.55E-06	0	1.51E-06	1.43E-06	0	0	0	0	2.45E-05	2.34E-05	1.30E-05	0	0	0	0	0	0	0	0	0	3.17E-06	0	0	0	0	0	1.69E-06	0	0	0	0	0	0	0	0	0	0	0	3.78E-06	0.000134	2.06E-06	0	0	1.84E-06	0	0	0	0		
M00523: RegB-RegA_(redox_response)_two-component_regulatory_system	0.001229	0.000873	0.000854	0.001083	0.000741	0.00069	[illegible]	[illegible]	[illegible]	[illegible]	[illegible]	[illegible]	[illegible]	[illegible]	[illegible]	[illegible]	[illegible]	[illegible]	[illegible]	[illegible]	[illegible]	[illegible]	[illegible]	[illegible]	[illegible]	[illegible]	[illegible]	[illegible]	[illegible]	[illegible]	[illegible]	[illegible]	[illegible]	[illegible]	[illegible]	[illegible]	[illegible]	[illegible]	[illegible]	[illegible]	[illegible]	[illegible]	[illegible]	[illegible]	[illegible]	[illegible]	[illegible]	[illegible]	[illegible]	[illegible]	[illegible]	[illegible]	[illegible]	[illegible]	[illegible]	[illegible]	[illegible]	[illegible]	[illegible]	[illegible]	[illegible]	[illegible]	[illegible]	[illegible]	[illegible]	[illegible]	[illegible]
M00524: FixL-FixJ_(nitrogen_fixation)_two-component_regulatory_system	0.000186	3.90E-05	8.76E-05	0.00015	0.000408	0.000348	[illegible]	[illegible]	[illegible]	[illegible]	[illegible]	[illegible]	[illegible]	[illegible]	[illegible]	[illegible]	[illegible]	[illegible]	[illegible]	[illegible]	[illegible]	[illegible]	[illegible]	[illegible]	[illegible]	[illegible]	[illegible]	[illegible]	[illegible]	[illegible]	[illegible]	[illegible]	[illegible]	[illegible]	[illegible]	[illegible]	[illegible]	[illegible]	[illegible]	[illegible]	[illegible]	[illegible]	[illegible]	[illegible]	[illegible]	[illegible]	[illegible]	[illegible]	[illegible]	[illegible]	[illegible]	[illegible]	[illegible]	[illegible]	[illegible]	[illegible]	[illegible]	[illegible]	[illegible]	[illegible]	[illegible]	[illegible]	[illegible]	[illegible]	[illegible]	[illegible]	[illegible]
M00525: Lysine_biosynthesis,_acetyl-DAP_pathway,_aspartate_=>_lysine	0.002345	0.002066	0.002166	0.002105	0.002108	0.002299	[illegible]	[illegible]	[illegible]	[illegible]	[illegible]	[illegible]	[illegible]	[illegible]	[illegible]	[illegible]	[illegible]	[illegible]	[illegible]	[illegible]	[illegible]	[illegible]	[illegible]	[illegible]	[illegible]	[illegible]	[illegible]	[illegible]	[illegible]	[illegible]	[illegible]	[illegible]	[illegible]	[illegible]	[illegible]	[illegible]	[illegible]	[illegible]	[illegible]	[illegible]	[illegible]	[illegible]	[illegible]	[illegible]	[illegible]	[illegible]	[illegible]	[illegible]	[illegible]	[illegible]	[illegible]	[illegible]	[illegible]	[illegible]	[illegible]	[illegible]	[illegible]	[illegible]	[illegible]	[illegible]	[illegible]	[illegible]	[illegible]	[illegible]	[illegible]	[illegible]	[illegible]
M00526: Lysine_biosynthesis,_DAP_dehydrogenase_pathway,_aspartate_=>_lysine	[illegible]	[illegible]	[illegible]	[illegible]	[illegible]	[illegible]	[illegible]	[illegible]	[illegible]	[illegible]	[illegible]	[illegible]	[illegible]	[illegible]	[illegible]	[illegible]	[illegible]	[illegible]	[illegible]	[illegible]	[illegible]	[illegible]	[illegible]	[illegible]	[illegible]	[illegible]	[illegible]	[illegible]	[illegible]	[illegible]	[illegible]	[illegible]	[illegible]	[illegible]	[illegible]	[illegible]	[illegible]	[illegible]	[illegible]	[illegible]	[illegible]	[illegible]	[illegible]	[illegible]	[illegible]	[illegible]	[illegible]	[illegible]	[illegible]	[illegible]	[illegible]	[illegible]	[illegible]	[illegible]	[illegible]	[illegible]	[illegible]	[illegible]	[illegible]	[illegible]	[illegible]	[illegible]	[illegible]	[illegible]	[illegible]	[illegible]	[illegible]
M00527: Lysine_biosynthesis,_DAP_aminotransferase_pathway,_aspartate_=>_lysine	[illegible]	[illegible]	[illegible]	[illegible]	[illegible]	[illegible]	[illegible]	[illegible]	[illegible]	[illegible]	[illegible]	[illegible]	[illegible]	[illegible]	[illegible]	[illegible]	[illegible]	[illegible]	[illegible]	[illegible]	[illegible]	[illegible]	[illegible]	[illegible]	[illegible]	[illegible]	[illegible]	[illegible]	[illegible]	[illegible]	[illegible]	[illegible]	[illegible]	[illegible]	[illegible]	[illegible]	[illegible]	[illegible]	[illegible]	[illegible]	[illegible]	[illegible]	[illegible]	[illegible]	[illegible]	[illegible]	[illegible]	[illegible]	[illegible]	[illegible]	[illegible]	[illegible]	[illegible]	[illegible]	[illegible]	[illegible]	[illegible]	[illegible]	[illegible]	[illegible]	[illegible]	[illegible]	[illegible]	[illegible]	[illegible]	[illegible]	[illegible]
M00528: Nitrification,_ammonia_=>_nitrite	[illegible]	[illegible]	[illegible]	[illegible]	[illegible]	[illegible]	[illegible]	[illegible]	[illegible]	[illegible]	[illegible]	[illegible]	[illegible]	[illegible]	[illegible]	[illegible]	[illegible]	[illegible]	[illegible]	[illegible]	[illegible]	[illegible]	[illegible]	[illegible]	[illegible]	[illegible]	[illegible]	[illegible]	[illegible]	[illegible]	[illegible]	[illegible]	[illegible]	[illegible]	[illegible]	[illegible]	[illegible]	[illegible]	[illegible]	[illegible]	[illegible]	[illegible]	[illegible]	[illegible]	[illegible]	[illegible]	[illegible]	[illegible]	[illegible]	[illegible]	[illegible]	[illegible]	[illegible]	[illegible]	[illegible]	[illegible]	[illegible]	[illegible]	[illegible]	[illegible]	[illegible]	[illegible]	[illegible]	[illegible]	[illegible]	[illegible]	[illegible]
M00529: Denitrification,_nitrate_=>_nitrogen	[illegible]	[illegible]	[illegible]	[illegible]	[illegible]	[illegible]	[illegible]	[illegible]	[illegible]	[illegible]	[illegible]	[illegible]	[illegible]	[illegible]	[illegible]	[illegible]	[illegible]	[illegible]	[illegible]	[illegible]	[illegible]	[illegible]	[illegible]	[illegible]	[illegible]	[illegible]	[illegible]	[illegible]	[illegible]	[illegible]	[illegible]	[illegible]	[illegible]	[illegible]	[illegible]	[illegible]	[illegible]	[illegible]	[illegible]	[illegible]	[illegible]	[illegible]	[illegible]	[illegible]	[illegible]	[illegible]	[illegible]	[illegible]	[illegible]	[illegible]	[illegible]	[illegible]	[illegible]	[illegible]	[illegible]	[illegible]	[illegible]	[illegible]	[illegible]	[illegible]	[illegible]	[illegible]	[illegible]	[illegible]	[illegible]	[illegible]	[illegible]
M00530: Dissimilatory_nitrate_reduction,_nitrate_=>_ammonia	[illegible]	[illegible]	[illegible]	[illegible]	[illegible]	[illegible]	[illegible]	[illegible]	[illegible]	[illegible]	[illegible]	[illegible]	[illegible]	[illegible]	[illegible]	[illegible]	[illegible]	[illegible]	[illegible]	[illegible]	[illegible]	[illegible]	[illegible]	[illegible]	[illegible]	[illegible]	[illegible]	[illegible]	[illegible]	[illegible]	[illegible]	[illegible]	[illegible]	[illegible]	[illegible]	[illegible]	[illegible]	[illegible]	[illegible]	[illegible]	[illegible]	[illegible]	[illegible]	[illegible]	[illegible]	[illegible]	[illegible]	[illegible]	[illegible]	[illegible]	[illegible]	[illegible]	[illegible]	[illegible]	[illegible]	[illegible]	[illegible]	[illegible]	[illegible]	[illegible]	[illegible]	[illegible]	[illegible]	[illegible]	[illegible]	[illegible]	[illegible]
M00531: Assimilatory_nitrate_reduction,_nitrate_=>_ammonia	[illegible]	[illegible]	[illegible]	[illegible]	[illegible]	[illegible]	[illegible]	[illegible]	[illegible]	[illegible]	[illegible]	[illegible]	[illegible]	[illegible]	[illegible]	[illegible]	[illegible]	[illegible]	[illegible]	[illegible]	[illegible]	[illegible]	[illegible]	[illegible]	[illegible]	[illegible]	[illegible]	[illegible]	[illegible]	[illegible]	[illegible]	[illegible]	[illegible]	[illegible]	[illegible]	[illegible]	[illegible]	[illegible]	[illegible]	[illegible]	[illegible]	[illegible]	[illegible]	[illegible]	[illegible]	[illegible]	[illegible]	[illegible]	[illegible]	[illegible]	[illegible]	[illegible]	[illegible]	[illegible]	[illegible]	[illegible]	[illegible]	[illegible]	[illegible]	[illegible]	[illegible]	[illegible]	[illegible]	[illegible]	[illegible]	[illegible]	[illegible]
M00532: Photorespiration	[illegible]	[illegible]	[illegible]	[illegible]	[illegible]	[illegible]	[illegible]	[illegible]	[illegible]	[illegible]	[illegible]	[illegible]	[illegible]	[illegible]	[illegible]	[illegible]	[illegible]	[illegible]	[illegible]	[illegible]	[illegible]	[illegible]	[illegible]	[illegible]	[illegible]	[illegible]	[illegible]	[illegible]	[illegible]	[illegible]	[illegible]	[illegible]	[illegible]	[illegible]	[illegible]	[illegible]	[illegible]	[illegible]	[illegible]	[illegible]	[illegible]	[illegible]	[illegible]	[illegible]	[illegible]	[illegible]	[illegible]	[illegible]	[illegible]	[illegible]	[illegible]	[illegible]	[illegible]	[illegible]	[illegible]	[illegible]	[illegible]	[illegible]	[illegible]	[illegible]	[illegible]	[illegible]	[illegible]	[illegible]	[illegible]	[illegible]	[illegible]
M00533: Homoprotocatechuate_degradation,_homoprotocatechuate_=>_2-oxohept-3-enedioate	[illegible]	[illegible]	[illegible]	[illegible]	[illegible]	[illegible]	[illegible]	[illegible]	[illegible]	[illegible]	[illegible]	[illegible]	[illegible]	[illegible]	[illegible]	[illegible]	[illegible]	[illegible]	[illegible]	[illegible]	[illegible]	[illegible]	[illegible]	[illegible]	[illegible]	[illegible]	[illegible]	[illegible]	[illegible]	[illegible]	[illegible]	[illegible]	[illegible]	[illegible]	[illegible]	[illegible]	[illegible]	[illegible]	[illegible]	[illegible]	[illegible]	[illegible]	[illegible]	[illegible]	[illegible]	[illegible]	[illegible]	[illegible]	[illegible]	[illegible]	[illegible]	[illegible]	[illegible]	[illegible]	[illegible]	[illegible]	[illegible]	[illegible]	[illegible]	[illegible]	[illegible]	[illegible]	[illegible]	[illegible]	[illegible]	[illegible]	[illegible]
M00534: Naphthalene_degradation,_naphthalene_=>_salicylate	[illegible]	[illegible]	[illegible]	[illegible]	[illegible]	[illegible]	[illegible]	[illegible]	[illegible]	[illegible]	[illegible]	[illegible]	[illegible]	[illegible]	[illegible]	[illegible]	[illegible]	[illegible]	[illegible]	[illegible]	[illegible]	[illegible]	[illegible]	[illegible]	[illegible]	[illegible]	[illegible]	[illegible]	[illegible]	[illegible]	[illegible]	[illegible]	[illegible]	[illegible]	[illegible]	[illegible]	[illegible]	[illegible]	[illegible]	[illegible]	[illegible]	[illegible]	[illegible]	[illegible]	[illegible]	[illegible]	[illegible]	[illegible]	[illegible]	[illegible]	[illegible]	[illegible]	[illegible]	[illegible]	[illegible]	[illegible]	[illegible]	[illegible]	[illegible]	[illegible]	[illegible]	[illegible]	[illegible]	[illegible]	[illegible]	[illegible]	[illegible]
M00535: Isoleucine_biosynthesis,_pyruvate_=>_2-oxobutanoate	[illegible]	[illegible]	[illegible]	[illegible]	[illegible]	[illegible]	[illegible]	[illegible]	[illegible]	[illegible]	[illegible]	[illegible]	[illegible]	[illegible]	[illegible]	[illegible]	[illegible]	[illegible]	[illegible]	[illegible]	[illegible]	[illegible]	[illegible]	[illegible]	[illegible]	[illegible]	[illegible]	[illegible]	[illegible]	[illegible]	[illegible]	[illegible]	[illegible]	[illegible]	[illegible]	[illegible]	[illegible]	[illegible]	[illegible]	[illegible]	[illegible]	[illegible]	[illegible]	[illegible]	[illegible]	[illegible]	[illegible]	[illegible]	[illegible]	[illegible]	[illegible]	[illegible]	[illegible]	[illegible]	[illegible]	[illegible]	[illegible]	[illegible]	[illegible]	[illegible]	[illegible]	[illegible]	[illegible]	[illegible]	[illegible]	[illegible]	[illegible]
M00537: Xylene_degradation,_xylene_=>_methylbenzoate	[illegible]	[illegible]	[illegible]	[illegible]	[illegible]	[illegible]	[illegible]	[illegible]	[illegible]	[illegible]	[illegible]	[illegible]	[illegible]	[illegible]	[illegible]	[illegible]	[illegible]	[illegible]	[illegible]	[illegible]	[illegible]	[illegible]	[illegible]	[illegible]	[illegible]	[illegible]	[illegible]	[illegible]	[illegible]	[illegible]	[illegible]	[illegible]	[illegible]	[illegible]	[illegible]	[illegible]	[illegible]	[illegible]	[illegible]	[illegible]	[illegible]	[illegible]	[illegible]	[illegible]	[illegible]	[illegible]	[illegible]	[illegible]	[illegible]	[illegible]	[illegible]	[illegible]	[illegible]	[illegible]	[illegible]	[illegible]	[illegible]	[illegible]	[illegible]	[illegible]	[illegible]	[illegible]	[illegible]	[illegible]	[illegible]	[illegible]	[illegible]
M00538: Toluene_degradation,_toluene_=>_benzoate	[illegible]	[illegible]	[illegible]	[illegible]	[illegible]	[illegible]	[illegible]	[illegible]	[illegible]	[illegible]	[illegible]	[illegible]	[illegible]	[illegible]	[illegible]	[illegible]	[illegible]	[illegible]	[illegible]	[illegible]	[illegible]	[illegible]	[illegible]	[illegible]	[illegible]	[illegible]	[illegible]	[illegible]	[illegible]	[illegible]	[illegible]	[illegible]	[illegible]	[illegible]	[illegible]	[illegible]	[illegible]	[illegible]	[illegible]	[illegible]	[illegible]	[illegible]	[illegible]	[illegible]	[illegible]	[illegible]	[illegible]	[illegible]	[illegible]	[illegible]	[illegible]	[illegible]	[illegible]	[illegible]	[illegible]	[illegible]	[illegible]	[illegible]	[illegible]	[illegible]	[illegible]	[illegible]	[illegible]	[illegible]	[illegible]	[illegible]	[illegible]
M00539: Cumate_degradation,_p-cumate_=>_2-oxopent-4-enoate_+_2-methylpropanoate	[illegible]	[illegible]	[illegible]	[illegible]	[illegible]	[illegible]	[illegible]	[illegible]	[illegible]	[illegible]	[illegible]	[illegible]	[illegible]	[illegible]	[illegible]	[illegible]	[illegible]	[illegible]	[illegible]	[illegible]	[illegible]	[illegible]	[illegible]	[illegible]	[illegible]	[illegible]	[illegible]	[illegible]	[illegible]	[illegible]	[illegible]	[illegible]	[illegible]	[illegible]	[illegible]	[illegible]	[illegible]	[illegible]	[illegible]	[illegible]	[illegible]	[illegible]	[illegible]	[illegible]	[illegible]	[illegible]	[illegible]	[illegible]	[illegible]	[illegible]	[illegible]	[illegible]	[illegible]	[illegible]	[illegible]	[illegible]	[illegible]	[illegible]	[illegible]	[illegible]	[illegible]	[illegible]	[illegible]	[illegible]	[illegible]	[illegible]	[illegible]
M00540: Benzoate_degradation,_cyclohexanecarboxylic_acid_=>pimeloyl-CoA	[illegible]	[illegible]	[illegible]	[illegible]	[illegible]	[illegible]	[illegible]	[illegible]	[illegible]	[illegible]	[illegible]	[illegible]	[illegible]	[illegible]	[illegible]	[illegible]	[illegible]	[illegible]	[illegible]	[illegible]	[illegible]	[illegible]	[illegible]	[illegible]	[illegible]	[illegible]	[illegible]	[illegible]	[illegible]	[illegible]	[illegible]	[illegible]	[illegible]	[illegible]	[illegible]	[illegible]	[illegible]	[illegible]	[illegible]	[illegible]	[illegible]	[illegible]	[illegible]	[illegible]	[illegible]	[illegible]	[illegible]	[illegible]	[illegible]	[illegible]	[illegible]	[illegible]	[illegible]	[illegible]	[illegible]	[illegible]	[illegible]	[illegible]	[illegible]	[illegible]	[illegible]	[illegible]	[illegible]	[illegible]	[illegible]	[illegible]	[illegible]
M00541: Benzoyl-CoA_degradation,_benzoyl-CoA_=>_3-hydroxypimeloyl-CoA	[illegible]	[illegible]	[illegible]	[illegible]	[illegible]	[illegible]	[illegible]	[illegible]	[illegible]	[illegible]	[illegible]	[illegible]	[illegible]	[illegible]	[illegible]	[illegible]	[illegible]	[illegible]	[illegible]	[illegible]	[illegible]	[illegible]	[illegible]	[illegible]	[illegible]	[illegible]	[illegible]	[illegible]	[illegible]	[illegible]	[illegible]	[illegible]	[illegible]	[illegible]	[illegible]	[illegible]	[illegible]	[illegible]	[illegible]	[illegible]	[illegible]	[illegible]	[illegible]	[illegible]	[illegible]	[illegible]	[illegible]	[illegible]	[illegible]	[illegible]	[illegible]	[illegible]	[illegible]	[illegible]	[illegible]	[illegible]	[illegible]	[illegible]	[illegible]	[illegible]	[illegible]	[illegible]	[illegible]	[illegible]	[illegible]	[illegible]	[illegible]
M00542: EHEC/EPEC_pathogenicity_signature,_T3SS_and_effectors	[illegible]	[illegible]	[illegible]	[illegible]	[illegible]	[illegible]	[illegible]	[illegible]	[illegible]	[illegible]	[illegible]	[illegible]	[illegible]	[illegible]	[illegible]	[illegible]	[illegible]	[illegible]	[illegible]	[illegible]	[illegible]	[illegible]	[illegible]	[illegible]	[illegible]	[illegible]	[illegible]	[illegible]	[illegible]	[illegible]	[illegible]	[illegible]	[illegible]	[illegible]	[illegible]	[illegible]	[illegible]	[illegible]	[illegible]	[illegible]	[illegible]	[illegible]	[illegible]	[illegible]	[illegible]	[illegible]	[illegible]	[illegible]	[illegible]	[illegible]	[illegible]	[illegible]	[illegible]	[illegible]	[illegible]	[illegible]	[illegible]	[illegible]	[illegible]	[illegible]	[illegible]	[illegible]	[illegible]	[illegible]	[illegible]	[illegible]	[illegible]
M00543: Biphenyl_degradation,_biphenyl_=>_2-oxopent-4-enoate_+_benzoate	[illegible]	[illegible]	[illegible]	[illegible]	[illegible]	[illegible]	[illegible]	[illegible]	[illegible]	[illegible]	[illegible]	[illegible]	[illegible]	[illegible]	[illegible]	[illegible]	[illegible]	[illegible]	[illegible]	[illegible]	[illegible]	[illegible]	[illegible]	[illegible]	[illegible]	[illegible]	[illegible]	[illegible]	[illegible]	[illegible]	[illegible]	[illegible]	[illegible]	[illegible]	[illegible]	[illegible]	[illegible]	[illegible]	[illegible]	[illegible]	[illegible]	[illegible]	[illegible]	[illegible]	[illegible]	[illegible]	[illegible]	[illegible]	[illegible]	[illegible]	[illegible]	[illegible]	[illegible]	[illegible]	[illegible]	[illegible]	[illegible]	[illegible]	[illegible]	[illegible]	[illegible]	[illegible]	[illegible]	[illegible]	[illegible]	[illegible]	[illegible]
M00544: Carbazole_degradation,_carbazole_=>_2-oxopent-4-enoate_+_anthranilate	[illegible]	[illegible]	[illegible]	[illegible]	[illegible]	[illegible]	[illegible]	[illegible]	[illegible]	[illegible]	[illegible]	[illegible]	[illegible]	[illegible]	[illegible]	[illegible]	[illegible]	[illegible]	[illegible]	[illegible]	[illegible]	[illegible]	[illegible]	[illegible]	[illegible]	[illegible]	[illegible]	[illegible]	[illegible]	[illegible]	[illegible]	[illegible]	[illegible]	[illegible]	[illegible]	[illegible]	[illegible]	[illegible]	[illegible]	[illegible]	[illegible]	[illegible]	[illegible]	[illegible]	[illegible]	[illegible]	[illegible]	[illegible]	[illegible]	[illegible]	[illegible]	[illegible]	[illegible]	[illegible]	[illegible]	[illegible]	[illegible]	[illegible]	[illegible]	[illegible]	[illegible]	[illegible]	[illegible]	[illegible]	[illegible]	[illegible]	[illegible]
M00545: Trans-cinnamate_degradation,_trans-cinnamate_=>_acetyl-CoA	[illegible]	[illegible]	[illegible]	[illegible]	[illegible]	[illegible]	[illegible]	[illegible]	[illegible]	[illegible]	[illegible]	[illegible]	[illegible]	[illegible]	[illegible]	[illegible]	[illegible]	[illegible]	[illegible]	[illegible]	[illegible]	[illegible]	[illegible]	[illegible]	[illegible]	[illegible]	[illegible]	[illegible]	[illegible]	[illegible]	[illegible]	[illegible]	[illegible]	[illegible]	[illegible]	[illegible]	[illegible]	[illegible]	[illegible]	[illegible]	[illegible]	[illegible]	[illegible]	[illegible]	[illegible]	[illegible]	[illegible]	[illegible]	[illegible]	[illegible]	[illegible]	[illegible]	[illegible]	[illegible]	[illegible]	[illegible]	[illegible]	[illegible]	[illegible]	[illegible]	[illegible]	[illegible]	[illegible]	[illegible]	[illegible]	[illegible]	[illegible]
M00546: Purine_degradation,_xanthine_=>_urea	[illegible]	[illegible]	[illegible]	[illegible]	[illegible]	[illegible]	[illegible]	[illegible]	[illegible]	[illegible]	[illegible]	[illegible]	[illegible]	[illegible]	[illegible]	[illegible]	[illegible]	[illegible]	[illegible]	[illegible]	[illegible]	[illegible]	[illegible]	[illegible]	[illegible]	[illegible]	[illegible]	[illegible]	[illegible]	[illegible]	[illegible]	[illegible]	[illegible]	[illegible]	[illegible]	[illegible]	[illegible]	[illegible]	[illegible]	[illegible]	[illegible]	[illegible]	[illegible]	[illegible]	[illegible]	[illegible]	[illegible]	[illegible]	[illegible]	[illegible]	[illegible]	[illegible]	[illegible]	[illegible]	[illegible]	[illegible]	[illegible]	[illegible]	[illegible]	[illegible]	[illegible]	[illegible]	[illegible]	[illegible]	[illegible]	[illegible]	[illegible]
M00547: Benzene/toluene_degradation,_benzene_=>_catechol_/_toluene_=>_3-methylcatechol	[illegible]	[illegible]	[illegible]	[illegible]	[illegible]	[illegible]	[illegible]	[illegible]	[illegible]	[illegible]	[illegible]	[illegible]	[illegible]	[illegible]	[illegible]	[illegible]	[illegible]	[illegible]	[illegible]	[illegible]	[illegible]	[illegible]	[illegible]	[illegible]	[illegible]	[illegible]	[illegible]	[illegible]	[illegible]	[illegible]	[illegible]	[illegible]	[illegible]	[illegible]	[illegible]	[illegible]	[illegible]	[illegible]	[illegible]	[illegible]	[illegible]	[illegible]	[illegible]	[illegible]	[illegible]	[illegible]	[illegible]	[illegible]	[illegible]	[illegible]	[illegible]	[illegible]	[illegible]	[illegible]	[illegible]	[illegible]	[illegible]	[illegible]	[illegible]	[illegible]	[illegible]	[illegible]	[illegible]	[illegible]	[illegible]	[illegible]	[illegible]
M00548: Benzene_degradation,_benzene_=>_catechol	[illegible]	[illegible]	[illegible]	[illegible]	[illegible]	[illegible]	[illegible]	[illegible]	[illegible]	[illegible]	[illegible]	[illegible]	[illegible]	[illegible]	[illegible]	[illegible]	[illegible]	[illegible]	[illegible]	[illegible]	[illegible]	[illegible]	[illegible]	[illegible]	[illegible]	[illegible]	[illegible]	[illegible]	[illegible]	[illegible]	[illegible]	[illegible]	[illegible]	[illegible]	[illegible]	[illegible]	[illegible]	[illegible]	[illegible]	[illegible]	[illegible]	[illegible]	[illegible]	[illegible]	[illegible]	[illegible]	[illegible]	[illegible]	[illegible]	[illegible]	[illegible]	[illegible]	[illegible]	[illegible]	[illegible]	[illegible]	[illegible]	[illegible]	[illegible]	[illegible]	[illegible]	[illegible]	[illegible]	[illegible]	[illegible]	[illegible]	[illegible]
M00549: Nucleotide_sugar_biosynthesis,_glucose_=>_UDP-glucose	[illegible]	[illegible]	[illegible]	[illegible]	[illegible]	[illegible]	[illegible]	[illegible]	[illegible]	[illegible]	[illegible]	[illegible]	[illegible]	[illegible]	[illegible]	[illegible]	[illegible]	[illegible]	[illegible]	[illegible]	[illegible]	[illegible]	[illegible]	[illegible]	[illegible]	[illegible]	[illegible]	[illegible]	[illegible]	[illegible]	[illegible]	[illegible]	[illegible]	[illegible]	[illegible]	[illegible]	[illegible]	[illegible]	[illegible]	[illegible]	[illegible]	[illegible]	[illegible]	[illegible]	[illegible]	[illegible]	[illegible]	[illegible]	[illegible]	[illegible]	[illegible]	[illegible]	[illegible]	[illegible]	[illegible]	[illegible]	[illegible]	[illegible]	[illegible]	[illegible]	[illegible]	[illegible]	[illegible]	[illegible]	[illegible]	[illegible]	[illegible]
M00550: Ascorbate_degradation,_ascorbate_=>_D-xylulose-5P	[illegible]	[illegible]	[illegible]	[illegible]	[illegible]	[illegible]	[illegible]	[illegible]	[illegible]	[illegible]	[illegible]	[illegible]	[illegible]	[illegible]	[illegible]	[illegible]	[illegible]	[illegible]	[illegible]	[illegible]	[illegible]	[illegible]	[illegible]	[illegible]	[illegible]	[illegible]	[illegible]	[illegible]	[illegible]	[illegible]	[illegible]	[illegible]	[illegible]	[illegible]	[illegible]	[illegible]	[illegible]	[illegible]	[illegible]	[illegible]	[illegible]	[illegible]	[illegible]	[illegible]	[illegible]	[illegible]	[illegible]	[illegible]	[illegible]	[illegible]	[illegible]	[illegible]	[illegible]	[illegible]	[illegible]	[illegible]	[illegible]	[illegible]	[illegible]	[illegible]	[illegible]	[illegible]	[illegible]	[illegible]	[illegible]	[illegible]	[illegible]
M00551: Benzoate_degradation,_benzoate_=>_catechol_/_methylbenzoate_=>_methylcatechol	[illegible]	[illegible]	[illegible]	[illegible]	[illegible]	[illegible]	[illegible]	[illegible]	[illegible]	[illegible]	[illegible]	[illegible]	[illegible]	[illegible]	[illegible]	[illegible]	[illegible]	[illegible]	[illegible]	[illegible]	[illegible]	[illegible]	[illegible]	[illegible]	[illegible]	[illegible]	[illegible]	[illegible]	[illegible]	[illegible]	[illegible]	[illegible]	[illegible]	[illegible]	[illegible]	[illegible]	[illegible]	[illegible]	[illegible]	[illegible]	[illegible]	[illegible]	[illegible]	[illegible]	[illegible]	[illegible]	[illegible]	[illegible]	[illegible]	[illegible]	[illegible]	[illegible]	[illegible]	[illegible]	[illegible]	[illegible]	[illegible]	[illegible]	[illegible]	[illegible]	[illegible]	[illegible]	[illegible]	[illegible]	[illegible]	[illegible]	[illegible]
M00552: D-galactonate_degradation,_De_Ley-Doudoroff_pathway,_D-galactonate_=>_glycerate-3P	[illegible]	[illegible]	[illegible]	[illegible]	[illegible]	[illegible]	[illegible]	[illegible]	[illegible]	[illegible]	[illegible]	[illegible]	[illegible]	[illegible]	[illegible]	[illegible]	[illegible]	[illegible]	[illegible]	[illegible]	[illegible]	[illegible]	[illegible]	[illegible]	[illegible]	[illegible]	[illegible]	[illegible]	[illegible]	[illegible]	[illegible]	[illegible]	[illegible]	[illegible]	[illegible]	[illegible]	[illegible]	[illegible]	[illegible]	[illegible]	[illegible]	[illegible]	[illegible]	[illegible]	[illegible]	[illegible]	[illegible]	[illegible]	[illegible]	[illegible]	[illegible]	[illegible]	[illegible]	[illegible]	[illegible]	[illegible]	[illegible]	[illegible]	[illegible]	[illegible]	[illegible]	[illegible]	[illegible]	[illegible]	[illegible]	[illegible]	[illegible]
M00554: Nucleotide_sugar_biosynthesis,_galactose_=>_UDP-galactose	[illegible]	[illegible]	[illegible]	[illegible]	[illegible]	[illegible]	[illegible]	[illegible]	[illegible]	[illegible]	[illegible]	[illegible]	[illegible]	[illegible]	[illegible]	[illegible]	[illegible]	[illegible]	[illegible]	[illegible]	[illegible]	[illegible]	[illegible]	[illegible]	[illegible]	[illegible]	[illegible]	[illegible]	[illegible]	[illegible]	[illegible]	[illegible]	[illegible]	[illegible]	[illegible]	[illegible]	[illegible]	[illegible]	[illegible]	[illegible]	[illegible]	[illegible]	[illegible]	[illegible]	[illegible]	[illegible]	[illegible]	[illegible]	[illegible]	[illegible]	[illegible]	[illegible]	[illegible]	[illegible]	[illegible]	[illegible]	[illegible]	[illegible]	[illegible]	[illegible]	[illegible]	[illegible]	[illegible]	[illegible]	[illegible]	[illegible]	[illegible]
M00555: Betaine_biosynthesis,_choline_=>_betaine	[illegible]	[illegible]	[illegible]	[illegible]	[illegible]	[illegible]	[illegible]	[illegible]	[illegible]	[illegible]	[illegible]	[illegible]	[illegible]	[illegible]	[illegible]	[illegible]	[illegible]	[illegible]	[illegible]	[illegible]	[illegible]	[illegible]	[illegible]	[illegible]	[illegible]	[illegible]	[illegible]	[illegible]	[illegible]	[illegible]	[illegible]	[illegible]	[illegible]	[illegible]	[illegible]	[illegible]	[illegible]	[illegible]	[illegible]	[illegible]	[illegible]	[illegible]	[illegible]	[illegible]	[illegible]	[illegible]	[illegible]	[illegible]	[illegible]	[illegible]	[illegible]	[illegible]	[illegible]	[illegible]	[illegible]	[illegible]	[illegible]	[illegible]	[illegible]	[illegible]	[illegible]	[illegible]	[illegible]	[illegible]	[illegible]	[illegible]	[illegible]
M00565: Trehalose_biosynthesis,_D-glucose_1P_=>_trehalose	[illegible]	[illegible]	[illegible]	[illegible]	[illegible]	[illegible]	[illegible]	[illegible]	[illegible]	[illegible]	[illegible]	[illegible]	[illegible]	[illegible]	[illegible]	[illegible]	[illegible]	[illegible]	[illegible]	[illegible]	[illegible]	[illegible]	[illegible]	[illegible]	[illegible]	[illegible]	[illegible]	[illegible]	[illegible]	[illegible]	[illegible]	[illegible]	[illegible]	[illegible]	[illegible]	[illegible]	[illegible]	[illegible]	[illegible]	[illegible]	[illegible]	[illegible]	[illegible]	[illegible]	[illegible]	[illegible]	[illegible]	[illegible]	[illegible]	[illegible]	[illegible]	[illegible]	[illegible]	[illegible]	[illegible]	[illegible]	[illegible]	[illegible]	[illegible]	[illegible]	[illegible]	[illegible]	[illegible]	[illegible]	[illegible]	[illegible]	[illegible]
M00566: Dipeptide_transport_system,_Firmicutes	[illegible]	[illegible]	[illegible]	[illegible]	[illegible]	[illegible]	[illegible]	[illegible]	[illegible]	[illegible]	[illegible]	[illegible]	[illegible]	[illegible]	[illegible]	[illegible]	[illegible]	[illegible]	[illegible]	[illegible]	[illegible]	[illegible]	[illegible]	[illegible]	[illegible]	[illegible]	[illegible]	[illegible]	[illegible]	[illegible]	[illegible]	[illegible]	[illegible]	[illegible]	[illegible]	[illegible]	[illegible]	[illegible]	[illegible]	[illegible]	[illegible]	[illegible]	[illegible]	[illegible]	[illegible]	[illegible]	[illegible]	[illegible]	[illegible]	[illegible]	[illegible]	[illegible]	[illegible]	[illegible]	[illegible]	[illegible]	[illegible]	[illegible]	[illegible]	[illegible]	[illegible]	[illegible]	[illegible]	[illegible]	[illegible]	[illegible]	[illegible]
M00568: Catechol_ortho-cleavage,_catechol_=>_3-oxoadipate	[illegible]	[illegible]	[illegible]	[illegible]	[illegible]	[illegible]	[illegible]	[illegible]	[illegible]	[illegible]	[illegible]	[illegible]	[illegible]	[illegible]	[illegible]	[illegible]	[illegible]	[illegible]	[illegible]	[illegible]	[illegible]	[illegible]	[illegible]	[illegible]	[illegible]	[illegible]	[illegible]	[illegible]	[illegible]	[illegible]	[illegible]	[illegible]	[illegible]	[illegible]	[illegible]	[illegible]	[illegible]	[illegible]	[illegible]	[illegible]	[illegible]	[illegible]	[illegible]	[illegible]	[illegible]	[illegible]	[illegible]	[illegible]	[illegible]	[illegible]	[illegible]	[illegible]	[illegible]	[illegible]	[illegible]	[illegible]	[illegible]	[illegible]	[illegible]	[illegible]	[illegible]	[illegible]	[illegible]	[illegible]	[illegible]	[illegible]	[illegible]
M00569: Catechol_meta-cleavage,_catechol_=>_acetyl-CoA_/_4-methylcatechol_=>_propanoyl-CoA	[illegible]	[illegible]	[illegible]	[illegible]	[illegible]	[illegible]	[illegible]	[illegible]	[illegible]	[illegible]	[illegible]	[illegible]	[illegible]	[illegible]	[illegible]	[illegible]	[illegible]	[illegible]	[illegible]	[illegible]	[illegible]	[illegible]	[illegible]	[illegible]	[illegible]	[illegible]	[illegible]	[illegible]	[illegible]	[illegible]	[illegible]	[illegible]	[illegible]	[illegible]	[illegible]	[illegible]	[illegible]	[illegible]	[illegible]	[illegible]	[illegible]	[illegible]	[illegible]	[illegible]	[illegible]	[illegible]	[illegible]	[illegible]	[illegible]	[illegible]	[illegible]	[illegible]	[illegible]	[illegible]	[illegible]	[illegible]	[illegible]	[illegible]	[illegible]	[illegible]	[illegible]	[illegible]	[illegible]	[illegible]	[illegible]	[illegible]	[illegible]
M00570: Isoleucine_biosynthesis,_threonine_=>_2-oxobutanoate_=>_isoleucine	[illegible]	[illegible]	[illegible]	[illegible]	[illegible]	[illegible]	[illegible]	[illegible]	[illegible]	[illegible]	[illegible]	[illegible]	[illegible]	[illegible]	[illegible]	[illegible]	[illegible]	[illegible]	[illegible]	[illegible]	[illegible]	[illegible]	[illegible]	[illegible]	[illegible]	[illegible]	[illegible]	[illegible]	[illegible]	[illegible]	[illegible]	[illegible]	[illegible]	[illegible]	[illegible]	[illegible]	[illegible]	[illegible]	[illegible]	[illegible]	[illegible]	[illegible]	[illegible]	[illegible]	[illegible]	[illegible]	[illegible]	[illegible]	[illegible]	[illegible]	[illegible]	[illegible]	[illegible]	[illegible]	[illegible]	[illegible]	[illegible]	[illegible]	[illegible]	[illegible]	[illegible]	[illegible]	[illegible]	[illegible]	[illegible]	[illegible]	[illegible]
M00571: AlgE-type_Mannuronan_C-5-Epimerase_transport_system	[illegible]	[illegible]	[illegible]	[illegible]	[illegible]	[illegible]	[illegible]	[illegible]	[illegible]	[illegible]	[illegible]	[illegible]	[illegible]	[illegible]	[illegible]	[illegible]	[illegible]	[illegible]	[illegible]	[illegible]	[illegible]	[illegible]	[illegible]	[illegible]	[illegible]	[illegible]	[illegible]	[illegible]	[illegible]	[illegible]	[illegible]	[illegible]	[illegible]	[illegible]	[illegible]	[illegible]	[illegible]	[illegible]	[illegible]	[illegible]	[illegible]	[illegible]	[illegible]	[illegible]	[illegible]	[illegible]	[illegible]	[illegible]	[illegible]	[illegible]	[illegible]	[illegible]	[illegible]	[illegible]	[illegible]	[illegible]	[illegible]	[illegible]	[illegible]	[illegible]	[illegible]	[illegible]	[illegible]	[illegible]	[illegible]	[illegible]	[illegible]
M00572: Pimeloyl-ACP_biosynthesis,_BioC-BioH_pathway,_malonyl-ACP_=>_pimeloyl-ACP	[illegible]	[illegible]	[illegible]	[illegible]	[illegible]	[illegible]	[illegible]	[illegible]	[illegible]	[illegible]	[illegible]	[illegible]	[illegible]	[illegible]	[illegible]	[illegible]	[illegible]	[illegible]	[illegible]	[illegible]	[illegible]	[illegible]	[illegible]	[illegible]	[illegible]	[illegible]	[illegible]	[illegible]	[illegible]	[illegible]	[illegible]	[illegible]	[illegible]	[illegible]	[illegible]	[illegible]	[illegible]	[illegible]	[illegible]	[illegible]	[illegible]	[illegible]	[illegible]	[illegible]	[illegible]	[illegible]	[illegible]	[illegible]	[illegible]	[illegible]	[illegible]	[illegible]	[illegible]	[illegible]	[illegible]	[illegible]	[illegible]	[illegible]	[illegible]	[illegible]	[illegible]	[illegible]	[illegible]	[illegible]	[illegible]	[illegible]	[illegible]
M00573: Biotin_biosynthesis,_BioI_pathway,_long-chain-acyl-ACP_=>_pimeloyl-ACP_=>_biotin	[illegible]	[illegible]	[illegible]	[illegible]	[illegible]	[illegible]	[illegible]	[illegible]	[illegible]	[illegible]	[illegible]	[illegible]	[illegible]	[illegible]	[illegible]	[illegible]	[illegible]	[illegible]	[illegible]	[illegible]	[illegible]	[illegible]	[illegible]	[illegible]	[illegible]	[illegible]	[illegible]	[illegible]	[illegible]	[illegible]	[illegible]	[illegible]	[illegible]	[illegible]	[illegible]	[illegible]	[illegible]	[illegible]	[illegible]	[illegible]	[illegible]	[illegible]	[illegible]	[illegible]	[illegible]	[illegible]	[illegible]	[illegible]	[illegible]	[illegible]	[illegible]	[illegible]	[illegible]	[illegible]	[illegible]	[illegible]	[illegible]	[illegible]	[illegible]	[illegible]	[illegible]	[illegible]	[illegible]	[illegible]	[illegible]	[illegible]	[illegible]
M00574: Pertussis_pathogenicity_signature_1,_pertussis_toxin	[illegible]	[illegible]	[illegible]	[illegible]	[illegible]	[illegible]	[illegible]	[illegible]	[illegible]	[illegible]	[illegible]	[illegible]	[illegible]	[illegible]	[illegible]	[illegible]	[illegible]	[illegible]	[illegible]	[illegible]	[illegible]	[illegible]	[illegible]	[illegible]	[illegible]	[illegible]	[illegible]	[illegible]	[illegible]	[illegible]	[illegible]	[illegible]	[illegible]	[illegible]	[illegible]	[illegible]	[illegible]	[illegible]	[illegible]	[illegible]	[illegible]	[illegible]	[illegible]	[illegible]	[illegible]	[illegible]	[illegible]	[illegible]	[illegible]	[illegible]	[illegible]	[illegible]	[illegible]	[illegible]	[illegible]	[illegible]	[illegible]	[illegible]	[illegible]	[illegible]	[illegible]	[illegible]	[illegible]	[illegible]	[illegible]	[illegible]	[illegible]
M00575: Pertussis_pathogenicity_signature_2,_T1SS	[illegible]	[illegible]	[illegible]	[illegible]	[illegible]	[illegible]	[illegible]	[illegible]	[illegible]	[illegible]	[illegible]	[illegible]	[illegible]	[illegible]	[illegible]	[illegible]	[illegible]	[illegible]	[illegible]	[illegible]	[illegible]	[illegible]	[illegible]	[illegible]	[illegible]	[illegible]	[illegible]	[illegible]	[illegible]	[illegible]	[illegible]	[illegible]	[illegible]	[illegible]	[illegible]	[illegible]	[illegible]	[illegible]	[illegible]	[illegible]	[illegible]	[illegible]	[illegible]	[illegible]	[illegible]	[illegible]	[illegible]	[illegible]	[illegible]	[illegible]	[illegible]	[illegible]	[illegible]	[illegible]	[illegible]	[illegible]	[illegible]	[illegible]	[illegible]	[illegible]	[illegible]	[illegible]	[illegible]	[illegible]	[illegible]	[illegible]	[illegible]
M00577: Biotin_biosynthesis,_BioW_pathway,_pimelate_=>_pimeloyl-CoA_=>_biotin	[illegible]	[illegible]	[illegible]	[illegible]	[illegible]	[illegible]	[illegible]	[illegible]	[illegible]	[illegible]	[illegible]	[illegible]	[illegible]	[illegible]	[illegible]	[illegible]	[illegible]	[illegible]	[illegible]	[illegible]	[illegible]	[illegible]	[illegible]	[illegible]	[illegible]	[illegible]	[illegible]	[illegible]	[illegible]	[illegible]	[illegible]	[illegible]	[illegible]	[illegible]	[illegible]	[illegible]	[illegible]	[illegible]	[illegible]	[illegible]	[illegible]	[illegible]	[illegible]	[illegible]	[illegible]	[illegible]	[illegible]	[illegible]	[illegible]	[illegible]	[illegible]	[illegible]	[illegible]	[illegible]	[illegible]	[illegible]	[illegible]	[illegible]	[illegible]	[illegible]	[illegible]	[illegible]	[illegible]	[illegible]	[illegible]	[illegible]	[illegible]
M00579: Phosphate_acetyltransferase-acetate_kinase_pathway,_acetyl-CoA_=>_acetate	[illegible]	[illegible]	[illegible]	[illegible]	[illegible]	[illegible]	[illegible]	[illegible]	[illegible]	[illegible]	[illegible]	[illegible]	[illegible]	[illegible]	[illegible]	[illegible]	[illegible]	[illegible]	[illegible]	[illegible]	[illegible]	[illegible]	[illegible]	[illegible]	[illegible]	[illegible]	[illegible]	[illegible]	[illegible]	[illegible]	[illegible]	[illegible]	[illegible]	[illegible]	[illegible]	[illegible]	[illegible]	[illegible]	[illegible]	[illegible]	[illegible]	[illegible]	[illegible]	[illegible]	[illegible]	[illegible]	[illegible]	[illegible]	[illegible]	[illegible]	[illegible]	[illegible]	[illegible]	[illegible]	[illegible]	[illegible]	[illegible]	[illegible]	[illegible]	[illegible]	[illegible]	[illegible]	[illegible]	[illegible]	[illegible]	[illegible]	[illegible]
M00580: Pentose_phosphate_pathway,_archaea,_fructose_6P_=>_ribose_5P	[illegible]	[illegible]	[illegible]	[illegible]	[illegible]	[illegible]	[illegible]	[illegible]	[illegible]	[illegible]	[illegible]	[illegible]	[illegible]	[illegible]	[illegible]	[illegible]	[illegible]	[illegible]	[illegible]	[illegible]	[illegible]	[illegible]	[illegible]	[illegible]	[illegible]	[illegible]	[illegible]	[illegible]	[illegible]	[illegible]	[illegible]	[illegible]	[illegible]	[illegible]	[illegible]	[illegible]	[illegible]	[illegible]	[illegible]	[illegible]	[illegible]	[illegible]	[illegible]	[illegible]	[illegible]	[illegible]	[illegible]	[illegible]	[illegible]	[illegible]	[illegible]	[illegible]	[illegible]	[illegible]	[illegible]	[illegible]	[illegible]	[illegible]	[illegible]	[illegible]	[illegible]	[illegible]	[illegible]	[illegible]	[illegible]	[illegible]	[illegible]
M00581: Biotin_transport_system	[illegible]	[illegible]	[illegible]	[illegible]	[illegible]	[illegible]	[illegible]	[illegible]	[illegible]	[illegible]	[illegible]	[illegible]	[illegible]	[illegible]	[illegible]	[illegible]	[illegible]	[illegible]	[illegible]	[illegible]	[illegible]	[illegible]	[illegible]	[illegible]	[illegible]	[illegible]	[illegible]	[illegible]	[illegible]	[illegible]	[illegible]	[illegible]	[illegible]	[illegible]	[illegible]	[illegible]	[illegible]	[illegible]	[illegible]	[illegible]	[illegible]	[illegible]	[illegible]	[illegible]	[illegible]	[illegible]	[illegible]	[illegible]	[illegible]	[illegible]	[illegible]	[illegible]	[illegible]	[illegible]	[illegible]	[illegible]	[illegible]	[illegible]	[illegible]	[illegible]	[illegible]	[illegible]	[illegible]	[illegible]	[illegible]	[illegible]	[illegible]
M00582: Energy-coupling_factor_transport_system	[illegible]	[illegible]	[illegible]	[illegible]	[illegible]	[illegible]	[illegible]	[illegible]	[illegible]	[illegible]	[illegible]	[illegible]	[illegible]	[illegible]	[illegible]	[illegible]	[illegible]	[illegible]	[illegible]	[illegible]	[illegible]	[illegible]	[illegible]	[illegible]	[illegible]	[illegible]	[illegible]	[illegible]	[illegible]	[illegible]	[illegible]	[illegible]	[illegible]	[illegible]	[illegible]	[illegible]	[illegible]	[illegible]	[illegible]	[illegible]	[illegible]	[illegible]	[illegible]	[illegible]	[illegible]	[illegible]	[illegible]	[illegible]	[illegible]	[illegible]	[illegible]	[illegible]	[illegible]	[illegible]	[illegible]	[illegible]	[illegible]	[illegible]	[illegible]	[illegible]	[illegible]	[illegible]	[illegible]	[illegible]	[illegible]	[illegible]	[illegible]
M00585: L-Cystine_transport_system	[illegible]	[illegible]	[illegible]	[illegible]	[illegible]	[illegible]	[illegible]	[illegible]	[illegible]	[illegible]	[illegible]	[illegible]	[illegible]	[illegible]	[illegible]	[illegible]	[illegible]	[illegible]	[illegible]	[illegible]	[illegible]	[illegible]	[illegible]	[illegible]	[illegible]	[illegible]	[illegible]	[illegible]	[illegible]	[illegible]	[illegible]	[illegible]	[illegible]	[illegible]	[illegible]	[illegible]	[illegible]	[illegible]	[illegible]	[illegible]	[illegible]	[illegible]	[illegible]	[illegible]	[illegible]	[illegible]	[illegible]	[illegible]	[illegible]	[illegible]	[illegible]	[illegible]	[illegible]	[illegible]	[illegible]	[illegible]	[illegible]	[illegible]	[illegible]	[illegible]	[illegible]	[illegible]	[illegible]	[illegible]	[illegible]	[illegible]	[illegible]
M00586: Putative_amino-acid_transport_system	[illegible]	[illegible]	[illegible]	[illegible]	[illegible]	[illegible]	[illegible]	[illegible]	[illegible]	[illegible]	[illegible]	[illegible]	[illegible]	[illegible]	[illegible]	[illegible]	[illegible]	[illegible]	[illegible]	[illegible]	[illegible]	[illegible]	[illegible]	[illegible]	[illegible]	[illegible]	[illegible]	[illegible]	[illegible]	[illegible]	[illegible]	[illegible]	[illegible]	[illegible]	[illegible]	[illegible]	[illegible]	[illegible]	[illegible]	[illegible]	[illegible]	[illegible]	[illegible]	[illegible]	[illegible]	[illegible]	[illegible]	[illegible]	[illegible]	[illegible]	[illegible]	[illegible]	[illegible]	[illegible]	[illegible]	[illegible]	[illegible]	[illegible]	[illegible]	[illegible]	[illegible]	[illegible]	[illegible]	[illegible]	[illegible]	[illegible]	[illegible]
M00587: Arginine/lysine/histidine/glutamine_transport_system	[illegible]	[illegible]	[illegible]	[illegible]	[illegible]	[illegible]	[illegible]	[illegible]	[illegible]	[illegible]	[illegible]	[illegible]	[illegible]	[illegible]	[illegible]	[illegible]	[illegible]	[illegible]	[illegible]	[illegible]	[illegible]	[illegible]	[illegible]	[illegible]	[illegible]	[illegible]	[illegible]	[illegible]	[illegible]	[illegible]	[illegible]	[illegible]	[illegible]	[illegible]	[illegible]	[illegible]	[illegible]	[illegible]	[illegible]	[illegible]	[illegible]	[illegible]	[illegible]	[illegible]	[illegible]	[illegible]	[illegible]	[illegible]	[illegible]	[illegible]	[illegible]	[illegible]	[illegible]	[illegible]	[illegible]	[illegible]	[illegible]	[illegible]	[illegible]	[illegible]	[illegible]	[illegible]	[illegible]	[illegible]	[illegible]	[illegible]	[illegible]
M00589: Putative_lysine_transport_system	[illegible]	[illegible]	[illegible]	[illegible]	[illegible]	[illegible]	[illegible]	[illegible]	[illegible]	[illegible]	[illegible]	[illegible]	[illegible]	[illegible]	[illegible]	[illegible]	[illegible]	[illegible]	[illegible]	[illegible]	[illegible]	[illegible]	[illegible]	[illegible]	[illegible]	[illegible]	[illegible]	[illegible]	[illegible]	[illegible]	[illegible]	[illegible]	[illegible]	[illegible]	[illegible]	[illegible]	[illegible]	[illegible]	[illegible]	[illegible]	[illegible]	[illegible]	[illegible]	[illegible]	[illegible]	[illegible]	[illegible]	[illegible]	[illegible]	[illegible]	[illegible]	[illegible]	[illegible]	[illegible]	[illegible]	[illegible]	[illegible]	[illegible]	[illegible]	[illegible]	[illegible]	[illegible]	[illegible]	[illegible]	[illegible]	[illegible]	[illegible]
M00590: Erythritol_transport_system	[illegible]	[illegible]	[illegible]	[illegible]	[illegible]	[illegible]	[illegible]	[illegible]	[illegible]	[illegible]	[illegible]	[illegible]	[illegible]	[illegible]	[illegible]	[illegible]	[illegible]	[illegible]	[illegible]	[illegible]	[illegible]	[illegible]	[illegible]	[illegible]	[illegible]	[illegible]	[illegible]	[illegible]	[illegible]	[illegible]	[illegible]	[illegible]	[illegible]	[illegible]	[illegible]	[illegible]	[illegible]	[illegible]	[illegible]	[illegible]	[illegible]	[illegible]	[illegible]	[illegible]	[illegible]	[illegible]	[illegible]	[illegible]	[illegible]	[illegible]	[illegible]	[illegible]	[illegible]	[illegible]	[illegible]	[illegible]	[illegible]	[illegible]	[illegible]	[illegible]	[illegible]	[illegible]	[illegible]	[illegible]	[illegible]	[illegible]	[illegible]
M00591: Putative_xylitol_transport_system	[illegible]	[illegible]	[illegible]	[illegible]	[illegible]	[illegible]	[illegible]	[illegible]	[illegible]	[illegible]	[illegible]	[illegible]	[illegible]	[illegible]	[illegible]	[illegible]	[illegible]	[illegible]	[illegible]	[illegible]	[illegible]	[illegible]	[illegible]	[illegible]	[illegible]	[illegible]	[illegible]	[illegible]	[illegible]	[illegible]	[illegible]	[illegible]	[illegible]	[illegible]	[illegible]	[illegible]	[illegible]	[illegible]	[illegible]	[illegible]	[illegible]	[illegible]	[illegible]	[illegible]	[illegible]	[illegible]	[illegible]	[illegible]	[illegible]	[illegible]	[illegible]	[illegible]	[illegible]	[illegible]	[illegible]	[illegible]	[illegible]	[illegible]	[illegible]	[illegible]	[illegible]	[illegible]	[illegible]	[illegible]	[illegible]	[illegible]	[illegible]
M00592: Inositol_transport_system	[illegible]	[illegible]	[illegible]	[illegible]	[illegible]	[illegible]	[illegible]	[illegible]	[illegible]	[illegible]	[illegible]	[illegible]	[illegible]	[illegible]	[illegible]	[illegible]	[illegible]	[illegible]	[illegible]	[illegible]	[illegible]	[illegible]	[illegible]	[illegible]	[illegible]	[illegible]	[illegible]	[illegible]	[illegible]	[illegible]	[illegible]	[illegible]	[illegible]	[illegible]	[illegible]	[illegible]	[illegible]	[illegible]	[illegible]	[illegible]	[illegible]	[illegible]	[illegible]	[illegible]	[illegible]	[illegible]	[illegible]	[illegible]	[illegible]	[illegible]	[illegible]	[illegible]	[illegible]	[illegible]	[illegible]	[illegible]	[illegible]	[illegible]	[illegible]	[illegible]	[illegible]	[illegible]	[illegible]	[illegible]	[illegible]	[illegible]	[illegible]
M00593: Inositol_transport_system	[illegible]	[illegible]	[illegible]	[illegible]	[illegible]	[illegible]	[illegible]	[illegible]	[illegible]	[illegible]	[illegible]	[illegible]	[illegible]	[illegible]	[illegible]	[illegible]	[illegible]	[illegible]	[illegible]	[illegible]	[illegible]	[illegible]	[illegible]	[illegible]	[illegible]	[illegible]	[illegible]	[illegible]	[illegible]	[illegible]	[illegible]	[illegible]	[illegible]	[illegible]	[illegible]	[illegible]	[illegible]	[illegible]	[illegible]	[illegible]	[illegible]	[illegible]	[illegible]	[illegible]	[illegible]	[illegible]	[illegible]	[illegible]	[illegible]	[illegible]	[illegible]	[illegible]	[illegible]	[illegible]	[illegible]	[illegible]	[illegible]	[illegible]	[illegible]	[illegible]	[illegible]	[illegible]	[illegible]	[illegible]	[illegible]	[illegible]	[illegible]
M00595: Thiosulfate_oxidation_by_SOX_complex,_thiosulfate_=>_sulfate	[illegible]	[illegible]	[illegible]	[illegible]	[illegible]	[illegible]	[illegible]	[illegible]	[illegible]	[illegible]	[illegible]	[illegible]	[illegible]	[illegible]	[illegible]	[illegible]	[illegible]	[illegible]	[illegible]	[illegible]	[illegible]	[illegible]	[illegible]	[illegible]	[illegible]	[illegible]	[illegible]	[illegible]	[illegible]	[illegible]	[illegible]	[illegible]	[illegible]	[illegible]	[illegible]	[illegible]	[illegible]	[illegible]	[illegible]	[illegible]	[illegible]	[illegible]	[illegible]	[illegible]	[illegible]	[illegible]	[illegible]	[illegible]	[illegible]	[illegible]	[illegible]	[illegible]	[illegible]	[illegible]	[illegible]	[illegible]	[illegible]	[illegible]	[illegible]	[illegible]	[illegible]	[illegible]	[illegible]	[illegible]	[illegible]	[illegible]	[illegible]
M00596: Dissimilatory_sulfate_reduction,_sulfate_=>_H2S	[illegible]	[illegible]	[illegible]	[illegible]	[illegible]	[illegible]	[illegible]	[illegible]	[illegible]	[illegible]	[illegible]	[illegible]	[illegible]	[illegible]	[illegible]	[illegible]	[illegible]	[illegible]	[illegible]	[illegible]	[illegible]	[illegible]	[illegible]	[illegible]	[illegible]	[illegible]	[illegible]	[illegible]	[illegible]	[illegible]	[illegible]	[illegible]	[illegible]	[illegible]	[illegible]	[illegible]	[illegible]	[illegible]	[illegible]	[illegible]	[illegible]	[illegible]	[illegible]	[illegible]	[illegible]	[illegible]	[illegible]	[illegible]	[illegible]	[illegible]	[illegible]	[illegible]	[illegible]	[illegible]	[illegible]	[illegible]	[illegible]	[illegible]	[illegible]	[illegible]	[illegible]	[illegible]	[illegible]	[illegible]	[illegible]	[illegible]	[illegible]
M00597: Anoxygenic_photosystem_II	[illegible]	[illegible]	[illegible]	[illegible]	[illegible]	[illegible]	[illegible]	[illegible]	[illegible]	[illegible]	[illegible]	[illegible]	[illegible]	[illegible]	[illegible]	[illegible]	[illegible]	[illegible]	[illegible]	[illegible]	[illegible]	[illegible]	[illegible]	[illegible]	[illegible]	[illegible]	[illegible]	[illegible]	[illegible]	[illegible]	[illegible]	[illegible]	[illegible]	[illegible]	[illegible]	[illegible]	[illegible]	[illegible]	[illegible]	[illegible]	[illegible]	[illegible]	[illegible]	[illegible]	[illegible]	[illegible]	[illegible]	[illegible]	[illegible]	[illegible]	[illegible]	[illegible]	[illegible]	[illegible]	[illegible]	[illegible]	[illegible]	[illegible]	[illegible]	[illegible]	[illegible]	[illegible]	[illegible]	[illegible]	[illegible]	[illegible]	[illegible]
M00599: Inositol-phosphate_transport_system	[illegible]	[illegible]	[illegible]	[illegible]	[illegible]	[illegible]	[illegible]	[illegible]	[illegible]	[illegible]	[illegible]	[illegible]	[illegible]	[illegible]	[illegible]	[illegible]	[illegible]	[illegible]	[illegible]	[illegible]	[illegible]	[illegible]	[illegible]	[illegible]	[illegible]	[illegible]	[illegible]	[illegible]	[illegible]	[illegible]	[illegible]	[illegible]	[illegible]	[illegible]	[illegible]	[illegible]	[illegible]	[illegible]	[illegible]	[illegible]	[illegible]	[illegible]	[illegible]	[illegible]	[illegible]	[illegible]	[illegible]	[illegible]	[illegible]	[illegible]	[illegible]	[illegible]	[illegible]	[illegible]	[illegible]	[illegible]	[illegible]	[illegible]	[illegible]	[illegible]	[illegible]	[illegible]	[illegible]	[illegible]	[illegible]	[illegible]	[illegible]
M00600: alpha-1,4-Digalacturonate_transport_system	[illegible]	[illegible]	[illegible]	[illegible]	[illegible]	[illegible]	[illegible]	[illegible]	[illegible]	[illegible]	[illegible]	[illegible]	[illegible]	[illegible]	[illegible]	[illegible]	[illegible]	[illegible]	[illegible]	[illegible]	[illegible]	[illegible]	[illegible]	[illegible]	[illegible]	[illegible]	[illegible]	[illegible]	[illegible]	[illegible]	[illegible]	[illegible]	[illegible]	[illegible]	[illegible]	[illegible]	[illegible]	[illegible]	[illegible]	[illegible]	[illegible]	[illegible]	[illegible]	[illegible]	[illegible]	[illegible]	[illegible]	[illegible]	[illegible]	[illegible]	[illegible]	[illegible]	[illegible]	[illegible]	[illegible]	[illegible]	[illegible]	[illegible]	[illegible]	[illegible]	[illegible]	[illegible]	[illegible]	[illegible]	[illegible]	[illegible]	[illegible]
M00601: Putative_chitobiose_transport_system	[illegible]	[illegible]	[illegible]	[illegible]	[illegible]	[illegible]	[illegible]	[illegible]	[illegible]	[illegible]	[illegible]	[illegible]	[illegible]	[illegible]	[illegible]	[illegible]	[illegible]	[illegible]	[illegible]	[illegible]	[illegible]	[illegible]	[illegible]	[illegible]	[illegible]	[illegible]	[illegible]	[illegible]	[illegible]	[illegible]	[illegible]	[illegible]	[illegible]	[illegible]	[illegible]	[illegible]	[illegible]	[illegible]	[illegible]	[illegible]	[illegible]	[illegible]	[illegible]	[illegible]	[illegible]	[illegible]	[illegible]	[illegible]	[illegible]	[illegible]	[illegible]	[illegible]	[illegible]	[illegible]	[illegible]	[illegible]	[illegible]	[illegible]	[illegible]	[illegible]	[illegible]	[illegible]	[illegible]	[illegible]	[illegible]	[illegible]	[illegible]
M00602: Arabinosaccharide_transport_system	[illegible]	[illegible]	[illegible]	[illegible]	[illegible]	[illegible]	[illegible]	[illegible]	[illegible]	[illegible]	[illegible]	[illegible]	[illegible]	[illegible]	[illegible]	[illegible]	[illegible]	[illegible]	[illegible]	[illegible]	[illegible]	[illegible]	[illegible]	[illegible]	[illegible]	[illegible]	[illegible]	[illegible]	[illegible]	[illegible]	[illegible]	[illegible]	[illegible]	[illegible]	[illegible]	[illegible]	[illegible]	[illegible]	[illegible]	[illegible]	[illegible]	[illegible]	[illegible]	[illegible]	[illegible]	[illegible]	[illegible]	[illegible]	[illegible]	[illegible]	[illegible]	[illegible]	[illegible]	[illegible]	[illegible]	[illegible]	[illegible]	[illegible]	[illegible]	[illegible]	[illegible]	[illegible]	[illegible]	[illegible]	[illegible]	[illegible]	[illegible]
M00603: Putative_aldouronate_transport_system	[illegible]	[illegible]	[illegible]	[illegible]	[illegible]	[illegible]	[illegible]	[illegible]	[illegible]	[illegible]	[illegible]	[illegible]	[illegible]	[illegible]	[illegible]	[illegible]	[illegible]	[illegible]	[illegible]	[illegible]	[illegible]	[illegible]	[illegible]	[illegible]	[illegible]	[illegible]	[illegible]	[illegible]	[illegible]	[illegible]	[illegible]	[illegible]	[illegible]	[illegible]	[illegible]	[illegible]	[illegible]	[illegible]	[illegible]	[illegible]	[illegible]	[illegible]	[illegible]	[illegible]	[illegible]	[illegible]	[illegible]	[illegible]	[illegible]	[illegible]	[illegible]	[illegible]	[illegible]	[illegible]	[illegible]	[illegible]	[illegible]	[illegible]	[illegible]	[illegible]	[illegible]	[illegible]	[illegible]	[illegible]	[illegible]	[illegible]	[illegible]
M00604: Trehalose_transport_system	[illegible]	[illegible]	[illegible]	[illegible]	[illegible]	[illegible]	[illegible]	[illegible]	[illegible]	[illegible]	[illegible]	[illegible]	[illegible]	[illegible]	[illegible]	[illegible]	[illegible]	[illegible]	[illegible]	[illegible]	[illegible]	[illegible]	[illegible]	[illegible]	[illegible]	[illegible]	[illegible]	[illegible]	[illegible]	[illegible]	[illegible]	[illegible]	[illegible]	[illegible]	[illegible]	[illegible]	[illegible]	[illegible]	[illegible]	[illegible]	[illegible]	[illegible]	[illegible]	[illegible]	[illegible]	[illegible]	[illegible]	[illegible]	[illegible]	[illegible]	[illegible]	[illegible]	[illegible]	[illegible]	[illegible]	[illegible]	[illegible]	[illegible]	[illegible]	[illegible]	[illegible]	[illegible]	[illegible]	[illegible]	[illegible]	[illegible]	[illegible]
M00605: Glucose/mannose_transport_system	[illegible]	[illegible]	[illegible]	[illegible]	[illegible]	[illegible]	[illegible]	[illegible]	[illegible]	[illegible]	[illegible]	[illegible]	[illegible]	[illegible]	[illegible]	[illegible]	[illegible]	[illegible]	[illegible]	[illegible]	[illegible]	[illegible]	[illegible]	[illegible]	[illegible]	[illegible]	[illegible]	[illegible]	[illegible]	[illegible]	[illegible]	[illegible]	[illegible]	[illegible]	[illegible]	[illegible]	[illegible]	[illegible]	[illegible]	[illegible]	[illegible]	[illegible]	[illegible]	[illegible]	[illegible]	[illegible]	[illegible]	[illegible]	[illegible]	[illegible]	[illegible]	[illegible]	[illegible]	[illegible]	[illegible]	[illegible]	[illegible]	[illegible]	[illegible]	[illegible]	[illegible]	[illegible]	[illegible]	[illegible]	[illegible]	[illegible]	[illegible]
M00606: N,N'-Diacetylchitobiose_transport_system	[illegible]	[illegible]	[illegible]	[illegible]	[illegible]	[illegible]	[illegible]	[illegible]	[illegible]	[illegible]	[illegible]	[illegible]	[illegible]	[illegible]	[illegible]	[illegible]	[illegible]	[illegible]	[illegible]	[illegible]	[illegible]	[illegible]	[illegible]	[illegible]	[illegible]	[illegible]	[illegible]	[illegible]	[illegible]	[illegible]	[illegible]	[illegible]	[illegible]	[illegible]	[illegible]	[illegible]	[illegible]	[illegible]	[illegible]	[illegible]	[illegible]	[illegible]	[illegible]	[illegible]	[illegible]	[illegible]	[illegible]	[illegible]	[illegible]	[illegible]	[illegible]	[illegible]	[illegible]	[illegible]	[illegible]	[illegible]	[illegible]	[illegible]	[illegible]	[illegible]	[illegible]	[illegible]	[illegible]	[illegible]	[illegible]	[illegible]	[illegible]
M00607: Glycerol_transport_system	[illegible]	[illegible]	[illegible]	[illegible]	[illegible]	[illegible]	[illegible]	[illegible]	[illegible]	[illegible]	[illegible]	[illegible]	[illegible]	[illegible]	[illegible]	[illegible]	[illegible]	[illegible]	[illegible]	[illegible]	[illegible]	[illegible]	[illegible]	[illegible]	[illegible]	[illegible]	[illegible]	[illegible]	[illegible]	[illegible]	[illegible]	[illegible]	[illegible]	[illegible]	[illegible]	[illegible]	[illegible]	[illegible]	[illegible]	[illegible]	[illegible]	[illegible]	[illegible]	[illegible]	[illegible]	[illegible]	[illegible]	[illegible]	[illegible]	[illegible]	[illegible]	[illegible]	[illegible]	[illegible]	[illegible]	[illegible]	[illegible]	[illegible]	[illegible]	[illegible]	[illegible]	[illegible]	[illegible]	[illegible]	[illegible]	[illegible]	[illegible]
M00608: 2-Oxocarboxylic_acid_chain_extension,_2-oxoglutarate_=>_2-oxoadipate_=>_2-oxopimelate_=>_2-oxosuberate	[illegible]	[illegible]	[illegible]	[illegible]	[illegible]	[illegible]	[illegible]	[illegible]	[illegible]	[illegible]	[illegible]	[illegible]	[illegible]	[illegible]	[illegible]	[illegible]	[illegible]	[illegible]	[illegible]	[illegible]	[illegible]	[illegible]	[illegible]	[illegible]	[illegible]	[illegible]	[illegible]	[illegible]	[illegible]	[illegible]	[illegible]	[illegible]	[illegible]	[illegible]	[illegible]	[illegible]	[illegible]	[illegible]	[illegible]	[illegible]	[illegible]	[illegible]	[illegible]	[illegible]	[illegible]	[illegible]	[illegible]	[illegible]	[illegible]	[illegible]	[illegible]	[illegible]	[illegible]	[illegible]	[illegible]	[illegible]	[illegible]	[illegible]	[illegible]	[illegible]	[illegible]	[illegible]	[illegible]	[illegible]	[illegible]	[illegible]	[illegible]
M00609: Cysteine_biosynthesis,_methionine_=>_cysteine	[illegible]	[illegible]	[illegible]	[illegible]	[illegible]	[illegible]	[illegible]	[illegible]	[illegible]	[illegible]	[illegible]	[illegible]	[illegible]	[illegible]	[illegible]	[illegible]	[illegible]	[illegible]	[illegible]	[illegible]	[illegible]	[illegible]	[illegible]	[illegible]	[illegible]	[illegible]	[illegible]	[illegible]	[illegible]	[illegible]	[illegible]	[illegible]	[illegible]	[illegible]	[illegible]	[illegible]	[illegible]	[illegible]	[illegible]	[illegible]	[illegible]	[illegible]	[illegible]	[illegible]	[illegible]	[illegible]	[illegible]	[illegible]	[illegible]	[illegible]	[illegible]	[illegible]	[illegible]	[illegible]	[illegible]	[illegible]	[illegible]	[illegible]	[illegible]	[illegible]	[illegible]	[illegible]	[illegible]	[illegible]	[illegible]	[illegible]	[illegible]
M00610: PTS_system,_D-glucosaminate-specific_II_component	[illegible]	[illegible]	[illegible]	[illegible]	[illegible]	[illegible]	[illegible]	[illegible]	[illegible]	[illegible]	[illegible]	[illegible]	[illegible]	[illegible]	[illegible]	[illegible]	[illegible]	[illegible]	[illegible]	[illegible]	[illegible]	[illegible]	[illegible]	[illegible]	[illegible]	[illegible]	[illegible]	[illegible]	[illegible]	[illegible]	[illegible]	[illegible]	[illegible]	[illegible]	[illegible]	[illegible]	[illegible]	[illegible]	[illegible]	[illegible]	[illegible]	[illegible]	[illegible]	[illegible]	[illegible]	[illegible]	[illegible]	[illegible]	[illegible]	[illegible]	[illegible]	[illegible]	[illegible]	[illegible]	[illegible]	[illegible]	[illegible]	[illegible]	[illegible]	[illegible]	[illegible]	[illegible]	[illegible]	[illegible]	[illegible]	[illegible]	[illegible]
M00611: Oxygenic_photosynthesis_in_plants_and_cyanobacteria	[illegible]	[illegible]	[illegible]	[illegible]	[illegible]	[illegible]	[illegible]	[illegible]	[illegible]	[illegible]	[illegible]	[illegible]	[illegible]	[illegible]	[illegible]	[illegible]	[illegible]	[illegible]	[illegible]	[illegible]	[illegible]	[illegible]	[illegible]	[illegible]	[illegible]	[illegible]	[illegible]	[illegible]	[illegible]	[illegible]	[illegible]	[illegible]	[illegible]	[illegible]	[illegible]	[illegible]	[illegible]	[illegible]	[illegible]	[illegible]	[illegible]	[illegible]	[illegible]	[illegible]	[illegible]	[illegible]	[illegible]	[illegible]	[illegible]	[illegible]	[illegible]	[illegible]	[illegible]	[illegible]	[illegible]	[illegible]	[illegible]	[illegible]	[illegible]	[illegible]	[illegible]	[illegible]	[illegible]	[illegible]	[illegible]	[illegible]	[illegible]
M00612: Anoxygenic_photosynthesis_in_purple_bacteria	[illegible]	[illegible]	[illegible]	[illegible]	[illegible]	[illegible]	[illegible]	[illegible]	[illegible]	[illegible]	[illegible]	[illegible]	[illegible]	[illegible]	[illegible]	[illegible]	[illegible]	[illegible]	[illegible]	[illegible]	[illegible]	[illegible]	[illegible]	[illegible]	[illegible]	[illegible]	[illegible]	[illegible]	[illegible]	[illegible]	[illegible]	[illegible]	[illegible]	[illegible]	[illegible]	[illegible]	[illegible]	[illegible]	[illegible]	[illegible]	[illegible]	[illegible]	[illegible]	[illegible]	[illegible]	[illegible]	[illegible]	[illegible]	[illegible]	[illegible]	[illegible]	[illegible]	[illegible]	[illegible]	[illegible]	[illegible]	[illegible]	[illegible]	[illegible]	[illegible]	[illegible]	[illegible]	[illegible]	[illegible]	[illegible]	[illegible]	[illegible]
M00613: Anoxygenic_photosynthesis_in_green_nonsulfur_bacteria	[illegible]	[illegible]	[illegible]	[illegible]	[illegible]	[illegible]	[illegible]	[illegible]	[illegible]	[illegible]	[illegible]	[illegible]	[illegible]	[illegible]	[illegible]	[illegible]	[illegible]	[illegible]	[illegible]	[illegible]	[illegible]	[illegible]	[illegible]	[illegible]	[illegible]	[illegible]	[illegible]	[illegible]	[illegible]	[illegible]	[illegible]	[illegible]	[illegible]	[illegible]	[illegible]	[illegible]	[illegible]	[illegible]	[illegible]	[illegible]	[illegible]	[illegible]	[illegible]	[illegible]	[illegible]	[illegible]	[illegible]	[illegible]	[illegible]	[illegible]	[illegible]	[illegible]	[illegible]	[illegible]	[illegible]	[illegible]	[illegible]	[illegible]	[illegible]	[illegible]	[illegible]	[illegible]	[illegible]	[illegible]	[illegible]	[illegible]	[illegible]
M00614: Anoxygenic_photosynthesis_in_green_sulfur_bacteria	[illegible]	[illegible]	[illegible]	[illegible]	[illegible]	[illegible]	[illegible]	[illegible]	[illegible]	[illegible]	[illegible]	[illegible]	[illegible]	[illegible]	[illegible]	[illegible]	[illegible]	[illegible]	[illegible]	[illegible]	[illegible]	[illegible]	[illegible]	[illegible]	[illegible]	[illegible]	[illegible]	[illegible]	[illegible]	[illegible]	[illegible]	[illegible]	[illegible]	[illegible]	[illegible]	[illegible]	[illegible]	[illegible]	[illegible]	[illegible]	[illegible]	[illegible]	[illegible]	[illegible]	[illegible]	[illegible]	[illegible]	[illegible]	[illegible]	[illegible]	[illegible]	[illegible]	[illegible]	[illegible]	[illegible]	[illegible]	[illegible]	[illegible]	[illegible]	[illegible]	[illegible]	[illegible]	[illegible]	[illegible]	[illegible]	[illegible]	[illegible]
M00615: Nitrate_assimilation	[illegible]	[illegible]	[illegible]	[illegible]	[illegible]	[illegible]	[illegible]	[illegible]	[illegible]	[illegible]	[illegible]	[illegible]	[illegible]	[illegible]	[illegible]	[illegible]	[illegible]	[illegible]	[illegible]	[illegible]	[illegible]	[illegible]	[illegible]	[illegible]	[illegible]	[illegible]	[illegible]	[illegible]	[illegible]	[illegible]	[illegible]	[illegible]	[illegible]	[illegible]	[illegible]	[illegible]	[illegible]	[illegible]	[illegible]	[illegible]	[illegible]	[illegible]	[illegible]	[illegible]	[illegible]	[illegible]	[illegible]	[illegible]	[illegible]	[illegible]	[illegible]	[illegible]	[illegible]	[illegible]	[illegible]	[illegible]	[illegible]	[illegible]	[illegible]	[illegible]	[illegible]	[illegible]	[illegible]	[illegible]	[illegible]	[illegible]	[illegible]
M00616: Sulfate-sulfur_assimilation	[illegible]	[illegible]	[illegible]	[illegible]	[illegible]	[illegible]	[illegible]	[illegible]	[illegible]	[illegible]	[illegible]	[illegible]	[illegible]	[illegible]	[illegible]	[illegible]	[illegible]	[illegible]	[illegible]	[illegible]	[illegible]	[illegible]	[illegible]	[illegible]	[illegible]	[illegible]	[illegible]	[illegible]	[illegible]	[illegible]	[illegible]	[illegible]	[illegible]	[illegible]	[illegible]	[illegible]	[illegible]	[illegible]	[illegible]	[illegible]	[illegible]	[illegible]	[illegible]	[illegible]	[illegible]	[illegible]	[illegible]	[illegible]	[illegible]	[illegible]	[illegible]	[illegible]	[illegible]	[illegible]	[illegible]	[illegible]	[illegible]	[illegible]	[illegible]	[illegible]	[illegible]	[illegible]	[illegible]	[illegible]	[illegible]	[illegible]	[illegible]
M00617: Methanogen	[illegible]	[illegible]	[illegible]	[illegible]	[illegible]	[illegible]	[illegible]	[illegible]	[illegible]	[illegible]	[illegible]	[illegible]	[illegible]	[illegible]	[illegible]	[illegible]	[illegible]	[illegible]	[illegible]	[illegible]	[illegible]	[illegible]	[illegible]	[illegible]	[illegible]	[illegible]	[illegible]	[illegible]	[illegible]	[illegible]	[illegible]	[illegible]	[illegible]	[illegible]	[illegible]	[illegible]	[illegible]	[illegible]	[illegible]	[illegible]	[illegible]	[illegible]	[illegible]	[illegible]	[illegible]	[illegible]	[illegible]	[illegible]	[illegible]	[illegible]	[illegible]	[illegible]	[illegible]	[illegible]	[illegible]	[illegible]	[illegible]	[illegible]	[illegible]	[illegible]	[illegible]	[illegible]	[illegible]	[illegible]	[illegible]	[illegible]	[illegible]
M00618: Acetogen	[illegible]	[illegible]	[illegible]	[illegible]	[illegible]	[illegible]	[illegible]	[illegible]	[illegible]	[illegible]	[illegible]	[illegible]	[illegible]	[illegible]	[illegible]	[illegible]	[illegible]	[illegible]	[illegible]	[illegible]	[illegible]	[illegible]	[illegible]	[illegible]	[illegible]	[illegible]	[illegible]	[illegible]	[illegible]	[illegible]	[illegible]	[illegible]	[illegible]	[illegible]	[illegible]	[illegible]	[illegible]	[illegible]	[illegible]	[illegible]	[illegible]	[illegible]	[illegible]	[illegible]	[illegible]	[illegible]	[illegible]	[illegible]	[illegible]	[illegible]	[illegible]	[illegible]	[illegible]	[illegible]	[illegible]	[illegible]	[illegible]	[illegible]	[illegible]	[illegible]	[illegible]	[illegible]	[illegible]	[illegible]	[illegible]	[illegible]	[illegible]
M00620: Incomplete_reductive_citrate_cycle,_acetyl-CoA_=>_oxoglutarate	[illegible]	[illegible]	[illegible]	[illegible]	[illegible]	[illegible]	[illegible]	[illegible]	[illegible]	[illegible]	[illegible]	[illegible]	[illegible]	[illegible]	[illegible]	[illegible]	[illegible]	[illegible]	[illegible]	[illegible]	[illegible]	[illegible]	[illegible]	[illegible]	[illegible]	[illegible]	[illegible]	[illegible]	[illegible]	[illegible]	[illegible]	[illegible]	[illegible]	[illegible]	[illegible]	[illegible]	[illegible]	[illegible]	[illegible]	[illegible]	[illegible]	[illegible]	[illegible]	[illegible]	[illegible]	[illegible]	[illegible]	[illegible]	[illegible]	[illegible]	[illegible]	[illegible]	[illegible]	[illegible]	[illegible]	[illegible]	[illegible]	[illegible]	[illegible]	[illegible]	[illegible]	[illegible]	[illegible]	[illegible]	[illegible]	[illegible]	[illegible]
M00622: Nicotinate_degradation,_nicotinate_=>_fumarate	[illegible]	[illegible]	[illegible]	[illegible]	[illegible]	[illegible]	[illegible]	[illegible]	[illegible]	[illegible]	[illegible]	[illegible]	[illegible]	[illegible]	[illegible]	[illegible]	[illegible]	[illegible]	[illegible]	[illegible]	[illegible]	[illegible]	[illegible]	[illegible]	[illegible]	[illegible]	[illegible]	[illegible]	[illegible]	[illegible]	[illegible]	[illegible]	[illegible]	[illegible]	[illegible]	[illegible]	[illegible]	[illegible]	[illegible]	[illegible]	[illegible]	[illegible]	[illegible]	[illegible]	[illegible]	[illegible]	[illegible]	[illegible]	[illegible]	[illegible]	[illegible]	[illegible]	[illegible]	[illegible]	[illegible]	[illegible]	[illegible]	[illegible]	[illegible]	[illegible]	[illegible]	[illegible]	[illegible]	[illegible]	[illegible]	[illegible]	[illegible]
M00623: Phthalate_degradation,_phthalate_=>_protocatechuate	[illegible]	[illegible]	[illegible]	[illegible]	[illegible]	[illegible]	[illegible]	[illegible]	[illegible]	[illegible]	[illegible]	[illegible]	[illegible]	[illegible]	[illegible]	[illegible]	[illegible]	[illegible]	[illegible]	[illegible]	[illegible]	[illegible]	[illegible]	[illegible]	[illegible]	[illegible]	[illegible]	[illegible]	[illegible]	[illegible]	[illegible]	[illegible]	[illegible]	[illegible]	[illegible]	[illegible]	[illegible]	[illegible]	[illegible]	[illegible]	[illegible]	[illegible]	[illegible]	[illegible]	[illegible]	[illegible]	[illegible]	[illegible]	[illegible]	[illegible]	[illegible]	[illegible]	[illegible]	[illegible]	[illegible]	[illegible]	[illegible]	[illegible]	[illegible]	[illegible]	[illegible]	[illegible]	[illegible]	[illegible]	[illegible]	[illegible]	[illegible]
M00624: Terephthalate_degradation,_terephthalate_=>_3,4-dihydroxybenzoate	[illegible]	[illegible]	[illegible]	[illegible]	[illegible]	[illegible]	[illegible]	[illegible]	[illegible]	[illegible]	[illegible]	[illegible]	[illegible]	[illegible]	[illegible]	[illegible]	[illegible]	[illegible]	[illegible]	[illegible]	[illegible]	[illegible]	[illegible]	[illegible]	[illegible]	[illegible]	[illegible]	[illegible]	[illegible]	[illegible]	[illegible]	[illegible]	[illegible]	[illegible]	[illegible]	[illegible]	[illegible]	[illegible]	[illegible]	[illegible]	[illegible]	[illegible]	[illegible]	[illegible]	[illegible]	[illegible]	[illegible]	[illegible]	[illegible]	[illegible]	[illegible]	[illegible]	[illegible]	[illegible]	[illegible]	[illegible]	[illegible]	[illegible]	[illegible]	[illegible]	[illegible]	[illegible]	[illegible]	[illegible]	[illegible]	[illegible]	[illegible]
M00625: Methicillin_resistance	[illegible]	[illegible]	[illegible]	[illegible]	[illegible]	[illegible]	[illegible]	[illegible]	[illegible]	[illegible]	[illegible]	[illegible]	[illegible]	[illegible]	[illegible]	[illegible]	[illegible]	[illegible]	[illegible]	[illegible]	[illegible]	[illegible]	[illegible]	[illegible]	[illegible]	[illegible]	[illegible]	[illegible]	[illegible]	[illegible]	[illegible]	[illegible]	[illegible]	[illegible]	[illegible]	[illegible]	[illegible]	[illegible]	[illegible]	[illegible]	[illegible]	[illegible]	[illegible]	[illegible]	[illegible]	[illegible]	[illegible]	[illegible]	[illegible]	[illegible]	[illegible]	[illegible]	[illegible]	[illegible]	[illegible]	[illegible]	[illegible]	[illegible]	[illegible]	[illegible]	[illegible]	[illegible]	[illegible]	[illegible]	[illegible]	[illegible]	[illegible]
M00627: beta-Lactam_resistance,_Bla_system	[illegible]	[illegible]	[illegible]	[illegible]	[illegible]	[illegible]	[illegible]	[illegible]	[illegible]	[illegible]	[illegible]	[illegible]	[illegible]	[illegible]	[illegible]	[illegible]	[illegible]	[illegible]	[illegible]	[illegible]	[illegible]	[illegible]	[illegible]	[illegible]	[illegible]	[illegible]	[illegible]	[illegible]	[illegible]	[illegible]	[illegible]	[illegible]	[illegible]	[illegible]	[illegible]	[illegible]	[illegible]	[illegible]	[illegible]	[illegible]	[illegible]	[illegible]	[illegible]	[illegible]	[illegible]	[illegible]	[illegible]	[illegible]	[illegible]	[illegible]	[illegible]	[illegible]	[illegible]	[illegible]	[illegible]	[illegible]	[illegible]	[illegible]	[illegible]	[illegible]	[illegible]	[illegible]	[illegible]	[illegible]	[illegible]	[illegible]	[illegible]
M00628: beta-Lactam_resistance,_AmpC_system	[illegible]	[illegible]	[illegible]	[illegible]	[illegible]	[illegible]	[illegible]	[illegible]	[illegible]	[illegible]	[illegible]	[illegible]	[illegible]	[illegible]	[illegible]	[illegible]	[illegible]	[illegible]	[illegible]	[illegible]	[illegible]	[illegible]	[illegible]	[illegible]	[illegible]	[illegible]	[illegible]	[illegible]	[illegible]	[illegible]	[illegible]	[illegible]	[illegible]	[illegible]	[illegible]	[illegible]	[illegible]	[illegible]	[illegible]	[illegible]	[illegible]	[illegible]	[illegible]	[illegible]	[illegible]	[illegible]	[illegible]	[illegible]	[illegible]	[illegible]	[illegible]	[illegible]	[illegible]	[illegible]	[illegible]	[illegible]	[illegible]	[illegible]	[illegible]	[illegible]	[illegible]	[illegible]	[illegible]	[illegible]	[illegible]	[illegible]	[illegible]
M00631: Other_carbohydrate_metabolism|D-Galacturonate_degradation_(bacteria)	[illegible]	[illegible]	[illegible]	[illegible]	[illegible]	[illegible]	[illegible]	[illegible]	[illegible]	[illegible]	[illegible]	[illegible]	[illegible]	[illegible]	[illegible]	[illegible]	[illegible]	[illegible]	[illegible]	[illegible]	[illegible]	[illegible]	[illegible]	[illegible]	[illegible]	[illegible]	[illegible]	[illegible]	[illegible]	[illegible]	[illegible]	[illegible]	[illegible]	[illegible]	[illegible]	[illegible]	[illegible]	[illegible]	[illegible]	[illegible]	[illegible]	[illegible]	[illegible]	[illegible]	[illegible]	[illegible]	[illegible]	[illegible]	[illegible]	[illegible]	[illegible]	[illegible]	[illegible]	[illegible]	[illegible]	[illegible]	[illegible]	[illegible]	[illegible]	[illegible]	[illegible]	[illegible]	[illegible]	[illegible]	[illegible]	[illegible]	[illegible]
M00632: Galactose_degradation,_Leloir_pathway,_galactose_=>_alpha-D-glucose-1P	[illegible]	[illegible]	[illegible]	[illegible]	[illegible]	[illegible]	[illegible]	[illegible]	[illegible]	[illegible]	[illegible]	[illegible]	[illegible]	[illegible]	[illegible]	[illegible]	[illegible]	[illegible]	[illegible]	[illegible]	[illegible]	[illegible]	[illegible]	[illegible]	[illegible]	[illegible]	[illegible]	[illegible]	[illegible]	[illegible]	[illegible]	[illegible]	[illegible]	[illegible]	[illegible]	[illegible]	[illegible]	[illegible]	[illegible]	[illegible]	[illegible]	[illegible]	[illegible]	[illegible]	[illegible]	[illegible]	[illegible]	[illegible]	[illegible]	[illegible]	[illegible]	[illegible]	[illegible]	[illegible]	[illegible]	[illegible]	[illegible]	[illegible]	[illegible]	[illegible]	[illegible]	[illegible]	[illegible]	[illegible]	[illegible]	[illegible]	[illegible]
M00633: Semi-phosphorylative_Entner-Doudoroff_pathway,_gluconate/galactonate_=>_glycerate-3P	[illegible]	[illegible]	[illegible]	[illegible]	[illegible]	[illegible]	[illegible]	[illegible]	[illegible]	[illegible]	[illegible]	[illegible]	[illegible]	[illegible]	[illegible]	[illegible]	[illegible]	[illegible]	[illegible]	[illegible]	[illegible]	[illegible]	[illegible]	[illegible]	[illegible]	[illegible]	[illegible]	[illegible]	[illegible]	[illegible]	[illegible]	[illegible]	[illegible]	[illegible]	[illegible]	[illegible]	[illegible]	[illegible]	[illegible]	[illegible]	[illegible]	[illegible]	[illegible]	[illegible]	[illegible]	[illegible]	[illegible]	[illegible]	[illegible]	[illegible]	[illegible]	[illegible]	[illegible]	[illegible]	[illegible]	[illegible]	[illegible]	[illegible]	[illegible]	[illegible]	[illegible]	[illegible]	[illegible]	[illegible]	[illegible]	[illegible]	[illegible]
M00634: Oleandomycin_transport_system	[illegible]	[illegible]	[illegible]	[illegible]	[illegible]	[illegible]	[illegible]	[illegible]	[illegible]	[illegible]	[illegible]	[illegible]	[illegible]	[illegible]	[illegible]	[illegible]	[illegible]	[illegible]	[illegible]	[illegible]	[illegible]	[illegible]	[illegible]	[illegible]	[illegible]	[illegible]	[illegible]	[illegible]	[illegible]	[illegible]	[illegible]	[illegible]	[illegible]	[illegible]	[illegible]	[illegible]	[illegible]	[illegible]	[illegible]	[illegible]	[illegible]	[illegible]	[illegible]	[illegible]	[illegible]	[illegible]	[illegible]	[illegible]	[illegible]	[illegible]	[illegible]	[illegible]	[illegible]	[illegible]	[illegible]	[illegible]	[illegible]	[illegible]	[illegible]	[illegible]	[illegible]	[illegible]	[illegible]	[illegible]	[illegible]	[illegible]	[illegible]
M00635: Tetracycline_resistance,_TetAB(46)_transporter	[illegible]	[illegible]	[illegible]	[illegible]	[illegible]	[illegible]	[illegible]	[illegible]	[illegible]	[illegible]	[illegible]	[illegible]	[illegible]	[illegible]	[illegible]	[illegible]	[illegible]	[illegible]	[illegible]	[illegible]	[illegible]	[illegible]	[illegible]	[illegible]	[illegible]	[illegible]	[illegible]	[illegible]	[illegible]	[illegible]	[illegible]	[illegible]	[illegible]	[illegible]	[illegible]	[illegible]	[illegible]	[illegible]	[illegible]	[illegible]	[illegible]	[illegible]	[illegible]	[illegible]	[illegible]	[illegible]	[illegible]	[illegible]	[illegible]	[illegible]	[illegible]	[illegible]	[illegible]	[illegible]	[illegible]	[illegible]	[illegible]	[illegible]	[illegible]	[illegible]	[illegible]	[illegible]	[illegible]	[illegible]	[illegible]	[illegible]	[illegible]
M00636: Phthalate_degradation,_phthalate_=>_protocatechuate	[illegible]	[illegible]	[illegible]	[illegible]	[illegible]	[illegible]	[illegible]	[illegible]	[illegible]	[illegible]	[illegible]	[illegible]	[illegible]	[illegible]	[illegible]	[illegible]	[illegible]	[illegible]	[illegible]	[illegible]	[illegible]	[illegible]	[illegible]	[illegible]	[illegible]	[illegible]	[illegible]	[illegible]	[illegible]	[illegible]	[illegible]	[illegible]	[illegible]	[illegible]	[illegible]	[illegible]	[illegible]	[illegible]	[illegible]	[illegible]	[illegible]	[illegible]	[illegible]	[illegible]	[illegible]	[illegible]	[illegible]	[illegible]	[illegible]	[illegible]	[illegible]	[illegible]	[illegible]	[illegible]	[illegible]	[illegible]	[illegible]	[illegible]	[illegible]	[illegible]	[illegible]	[illegible]	[illegible]	[illegible]	[illegible]	[illegible]	[illegible]
M00637: Anthranilate_degradation,_anthranilate_=>_catechol	[illegible]	[illegible]	[illegible]	[illegible]	[illegible]	[illegible]	[illegible]	[illegible]	[illegible]	[illegible]	[illegible]	[illegible]	[illegible]	[illegible]	[illegible]	[illegible]	[illegible]	[illegible]	[illegible]	[illegible]	[illegible]	[illegible]	[illegible]	[illegible]	[illegible]	[illegible]	[illegible]	[illegible]	[illegible]	[illegible]	[illegible]	[illegible]	[illegible]	[illegible]	[illegible]	[illegible]	[illegible]	[illegible]	[illegible]	[illegible]	[illegible]	[illegible]	[illegible]	[illegible]	[illegible]	[illegible]	[illegible]	[illegible]	[illegible]	[illegible]	[illegible]	[illegible]	[illegible]	[illegible]	[illegible]	[illegible]	[illegible]	[illegible]	[illegible]	[illegible]	[illegible]	[illegible]	[illegible]	[illegible]	[illegible]	[illegible]	[illegible]

Table S4. V

KEGG_Module	Habitat																																																																One-class test (strict, p < 0.01)	p value
	India oil refineries				Arctic												China oil refineries									Mangrove			Marine sediments							Taiga upper active layer				Taiga bottom active layer				Taiga permafrost layer				Oil sands core							Oil sands tailings pond upper				Oil sands tailings pond median			Oil sands tailings pond deep					
	I1	I2	I3	I4	A1	A10	A11	A12	A2	A3	A4	A5	A6	A7	A8	A9	C1	C2	C3	C4	C5	C6	C7	C8	C9	M1	M2	M3	DWH1	DWH2	DWH3	DWH4	DWH5	DWH6	DWH7	Tu1	Tu2	Tu3	Tu4	Tb1	Tb2	Tb3	Tb4	Tp1	Tp2	Tp3	Tp4	OSC1	OSC12	OSC3	OSC4	OSC5	OSC7	OSC9	OSTPu1	OSTPu2	OSTPu4	OSTPu3	OSTPm2	OSTPm6	OSTPm4	OSTPd1	OSTPd2	OSTPd3	OSTPd4		
M00638: Salicylate_degradation,_salicylate_=>_gentisate	1.34E-06	1.11E-05	3.65E-06	3.23E-06	5.42E-06	7.12E-05	9.20E-07	1.82E-06	1.16E-05	9.18E-07	0	3.31E-06	0	5.22E-06	4.75E-06	3.36E-05	1.76E-06	1.16E-05	7.89E-06	6.03E-06	9.28E-06	2.04E-05	3.27E-05	7.41E-06	1.95E-05	4.90E-06	6.68E-06	2.45E-06	0	0.000107	3.70E-06	1.42E-05	2.13E-05	8.43E-05	7.09E-05	1.94E-05	3.60E-06	2.77E-05	4.68E-06	3.66E-06	5.03E-05	3.25E-06	3.42E-06	5.41E-05	6.96E-05	1.64E-06	0.000106	9.90E-05	0.000102	0.000547	9.60E-05	8.35E-05	8.03E-05	7.47E-05	0	0	0	8.23E-06	1.93E-06	1.93E-05	1.84E-06	1.86E-06	0	2.99E-05	1.59E-05	Oil sands core	0.004412
M00639: Multidrug_resistance,_efflux_pump_MexCD-OprJ	1.34E-06	8.36E-06	1.00E-05	8.63E-05	0	2.70E-06	2.76E-06	0	7.76E-07	9.18E-07	0	4.13E-06	8.74E-07	0	0	7.81E-07	1.76E-06	1.40E-05	1.58E-06	4.52E-06	3.57E-06	3.84E-05	2.34E-06	3.37E-06	2.68E-05	8.16E-07	0	3.26E-06	0	0	0	8.86E-07	0	0	9.78E-06	3.26E-05	0	2.37E-06	0	0	0	3.25E-06	0	5.07E-06	0	0	9.50E-05	1.45E-05	1.67E-05	3.73E-05	1.45E-05	2.31E-05	3.57E-05	3.06E-05	2.45E-05	5.48E-05	3.05E-06	2.47E-05	0.000128	2.12E-05	6.45E-06	1.58E-05	4.96E-05	2.80E-05	2.59E-05		
M00641: Multidrug_resistance,_efflux_pump_MexEF-OprN	2.14E-05	7.87E-05	1.46E-05	7.42E-05	0.000127	2.52E-05	5.52E-06	1.36E-05	3.11E-06	9.18E-06	4.35E-05	1.98E-05	5.24E-06	2.61E-06	4.67E-05	6.25E-06	0.000103	0.000281	0.000143	0.00042	0.00032	0.000179	0.000282	0.000584	0.000187	0.00016	0.000117	0.000227	0.000126	0.000215	0.000228	0.000354	0.00048	0.000415	0.000353	6.62E-05	1.80E-06	1.03E-05	0	1.01E-05	4.19E-06	2.27E-05	3.42E-06	1.44E-05	2.61E-06	1.15E-05	0	5.17E-05	5.57E-05	5.00E-05	6.07E-05	6.84E-05	5.80E-05	6.12E-05	3.82E-05	5.85E-05	1.12E-05	4.32E-05	0.000226	4.33E-05	1.38E-05	0.00013	5.53E-05	5.60E-05	4.98E-05	Marine sediments	9.89E-07
M00642: Multidrug_resistance,_efflux_pump_MexJK-OprM	0.001093	0.001357	0.000764	0.001023	0.001007	0.00073	0.000664	0.000806	0.00106	0.000833	0.000927	0.000915	0.000573	0.000591	0.001052	0.001129	0.000211	0.000379	0.000242	0.000397	0.000415	0.000616	0.000378	0.000296	0.000502	0.000491	0.000492	0.000513	0.000772	0.000684	0	0.000768	0	0	0.000684	0.000662	0.000986	0.000202	0.000549	0.000495	0.001025	0.000615	0.000962	0.000951	0.000995	0.000182	0.000933	0.000802	0.000746	0.001444	0.000809	0.00082	0.000766	0.000791	0.001591	0.001742	0.00093	0.001111	0.001611	0.000867	0.001344	0.001653	0.001794	0.001192	0.001038	Oil sands tailings pond deep	3.64E-05
M00643: Multidrug_resistance,_efflux_pump_MexXY-OprM	0.001023	0.001288	0	0	0.001006	0.000639	0.000617	0.00078	0	0	0	0	0	0.000584	0.00105	0	0	0	0	0	0.000377	0.000529	0.000331	0.000256	0.000435	0.000464	0.000469	0	0	0	0	0	0	0	0	0.000427	0	0	0	0	0	0	0	0.000388	0	0	0	0.000809	0.000762	0.001189	0.00082	0.000835	0.000796	0.000782	0	0	0	0	0	0.000858	0.001321	0	0	0.001141	0.001028	Oil sands core	0.004151
M00644: Multidrug_resistance,_efflux_pump_MexHI-OprD	2.18E-05	5.57E-05	1.28E-05	7.32E-05	9.70E-05	2.16E-05	[illegible]	[illegible]	[illegible]	[illegible]	[illegible]	[illegible]	[illegible]	[illegible]	[illegible]	[illegible]	[illegible]	[illegible]	[illegible]	[illegible]	[illegible]	[illegible]	[illegible]	[illegible]	[illegible]	[illegible]	[illegible]	[illegible]	[illegible]	[illegible]	[illegible]	[illegible]	[illegible]	[illegible]	[illegible]	[illegible]	[illegible]	[illegible]	[illegible]	[illegible]	[illegible]	[illegible]	[illegible]	[illegible]	[illegible]	[illegible]	[illegible]	[illegible]	[illegible]	[illegible]	[illegible]	[illegible]	[illegible]	[illegible]	[illegible]	[illegible]	[illegible]	[illegible]	[illegible]	[illegible]	[illegible]	[illegible]	4.45E-06	4.96E-05	3.65E-05	Taiga bottom active layer	4.56E-05
M00646: Multidrug_resistance,_efflux_pump_AcrAD-TolC	0.001464	0.0016	0.001941	0.001953	0.001517	0.001248	[illegible]	[illegible]	[illegible]	[illegible]	[illegible]	[illegible]	[illegible]	[illegible]	[illegible]	[illegible]	[illegible]	[illegible]	[illegible]	[illegible]	[illegible]	[illegible]	[illegible]	[illegible]	[illegible]	[illegible]	[illegible]	[illegible]	[illegible]	[illegible]	[illegible]	[illegible]	[illegible]	[illegible]	[illegible]	[illegible]	[illegible]	[illegible]	[illegible]	[illegible]	[illegible]	[illegible]	[illegible]	[illegible]	[illegible]	[illegible]	[illegible]	[illegible]	[illegible]	[illegible]	[illegible]	[illegible]	[illegible]	[illegible]	[illegible]	[illegible]	[illegible]	[illegible]	[illegible]	[illegible]	[illegible]	[illegible]	0.002099	0.001777	0.001571	Oil sands tailings pond deep	3.15E-06
M00647: Multidrug_resistance,_efflux_pump_AcrAB-TolC/SmeDEF	0.001338	0.001496	0.001475	0.001647	0.001398	0.001097	[illegible]	[illegible]	[illegible]	[illegible]	[illegible]	[illegible]	[illegible]	[illegible]	[illegible]	[illegible]	[illegible]	[illegible]	[illegible]	[illegible]	[illegible]	[illegible]	[illegible]	[illegible]	[illegible]	[illegible]	[illegible]	[illegible]	[illegible]	[illegible]	[illegible]	[illegible]	[illegible]	[illegible]	[illegible]	[illegible]	[illegible]	[illegible]	[illegible]	[illegible]	[illegible]	[illegible]	[illegible]	[illegible]	[illegible]	[illegible]	[illegible]	[illegible]	[illegible]	[illegible]	[illegible]	[illegible]	[illegible]	[illegible]	[illegible]	[illegible]	[illegible]	[illegible]	[illegible]	[illegible]	[illegible]	[illegible]	0.001911	0.001652	0.001411	Oil sands tailings pond deep	2.89E-06
M00648: Multidrug_resistance,_efflux_pump_MdtABC	0.001139	0.000827	0.000417	0.000424	0.000302	0.000297	[illegible]	[illegible]	[illegible]	[illegible]	[illegible]	[illegible]	[illegible]	[illegible]	[illegible]	[illegible]	[illegible]	[illegible]	[illegible]	[illegible]	[illegible]	[illegible]	[illegible]	[illegible]	[illegible]	[illegible]	[illegible]	[illegible]	[illegible]	[illegible]	[illegible]	[illegible]	[illegible]	[illegible]	[illegible]	[illegible]	[illegible]	[illegible]	[illegible]	[illegible]	[illegible]	[illegible]	[illegible]	[illegible]	[illegible]	[illegible]	[illegible]	[illegible]	[illegible]	[illegible]	[illegible]	[illegible]	[illegible]	[illegible]	[illegible]	[illegible]	[illegible]	[illegible]	[illegible]	[illegible]	[illegible]	[illegible]	0.00075	0.000512	0.000373		
M00649: Multidrug_resistance,_efflux_pump_AdeABC	0.000113	2.60E-05	3.59E-05	4.41E-05	1.55E-05	7.93E-05	[illegible]	[illegible]	[illegible]	[illegible]	[illegible]	[illegible]	[illegible]	[illegible]	[illegible]	[illegible]	[illegible]	[illegible]	[illegible]	[illegible]	[illegible]	[illegible]	[illegible]	[illegible]	[illegible]	[illegible]	[illegible]	[illegible]	[illegible]	[illegible]	[illegible]	[illegible]	[illegible]	[illegible]	[illegible]	[illegible]	[illegible]	[illegible]	[illegible]	[illegible]	[illegible]	[illegible]	[illegible]	[illegible]	[illegible]	[illegible]	[illegible]	[illegible]	[illegible]	[illegible]	[illegible]	[illegible]	[illegible]	[illegible]	[illegible]	[illegible]	[illegible]	[illegible]	[illegible]	[illegible]	[illegible]	[illegible]	0	4.96E-05	2.86E-05	Oil sands core	0.003185
M00651: Vancomycin_resistance,_D-Ala-D-Lac_type	7.55E-05	3.95E-05	0.000118	6.08E-05	0.000216	0.000355	[illegible]	[illegible]	[illegible]	[illegible]	[illegible]	[illegible]	[illegible]	[illegible]	[illegible]	[illegible]	[illegible]	[illegible]	[illegible]	[illegible]	[illegible]	[illegible]	[illegible]	[illegible]	[illegible]	[illegible]	[illegible]	[illegible]	[illegible]	[illegible]	[illegible]	[illegible]	[illegible]	[illegible]	[illegible]	[illegible]	[illegible]	[illegible]	[illegible]	[illegible]	[illegible]	[illegible]	[illegible]	[illegible]	[illegible]	[illegible]	[illegible]	[illegible]	[illegible]	[illegible]	[illegible]	[illegible]	[illegible]	[illegible]	[illegible]	[illegible]	[illegible]	[illegible]	[illegible]	[illegible]	[illegible]	[illegible]	3.50E-05	3.77E-05	5.55E-05	Arctic	8.42E-06
M00652: Vancomycin_resistance,_D-Ala-D-Ser_type	0	0	0	0	2.06E-06	1.20E-06	[illegible]	[illegible]	[illegible]	[illegible]	[illegible]	[illegible]	[illegible]	[illegible]	[illegible]	[illegible]	[illegible]	[illegible]	[illegible]	[illegible]	[illegible]	[illegible]	[illegible]	[illegible]	[illegible]	[illegible]	[illegible]	[illegible]	[illegible]	[illegible]	[illegible]	[illegible]	[illegible]	[illegible]	[illegible]	[illegible]	[illegible]	[illegible]	[illegible]	[illegible]	[illegible]	[illegible]	[illegible]	[illegible]	[illegible]	[illegible]	[illegible]	[illegible]	[illegible]	[illegible]	[illegible]	[illegible]	[illegible]	[illegible]	[illegible]	[illegible]	[illegible]	[illegible]	[illegible]	[illegible]	[illegible]	[illegible]	0	1.29E-06	1.33E-06		
M00653: AauS-AauR_(acidic_amino_acids_utilization)_two-component_regulatory_system	0	0	0	0	0	1.80E-06	[illegible]	[illegible]	[illegible]	[illegible]	[illegible]	[illegible]	[illegible]	[illegible]	[illegible]	[illegible]	[illegible]	[illegible]	[illegible]	[illegible]	[illegible]	[illegible]	[illegible]	[illegible]	[illegible]	[illegible]	[illegible]	[illegible]	[illegible]	[illegible]	[illegible]	[illegible]	[illegible]	[illegible]	[illegible]	[illegible]	[illegible]	[illegible]	[illegible]	[illegible]	[illegible]	[illegible]	[illegible]	[illegible]	[illegible]	[illegible]	[illegible]	[illegible]	[illegible]	[illegible]	[illegible]	[illegible]	[illegible]	[illegible]	[illegible]	[illegible]	[illegible]	[illegible]	[illegible]	[illegible]	[illegible]	[illegible]	5.73E-06	1.35E-05	1.79E-05		
M00654: ParS-ParR_(polymyxin-adaptive_resistance)_two-component_regulatory_system	0	0	0	0	0	1.80E-06	[illegible]	[illegible]	[illegible]	[illegible]	[illegible]	[illegible]	[illegible]	[illegible]	[illegible]	[illegible]	[illegible]	[illegible]	[illegible]	[illegible]	[illegible]	[illegible]	[illegible]	[illegible]	[illegible]	[illegible]	[illegible]	[illegible]	[illegible]	[illegible]	[illegible]	[illegible]	[illegible]	[illegible]	[illegible]	[illegible]	[illegible]	[illegible]	[illegible]	[illegible]	[illegible]	[illegible]	[illegible]	[illegible]	[illegible]	[illegible]	[illegible]	[illegible]	[illegible]	[illegible]	[illegible]	[illegible]	[illegible]	[illegible]	[illegible]	[illegible]	[illegible]	[illegible]	[illegible]	[illegible]	[illegible]	[illegible]	5.73E-06	1.35E-05	1.79E-05		
M00655: AdeS-AdeR_two-component_regulatory_system	0.000118	2.79E-05	6.57E-05	4.84E-05	2.01E-05	[illegible]	[illegible]	[illegible]	[illegible]	[illegible]	[illegible]	[illegible]	[illegible]	[illegible]	[illegible]	[illegible]	[illegible]	[illegible]	[illegible]	[illegible]	[illegible]	[illegible]	[illegible]	[illegible]	[illegible]	[illegible]	[illegible]	[illegible]	[illegible]	[illegible]	[illegible]	[illegible]	[illegible]	[illegible]	[illegible]	[illegible]	[illegible]	[illegible]	[illegible]	[illegible]	[illegible]	[illegible]	[illegible]	[illegible]	[illegible]	[illegible]	[illegible]	[illegible]	[illegible]	[illegible]	[illegible]	[illegible]	[illegible]	[illegible]	[illegible]	[illegible]	[illegible]	[illegible]	[illegible]	[illegible]	[illegible]	[illegible]	0	6.96E-05	5.18E-05	Oil sands core	0.003717
M00656: VanS-VanR_(VanB_type_vancomycin_resistance)_two-component_regulatory_system	0	0	0	0	0	[illegible]	[illegible]	[illegible]	[illegible]	[illegible]	[illegible]	[illegible]	[illegible]	[illegible]	[illegible]	[illegible]	[illegible]	[illegible]	[illegible]	[illegible]	[illegible]	[illegible]	[illegible]	[illegible]	[illegible]	[illegible]	[illegible]	[illegible]	[illegible]	[illegible]	[illegible]	[illegible]	[illegible]	[illegible]	[illegible]	[illegible]	[illegible]	[illegible]	[illegible]	[illegible]	[illegible]	[illegible]	[illegible]	[illegible]	[illegible]	[illegible]	[illegible]	[illegible]	[illegible]	[illegible]	[illegible]	[illegible]	[illegible]	[illegible]	[illegible]	[illegible]	[illegible]	[illegible]	[illegible]	[illegible]	[illegible]	[illegible]	0	0	0		
M00657: VanS-VanR_(VanE_type_vancomycin_resistance)_two-component_regulatory_system	0	0	0	0	3.09E-06	[illegible]	[illegible]	[illegible]	[illegible]	[illegible]	[illegible]	[illegible]	[illegible]	[illegible]	[illegible]	[illegible]	[illegible]	[illegible]	[illegible]	[illegible]	[illegible]	[illegible]	[illegible]	[illegible]	[illegible]	[illegible]	[illegible]	[illegible]	[illegible]	[illegible]	[illegible]	[illegible]	[illegible]	[illegible]	[illegible]	[illegible]	[illegible]	[illegible]	[illegible]	[illegible]	[illegible]	[illegible]	[illegible]	[illegible]	[illegible]	[illegible]	[illegible]	[illegible]	[illegible]	[illegible]	[illegible]	[illegible]	[illegible]	[illegible]	[illegible]	[illegible]	[illegible]	[illegible]	[illegible]	[illegible]	[illegible]	[illegible]	0	1.93E-06	1.99E-06		
M00658: VanS-VanR_(actinomycete_type_vancomycin_resistance)_two-component_regulatory_system	9.22E-05	2.37E-05	4.01E-05	9.68E-06	0.000311	[illegible]	[illegible]	[illegible]	[illegible]	[illegible]	[illegible]	[illegible]	[illegible]	[illegible]	[illegible]	[illegible]	[illegible]	[illegible]	[illegible]	[illegible]	[illegible]	[illegible]	[illegible]	[illegible]	[illegible]	[illegible]	[illegible]	[illegible]	[illegible]	[illegible]	[illegible]	[illegible]	[illegible]	[illegible]	[illegible]	[illegible]	[illegible]	[illegible]	[illegible]	[illegible]	[illegible]	[illegible]	[illegible]	[illegible]	[illegible]	[illegible]	[illegible]	[illegible]	[illegible]	[illegible]	[illegible]	[illegible]	[illegible]	[illegible]	[illegible]	[illegible]	[illegible]	[illegible]	[illegible]	[illegible]	[illegible]	[illegible]	0	3.86E-06	0	Arctic	1.98E-06
M00660: Xanthomonas_spp_pathogenicity_signature,_T3SS_and_effectors	2.09E-05	2.09E-05	7.09E-06	2.28E-05	2.68E-05	[illegible]	[illegible]	[illegible]	[illegible]	[illegible]	[illegible]	[illegible]	[illegible]	[illegible]	[illegible]	[illegible]	[illegible]	[illegible]	[illegible]	[illegible]	[illegible]	[illegible]	[illegible]	[illegible]	[illegible]	[illegible]	[illegible]	[illegible]	[illegible]	[illegible]	[illegible]	[illegible]	[illegible]	[illegible]	[illegible]	[illegible]	[illegible]	[illegible]	[illegible]	[illegible]	[illegible]	[illegible]	[illegible]	[illegible]	[illegible]	[illegible]	[illegible]	[illegible]	[illegible]	[illegible]	[illegible]	[illegible]	[illegible]	[illegible]	[illegible]	[illegible]	[illegible]	[illegible]	[illegible]	[illegible]	[illegible]	[illegible]	3.50E-05	9.19E-05	4.05E-05	Oil sands core	7.16E-05
M00664: Nodulation	2.67E-06	2.79E-06	1.82E-06	0	0	[illegible]	[illegible]	[illegible]	[illegible]	[illegible]	[illegible]	[illegible]	[illegible]	[illegible]	[illegible]	[illegible]	[illegible]	[illegible]	[illegible]	[illegible]	[illegible]	[illegible]	[illegible]	[illegible]	[illegible]	[illegible]	[illegible]	[illegible]	[illegible]	[illegible]	[illegible]	[illegible]	[illegible]	[illegible]	[illegible]	[illegible]	[illegible]	[illegible]	[illegible]	[illegible]	[illegible]	[illegible]	[illegible]	[illegible]	[illegible]	[illegible]	[illegible]	[illegible]	[illegible]	[illegible]	[illegible]	[illegible]	[illegible]	[illegible]	[illegible]	[illegible]	[illegible]	[illegible]	[illegible]	[illegible]	[illegible]	[illegible]	0	5.41E-05	3.79E-05	Oil sands core	0.000869
M00667: Tetracycline_resistance,_efflux_pump	0	0	0	0	0	[illegible]	[illegible]	[illegible]	[illegible]	[illegible]	[illegible]	[illegible]	[illegible]	[illegible]	[illegible]	[illegible]	[illegible]	[illegible]	[illegible]	[illegible]	[illegible]	[illegible]	[illegible]	[illegible]	[illegible]	[illegible]	[illegible]	[illegible]	[illegible]	[illegible]	[illegible]	[illegible]	[illegible]	[illegible]	[illegible]	[illegible]	[illegible]	[illegible]	[illegible]	[illegible]	[illegible]	[illegible]	[illegible]	[illegible]	[illegible]	[illegible]	[illegible]	[illegible]	[illegible]	[illegible]	[illegible]	[illegible]	[illegible]	[illegible]	[illegible]	[illegible]	[illegible]	[illegible]	[illegible]	[illegible]	[illegible]	[illegible]	0	0	0		
M00668: Tetracycline_resistance,_TetA_transporter	0.000325	0.000255	0.000305	0.000303	0.000664	[illegible]	[illegible]	[illegible]	[illegible]	[illegible]	[illegible]	[illegible]	[illegible]	[illegible]	[illegible]	[illegible]	[illegible]	[illegible]	[illegible]	[illegible]	[illegible]	[illegible]	[illegible]	[illegible]	[illegible]	[illegible]	[illegible]	[illegible]	[illegible]	[illegible]	[illegible]	[illegible]	[illegible]	[illegible]	[illegible]	[illegible]	[illegible]	[illegible]	[illegible]	[illegible]	[illegible]	[illegible]	[illegible]	[illegible]	[illegible]	[illegible]	[illegible]	[illegible]	[illegible]	[illegible]	[illegible]	[illegible]	[illegible]	[illegible]	[illegible]	[illegible]	[illegible]	[illegible]	[illegible]	[illegible]	[illegible]	[illegible]	5.73E-06	0.000249	0.000106	Taiga bottom active layer	9.59E-07
M00669: gamma-Hexachlorocyclohexane_transport_system	0.002996	0.002991	0.003662	0.003457	0.002859	[illegible]	[illegible]	[illegible]	[illegible]	[illegible]	[illegible]	[illegible]	[illegible]	[illegible]	[illegible]	[illegible]	[illegible]	[illegible]	[illegible]	[illegible]	[illegible]	[illegible]	[illegible]	[illegible]	[illegible]	[illegible]	[illegible]	[illegible]	[illegible]	[illegible]	[illegible]	[illegible]	[illegible]	[illegible]	[illegible]	[illegible]	[illegible]	[illegible]	[illegible]	[illegible]	[illegible]	[illegible]	[illegible]	[illegible]	[illegible]	[illegible]	[illegible]	[illegible]	[illegible]	[illegible]	[illegible]	[illegible]	[illegible]	[illegible]	[illegible]	[illegible]	[illegible]	[illegible]	[illegible]	[illegible]	[illegible]	[illegible]	0.004001	0.003097	0.002644	Oil sands tailings pond deep	0.003198
M00670: Mce_transport_system	0.002996	0.002991	0.003662	0.003457	0.002859	[illegible]	[illegible]	[illegible]	[illegible]	[illegible]	[illegible]	[illegible]	[illegible]	[illegible]	[illegible]	[illegible]	[illegible]	[illegible]	[illegible]	[illegible]	[illegible]	[illegible]	[illegible]	[illegible]	[illegible]	[illegible]	[illegible]	[illegible]	[illegible]	[illegible]	[illegible]	[illegible]	[illegible]	[illegible]	[illegible]	[illegible]	[illegible]	[illegible]	[illegible]	[illegible]	[illegible]	[illegible]	[illegible]	[illegible]	[illegible]	[illegible]	[illegible]	[illegible]	[illegible]	[illegible]	[illegible]	[illegible]	[illegible]	[illegible]	[illegible]	[illegible]	[illegible]	[illegible]	[illegible]	[illegible]	[illegible]	[illegible]	0.004001	0.003097	0.002644	Oil sands tailings pond deep	0.003198
M00673: Cephamycin_C_biosynthesis,_aminoadipate_+_cysteine_+_valine_=>_cephamycin_C	9.68E-06	1.32E-05	3.33E-05	2.14E-05	1.20E-05	[illegible]	[illegible]	[illegible]	[illegible]	[illegible]	[illegible]	[illegible]	[illegible]	[illegible]	[illegible]	[illegible]	[illegible]	[illegible]	[illegible]	[illegible]	[illegible]	[illegible]	[illegible]	[illegible]	[illegible]	[illegible]	[illegible]	[illegible]	[illegible]	[illegible]	[illegible]	[illegible]	[illegible]	[illegible]	[illegible]	[illegible]	[illegible]	[illegible]	[illegible]	[illegible]	[illegible]	[illegible]	[illegible]	[illegible]	[illegible]	[illegible]	[illegible]	[illegible]	[illegible]	[illegible]	[illegible]	[illegible]	[illegible]	[illegible]	[illegible]	[illegible]	[illegible]	[illegible]	[illegible]	[illegible]	[illegible]	[illegible]	1.43E-06	1.06E-05	3.29E-05		
M00694: cGMP_signaling	0	0	0	3.23E-06	0	[illegible]	[illegible]	[illegible]	[illegible]	[illegible]	[illegible]	[illegible]	[illegible]	[illegible]	[illegible]	[illegible]	[illegible]	[illegible]	[illegible]	[illegible]	[illegible]	[illegible]	[illegible]	[illegible]	[illegible]	[illegible]	[illegible]	[illegible]	[illegible]	[illegible]	[illegible]	[illegible]	[illegible]	[illegible]	[illegible]	[illegible]	[illegible]	[illegible]	[illegible]	[illegible]	[illegible]	[illegible]	[illegible]	[illegible]	[illegible]	[illegible]	[illegible]	[illegible]	[illegible]	[illegible]	[illegible]	[illegible]	[illegible]	[illegible]	[illegible]	[illegible]	[illegible]	[illegible]	[illegible]	[illegible]	[illegible]	[illegible]	0	0	0		
M00695: cAMP_signaling	0.000188	0.000215	0.000254	0.000263	0.000185	[illegible]	[illegible]	[illegible]	[illegible]	[illegible]	[illegible]	[illegible]	[illegible]	[illegible]	[illegible]	[illegible]	[illegible]	[illegible]	[illegible]	[illegible]	[illegible]	[illegible]	[illegible]	[illegible]	[illegible]	[illegible]	[illegible]	[illegible]	[illegible]	[illegible]	[illegible]	[illegible]	[illegible]	[illegible]	[illegible]	[illegible]	[illegible]	[illegible]	[illegible]	[illegible]	[illegible]	[illegible]	[illegible]	[illegible]	[illegible]	[illegible]	[illegible]	[illegible]	[illegible]	[illegible]	[illegible]	[illegible]	[illegible]	[illegible]	[illegible]	[illegible]	[illegible]	[illegible]	[illegible]	[illegible]	[illegible]	[illegible]	0.000216	0.00024	0.000251	China oil refineries	0.000523
M00696: Multidrug_resistance,_efflux_pump_AcrEF-TolC	0	0	0	0	0	[illegible]	[illegible]	[illegible]	[illegible]	[illegible]	[illegible]	[illegible]	[illegible]	[illegible]	[illegible]	[illegible]	[illegible]	[illegible]	[illegible]	[illegible]	[illegible]	[illegible]	[illegible]	[illegible]	[illegible]	[illegible]	[illegible]	[illegible]	[illegible]	[illegible]	[illegible]	[illegible]	[illegible]	[illegible]	[illegible]	[illegible]	[illegible]	[illegible]	[illegible]	[illegible]	[illegible]	[illegible]	[illegible]	[illegible]	[illegible]	[illegible]	[illegible]	[illegible]	[illegible]	[illegible]	[illegible]	[illegible]	[illegible]	[illegible]	[illegible]	[illegible]	[illegible]	[illegible]	[illegible]	[illegible]	[illegible]	[illegible]	0	0	0	Oil sands core	1.30E-07
M00697: Multidrug_resistance,_efflux_pump_MdtEF-TolC	0.00093	0.000916	0.001018	0.000984	0.000733	[illegible]	[illegible]	[illegible]	[illegible]	[illegible]	[illegible]	[illegible]	[illegible]	[illegible]	[illegible]	[illegible]	[illegible]	[illegible]	[illegible]	[illegible]	[illegible]	[illegible]	[illegible]	[illegible]	[illegible]	[illegible]	[illegible]	[illegible]	[illegible]	[illegible]	[illegible]	[illegible]	[illegible]	[illegible]	[illegible]	[illegible]	[illegible]	[illegible]	[illegible]	[illegible]	[illegible]	[illegible]	[illegible]	[illegible]	[illegible]	[illegible]	[illegible]	[illegible]	[illegible]	[illegible]	[illegible]	[illegible]	[illegible]	[illegible]	[illegible]	[illegible]	[illegible]	[illegible]	[illegible]	[illegible]	[illegible]	[illegible]	0.001098	0.000968	0.000646	Oil sands core	0.00049
M00698: Multidrug_resistance,_efflux_pump_BpeEF-OprC	0.000212	0.000649	0.000431	0.000675	0.000767	[illegible]	[illegible]	[illegible]	[illegible]	[illegible]	[illegible]	[illegible]	[illegible]	[illegible]	[illegible]	[illegible]	[illegible]	[illegible]	[illegible]	[illegible]	[illegible]	[illegible]	[illegible]	[illegible]	[illegible]	[illegible]	[illegible]	[illegible]	[illegible]	[illegible]	[illegible]	[illegible]	[illegible]	[illegible]	[illegible]	[illegible]	[illegible]	[illegible]	[illegible]	[illegible]	[illegible]	[illegible]	[illegible]	[illegible]	[illegible]	[illegible]	[illegible]	[illegible]	[illegible]	[illegible]	[illegible]	[illegible]	[illegible]	[illegible]	[illegible]	[illegible]	[illegible]	[illegible]	[illegible]	[illegible]	[illegible]	[illegible]	0.000814	0.000753	0.000277	Taiga bottom active layer	0.000203
M00699: Multidrug_resistance,_efflux_pump_AmeABC	0.002036	0.00214	0.002525	0.002921	0.002496	[illegible]	[illegible]	[illegible]	[illegible]	[illegible]	[illegible]	[illegible]	[illegible]	[illegible]	[illegible]	[illegible]	[illegible]	[illegible]	[illegible]	[illegible]	[illegible]	[illegible]	[illegible]	[illegible]	[illegible]	[illegible]	[illegible]	[illegible]	[illegible]	[illegible]	[illegible]	[illegible]	[illegible]	[illegible]	[illegible]	[illegible]	[illegible]	[illegible]	[illegible]	[illegible]	[illegible]	[illegible]	[illegible]	[illegible]	[illegible]	[illegible]	[illegible]	[illegible]	[illegible]	[illegible]	[illegible]	[illegible]	[illegible]	[illegible]	[illegible]	[illegible]	[illegible]	[illegible]	[illegible]	[illegible]	[illegible]	[illegible]	0.002777	0.00261	0.002492	Marine sediments	5.36E-06
M00700: Multidrug_resistance,_efflux_pump_AbcA	0	1.39E-06	1.82E-06	0	0	[illegible]	[illegible]	[illegible]	[illegible]	[illegible]	[illegible]	[illegible]	[illegible]	[illegible]	[illegible]	[illegible]	[illegible]	[illegible]	[illegible]	[illegible]	[illegible]	[illegible]	[illegible]	[illegible]	[illegible]	[illegible]	[illegible]	[illegible]	[illegible]	[illegible]	[illegible]	[illegible]	[illegible]	[illegible]	[illegible]	[illegible]	[illegible]	[illegible]	[illegible]	[illegible]	[illegible]	[illegible]	[illegible]	[illegible]	[illegible]	[illegible]	[illegible]	[illegible]	[illegible]	[illegible]	[illegible]	[illegible]	[illegible]	[illegible]	[illegible]	[illegible]	[illegible]	[illegible]	[illegible]	[illegible]	[illegible]	[illegible]	0	0	5.98E-06		
M00701: Multidrug_resistance,_efflux_pump_EmrAB	0.003778	0.002598	0.00132	0.001588	0.001054	[illegible]	[illegible]	[illegible]	[illegible]	[illegible]	[illegible]	[illegible]	[illegible]	[illegible]	[illegible]	[illegible]	[illegible]	[illegible]	[illegible]	[illegible]	[illegible]	[illegible]	[illegible]	[illegible]	[illegible]	[illegible]	[illegible]	[illegible]	[illegible]	[illegible]	[illegible]	[illegible]	[illegible]	[illegible]	[illegible]	[illegible]	[illegible]	[illegible]	[illegible]	[illegible]	[illegible]	[illegible]	[illegible]	[illegible]	[illegible]	[illegible]	[illegible]	[illegible]	[illegible]	[illegible]	[illegible]	[illegible]	[illegible]	[illegible]	[illegible]	[illegible]	[illegible]	[illegible]	[illegible]	[illegible]	[illegible]	[illegible]	0.000914	0.001676	0.0012	Taiga bottom active layer	0.004326
M00702: Multidrug_resistance,_efflux_pump_NorB	0	0	6.11E-05	8.07E-07	1.55E-06	[illegible]	[illegible]	[illegible]	[illegible]	[illegible]	[illegible]	[illegible]	[illegible]	[illegible]	[illegible]	[illegible]	[illegible]	[illegible]	[illegible]	[illegible]	[illegible]	[illegible]	[illegible]	[illegible]	[illegible]	[illegible]	[illegible]	[illegible]	[illegible]	[illegible]	[illegible]	[illegible]	[illegible]	[illegible]	[illegible]	[illegible]	[illegible]	[illegible]	[illegible]	[illegible]	[illegible]	[illegible]	[illegible]	[illegible]	[illegible]	[illegible]	[illegible]	[illegible]	[illegible]	[illegible]	[illegible]	[illegible]	[illegible]	[illegible]	[illegible]	[illegible]	[illegible]	[illegible]	[illegible]	[illegible]	[illegible]	[illegible]	0	1.35E-05	1.20E-05		
M00704: Tetracycline_resistance,_efflux_pump_Tet38	0	0	0	0	0	[illegible]	[illegible]	[illegible]	[illegible]	[illegible]	[illegible]	[illegible]	[illegible]	[illegible]	[illegible]	[illegible]	[illegible]	[illegible]	[illegible]	[illegible]	[illegible]	[illegible]	[illegible]	[illegible]	[illegible]	[illegible]	[illegible]	[illegible]	[illegible]	[illegible]	[illegible]	[illegible]	[illegible]	[illegible]	[illegible]	[illegible]	[illegible]	[illegible]	[illegible]	[illegible]	[illegible]	[illegible]	[illegible]	[illegible]	[illegible]	[illegible]	[illegible]	[illegible]	[illegible]	[illegible]	[illegible]	[illegible]	[illegible]	[illegible]	[illegible]	[illegible]	[illegible]	[illegible]	[illegible]	[illegible]	[illegible]	[illegible]	0	3.86E-06	0	Taiga upper active layer	0.000162
M00705: Multidrug_resistance,_efflux_pump_MepA	0	0	0	0	0	[illegible]	[illegible]	[illegible]	[illegible]	[illegible]	[illegible]	[illegible]	[illegible]	[illegible]	[illegible]	[illegible]	[illegible]	[illegible]	[illegible]	[illegible]	[illegible]	[illegible]	[illegible]	[illegible]	[illegible]	[illegible]	[illegible]	[illegible]	[illegible]	[illegible]	[illegible]	[illegible]	[illegible]	[illegible]	[illegible]	[illegible]	[illegible]	[illegible]	[illegible]	[illegible]	[illegible]	[illegible]	[illegible]	[illegible]	[illegible]	[illegible]	[illegible]	[illegible]	[illegible]	[illegible]	[illegible]	[illegible]	[illegible]	[illegible]	[illegible]	[illegible]	[illegible]	[illegible]	[illegible]	[illegible]	[illegible]	[illegible]	0	3.86E-06	0		
M00706: Multidrug_resistance,_EfrAB_transporter	0	0	0	0	0	[illegible]	[illegible]	[illegible]	[illegible]	[illegible]	[illegible]	[illegible]	[illegible]	[illegible]	[illegible]	[illegible]	[illegible]	[illegible]	[illegible]	[illegible]	[illegible]	[illegible]	[illegible]	[illegible]	[illegible]	[illegible]	[illegible]	[illegible]	[illegible]	[illegible]	[illegible]	[illegible]	[illegible]	[illegible]	[illegible]	[illegible]	[illegible]	[illegible]	[illegible]	[illegible]	[illegible]	[illegible]	[illegible]	[illegible]	[illegible]	[illegible]	[illegible]	[illegible]	[illegible]	[illegible]	[illegible]	[illegible]	[illegible]	[illegible]	[illegible]	[illegible]	[illegible]	[illegible]	[illegible]	[illegible]	[illegible]	[illegible]	0	0	1.99E-06		
M00707: Multidrug_resistance,_SmdAB/MdlAB_transporter	0	0	0	3.23E-06	0	[illegible]	[illegible]	[illegible]	[illegible]	[illegible]	[illegible]	[illegible]	[illegible]	[illegible]	[illegible]	[illegible]	[illegible]	[illegible]	[illegible]	[illegible]	[illegible]	[illegible]	[illegible]	[illegible]	[illegible]	[illegible]	[illegible]	[illegible]	[illegible]	[illegible]	[illegible]	[illegible]	[illegible]	[illegible]	[illegible]	[illegible]	[illegible]	[illegible]	[illegible]	[illegible]	[illegible]	[illegible]	[illegible]	[illegible]	[illegible]	[illegible]	[illegible]	[illegible]	[illegible]	[illegible]	[illegible]	[illegible]	[illegible]	[illegible]	[illegible]	[illegible]	[illegible]	[illegible]	[illegible]	[illegible]	[illegible]	[illegible]	0	2.32E-05	1.99E-05		
M00708: Multidrug_resistance,_PatAB_transporter	0	0	0	0	0	[illegible]	[illegible]	[illegible]	[illegible]	[illegible]	[illegible]	[illegible]	[illegible]	[illegible]	[illegible]	[illegible]	[illegible]	[illegible]	[illegible]	[illegible]	[illegible]	[illegible]	[illegible]	[illegible]	[illegible]	[illegible]	[illegible]	[illegible]	[illegible]	[illegible]	[illegible]	[illegible]	[illegible]	[illegible]	[illegible]	[illegible]	[illegible]	[illegible]	[illegible]	[illegible]	[illegible]	[illegible]	[illegible]	[illegible]	[illegible]	[illegible]	[illegible]	[illegible]	[illegible]	[illegible]	[illegible]	[illegible]	[illegible]	[illegible]	[illegible]	[illegible]	[illegible]	[illegible]	[illegible]	[illegible]	[illegible]	[illegible]	0	0	7.97E-06		
M00709: Macrolide_resistance,_MacAB-TolC_transporter	0.00109	0.000941	0.000811	0.000965	0.000862	[illegible]	[illegible]	[illegible]	[illegible]	[illegible]	[illegible]	[illegible]	[illegible]	[illegible]	[illegible]	[illegible]	[illegible]	[illegible]	[illegible]	[illegible]	[illegible]	[illegible]	[illegible]	[illegible]	[illegible]	[illegible]	[illegible]	[illegible]	[illegible]	[illegible]	[illegible]	[illegible]	[illegible]	[illegible]	[illegible]	[illegible]	[illegible]	[illegible]	[illegible]	[illegible]	[illegible]	[illegible]	[illegible]	[illegible]	[illegible]	[illegible]	[illegible]	[illegible]	[illegible]	[illegible]	[illegible]	[illegible]	[illegible]	[illegible]	[illegible]	[illegible]	[illegible]	[illegible]	[illegible]	[illegible]	[illegible]	[illegible]	0.000768	0.001053	0.000792	Oil sands core	0.000643
M00711: Multidrug_resistance,_efflux_pump_MdtIJ	0	0	0	0	0	[illegible]	[illegible]	[illegible]	[illegible]	[illegible]	[illegible]	[illegible]	[illegible]	[illegible]	[illegible]	[illegible]	[illegible]	[illegible]	[illegible]	[illegible]	[illegible]	[illegible]	[illegible]	[illegible]	[illegible]	[illegible]	[illegible]	[illegible]	[illegible]	[illegible]	[illegible]	[illegible]	[illegible]	[illegible]	[illegible]	[illegible]	[illegible]	[illegible]	[illegible]	[illegible]	[illegible]	[illegible]	[illegible]	[illegible]	[illegible]	[illegible]	[illegible]	[illegible]	[illegible]	[illegible]	[illegible]	[illegible]	[illegible]	[illegible]	[illegible]	[illegible]	[illegible]	[illegible]	[illegible]	[illegible]	[illegible]	[illegible]	0	2.32E-05	1.99E-05		
M00712: Multidrug_resistance,_efflux_pump_YkkCD	0	0	1.82E-06	0	0	[illegible]	[illegible]	[illegible]	[illegible]	[illegible]	[illegible]	[illegible]	[illegible]	[illegible]	[illegible]	[illegible]	[illegible]	[illegible]	[illegible]	[illegible]	[illegible]	[illegible]	[illegible]	[illegible]	[illegible]	[illegible]	[illegible]	[illegible]	[illegible]	[illegible]	[illegible]	[illegible]	[illegible]	[illegible]	[illegible]	[illegible]	[illegible]	[illegible]	[illegible]	[illegible]	[illegible]	[illegible]	[illegible]	[illegible]	[illegible]	[illegible]	[illegible]	[illegible]	[illegible]	[illegible]	[illegible]	[illegible]	[illegible]	[illegible]	[illegible]	[illegible]	[illegible]	[illegible]	[illegible]	[illegible]	[illegible]	[illegible]	0	3.86E-06	0		
M00713: Fluoroquinolone_resistance,_efflux_pump_LfrA	0.000116	2.79E-05	5.47E-05	2.26E-05	0.000467	[illegible]	[illegible]	[illegible]	[illegible]	[illegible]	[illegible]	[illegible]	[illegible]	[illegible]	[illegible]	[illegible]	[illegible]	[illegible]	[illegible]	[illegible]	[illegible]	[illegible]	[illegible]	[illegible]	[illegible]	[illegible]	[illegible]	[illegible]	[illegible]	[illegible]	[illegible]	[illegible]	[illegible]	[illegible]	[illegible]	[illegible]	[illegible]	[illegible]	[illegible]	[illegible]	[illegible]	[illegible]	[illegible]	[illegible]	[illegible]	[illegible]	[illegible]	[illegible]	[illegible]	[illegible]	[illegible]	[illegible]	[illegible]	[illegible]	[illegible]	[illegible]	[illegible]	[illegible]	[illegible]	[illegible]	[illegible]	[illegible]	1.91E-06	3.67E-05	5.18E-05	Arctic	4.58E-06
M00714: Multidrug_resistance,_efflux_pump_QacA	0.000116	2.79E-05	5.47E-05	2.26E-05	0.000467	[illegible]	[illegible]	[illegible]	[illegible]	[illegible]	[illegible]	[illegible]	[illegible]	[illegible]	[illegible]	[illegible]	[illegible]	[illegible]	[illegible]	[illegible]	[illegible]	[illegible]	[illegible]	[illegible]	[illegible]	[illegible]	[illegible]	[illegible]	[illegible]	[illegible]	[illegible]	[illegible]	[illegible]	[illegible]	[illegible]	[illegible]	[illegible]	[illegible]	[illegible]	[illegible]	[illegible]	[illegible]	[illegible]	[illegible]	[illegible]	[illegible]	[illegible]	[illegible]	[illegible]	[illegible]	[illegible]	[illegible]	[illegible]	[illegible]	[illegible]	[illegible]	[illegible]	[illegible]	[illegible]	[illegible]	[illegible]	[illegible]	1.91E-06	3.67E-05	5.18E-05	Arctic	4.58E-06
M00715: Lincosamide_resistance,_efflux_pump_LmrB	0.000176	4.60E-05	8.21E-05	1.94E-05	0.000534	[illegible]	[illegible]	[illegible]	[illegible]	[illegible]	[illegible]	[illegible]	[illegible]	[illegible]	[illegible]	[illegible]	[illegible]	[illegible]	[illegible]	[illegible]	[illegible]	[illegible]	[illegible]	[illegible]	[illegible]	[illegible]	[illegible]	[illegible]	[illegible]	[illegible]	[illegible]	[illegible]	[illegible]	[illegible]	[illegible]	[illegible]	[illegible]	[illegible]	[illegible]	[illegible]	[illegible]	[illegible]	[illegible]	[illegible]	[illegible]	[illegible]	[illegible]	[illegible]	[illegible]	[illegible]	[illegible]	[illegible]	[illegible]	[illegible]	[illegible]	[illegible]	[illegible]	[illegible]	[illegible]	[illegible]	[illegible]	[illegible]	1.91E-06	7.73E-06	7.97E-06	Arctic	1.24E-07
M00716: ArlS-ArlR_(virulence_regulation)_two-component_regulatory_system	0	0	0	0	0	[illegible]	[illegible]	[illegible]	[illegible]	[illegible]	[illegible]	[illegible]	[illegible]	[illegible]	[illegible]	[illegible]	[illegible]	[illegible]	[illegible]	[illegible]	[illegible]	[illegible]	[illegible]	[illegible]	[illegible]	[illegible]	[illegible]	[illegible]	[illegible]	[illegible]	[illegible]	[illegible]	[illegible]	[illegible]	[illegible]	[illegible]	[illegible]	[illegible]	[illegible]	[illegible]	[illegible]	[illegible]	[illegible]	[illegible]	[illegible]	[illegible]	[illegible]	[illegible]	[illegible]	[illegible]	[illegible]	[illegible]	[illegible]	[illegible]	[illegible]	[illegible]	[illegible]	[illegible]	[illegible]	[illegible]	[illegible]	[illegible]	0	3.86E-06	0		
M00717: Multidrug_resistance,_efflux_pump_NorA	0	0	0	0	0	[illegible]	[illegible]	[illegible]	[illegible]	[illegible]	[illegible]	[illegible]	[illegible]	[illegible]	[illegible]	[illegible]	[illegible]	[illegible]	[illegible]	[illegible]	[illegible]	[illegible]	[illegible]	[illegible]	[illegible]	[illegible]	[illegible]	[illegible]	[illegible]	[illegible]	[illegible]	[illegible]	[illegible]	[illegible]	[illegible]	[illegible]	[illegible]	[illegible]	[illegible]	[illegible]	[illegible]	[illegible]	[illegible]	[illegible]	[illegible]	[illegible]	[illegible]	[illegible]	[illegible]	[illegible]	[illegible]	[illegible]	[illegible]	[illegible]	[illegible]	[illegible]	[illegible]	[illegible]	[illegible]	[illegible]	[illegible]	[illegible]	0	3.86E-06	2.99E-06		
M00718: Multidrug_resistance,_efflux_pump_MexAB-OprM	0.00228	0.002594	0.002525	0.002921	0.002496	[illegible]	[illegible]	[illegible]	[illegible]	[illegible]	[illegible]	[illegible]	[illegible]	[illegible]	[illegible]	[illegible]	[illegible]	[illegible]	[illegible]	[illegible]	[illegible]	[illegible]	[illegible]	[illegible]	[illegible]	[illegible]	[illegible]	[illegible]	[illegible]	[illegible]	[illegible]	[illegible]	[illegible]	[illegible]	[illegible]	[illegible]	[illegible]	[illegible]	[illegible]	[illegible]	[illegible]	[illegible]	[illegible]	[illegible]	[illegible]	[illegible]	[illegible]	[illegible]	[illegible]	[illegible]	[illegible]	[illegible]	[illegible]	[illegible]	[illegible]	[illegible]	[illegible]	[illegible]	[illegible]	[illegible]	[illegible]	[illegible]	0.003099	0.002616	0.002529	Marine sediments	1.96E-06
M00720: Multidrug_resistance,_efflux_pump_VexEF-TolC	0.000936	0.000914	0.001006	0.001033	0.000606	[illegible]	[illegible]	[illegible]	[illegible]	[illegible]	[illegible]	[illegible]	[illegible]	[illegible]	[illegible]	[illegible]	[illegible]	[illegible]	[illegible]	[illegible]	[illegible]	[illegible]	[illegible]	[illegible]	[illegible]	[illegible]	[illegible]	[illegible]	[illegible]	[illegible]	[illegible]	[illegible]	[illegible]	[illegible]	[illegible]	[illegible]	[illegible]	[illegible]	[illegible]	[illegible]	[illegible]	[illegible]	[illegible]	[illegible]	[illegible]	[illegible]	[illegible]	[illegible]	[illegible]	[illegible]	[illegible]	[illegible]	[illegible]	[illegible]	[illegible]	[illegible]	[illegible]	[illegible]	[illegible]	[illegible]	[illegible]	[illegible]	0.001092	0.000884	0	India oil refineries	0.006616
M00721: Cationic_antimicrobial_peptide_(CAMP)_resistance,_arnBCADTEF_operon	0	0.000132	5.21E-05	8.18E-05	9.55E-05	[illegible]	[illegible]	[illegible]	[illegible]	[illegible]	[illegible]	[illegible]	[illegible]	[illegible]	[illegible]	[illegible]	[illegible]	[illegible]	[illegible]	[illegible]	[illegible]	[illegible]	[illegible]	[illegible]	[illegible]	[illegible]	[illegible]	[illegible]	[illegible]	[illegible]	[illegible]	[illegible]	[illegible]	[illegible]	[illegible]	[illegible]	[illegible]	[illegible]	[illegible]	[illegible]	[illegible]	[illegible]	[illegible]	[illegible]	[illegible]	[illegible]	[illegible]	[illegible]	[illegible]	[illegible]	[illegible]	[illegible]	[illegible]	[illegible]	[illegible]	[illegible]	[illegible]	[illegible]	[illegible]	[illegible]	[illegible]	[illegible]	8.75E-05	7.95E-05	8.34E-05	Marine sediments	3.64E-06
M00722: Cationic_antimicrobial_peptide_(CAMP)_resistance,_phosphoethanolamine_transferase_PmrC	0.00021	0.0004	0.000376	0.000372	0.000398	[illegible]	[illegible]	[illegible]	[illegible]	[illegible]	[illegible]	[illegible]	[illegible]	[illegible]	[illegible]	[illegible]	[illegible]	[illegible]	[illegible]	[illegible]	[illegible]	[illegible]	[illegible]	[illegible]	[illegible]	[illegible]	[illegible]	[illegible]	[illegible]	[illegible]	[illegible]	[illegible]	[illegible]	[illegible]	[illegible]	[illegible]	[illegible]	[illegible]	[illegible]	[illegible]	[illegible]	[illegible]	[illegible]	[illegible]	[illegible]	[illegible]	[illegible]	[illegible]	[illegible]	[illegible]	[illegible]	[illegible]	[illegible]	[illegible]	[illegible]	[illegible]	[illegible]	[illegible]	[illegible]	[illegible]	[illegible]	[illegible]	0.000792	0.00081	0.000427	Oil sands core	7.42E-07
M00723: Cationic_antimicrobial_peptide_(CAMP)_resistance,_phosphoethanolamine_transferase_EptB	0.000666	0.000371	0.000134	0.000244	0.000163	[illegible]	[illegible]	[illegible]	[illegible]	[illegible]	[illegible]	[illegible]	[illegible]	[illegible]	[illegible]	[illegible]	[illegible]	[illegible]	[illegible]	[illegible]	[illegible]	[illegible]	[illegible]	[illegible]	[illegible]	[illegible]	[illegible]	[illegible]	[illegible]	[illegible]	[illegible]	[illegible]	[illegible]	[illegible]	[illegible]	[illegible]	[illegible]	[illegible]	[illegible]	[illegible]	[illegible]	[illegible]	[illegible]	[illegible]	[illegible]	[illegible]	[illegible]	[illegible]	[illegible]	[illegible]	[illegible]	[illegible]	[illegible]	[illegible]	[illegible]	[illegible]	[illegible]	[illegible]	[illegible]	[illegible]	[illegible]	[illegible]	1.34E-05	4.06E-05	3.99E-05	Marine sediments	3.15E-06
M00724: Cationic_antimicrobial_peptide_(CAMP)_resistance,_palmitoyl_transferase_PagP	0.000666	0.000371	0.000134	0.000244	0.000163	[illegible]	[illegible]	[illegible]	[illegible]	[illegible]	[illegible]	[illegible]	[illegible]	[illegible]	[illegible]	[illegible]	[illegible]	[illegible]	[illegible]	[illegible]	[illegible]	[illegible]	[illegible]	[illegible]	[illegible]	[illegible]	[illegible]	[illegible]	[illegible]	[illegible]	[illegible]	[illegible]	[illegible]	[illegible]	[illegible]	[illegible]	[illegible]	[illegible]	[illegible]	[illegible]	[illegible]	[illegible]	[illegible]	[illegible]	[illegible]	[illegible]	[illegible]	[illegible]	[illegible]	[illegible]	[illegible]	[illegible]	[illegible]	[illegible]	[illegible]	[illegible]	[illegible]	[illegible]	[illegible]	[illegible]	[illegible]	[illegible]	1.34E-05	4.06E-05	3.99E-05	Marine sediments	4.99E-06
M00725: Cationic_antimicrobial_peptide_(CAMP)_resistance,_dltABCD_operon	0	0	1.82E-06	0	0	[illegible]	[illegible]	[illegible]	[illegible]	[illegible]	[illegible]	[illegible]	[illegible]	[illegible]	[illegible]	[illegible]	[illegible]	[illegible]	[illegible]	[illegible]	[illegible]	[illegible]	[illegible]	[illegible]	[illegible]	[illegible]	[illegible]	[illegible]	[illegible]	[illegible]	[illegible]	[illegible]	[illegible]	[illegible]	[illegible]	[illegible]	[illegible]	[illegible]	[illegible]	[illegible]	[illegible]	[illegible]	[illegible]	[illegible]	[illegible]	[illegible]	[illegible]	[illegible]	[illegible]	[illegible]	[illegible]	[illegible]	[illegible]	[illegible]	[illegible]	[illegible]	[illegible]	[illegible]	[illegible]	[illegible]	[illegible]	[illegible]	0	5.15E-06	9.96E-06	China oil refineries	6.45E-06
M00726: Cationic_antimicrobial_peptide_(CAMP)_resistance,_lysyl-phosphatidylglycerol_(L-PG)_synthase_MprF	0.000214	0.00016	9.58E-05	0.000167	0.000177	[illegible]	[illegible]	[illegible]	[illegible]	[illegible]	[illegible]	[illegible]	[illegible]	[illegible]	[illegible]	[illegible]	[illegible]	[illegible]	[illegible]	[illegible]	[illegible]	[illegible]	[illegible]	[illegible]	[illegible]	[illegible]	[illegible]	[illegible]	[illegible]	[illegible]	[illegible]	[illegible]	[illegible]	[illegible]	[illegible]	[illegible]	[illegible]	[illegible]	[illegible]	[illegible]	[illegible]	[illegible]	[illegible]	[illegible]	[illegible]	[illegible]	[illegible]	[illegible]	[illegible]	[illegible]	[illegible]	[illegible]	[illegible]	[illegible]	[illegible]	[illegible]	[illegible]	[illegible]	[illegible]	[illegible]	[illegible]	[illegible]	3.05E-05	8.50E-05	0.000118	Taiga permafrost layer	0.000449
M00727: Cationic_antimicrobial_peptide_(CAMP)_resistance,_N-acetylmuramoyl-L-alanine_amidase_AmiA_and_AmiC	0.001731	0.00179	0.001194	0.001245	0.0009	[illegible]	[illegible]	[illegible]	[illegible]	[illegible]	[illegible]	[illegible]	[illegible]	[illegible]	[illegible]	[illegible]	[illegible]	[illegible]	[illegible]	[illegible]	[illegible]	[illegible]	[illegible]	[illegible]	[illegible]	[illegible]	[illegible]	[illegible]	[illegible]	[illegible]	[illegible]	[illegible]	[illegible]	[illegible]	[illegible]	[illegible]	[illegible]	[illegible]	[illegible]	[illegible]	[illegible]	[illegible]	[illegible]	[illegible]	[illegible]	[illegible]	[illegible]	[illegible]	[illegible]	[illegible]	[illegible]	[illegible]	[illegible]	[illegible]	[illegible]	[illegible]	[illegible]	[illegible]	[illegible]	[illegible]	[illegible]	[illegible]	0.001175	0.000916	0.001153	India oil refineries	0.002287
M00728: Cationic_antimicrobial_peptide_(CAMP)_resistance,_envelope_protein_folding_and_degrading__factors_DegP_and_DsbA	0.001774	0.002117	0.001813	0.002167	0.001684	[illegible]	[illegible]	[illegible]	[illegible]	[illegible]	[illegible]	[illegible]	[illegible]	[illegible]	[illegible]	[illegible]	[illegible]	[illegible]	[illegible]	[illegible]	[illegible]	[illegible]	[illegible]	[illegible]	[illegible]	[illegible]	[illegible]	[illegible]	[illegible]	[illegible]	[illegible]	[illegible]	[illegible]	[illegible]	[illegible]	[illegible]	[illegible]	[illegible]	[illegible]	[illegible]	[illegible]	[illegible]	[illegible]	[illegible]	[illegible]	[illegible]	[illegible]	[illegible]	[illegible]	[illegible]	[illegible]	[illegible]	[illegible]	[illegible]	[illegible]	[illegible]	[illegible]	[illegible]	[illegible]	[illegible]	[illegible]	[illegible]	0.002208	0.001767	0.001765	China oil refineries	0.000239
M00729: Fluoroquinolone_resistance,_gyrase-protecting_protein_Qnr	0.001859	0.001779	0.001948	0.001853	0.001925	[illegible]	[illegible]	[illegible]	[illegible]	[illegible]	[illegible]	[illegible]	[illegible]	[illegible]	[illegible]	[illegible]	[illegible]	[illegible]	[illegible]	[illegible]	[illegible]	[illegible]	[illegible]	[illegible]	[illegible]	[illegible]	[illegible]	[illegible]	[illegible]	[illegible]	[illegible]	[illegible]	[illegible]	[illegible]	[illegible]	[illegible]	[illegible]	[illegible]	[illegible]	[illegible]	[illegible]	[illegible]	[illegible]	[illegible]	[illegible]	[illegible]	[illegible]	[illegible]	[illegible]	[illegible]	[illegible]	[illegible]	[illegible]	[illegible]	[illegible]	[illegible]	[illegible]	[illegible]	[illegible]	[illegible]	[illegible]	[illegible]	0.001672	0.001657	0.002135	Mangrove	0.002375
M00731: Bacitracin_transport_system	0	0	0	0	0	[illegible]	[illegible]	[illegible]	[illegible]	[illegible]	[illegible]	[illegible]	[illegible]	[illegible]	[illegible]	[illegible]	[illegible]	[illegible]	[illegible]	[illegible]	[illegible]	[illegible]	[illegible]	[illegible]	[illegible]	[illegible]	[illegible]	[illegible]	[illegible]	[illegible]	[illegible]	[illegible]	[illegible]	[illegible]	[illegible]	[illegible]	[illegible]	[illegible]	[illegible]	[illegible]	[illegible]	[illegible]	[illegible]	[illegible]	[illegible]	[illegible]	[illegible]	[illegible]	[illegible]	[illegible]	[illegible]	[illegible]	[illegible]	[illegible]	[illegible]	[illegible]	[illegible]	[illegible]	[illegible]	[illegible]	[illegible]	[illegible]	0	3.86E-06	0		
M00734: BraS-BraR_(bacitracin_transport)_two-component_regulatory_system	0	0	0	0	0	[illegible]	[illegible]	[illegible]	[illegible]	[illegible]	[illegible]	[illegible]	[illegible]	[illegible]	[illegible]	[illegible]	[illegible]	[illegible]	[illegible]	[illegible]	[illegible]	[illegible]	[illegible]	[illegible]	[illegible]	[illegible]	[illegible]	[illegible]	[illegible]	[illegible]	[illegible]	[illegible]	[illegible]	[illegible]	[illegible]	[illegible]	[illegible]	[illegible]	[illegible]	[illegible]	[illegible]	[illegible]	[illegible]	[illegible]	[illegible]	[illegible]	[illegible]	[illegible]	[illegible]	[illegible]	[illegible]	[illegible]	[illegible]	[illegible]	[illegible]	[illegible]	[illegible]	[illegible]	[illegible]	[illegible]	[illegible]	[illegible]	0	3.86E-06	0		
M00736: Nocardicin_A_biosynthesis,_L-pHPG_+_arginine_+_serine_=>_nocardicin_A	1.16E-05	9.75E-06	7.30E-06	1.83E-05	3.56E-05	[illegible]	[illegible]	[illegible]	[illegible]	[illegible]	[illegible]	[illegible]	[illegible]	[illegible]	[illegible]	[illegible]	[illegible]	[illegible]	[illegible]	[illegible]	[illegible]	[illegible]	[illegible]	[illegible]	[illegible]	[illegible]	[illegible]	[illegible]	[illegible]	[illegible]	[illegible]	[illegible]	[illegible]	[illegible]	[illegible]	[illegible]	[illegible]	[illegible]	[illegible]	[illegible]	[illegible]	[illegible]	[illegible]	[illegible]	[illegible]	[illegible]	[illegible]	[illegible]	[illegible]	[illegible]	[illegible]	[illegible]	[illegible]	[illegible]	[illegible]	[illegible]	[illegible]	[illegible]	[illegible]	[illegible]	[illegible]	[illegible]	2.80E-05	7.21E-05	3.25E-05	Oil sands core	3.59E-06
M00737: Bacitracin_resistance,_VraDE_transporter	0	0	0	0	0	[illegible]	[illegible]	[illegible]	[illegible]	[illegible]	[illegible]	[illegible]	[illegible]	[illegible]	[illegible]	[illegible]	[illegible]	[illegible]	[illegible]	[illegible]	[illegible]	[illegible]	[illegible]	[illegible]	[illegible]	[illegible]	[illegible]	[illegible]	[illegible]	[illegible]	[illegible]	[illegible]	[illegible]	[illegible]	[illegible]	[illegible]	[illegible]	[illegible]	[illegible]	[illegible]	[illegible]	[illegible]	[illegible]	[illegible]	[illegible]	[illegible]	[illegible]	[illegible]	[illegible]	[illegible]	[illegible]	[illegible]	[illegible]	[illegible]	[illegible]	[illegible]	[illegible]	[illegible]	[illegible]	[illegible]	[illegible]	[illegible]	0	3.86E-06	0		
M00738: Bacitracin_resistance,_BceAB_transporter	0	0	0	0	0	[illegible]	[illegible]	[illegible]	[illegible]	[illegible]	[illegible]	[illegible]	[illegible]	[illegible]	[illegible]	[illegible]	[illegible]	[illegible]	[illegible]	[illegible]	[illegible]	[illegible]	[illegible]	[illegible]	[illegible]	[illegible]	[illegible]	[illegible]	[illegible]	[illegible]	[illegible]	[illegible]	[illegible]	[illegible]	[illegible]	[illegible]	[illegible]	[illegible]	[illegible]	[illegible]	[illegible]	[illegible]	[illegible]	[illegible]	[illegible]	[illegible]	[illegible]	[illegible]	[illegible]	[illegible]	[illegible]	[illegible]	[illegible]	[illegible]	[illegible]	[illegible]	[illegible]	[illegible]	[illegible]	[illegible]	[illegible]	[illegible]	0	0	0		
M00739: Cationic_peptide_transport_system	0	0	0	0	0	[illegible]	[illegible]	[illegible]	[illegible]	[illegible]	[illegible]	[illegible]	[illegible]	[illegible]	[illegible]	[illegible]	[illegible]	[illegible]	[illegible]	[illegible]	[illegible]	[illegible]	[illegible]	[illegible]	[illegible]	[illegible]	[illegible]	[illegible]	[illegible]	[illegible]	[illegible]	[illegible]	[illegible]	[illegible]	[illegible]	[illegible]	[illegible]	[illegible]	[illegible]	[illegible]	[illegible]	[illegible]	[illegible]	[illegible]	[illegible]	[illegible]	[illegible]	[illegible]	[illegible]	[illegible]	[illegible]	[illegible]	[illegible]	[illegible]	[illegible]	[illegible]	[illegible]	[illegible]	[illegible]	[illegible]	[illegible]	[illegible]	0	2.32E-05	1.99E-05		
M00740: Methylaspartate_cycle	0.001819	0.001694	0.001886	0.001731	0.001644	[illegible]	[illegible]	[illegible]	[illegible]	[illegible]	[illegible]	[illegible]	[illegible]	[illegible]	[illegible]	[illegible]	[illegible]	[illegible]	[illegible]	[illegible]	[illegible]	[illegible]	[illegible]	[illegible]	[illegible]	[illegible]	[illegible]	[illegible]	[illegible]	[illegible]	[illegible]	[illegible]	[illegible]	[illegible]	[illegible]	[illegible]	[illegible]	[illegible]	[illegible]	[illegible]	[illegible]	[illegible]	[illegible]	[illegible]	[illegible]	[illegible]	[illegible]	[illegible]	[illegible]	[illegible]	[illegible]	[illegible]	[illegible]	[illegible]	[illegible]	[illegible]	[illegible]	[illegible]	[illegible]	[illegible]	[illegible]	[illegible]	0.001667	0.001735	0.001588	India oil refineries	0.002529
M00741: Propanoyl-CoA_metabolism,_propanoyl-CoA_=>_succinyl-CoA	0.000745	0.000833	0.000819	0.000695	0.000883	[illegible]	[illegible]	[illegible]	[illegible]	[illegible]	[illegible]	[illegible]	[illegible]	[illegible]	[illegible]	[illegible]	[illegible]	[illegible]	[illegible]	[illegible]	[illegible]	[illegible]	[illegible]	[illegible]	[illegible]	[illegible]	[illegible]	[illegible]	[illegible]	[illegible]	[illegible]	[illegible]	[illegible]	[illegible]	[illegible]	[illegible]	[illegible]	[illegible]	[illegible]	[illegible]	[illegible]	[illegible]	[illegible]	[illegible]	[illegible]	[illegible]	[illegible]	[illegible]	[illegible]	[illegible]	[illegible]	[illegible]	[illegible]	[illegible]	[illegible]	[illegible]	[illegible]	[illegible]	[illegible]	[illegible]	[illegible]	[illegible]	0.000704	0.000596	0.000684	Taiga permafrost layer	1.69E-06
M00742: Aminoglycoside_resistance,_protease_FtsH	0.00139	0.001455	0.001634	0.001759	0.001706	[illegible]	[illegible]	[illegible]	[illegible]	[illegible]	[illegible]	[illegible]	[illegible]	[illegible]	[illegible]	[illegible]	[illegible]	[illegible]	[illegible]	[illegible]	[illegible]	[illegible]	[illegible]	[illegible]	[illegible]	[illegible]	[illegible]	[illegible]	[illegible]	[illegible]	[illegible]	[illegible]	[illegible]	[illegible]	[illegible]	[illegible]	[illegible]	[illegible]	[illegible]	[illegible]	[illegible]	[illegible]	[illegible]	[illegible]	[illegible]	[illegible]	[illegible]	[illegible]	[illegible]	[illegible]	[illegible]	[illegible]	[illegible]	[illegible]	[illegible]	[illegible]	[illegible]	[illegible]	[illegible]	[illegible]	[illegible]	[illegible]	0.001677	0.001701	0.001514	Marine sediments	7.05E-06
M00743: Aminoglycoside_resistance,_protease_HtpX	0.00079	0.001328	0.001256	0.001417	0.001043	[illegible]	[illegible]	[illegible]	[illegible]	[illegible]	[illegible]	[illegible]	[illegible]	[illegible]	[illegible]	[illegible]	[illegible]	[illegible]	[illegible]	[illegible]	[illegible]	[illegible]	[illegible]	[illegible]	[illegible]	[illegible]	[illegible]	[illegible]	[illegible]	[illegible]	[illegible]	[illegible]	[illegible]	[illegible]	[illegible]	[illegible]	[illegible]	[illegible]	[illegible]	[illegible]	[illegible]	[illegible]	[illegible]	[illegible]	[illegible]	[illegible]	[illegible]	[illegible]	[illegible]	[illegible]	[illegible]	[illegible]	[illegible]	[illegible]	[illegible]	[illegible]	[illegible]	[illegible]	[illegible]	[illegible]	[illegible]	[illegible]	0.001774	0.001691	0.001378	Oil sands core	1.11E-05
M00744: Cationic_antimicrobial_peptide_(CAMP)_resistance,_protease_PgtE	0.000666	0.000371	0.000134	0.000244	0.000163	[illegible]	[illegible]	[illegible]	[illegible]	[illegible]	[illegible]	[illegible]	[illegible]	[illegible]	[illegible]	[illegible]	[illegible]	[illegible]	[illegible]	[illegible]	[illegible]	[illegible]	[illegible]	[illegible]	[illegible]	[illegible]	[illegible]	[illegible]	[illegible]	[illegible]	[illegible]	[illegible]	[illegible]	[illegible]	[illegible]	[illegible]	[illegible]	[illegible]	[illegible]	[illegible]	[illegible]	[illegible]	[illegible]	[illegible]	[illegible]	[illegible]	[illegible]	[illegible]	[illegible]	[illegible]	[illegible]	[illegible]	[illegible]	[illegible]	[illegible]	[illegible]	[illegible]	[illegible]	[illegible]	[illegible]	[illegible]	[illegible]	1.34E-05	4.06E-05	3.99E-05	Marine sediments	3.15E-06
M00745: Imipenem_resistance,_repression_of_porin_OprD	0.000784	0.000916	0.000537	0.000653	0.000435	[illegible]	[illegible]	[illegible]	[illegible]	[illegible]	[illegible]	[illegible]	[illegible]	[illegible]	[illegible]	[illegible]	[illegible]	[illegible]	[illegible]	[illegible]	[illegible]	[illegible]	[illegible]	[illegible]	[illegible]	[illegible]	[illegible]	[illegible]	[illegible]	[illegible]	[illegible]	[illegible]	[illegible]	[illegible]	[illegible]	[illegible]	[illegible]	[illegible]	[illegible]	[illegible]	[illegible]	[illegible]	[illegible]	[illegible]	[illegible]	[illegible]	[illegible]	[illegible]	[illegible]	[illegible]	[illegible]	[illegible]	[illegible]	[illegible]	[illegible]	[illegible]	[illegible]	[illegible]	[illegible]	[illegible]	[illegible]	[illegible]	0.001214	0.000954	0.000476	Oil sands tailings pond deep	2.93E-05
M00746: Multidrug_resistance,_repression_of_porin_OmpF	0	0	0	0	0	[illegible]	[illegible]	[illegible]	[illegible]	[illegible]	[illegible]	[illegible]	[illegible]	[illegible]	[illegible]	[illegible]	[illegible]	[illegible]	[illegible]	[illegible]	[illegible]	[illegible]	[illegible]	[illegible]	[illegible]	[illegible]	[illegible]	[illegible]	[illegible]	[illegible]	[illegible]	[illegible]	[illegible]	[illegible]	[illegible]	[illegible]	[illegible]	[illegible]	[illegible]	[illegible]	[illegible]	[illegible]	[illegible]	[illegible]	[illegible]	[illegible]	[illegible]	[illegible]	[illegible]	[illegible]	[illegible]	[illegible]	[illegible]	[illegible]	[illegible]	[illegible]	[illegible]	[illegible]	[illegible]	[illegible]	[illegible]	[illegible]	0	1.16E-05	9.96E-06		
M00747: Bacitracin_transport_system	0	0	0	0	0	[illegible]	[illegible]	[illegible]	[illegible]	[illegible]	[illegible]	[illegible]	[illegible]	[illegible]	[illegible]	[illegible]	[illegible]	[illegible]	[illegible]	[illegible]	[illegible]	[illegible]	[illegible]	[illegible]	[illegible]	[illegible]	[illegible]	[illegible]	[illegible]	[illegible]	[illegible]	[illegible]	[illegible]	[illegible]	[illegible]	[illegible]	[illegible]	[illegible]	[illegible]	[illegible]	[illegible]	[illegible]	[illegible]	[illegible]	[illegible]	[illegible]	[illegible]	[illegible]	[illegible]	[illegible]	[illegible]	[illegible]	[illegible]	[illegible]	[illegible]	[illegible]	[illegible]	[illegible]	[illegible]	[illegible]	[illegible]	[illegible]	0	1.93E-06	3.99E-06		
M00754: Nisin_resistance,_phage_shock_protein_homolog_LiaH	1.40E-05	3.06E-05	5.84E-05	4.03E-05	7.74E-07	[illegible]	[illegible]	[illegible]	[illegible]	[illegible]	[illegible]	[illegible]	[illegible]	[illegible]	[illegible]	[illegible]	[illegible]	[illegible]	[illegible]	[illegible]	[illegible]	[illegible]	[illegible]	[illegible]	[illegible]	[illegible]	[illegible]	[illegible]	[illegible]	[illegible]	[illegible]	[illegible]	[illegible]	[illegible]	[illegible]	[illegible]	[illegible]	[illegible]	[illegible]	[illegible]	[illegible]	[illegible]	[illegible]	[illegible]	[illegible]	[illegible]	[illegible]	[illegible]	[illegible]	[illegible]	[illegible]	[illegible]	[illegible]	[illegible]	[illegible]	[illegible]	[illegible]	[illegible]	[illegible]	[illegible]	[illegible]	[illegible]	2.86E-06	1.93E-05	8.67E-05		
M00760: Erythromycin_resistance,_macrolide_2'-phosphotransferase_I_MphA	0	0	0	0	0	[illegible]	[illegible]	[illegible]	[illegible]	[illegible]	[illegible]	[illegible]	[illegible]	[illegible]	[illegible]	[illegible]	[illegible]	[illegible]	[illegible]	[illegible]	[illegible]	[illegible]	[illegible]	[illegible]	[illegible]	[illegible]	[illegible]	[illegible]	[illegible]	[illegible]	[illegible]	[illegible]	[illegible]	[illegible]	[illegible]	[illegible]	[illegible]	[illegible]	[illegible]	[illegible]	[illegible]	[illegible]	[illegible]	[illegible]	[illegible]	[illegible]	[illegible]	[illegible]	[illegible]	[illegible]	[illegible]	[illegible]	[illegible]	[illegible]	[illegible]	[illegible]	[illegible]	[illegible]	[illegible]	[illegible]	[illegible]	[illegible]	0	0	0		
M00761: Undecaprenylphosphate_alpha-L-Ara4N_biosynthesis,_UDP-GlcA_=>_undecaprenyl_phosphate_alpha-L-Ara4N	1.47E-05	0	4.83E-05	0	0	[illegible]	[illegible]	[illegible]	[illegible]	[illegible]	[illegible]	[illegible]	[illegible]	[illegible]	[illegible]	[illegible]	[illegible]	[illegible]	[illegible]	[illegible]	[illegible]	[illegible]	[illegible]	[illegible]	[illegible]	[illegible]	[illegible]	[illegible]	[illegible]	[illegible]	[illegible]	[illegible]	[illegible]	[illegible]	[illegible]	[illegible]	[illegible]	[illegible]	[illegible]	[illegible]	[illegible]	[illegible]	[illegible]	[illegible]	[illegible]	[illegible]	[illegible]	[illegible]	[illegible]	[illegible]	[illegible]	[illegible]	[illegible]	[illegible]	[illegible]	[illegible]	[illegible]	[illegible]	[illegible]	[illegible]	[illegible]	[illegible]	0.000281	0.000173	0.000185	Oil sands tailings pond deep	9.17E-05
M00762: Copper-processing_system	0.000182	0.000506	0.000648	0.000607	0.000422	[illegible]	[illegible]	[illegible]	[illegible]	[illegible]	[illegible]	[illegible]	[illegible]	[illegible]	[illegible]	[illegible]	[illegible]	[illegible]	[illegible]	[illegible]	[illegible]	[illegible]	[illegible]	[illegible]	[illegible]	[illegible]	[illegible]	[illegible]	[illegible]	[illegible]	[illegible]	[illegible]	[illegible]	[illegible]	[illegible]	[illegible]	[illegible]	[illegible]	[illegible]	[illegible]	[illegible]	[illegible]	[illegible]	[illegible]	[illegible]	[illegible]	[illegible]	[illegible]	[illegible]	[illegible]	[illegible]	[illegible]	[illegible]	[illegible]	[illegible]	[illegible]	[illegible]	[illegible]	[illegible]	[illegible]	[illegible]	[illegible]	0.001382	0.000854	0.000377	Oil sands tailings pond deep	2.86E-05
M00763: Ornithine_biosynthesis,_mediated_by_LysW,_glutamate_=>_ornithine	0.000288	0.000245	0.000207	0.000175	1.03E-05	[illegible]	[illegible]	[illegible]	[illegible]	[illegible]	[illegible]	[illegible]	[illegible]	[illegible]	[illegible]	[illegible]	[illegible]	[illegible]	[illegible]	[illegible]	[illegible]	[illegible]	[illegible]	[illegible]	[illegible]	[illegible]	[illegible]	[illegible]	[illegible]	[illegible]	[illegible]	[illegible]	[illegible]	[illegible]	[illegible]	[illegible]	[illegible]	[illegible]	[illegible]	[illegible]	[illegible]	[illegible]	[illegible]	[illegible]	[illegible]	[illegible]	[illegible]	[illegible]	[illegible]	[illegible]	[illegible]	[illegible]	[illegible]	[illegible]	[illegible]	[illegible]	[illegible]	[illegible]	[illegible]	[illegible]	[illegible]	[illegible]	8.91E-06	4.89E-05	0.000203	China oil refineries	7.80E-06
M00764: PTS_system,_fructoselysine_and_glucoselysine-specific_II_component	0	1.39E-06	0	0	3.09E-06	[illegible]	[illegible]	[illegible]	[illegible]	[illegible]	[illegible]	[illegible]	[illegible]	[illegible]	[illegible]	[illegible]	[illegible]	[illegible]	[illegible]	[illegible]	[illegible]	[illegible]	[illegible]	[illegible]	[illegible]	[illegible]	[illegible]	[illegible]	[illegible]	[illegible]	[illegible]	[illegible]	[illegible]	[illegible]	[illegible]	[illegible]	[illegible]	[illegible]	[illegible]	[illegible]	[illegible]	[illegible]	[illegible]	[illegible]	[illegible]	[illegible]	[illegible]	[illegible]	[illegible]	[illegible]	[illegible]	[illegible]	[illegible]	[illegible]	[illegible]	[illegible]	[illegible]	[illegible]	[illegible]	[illegible]	[illegible]	[illegible]	0	1.93E-06	4.98E-06		
M00766:Streptomycin_resistance,_deactivating_enzyme_StrAB	1.47E-05	2.79E-06	5.47E-06	6.46E-06	0.000113	[illegible]	[illegible]	[illegible]	[illegible]	[illegible]	[illegible]	[illegible]	[illegible]	[illegible]	[illegible]	[illegible]	[illegible]	[illegible]	[illegible]	[illegible]	[illegible]	[illegible]	[illegible]	[illegible]	[illegible]	[illegible]	[illegible]	[illegible]	[illegible]	[illegible]	[illegible]	[illegible]	[illegible]	[illegible]	[illegible]	[illegible]	[illegible]	[illegible]	[illegible]	[illegible]	[illegible]	[illegible]	[illegible]	[illegible]	[illegible]	[illegible]	[illegible]	[illegible]	[illegible]	[illegible]	[illegible]	[illegible]	[illegible]	[illegible]	[illegible]	[illegible]	[illegible]	[illegible]	[illegible]	[illegible]	[illegible]	[illegible]	5.73E-06	2.70E-05	1.79E-05	Taiga upper active layer	6.21E-06
M00767:Multidrug_resistance,_efflux_pump_OqxAB	6.68E-07	0	0	0	4.87E-05	[illegible]	[illegible]	[illegible]	[illegible]	[illegible]	[illegible]	[illegible]	[illegible]	[illegible]	[illegible]	[illegible]	[illegible]	[illegible]	[illegible]	[illegible]	[illegible]	[illegible]	[illegible]	[illegible]	[illegible]	[illegible]	[illegible]	[illegible]	[illegible]	[illegible]	[illegible]	[illegible]	[illegible]	[illegible]	[illegible]	[illegible]	[illegible]	[illegible]	[illegible]	[illegible]	[illegible]	[illegible]	[illegible]	[illegible]	[illegible]	[illegible]	[illegible]	[illegible]	[illegible]	[illegible]	[illegible]	[illegible]	[illegible]	[illegible]	[illegible]	[illegible]	[illegible]	[illegible]	[illegible]	[illegible]	[illegible]	[illegible]	0	1.26E-05	0	China oil refineries	8.32E-06
M00768: Multidrug_resistance,_efflux_pump_GesABC	0.001037	0.001419	0.000891	0.001018	0.001093	[illegible]	[illegible]	[illegible]	[illegible]	[illegible]	[illegible]	[illegible]	[illegible]	[illegible]	[illegible]	[illegible]	[illegible]	[illegible]	[illegible]	[illegible]	[illegible]	[illegible]	[illegible]	[illegible]	[illegible]	[illegible]	[illegible]	[illegible]	[illegible]	[illegible]	[illegible]	[illegible]	[illegible]	[illegible]	[illegible]	[illegible]	[illegible]	[illegible]	[illegible]	[illegible]	[illegible]	[illegible]	[illegible]	[illegible]	[illegible]	[illegible]	[illegible]	[illegible]	[illegible]	[illegible]	[illegible]	[illegible]	[illegible]	[illegible]	[illegible]	[illegible]	[illegible]	[illegible]	[illegible]	[illegible]	[illegible]	[illegible]	0.00184	0.001344	0.00108	Oil sands tailings pond deep	9.32E-07
M00769: Multidrug_resistance,_efflux_pump_MexPQ-OpmE	6.50E-05	0.000237	0.000303	0.000225	0.000247	[illegible]	[illegible]	[illegible]	[illegible]	[illegible]	[illegible]	[illegible]	[illegible]	[illegible]	[illegible]	[illegible]	[illegible]	[illegible]	[illegible]	[illegible]	[illegible]	[illegible]	[illegible]	[illegible]	[illegible]	[illegible]	[illegible]	[illegible]	[illegible]	[illegible]	[illegible]	[illegible]	[illegible]	[illegible]	[illegible]	[illegible]	[illegible]	[illegible]	[illegible]	[illegible]	[illegible]	[illegible]	[illegible]	[illegible]	[illegible]	[illegible]	[illegible]	[illegible]	[illegible]	[illegible]	[illegible]	[illegible]	[illegible]	[illegible]	[illegible]	[illegible]	[illegible]	[illegible]	[illegible]	[illegible]	[illegible]	[illegible]	0.00051	0.000311	9.23E-05	Oil sands tailings pond deep	4.52E-07
M00770: PfeS-PfeR_(enterobactin-dependent_iron_acquisition)_two-component_regulatory_system	0	0	0	0	0	[illegible]	[illegible]	[illegible]	[illegible]	[illegible]	[illegible]	[illegible]	[illegible]	[illegible]	[illegible]	[illegible]	[illegible]	[illegible]	[illegible]	[illegible]	[illegible]	[illegible]	[illegible]	[illegible]	[illegible]	[illegible]	[illegible]	[illegible]	[illegible]	[illegible]	[illegible]	[illegible]	[illegible]	[illegible]	[illegible]	[illegible]	[illegible]	[illegible]	[illegible]	[illegible]	[illegible]	[illegible]	[illegible]	[illegible]	[illegible]	[illegible]	[illegible]	[illegible]	[illegible]	[illegible]	[illegible]	[illegible]	[illegible]	[illegible]	[illegible]	[illegible]	[illegible]	[illegible]	[illegible]	[illegible]	[illegible]	[illegible]	5.73E-06	1.35E-05	1.99E-05		
M00771: FitF-FitH_(insecticidal_toxin_regulation)_two-component_regulatory_system_	0.000633	5.57E-05	7.30E-06	4.84E-06	1.86E-05	[illegible]	[illegible]	[illegible]	[illegible]	[illegible]	[illegible]	[illegible]	[illegible]	[illegible]	[illegible]	[illegible]	[illegible]	[illegible]	[illegible]	[illegible]	[illegible]	[illegible]	[illegible]	[illegible]	[illegible]	[illegible]	[illegible]	[illegible]	[illegible]	[illegible]	[illegible]	[illegible]	[illegible]	[illegible]	[illegible]	[illegible]	[illegible]	[illegible]	[illegible]	[illegible]	[illegible]	[illegible]	[illegible]	[illegible]	[illegible]	[illegible]	[illegible]	[illegible]	[illegible]	[illegible]	[illegible]	[illegible]	[illegible]	[illegible]	[illegible]	[illegible]	[illegible]	[illegible]	[illegible]	[illegible]	[illegible]	[illegible]	0	3.86E-06	7.97E-06	India oil refineries	0.000144
M00772: HupT-HupR_(hydrogenase_synthesis_regulation)_two-component_regulatory_system_	7.88E-05	0.000315	6.93E-05	0.000207	0.000299	[illegible]	[illegible]	[illegible]	[illegible]	[illegible]	[illegible]	[illegible]	[illegible]	[illegible]	[illegible]	[illegible]	[illegible]	[illegible]	[illegible]	[illegible]	[illegible]	[illegible]	[illegible]	[illegible]	[illegible]	[illegible]	[illegible]	[illegible]	[illegible]	[illegible]	[illegible]	[illegible]	[illegible]	[illegible]	[illegible]	[illegible]	[illegible]	[illegible]	[illegible]	[illegible]	[illegible]	[illegible]	[illegible]	[illegible]	[illegible]	[illegible]	[illegible]	[illegible]	[illegible]	[illegible]	[illegible]	[illegible]	[illegible]	[illegible]	[illegible]	[illegible]	[illegible]	[illegible]	[illegible]	[illegible]	[illegible]	[illegible]	0.000563	0.000209	5.18E-05		
M00773: Tylosin_biosynthesis,_methylmalonyl-CoA_+_malonyl-CoA_=>_tylactone_=>_tylosin	0	0	0	0	0	[illegible]	[illegible]	[illegible]	[illegible]	[illegible]	[illegible]	[illegible]	[illegible]	[illegible]	[illegible]	[illegible]	[illegible]	[illegible]	[illegible]	[illegible]	[illegible]	[illegible]	[illegible]	[illegible]	[illegible]	[illegible]	[illegible]	[illegible]	[illegible]	[illegible]	[illegible]	[illegible]	[illegible]	[illegible]	[illegible]	[illegible]	[illegible]	[illegible]	[illegible]	[illegible]	[illegible]	[illegible]	[illegible]	[illegible]	[illegible]	[illegible]	[illegible]	[illegible]	[illegible]	[illegible]	[illegible]	[illegible]	[illegible]	[illegible]	[illegible]	[illegible]	[illegible]	[illegible]	[illegible]	[illegible]	[illegible]	[illegible]	0	0	0		
M00774: Erythromycin_biosynthesis,_propanoyl-CoA_+_methylmalonyl-CoA_=>_deoxyerythronolide_B_=>_erythromycin_A/B	0	0	0	0	7.74E-07	[illegible]	[illegible]	[illegible]	[illegible]	[illegible]	[illegible]	[illegible]	[illegible]	[illegible]	[illegible]	[illegible]	[illegible]	[illegible]	[illegible]	[illegible]	[illegible]	[illegible]	[illegible]	[illegible]	[illegible]	[illegible]	[illegible]	[illegible]	[illegible]	[illegible]	[illegible]	[illegible]	[illegible]	[illegible]	[illegible]	[illegible]	[illegible]	[illegible]	[illegible]	[illegible]	[illegible]	[illegible]	[illegible]	[illegible]	[illegible]	[illegible]	[illegible]	[illegible]	[illegible]	[illegible]	[illegible]	[illegible]	[illegible]	[illegible]	[illegible]	[illegible]	[illegible]	[illegible]	[illegible]	[illegible]	[illegible]	[illegible]	0	0	0		
M00775: Oleandomycin_biosynthesis,_malonyl-CoA_+_methylmalonyl-CoA_=>_8,8a-deoxyoleandolide_=>_oleandomycin	0	0	0	0	5.16E-06	[illegible]	[illegible]	[illegible]	[illegible]	[illegible]	[illegible]	[illegible]	[illegible]	[illegible]	[illegible]	[illegible]	[illegible]	[illegible]	[illegible]	[illegible]	[illegible]	[illegible]	[illegible]	[illegible]	[illegible]	[illegible]	[illegible]	[illegible]	[illegible]	[illegible]	[illegible]	[illegible]	[illegible]	[illegible]	[illegible]	[illegible]	[illegible]	[illegible]	[illegible]	[illegible]	[illegible]	[illegible]	[illegible]	[illegible]	[illegible]	[illegible]	[illegible]	[illegible]	[illegible]	[illegible]	[illegible]	[illegible]	[illegible]	[illegible]	[illegible]	[illegible]	[illegible]	[illegible]	[illegible]	[illegible]	[illegible]	[illegible]	0	0	0		
M00776: Pikromycin/methymycin/neomethymycin_biosynthesis,_methylmalonyl-CoA_+_malonyl-CoA_=>_narbonolide/10-deoxymethynolide_=>_pikromycin/methymycin/neomethymycin	0	0	0	0	0	[illegible]	[illegible]	[illegible]	[illegible]	[illegible]	[illegible]	[illegible]	[illegible]	[illegible]	[illegible]	[illegible]	[illegible]	[illegible]	[illegible]	[illegible]	[illegible]	[illegible]	[illegible]	[illegible]	[illegible]	[illegible]	[illegible]	[illegible]	[illegible]	[illegible]	[illegible]	[illegible]	[illegible]	[illegible]	[illegible]	[illegible]	[illegible]	[illegible]	[illegible]	[illegible]	[illegible]	[illegible]	[illegible]	[illegible]	[illegible]	[illegible]	[illegible]	[illegible]	[illegible]	[illegible]	[illegible]	[illegible]	[illegible]	[illegible]	[illegible]	[illegible]	[illegible]	[illegible]	[illegible]	[illegible]	[illegible]	[illegible]	0	0	0		
M00777: Avermectin_biosynthesis,_2-methylbutanoyl-CoA/isobutyryl-CoA_=>_6,8a-Seco-6,8a-deoxy-5-oxoavermectin_1a/1b_aglycone_=>_avermectin_A1a/B1a/A1b/B1b	1.78E-06	0	0	2.15E-06	0	[illegible]	[illegible]	[illegible]	[illegible]	[illegible]	[illegible]	[illegible]	[illegible]	[illegible]	[illegible]	[illegible]	[illegible]	[illegible]	[illegible]	[illegible]	[illegible]	[illegible]	[illegible]	[illegible]	[illegible]	[illegible]	[illegible]	[illegible]	[illegible]	[illegible]	[illegible]	[illegible]	[illegible]	[illegible]	[illegible]	[illegible]	[illegible]	[illegible]	[illegible]	[illegible]	[illegible]	[illegible]	[illegible]	[illegible]	[illegible]	[illegible]	[illegible]	[illegible]	[illegible]	[illegible]	[illegible]	[illegible]	[illegible]	[illegible]	[illegible]	[illegible]	[illegible]	[illegible]	[illegible]	[illegible]	[illegible]	[illegible]	0	0	0		
M00778: Type_II_polyketide_backbone_biosynthesis,_acyl-CoA_+_malonyl-CoA_=>_polyketide	1.34E-06	0	6.08E-07	2.96E-06	4.13E-06	[illegible]	[illegible]	[illegible]	[illegible]	[illegible]	[illegible]	[illegible]	[illegible]	[illegible]	[illegible]	[illegible]	[illegible]	[illegible]	[illegible]	[illegible]	[illegible]	[illegible]	[illegible]	[illegible]	[illegible]	[illegible]	[illegible]	[illegible]	[illegible]	[illegible]	[illegible]	[illegible]	[illegible]	[illegible]	[illegible]	[illegible]	[illegible]	[illegible]	[illegible]	[illegible]	[illegible]	[illegible]	[illegible]	[illegible]	[illegible]	[illegible]	[illegible]	[illegible]	[illegible]	[illegible]	[illegible]	[illegible]	[illegible]	[illegible]	[illegible]	[illegible]	[illegible]	[illegible]	[illegible]	[illegible]	[illegible]	[illegible]	0	0	0		
M00779: Dihydrokalafungin_biosynthesis,_octaketide_=>_dihydrokalafungin	1.67E-06	0	0	8.07E-07	8.12E-06	[illegible]	[illegible]	[illegible]	[illegible]	[illegible]	[illegible]	[illegible]	[illegible]	[illegible]	[illegible]	[illegible]	[illegible]	[illegible]	[illegible]	[illegible]	[illegible]	[illegible]	[illegible]	[illegible]	[illegible]	[illegible]	[illegible]	[illegible]	[illegible]	[illegible]	[illegible]	[illegible]	[illegible]	[illegible]	[illegible]	[illegible]	[illegible]	[illegible]	[illegible]	[illegible]	[illegible]	[illegible]	[illegible]	[illegible]	[illegible]	[illegible]	[illegible]	[illegible]	[illegible]	[illegible]	[illegible]	[illegible]	[illegible]	[illegible]	[illegible]	[illegible]	[illegible]	[illegible]	[illegible]	[illegible]	[illegible]	[illegible]	0	0	0		
M00780: Tetracycline/oxytetracycline_biosynthesis,_pretetramide_=>_tetracycline/oxytetracycline	0	0	0	0	0	[illegible]	[illegible]	[illegible]	[illegible]	[illegible]	[illegible]	[illegible]	[illegible]	[illegible]	[illegible]	[illegible]	[illegible]	[illegible]	[illegible]	[illegible]	[illegible]	[illegible]	[illegible]	[illegible]	[illegible]	[illegible]	[illegible]	[illegible]	[illegible]	[illegible]	[illegible]	[illegible]	[illegible]	[illegible]	[illegible]	[illegible]	[illegible]	[illegible]	[illegible]	[illegible]	[illegible]	[illegible]	[illegible]	[illegible]	[illegible]	[illegible]	[illegible]	[illegible]	[illegible]	[illegible]	[illegible]	[illegible]	[illegible]	[illegible]	[illegible]	[illegible]	[illegible]	[illegible]	[illegible]	[illegible]	[illegible]	[illegible]	0	0	0		
M00781: Nogalavinone/aklavinone_biosynthesis,_deoxynogalonate/deoxyaklanonate_=>_nogalavinone/aklavinone	0	0	0	0	0	[illegible]	[illegible]	[illegible]	[illegible]	[illegible]	[illegible]	[illegible]	[illegible]	[illegible]	[illegible]	[illegible]	[illegible]	[illegible]	[illegible]	[illegible]	[illegible]	[illegible]	[illegible]	[illegible]	[illegible]	[illegible]	[illegible]	[illegible]	[illegible]	[illegible]	[illegible]	[illegible]	[illegible]	[illegible]	[illegible]	[illegible]	[illegible]	[illegible]	[illegible]	[illegible]	[illegible]	[illegible]	[illegible]	[illegible]	[illegible]	[illegible]	[illegible]	[illegible]	[illegible]	[illegible]	[illegible]	[illegible]	[illegible]	[illegible]	[illegible]	[illegible]	[illegible]	[illegible]	[illegible]	[illegible]	[illegible]	[illegible]	0	0	0		
M00782: Mithramycin_biosynthesis,_4-demethylpremithramycinone_=>_mithramycin	0	0	0	0	0	[illegible]	[illegible]	[illegible]	[illegible]	[illegible]	[illegible]	[illegible]	[illegible]	[illegible]	[illegible]	[illegible]	[illegible]	[illegible]	[illegible]	[illegible]	[illegible]	[illegible]	[illegible]	[illegible]	[illegible]	[illegible]	[illegible]	[illegible]	[illegible]	[illegible]	[illegible]	[illegible]	[illegible]	[illegible]	[illegible]	[illegible]	[illegible]	[illegible]	[illegible]	[illegible]	[illegible]	[illegible]	[illegible]	[illegible]	[illegible]	[illegible]	[illegible]	[illegible]	[illegible]	[illegible]	[illegible]	[illegible]	[illegible]	[illegible]	[illegible]	[illegible]	[illegible]	[illegible]	[illegible]	[illegible]	[illegible]	[illegible]	0	0	0		
M00783: Tetracenomycin_C/8-demethyltetracenomycin_C_biosynthesis,_tetracenomycin_F2_=>_tetracenomycin_C/8-demethyltetracenomycin_C	0	0	0	0	0	[illegible]	[illegible]	[illegible]	[illegible]	[illegible]	[illegible]	[illegible]	[illegible]	[illegible]	[illegible]	[illegible]	[illegible]	[illegible]	[illegible]	[illegible]	[illegible]	[illegible]	[illegible]	[illegible]	[illegible]	[illegible]	[illegible]	[illegible]	[illegible]	[illegible]	[illegible]	[illegible]	[illegible]	[illegible]	[illegible]	[illegible]	[illegible]	[illegible]	[illegible]	[illegible]	[illegible]	[illegible]	[illegible]	[illegible]	[illegible]	[illegible]	[illegible]	[illegible]	[illegible]	[illegible]	[illegible]	[illegible]	[illegible]	[illegible]	[illegible]	[illegible]	[illegible]	[illegible]	[illegible]	[illegible]	[illegible]	[illegible]	0	0	0		
M00784: Elloramycin_biosynthesis,_8-demethyltetracenomycin_C_=>_elloramycin_A	0	9.29E-07	0	1.08E-06	6.19E-06	[illegible]	[illegible]	[illegible]	[illegible]	[illegible]	[illegible]	[illegible]	[illegible]	[illegible]	[illegible]	[illegible]	[illegible]	[illegible]	[illegible]	[illegible]	[illegible]	[illegible]	[illegible]	[illegible]	[illegible]	[illegible]	[illegible]	[illegible]	[illegible]	[illegible]	[illegible]	[illegible]	[illegible]	[illegible]	[illegible]	[illegible]	[illegible]	[illegible]	[illegible]	[illegible]	[illegible]	[illegible]	[illegible]	[illegible]	[illegible]	[illegible]	[illegible]	[illegible]	[illegible]	[illegible]	[illegible]	[illegible]	[illegible]	[illegible]	[illegible]	[illegible]	[illegible]	[illegible]	[illegible]	[illegible]	[illegible]	[illegible]	0	0	0		
M00785: Cycloserine_biosynthesis,_arginine/serine_=>_cycloserine	0	0	0	0	0	[illegible]	[illegible]	[illegible]	[illegible]	[illegible]	[illegible]	[illegible]	[illegible]	[illegible]	[illegible]	[illegible]	[illegible]	[illegible]	[illegible]	[illegible]	[illegible]	[illegible]	[illegible]	[illegible]	[illegible]	[illegible]	[illegible]	[illegible]	[illegible]	[illegible]	[illegible]	[illegible]	[illegible]	[illegible]	[illegible]	[illegible]	[illegible]	[illegible]	[illegible]	[illegible]	[illegible]	[illegible]	[illegible]	[illegible]	[illegible]	[illegible]	[illegible]	[illegible]	[illegible]	[illegible]	[illegible]	[illegible]	[illegible]	[illegible]	[illegible]	[illegible]	[illegible]	[illegible]	[illegible]	[illegible]	[illegible]	[illegible]	0	0	0		

Table S4. VI

Supplementary data. Table S4. Coverage of KEGG functional modules in oil contaminated environments.
Percentage of coverage of KEGG functional modules as estimated by HUMAnN2 is described. Presence/absence of KEGG functional was defined according to the following criteria: median of percentage coverage estimates for samples of each oil polluted habitat > 0.9 represents presence; < 0.1 denotes absence. Core Modules (denoted by presence in all habitats) are colored in green while Varied Modules (denoted by presence/absence in different habitats) are colored in red.

KEGG_Module	India_oil_refineries				Arctic												China_oil_refineries									Mangrove		
	I1	I2	I3	I4	A1	A10	A11	A12	A2	A3	A4	A5	A6	A7	A8	A9	C1	C2	C3	C4	C5	C6	C7	C8	C9	M1	M2	M3
M00001: Glycolysis_(Embden-Meyerhof_pathway),_glucose_=>_pyruvate	0.466667	0.433333	0.466667	0.4	0.433333	0.366667	0.4	0.433333	0.433333	0.433333	0.4	0.433333	0.466667	0.433333	0.433333	0.433333	0.533333	0.566667	0.6	0.566667	0.566667	0.5	0.566667	0.566667	0.5	0.5	0.533333	0.466667
M00002: Glycolysis,_core_module_involving_three-carbon_compounds	0.666667	0.666667	0.666667	0.666667	0.666667	0.583333	0.583333	0.666667	0.666667	0.75	0.666667	0.666667	0.666667	0.583333	0.666667	0.666667	0.75	0.75	0.75	0.75	0.75	0.75	0.75	0.75	0.75	0.75	0.75	0.75
M00003: Gluconeogenesis,_oxaloacetate_=>_fructose-6P	0.55	0.5	0.6	0.5	0.55	0.45	0.5	0.6	0.5	0.6	0.55	0.6	0.55	0.5	0.6	0.5	0.7	0.75	0.75	0.75	0.75	0.65	0.75	0.8	0.65	0.7	0.75	0.6
M00004: Pentose_phosphate_pathway_(Pentose_phosphate_cycle)	0.6	0.6	0.666667	0.6	0.6	0.6	0.6	0.6	0.533333	0.533333	0.6	0.533333	0.533333	0.533333	0.6	0.6	0.666667	0.733333	0.666667	0.733333	0.733333	0.666667	0.733333	0.733333	0.666667	0.666667	0.666667	0.666667
M00005: PRPP_biosynthesis,_ribose_5P_=>_PRPP	1	1	1	1	1	1	1	1	1	1	1	1	1	1	1	1	1	1	1	1	1	1	1	1	1	1	1	1
M00006: Pentose_phosphate_pathway,_oxidative_phase,_glucose_6P_=>_ribulose_5P	0.5	0.5	0.5	0.5	0.5	0.5	0.5	0.5	0.5	0.5	0.5	0.5	0.5	0.5	0.5	0.5	0.5	0.666667	0.5	0.666667	0.666667	0.5	0.666667	0.666667	0.5	0.5	0.5	0.5
M00007: Pentose_phosphate_pathway,_non-oxidative_phase,_fructose_6P_=>_ribose_5P	0.833333	0.833333	1	0.833333	0.833333	0.833333	0.833333	0.833333	0.666667	0.666667	0.833333	0.666667	0.666667	0.666667	0.833333	0.833333	1	1	1	1	1	1	1	1	1	1	1	1
M00008: Entner-Doudoroff_pathway,_glucose-6P_=>_glyceraldehyde-3P_+_pyruvate	0.8	0.8	0.6	0.6	0.8	0.6	0.8	0.8	0.6	0.6	0.6	0.6	0.6	0.6	0.6	0.6	0.6	0.8	0.6	0.8	0.8	0.8	0.8	0.8	0.8	0.8	0.8	0.8
M00009: Citrate_cycle_(TCA_cycle,_Krebs_cycle)	0.487179	0.512821	0.512821	0.487179	0.512821	0.512821	0.461538	0.487179	0.487179	0.461538	0.487179	0.512821	0.487179	0.487179	0.512821	0.487179	0.487179	0.538462	0.512821	0.564103	0.512821	0.538462	0.589744	0.512821	0.564103	0.615385	0.564103	0.615385
M00010: Citrate_cycle,_first_carbon_oxidation,_oxaloacetate_=>_2-oxoglutarate	1	1	0.8	1	0.6	0.8	0.6	0.6	0.8	0.6	0.6	0.8	0.6	0.6	0.8	0.8	0.8	0.8	0.8	0.8	0.8	1	0.8	0.8	1	1	1	1
M00011: Citrate_cycle,_second_carbon_oxidation,_2-oxoglutarate_=>_oxaloacetate	0.411765	0.441176	0.470588	0.411765	0.5	0.470588	0.441176	0.470588	0.441176	0.441176	0.470588	0.470588	0.470588	0.470588	0.470588	0.441176	0.441176	0.5	0.470588	0.529412	0.470588	0.470588	0.558824	0.470588	0.5	0.558824	0.5	0.558824
M00012: Glyoxylate_cycle	0.666667	0.666667	0.666667	0.666667	0.555556	0.666667	0.555556	0.555556	0.555556	0.555556	0.555556	0.555556	0.555556	0.555556	0.555556	0.555556	0.555556	0.555556	0.555556	0.555556	0.555556	0.666667	0.555556	0.555556	0.666667	0.666667	0.666667	0.666667
M00013: Malonate_semialdehyde_pathway,_propanoyl-CoA_=>_acetyl-CoA	0.333333	0.333333	0.333333	0.333333	0.333333	0.333333	0.333333	0.333333	0.333333	0.333333	0.333333	0.333333	0.333333	0.333333	0.333333	0.333333	0.333333	0.333333	0.333333	0.333333	0.333333	0.333333	0.333333	0.333333	0.333333	0.333333	0.333333	0.333333
M00014: Glucuronate_pathway_(uronate_pathway)	0.25	0.25	0.083333	0.083333	0.166667	0.166667	0.166667	0.166667	0.25	0.166667	0.166667	0.25	0.166667	0.166667	0.25	0.25	0.25	0.25	0.25	0.25	0.25	0.25	0.25	0.25	0.25	0.25	0.25	0.25
M00015: Proline_biosynthesis,_glutamate_=>_proline	0.75	0.75	0.75	0.75	0.75	0.75	0.75	0.75	0.75	0.75	0.75	0.75	0.75	0.75	0.75	0.75	0.75	0.75	0.75	0.75	0.75	0.75	0.75	0.75	0.75	0.75	0.75	0.75
M00016: Lysine_biosynthesis,_succinyl-DAP_pathway,_aspartate_=>_lysine	0.714286	0.714286	0.714286	0.714286	0.714286	0.714286	0.714286	0.714286	0.714286	0.714286	0.714286	0.714286	0.714286	0.714286	0.714286	0.714286	0.714286	0.714286	0.714286	0.714286	0.714286	0.714286	0.714286	0.642857	0.714286	0.785714	0.714286	0.785714
M00017: Methionine_biosynthesis,_apartate_=>_homoserine_=>_methionine	0.538462	0.461538	0.538462	0.538462	0.538462	0.538462	0.538462	0.538462	0.538462	0.538462	0.538462	0.538462	0.461538	0.461538	0.538462	0.538462	0.538462	0.538462	0.538462	0.538462	0.538462	0.538462	0.538462	0.538462	0.538462	0.615385	0.538462	0.615385
M00018: Threonine_biosynthesis,_aspartate_=>_homoserine_=>_threonine	0.5	0.5	0.416667	0.416667	0.5	0.416667	0.5	0.5	0.416667	0.416667	0.5	0.5	0.416667	0.416667	0.5	0.5	0.5	0.5	0.5	0.5	0.5	0.5	0.5	0.5	0.5	0.583333	0.5	0.583333
M00019: Valine/isoleucine_biosynthesis,_pyruvate_=>_valine_/_2-oxobutanoate_=>_isoleucine	0.833333	0.833333	0.833333	0.833333	0.833333	0.833333	0.833333	0.833333	0.833333	0.833333	0.833333	0.833333	0.833333	0.833333	0.833333	0.833333	0.833333	0.833333	0.833333	0.833333	0.833333	0.833333	0.833333	0.833333	0.833333	0.833333	0.833333	0.833333
M00020: Serine_biosynthesis,_glycerate-3P_=>_serine	1	1	1	1	1	1	1	1	1	1	1	1	1	1	1	1	1	1	1	1	1	1	1	1	1	1	1	1
M00021: Cysteine_biosynthesis,_serine_=>_cysteine	0.6	0.6	0.6	0.6	0.6	0.6	0.6	0.6	0.6	0.6	0.6	0.6	0.4	0.4	0.6	0.6	0.6	0.6	0.6	0.6	0.6	0.6	0.6	0.6	0.6	0.6	0.6	0.6
M00022: Shikimate_pathway,_phosphoenolpyruvate_+_erythrose-4P_=>_chorismate	0.6	0.6	0.533333	0.533333	0.533333	0.533333	0.466667	0.533333	0.6	0.6	0.6	0.533333	0.533333	0.533333	0.533333	0.533333	0.6	0.6	0.6	0.666667	0.666667	0.6	0.6	0.733333	0.6	0.6	0.6	0.6
M00023: Tryptophan_biosynthesis,_chorismate_=>_tryptophan	0.4375	0.4375	0.5	0.5	0.5	0.5	0.4375	0.4375	0.4375	0.5	0.5	0.4375	0.5	0.5	0.5	0.4375	0.5	0.5	0.5	0.5	0.5	0.5	0.5	0.5	0.5	0.5	0.5	0.5
M00024: Phenylalanine_biosynthesis,_chorismate_=>_phenylalanine	0.285714	0.285714	0.214286	0.214286	0.214286	0.214286	0.285714	0.285714	0.214286	0.142857	0.142857	0.142857	0.142857	0.214286	0.142857	0.142857	0.285714	0.285714	0.285714	0.214286	0.285714	0.357143	0.285714	0.214286	0.357143	0.142857	0.142857	0.142857
M00025: Tyrosine_biosynthesis,_chorismate_=>_tyrosine	0.285714	0.285714	0.214286	0.214286	0.214286	0.214286	0.285714	0.285714	0.214286	0.142857	0.142857	0.142857	0.142857	0.214286	0.142857	0.142857	0.285714	0.285714	0.285714	0.214286	0.285714	0.357143	0.285714	0.214286	0.357143	0.142857	0.142857	0.142857
M00026: Histidine_biosynthesis,_PRPP_=>_histidine	0.578947	0.578947	0.631579	0.578947	0.526316	0.526316	0.578947	0.578947	0.526316	0.526316	0.473684	0.526316	0.526316	0.526316	0.526316	0.526316	0.684211	0.631579	0.684211	0.631579	0.631579	0.684211	0.631579	0.631579	0.631579	0.631579	0.631579	0.631579
M00027: GABA_(gamma-Aminobutyrate)_shunt	0.285714	0.428571	0.285714	0.285714	0.285714	0.142857	0.285714	0.142857	0.285714	0.142857	0.142857	0.142857	0.285714	0.285714	0.285714	0.142857	0.571429	0.571429	0.571429	0.571429	0.571429	0.428571	0.571429	0.571429	0.428571	0.285714	0.285714	0.142857
M00028: Ornithine_biosynthesis,_glutamate_=>_ornithine	0.5	0.571429	0.5	0.5	0.571429	0.571429	0.571429	0.571429	0.571429	0.571429	0.571429	0.571429	0.5	0.5	0.571429	0.5	0.5	0.571429	0.5	0.571429	0.571429	0.571429	0.571429	0.571429	0.571429	0.571429	0.571429	0.571429
M00029: Urea_cycle	0.666667	0.666667	0.666667	0.666667	0.666667	0.666667	0.666667	0.666667	0.666667	0.666667	0.666667	0.666667	0.666667	0.666667	0.666667	0.666667	0.666667	0.666667	0.666667	0.666667	0.666667	0.666667	0.666667	0.666667	0.666667	0.666667	0.666667	0.666667
M00030: Lysine_biosynthesis,_AAA_pathway,_2-oxoglutarate_=>_2-aminoadipate_=>_lysine	0	0	0	0	0	0	0	0	0	0	0	0	0	0	0	0	0.2	0	0	0	0	0	0	0.1	0	0	0	0
M00031: Lysine_biosynthesis,_mediated_by_LysW,_2-aminoadipate_=>_lysine	0.166667	0.166667	0	0	0	0	0	0	0	0	0	0	0	0	0	0	1	1	1	1	0.5	0.5	0.5	0.333333	0.5	0	0	0
M00032: Lysine_degradation,_lysine_=>_saccharopine_=>_acetoacetyl-CoA	0.277778	0.222222	0.277778	0.277778	0.277778	0.222222	0.277778	0.277778	0.222222	0.277778	0.277778	0.222222	0.277778	0.277778	0.277778	0.222222	0.277778	0.277778	0.277778	0.277778	0.277778	0.333333	0.277778	0.333333	0.277778	0.277778	0.277778	0.277778
M00033: Ectoine_biosynthesis,_aspartate_=>_ectoine	0.4	0.4	0.4	0.4	0.4	0.4	0.4	0.4	0.4	0.4	0.4	0.4	0.4	0.4	0.4	0.4	0.6	0.6	0.8	0.6	0.6	0.6	0.4	0.4	0.6	0.6	0.6	0.6
M00034: Methionine_salvage_pathway	0.368421	0.368421	0.368421	0.368421	0.263158	0.315789	0.263158	0.315789	0.315789	0.263158	0.263158	0.263158	0.210526	0.263158	0.315789	0.263158	0.315789	0.315789	0.315789	0.315789	0.315789	0.368421	0.315789	0.315789	0.368421	0.315789	0.263158	0.315789
M00035: Methionine_degradation	0.428571	0.428571	0.571429	0.428571	0.571429	0.428571	0.571429	0.571429	0.428571	0.571429	0.428571	0.428571	0.571429	0.571429	0.428571	0.428571	0.571429	0.571429	0.571429	0.571429	0.571429	0.571429	0.571429	0.571429	0.571429	0.571429	0.571429	0.571429
M00036: Leucine_degradation,_leucine_=>_acetoacetate_+_acetyl-CoA	0.692308	0.692308	0.692308	0.692308	0.615385	0.615385	0.615385	0.692308	0.692308	0.692308	0.692308	0.692308	0.538462	0.615385	0.692308	0.692308	0.692308	0.769231	0.769231	0.692308	0.769231	0.846154	0.769231	0.692308	0.769231	0.692308	0.692308	0.692308
M00038: Tryptophan_metabolism,_tryptophan_=>_kynurenine_=>_2-aminomuconate	0.2	0.2	0.2	0.2	0.2	0.2	0.2	0.3	0.1	0.2	0.2	0.1	0.1	0.1	0.2	0.2	0.5	0.5	0.5	0.4	0.7	0.4	0.4	0.5	0.5	0	0.1	0
M00039: Monolignol_biosynthesis,_phenylalanine_/_tyrosine__=>_monolignol	0	0	0	0	0	0	0	0	0	0	0	0	0	0	0	0	0	0	0	0	0	0	0	0	0	0	0	0
M00040: Tyrosine_biosynthesis,_prephanate_=>_pretyrosine_=>_tyrosine	0.2	0.2	0.2	0.2	0	0.2	0.2	0.2	0	0	0	0	0	0	0	0	0	0	0	0.2	0.2	0.2	0	0.2	0.2	0	0	0
M00042: Catecholamine_biosynthesis,_tyrosine_=>_dopamine_=>_noradrenaline_=>_adrenaline	0.166667	0.166667	0	0	0	0	0	0	0	0	0	0	0	0	0	0	0.166667	0	0	0	0	0	0	0	0	0.166667	0.166667	0.166667
M00044: Tyrosine_degradation,_tyrosine_=>_homogentisate	0.833333	0.833333	0.5	0.666667	0.166667	0.666667	0.5	0.333333	0.5	0.333333	0.333333	0.333333	0.333333	0.333333	0.5	0.5	0.5	0.5	0.5	0.5	0.5	0.666667	0.5	0.5	0.666667	0.333333	0.333333	0.333333
M00045: Histidine_degradation,_histidine_=>_N-formiminoglutamate_=>_glutamate	0.5	0.5	0.75	0.5	1	0.5	1	1	0.75	0.75	0.75	0.75	1	1	0.75	0.5	1	1	0.75	0.75	0.75	1	0.75	0.75	1	0.75	0.75	0.75
M00046: Pyrimidine_degradation,_uracil_=>_beta-alanine,_thymine_=>_3-aminoisobutanoate	0	0	0	0	0	0	0	0	0	0	0	0	0	0	0	0	0	0	0	0	0	0	0	0	0	0	0	0
M00047: Creatine_pathway	0	0	0	0	0	0	0	0	0	0	0	0	0	0	0	0	0	0	0	0	0	0	0	0	0	0	0	0
M00048: Inosine_monophosphate_biosynthesis,_PRPP_+_glutamine_=>_IMP	0.44	0.4	0.4	0.44	0.4	0.4	0.4	0.4	0.4	0.4	0.4	0.4	0.4	0.4	0.4	0.4	0.44	0.44	0.44	0.4	0.4	0.44	0.4	0.44	0.44	0.48	0.48	0.48
M00049: Adenine_ribonucleotide_biosynthesis,_IMP_=>_ADP,ATP	0.555556	0.555556	0.555556	0.555556	0.555556	0.555556	0.555556	0.555556	0.555556	0.555556	0.555556	0.555556	0.555556	0.555556	0.555556	0.555556	0.555556	0.555556	0.555556	0.555556	0.555556	0.555556	0.555556	0.555556	0.555556	0.555556	0.555556	0.555556
M00050: Guanine_ribonucleotide_biosynthesis_IMP_=>_GDP,GTP	0.833333	0.833333	0.833333	0.833333	0.833333	0.833333	0.833333	0.833333	0.833333	0.833333	0.833333	0.833333	0.833333	0.833333	0.833333	0.833333	0.833333	0.833333	0.833333	0.833333	0.833333	0.833333	0.833333	0.833333	0.833333	0.833333	0.833333	0.833333
M00051: Uridine_monophosphate_biosynthesis,_glutamine_(+_PRPP)_=>_UMP	0.5	0.5	0.5	0.4375	0.5	0.4375	0.4375	0.4375	0.5	0.5	0.5	0.5	0.5	0.4375	0.5	0.5	0.5625	0.5	0.5	0.5	0.5	0.5	0.5	0.5625	0.5625	0.5625	0.5	0.5
M00052: Pyrimidine_ribonucleotide_biosynthesis,_UMP_=>_UDP/UTP,CDP/CTP	0.428571	0.428571	0.428571	0.428571	0.428571	0.428571	0.428571	0.428571	0.428571	0.428571	0.428571	0.428571	0.428571	0.428571	0.428571	0.428571	0.428571	0.428571	0.428571	0.428571	0.428571	0.428571	0.428571	0.428571	0.428571	0.428571	0.428571	0.428571
M00053: Pyrimidine_deoxyribonuleotide_biosynthesis,_CDP/CTP_=>_dCDP/dCTP,dTDP/dTTP	0.5	0.571429	0.571429	0.571429	0.571429	0.571429	0.5	0.5	0.5	0.571429	0.571429	0.5	0.5	0.5	0.571429	0.5	0.5	0.571429	0.571429	0.571429	0.571429	0.5	0.571429	0.571429	0.5	0.571429	0.571429	0.571429
M00055: N-glycan_precursor_biosynthesis	0	0	0	0	0	0	0	0	0	0	0	0	0	0	0	0	0	0	0	0	0	0	0	0	0	0	0	0
M00060: Lipopolysaccharide_biosynthesis,_KDO2-lipid_A	0.8	0.8	0.9	0.8	0.7	0.8	0.8	0.9	0.7	0.8	0.8	0.7	0.7	0.7	0.7	0.7	0.7	0.9	0.7	0.9	0.9	0.8	0.9	0.9	0.9	0.9	0.9	0.9
M00061: D-Glucuronate_degradation	0.571429	0.571429	0.142857	0.142857	0.571429	0.142857	0.571429	0.571429	0.571429	0.285714	0.285714	0.571429	0.571429	0.571429	0.285714	0.571429	0.428571	0.428571	0.571429	0.428571	0.428571	0.428571	0.428571	0.428571	0.428571	0.285714	0.285714	0.285714
M00063: CMP-KDO_biosynthesis	1	1	1	1	1	1	1	1	1	1	1	1	1	1	1	1	1	1	1	1	1	1	1	1	1	1	1	1
M00064: ADP-L-glycero-D-manno-heptose_biosynthesis	1	1	1	1	0.75	0.75	0.5	0.5	1	0.75	0.75	0.75	0.75	0.5	1	1	0.75	1	0.75	1	1	0.75	1	1	1	1	1	1
M00066: Lactosylceramide_biosynthesis	0.5	0.5	0	0	0	0	0	0	0.5	0	0	0.5	0	0	0.5	0.5	0.5	0.5	0.5	0.5	0.5	0.5	0.5	0.5	0.5	0	0	0
M00072: N-glycosylation_by_oligosaccharyltransferase	0	0	0	0	0	0	0	0	0	0	0	0	0	0	0	0	0	0	0	0.125	0	0	0	0	0	0	0	0
M00073: N-glycan_precursor_trimming	0	0	0	0	0	0	0	0	0	0	0	0	0	0	0	0	0	0	0	0	0	0	0	0	0	0	0	0
M00075: N-glycan_biosynthesis,_complex_type	0	0	0	0	0	0	0	0	0	0	0	0	0	0	0	0	0	0	0	0	0	0	0	0	0	0	0	0
M00076: Dermatan_sulfate_degradation	0	0	0	0	0	0	0	0	0	0	0	0	0	0	0	0	0	0	0	0	0	0	0	1	0	0	0	0
M00077: Chondroitin_sulfate_degradation	0	0	0	0	0	0	0	0	0	0	0	0	0	0	0	0	0	0	0	0	0	0	0	0.25	0	0	0	0
M00078: Heparan_sulfate_degradation	0.111111	0	0	0	0	0	0	0	0	0	0	0	0	0	0	0	0	0.111111	0	0.222222	0.222222	0	0.111111	0.222222	0	0	0	0
M00080: Lipopolysaccharide_biosynthesis,_inner_core_=>_outer_core_=>_O-antigen	0.25	0.333333	0.25	0.166667	0.166667	0.25	0.083333	0.166667	0.25	0.25	0.25	0.25	0.25	0.083333	0.25	0.25	0.333333	0.416667	0.333333	0.416667	0.416667	0.333333	0.416667	0.416667	0.333333	0.416667	0.416667	0.416667
M00081: Pectin_degradation	0.666667	0.666667	0	0	0	0	0.333333	0	0.666667	0	0	0.333333	0	0	0.333333	0.333333	0.333333	0.333333	0.333333	0.333333	0.333333	0.333333	0.333333	0.333333	0.333333	0	0	0
M00082: Fatty_acid_biosynthesis,_initiation	0.428571	0.428571	0.428571	0.428571	0.5	0.5	0.5	0.5	0.5	0.5	0.5	0.5	0.5	0.5	0.428571	0.428571	0.5	0.5	0.571429	0.5	0.5	0.5	0.5	0.5	0.5	0.428571	0.428571	0.428571
M00083: Fatty_acid_biosynthesis,_elongation	0.466667	0.4	0.533333	0.466667	0.466667	0.4	0.4	0.466667	0.4	0.533333	0.533333	0.4	0.333333	0.333333	0.4	0.4	0.4	0.466667	0.4	0.533333	0.466667	0.4	0.466667	0.533333	0.466667	0.533333	0.6	0.533333
M00085: Fatty_acid_biosynthesis,_elongation,_mitochondria	0	0	0	0	0	0	0	0	0	0	0	0	0	0	0	0	0	0	0	0	0	0	0	0.166667	0	0	0	0
M00086: beta-Oxidation,_acyl-CoA_synthesis	0.5	0.5	0.5	0.5	0.5	0.5	0.5	0.5	0.5	0.5	0.5	0.5	0.5	0.5	0.5	0.5	0.5	0.5	0.5	0.5	0.5	0.5	0.5	0.5	0.5	0.5	0.5	0.5
M00087: beta-Oxidation	0.263158	0.210526	0.263158	0.263158	0.368421	0.263158	0.315789	0.315789	0.263158	0.315789	0.315789	0.210526	0.368421	0.368421	0.315789	0.210526	0.315789	0.263158	0.315789	0.263158	0.263158	0.473684	0.263158	0.263158	0.368421	0.315789	0.315789	0.368421
M00088: Ketone_body_biosynthesis,_acetyl-CoA_=>_acetoacetate/3-hydroxybutyrate/acetone	0.6	0.6	0.6	0.6	0.6	0.6	0.6	0.6	0.6	0.6	0.6	0.6	0.8	0.8	0.6	0.6	0.8	0.6	0.6	0.6	0.6	0.8	0.6	0.6	0.6	0.6	0.6	0.6
M00089: Triacylglycerol_biosynthesis	0.142857	0.142857	0.142857	0.142857	0.142857	0.142857	0.142857	0.142857	0.142857	0.142857	0.142857	0.142857	0.142857	0.142857	0.142857	0.142857	0.238095	0.190476	0.285714	0.142857	0.142857	0.238095	0.142857	0.142857	0.238095	0.142857	0.142857	0.142857
M00090: Phosphatidylcholine_(PC)_biosynthesis,_choline_=>_PC	0	0	0	0	0	0	0	0	0	0	0	0	0	0	0	0	0	0	0	0	0	0	0	0	0	0	0	0
M00091: Phosphatidylcholine_(PC)_biosynthesis,_PE_=>_PC	0	0	0	0.25	0	0	0	0	0	0	0	0	0	0	0	0	0	0	0	0	0	0	0	0	0	0	0	0
M00092: Phosphatidylethanolamine_(PE)_biosynthesis,_ethanolamine_=>_PE	0	0	0	0	0	0	0	0	0	0	0	0	0	0	0	0	0	0	0	0	0	0	0	0	0	0	0	0
M00093: Phosphatidylethanolamine_(PE)_biosynthesis,_PA_=>_PS_=>_PE	0.75	0.75	0.75	0.75	0.75	0.75	0.75	0.75	0.75	0.75	0.75	0.75	0.75	0.75	0.75	0.75	0.75	0.75	0.75	0.75	0.75	0.75	0.75	0.75	0.75	0.75	0.75	0.75
M00095: C5_isoprenoid_biosynthesis,_mevalonate_pathway	0.125	0.125	0.25	0.125	0.25	0.125	0.25	0.25	0.125	0.125	0.125	0.125	0.25	0.25	0.125	0.125	0.625	0.25	0.25	0.25	0.25	0.25	0.25	0.25	0.25	0.25	0.25	0.25
M00096: C5_isoprenoid_biosynthesis,_non-mevalonate_pathway	0.8	0.7	0.8	0.7	0.9	0.8	0.9	0.9	0.8	0.8	0.8	0.8	0.9	0.9	0.8	0.8	0.9	0.8	0.9	0.8	0.9	0.8	0.9	0.8	0.8	0.9	0.9	0.9
M00097: beta-Carotene_biosynthesis,_GGAP_=>_beta-carotene	0.333333	0.333333	0.166667	0.166667	0.166667	0.166667	0.166667	0.166667	0.333333	0.166667	0.166667	0.166667	0.166667	0.166667	0.166667	0.166667	0.5	0.5	0.5	0.166667	0.5	0.166667	0.5	0.5	0.666667	0.333333	0.333333	0.333333
M00098: Acylglycerol_degradation	0	0	0	0	0	0	0	0	0	0	0	0	0	0	0	0	0.076923	0.076923	0.076923	0.076923	0.076923	0.076923	0.076923	0.076923	0.076923	0	0	0
M00099: Sphingosine_biosynthesis	0	0	0	0	0	0	0	0	0	0	0	0	0	0	0	0	0	0	0	0	0	0	0	0	0	0	0	0
M00101: Cholesterol_biosynthesis,_squalene_2,3-epoxide_=>_cholesterol	0	0	0	0	0	0	0	0	0	0	0	0	0	0	0	0	0	0	0	0	0	0	0	0	0	0	0	0
M00102: Ergocalciferol_biosynthesis	0	0	0	0	0	0	0	0	0	0	0	0	0	0	0	0	0	0	0	0	0	0	0	0	0	0	0	0
M00104: Bile_acid_biosynthesis,_cholesterol_=>_cholate	0	0	0	0.083333	0	0.083333	0.083333	0	0	0	0	0	0	0	0	0	0.083333	0.083333	0.083333	0	0.083333	0.083333	0.083333	0	0.083333	0	0	0
M00107: Steroid_hormone_biosynthesis,_cholesterol_=>_prognenolone_=>_progesterone	0	0	0	0	0	0	0	0	0	0	0	0	0	0	0	0	0	0	0.333333	0	0	0	0	0	0	0	0	0
M00112: Tocopherol_biosynthesis	0	0	0	0	0	0	0	0	0	0	0	0	0	0	0	0	0.2	0	0	0	0.2	0	0	0.2	0	0	0	0
M00114: Ascorbate_biosynthesis,_plants,_glucose-6P_=>_ascorbate	0.3	0.25	0.35	0.25	0.25	0.25	0.25	0.25	0.2	0.3	0.3	0.2	0.2	0.25	0.25	0.2	0.45	0.45	0.5	0.45	0.45	0.4	0.45	0.45	0.45	0.4	0.4	0.45
M00115: NAD_biosynthesis,_aspartate_=>_NAD	0.857143	0.857143	0.857143	0.857143	0.714286	0.714286	0.714286	0.714286	0.857143	0.714286	0.714286	0.857143	0.857143	0.714286	0.857143	0.857143	0.857143	0.857143	0.857143	0.857143	0.857143	0.857143	0.857143	0.857143	0.857143	0.857143	0.857143	0.857143
M00116: Menaquinone_biosynthesis,_chorismate_=>_menaquinone	0.166667	0.166667	0.5	0.166667	0.583333	0.166667	0.583333	0.583333	0.166667	0.5	0.166667	0.166667	0.583333	0.583333	0.166667	0.166667	0.75	0.666667	0.75	0.666667	0.666667	0.75	0.666667	0.75	0.75	0.666667	0.666667	0.666667
M00117: Ubiquinone_biosynthesis,_prokaryotes,_chorismate_=>_ubiquinone	0.545455	0.545455	0.545455	0.545455	0.545455	0.545455	0.545455	0.545455	0.545455	0.454545	0.545455	0.545455	0.363636	0.272727	0.545455	0.454545	0.545455	0.545455	0.454545	0.545455	0.545455	0.636364	0.545455	0.545455	0.636364	0.545455	0.545455	0.545455
M00118: Glutathione_biosynthesis,_glutamate_=>_glutathione	0.5	0.5	0.5	0.5	0.5	0.5	0.5	0.5	0.25	0.25	0.5	0.5	0	0.25	0.5	0.5	0.5	0.5	0.5	0.5	0.5	0.5	0.5	0.5	0.5	0.5	0.5	0.5
M00119: Pantothenate_biosynthesis,_valine/L-aspartate_=>_pantothenate	0.833333	0.833333	0.833333	0.833333	0.833333	0.833333	0.833333	0.833333	0.833333	0.833333	0.833333	0.833333	0.833333	0.833333	0.833333	0.833333	0.833333	0.833333	0.833333	0.833333	0.833333	0.833333	0.833333	0.833333	0.833333	0.833333	0.833333	0.833333
M00120: Coenzyme_A_biosynthesis,_pantothenate_=>_CoA	0.363636	0.363636	0.363636	0.363636	0.454545	0.363636	0.454545	0.454545	0.363636	0.363636	0.454545	0.363636	0.454545	0.454545	0.363636	0.363636	0.454545	0.454545	0.454545	0.363636	0.363636	0.454545	0.454545	0.454545	0.363636	0.363636	0.363636	0.363636
M00121: Heme_biosynthesis,_glutamate_=>_protoheme/siroheme	0.695652	0.652174	0.695652	0.608696	0.652174	0.565217	0.565217	0.652174	0.608696	0.608696	0.608696	0.652174	0.565217	0.565217	0.608696	0.608696	0.652174	0.652174	0.652174	0.652174	0.652174	0.652174	0.652174	0.652174	0.652174	0.652174	0.695652	0.652174
M00122: Cobalamin_biosynthesis,_cobinamide_=>_cobalamin	0.777778	0.888889	0.888889	0.888889	0.888889	0.888889	0.888889	0.888889	0.777778	0.888889	0.888889	0.777778	0.777778	0.888889	0.888889	0.777778	0.888889	0.888889	0.888889	0.888889	0.888889	0.888889	0.888889	0.888889	0.888889	0.888889	0.888889	0.888889
M00123: Biotin_biosynthesis,_pimeloyl-ACP/CoA_=>_biotin	0.666667	0.666667	0.666667	0.666667	0.666667	0.666667	0.666667	0.666667	0.666667	0.666667	0.666667	0.666667	0.666667	0.666667	0.666667	0.666667	0.666667	0.666667	0.666667	0.666667	0.666667	0.666667	0.666667	0.666667	0.666667	0.666667	0.666667	0.666667

KEGG_Module	Marine_sediments							Taiga_upper_active_layer				Taiga_bottom_active_layer				Taiga_permafrost_layer				Oil_sands_core							Oil_sands_tailings_pond_upper				Oil_sands_tailings_pond_median			Oil_sands_tailings_pond_deep			
	DWH1	DWH2	DWH3	DWH4	DWH5	DWH6	DWH7	Tu1	Tu2	Tu3	Tu4	Tb1	Tb2	Tb3	Tb4	Tp1	Tp2	Tp3	Tp4	OSC1	OSC12	OSC3	OSC4	OSC5	OSC7	OSC9	OSTPu1	OSTPu2	OSTPu4	OSTPu3	OSTPm2	OSTPm6	OSTPm4	OSTPd1	OSTPd2	OSTPd3	OSTPd4
M00001	0.466667	0.433333	0.433333	0.433333	0.4	0.4	0.433333	0.4	0.366667	0.433333	0.4	0.4	0.433333	0.433333	0.366667	0.433333	0.4	0.5	0.366667	0.433333	0.433333	0.4	0.4	0.433333	0.433333	0.433333	0.4	0.4	0.433333	0.433333	0.433333	0.466667	0.4	0.4	0.4	0.4	0.433333
M00002	0.75	0.666667	0.75	0.666667	0.666667	0.583333	0.666667	0.583333	0.583333	0.666667	0.583333	0.583333	0.666667	0.666667	0.583333	0.666667	0.583333	0.666667	0.583333	0.666667	0.666667	0.666667	0.666667	0.666667	0.666667	0.666667	0.666667	0.666667	0.75	0.75	0.75	0.75	0.666667	0.666667	0.666667	0.666667	0.75
M00003	0.55	0.55	0.55	0.55	0.5	0.5	0.55	0.55	0.55	0.5	0.55	0.55	0.65	0.6	0.55	0.65	0.6	0.5	0.55	0.6	0.6	0.5	0.6	0.6	0.6	0.6	0.5	0.5	0.6	0.6	0.55	0.65	0.5	0.5	0.5	0.5	0.55
M00004	0.6	0.6	0.6	0.6	0.6	0.6	0.6	0.6	0.533333	0.666667	0.6	0.6	0.6	0.666667	0.6	0.6	0.6	0.733333	0.533333	0.533333	0.533333	0.533333	0.533333	0.533333	0.533333	0.6	0.533333	0.466667	0.533333	0.533333	0.6	0.666667	0.533333	0.466667	0.466667	0.6	0.6
M00005	1	1	1	1	1	1	1	1	1	1	1	1	1	1	1	1	1	1	1	1	1	1	1	1	1	1	1	1	1	1	1	1	1	1	1	1	1
M00006	0.5	0.5	0.5	0.5	0.5	0.5	0.5	0.5	0.333333	0.666667	0.5	0.5	0.5	0.5	0.5	0.5	0.5	0.666667	0.333333	0.5	0.5	0.5	0.5	0.5	0.5	0.5	0.333333	0.333333	0.333333	0.333333	0.5	0.5	0.333333	0.333333	0.333333	0.5	0.5
M00007	0.833333	0.833333	0.833333	0.833333	0.833333	0.833333	0.833333	0.833333	0.833333	0.833333	0.833333	0.833333	0.833333	1	0.833333	0.833333	0.833333	1	0.833333	0.666667	0.666667	0.666667	0.666667	0.666667	0.666667	0.833333	0.833333	0.666667	0.833333	0.833333	0.833333	1	0.833333	0.666667	0.666667	0.833333	0.833333
M00008	0.8	0.8	0.8	0.8	0.8	0.8	0.8	0.8	0.8	1	0.8	0.8	0.8	0.8	0.8	0.8	0.8	0.8	0.8	0.8	0.8	0.8	0.8	0.8	0.8	0.8	0.6	0.6	0.4	0.4	0.4	0.6	0.6	0.6	0.6	0.8	0.6
M00009	0.538462	0.512821	0.512821	0.538462	0.512821	0.435897	0.538462	0.487179	0.461538	0.487179	0.410256	0.512821	0.487179	0.461538	0.487179	0.487179	0.487179	0.435897	0.487179	0.435897	0.435897	0.461538	0.435897	0.435897	0.435897	0.435897	0.512821	0.487179	0.615385	0.589744	0.512821	0.564103	0.538462	0.487179	0.435897	0.538462	0.589744
M00010	1	0.8	0.8	1	0.8	0.8	1	0.8	0.6	0.8	0.6	0.8	0.8	0.8	0.8	0.8	0.8	0.8	0.8	0.8	0.8	0.8	0.8	0.8	0.8	0.8	0.8	0.8	1	0.8	0.8	0.8	0.8	0.8	0.8	0.8	0.8
M00011	0.470588	0.470588	0.470588	0.470588	0.470588	0.382353	0.470588	0.441176	0.441176	0.441176	0.382353	0.470588	0.441176	0.411765	0.441176	0.441176	0.441176	0.382353	0.441176	0.382353	0.382353	0.411765	0.382353	0.382353	0.382353	0.382353	0.470588	0.441176	0.558824	0.558824	0.470588	0.529412	0.5	0.441176	0.382353	0.5	0.558824
M00012	0.666667	0.666667	0.666667	0.666667	0.666667	0.444444	0.666667	0.666667	0.555556	0.555556	0.555556	0.666667	0.666667	0.555556	0.666667	0.666667	0.666667	0.555556	0.666667	0.666667	0.666667	0.666667	0.666667	0.666667	0.666667	0.666667	0.666667	0.666667	0.666667	0.666667	0.666667	0.555556	0.666667	0.666667	0.666667	0.666667	0.666667
M00013	0.333333	0.333333	0.333333	0.333333	0.333333	0.333333	0.333333	0.333333	0.333333	0.333333	0.333333	0.333333	0.333333	0.333333	0.333333	0.333333	0.333333	0.333333	0.333333	0.333333	0.333333	0.333333	0.333333	0.333333	0.333333	0.333333	0.333333	0.333333	0.333333	0.333333	0.333333	0.333333	0.333333	0.333333	0.333333	0.333333	0.333333
M00014	0.166667	0.166667	0.166667	0.166667	0.166667	0.166667	0.166667	0.25	0.166667	0.25	0.166667	0.166667	0.166667	0.333333	0.25	0.25	0.083333	0.25	0.25	0.083333	0.083333	0.166667	0.083333	0.166667	0.166667	0.166667	0.083333	0.083333	0.25	0.083333	0.083333	0.166667	0.083333	0.083333	0.083333	0.083333	0.25
M00015	0.75	0.75	0.75	0.75	0.75	0.75	0.75	0.75	0.75	0.75	0.75	0.75	0.75	0.75	0.75	0.75	0.75	0.75	0.75	0.75	0.75	0.75	0.75	0.75	0.75	0.75	0.75	0.75	0.75	0.75	0.75	0.75	0.75	0.75	0.75	0.75	0.75
M00016	0.714286	0.714286	0.714286	0.714286	0.714286	0.642857	0.714286	0.642857	0.642857	0.714286	0.642857	0.642857	0.642857	0.642857	0.642857	0.714286	0.714286	0.714286	0.642857	0.714286	0.714286	0.714286	0.714286	0.714286	0.714286	0.714286	0.714286	0.714286	0.642857	0.714286	0.714286	0.714286	0.642857	0.714286	0.642857	0.714286	0.642857
M00017	0.538462	0.538462	0.461538	0.538462	0.461538	0.461538	0.538462	0.538462	0.538462	0.538462	0.538462	0.538462	0.538462	0.538462	0.538462	0.538462	0.538462	0.538462	0.538462	0.538462	0.461538	0.461538	0.461538	0.461538	0.538462	0.538462	0.461538	0.461538	0.461538	0.461538	0.461538	0.615385	0.461538	0.461538	0.461538	0.461538	0.461538
M00018	0.416667	0.333333	0.333333	0.416667	0.333333	0.333333	0.416667	0.5	0.416667	0.5	0.416667	0.416667	0.416667	0.5	0.416667	0.5	0.416667	0.5	0.416667	0.416667	0.416667	0.416667	0.416667	0.416667	0.416667	0.416667	0.416667	0.416667	0.5	0.416667	0.416667	0.5	0.416667	0.416667	0.416667	0.416667	0.416667
M00019	0.833333	0.833333	0.833333	0.833333	0.833333	0.833333	0.833333	0.833333	0.833333	0.833333	0.833333	0.833333	0.833333	0.833333	0.833333	0.833333	0.833333	0.833333	0.833333	0.833333	0.833333	0.833333	0.833333	0.833333	0.833333	0.833333	0.833333	0.833333	0.833333	0.833333	0.833333	0.833333	0.833333	0.833333	0.833333	0.833333	0.833333
M00020	1	1	1	1	1	1	1	1	1	1	1	1	1	1	1	1	1	1	1	1	1	1	1	1	1	1	1	1	1	1	1	1	1	1	1	1	1
M00021	0.6	0.6	0.6	0.6	0.6	0.4	0.6	0.6	0.4	0.6	0.4	0.4	0.6	0.6	0.4	0.6	0.6	0.6	0.6	0.6	0.6	0.6	0.6	0.6	0.6	0.6	0.6	0.6	0.6	0.6	0.6	0.6	0.6	0.6	0.6	0.6	0.6
M00022	0.6	0.533333	0.533333	0.533333	0.533333	0.533333	0.533333	0.6	0.466667	0.533333	0.6	0.466667	0.533333	0.533333	0.533333	0.533333	0.466667	0.6	0.466667	0.466667	0.466667	0.466667	0.466667	0.466667	0.466667	0.466667	0.533333	0.533333	0.6	0.533333	0.533333	0.666667	0.533333	0.533333	0.533333	0.533333	0.6
M00023	0.5	0.4375	0.5	0.4375	0.4375	0.4375	0.4375	0.5	0.4375	0.5	0.4375	0.5	0.4375	0.5	0.4375	0.5	0.4375	0.5	0.4375	0.5	0.5	0.5	0.5	0.5	0.5	0.5	0.5	0.5	0.5	0.5	0.5	0.5	0.5	0.5	0.5	0.5	0.5
M00024	0.142857	0.214286	0.142857	0.142857	0.142857	0.214286	0.214286	0.285714	0.142857	0.285714	0.142857	0.142857	0.285714	0.214286	0.214286	0.285714	0.285714	0.142857	0.214286	0.285714	0.214286	0.285714	0.214286	0.214286	0.285714	0.285714	0.142857	0.142857	0.142857	0.142857	0.142857	0.214286	0.142857	0.142857	0.142857	0.214286	0.214286
M00025	0.142857	0.214286	0.214286	0.214286	0.214286	0.214286	0.214286	0.285714	0.071429	0.285714	0.071429	0.142857	0.285714	0.214286	0.214286	0.285714	0.285714	0.142857	0.285714	0.285714	0.214286	0.214286	0.214286	0.214286	0.214286	0.285714	0.214286	0.214286	0.214286	0.214286	0.214286	0.214286	0.214286	0.214286	0.214286	0.214286	0.214286
M00026	0.578947	0.578947	0.578947	0.578947	0.578947	0.578947	0.578947	0.578947	0.578947	0.631579	0.578947	0.631579	0.578947	0.526316	0.631579	0.578947	0.526316	0.631579	0.631579	0.578947	0.578947	0.578947	0.578947	0.578947	0.578947	0.578947	0.526316	0.526316	0.578947	0.578947	0.578947	0.578947	0.526316	0.526316	0.526316	0.578947	0.578947
M00027	0.142857	0.142857	0.142857	0.142857	0.142857	0.285714	0.142857	0.142857	0.142857	0.428571	0.142857	0.142857	0.142857	0.428571	0.142857	0.285714	0.142857	0.428571	0.285714	0.285714	0.142857	0.285714	0.142857	0.142857	0.142857	0.142857	0.142857	0.142857	0.142857	0.142857	0.142857	0.428571	0.142857	0.142857	0.142857	0.142857	0.142857
M00028	0.571429	0.5	0.571429	0.5	0.5	0.5	0.5	0.428571	0.357143	0.5	0.357143	0.357143	0.428571	0.428571	0.357143	0.428571	0.5	0.5	0.428571	0.5	0.5	0.571429	0.5	0.5	0.5	0.5	0.428571	0.428571	0.5	0.5	0.571429	0.571429	0.357143	0.428571	0.357143	0.5	0.571429
M00029	0.666667	0.5	0.5	0.666667	0.5	0.5	0.666667	0.666667	0.5	0.666667	0.5	0.666667	0.666667	0.666667	0.5	0.666667	0.666667	0.666667	0.666667	0.666667	0.666667	0.666667	0.666667	0.666667	0.666667	0.666667	0.666667	0.5	0.666667	0.666667	0.666667	0.666667	0.666667	0.5	0.5	0.666667	0.666667
M00030	0	0	0	0	0	0.1	0	0	0	0	0	0	0.1	0	0	0	0	0	0	0	0	0	0	0	0	0	0	0	0	0	0	0	0	0	0	0	0
M00031	0	0	0	0	0	0	0	0	0	0.166667	0	0	0	1	0	0	0	1	0	0	0	0	0	0	0	0	0	0	0	0	0	0.166667	0	0	0	0	0
M00032	0.277778	0.277778	0.277778	0.277778	0.277778	0.333333	0.277778	0.333333	0.277778	0.277778	0.277778	0.277778	0.333333	0.277778	0.333333	0.333333	0.333333	0.277778	0.333333	0.333333	0.333333	0.277778	0.333333	0.333333	0.333333	0.333333	0.222222	0.277778	0.222222	0.222222	0.277778	0.277778	0.222222	0.277778	0.222222	0.277778	0.277778
M00033	0.4	0.6	0.6	0.6	0.6	0.4	0.6	0.4	0.4	0.4	0.4	0.4	0.4	0.4	0.4	0.4	0.4	0.4	0.4	0.6	0.6	0.6	0.6	0.6	0.6	0.4	0.4	0.4	0.4	0.4	0.4	0.4	0.4	0.4	0.4	0.6	0.6
M00034	0.210526	0.263158	0.263158	0.263158	0.263158	0.315789	0.263158	0.368421	0.210526	0.263158	0.263158	0.315789	0.315789	0.368421	0.210526	0.315789	0.263158	0.368421	0.263158	0.210526	0.263158	0.263158	0.210526	0.210526	0.210526	0.263158	0.368421	0.368421	0.368421	0.368421	0.368421	0.315789	0.368421	0.368421	0.368421	0.368421	0.368421
M00035	0.428571	0.428571	0.428571	0.428571	0.428571	0.571429	0.428571	0.428571	0.428571	0.571429	0.428571	0.428571	0.428571	0.428571	0.428571	0.428571	0.428571	0.571429	0.428571	0.428571	0.428571	0.428571	0.428571	0.428571	0.428571	0.428571	0.428571	0.428571	0.428571	0.428571	0.428571	0.571429	0.428571	0.428571	0.428571	0.428571	0.428571
M00036	0.615385	0.615385	0.615385	0.615385	0.615385	0.692308	0.615385	0.846154	0.692308	0.692308	0.615385	0.615385	0.846154	0.846154	0.846154	0.846154	0.846154	0.769231	0.846154	0.692308	0.615385	0.615385	0.615385	0.615385	0.615385	0.615385	0.615385	0.615385	0.615385	0.615385	0.615385	0.692308	0.615385	0.615385	0.615385	0.615385	0.615385
M00038	0.2	0.2	0	0.2	0	0.2	0.2	0.4	0.4	0.5	0.1	0	0.4	0.2	0.4	0.4	0.4	0.3	0.4	0.2	0.3	0.4	0.2	0.1	0.3	0.4	0	0.3	0	0	0	0.2	0.3	0.3	0.3	0.3	0
M00039	0	0	0	0	0	0	0	0	0	0	0	0	0	0	0	0	0	0	0	0	0	0	0	0	0	0	0	0	0	0	0	0	0	0	0	0	0
M00040	0	0.2	0.2	0.2	0.2	0.4	0.2	0.4	0.4	0	0.4	0.4	0.4	0.2	0.4	0.4	0.4	0	0.4	0.2	0.2	0.2	0.2	0.2	0.2	0.2	0.2	0.2	0.2	0.2	0.2	0	0.2	0.2	0.2	0.2	0.2
M00042	0	0	0	0	0	0	0	0	0	0	0	0	0	0	0	0	0	0	0	0	0	0.166667	0	0	0	0	0	0	0	0	0	0.166667	0	0	0	0	0
M00044	0.5	0.5	0.333333	0.5	0.333333	0.333333	0.5	0.833333	0.833333	0.5	0.666667	0.666667	0.833333	0.5	0.833333	0.833333	0.833333	0.5	0.833333	0.666667	0.833333	0.666667	0.666667	0.666667	0.666667	0.833333	0.5	0.5	0.166667	0.666667	0.333333	0.5	0.666667	0.5	0.5	0.833333	0.333333
M00045	0.5	0.5	0.5	0.5	0.5	0.25	0.5	0.75	0	1	0.25	0	0.25	0.5	0	0.75	0.25	0.75	0	1	1	1	1	1	1	1	0	0	0.5	0.5	0	1	0	0	0	1	1
M00046	0	0	0	0	0	0.333333	0	0	0	0.333333	0	0	0	0	0	0	0	0	0	0	0	0.333333	0	0	0	0	0	0	0.333333	0	0	0	0	0	0	0	0
M00047	0	0	0	0	0	0	0	0	0	0	0	0	0	0	0	0	0	0	0	0	0	0	0	0	0	0	0	0	0	0	0	0	0	0	0	0	0
M00048	0.44	0.44	0.44	0.44	0.44	0.4	0.44	0.44	0.44	0.4	0.4	0.44	0.44	0.4	0.48	0.44	0.44	0.4	0.48	0.4	0.4	0.4	0.4	0.4	0.4	0.44	0.44	0.44	0.44	0.44	0.44	0.4	0.44	0.44	0.44	0.44	0.4
M00049	0.555556	0.555556	0.555556	0.555556	0.555556	0.555556	0.555556	0.555556	0.555556	0.555556	0.555556	0.555556	0.555556	0.555556	0.555556	0.555556	0.555556	0.555556	0.555556	0.555556	0.555556	0.555556	0.555556	0.555556	0.555556	0.555556	0.555556	0.555556	0.555556	0.555556	0.555556	0.555556	0.555556	0.555556	0.555556	0.555556	0.555556
M00050	0.833333	0.833333	0.833333	0.833333	0.833333	0.833333	0.833333	0.833333	0.833333	0.833333	0.833333	0.833333	0.833333	0.833333	0.833333	0.833333	0.833333	0.833333	0.833333	0.833333	0.833333	0.833333	0.833333	0.833333	0.833333	0.833333	0.833333	0.833333	0.833333	0.833333	0.833333	0.833333	0.833333	0.833333	0.833333	0.833333	0.833333
M00051	0.5	0.4375	0.5	0.5	0.4375	0.4375	0.4375	0.4375	0.4375	0.5	0.4375	0.4375	0.4375	0.5	0.4375	0.4375	0.4375	0.5	0.4375	0.4375	0.4375	0.4375	0.4375	0.4375	0.4375	0.4375	0.4375	0.4375	0.5	0.5	0.5	0.5	0.5	0.4375	0.4375	0.4375	0.5
M00052	0.428571	0.428571	0.428571	0.428571	0.428571	0.428571	0.428571	0.428571	0.428571	0.428571	0.428571	0.428571	0.428571	0.428571	0.428571	0.428571	0.428571	0.428571	0.428571	0.428571	0.428571	0.428571	0.428571	0.428571	0.428571	0.428571	0.428571	0.428571	0.428571	0.428571	0.428571	0.428571	0.428571	0.285714	0.285714	0.428571	0.428571
M00053	0.571429	0.5	0.571429	0.571429	0.571429	0.5	0.5	0.571429	0.5	0.5	0.5	0.571429	0.5	0.5	0.5	0.571429	0.5	0.5	0.5	0.5	0.5	0.571429	0.5	0.5	0.5	0.5	0.571429	0.571429	0.571429	0.571429	0.571429	0.571429	0.571429	0.571429	0.571429	0.571429	0.571429
M00055	0	0	0	0	0	0	0	0	0	0	0	0	0	0	0	0	0	0	0	0	0	0	0	0	0	0	0	0	0	0	0	0	0	0	0	0	0
M00060	0.9	0.9	0.9	0.9	0.8	0.8	0.9	0.7	0.7	0.3	0.7	0.7	0.7	0.7	0.7	0.7	0.8	0.4	0.7	0.8	0.8	0.8	0.8	0.8	0.8	0.8	0.8	0.8	0.8	0.8	0.8	0.9	0.8	0.8	0.8	0.8	0.8
M00061	0.142857	0.142857	0.142857	0.142857	0.142857	0.285714	0.142857	0.714286	0.714286	0.714286	0.714286	0.714286	0.571429	0.714286	0.714286	0.714286	0.142857	0.428571	0.714286	0.714286	0.714286	0.428571	0.714286	0.714286	0.857143	0.857143	0.142857	0.142857	0.142857	0	0	0.285714	0.142857	0.142857	0.142857	0.428571	0.142857
M00063	1	1	1	1	1	1	1	1	0.75	0.75	0.75	0.75	1	1	0.75	1	1	0.5	1	1	1	1	1	1	1	1	1	1	1	1	1	1	1	1	1	1	1
M00064	0.75	0.75	0.75	0.75	0.75	0.75	0.75	0.75	0.25	0.75	0.5	0.5	1	0.75	0.5	1	0.5	0.75	0.5	0.5	0.5	0.75	0.5	0.5	0.5	0.5	0.5	0.5	0.75	0.5	1	1	0.5	0.75	0.5	0.5	0.75
M00066	0	0	0	0	0	0	0	0	0	0	0.5	0	0.5	0.5	0.5	0.5	0	0.5	0	0	0	0	0	0	0	0	0	0	0	0	0	0	0	0	0	0	0
M00072	0	0	0	0	0	0	0	0	0	0	0	0	0	0	0	0	0	0	0	0	0	0	0	0	0	0	0	0	0	0	0	0	0	0	0	0	0
M00073	0	0	0	0	0	0	0	0	0	0	0	0	0	0	0	0	0	0	0	0	0	0	0	0	0	0	0	0	0	0	0	0	0	0	0	0	0
M00075	0	0	0	0	0	0	0	0	0	0	0	0	0	0	0	0	0	0	0	0	0	0	0	0	0	0	0	0	0	0	0	0	0	0	0	0	0
M00076	0	0	0	0	0	0	0	0	0	0	0	0	0	0	0	0	0	0	0	0	0	0	0	0	0	0	0	0	0	0	0	0	0	0	0	0	0
M00077	0	0	0	0	0	0	0	0	0.25	0	0	0.5	0	0.25	0.5	0	0	0	0.5	0	0	0	0	0	0	0	0	0	0	0	0	0	0	0	0	0	0
M00078	0	0	0	0	0	0.111111	0	0	0.222222	0	0.111111	0.222222	0.111111	0.222222	0.222222	0.111111	0	0	0.222222	0	0	0	0	0	0	0	0	0	0	0	0	0	0	0	0	0	0
M00080	0.333333	0.166667	0.333333	0.166667	0.166667	0.25	0.166667	0.25	0.083333	0.083333	0.083333	0.083333	0.25	0.25	0.083333	0.25	0.333333	0.25	0.083333	0.25	0.25	0.333333	0.166667	0.083333	0.25	0.333333	0.333333	0.333333	0.25	0.333333	0.5	0.333333	0.333333	0.5	0.25	0.333333	0.333333
M00081	0	0	0	0	0	0.333333	0	0.333333	0.333333	0	0.333333	0.333333	0.333333	0.333333	0.333333	0.333333	0.333333	0	0.333333	0.333333	0	0.666667	0.333333	0.333333	0.333333	0.333333	0	0	0	0	0	0	0	0	0	0	0
M00082	0.428571	0.428571	0.428571	0.428571	0.428571	0.428571	0.428571	0.428571	0.428571	0.571429	0.428571	0.428571	0.428571	0.5	0.428571	0.428571	0.428571	0.571429	0.428571	0.428571	0.428571	0.428571	0.428571	0.428571	0.5	0.5	0.428571	0.428571	0.428571	0.428571	0.428571	0.428571	0.428571	0.428571	0.428571	0.428571	0.428571
M00083	0.533333	0.533333	0.466667	0.6	0.466667	0.533333	0.6	0.533333	0.533333	0.333333	0.466667	0.533333	0.466667	0.466667	0.533333	0.466667	0.466667	0.333333	0.533333	0.466667	0.466667	0.466667	0.466667	0.466667	0.466667	0.466667	0.4	0.466667	0.466667	0.466667	0.4	0.533333	0.466667	0.466667	0.466667	0.466667	0.466667
M00085	0	0	0	0	0	0	0	0	0	0	0	0	0	0	0	0	0	0	0	0	0	0	0	0	0	0	0	0	0	0	0	0	0	0	0	0	0
M00086	0.5	0.5	0.5	0.5	0.5	0.5	0.5	0.5	0.5	0.5	0.5	0.5	0.5	0.5	0.5	0.5	0.5	0.5	0.5	0.5	0.5	0.5	0.5	0.5	0.5	0.5	0.5	0.5	0.5	0.5	0.5	0.5	0.5	0.5	0.5	0.5	0.5
M00087	0.368421	0.368421	0.368421	0.368421	0.368421	0.263158	0.315789	0.263158	0.263158	0.368421	0.263158	0.263158	0.263158	0.263158	0.263158	0.263158	0.263158	0.315789	0.315789	0.368421	0.421053	0.315789	0.421053	0.421053	0.421053	0.421053	0.263158	0.263158	0.210526	0.263158	0.315789	0.315789	0.263158	0.315789	0.210526	0.315789	0.315789
M00088	0.6	0.6	0.4	0.6	0.4	0.6	0.6	0.6	0.6	0.6	0.6	0.6	0.6	0.6	0.6	0.6	0.6	0.6	0.6	0.8	0.8	0.8	0.8	0.8	0.8	0.8	0.6	0.6	0.6	0.6	0.6	0.6	0.6	0.6	0.6	0.6	0.6
M00089	0.142857	0.142857	0.142857	0.142857	0.190476	0.142857	0.190476	0.142857	0.142857	0.238095	0.142857	0.142857	0.142857	0.142857	0.142857	0.142857	0.142857	0.142857	0.142857	0.190476	0.238095	0.142857	0.238095	0.238095	0.238095	0.238095	0.142857	0.142857	0.142857	0.142857	0.142857	0.142857	0.142857	0.142857	0.142857	0.190476	0.142857
M00090	0	0	0	0	0	0	0	0	0	0	0	0	0	0	0	0	0	0	0	0	0	0	0	0	0	0	0	0	0	0	0	0	0	0	0	0	0
M00091	0	0	0	0	0	0	0	0.25	0	0	0.25	0.25	0	0	0	0	0	0	0	0	0	0	0	0	0	0	0	0	0	0	0	0	0	0	0	0	0
M00092	0	0	0	0	0	0	0	0	0	0	0	0	0	0	0	0	0	0	0	0	0	0	0	0	0	0	0	0	0	0	0	0	0	0	0	0	0
M00093	0.75	0.75	0.75	0.75	0.75	0.75	0.75	0.75	0.75	0.75	0.75	0.75	0.75	0.75	0.75	0.75	0.75	0.75	0.75	0.75	0.75	0.75	0.75	0.75	0.75	0.75	0.75	0.75	0.75	0.75	0.75	0.75	0.75	0.75	0.75	0.75	0.75
M00095	0.125	0.125	0.125	0.125	0.125	0.125	0.125	0.125	0.125	0.25	0.125	0.125	0.125	0.25	0.125	0.125	0.125	0.25	0.125	0.125	0.125	0.125	0.125	0.125	0.125	0.125	0.125	0.125	0.125	0.125	0.125	0.25	0.125	0.125	0.125	0.125	0.125
M00096	0.8	0.8	0.8	0.7	0.8	0.8	0.7	0.8	0.6	0.9	0.6	0.6	0.8	0.9	0.6	0.8	0.8	0.8	0.8	0.8	0.8	0.8	0.8	0.8	0.8	0.8	0.7	0.7	0.8	0.8	0.7	0.9	0.7	0.7	0.7	0.8	0.8
M00097	0.166667	0.166667	0.166667	0.166667	0.166667	0.166667	0.166667	0.333333	0.166667	0.333333	0.166667	0.166667	0.333333	0.166667	0.333333	0.333333	0.333333	0.166667	0.333333	0.166667	0.166667	0.166667	0.166667	0.166667	0.166667	0.166667	0.166667	0.166667	0.166667	0.166667	0.166667	0.166667	0.166667	0.166667	0.166667	0.166667	0.166667
M00098	0	0	0	0	0	0.076923	0	0	0	0.076923	0	0	0	0	0	0	0	0.076923	0	0.076923	0.076923	0.076923	0.076923	0.076923	0.076923	0.076923	0	0	0	0	0	0	0	0	0	0.076923	0
M00099	0	0	0	0	0	0	0	0	0	0	0	0	0	0	0	0	0	0	0	0	0	0	0	0	0	0	0	0	0	0	0	0	0	0	0	0	0
M00101	0	0	0	0	0	0	0	0	0	0	0	0	0	0	0	0	0	0	0	0	0	0	0	0	0	0	0	0	0	0	0	0	0	0	0	0	0
M00102	0	0	0	0	0	0	0	0	0	0	0	0	0	0	0	0	0	0	0	0	0	0	0	0	0	0	0	0	0	0	0	0	0	0	0	0	0
M00104	0	0	0	0	0	0	0	0.166667	0.083333	0.083333	0	0.083333	0.166667	0	0.166667	0.166667	0.166667	0.083333	0.166667	0.083333	0.083333	0.083333	0.083333	0.083333	0.083333	0.083333	0	0.083333	0	0	0	0	0.083333	0.083333	0.083333	0.083333	0
M00107	0	0	0	0	0	0	0	0	0	0	0	0	0	0	0	0	0	0	0	0	0	0	0	0	0	0.333333	0	0.333333	0	0	0	0	0.333333	0.333333	0.333333	0.333333	0
M00112	0	0	0	0	0	0	0	0	0	0	0	0	0	0	0	0	0	0	0	0	0	0	0	0	0	0	0	0	0	0	0	0	0	0	0	0	0
M00114	0.35	0.25	0.3	0.3	0.25	0.3	0.25	0.35	0.35	0.3	0.25	0.35	0.3	0.35	0.35	0.35	0.3	0.3	0.35	0.3	0.3	0.3	0.3	0.3	0.3	0.3	0.3	0.3	0.4	0.35	0.35	0.45	0.25	0.25	0.25	0.35	0.35
M00115	0.857143	0.857143	0.857143	0.857143	0.857143	0.857143	0.857143	0.857143	0.857143	0.714286	0.857143	0.857143	0.857143	0.857143	0.857143	0.857143	0.857143	0.857143	0.714286	0.857143	0.857143	0.857143	0.857143	0.857143	0.857143	0.857143	0.857143	0.714286	0.857143	0.857143	0.857143	0.857143	0.714286	0.857143	0.714286	0.857143	0.857143
M00116	0.166667	0.083333	0.083333	0.083333	0.083333	0.083333	0.083333	0.083333	0.083333	0.666667	0.083333	0.083333	0.083333	0.666667	0.083333	0.083333	0.083333	0.666667	0.083333	0.166667	0.166667	0.083333	0.166667	0.166667	0.166667	0.25	0.166667	0.166667	0.166667	0.166667	0.166667	0.666667	0.166667	0.166667	0.083333	0.25	0.166667
M00117	0.545455	0.545455	0.545455	0.545455	0.545455	0.545455	0.545455	0.545455	0.545455	0.363636	0.545455	0.545455	0.545455	0.545455	0.545455	0.545455	0.545455	0.181818	0.545455	0.636364	0.636364	0.636364	0.636364	0.636364	0.636364	0.636364	0.636364	0.636364	0.454545	0.636364	0.636364	0.454545	0.636364	0.636364	0.545455	0.636364	0.545455
M00118	0.5	0.5	0.5	0.5	0.5	0.5	0.5	0.5	0.5	0.5	0.5	0.5	0.5	0.5	0.5	0.5	0.5	0	0.5	0.5	0.5	0.5	0.5	0.5	0.5	0.5	0.5	0.5	0.25	0.5	0.5	0.5	0.5	0.5	0.5	0.5	0.5
M00119	0.833333	0.833333	0.833333	0.833333	0.833333	0.833333	0.833333	0.833333	0.833333	0.833333	0.666667	0.833333	0.833333	0.833333	0.833333	0.833333	0.833333	0.833333	0.833333	0.833333	0.833333	0.833333	0.833333	0.833333	0.833333	0.833333	0.833333	0.833333	0.833333	0.833333	0.833333	0.833333	0.833333	0.833333	0.833333	0.833333	0.833333
M00120	0.363636	0.363636	0.363636	0.363636	0.363636	0.363636	0.363636	0.454545	0.363636	0.454545	0.454545	0.363636	0.363636	0.363636	0.363636	0.363636	0.363636	0.454545	0.363636	0.454545	0.454545	0.454545	0.454545	0.454545	0.454545	0.454545	0.363636	0.363636	0.363636	0.363636	0.363636	0.363636	0.363636	0.363636	0.363636	0.363636	0.363636
M00121	0.652174	0.565217	0.652174	0.652174	0.608696	0.565217	0.565217	0.565217	0.478261	0.695652	0.521739	0.478261	0.565217	0.695652	0.521739	0.608696	0.565217	0.652174	0.521739	0.521739	0.521739	0.521739	0.521739	0.521739	0.565217	0.565217	0.565217	0.565217	0.565217	0.652174	0.652174	0.652174	0.565217	0.565217	0.521739	0.565217	0.652174
M00122	0.888889	0.888889	0.888889	0.888889	1	1	1	1	0.222222	0.888889	0.888889	0.666667	0.888889	0.555556	0.222222	0.888889	0.888889	0.888889	0.222222	1	1	1	1	1	1	1	0.888889	0.888889	0.888889	0.888889	1	0.888889	0.888889	1	0.888889	1	0.888889
M00123	0.666667	0.666667	0.666667	0.666667	0.666667	0.666667	0.666667	0.666667	0.333333	0.666667	0.666667	0.333333	0.666667	0.333333	0.333333	0.666667	0.666667	0.666667	0.5	0.666667	0.666667	0.666667	0.666667	0.666667	0.666667	0.666667	0.666667	0.666667	0.666667	0.666667	0.666667	0.666667	0.666667	0.666667	0.666667	0.666667	0.666667

KEGG_Module	1 if median >= 90% for the samples per site; -1 if median <= 10% for the samples per site												Kind of Module inferred (Core or Varied)
	India_oil_refineries	Arctic	China_oil_refineries	Mangrove	Marine_sediments	Taiga_upper_active_layer	Taiga_bottom_active_layer	Taiga_permafrost_layer	Oil_sands_core	Oil_sands_tailings_pond_upper	Oil_sands_tailings_pond_median	Oil_sands_tailings_pond_deep	
M00001	0	0	0	0	0	0	0	0	0	0	0	0	
M00002	0	0	0	0	0	0	0	0	0	0	0	0	
M00003	0	0	0	0	0	0	0	0	0	0	0	0	
M00004	0	0	0	0	0	0	0	0	0	0	0	0	
M00005	1	1	1	1	1	1	1	1	1	1	1	1	core
M00006	0	0	0	0	0	0	0	0	0	0	0	0	
M00007	0	0	1	1	0	0	0	0	0	0	0	0	
M00008	0	0	0	0	0	0	0	0	0	0	0	0	
M00009	0	0	0	0	0	0	0	0	0	0	0	0	
M00010	1	0	0	1	0	0	0	0	0	0	0	0	
M00011	0	0	0	0	0	0	0	0	0	0	0	0	
M00012	0	0	0	0	0	0	0	0	0	0	0	0	
M00013	0	0	0	0	0	0	0	0	0	0	0	0	
M00014	0	0	0	0	0	0	0	0	0	-1	-1	-1	
M00015	0	0	0	0	0	0	0	0	0	0	0	0	
M00016	0	0	0	0	0	0	0	0	0	0	0	0	
M00017	0	0	0	0	0	0	0	0	0	0	0	0	
M00018	0	0	0	0	0	0	0	0	0	0	0	0	
M00019	0	0	0	0	0	0	0	0	0	0	0	0	
M00020	1	1	1	1	1	1	1	1	1	1	1	1	core
M00021	0	0	0	0	0	0	0	0	0	0	0	0	
M00022	0	0	0	0	0	0	0	0	0	0	0	0	
M00023	0	0	0	0	0	0	0	0	0	0	0	0	
M00024	0	0	0	0	0	0	0	0	0	0	0	0	
M00025	0	0	0	0	0	0	0	0	0	0	0	0	
M00026	0	0	0	0	0	0	0	0	0	0	0	0	
M00027	0	0	0	0	0	0	0	0	0	0	0	0	
M00028	0	0	0	0	0	0	0	0	0	0	0	0	
M00029	0	0	0	0	0	0	0	0	0	0	0	0	
M00030	-1	-1	-1	-1	-1	-1	-1	-1	-1	-1	-1	-1	
M00031	-1	-1	0	-1	-1	-1	-1	-1	-1	-1	-1	-1	
M00032	0	0	0	0	0	0	0	0	0	0	0	0	
M00033	0	0	0	0	0	0	0	0	0	0	0	0	
M00034	0	0	0	0	0	0	0	0	0	0	0	0	
M00035	0	0	0	0	0	0	0	0	0	0	0	0	
M00036	0	0	0	0	0	0	0	0	0	0	0	0	
M00038	0	0	0	-1	0	0	0	0	0	-1	0	0	
M00039	-1	-1	-1	-1	-1	-1	-1	-1	-1	-1	-1	-1	
M00040	0	-1	0	-1	0	0	0	0	0	0	0	0	
M00042	-1	-1	-1	0	-1	-1	-1	-1	-1	-1	-1	-1	
M00044	0	0	0	0	0	0	0	0	0	0	0	0	
M00045	0	0	0	0	0	0	0	0	1	0	-1	0	varied
M00046	-1	-1	-1	-1	-1	-1	-1	-1	-1	-1	-1	-1	
M00047	-1	-1	-1	-1	-1	-1	-1	-1	-1	-1	-1	-1	
M00048	0	0	0	0	0	0	0	0	0	0	0	0	
M00049	0	0	0	0	0	0	0	0	0	0	0	0	
M00050	0	0	0	0	0	0	0	0	0	0	0	0	
M00051	0	0	0	0	0	0	0	0	0	0	0	0	
M00052	0	0	0	0	0	0	0	0	0	0	0	0	
M00053	0	0	0	0	0	0	0	0	0	0	0	0	
M00055	-1	-1	-1	-1	-1	-1	-1	-1	-1	-1	-1	-1	
M00060	0	0	0	0	0	0	0	0	0	0	0	0	
M00061	0	0	0	0	0	0	0	0	0	0	0	0	
M00063	1	1	1	1	1	0	0	1	1	1	1	1	
M00064	1	0	1	1	0	0	0	0	0	0	1	0	
M00066	0	-1	0	-1	-1	-1	0	0	-1	-1	-1	-1	
M00072	-1	-1	-1	-1	-1	-1	-1	-1	-1	-1	-1	-1	
M00073	-1	-1	-1	-1	-1	-1	-1	-1	-1	-1	-1	-1	
M00075	-1	-1	-1	-1	-1	-1	-1	-1	-1	-1	-1	-1	
M00076	-1	-1	-1	-1	-1	-1	-1	-1	-1	-1	-1	-1	
M00077	-1	-1	-1	-1	-1	-1	0	-1	-1	-1	-1	-1	
M00078	-1	-1	0	-1	-1	-1	0	-1	-1	-1	-1	-1	
M00080	0	0	0	0	0	-1	0	0	0	0	0	0	
M00081	0	-1	0	-1	-1	0	0	0	0	-1	-1	-1	
M00082	0	0	0	0	0	0	0	0	0	0	0	0	
M00083	0	0	0	0	0	0	0	0	0	0	0	0	
M00085	-1	-1	-1	-1	-1	-1	-1	-1	-1	-1	-1	-1	
M00086	0	0	0	0	0	0	0	0	0	0	0	0	
M00087	0	0	0	0	0	0	0	0	0	0	0	0	
M00088	0	0	0	0	0	0	0	0	0	0	0	0	
M00089	0	0	0	0	0	0	0	0	0	0	0	0	
M00090	-1	-1	-1	-1	-1	-1	-1	-1	-1	-1	-1	-1	
M00091	-1	-1	-1	-1	-1	0	-1	-1	-1	-1	-1	-1	
M00092	-1	-1	-1	-1	-1	-1	-1	-1	-1	-1	-1	-1	
M00093	0	0	0	0	0	0	0	0	0	0	0	0	
M00095	0	0	0	0	0	0	0	0	0	0	0	0	
M00096	0	0	0	0	0	0	0	0	0	0	0	0	
M00097	0	0	0	0	0	0	0	0	0	0	0	0	
M00098	-1	-1	-1	-1	-1	-1	-1	-1	-1	-1	-1	-1	
M00099	-1	-1	-1	-1	-1	-1	-1	-1	-1	-1	-1	-1	
M00101	-1	-1	-1	-1	-1	-1	-1	-1	-1	-1	-1	-1	
M00102	-1	-1	-1	-1	-1	-1	-1	-1	-1	-1	-1	-1	
M00104	-1	-1	-1	-1	-1	-1	0	0	-1	-1	-1	-1	
M00107	-1	-1	-1	-1	-1	-1	-1	-1	-1	-1	-1	0	
M00112	-1	-1	-1	-1	-1	-1	-1	-1	-1	-1	-1	-1	
M00114	0	0	0	0	0	0	0	0	0	0	0	0	
M00115	0	0	0	0	0	0	0	0	0	0	0	0	
M00116	0	0	0	0	-1	-1	-1	-1	0	0	0	0	
M00117	0	0	0	0	0	0	0	0	0	0	0	0	
M00118	0	0	0	0	0	0	0	0	0	0	0	0	
M00119	0	0	0	0	0	0	0	0	0	0	0	0	
M00120	0	0	0	0	0	0	0	0	0	0	0	0	
M00121	0	0	0	0	0	0	0	0	0	0	0	0	
M00122	0	0	0	0	0	0	0	0	1	0	0	1	
M00123	0	0	0	0	0	0	0	0	0	0	0	0	

Table S4. I

KEGG_Module	Habitat																											
	India_oil_refineries				Arctic												China_oil_refineries									Mangrove		
	I1	I2	I3	I4	A1	A10	A11	A12	A2	A3	A4	A5	A6	A7	A8	A9	C1	C2	C3	C4	C5	C6	C7	C8	C9	M1	M2	M3
M00124: Pyridoxal_biosynthesis,_erythrose-4P_=>_pyridoxal-5P	0.666667	0.666667	0.666667	0.666667	0.666667	0.666667	0.666667	0.666667	0.666667	0.666667	0.666667	0.666667	0.666667	0.666667	0.666667	0.666667	0.666667	0.666667	0.666667	0.666667	0.666667	0.833333	0.666667	0.666667	0.666667	0.666667	0.666667	0.666667
M00125: Riboflavin_biosynthesis,_GTP_=>_riboflavin/FMN/FAD	0.5	0.5	0.416667	0.416667	0.416667	0.416667	0.416667	0.416667	0.5	0.416667	0.416667	0.416667	0.416667	0.416667	0.416667	0.416667	0.583333	0.583333	0.583333	0.583333	0.583333	0.5	0.583333	0.583333	0.583333	0.416667	0.416667	0.416667
M00126: Tetrahydrofolate_biosynthesis,_GTP_=>_THF	0.391304	0.347826	0.391304	0.391304	0.347826	0.391304	0.391304	0.391304	0.347826	0.391304	0.391304	0.347826	0.434783	0.434783	0.391304	0.347826	0.391304	0.391304	0.391304	0.391304	0.391304	0.391304	0.391304	0.391304	0.391304	0.391304	0.391304	0.391304
M00127: Thiamine_biosynthesis,_AIR_=>_thiamine-P/thiamine-2P	0.4	0.4	0.4	0.4	0.6	0.5	0.6	0.6	0.4	0.6	0.6	0.6	0.6	0.6	0.6	0.4	0.5	0.4	0.4	0.4	0.4	0.4	0.4	0.4	0.4	0.6	0.6	0.6
M00128: Ubiquinone_biosynthesis,_eukaryotes,_4-hydroxybenzoate_=>_ubiquinone	0.166667	0.166667	0.166667	0.166667	0	0.166667	0.166667	0.166667	0	0	0	0	0	0	0	0.166667	0	0	0	0	0	0.166667	0	0	0.166667	0.166667	0.166667	0.166667
M00129: Ascorbate_biosynthesis,_animals,_glucose-1P_=>_ascorbate	0.428571	0.428571	0.428571	0.428571	0.428571	0.428571	0.428571	0.428571	0.428571	0.428571	0.428571	0.428571	0.428571	0.428571	0.428571	0.428571	0.428571	0.428571	0.428571	0.428571	0.428571	0.428571	0.428571	0.428571	0.428571	0.428571	0.428571	0.428571
M00130: Inositol_phosphate_metabolism,_PI=>_PIP2_=>_Ins(1,4,5)P3_=>_Ins(1,3,4,5)P4	0	0	0	0	0	0	0	0	0	0	0	0	0	0	0	0	0	0	0	0	0	0	0	0	0	0	0	0
M00131: Inositol_phosphate_metabolism,_Ins(1,3,4,5)P4_=>_Ins(1,3,4)P3_=>_myo-inositol	0.125	0.125	0.125	0.125	0.125	0.125	0.125	0.125	0.125	0.125	0.125	0.125	0.125	0.125	0.125	0.125	0.125	0.125	0.125	0.125	0.125	0.125	0.125	0.125	0.125	0.125	0.125	0.125
M00133: Polyamine_biosynthesis,_arginine_=>_agmatine_=>_putrescine_=>_spermidine	0.714286	0.571429	0.857143	0.571429	0.428571	0.428571	0.428571	0.428571	0.571429	0.571429	0.571429	0.571429	0.428571	0.428571	0.571429	0.571429	0.571429	0.571429	0.571429	0.571429	0.714286	0.571429	0.571429	0.714286	0.571429	0.714286	0.714286	0.714286
M00134: Polyamine_biosynthesis,_arginine_=>_ornithine_=>_putrescine	1	1	0.5	1	1	1	1	0.5	0.5	0.5	1	0.5	0.5	0.5	1	0.5	1	0.5	0.5	0.5	0.5	1	0.5	1	1	0.5	0.5	0.5
M00135: GABA_biosynthesis,_eukaryotes,_putrescine_=>_GABA	0.4	0.4	0.4	0.4	0.6	0.2	0.4	0.4	0.4	0.4	0.4	0.4	0.6	0.6	0.4	0.4	0.4	0.4	0.4	0.4	0.4	0.4	0.4	0.4	0.4	0.2	0.4	0.2
M00136: GABA_biosynthesis,_prokaryotes,_putrescine_=>_GABA	0.5	0	0	0	0	0	0	0	0	0	0	0	0	0	0	0	0	0	0	0	0	0	0	0	0	0	0.25	0
M00137: Flavanone_biosynthesis,_phenylalanine_=>_naringenin	0	0	0	0	0	0	0	0	0	0	0	0	0	0	0	0	0	0	0	0	0	0	0	0	0	0	0	0
M00140: C1-unit_interconversion,_prokaryotes	1	0.666667	0.666667	0.666667	1	1	0.666667	0.666667	1	1	1	1	1	0.666667	1	1	1	1	1	1	1	1	1	1	1	1	1	1
M00143: NADH_dehydrogenase_(ubiquinone)_Fe-S_protein/flavoprotein_complex,_mitochondria	0	0	0	0	0	0	0	0	0	0	0	0	0	0	0	0	0	0	0	0	0	0	0	0	0	0.090909	0.090909	0.090909
M00144: NADH:quinone_oxidoreductase,_prokaryotes	0.777778	0.777778	0.777778	0.777778	0.777778	0.777778	0.777778	0.777778	0.777778	0.777778	0.777778	0.777778	0.777778	0.777778	0.777778	0.777778	0.777778	0.777778	0.777778	0.777778	0.777778	0.833333	0.777778	0.777778	0.777778	0.777778	0.777778	0.777778
M00145: NAD(P)H:quinone_oxidoreductase,_chloroplasts_and_cyanobacteria	0	0	0	0	0	0	0	0	0	0	0	0	0.071429	0.071429	0	0	0.071429	0.071429	0.071429	0	0.142857	0	0.142857	0.142857	0.142857	0	0.071429	0
M00146: NADH_dehydrogenase_(ubiquinone)_1_alpha_subcomplex	0	0	0	0	0	0	0	0	0	0	0	0	0	0	0	0	0	0	0	0	0	0	0	0	0	0	0	0
M00149: Succinate_dehydrogenase,_prokaryotes	1	1	1	1	1	1	1	1	1	1	1	1	1	1	1	1	1	1	1	1	1	1	1	1	1	1	1	1
M00150: Fumarate_reductase,_prokaryotes	0	0	0	0	0	0	0	0	0	0	0	0	0	0	0	0	0	0	0.25	0	0	0	0.25	0	0	0.25	0.25	0.25
M00151: Cytochrome_bc1_complex_respiratory_unit	0.4	0.4	0.3	0.3	0.6	0.3	0.6	0.6	0.2	0.1	0.3	0.4	0.3	0.3	0.3	0.4	0.5	0.3	0.4	0.2	0.3	0.6	0.2	0.2	0.3	0.3	0.3	0.3
M00153: Cytochrome_d_ubiquinol_oxidase	1	1	1	1	1	1	1	1	1	1	1	1	1	1	1	1	1	1	1	1	1	1	1	1	1	1	1	1
M00154: Cytochrome_c_oxidase	0.111111	0.111111	0.111111	0.111111	0.055556	0.111111	0.111111	0.111111	0.055556	0.055556	0.055556	0.055556	0.055556	0.055556	0.055556	0.055556	0.055556	0.055556	0.055556	0.055556	0.055556	0.111111	0.055556	0.055556	0.111111	0.055556	0.111111	0.111111
M00155: Cytochrome_c_oxidase,_prokaryotes	0.6	0.8	0.6	0.6	0.6	0.6	0.6	0.6	0.6	0.6	0.6	0.6	0.6	0.6	0.6	0.6	0.8	0.8	0.8	0.8	0.8	0.8	0.8	0.8	0.8	0.8	0.8	0.8
M00156: Cytochrome_c_oxidase,_cbb3-type	0.8	0.8	1	0.8	0.2	0.8	0.8	0.8	0.6	0.4	0.4	0.6	0.2	0	0.6	0.6	0.6	0.8	0.6	0.8	0.8	0.8	0.8	0.8	0.8	1	1	1
M00157: F-type_ATPase,_prokaryotes_and_chloroplasts	1	1	1	1	1	1	1	1	1	1	1	1	1	1	1	1	1	1	1	1	1	1	1	1	1	1	1	1
M00159: V-type_ATPase,_prokaryotes	0	0	0	0	0	0	0	0	0	0	0	0	0	0	0	0	0	0	0	0	0	0	0	0	0	0.666667	0.666667	0.666667
M00160: V-type_ATPase,_eukaryotes	0	0	0	0	0	0	0	0	0	0	0	0	0	0	0	0	0	0	0	0	0	0	0	0	0	0	0	0
M00161: Photosystem_II	0	0	0	0	0	0	0	0	0	0	0	0	0	0	0	0	0	0	0	0	0.166667	0	0.166667	0.333333	0.333333	0	0	0
M00162: Cytochrome_b6f_complex	0	0	0	0	0	0	0	0	0	0	0	0	0	0	0	0	0	0.125	0	0	0.125	0	0.125	0.125	0.125	0	0	0
M00163: Photosystem_I	0	0	0	0	0	0	0	0	0	0	0	0	0	0	0	0	0	0	0	0	0	0	0	0	0	0	0	0
M00165: Reductive_pentose_phosphate_cycle_(Calvin_cycle)	0.434783	0.391304	0.434783	0.434783	0.347826	0.347826	0.347826	0.391304	0.26087	0.304348	0.347826	0.304348	0.304348	0.304348	0.347826	0.304348	0.478261	0.434783	0.521739	0.391304	0.478261	0.391304	0.478261	0.478261	0.434783	0.347826	0.478261	0.347826
M00166: Reductive_pentose_phosphate_cycle,_ribulose-5P_=>_glyceraldehyde-3P	0.571429	0.571429	0.714286	0.714286	0.285714	0.428571	0.285714	0.285714	0.285714	0.285714	0.285714	0.285714	0.285714	0.285714	0.285714	0.285714	0.571429	0.428571	0.714286	0.285714	0.571429	0.428571	0.571429	0.428571	0.571429	0.285714	0.571429	0.285714
M00167: Reductive_pentose_phosphate_cycle,_glyceraldehyde-3P_=>_ribulose-5P	0.375	0.3125	0.3125	0.3125	0.375	0.3125	0.375	0.4375	0.25	0.3125	0.375	0.3125	0.3125	0.3125	0.375	0.3125	0.4375	0.4375	0.4375	0.4375	0.4375	0.375	0.4375	0.5	0.375	0.375	0.4375	0.375
M00168: CAM_(Crassulacean_acid_metabolism),_dark	0.5	0.5	0.5	0.5	0.5	0.5	0.5	0.5	0.5	0.5	0.5	0.5	0.5	0.5	0.5	0.5	0.5	0.5	0.5	0.5	0.5	0.5	0.5	0.5	0.5	0.5	0.5	0.5
M00169: CAM_(Crassulacean_acid_metabolism),_light	1	1	1	1	1	1	1	1	1	1	1	1	1	1	1	0.5	1	1	1	1	1	1	1	1	1	1	1	1
M00170: C4-dicarboxylic_acid_cycle,_phosphoenolpyruvate_carboxykinase_type	0.5	0.25	0.5	0.25	0.25	0.25	0.25	0.25	0.5	0.25	0.25	0.5	0.25	0.25	0.5	0.5	0.5	0.5	0.5	0.5	0.5	0.5	0.5	0.5	0.5	0.5	0.5	0.5
M00172: C4-dicarboxylic_acid_cycle,_NADP_-_malic_enzyme_type	0.75	0.75	0.75	0.75	0.75	0.75	0.75	0.75	0.75	0.75	0.75	0.75	0.75	0.75	0.75	0.5	0.75	0.75	0.75	0.75	0.75	0.75	0.75	0.75	0.75	0.75	0.75	0.75
M00173: Reductive_citrate_cycle_(Arnon-Buchanan_cycle)	0.418605	0.44186	0.465116	0.418605	0.44186	0.44186	0.395349	0.44186	0.395349	0.488372	0.418605	0.418605	0.418605	0.418605	0.418605	0.372093	0.418605	0.488372	0.44186	0.581395	0.534884	0.418605	0.534884	0.488372	0.465116	0.674419	0.604651	0.674419
M00174: Methane_oxidation,_methanotroph,_methane_=>_formaldehyde	0	0	0	0	0	0	0	0	0	0	0	0	0	0	0	0	0	0	0	0	0	0	0	0	0	0	0	0
M00175: Nitrogen_fixation,_nitrogen_=>_ammonia	0	0	0	0	0	0	0	0	0	0.75	0.75	0	0	0	0	0	0	0.75	0	0.75	0.75	0	0.75	0.75	0.75	0.75	0.75	0.75
M00176: Assimilatory_sulfate_reduction,_sulfate_=>_H2S	0.666667	0.5	0.5	0.583333	0.333333	0.5	0.416667	0.5	0.5	0.333333	0.416667	0.333333	0.416667	0.333333	0.583333	0.416667	0.75	0.75	0.75	0.75	0.75	0.75	0.75	0.75	0.75	0.583333	0.583333	0.666667
M00177: Ribosome,_eukaryotes	0	0	0	0	0	0	0	0	0	0	0	0	0	0	0	0	0	0	0	0	0	0	0	0	0	0	0	0
M00178: Ribosome,_bacteria	0.981818	0.981818	0.981818	0.981818	0.981818	0.981818	0.981818	0.981818	0.981818	0.981818	0.981818	0.981818	0.981818	0.981818	0.981818	0.981818	0.981818	0.981818	0.981818	0.981818	0.981818	0.981818	0.981818	0.981818	0.981818	0.981818	0.981818	0.981818
M00183: RNA_polymerase,_bacteria	0.666667	0.666667	0.666667	0.666667	0.666667	0.666667	0.666667	0.666667	0.666667	0.666667	0.666667	0.666667	0.666667	0.666667	0.666667	0.666667	0.666667	0.666667	0.666667	0.666667	0.666667	0.666667	0.666667	0.666667	0.666667	0.666667	0.666667	0.666667
M00185: Sulfate_transport_system	1	1	1	1	0.75	1	1	0.75	0.25	0	0.75	0.75	0	0.25	0.75	0.75	1	1	1	1	1	1	1	1	1	1	1	1
M00186: Tungstate_transport_system	0	1	1	1	0	0.333333	0	0	0	0	0	0	0	0	0	0	0.666667	1	0.666667	1	1	0.666667	1	1	1	0.333333	0	0
M00188: NitT/TauT_family_transport_system	1	1	1	1	1	1	1	1	1	1	1	1	1	1	1	1	1	1	1	1	1	1	1	1	1	1	1	1
M00189: Molybdate_transport_system	0.75	0.75	0.75	0.75	0.75	0.75	0.75	0.75	0.75	0.75	0.75	0.75	0.75	0.75	0.75	0.75	0.75	0.75	0.75	0.75	0.75	0.75	0.75	0.75	0.75	0.75	0.75	0.75
M00190: Iron(III)_transport_system	1	1	1	1	1	1	1	1	0.333333	0	1	1	0	1	1	1	1	1	1	1	1	1	1	1	1	1	1	1
M00191: Thiamine_transport_system	0	0	0	0	0	0	0	0	0	0	0	0	0	0	0	0	0	0	0	0	0	0	0	0.333333	0	0	0	0
M00192: Putative_thiamine_transport_system	0	0	0	0	0	0	0	0	0	0	0	0	0	0	0	0	0	0	0	0	0	0	0	0	0	0	0	0
M00193: Putative_spermidine/putrescine_transport_system	0.75	0.75	0	0.5	1	1	1	1	0.75	1	1	0.75	1	1	0.75	0.75	1	1	1	1	1	1	1	1	1	1	1	1
M00194: Maltose/maltodextrin_transport_system	0	0.2	0.2	0.2	0.2	0.2	0	0	0	0.2	0.2	0.2	0	0	0.2	0.2	0.2	0.2	0.2	0.4	0.2	0.4	0.2	0.2	0.4	0.2	0.4	0.2
M00196: Multiple_sugar_transport_system	0.25	0.25	0.25	0.25	1	0.25	1	1	1	1	1	0.25	1	1	0.25	0.25	1	1	1	1	1	1	1	1	1	1	1	1
M00197: Putative_fructooligosaccharide_transport_system	0.25	0	0.25	0.25	0	0	0	0	0	0	0	0	0	0	0	0	0.25	0.25	0.25	0.25	0.25	0.25	0.25	0.25	0.25	0.25	0.25	0.25
M00198: Putative_sn-glycerol-phosphate_transport_system	0	1	1	0.75	1	0.75	1	0.75	0.75	0.75	1	1	0.75	0.75	1	0.75	0.75	0.75	0.75	1	1	0.75	1	0.5	0.75	0.5	0.5	0
M00199: L-Arabinose/lactose_transport_system	0	0	0	0	0	0	0	0	0	0	0	0	0	0	0	0	0	0	0	0	0	0	0	0	0	0	0	0
M00200: Putative_sorbitol/mannitol_transport_system	0.2	0.2	0.2	0.2	0.2	0.2	0.2	0.4	0.2	0.2	0.2	0.2	0.2	0.2	0.2	0.2	0.2	0.2	0.2	0.4	0.2	0.4	0.2	0.2	0.4	0.2	0.4	0.2
M00201: alpha-Glucoside_transport_system	0.2	0.2	0.2	0.2	0.8	0.2	0.2	0.6	0.2	0.2	0.2	0.2	0.8	0.8	0.2	0.2	0.8	0.2	0.8	0.2	0.2	0.2	0.2	0.2	0.2	0.2	0.2	0.2
M00202: Oligogalacturonide_transport_system	0	0	0	0	0	0	0	0	0	0	0	0	0	0	0	0	0	0	0	0	0	0	0	0	0	0	0	0
M00204: Trehalose/maltose_transport_system	0	0	0	0	0	0	0	0.25	0	0	0	0	0	0	0	0	0	0	0	0.25	0	0.25	0	0	0.25	0	0.25	0
M00205: N-Acetylglucosamine_transport_system	0	0	0	0	0	0	0	0	0	0	0	0	0	0	0	0	0	0	0	0	0	0	0	0	0	0	0	0
M00206: Cellobiose_transport_system	0.25	0.25	0.25	0.25	1	0.25	1	0.25	0.25	0.25	0.25	0.25	1	1	0.25	0.25	0.25	0.25	1	0.25	0.25	0.25	0.25	0.25	0.25	0.25	0.25	0.25
M00207: Putative_multiple_sugar_transport_system	0.8	0.8	0.8	0.8	0.8	0.8	0.8	1	0.8	0.8	0.8	0.8	0.8	0.8	0.8	0.8	0.8	0.8	0.8	1	0.8	1	0.8	0.8	1	0.8	1	0.8
M00208: Glycine_betaine/proline_transport_system	0	0	1	0.666667	1	1	1	1	1	1	1	1	1	1	0.333333	0	1	1	1	1	1	1	1	1	1	0.333333	0.333333	0.333333
M00209: Osmoprotectant_transport_system	1	1	0.333333	0	1	0.333333	1	0.666667	1	0.666667	0.666667	1	1	1	0.666667	1	1	1	1	1	1	1	1	0.666667	1	0.333333	0.333333	0.666667
M00210: Phospholipid_transport_system	1	1	1	1	0.8	1	0.8	0.8	0.6	0.6	0.6	0.6	0.6	0.6	0.8	0.8	0.8	0.8	0.8	0.8	0.8	0.8	0.8	0.8	0.8	0.8	0.8	0.8
M00211: Putative_ABC_transport_system	1	1	0.5	0.5	0	0	0	0	1	0.5	0.5	0	0.5	0.5	1	0.5	0.5	0.5	0.5	1	0.5	0	0.5	0.5	0.5	1	1	0.5
M00212: Ribose_transport_system	1	0.666667	0.666667	0.666667	1	1	1	1	1	1	1	1	1	1	1	1	1	1	1	1	1	1	1	1	1	1	1	1
M00213: L-Arabinose_transport_system	0	0	0	0	0	0	0	0	0	0	0	0	0	0	0	0	0	0	0	0	0	0	0	0	0	0	0	0
M00214: Methyl-galactoside_transport_system	0	0	0	0	0	0	0	0	0	0	0	0	0	0	0	0	0	0	0	0	0	0	0	0	0	0	0	0
M00215: D-Xylose_transport_system	0	0	0	0	0	0	0	0	0	0	0	0	0	0	0	0	0	1	1	1	1	1	1	1	0.333333	0	0	0
M00216: Multiple_sugar_transport_system	0	0	0	0	1	0.333333	1	1	0.333333	1	0.333333	1	1	1	0	0	1	1	1	0	0	1	1	0	0	0	0	0
M00217: D-Allose_transport_system	0	0	0	0	0	0	0	0	0	0	0	0	0	0	0	0	0	0	0	0	0	0	0	0	0	0	0	0
M00218: Fructose_transport_system	0	0	0	0	0	0	0	0	0	0	0	0	0	0	0	0	0	0	0	0	0	0	0	0	0	0	0	0
M00219: AI-2_transport_system	0	0	0	0	0	0	0	0	0	0	0	0	0	0	0	0	0	0	0	0	0	0	0	0	0	0	0	0
M00220: Rhamnose_transport_system	0	0	0	0	0	0	0	0	0	0	0	0	0	0	0	0	1	0.5	0.5	0.5	0.5	0.25	0.5	0.75	0.25	0	0	0
M00221: Putative_simple_sugar_transport_system	1	1	1	1	1	1	1	1	1	1	1	1	1	1	1	1	1	1	1	1	1	1	1	1	1	1	1	1
M00222: Phosphate_transport_system	1	1	1	1	1	1	1	1	1	1	1	1	1	1	1	1	1	1	1	1	1	1	1	1	1	1	1	1
M00223: Phosphonate_transport_system	1	1	1	1	1	1	1	1	1	1	1	1	1	1	1	1	1	1	1	1	1	1	1	1	1	1	1	1
M00224: Fluoroquinolone_transport_system	0	0	0	0	0	0	0	0	0	0	0	0	0	0	0	0	1	0	0	0	0	0	0	0	0	0	0	0
M00225: Lysine/arginine/ornithine_transport_system	0	0	0	0	0	0	0	0	0	0	0	0	0	0	0	0	0	0	0	0	0	0	0	0	0	0	0	0
M00226: Histidine_transport_system	0	0	0	0	0	0	0	0	0	0	0	0	0	0	0	0	0	0	0	0	0	0	0	0	0	0	0	0
M00227: Glutamine_transport_system	0	0	0	0	0	0	0	0	0	0	0	0	0	0	0	0	0	0	0	0	0	0	0	0	0	0	0	0
M00228: Putative_glutamine_transport_system	0	0	0	0	0	0	0	0	0	0	0	0	0	0	0	0	0	0	0	0	0	0	0	0	0	0	0	0
M00229: Arginine_transport_system	0	0	0	0	0	0	0	0	0	0	0	0	0	0	0	0	0	0	0	0	0	0	0	0	0	0	0	0
M00230: Glutamate/aspartate_transport_system	0.25	1	0.25	0.25	1	1	0.25	0.25	0.25	1	1	0.25	0.25	0.25	1	0.25	0	0.25	0	0	0	1	0.25	0	1	0.25	0.25	0.25
M00231: Octopine/nopaline_transport_system	0	0	0	0	0	0	0	0	0	0	0	0	0	0	0	0	0	0	0	0	0	0	0	0	0	0	0	0
M00232: General_L-amino_acid_transport_system	0	0	0	0	1	0	0	1	0	1	1	0	1	0	0.25	0	1	0	0.25	0	0	0	0	0	1	1	1	1
M00233: Glutamate_transport_system	0	0	0	0	1	0	1	1	0	1	0.25	1	1	1	0	0	1	0.25	1	0	0	1	0	0	0	0	0	0
M00234: Cystine_transport_system	0	0	0	0	0.666667	0	1	0.666667	0	0	0	0	1	1	0	0	0	0	0.333333	0	0	0.333333	0	0	0.333333	0	0	0
M00235: Arginine/ornithine_transport_system	0	0	0	0	0	0	0	0	0	0	0	0	0	0	0	0	0	0	0	0	0	0	0	0	0	0	0	0
M00236: Putative_polar_amino_acid_transport_system	1	1	1	1	1	1	1	1	1	1	1	1	1	1	1	1	1	1	1	1	1	1	1	1	1	1	1	1
M00237: Branched-chain_amino_acid_transport_system	1	1	1	1	1	1	1	1	1	1	1	1	1	1	1	1	1	1	1	1	1	1	1	1	1	1	1	1

KEGG_Module	Habitat																																				
	Marine_sediments							Taiga_upper_active_layer				Taiga_bottom_active_layer				Taiga_permafrost_layer				Oil_sands_core							Oil_sands_tailings_pond_upper				Oil_sands_tailings_pond_median			Oil_sands_tailings_pond_deep			
	DWH1	DWH2	DWH3	DWH4	DWH5	DWH6	DWH7	Tu1	Tu2	Tu3	Tu4	Tb1	Tb2	Tb3	Tb4	Tp1	Tp2	Tp3	Tp4	OSC1	OSC12	OSC3	OSC4	OSC5	OSC7	OSC9	OSTPu1	OSTPu2	OSTPu4	OSTPu3	OSTPm2	OSTPm6	OSTPm4	OSTPd1	OSTPd2	OSTPd3	OSTPd4
M00124: Pyridoxal_biosynthesis,_erythrose-4P_=>_pyridoxal-5P	0.666667	0.666667	0.666667	0.666667	0.666667	0.666667	0.666667	0.666667	0.666667	0.5	0.666667	0.666667	0.666667	0.666667	0.666667	0.666667	0.666667	0.5	0.666667	0.833333	0.833333	0.666667	0.833333	0.833333	0.833333	0.833333	0.666667	0.666667	0.666667	0.666667	0.666667	0.666667	0.666667	0.666667	0.666667	0.833333	0.666667
M00125: Riboflavin_biosynthesis,_GTP_=>_riboflavin/FMN/FAD	0.416667	0.416667	0.416667	0.5	0.5	0.5	0.5	0.5	0.5	0.666667	0.5	0.416667	0.5	0.416667	0.5	0.5	0.5	0.416667	0.5	0.5	0.5	0.666667	0.5	0.5	0.5	0.583333	0.416667	0.5	0.416667	0.416667	0.5	0.5	0.5	0.5	0.5	0.583333	0.416667
M00126: Tetrahydrofolate_biosynthesis,_GTP_=>_THF	0.391304	0.391304	0.391304	0.391304	0.391304	0.391304	0.391304	0.347826	0.347826	0.347826	0.347826	0.347826	0.347826	0.347826	0.347826	0.347826	0.347826	0.347826	0.347826	0.347826	0.347826	0.391304	0.347826	0.347826	0.347826	0.347826	0.347826	0.347826	0.391304	0.391304	0.391304	0.391304	0.391304	0.347826	0.347826	0.391304	0.391304
M00127: Thiamine_biosynthesis,_AIR_=>_thiamine-P/thiamine-2P	0.4	0.4	0.6	0.4	0.4	0.4	0.4	0.4	0.4	0.5	0.4	0.4	0.4	0.4	0.4	0.4	0.4	0.4	0.4	0.4	0.4	0.4	0.4	0.4	0.4	0.4	0.4	0.4	0.6	0.5	0.5	0.5	0.4	0.4	0.4	0.4	0.5
M00128: Ubiquinone_biosynthesis,_eukaryotes,_4-hydroxybenzoate_=>_ubiquinone	0.166667	0.166667	0	0.166667	0.166667	0	0.166667	0.166667	0.166667	0	0.166667	0.166667	0.166667	0.166667	0.166667	0.166667	0.166667	0	0.166667	0.166667	0.166667	0.166667	0.166667	0.166667	0.166667	0.166667	0.166667	0.166667	0	0.166667	0.166667	0	0.166667	0.166667	0.166667	0.166667	0.166667
M00129: Ascorbate_biosynthesis,_animals,_glucose-1P_=>_ascorbate	0.428571	0.428571	0.428571	0.428571	0.428571	0.428571	0.428571	0.428571	0.571429	0.571429	0.428571	0.571429	0.428571	0.571429	0.571429	0.428571	0.428571	0.285714	0.571429	0.428571	0.428571	0.428571	0.428571	0.428571	0.428571	0.428571	0.428571	0.428571	0.285714	0.428571	0.428571	0.285714	0.428571	0.428571	0.428571	0.428571	0.428571
M00130: Inositol_phosphate_metabolism,_PI=>_PIP2_=>_Ins(1,4,5)P3_=>_Ins(1,3,4,5)P4	0	0	0	0	0	0	0	0	0	0	0	0	0	0	0	0	0	0	0	0	0	0	0	0	0	0	0	0	0	0	0	0	0	0	0	0	0
M00131: Inositol_phosphate_metabolism,_Ins(1,3,4,5)P4_=>_Ins(1,3,4)P3_=>_myo-inositol	0.125	0.125	0.125	0.125	0.125	0.125	0.125	0.125	0.125	0.125	0.125	0.125	0.125	0.125	0.125	0.125	0.125	0.125	0.125	0.125	0.125	0.125	0.125	0.125	0.125	0.125	0.125	0.125	0.125	0.125	0.125	0.125	0.125	0.125	0.125	0.125	0.125
M00133: Polyamine_biosynthesis,_arginine_=>_agmatine_=>_putrescine_=>_spermidine	0.571429	0.571429	0.571429	0.571429	0.571429	0.571429	0.571429	0.428571	0	0.285714	0.142857	0.285714	0.142857	0.428571	0	0.285714	0.285714	0.428571	0.428571	0.285714	0.571429	0.571429	0.285714	0.285714	0.428571	0.571429	0.428571	0.428571	0.714286	0.571429	0.714286	0.714286	0.428571	0.428571	0.428571	0.571429	0.571429
M00134: Polyamine_biosynthesis,_arginine_=>_ornithine_=>_putrescine	0.5	0	0	0.5	0.5	0.5	1	1	0.5	0.5	0.5	1	1	1	0.5	1	1	0.5	1	1	1	1	1	1	1	1	0.5	1	0.5	0.5	0.5	1	1	1	1	1	1
M00135: GABA_biosynthesis,_eukaryotes,_putrescine_=>_GABA	0.2	0.2	0.2	0.2	0.2	0.2	0.2	0.4	0.4	0.4	0.4	0.4	0.4	0.4	0.4	0.4	0.4	0.4	0.4	0.4	0.4	0.4	0.4	0.4	0.4	0.4	0.2	0.2	0.2	0.2	0.4	0.4	0.2	0.4	0.2	0.4	0.4
M00136: GABA_biosynthesis,_prokaryotes,_putrescine_=>_GABA	0.25	0.5	0.5	0.5	0.5	0.5	0.5	0.5	0.5	0	0.25	0.5	0	0.25	0.5	0.25	0	0	0.5	0.25	0.25	0.5	0.25	0.25	0.25	0.25	0	0	0	0	0	0	0	0	0	0.25	0
M00137: Flavanone_biosynthesis,_phenylalanine_=>_naringenin	0	0	0	0	0	0	0	0	0	0	0	0	0	0	0	0	0	0	0	0	0	0	0	0	0	0	0	0	0	0	0	0	0	0	0	0	0
M00140: C1-unit_interconversion,_prokaryotes	1	1	1	1	1	1	1	1	0.666667	1	1	0.666667	0.666667	1	0.666667	1	0.666667	1	0.666667	0.666667	0.666667	0.666667	0.666667	0.666667	0.666667	0.666667	0.666667	0.666667	1	1	1	1	1	0.666667	0.666667	0.666667	1
M00143: NADH_dehydrogenase_(ubiquinone)_Fe-S_protein/flavoprotein_complex,_mitochondria	0	0	0	0	0	0	0	0	0	0	0	0	0	0	0	0	0	0	0	0	0	0	0	0	0	0	0	0	0	0	0	0	0	0	0	0	0
M00144: NADH:quinone_oxidoreductase,_prokaryotes	0.777778	0.777778	0.777778	0.777778	0.777778	0.777778	0.777778	0.777778	0.777778	0.777778	0.777778	0.777778	0.777778	0.777778	0.777778	0.777778	0.777778	0.777778	0.777778	0.833333	0.833333	0.777778	0.833333	0.833333	0.833333	0.833333	0.777778	0.777778	0.777778	0.777778	0.777778	0.777778	0.777778	0.777778	0.777778	0.833333	0.833333
M00145: NAD(P)H:quinone_oxidoreductase,_chloroplasts_and_cyanobacteria	0	0	0	0	0	0	0	0	0	0	0	0	0	0	0	0	0	0	0	0	0	0	0	0	0	0	0	0	0	0	0	0	0	0	0	0	0
M00146: NADH_dehydrogenase_(ubiquinone)_1_alpha_subcomplex	0	0	0	0	0	0	0	0	0	0	0	0	0	0	0	0	0	0	0	0	0	0	0	0	0	0	0	0	0	0	0	0	0	0	0	0	0
M00149: Succinate_dehydrogenase,_prokaryotes	1	1	1	1	1	1	1	1	1	1	1	1	1	1	1	1	1	1	1	1	1	1	1	1	1	1	1	1	1	1	1	1	1	1	1	1	1
M00150: Fumarate_reductase,_prokaryotes	0	0	0	0	0	0	0	0	0	0	0	0	0	0	0	0	0	0	0	0	0	0	0	0	0	0	0	0	0.25	0.25	0	0	0	0	0	0	0
M00151: Cytochrome_bc1_complex_respiratory_unit	0.3	0.3	0.3	0.3	0.3	0.3	0.3	0.3	0.3	0.4	0.3	0.3	0.3	0.2	0.3	0.3	0.3	0.4	0.3	0.3	0.3	0.3	0.3	0.3	0.3	0.3	0.3	0.3	0.2	0.3	0.3	0.2	0.3	0.3	0.3	0.3	0.3
M00153: Cytochrome_d_ubiquinol_oxidase	1	1	1	1	1	1	1	1	1	1	1	1	1	1	1	1	1	1	1	1	1	1	1	1	1	1	1	1	1	1	1	1	1	1	0	1	1
M00154: Cytochrome_c_oxidase	0.111111	0.111111	0.111111	0.111111	0.111111	0.111111	0.111111	0.111111	0.111111	0.055556	0.111111	0.111111	0.111111	0.111111	0.111111	0.111111	0.111111	0.055556	0.111111	0.111111	0.111111	0.111111	0.111111	0.111111	0.111111	0.111111	0.111111	0.111111	0.055556	0.111111	0.111111	0.055556	0.111111	0.111111	0.111111	0.111111	0.055556
M00155: Cytochrome_c_oxidase,_prokaryotes	0.6	0.6	0.6	0.6	0.6	0.6	0.6	0.6	0.6	0.6	0.6	0.6	0.6	0.8	0.6	0.6	0.6	0.8	0.6	0.6	0.6	0.6	0.6	0.6	0.6	0.6	0.6	0.6	0.6	0.6	0.6	0.8	0.6	0.6	0.6	0.6	0.6
M00156: Cytochrome_c_oxidase,_cbb3-type	1	1	1	1	0.8	1	1	0.8	0.8	0	0.8	0.8	0.8	0.8	0.8	0.8	0.8	0.4	0.8	0.8	0.8	0.8	0.8	0.8	0.8	0.8	0.8	0.8	0.4	0.8	0.8	0.8	0.8	0.8	0.8	0.8	0.8
M00157: F-type_ATPase,_prokaryotes_and_chloroplasts	1	1	1	1	1	1	1	1	1	1	1	1	1	1	1	1	1	1	1	1	1	1	1	1	1	1	1	1	1	1	1	1	1	1	1	1	1
M00159: V-type_ATPase,_prokaryotes	0	0	0	0	0	0	0	0	0	0	0	0	0	0	0	0	0	0	0	0	0	0	0	0	0	0	0	0	0.777778	0.555556	0	0.666667	0	0	0	0	0.666667
M00160: V-type_ATPase,_eukaryotes	0	0	0	0	0	0	0	0	0	0	0	0	0	0	0	0	0	0	0	0	0	0	0	0	0	0	0	0	0	0	0	0	0	0	0	0	0
M00161: Photosystem_II	0	0	0	0	0	0	0	0	0	0	0	0	0	0	0	0	0	0	0	0	0	0	0	0	0	0	0	0	0	0	0	0	0	0	0	0	0
M00162: Cytochrome_b6f_complex	0	0	0	0	0	0	0	0	0	0	0	0	0	0	0	0	0	0	0	0	0	0	0	0	0	0	0	0	0	0	0	0	0	0	0	0	0
M00163: Photosystem_I	0	0	0	0	0	0	0	0	0	0	0	0	0	0	0	0	0	0	0	0	0	0	0	0	0	0	0	0	0	0	0	0	0	0	0	0	0
M00165: Reductive_pentose_phosphate_cycle_(Calvin_cycle)	0.391304	0.347826	0.304348	0.347826	0.304348	0.347826	0.347826	0.434783	0.391304	0.304348	0.434783	0.434783	0.434783	0.391304	0.391304	0.434783	0.478261	0.304348	0.391304	0.347826	0.347826	0.304348	0.347826	0.347826	0.347826	0.391304	0.391304	0.347826	0.391304	0.478261	0.391304	0.347826	0.434783	0.347826	0.26087	0.391304	0.304348
M00166: Reductive_pentose_phosphate_cycle,_ribulose-5P_=>_glyceraldehyde-3P	0.428571	0.285714	0.285714	0.285714	0.285714	0.285714	0.285714	0.428571	0.285714	0.285714	0.428571	0.428571	0.428571	0.285714	0.285714	0.428571	0.571429	0.285714	0.285714	0.428571	0.285714	0.285714	0.285714	0.285714	0.285714	0.285714	0.571429	0.571429	0.428571	0.714286	0.571429	0.285714	0.714286	0.571429	0.285714	0.571429	0.285714
M00167: Reductive_pentose_phosphate_cycle,_glyceraldehyde-3P_=>_ribulose-5P	0.375	0.375	0.3125	0.375	0.3125	0.375	0.375	0.4375	0.4375	0.3125	0.4375	0.4375	0.4375	0.4375	0.4375	0.4375	0.4375	0.3125	0.4375	0.375	0.375	0.25	0.375	0.375	0.375	0.4375	0.3125	0.25	0.375	0.375	0.3125	0.375	0.3125	0.25	0.25	0.3125	0.3125
M00168: CAM_(Crassulacean_acid_metabolism),_dark	0.5	0.5	0.5	0.5	0.5	0.5	0.5	0.5	0.5	0.5	0.5	0.5	0.5	0.5	0.5	0.5	0.5	0.5	0.5	0.5	0.5	0.5	0.5	0.5	0.5	0.5	0.5	0.5	0.5	0.5	0.5	0.5	0.5	0.5	0.5	0.5	0.5
M00169: CAM_(Crassulacean_acid_metabolism),_light	1	1	1	1	1	1	1	1	1	0.5	1	1	1	1	1	1	1	0.5	1	1	1	1	1	1	1	1	1	0.5	1	1	1	1	1	0.5	0.5	1	1
M00170: C4-dicarboxylic_acid_cycle,_phosphoenolpyruvate_carboxykinase_type	0.25	0.5	0.5	0.5	0.5	0.5	0.5	0.5	0.5	0.25	0.5	0.5	0.5	0.25	0.5	0.5	0.5	0.25	0.5	0.25	0.25	0.25	0.25	0.25	0.25	0.25	0.25	0.25	0.25	0.25	0.25	0.5	0.25	0.25	0.25	0.25	0.25
M00172: C4-dicarboxylic_acid_cycle,_NADP_-_malic_enzyme_type	0.75	0.75	0.75	0.75	0.75	0.75	0.75	0.75	0.75	0.5	0.75	0.75	0.75	0.75	0.75	0.75	0.75	0.5	0.75	0.75	0.75	0.75	0.75	0.75	0.75	0.75	0.75	0.5	0.75	0.75	0.75	0.75	0.75	0.5	0.5	0.75	0.75
M00173: Reductive_citrate_cycle_(Arnon-Buchanan_cycle)	0.511628	0.44186	0.534884	0.465116	0.465116	0.372093	0.465116	0.395349	0.348837	0.395349	0.325581	0.395349	0.395349	0.348837	0.372093	0.395349	0.395349	0.372093	0.372093	0.372093	0.372093	0.372093	0.372093	0.372093	0.372093	0.372093	0.44186	0.372093	0.651163	0.534884	0.534884	0.604651	0.44186	0.372093	0.325581	0.44186	0.627907
M00174: Methane_oxidation,_methanotroph,_methane_=>_formaldehyde	0	0	0	0	0	0	0	0	0	0	0	0	0	0	0	0	0	0	0	0	0	0	0	0	0	0	0	0	0	0	0	0	0.083333	0	0	0	0
M00175: Nitrogen_fixation,_nitrogen_=>_ammonia	0	0	0.75	0	0	0	0	0.25	0	0	0.75	0.75	0.25	0	0	0	0	0	0	0	0	0	0	0	0	0	0.75	0.75	0.75	0.75	0.75	0.75	0	0.75	0	0.75	0.75
M00176: Assimilatory_sulfate_reduction,_sulfate_=>_H2S	0.583333	0.583333	0.583333	0.666667	0.666667	0.666667	0.666667	0.416667	0.416667	0.583333	0.416667	0.416667	0.5	0.666667	0.416667	0.583333	0.5	0.583333	0.5	0.5	0.5	0.583333	0.5	0.5	0.5	0.583333	0.333333	0.416667	0.333333	0.5	0.5	0.416667	0.416667	0.416667	0.416667	0.583333	0.5
M00177: Ribosome,_eukaryotes	0	0	0	0	0	0	0	0	0	0	0	0	0	0	0	0	0	0	0	0	0	0	0	0	0	0	0.012658	0	0	0	0	0	0.012658	0.012658	0.012658	0.012658	0
M00178: Ribosome,_bacteria	0.981818	0.981818	0.981818	0.981818	0.981818	0.981818	0.981818	0.981818	0.981818	0.981818	0.981818	0.981818	0.981818	0.981818	0.981818	0.981818	0.981818	0.981818	0.981818	0.981818	0.981818	0.981818	0.981818	0.981818	0.981818	0.981818	0.981818	0.981818	0.981818	0.981818	0.981818	0.981818	0.981818	0.981818	0.981818	0.981818	0.981818
M00183: RNA_polymerase,_bacteria	0.666667	0.666667	0.666667	0.666667	0.666667	0.666667	0.666667	0.666667	0.666667	0.666667	0.666667	0.666667	0.666667	0.666667	0.666667	0.666667	0.666667	0.666667	0.666667	0.666667	0.666667	0.666667	0.666667	0.666667	0.666667	0.666667	0.666667	0.666667	0.666667	0.666667	0.666667	0.666667	0.666667	0.666667	0.666667	0.666667	0.666667
M00185: Sulfate_transport_system	1	0.75	0.75	1	0.75	1	1	1	1	1	1	1	1	1	1	1	1	0.5	1	1	1	1	1	1	1	1	1	1	0.25	1	1	0.75	1	1	1	1	1
M00186: Tungstate_transport_system	0	0	0	0	0	0	0	0	0	0.666667	0	0	0	0.666667	0	0	0	0.666667	0	0	0	1	0	0	0	0.333333	1	1	1	1	1	1	1	1	1	1	1
M00188: NitT/TauT_family_transport_system	1	1	1	1	1	1	1	1	1	1	1	1	1	1	1	1	1	1	1	1	1	1	1	1	1	1	1	1	1	1	1	1	1	1	1	1	1
M00189: Molybdate_transport_system	0.75	0.75	0.75	0.75	0.75	0.75	0.75	0.75	0.75	0.75	0.75	0.75	0.75	0.75	0.75	0.75	0.75	0.75	0.75	0.75	0.75	0.75	0.75	0.75	0.75	0.75	0.75	0.75	0.75	0.75	0.75	0.75	0.75	0.75	0.75	0.75	0.75
M00190: Iron(III)_transport_system	1	1	1	1	1	1	1	1	0	1	1	0.666667	1	1	0	1	1	1	1	1	1	1	1	1	1	1	1	1	1	1	1	1	1	1	1	1	1
M00191: Thiamine_transport_system	0	0	0	0	0	0.333333	0	0	0	0	0	0	0	0	0	0	0	0	0	1	0	0	0	0	1	0	0	0	0	0	0	0	0	0	0	0	0
M00192: Putative_thiamine_transport_system	0	0	0	0	0	0	0	0	0	0	0	0	0	0	0	0	0	0	0	0	0	0	0	0	0	0	0	0	0	0	0	0	0	0	0	0	0
M00193: Putative_spermidine/putrescine_transport_system	0.75	1	0.75	1	1	1	1	1	0.5	1	1	0.75	1	1	1	1	1	1	1	1	1	1	1	1	1	1	0.75	0.25	1	1	1	1	1	0.25	0.25	1	1
M00194: Maltose/maltodextrin_transport_system	0.2	0.4	0.2	0.2	0.2	0.4	0.4	0.4	0.2	0.2	0	0.4	0.2	0	0	0.4	0.4	0	0	0.4	0.4	0.4	0.4	0.4	0.4	0.4	0.2	0	0.2	0.2	0.2	0.2	0.2	0	0	0.4	0.2
M00196: Multiple_sugar_transport_system	1	0.25	0.25	0.25	0.25	0.25	0.25	1	0	1	0.25	0.25	0.25	1	0.25	1	0.25	1	0	1	0.5	1	1	0.5	1	1	0.25	0	1	0.5	0.25	1	0.25	0	0	0.25	1
M00197: Putative_fructooligosaccharide_transport_system	0	0.25	0.25	0.25	0.25	0	0.25	0	0	0	0	0	0	0	0	0	0	0	0	0	0	0.25	0	0	0.25	0.25	0	0	0.25	0	0	0	0	0	0	0	0
M00198: Putative_sn-glycerol-phosphate_transport_system	0	0	0	0	0	0.5	0	1	0	0.75	0	0.75	0.75	0.75	0	1	1	0.75	1	1	1	1	1	1	1	1	1	1	1	1	1	1	0.75	1	1	1	0.75
M00199: L-Arabinose/lactose_transport_system	0	0	0	0	0	0	0	0	0	0	0	0	0	0	0	0	0	0	0	0	0	0	0	0	0	0	0	0	0	0	0	0	0	0	0	0	0
M00200: Putative_sorbitol/mannitol_transport_system	0.2	0.4	0.2	0.2	0.2	0.4	0.4	0.4	0	0.8	0.4	0.4	0.2	0.2	0.4	0.4	0.4	0.2	0.2	0.4	0.4	0.4	0.4	0.4	0.4	0.4	0.2	0	0.2	0.2	0.2	0.2	0.2	0	0	0.4	0.2
M00201: alpha-Glucoside_transport_system	0.2	0.2	0.2	0.2	0.2	0.2	0.2	0.2	0	0.8	0.2	0.2	0.2	0.2	0.2	0.2	0.2	0.6	0	0.2	0.2	0.2	0.2	0.2	0.2	0.2	0.2	0	0.2	0.2	0.2	0.2	0.2	0	0	0.2	0.2
M00202: Oligogalacturonide_transport_system	0	0	0	0	0	0	0	0	0	0	0	0.75	0	0	0.75	0	0	0	0.75	0	0	0	0	0	0	0	0	0	0	0	0	0	0	0	0	0	0
M00204: Trehalose/maltose_transport_system	0	0.25	0	0	0	0.25	0.25	0.25	0.25	0	0.25	0.25	0	0	0.25	0.25	0.25	0.75	0.25	0.25	0.25	0.25	0.25	0.25	0.25	0.25	0	0	0	0	0	0	0	0	0	0.25	0
M00205: N-Acetylglucosamine_transport_system	0	0	0	0	0	0	0	0	0	1	0	0	0	0	0	0	0	0	0	0	0	0	0	0	0	0	0	0	0	0	0	0	0	0	0	0	0
M00206: Cellobiose_transport_system	0.25	0	0.25	0.25	0.25	0.25	0.25	0.25	0	1	0.25	0.25	0.25	0.25	0.25	0.25	0.25	0.5	0	0.25	0.25	0.25	0.25	0.25	0.25	0.25	0	0	0.25	0.25	0.25	0.25	0.25	0	0	0	0.25
M00207: Putative_multiple_sugar_transport_system	0.8	1	0.8	0.8	0.8	1	1	1	0.2	0.8	1	1	0.8	0.8	0.4	1	1	0.8	0.8	1	1	1	1	1	1	1	0.8	0	0.8	0.8	0.8	0.8	0.8	0.6	0	1	0.8
M00208: Glycine_betaine/proline_transport_system	0.333333	1	0.333333	1	1	1	1	1	0	1	0.666667	0	0	0	0	0.333333	0	0.333333	0.333333	1	1	1	1	1	1	1	1	1	0.333333	0	0.333333	0.333333	1	1	1	1	0.333333
M00209: Osmoprotectant_transport_system	0.333333	0	0.333333	0	0	0	0	0.666667	0.666667	1	0.666667	0.666667	0.666667	1	0.666667	1	0.666667	1	0.666667	0.666667	0.666667	0.666667	0.666667	0.666667	1	1	0	0	0.666667	0.333333	0	0.333333	0	0	0	0.333333	0.333333
M00210: Phospholipid_transport_system	1	1	1	1	1	0.8	1	0.8	0.8	0.6	0.8	0.8	0.8	0.8	0.8	0.8	1	0.6	0.8	1	1	1	0.8	1	1	1	1	1	0.8	1	1	0.8	1	1	1	1	0.8
M00211: Putative_ABC_transport_system	0	0	0.5	0	0	0	0	0	0	0.5	0	0	0	0	0	0	0	0.5	0	0	0	0.5	0	0	0	0	0.5	0.5	0.5	0	0.5	0.5	0	0.5	0	0	0.5
M00212: Ribose_transport_system	1	1	1	1	1	1	1	1	0.333333	1	1	1	1	1	0.333333	1	1	1	0.666667	1	1	1	1	1	1	1	1	0	1	1	1	1	1	0	0	1	1
M00213: L-Arabinose_transport_system	0	0	0	0	0	0	0	0	0	0	0	0	0	0	0	0	0	0	0	0	0	0	0	0	0	0	0	0	0	0	0	0	0	0	0	0	0
M00214: Methyl-galactoside_transport_system	0	0	0	0	0	0	0	0	0	0	0	0	0	0	0	0	0	0	0	0	0	0	0	0	0	0	0	0	0	0	0	0	0	0	0	0	0
M00215: D-Xylose_transport_system	0	0	0	0	0	1	0	0	0	1	0	0	0	0	0	0	0	1	0	1	0	1	0	1	1	1	0	0	0	0	0	0	0	0	0	0	0
M00216: Multiple_sugar_transport_system	0	0	0	0	0	0	0	0	0	1	0	0	0	0	0	0	0	0	0	0.333333	0	0	0.333333	0.333333	0.333333	1	0	0	0	0	0	0	0	0	0	0	0
M00217: D-Allose_transport_system	0	0	0	0	0	0	0	0	0	0	0	0	0	0	0	0	0	0	0	0	0	0	0	0	0	0	0	0	0	0	0	0	0	0	0	0	0
M00218: Fructose_transport_system	0	0	0	0	0	0	0	0	0	0	0	0	0	0	0	0	0	0	0	0	0	0	0	0	0	0	0	0	0	0	0	0	0	0	0	0	0
M00219: AI-2_transport_system	0	0	0	0	0	0	0	0	0	0	0	0	0	0	0	0	0	0	0	0	0	0	0	0	0	0	0	0	0	0	0	0	0	0	0	0	0
M00220: Rhamnose_transport_system	0	0	0	0	0	0.5	0	0	0	0.25	0	0	0	0	0	0	0	0.25	0	0	0	0	0	0	0	0	0	0	0	0	0	0	0	0	0	0	0
M00221: Putative_simple_sugar_transport_system	1	1	1	1	1	1	1	1	0.333333	1	1	0.666667	1	1	0.333333	1	1	1	1	1	1	1	1	1	1	1	1	1	1	1	1	1	1	1	1	1	1
M00222: Phosphate_transport_system	1	1	1	1	1	1	1	1	1	1	1	1	1	1	1	1	1	1	1	1	1	1	1	1	1	1	1	1	1	1	1	1	1	1	1	1	1
M00223: Phosphonate_transport_system	1	1	1	1	1	1	1	1	1	0.333333	1	1	1	1	1	1	1	0	1	1	1	1	1	1	1	1	1	1	1	1	1	1	1	1	1	1	1
M00224: Fluoroquinolone_transport_system	0	0	0	0	0	0	0	0	0	0	0	0	0	0	0	0	0	0	0	0	0	0	0	0	0	0	0	0	0	0	0	0	0	0	0	0	0
M00225: Lysine/arginine/ornithine_transport_system	0	0	0	0	0	0	0	0.25	0.25	0	0	0.25	0	0	0.25	0	0	0	0.25	0	0	0	0	0	0	0	0	0	0	0	0	0	0	0	0	0	0
M00226: Histidine_transport_system	0	0	0	0	0	0	0	0	0	0	0	0	0	0	0	0	0	0	0	0	0	0	0	0	0	0	0	0	0	0	0	0	0	0	0	0	0
M00227: Glutamine_transport_system	0	0	0	0	0	0	0	0	0	0	0	0	0	0	0	0	0	0	0	0	0	0	0	0	0	0	0	0	0	0	0	0	0	0	0	0	0
M00228: Putative_glutamine_transport_system	0	0	0	0	0	0	0	0	0	0	0	0	0	0	0	0	0	0	0	0	0	0	0	0	0	0	0	0	0	0	0	0	0	0	0	0	0
M00229: Arginine_transport_system	0	0	0	0	0	0	0	0	0	0	0	0	0	0	0	0	0	0	0	0	0	0	0	0	0	0	0	0	0	0	0	0	0	0	0	0	0
M00230: Glutamate/aspartate_transport_system	0.25	0.25	1	1	1	0	0.25	0.25	0	0	0	0	0.25	0	0	0.25	0.25	0	0	1	1	1	1	1	1	1	1	1	1	1	1	1	1	1	1	1	1
M00231: Octopine/nopaline_transport_system	0	0	0	0	0	0	0	0	0	0	0	0	0	0	0	0	0	0	0	0	0	0	0	0	0	0	0	0	0	0	0	0	0	0	0	0	0
M00232: General_L-amino_acid_transport_system	1	1	1	1	1	1	1	1	0.25	0	1	0.5	0.25	0.25	0.25	0.25	0.25	0	0.5	1	1	0.5	1	1	1	1	0	0	0	0	0	1	0	0	0	1	1
M00233: Glutamate_transport_system	0	0	0	0	0	0	0	0	0	1	0	0	0	0	0	0	0	1	0	0	0	0	0	0	0	0	0	0	0	0	0	0	0	0	0	0	0
M00234: Cystine_transport_system	0	0	0	0	0	0	0	0	0	1	0	0	0	0	0	0	0	0	0	0.666667	0.666667	0.666667	0.666667	0.666667	0.666667	0.666667	0	0	0	0	0	0	0	0	0	0.333333	0
M00235: Arginine/ornithine_transport_system	0	0	0	0	0	0	0	0	0	0	0	0	0	0	0	0	0	0	0	0	0	0	0	0	0	0	0	0	0	0	0	0	0	0	0	0	0
M00236: Putative_polar_amino_acid_transport_system	1	1	1	1	1	1	1	1	0.666667	1	1	1	1	1	0.666667	1	1	1	1	1	1	1	1	1	1	1	1	1	1	1	1	1	1	1	1	1	1
M00237: Branched-chain_amino_acid_transport_system	1	1	1	1	1	1	1	1	1	1	1	1	1	1	1	1	1	1	1	1	1	1	1	1	1	1	1	1	1	1	1	1	1	1	1	1	1

KEGG_Module	1 if median >= 90% for the samples per site; -1 if median <= 10% for the samples per site												Kind of Module inferred (Core or Varied)
	India_oil_refineries	Arctic	China_oil_refineries	Mangrove	Marine_sediments	Taiga_upper_active_layer	Taiga_bottom_active_layer	Taiga_permafrost_layer	Oil_sands_core	Oil_sands_tailings_pond_upper	Oil_sands_tailings_pond_median	Oil_sands_tailings_pond_deep	
M00124: Pyridoxal_biosynthesis,_erythrose-4P_=>_pyridoxal-5P	0	0	0	0	0	0	0	0	0	0	0	0	
M00125: Riboflavin_biosynthesis,_GTP_=>_riboflavin/FMN/FAD	0	0	0	0	0	0	0	0	0	0	0	0	
M00126: Tetrahydrofolate_biosynthesis,_GTP_=>_THF	0	0	0	0	0	0	0	0	0	0	0	0	
M00127: Thiamine_biosynthesis,_AIR_=>_thiamine-P/thiamine-2P	0	0	0	0	0	0	0	0	0	0	0	0	
M00128: Ubiquinone_biosynthesis,_eukaryotes,_4-hydroxybenzoate_=>_ubiquinone	0	-1	-1	0	0	0	0	0	0	0	0	0	
M00129: Ascorbate_biosynthesis,_animals,_glucose-1P_=>_ascorbate	0	0	0	0	0	0	0	0	0	0	0	0	
M00130: Inositol_phosphate_metabolism,_PI=>_PIP2_=>_Ins(1,4,5)P3_=>_Ins(1,3,4,5)P4	-1	-1	-1	-1	-1	-1	-1	-1	-1	-1	-1	-1	
M00131: Inositol_phosphate_metabolism,_Ins(1,3,4,5)P4_=>_Ins(1,3,4)P3_=>_myo-inositol	0	0	0	0	0	0	0	0	0	0	0	0	
M00133: Polyamine_biosynthesis,_arginine_=>_agmatine_=>_putrescine_=>_spermidine	0	0	0	0	0	0	0	0	0	0	0	0	
M00134: Polyamine_biosynthesis,_arginine_=>_ornithine_=>_putrescine	1	0	0	0	0	0	1	1	1	0	1	1	
M00135: GABA_biosynthesis,_eukaryotes,_putrescine_=>_GABA	0	0	0	0	0	0	0	0	0	0	0	0	
M00136: GABA_biosynthesis,_prokaryotes,_putrescine_=>_GABA	-1	-1	-1	-1	0	0	0	0	0	-1	-1	-1	
M00137: Flavanone_biosynthesis,_phenylalanine_=>_naringenin	-1	-1	-1	-1	-1	-1	-1	-1	-1	-1	-1	-1	
M00140: C1-unit_interconversion,_prokaryotes	0	1	1	1	1	1	0	0	0	0	1	0	
M00143: NADH_dehydrogenase_(ubiquinone)_Fe-S_protein/flavoprotein_complex,_mitochondria	-1	-1	-1	-1	-1	-1	-1	-1	-1	-1	-1	-1	
M00144: NADH:quinone_oxidoreductase,_prokaryotes	0	0	0	0	0	0	0	0	0	0	0	0	
M00145: NAD(P)H:quinone_oxidoreductase,_chloroplasts_and_cyanobacteria	-1	-1	-1	-1	-1	-1	-1	-1	-1	-1	-1	-1	
M00146: NADH_dehydrogenase_(ubiquinone)_1_alpha_subcomplex	-1	-1	-1	-1	-1	-1	-1	-1	-1	-1	-1	-1	
M00149: Succinate_dehydrogenase,_prokaryotes	1	1	1	1	1	1	1	1	1	1	1	1	core
M00150: Fumarate_reductase,_prokaryotes	-1	-1	-1	0	-1	-1	-1	-1	-1	0	-1	-1	
M00151: Cytochrome_bc1_complex_respiratory_unit	0	0	0	0	0	0	0	0	0	0	0	0	
M00153: Cytochrome_d_ubiquinol_oxidase	1	1	1	1	1	1	1	1	1	1	1	1	core
M00154: Cytochrome_c_oxidase	0	-1	-1	0	0	0	0	0	0	0	0	0	
M00155: Cytochrome_c_oxidase,_prokaryotes	0	0	0	0	0	0	0	0	0	0	0	0	
M00156: Cytochrome_c_oxidase,_cbb3-type	0	0	0	1	1	0	0	0	0	0	0	0	
M00157: F-type_ATPase,_prokaryotes_and_chloroplasts	1	1	1	1	1	1	1	1	1	1	1	1	core
M00159: V-type_ATPase,_prokaryotes	-1	-1	-1	0	-1	-1	-1	-1	-1	0	-1	-1	
M00160: V-type_ATPase,_eukaryotes	-1	-1	-1	-1	-1	-1	-1	-1	-1	-1	-1	-1	
M00161: Photosystem_II	-1	-1	-1	-1	-1	-1	-1	-1	-1	-1	-1	-1	
M00162: Cytochrome_b6f_complex	-1	-1	0	-1	-1	-1	-1	-1	-1	-1	-1	-1	
M00163: Photosystem_I	-1	-1	-1	-1	-1	-1	-1	-1	-1	-1	-1	-1	
M00165: Reductive_pentose_phosphate_cycle_(Calvin_cycle)	0	0	0	0	0	0	0	0	0	0	0	0	
M00166: Reductive_pentose_phosphate_cycle,_ribulose-5P_=>_glyceraldehyde-3P	0	0	0	0	0	0	0	0	0	0	0	0	
M00167: Reductive_pentose_phosphate_cycle,_glyceraldehyde-3P_=>_ribulose-5P	0	0	0	0	0	0	0	0	0	0	0	0	
M00168: CAM_(Crassulacean_acid_metabolism),_dark	0	0	0	0	0	0	0	0	0	0	0	0	
M00169: CAM_(Crassulacean_acid_metabolism),_light	1	1	1	1	1	1	1	1	1	1	1	0	
M00170: C4-dicarboxylic_acid_cycle,_phosphoenolpyruvate_carboxykinase_type	0	0	0	0	0	0	0	0	0	0	0	0	
M00172: C4-dicarboxylic_acid_cycle,_NADP_-_malic_enzyme_type	0	0	0	0	0	0	0	0	0	0	0	0	
M00173: Reductive_citrate_cycle_(Arnon-Buchanan_cycle)	0	0	0	0	0	0	0	0	0	0	0	0	
M00174: Methane_oxidation,_methanotroph,_methane_=>_formaldehyde	-1	-1	-1	-1	-1	-1	-1	-1	-1	-1	-1	-1	
M00175: Nitrogen_fixation,_nitrogen_=>_ammonia	-1	-1	0	0	-1	0	0	-1	-1	0	0	0	
M00176: Assimilatory_sulfate_reduction,_sulfate_=>_H2S	0	0	0	0	0	0	0	0	0	0	0	0	
M00177: Ribosome,_eukaryotes	-1	-1	-1	-1	-1	-1	-1	-1	-1	-1	-1	-1	
M00178: Ribosome,_bacteria	1	1	1	1	1	1	1	1	1	1	1	1	core
M00183: RNA_polymerase,_bacteria	0	0	0	0	0	0	0	0	0	0	0	0	
M00185: Sulfate_transport_system	1	0	1	1	1	1	1	1	1	1	1	1	
M00186: Tungstate_transport_system	1	-1	1	-1	-1	-1	-1	-1	-1	1	1	1	varied
M00188: NitT/TauT_family_transport_system	1	1	1	1	1	1	1	1	1	1	1	1	core
M00189: Molybdate_transport_system	0	0	0	0	0	0	0	0	0	0	0	0	
M00190: Iron(III)_transport_system	1	1	1	1	1	1	0	1	1	1	1	1	
M00191: Thiamine_transport_system	-1	-1	-1	-1	-1	-1	-1	-1	-1	-1	-1	-1	
M00192: Putative_thiamine_transport_system	-1	-1	-1	-1	-1	-1	-1	-1	-1	-1	-1	-1	
M00193: Putative_spermidine/putrescine_transport_system	0	1	1	1	1	1	0	1	1	0	1	0	
M00194: Maltose/maltodextrin_transport_system	0	0	0	0	0	0	0	0	0	0	0	0	
M00196: Multiple_sugar_transport_system	0	1	1	1	0	0	0	0	1	0	0	0	
M00197: Putative_fructooligosaccharide_transport_system	0	-1	0	0	0	-1	-1	-1	-1	-1	-1	-1	
M00198: Putative_sn-glycerol-phosphate_transport_system	0	0	0	0	-1	0	0	1	1	1	1	1	varied
M00199: L-Arabinose/lactose_transport_system	-1	-1	-1	-1	-1	-1	-1	-1	-1	-1	-1	-1	
M00200: Putative_sorbitol/mannitol_transport_system	0	0	0	0	0	0	0	0	0	0	0	0	
M00201: alpha-Glucoside_transport_system	0	0	0	0	0	0	0	0	0	0	0	0	
M00202: Oligogalacturonide_transport_system	-1	-1	-1	-1	-1	-1	0	-1	-1	-1	-1	-1	
M00204: Trehalose/maltose_transport_system	-1	-1	-1	-1	-1	0	0	0	0	-1	-1	-1	
M00205: N-Acetylglucosamine_transport_system	-1	-1	-1	-1	-1	-1	-1	-1	-1	-1	-1	-1	
M00206: Cellobiose_transport_system	0	0	0	0	0	0	0	0	0	0	0	-1	
M00207: Putative_multiple_sugar_transport_system	0	0	0	0	0	0	0	0	1	0	0	0	
M00208: Glycine_betaine/proline_transport_system	0	1	1	0	1	0	-1	0	1	0	0	1	varied
M00209: Osmoprotectant_transport_system	0	1	1	0	-1	0	0	0	0	0	-1	0	varied
M00210: Phospholipid_transport_system	1	0	0	0	1	0	0	0	1	1	1	1	
M00211: Putative_ABC_transport_system	0	0	0	1	-1	-1	-1	-1	-1	0	0	0	varied
M00212: Ribose_transport_system	0	1	1	1	1	1	1	1	1	1	1	0	
M00213: L-Arabinose_transport_system	-1	-1	-1	-1	-1	-1	-1	-1	-1	-1	-1	-1	
M00214: Methyl-galactoside_transport_system	-1	-1	-1	-1	-1	-1	-1	-1	-1	-1	-1	-1	
M00215: D-Xylose_transport_system	-1	-1	1	-1	-1	-1	-1	-1	1	-1	-1	-1	varied
M00216: Multiple_sugar_transport_system	-1	1	1	-1	-1	-1	-1	-1	0	-1	-1	-1	varied
M00217: D-Allose_transport_system	-1	-1	-1	-1	-1	-1	-1	-1	-1	-1	-1	-1	
M00218: Fructose_transport_system	-1	-1	-1	-1	-1	-1	-1	-1	-1	-1	-1	-1	
M00219: AI-2_transport_system	-1	-1	-1	-1	-1	-1	-1	-1	-1	-1	-1	-1	
M00220: Rhamnose_transport_system	-1	-1	0	-1	-1	-1	-1	-1	-1	-1	-1	-1	
M00221: Putative_simple_sugar_transport_system	1	1	1	1	1	1	0	1	1	1	1	1	
M00222: Phosphate_transport_system	1	1	1	1	1	1	1	1	1	1	1	1	core
M00223: Phosphonate_transport_system	1	1	1	1	1	1	1	1	1	1	1	1	core
M00224: Fluoroquinolone_transport_system	-1	-1	-1	-1	-1	-1	-1	-1	-1	-1	-1	-1	
M00225: Lysine/arginine/ornithine_transport_system	-1	-1	-1	-1	-1	0	0	-1	-1	-1	-1	-1	
M00226: Histidine_transport_system	-1	-1	-1	-1	-1	-1	-1	-1	-1	-1	-1	-1	
M00227: Glutamine_transport_system	-1	-1	-1	-1	-1	-1	-1	-1	-1	-1	-1	-1	
M00228: Putative_glutamine_transport_system	-1	-1	-1	-1	-1	-1	-1	-1	-1	-1	-1	-1	
M00229: Arginine_transport_system	-1	-1	-1	-1	-1	-1	-1	-1	-1	-1	-1	-1	
M00230: Glutamate/aspartate_transport_system	0	0	-1	0	0	-1	-1	0	1	1	1	1	varied
M00231: Octopine/nopaline_transport_system	-1	-1	-1	-1	-1	-1	-1	-1	-1	-1	-1	-1	
M00232: General_L-amino_acid_transport_system	-1	0	-1	1	1	0	0	0	1	-1	-1	0	varied
M00233: Glutamate_transport_system	-1	1	-1	-1	-1	-1	-1	-1	-1	-1	-1	-1	varied
M00234: Cystine_transport_system	-1	-1	-1	-1	-1	-1	-1	-1	0	-1	-1	-1	
M00235: Arginine/ornithine_transport_system	-1	-1	-1	-1	-1	-1	-1	-1	-1	-1	-1	-1	
M00236: Putative_polar_amino_acid_transport_system	1	1	1	1	1	1	1	1	1	1	1	1	core
M00237: Branched-chain_amino_acid_transport_system	1	1	1	1	1	1	1	1	1	1	1	1	core

Table S4. II

KEGG_Module	India_oil_refineries				Arctic												China_oil_refineries								
	I1	I2	I3	I4	A1	A10	A11	A12	A2	A3	A4	A5	A6	A7	A8	A9	C1	C2	C3	C4	C5	C6	C7	C8	C9
M00238: D-Methionine_transport_system	1	0.666667	1	0.333333	1	1	1	1	1	1	1	1	1	1	1	1	1	1	1	1	1	1	1	0.666667	1
M00239: Peptides/nickel_transport_system	1	1	1	1	1	1	1	1	1	1	1	1	1	1	1	1	1	1	1	1	1	1	1	1	1
M00240: Iron_complex_transport_system	1	1	1	1	1	1	1	1	1	1	1	1	1	1	1	1	1	1	1	1	1	1	1	1	1
M00241: Vitamin_B12_transport_system	0	0	0	0	0	0	0	0	0	0	0	0	0	0	0	0	0	0	0	0	0	0	0	0	0
M00242: Zinc_transport_system	0	0	1	1	1	0	0	1	0.666667	1	1	0	1	1	1	0	1	1	1	1	1	1	1	1	1
M00243: Manganese/iron_transport_system	0	0	0	0	0	0	0	0	0	0	0	0	0.333333	0.333333	0	0	1	1	1	1	1	0.666667	1	1	1
M00244: Putative_zinc/manganese_transport_system	0	0.333333	0.333333	0.333333	1	0.333333	1	1	1	1	1	1	1	1	0.333333	0	1	1	1	0.333333	0.333333	1	1	0.333333	1
M00245: Cobalt/nickel_transport_system	0	0	0.25	0	0.5	1	0.25	0.75	0.5	1	1	0	1	1	1	0	1	1	1	1	1	1	1	1	1
M00246: Nickel_transport_system	0	0	0.166667	0	0.333333	0.666667	0.166667	0.5	0.333333	0.666667	0.833333	0	0.666667	0.666667	0.666667	0	0.666667	0.666667	0.666667	0.666667	0.666667	0.666667	0.666667	0.666667	0.666667
M00247: Putative_ABC_transport_system	0	0	0	0	1	1	1	1	1	1	1	0.333333	1	0	1	0	0	0.333333	0	0.333333	0.333333	1	0.333333	0.333333	1
M00249: Capsular_polysaccharide_transport_system	0	0	0	0	0	0	0	0	0	0	0	0	0	0	0	0	0	0	0	0	0	0	0	0	0
M00250: Lipopolysaccharide_transport_system	1	1	1	1	1	1	1	1	1	1	1	1	1	1	1	1	1	1	1	1	1	1	1	1	1
M00251: Teichoic_acid_transport_system	0	0	0	0	0	0	0	0	0	0	0	0	0	0	0	0	0	0	0	0	0	0	0	0	0
M00252: Lipooligosaccharide_transport_system	0	1	1	1	0	0	0	0	0	0	0	0	0	0	0	0	0	0	0.5	0	0	0.5	0.5	0	0
M00253: Sodium_transport_system	0	0	0	0	0	0	0	0	0	0	0	0	0	0	0	0	0	1	0	1	1	0	0	1	0
M00254: ABC-2_type_transport_system	1	1	1	1	1	1	1	1	1	1	1	1	1	1	1	1	1	1	1	1	1	1	1	1	1
M00255: Lipoprotein-releasing_system	1	1	1	1	1	1	1	1	1	1	1	1	1	1	1	1	1	1	1	1	1	1	1	1	1
M00256: Cell_division_transport_system	1	1	1	1	1	1	1	1	1	1	1	1	1	1	1	1	1	1	1	1	1	1	1	1	1
M00257: Hemin_transport_system	0	0	0	0	0	0	0	0	0	0	0	0	0	0	0	0	0	0	0	0	0	0	0	0	0
M00258: Putative_ABC_transport_system	1	1	1	1	1	1	1	1	1	1	1	1	1	1	1	1	1	1	1	1	1	1	1	1	1
M00259: Heme_transport_system	1	1	1	1	1	1	1	1	0.666667	0.666667	1	0.666667	0.666667	0	0.666667	0.666667	1	1	1	1	1	1	1	1	1
M00260: DNA_polymerase_III_complex,_bacteria	0.636364	0.636364	0.636364	0.636364	0.636364	0.636364	0.636364	0.636364	0.636364	0.636364	0.636364	0.636364	0.636364	0.636364	0.636364	0.636364	0.636364	0.636364	0.545455	0.636364	0.636364	0.727273	0.545455	0.545455	0.636364
M00265: PTS_system,_glucose-specific_II_component	0	0	0	0	0	0	0	0	0	0	0	0	0	0	0	0	0	0	0	0	0	0	0	0.333333	0
M00266: PTS_system,_maltose_and_glucose-specific_II_component	0	0	0	0	0	0	0	0	0	0	0	0	0	0	0	0	0	0	0	0	0	0	0	0	0
M00267: PTS_system,_N-acetylglucosamine-specific_II_component	0	0	0	0	0	0	0	0	0	0	0	0	0	0	0	0	0	0	0	0	0	0	0	0	0
M00268: PTS_system,_arbutin-like_II_component	0	0	0	0	0	0	0	0	0	0	0	0	0	0	0	0	0	0	0	0	0	0	0	0	0
M00269: PTS_system,_sucrose-specific_II_component	0	0	0	0	0	0	0	0	0	0	0	0	0	0	0	0	0	0	0	0	0	0	0	0	0
M00270: PTS_system,_trehalose-specific_II_component	0	0	0	0	0	0	0	0	0	0	0	0	0	0	0	0	0	0	0	0	0	0	0	0	0
M00271: PTS_system,_beta-glucosides-specific_II_component	0	0	0	0	0	0	0.333333	0	0	0	0	0	0.333333	0.333333	0	0	0	0	0	0	0	0	0	0	0
M00272: PTS_system,_arbutin-,_cellobiose-,_and_salicin-specific_II_component	0	0	0	0	0	0	0	0	0	0	0	0	0	0	0	0	0	0	0	0	0	0	0	0	0
M00273: PTS_system,_fructose-specific_II_component	0	0	0	0	0.333333	0	0.333333	0	0	0	0	0	0.333333	0.333333	0	0	0.333333	0.666667	0.333333	0.666667	0.666667	0.333333	0.666667	0.666667	0.333333
M00274: PTS_system,_mannitol-specific_II_component	0	0	0	0	0	0	0	0	0	0	0	0	0.666667	0.666667	0	0	0	0	0	0	0	0	0	0	0
M00275: PTS_system,_cellobiose-specific_II_component	0	0	0	0	0	0	0	0	0	0	0	0	0.333333	0.333333	0	0	0	0	0	0	0	0	0	0	0
M00276: PTS_system,_mannose-specific_II_component	0	0	0	0	0.25	0	0	0	0	0.25	0.25	0	0	0	0.25	0	0	0.25	0	0.25	0.25	0	0.25	0.25	0
M00277: PTS_system,_N-acetylgalactosamine-specific_II_component	0	0	0	0	0	0	0	0	0	0	0	0	0	0	0	0	0	0	0	0	0	0	0	0	0
M00279: PTS_system,_galactitol-specific_II_component	0	0	0	0	0	0	0	0	0	0	0	0	0	0	0	0	0	0	0	0	0	0	0	0	0
M00280: PTS_system,_glucitol/sorbitol-specific_II_component	0	0	0	0	0	0	0	0	0	0	0	0	0.333333	0.333333	0	0	1	0	0.333333	0	0	0	0	0	0
M00281: PTS_system,_lactose-specific_II_component	0	0	0	0	0	0	0	0	0	0	0	0	0	0	0	0	0	0	0	0	0	0	0	0	0
M00282: PTS_system,_D-glucosamine-specific_II_component	0	0	0	0	0	0	0	0	0	0	0	0	0	0	0	0	0	0	0	0	0	0	0	0	0
M00283: PTS_system,_ascorbate-specific_II_component	0.333333	0	0.333333	0	0	0.333333	0.333333	0	0	0	0	0	0	0	0	0	0	0	0	0	0	0	0	0	0
M00287: PTS_system,_galactosamine-specific_II_component	0	0	0	0	0	0	0	0	0	0	0	0	0	0	0	0	0	0	0	0	0	0	0	0	0
M00288: RPA_complex	0	0	0	0	0	0	0	0	0	0	0	0	0	0	0	0	0	0	0	0	0	0	0	0	0
M00290: Holo-TFIIH_complex	0	0	0	0	0.1	0	0	0	0	0	0	0	0.1	0.1	0	0	0	0	0.1	0	0	0.1	0	0	0
M00295: BRCA1-associated_genome_surveillance_complex_(BASC)	0	0	0	0	0	0	0	0	0	0	0	0	0	0	0	0	0	0	0	0	0	0	0	0	0
M00296: BER_complex	0	0	0	0	0	0	0	0	0	0	0	0	0	0	0	0	0	0	0	0	0	0	0	0	0
M00298: Multidrug/hemolysin_transport_system	0	0	0	0	0	0	0	0	0	0	0	0	0	0	0	0	0	0	0	0	0	0	0	0	0
M00299: Spermidine/putrescine_transport_system	0	0	0.25	0.25	1	1	1	1	1	1	1	1	1	1	1	0	1	1	1	1	1	1	1	1	1
M00300: Putrescine_transport_system	1	0	0	0	0	0	0	0	0	0	0	0	0	0	0	0	0	0	0	0	0	0.25	0	0	0.25
M00301: Mannopine_transport_system	0	0	0	0	0	0	0	0	0	0	0	0	0	0	0	0	0	0	0	0	0	0	0	0	0
M00302: 2-Aminoethylphosphonate_transport_system	0	0	0	0	0	0	0	0	0	0	0	0	0	0	0	0	0	0	0	0	0	0	0	0	0
M00304: PTS_system,_fructose-specific_II_component	0	0	0	0	0	0	0	0	0	0	0	0	0	0	0	0	0	0	0	0	0	0	0	0	0
M00305: PTS_system,_2-O-A-mannosyl-D-glycerate-specific_II_component	0	0	0	0	0	0	0	0	0	0	0	0	0	0	0	0	0	0	0	0	0	0	0	0	0
M00306: PTS_system,_fructose-specific_II-like_component	0	0	0	0	0	0	0	0	0	0	0	0	0	0	0	0	0	0	0	0	0	0	0	0	0
M00307: Pyruvate_oxidation,_pyruvate_=>_acetyl-CoA	0.6	0.6	0.6	0.6	0.5	0.5	0.5	0.5	0.5	0.8	0.5	0.5	0.5	0.5	0.5	0.5	0.6	0.6	0.6	0.9	0.9	0.5	0.6	0.6	0.6
M00308: Semi-phosphorylative_Entner-Doudoroff_pathway,_gluconate_=>_glycerate-3P	0.571429	0.571429	0.428571	0.428571	0.571429	0.428571	0.571429	0.571429	0.571429	0.571429	0.428571	0.571429	0.571429	0.571429	0.428571	0.571429	0.571429	0.571429	0.571429	0.714286	0.571429	0.571429	0.571429	0.571429	0.571429
M00309: Non-phosphorylative_Entner-Doudoroff_pathway,_gluconate/galactonate_=>_glycerate	0	0.125	0.125	0.125	0.125	0.125	0.125	0.125	0.125	0.125	0.125	0.125	0.125	0.125	0.125	0.125	0.125	0.125	0.125	0.125	0.125	0.125	0.125	0.125	0.125
M00314: Bacitracin_transport_system	0	0	0	0	0	0	0	0	0	0	0	0	0	0	0	0	0	0	0	0	0	0	0	0	0
M00315: Uncharacterized_ABC_transport_system	0	0	0	0	0	0	0	0	0	0	0	0	0	0	0	0	0	0	0	0	0	0	0	0	0
M00316: Manganese_transport_system	0	0	0	0	0	0	0	0	0	0	0	0	0	0	0	0	0	0	0	0	0	0	0	0	0
M00317: Manganese/iron_transport_system	0	0	0	0	0	0	0	0	0	0	0	0	0	0	0	0	0	0	0	0	0	0	0	0	0
M00318: Iron/zinc/copper_transport_system	0	0	0	0	0	0	0	0	0	0	0	0	0	0	0	0	0	0	0	0	0	0	0	0.333333	0
M00319: Manganese/zinc/iron_transport_system	0	0	0	0	0	0	0	0	0	0	0	0	0	0	0	0	1	0.25	0.25	0.75	0.25	0.25	0.25	0.25	0.25
M00320: Lipopolysaccharide_export_system	1	1	1	1	1	1	1	1	1	1	1	1	1	1	1	1	1	1	1	1	1	1	1	1	1
M00321: Bicarbonate_transport_system	0	0	0	0	0	0	0	0	0	0	0	0	0	0	0	0	0	0	0	0	0	0	0	0	0
M00322: Neutral_amino_acid_transport_system	0	0	0	0	0	0	0	0	0	0	0	0	0	0	0	0	0	0	0.2	0	0.2	0	0.2	0.2	0.2
M00323: Urea_transport_system	0.2	1	0	1	0	0.2	0	0	0	0	0	0	0	0	0	0	1	1	1	0.8	0.8	0.8	0.8	0.8	1
M00324: Dipeptide_transport_system	0	0	0	0	0	0	0	0	0	0	0	0	0	0	0	0	0	0	0	0	0	0	0	0	0
M00325: alpha-Hemolysin/cyclolysin_transport_system	0.333333	0.333333	0.333333	0.333333	0.333333	0.333333	0.333333	0.333333	0.333333	0.333333	0.333333	0.333333	0.333333	0	0.333333	0.333333	0.333333	0.333333	0.333333	0.333333	0.333333	0.333333	0.333333	0.333333	0.333333
M00326: RTX_toxin_transport_system	0	0	0	0	0	0	0	0	0	0	0	0	0	0	0	0	0	0	0	0	0	0	0	0	0
M00327: S-Layer_protein_transport_system	0	0	0	0	0	0	0	0	0	0	0	0	0	0	0	0	0	0	0	0	0	0	0	0	0
M00328: Hemophore/metalloprotease_transport_system	0	0	0	0	0	0	0	0	0	0	0	0	0	0	0	0	0	0	0	0	0	0	0	0	0
M00329: Multiple_protein_transport_system	0	0	0	0	0	0	0	0	0	0	0	0	0	0	0	0	0	0	0	0	0	0	0	0	0
M00330: Adhesin_protein_transport_system	0	0	0	0	0	0	0	0	0	0	0	0	0	0	0	0	0	0.333333	0	0.333333	0.333333	0	0.333333	0.666667	0
M00331: Type_II_general_secretion_system	0.764706	0.764706	0.823529	0.764706	0.647059	0.705882	0.588235	0.588235	0.588235	0.647059	0.647059	0.588235	0.294118	0.588235	0.647059	0.588235	0.294118	0.470588	0.411765	0.411765	0.352941	0.705882	0.352941	0.470588	0.588235
M00332: Type_III_secretion_system	0	0	0	0	0	0	0	0	0	0	0	0	0	0	0	0	0	0	0	0	0	0	0	0	0
M00333: Type_IV_secretion_system	0.75	0.75	0.666667	0.666667	0.666667	0.666667	0.666667	0.666667	0.75	0.5	0.666667	0.75	0.333333	0.583333	0.75	0.75	0.5	0.416667	0.083333	0.333333	0.5	0.416667	0.416667	0.416667	0.416667
M00334: Type_VI_secretion_system	0.470588	0.117647	0.117647	0.117647	0.058824	0.058824	0.058824	0.058824	0.058824	0	0	0.058824	0.058824	0.058824	0	0.117647	0.058824	0.352941	0.058824	0.588235	0.588235	0.352941	0.294118	0.588235	0.235294
M00335: Sec_(secretion)_system	0.846154	0.846154	0.846154	0.846154	0.846154	0.846154	0.846154	0.923077	0.769231	0.769231	0.846154	0.846154	0.769231	0.769231	0.846154	0.846154	0.923077	0.923077	0.846154	0.846154	0.923077	0.846154	0.846154	0.846154	0.846154
M00336: Twin-arginine_translocation_(Tat)_system	0.75	0.75	0.75	0.75	0.75	0.75	0.75	0.75	0.75	0.75	0.75	0.75	0.75	0.75	0.75	0.75	0.75	0.75	0.75	0.75	0.75	0.75	0.75	0.75	0.75
M00338: Cysteine_biosynthesis,_homocysteine_+_serine_=>_cysteine	0.333333	0.333333	0.333333	0.333333	0.333333	0	0.333333	0.333333	0.333333	0.333333	0	0	0.333333	0.333333	0.333333	0	0.666667	0.666667	0.666667	0.666667	0.666667	0.666667	0.666667	0.666667	0.666667
M00339: RaxAB-RaxC_type_I_secretion_system	0.333333	0.333333	0.333333	0.333333	0.333333	0.333333	0.333333	0.333333	0.333333	0.333333	0.333333	0.333333	0.333333	0	0.333333	0.333333	0.333333	0.333333	0.333333	0.333333	0.333333	0.333333	0.333333	0.333333	0.333333
M00342: Bacterial_proteasome	0	0	0	0	0	0	0	0	0	0	0	0	0.25	0.25	0	0	1	1	1	1	1	1	1	1	0.25
M00343: Archaeal_proteasome	0	0	0	0	0	0	0	0	0	0	0	0	0	0	0	0	0.666667	0.666667	0.666667	0.666667	0.666667	0.666667	0.666667	0.666667	0
M00344: Formaldehyde_assimilation,_xylulose_monophosphate_pathway	0.5	0.5	0.5	0.5	0.5	0.5	0.5	0.5	0.5	0.5	0.5	0.5	0.5	0.5	0.5	0.5	0.5	0.5	0.5	0.5	0.5	0.5	0.5	0.5	0.5
M00345: Formaldehyde_assimilation,_ribulose_monophosphate_pathway	0.285714	0.285714	0.285714	0.285714	0.285714	0.285714	0.285714	0.285714	0.285714	0.285714	0.285714	0.285714	0.571429	0.571429	0.285714	0.285714	0.285714	0.285714	0.428571	0.285714	0.285714	0.285714	0.285714	0.285714	0.285714
M00346: Formaldehyde_assimilation,_serine_pathway	0.4	0.4	0.5	0.6	0.4	0.4	0.4	0.4	0.4	0.4	0.4	0.4	0.4	0.4	0.4	0.4	0.5	0.6	0.4	0.6	0.6	0.6	0.6	0.6	0.5
M00348: Glutathione_transport_system	0	0	0	0	0	0	0	0	0	0	0	0	0	0	0	0	0	0	0	0	0	0	0	0	0
M00349: Microcin_C_transport_system	0	0	1	1	0	0	0	0	0	0	0	0	0	0	0	0	0	0	0	0	0	1	0	0	1
M00350: Capsaicin_biosynthesis,_L-Phenylalanine_=>_Capsaicin	0	0	0	0	0	0	0	0	0	0	0	0	0	0	0	0	0	0	0	0	0	0	0	0	0
M00355: Spliceosome,_35S_U5-snRNP	0	0	0	0	0	0	0	0	0	0	0	0	0	0	0	0	0	0	0	0	0	0	0	0	0
M00356: Methanogenesis,_methanol_=>_methane	0	0	0	0	0	0	0	0	0	0	0	0	0	0	0	0	0	0.222222	0	0.333333	0	0	0.222222	0	0
M00357: Methanogenesis,_acetate_=>_methane	0	0	0	0	0	0	0	0	0	0	0	0	0	0	0	0	0	0.285714	0	0.285714	0	0.190476	0.238095	0	0
M00358: Coenzyme_M_biosynthesis	0	0	0.2	0	0.2	0.2	0.2	0.2	0.2	0.2	0.2	0.2	0.2	0.2	0.2	0	0.2	0.2	0.2	0.2	0.2	0.2	0.2	0.2	0.2
M00359: Aminoacyl-tRNA_biosynthesis,_eukaryotes	0.909091	0.909091	0.954545	0.909091	0.954545	0.909091	0.909091	0.954545	0.909091	0.954545	0.909091	0.954545	0.954545	0.909091	0.909091	0.909091	0.954545	0.954545	0.954545	0.954545	0.954545	0.954545	0.954545	0.954545	0.954545
M00360: Aminoacyl-tRNA_biosynthesis,_prokaryotes	0.741935	0.741935	0.774194	0.741935	0.741935	0.709677	0.709677	0.741935	0.709677	0.741935	0.709677	0.741935	0.741935	0.709677	0.709677	0.709677	0.774194	0.741935	0.741935	0.806452	0.774194	0.774194	0.741935	0.774194	0.774194

KEGG_Module	Mangrove			Marine_sediments							Taiga_upper_active_layer				Taiga_bottom_active_layer				Taiga_permafrost_layer			
	M1	M2	M3	DWH1	DWH2	DWH3	DWH4	DWH5	DWH6	DWH7	Tu1	Tu2	Tu3	Tu4	Tb1	Tb2	Tb3	Tb4	Tp1	Tp2	Tp3	Tp4
M00238: D-Methionine_transport_system	1	1	1	1	1	1	1	1	0.333333	1	1	1	1	1	1	1	1	1	1	1	1	1
M00239: Peptides/nickel_transport_system	1	1	1	1	1	1	1	1	1	1	1	0.8	1	1	1	1	1	1	1	1	1	1
M00240: Iron_complex_transport_system	1	1	1	1	1	1	1	1	1	1	1	1	1	1	1	1	1	1	1	1	1	1
M00241: Vitamin_B12_transport_system	0	0	0	0	0	0	0	0	0	0	0	0	0	0	0	0	0	0	0	0	0	0
M00242: Zinc_transport_system	1	1	1	1	1	1	1	1	1	1	1	0	1	0	0	0	1	0	0	0	1	0
M00243: Manganese/iron_transport_system	0	0	0	0	0	0	0	0	0	0	0	0	0.666667	0	0	0	0	0	0	0	0.666667	0
M00244: Putative_zinc/manganese_transport_system	0	0.333333	0	0	0	0	0	0	0	0	0.333333	0	1	0	0.333333	0.333333	0.333333	0	0.333333	0.333333	1	0
M00245: Cobalt/nickel_transport_system	1	1	1	1	0.5	1	0.5	0.5	0	0.5	1	0	1	0	0.75	0.5	0.75	0	0.5	0	0.75	0
M00246: Nickel_transport_system	0.666667	0.666667	0.666667	0.666667	0.333333	0.666667	0.333333	0.333333	0	0.333333	0.666667	0	0.666667	0	0.5	0.333333	0.5	0	0.333333	0	0.5	0
M00247: Putative_ABC_transport_system	1	0.666667	0.666667	0.333333	0	0.333333	0	0	0	0	0.333333	0	1	0	0	0	0	0	0.333333	0.333333	0	0
M00249: Capsular_polysaccharide_transport_system	0	0	0	0	0	0	0	0	0.666667	0	1	0.666667	0	0.333333	0.333333	1	0	1	1	1	0	1
M00250: Lipopolysaccharide_transport_system	1	1	1	1	1	1	1	1	1	1	1	1	1	1	1	1	1	1	1	1	1	1
M00251: Teichoic_acid_transport_system	0	0	0	0	0	0	0	0	0	0	0	0	1	0	0	0	0	0	0	0	0	0
M00252: Lipooligosaccharide_transport_system	0	0	0	0	0	0	0	0	0	0	0	0	0.5	0	0	0	0	0	0	1	0.5	0
M00253: Sodium_transport_system	0	0	0	0	0	0	0	0	0	0	0	0	0	0	0	0	0	0	0	0	0	0
M00254: ABC-2_type_transport_system	1	1	1	1	1	1	1	1	1	1	1	1	1	1	1	1	1	1	1	1	1	1
M00255: Lipoprotein-releasing_system	1	1	1	1	1	1	1	1	1	1	1	1	1	1	1	1	1	1	1	1	1	1
M00256: Cell_division_transport_system	1	1	1	1	1	1	1	1	1	1	1	1	1	1	1	1	1	1	1	1	1	1
M00257: Hemin_transport_system	0	0	0	0	0	0	0	0	0	0	0	0	0	0	0	0	0	0	0	0	0	0
M00258: Putative_ABC_transport_system	1	1	1	1	1	1	1	1	1	1	1	1	1	1	1	1	1	1	1	1	1	1
M00259: Heme_transport_system	1	1	1	1	1	1	1	1	1	1	1	1	1	1	1	1	1	1	1	1	1	1
M00260: DNA_polymerase_III_complex,_bacteria	0.636364	0.636364	0.636364	0.636364	0.636364	0.636364	0.636364	0.636364	0.636364	0.636364	0.636364	0.636364	0.545455	0.636364	0.636364	0.636364	0.636364	0.636364	0.636364	0.636364	0.545455	0.636364
M00265: PTS_system,_glucose-specific_II_component	0	0	0	0	0	0	0	0	0	0	0	0	0	0	0	0	0	0	0	0	0	0
M00266: PTS_system,_maltose_and_glucose-specific_II_component	0	0	0	0	0	0	0	0	0	0	0	0	0	0	0	0	0	0	0	0	0	0
M00267: PTS_system,_N-acetylglucosamine-specific_II_component	0	0	0	0	0	0	0	0	0	0	0.333333	0	0	0	0.333333	0	0.333333	0.333333	0	0	0	0.333333
M00268: PTS_system,_arbutin-like_II_component	0	0	0	0	0	0	0	0	0	0	0	0	0	0	0	0	0	0	0	0	0	0
M00269: PTS_system,_sucrose-specific_II_component	0	0	0	0	0	0	0	0	0	0	0	0	0	0	0	0	0	0	0	0	0	0
M00270: PTS_system,_trehalose-specific_II_component	0	0	0	0	0	0	0	0	0	0	0	0	0	0	0	0	0	0	0	0	0	0
M00271: PTS_system,_beta-glucosides-specific_II_component	0	0	0	0	0	0	0	0	0	0	0	0	0.333333	0	0	0	0	0	0	0	0	0
M00272: PTS_system,_arbutin-,_cellobiose-,_and_salicin-specific_II_component	0	0	0	0	0	0	0	0	0	0	0	0	0	0	0	0	0	0	0	0	0	0
M00273: PTS_system,_fructose-specific_II_component	0.666667	0.666667	0.666667	0	0	0	0	0.333333	0.333333	0	0	0	0.333333	0	0	0	0	0	0	0	0	0
M00274: PTS_system,_mannitol-specific_II_component	0	0	0	0	0	0	0	0	0	0	0	0	0	0	0	0	0	0	0	0	0	0
M00275: PTS_system,_cellobiose-specific_II_component	0	0	0	0	0	0	0	0	0	0	0	0	0	0	0	0	0	0	0	0	0	0
M00276: PTS_system,_mannose-specific_II_component	0.25	0.25	0.25	0.25	0.25	0.25	0	0	0.25	0	0.25	0.25	0	0.25	0.25	0.25	0.25	0.25	0.25	0.25	0	0.25
M00277: PTS_system,_N-acetylgalactosamine-specific_II_component	0	0	0	0	0	0	0	0	0	0	0	0	0	0	0	0	0	0	0	0	0	0
M00279: PTS_system,_galactitol-specific_II_component	0	0	0	0	0	0	0	0	0	0	0	0	0	0	0	0	0	0	0	0	0	0
M00280: PTS_system,_glucitol/sorbitol-specific_II_component	0	0	0	0	0	0	0	0	0	0	0	0	0	0	0	0	0	0	0	0	0	0
M00281: PTS_system,_lactose-specific_II_component	0	0	0	0	0	0	0	0	0	0	0	0	0	0	0	0	0	0	0	0	0	0
M00282: PTS_system,_D-glucosamine-specific_II_component	0	0	0	0	0	0	0	0	0	0	0	0	0	0	0	0	0	0	0	0	0	0
M00283: PTS_system,_ascorbate-specific_II_component	0	0	0	0	0	0	0	0	0	0	0	0	0.333333	0	0	0	0	0	0	0.333333	0	0
M00287: PTS_system,_galactosamine-specific_II_component	0	0	0	0	0	0	0	0	0	0	0	0	0	0	0	0	0	0	0	0	0	0
M00288: RPA_complex	0	0	0	0	0	0	0	0	0	0	0	0	0	0	0	0	0	0	0	0	0	0
M00290: Holo-TFIIH_complex	0	0	0	0	0	0	0	0	0	0	0	0	0.1	0	0	0	0	0	0	0	0	0
M00295: BRCA1-associated_genome_surveillance_complex_(BASC)	0	0	0	0	0	0	0	0	0	0	0	0	0	0	0	0	0	0	0	0	0	0
M00296: BER_complex	0	0	0	0	0	0	0	0	0	0	0	0	0	0	0	0	0	0	0	0	0	0
M00298: Multidrug/hemolysin_transport_system	0	0	0	0	0	0	0	0	0	0	0	0	0	0	0	0	0	0	0	0	0	0
M00299: Spermidine/putrescine_transport_system	1	1	1	1	1	1	1	1	1	1	1	1	1	1	1	1	1	1	1	0	1	1
M00300: Putrescine_transport_system	0.25	0.25	0.25	0.25	1	0.25	1	1	1	1	0.25	0	0	0	0	0	0	0	0	0	0	0
M00301: Mannopine_transport_system	0	0	0	0	0	0	0	0	0	0	0	0	0	0	0	0	0	0	0	0	0	0
M00302: 2-Aminoethylphosphonate_transport_system	0	0	0	0	0	0	0	0	0	0	0	0	0	0	0	0	0	0	0	0	0	0
M00304: PTS_system,_fructose-specific_II_component	0	0	0	0	0	0	0	0	0	0	0	0	0	0	0	0	0	0	0	0	0	0
M00305: PTS_system,_2-O-A-mannosyl-D-glycerate-specific_II_component	0	0	0	0	0	0	0	0	0	0	0	0	0	0	0	0	0	0	0	0	0	0
M00306: PTS_system,_fructose-specific_II-like_component	0	0	0	0	0	0	0	0	0	0	0	0	0	0	0	0	0	0	0	0	0	0
M00307: Pyruvate_oxidation,_pyruvate_=>_acetyl-CoA	1	0.9	1	0.8	0.5	0.8	0.5	0.5	0.5	0.5	0.5	0.4	0.5	0.5	0.4	0.5	0.5	0.4	0.5	0.5	0.5	0.5
M00308: Semi-phosphorylative_Entner-Doudoroff_pathway,_gluconate_=>_glycerate-3P	0.571429	0.571429	0.571429	0.428571	0.428571	0.428571	0.428571	0.428571	0.571429	0.428571	0.571429	0.571429	0.571429	0.571429	0.571429	0.571429	0.571429	0.571429	0.571429	0.428571	0.714286	0.571429
M00309: Non-phosphorylative_Entner-Doudoroff_pathway,_gluconate/galactonate_=>_glycerate	0.125	0.125	0.125	0.125	0.125	0.125	0.125	0.125	0	0.125	0	0	0.125	0	0	0.125	0	0	0	0	0.125	0
M00314: Bacitracin_transport_system	0	0	0	0	0	0	0	0	0	0	0	0	0	0	0	0	0	0	0	0	0	0
M00315: Uncharacterized_ABC_transport_system	0	0	0	0	0	0	0	0	0	0	0	0	0	0	0	0	0	0	0	0	0	0
M00316: Manganese_transport_system	0	0	0	0	0	0	0	0	0	0	0	0	1	0	0	0	0	0	0	0	0	0
M00317: Manganese/iron_transport_system	0	0	0	0	0	0	0	0	0	0	0	0	0	0	0	0	0	0	0	0	0	0
M00318: Iron/zinc/copper_transport_system	0	0	0	0	0	0	0	0	0	0	0	0	0	0	0	0	0	0	0	0	0	0
M00319: Manganese/zinc/iron_transport_system	0	0	0	0	0	0	0	0	0.25	0	0	0	0.25	0	0	0	0.25	0	0	0	0.25	0
M00320: Lipopolysaccharide_export_system	1	1	1	1	1	1	1	1	1	1	1	1	0.666667	1	1	1	1	1	1	1	0.333333	1
M00321: Bicarbonate_transport_system	0	0	0	0	0	0	0	0	0	0	0	0	0	0	0	0	0	0	0	0	0	0
M00322: Neutral_amino_acid_transport_system	0	0	0	0	0	0	0	0	0	0	0	0	0	0	0	0	0	0	0	0	0	0
M00323: Urea_transport_system	0.2	0.2	0.2	0.2	0.2	0.2	0.2	0.8	0.8	0.8	1	0	0	1	1	1	0	0	1	1	0	0
M00324: Dipeptide_transport_system	0	0	0	0	0	0	0	0	0	0	0	0	0	0	0	0	0	0	0	0	0	0
M00325: alpha-Hemolysin/cyclolysin_transport_system	0.333333	0.333333	0.333333	0.333333	0.333333	0.333333	0.333333	0	0.333333	0.333333	0.333333	0.333333	0.333333	0.333333	0.333333	0.333333	0.333333	0.333333	0.333333	0.333333	0.333333	0.333333
M00326: RTX_toxin_transport_system	0	0	0	0	0	0	0	0	0	0	0	0	0.25	0	0	0	0.25	0	0.25	0	0	0
M00327: S-Layer_protein_transport_system	0	0	0	0	0	0	0	0	0	0	0	0	0	0	0	0	0	0	0	0	0	0
M00328: Hemophore/metalloprotease_transport_system	0	0	0	0	0	0	0	0	0	0	0	0	0	0	0	0	0	0	0	0	0	0
M00329: Multiple_protein_transport_system	0	0	0	0	0	0	0	0	0	0	0	0	0	0	0	0	0	0	0	0	0	0
M00330: Adhesin_protein_transport_system	0	0	0.333333	0	0	0	0.666667	1	0.666667	0	0	0	0	0	0	0	0	0	0	0	0	0
M00331: Type_II_general_secretion_system	0.764706	0.764706	0.764706	0.823529	0.764706	0.764706	0.823529	0.764706	0.529412	0.764706	0.588235	0.352941	0.176471	0.352941	0.352941	0.352941	0.294118	0.352941	0.588235	0.647059	0.235294	0.352941
M00332: Type_III_secretion_system	0	0	0	0	0	0	0	0	0	0	0	0	0	0	0	0	0	0	0	0	0	0
M00333: Type_IV_secretion_system	0.083333	0.666667	0.5	0.666667	0.666667	0.083333	0.666667	0	0.666667	0.666667	0.833333	0.833333	0.416667	0.75	0.833333	0.75	0.75	0.833333	0.833333	0.75	0.083333	0.833333
M00334: Type_VI_secretion_system	0.235294	0.294118	0.470588	0.647059	0.705882	0.647059	0.764706	0.647059	0.705882	0.764706	0.058824	0	0.058824	0	0	0.117647	0	0	0.117647	0.176471	0.058824	0
M00335: Sec_(secretion)_system	0.846154	0.846154	0.923077	0.846154	0.846154	0.846154	0.846154	0.846154	0.923077	0.846154	0.923077	0.923077	0.769231	0.923077	0.923077	0.846154	0.923077	0.923077	0.923077	0.846154	0.769231	0.923077
M00336: Twin-arginine_translocation_(Tat)_system	0.75	0.75	0.75	0.75	0.75	0.75	0.75	0.75	0.75	0.75	0.75	0.75	0.75	0.75	0.75	0.75	0.75	0.75	0.75	0.75	0.75	0.75
M00338: Cysteine_biosynthesis,_homocysteine_+_serine_=>_cysteine	0.666667	0.666667	0.666667	0	0	0	0	0	0.333333	0	0	0	0.666667	0	0	0	0.333333	0	0	0	0.666667	0
M00339: RaxAB-RaxC_type_I_secretion_system	0.333333	0.333333	0.333333	0	0	0	0.333333	0.333333	0.333333	0.333333	0.333333	0.333333	0.333333	0.333333	0.333333	0.333333	0	0.333333	0.333333	0.333333	0	0.333333
M00342: Bacterial_proteasome	0	0	0	0	0	0	0	0	0.25	0	0	0	1	0	0	0	0	0	0	0	0.5	0
M00343: Archaeal_proteasome	0	0	0	0	0	0	0	0	0	0	0	0	0.666667	0	0	0	0	0	0	0	0	0
M00344: Formaldehyde_assimilation,_xylulose_monophosphate_pathway	0.5	0.5	0.5	0.5	0.5	0.5	0.5	0.5	0.5	0.5	0.5	0.5	0.25	0.5	0.5	0.5	0.75	0.5	0.5	0.5	0.5	0.5
M00345: Formaldehyde_assimilation,_ribulose_monophosphate_pathway	0.285714	0.285714	0.285714	0.285714	0.285714	0.285714	0.285714	0.285714	0.285714	0.285714	0.285714	0	0.285714	0.285714	0.285714	0.285714	0.285714	0.142857	0.285714	0.285714	0.428571	0.142857
M00346: Formaldehyde_assimilation,_serine_pathway	0.5	0.4	0.5	0.4	0.5	0.4	0.5	0.5	0.6	0.6	0.4	0.4	0.5	0.5	0.4	0.4	0.4	0.4	0.4	0.4	0.5	0.4
M00348: Glutathione_transport_system	0	0	0	0	0	0	0	0	0	0	0	0	0	0	0	0	0	0	0	0	0	0.25
M00349: Microcin_C_transport_system	0	0	0	0	0	0	0	1	1	1	1	0	0	1	0.25	0	0	0	1	0.25	0	0
M00350: Capsaicin_biosynthesis,_L-Phenylalanine_=>_Capsaicin	0	0	0	0	0	0	0	0	0	0	0	0	0	0	0	0	0	0	0	0	0	0
M00355: Spliceosome,_35S_U5-snRNP	0	0	0	0	0	0	0	0	0	0	0	0	0	0	0	0	0	0	0	0	0	0
M00356: Methanogenesis,_methanol_=>_methane	0	0	0	0	0	0	0	0	0	0	0	0	0	0	0	0	0	0	0	0	0	0
M00357: Methanogenesis,_acetate_=>_methane	0	0.333333	0	0	0	0	0	0	0	0	0	0	0	0	0	0	0	0	0	0	0	0
M00358: Coenzyme_M_biosynthesis	0.2	0.2	0.2	0.2	0	0.2	0.2	0	0.2	0	0	0	0.2	0	0	0	0	0	0	0	0.2	0
M00359: Aminoacyl-tRNA_biosynthesis,_eukaryotes	0.954545	0.954545	0.954545	0.909091	0.909091	0.909091	0.909091	0.909091	0.909091	0.909091	0.863636	0.772727	0.863636	0.772727	0.772727	0.863636	0.863636	0.772727	0.863636	0.863636	0.863636	0.818182
M00360: Aminoacyl-tRNA_biosynthesis,_prokaryotes	0.774194	0.774194	0.774194	0.709677	0.709677	0.741935	0.741935	0.741935	0.741935	0.741935	0.709677	0.645161	0.645161	0.645161	0.645161	0.709677	0.709677	0.645161	0.709677	0.709677	0.612903	0.677419

KEGG_Module	Oil_sands_core							Oil_sands_tailings_pond_upper				Oil_sands_tailings_pond_median			Oil_sands_tailings_pond_deep				1 if median >= 90% for the samples per site; -1 if median <= 10% for the samples per site												Kind of Module inferred (Core or Varied)
	OSC1	OSC12	OSC3	OSC4	OSC5	OSC7	OSC9	OSTPu1	OSTPu2	OSTPu4	OSTPu3	OSTPm2	OSTPm6	OSTPm4	OSTPd1	OSTPd2	OSTPd3	OSTPd4	India_oil_refineries	Arctic	China_oil_refineries	Mangrove	Marine_sediments	Taiga_upper_active_layer	Taiga_bottom_active_layer	Taiga_permafrost_layer	Oil_sands_core	Oil_sands_tailings_pond_upper	Oil_sands_tailings_pond_median	Oil_sands_tailings_pond_deep	
M00238: D-Methionine_transport_system	1	1	1	1	1	1	1	1	1	1	1	1	1	1	1	1	1	1	0	1	1	1	1	1	1	1	1	1	1	1	
M00239: Peptides/nickel_transport_system	1	1	1	1	1	1	1	1	1	1	1	1	1	1	1	1	1	1	1	1	1	1	1	1	1	1	1	1	1	1	core
M00240: Iron_complex_transport_system	1	1	1	1	1	1	1	1	1	1	1	1	1	1	1	1	1	1	1	1	1	1	1	1	1	1	1	1	1	1	core
M00241: Vitamin_B12_transport_system	0	0	0	0	0	0	0	0	0	0	0	0	0	0	0	0	0	0	-1	-1	-1	-1	-1	-1	-1	-1	-1	-1	-1	-1	
M00242: Zinc_transport_system	1	0.666667	1	1	0.666667	1	1	0	0	1	1	1	1	0.666667	0	0	1	1	0	1	1	1	1	0	-1	-1	1	0	1	0	varied
M00243: Manganese/iron_transport_system	0	0	0	0	0	0	0	0	0	0	0	0	0	0	0	0	0	0	-1	-1	1	-1	-1	-1	-1	-1	-1	-1	-1	-1	varied
M00244: Putative_zinc/manganese_transport_system	0	0	0	0	0	1	1	0.333333	0.333333	0.333333	0.333333	0.666667	0.333333	0.333333	0.333333	0	0.333333	0	0	1	1	-1	-1	0	0	0	-1	0	0	0	varied
M00245: Cobalt/nickel_transport_system	0	0	0	0	0	0	0	1	1	1	1	1	1	1	1	0	1	1	-1	0	1	1	0	0	0	0	-1	1	1	1	varied
M00246: Nickel_transport_system	0	0	0	0	0	0	0	0.666667	0.666667	0.666667	0.666667	0.666667	0.833333	0.666667	0.666667	0	0.666667	0.833333	-1	0	0	0	0	0	0	0	-1	0	0	0	
M00247: Putative_ABC_transport_system	1	1	1	1	1	1	1	1	1	1	0.333333	0.333333	1	1	1	1	1	1	-1	1	0	0	-1	0	-1	0	1	1	1	1	varied
M00249: Capsular_polysaccharide_transport_system	0	0	0	0	0	0	0	0	0	0	0	0	0	0	0	0	0	0	-1	-1	-1	-1	-1	0	0	1	-1	-1	-1	-1	varied
M00250: Lipopolysaccharide_transport_system	1	1	1	1	1	1	1	1	1	1	1	1	1	1	1	1	1	1	1	1	1	1	1	1	1	1	1	1	1	1	core
M00251: Teichoic_acid_transport_system	0	0	0	0	0	0	0	0	0	0	0	0	0	0	0	0	0	0	-1	-1	-1	-1	-1	-1	-1	-1	-1	-1	-1	-1	
M00252: Lipooligosaccharide_transport_system	1	1	1	0	0	1	1	1	1	1	1	1	0	1	1	1	1	0	1	-1	-1	-1	-1	-1	-1	0	1	1	1	1	varied
M00253: Sodium_transport_system	0	0	0	0	0	0	0	0	0	0	0	0	0	0	0	0	0	0	-1	-1	-1	-1	-1	-1	-1	-1	-1	-1	-1	-1	
M00254: ABC-2_type_transport_system	1	1	1	1	1	1	1	1	1	1	1	1	1	1	1	1	1	1	1	1	1	1	1	1	1	1	1	1	1	1	core
M00255: Lipoprotein-releasing_system	1	1	1	1	1	1	1	1	1	1	1	1	1	1	1	1	1	1	1	1	1	1	1	1	1	1	1	1	1	1	core
M00256: Cell_division_transport_system	1	1	1	1	1	1	1	1	1	1	1	1	1	1	1	1	1	1	1	1	1	1	1	1	1	1	1	1	1	1	core
M00257: Hemin_transport_system	0	0	0	0	0	0	0	0	0	0	0	0	0	0	0	0	0	0	-1	-1	-1	-1	-1	-1	-1	-1	-1	-1	-1	-1	
M00258: Putative_ABC_transport_system	1	1	1	1	1	1	1	1	1	1	1	1	1	1	1	1	1	1	1	1	1	1	1	1	1	1	1	1	1	1	core
M00259: Heme_transport_system	1	1	1	1	1	1	1	0	1	0	0	1	1	0	1	0	1	1	1	0	1	1	1	1	1	1	1	-1	1	1	varied
M00260: DNA_polymerase_III_complex,_bacteria	0.636364	0.727273	0.636364	0.727273	0.727273	0.727273	0.727273	0.636364	0.636364	0.727273	0.636364	0.636364	0.636364	0.636364	0.636364	0.636364	0.636364	0.636364	0	0	0	0	0	0	0	0	0	0	0	0	
M00265: PTS_system,_glucose-specific_II_component	0	0	0	0	0	0	0	0	0	0	0	0	0	0	0	0	0	0	-1	-1	-1	-1	-1	-1	-1	-1	-1	-1	-1	-1	
M00266: PTS_system,_maltose_and_glucose-specific_II_component	0	0	0	0	0	0	0	0	0	0	0	0	0	0	0	0	0	0	-1	-1	-1	-1	-1	-1	-1	-1	-1	-1	-1	-1	
M00267: PTS_system,_N-acetylglucosamine-specific_II_component	0	0	0	0	0	0	0	0	0	0	0	0	0	0	0	0	0	0	-1	-1	-1	-1	-1	-1	0	-1	-1	-1	-1	-1	
M00268: PTS_system,_arbutin-like_II_component	0	0	0	0	0	0	0	0	0	0	0	0	0	0	0	0	0	0	-1	-1	-1	-1	-1	-1	-1	-1	-1	-1	-1	-1	
M00269: PTS_system,_sucrose-specific_II_component	0	0	0.333333	0	0	0	0	0	0	0	0	0	0	0	0	0	0	0	-1	-1	-1	-1	-1	-1	-1	-1	-1	-1	-1	-1	
M00270: PTS_system,_trehalose-specific_II_component	0	0	0	0	0	0	0	0	0	0	0	0	0	0	0	0	0	0	-1	-1	-1	-1	-1	-1	-1	-1	-1	-1	-1	-1	
M00271: PTS_system,_beta-glucosides-specific_II_component	0	0	0	0	0	0	0	0	0	0.333333	0	0	0	0	0	0	0	0	-1	-1	-1	-1	-1	-1	-1	-1	-1	-1	-1	-1	
M00272: PTS_system,_arbutin-,_cellobiose-,_and_salicin-specific_II_component	0	0	0	0	0	0	0	0	0	0	0	0	0	0	0	0	0	0	-1	-1	-1	-1	-1	-1	-1	-1	-1	-1	-1	-1	
M00273: PTS_system,_fructose-specific_II_component	0	0	0.333333	0	0	0	0	0	0	0.666667	0	0	0.333333	0	0	0	0	0	-1	-1	0	0	-1	-1	-1	-1	-1	-1	-1	-1	
M00274: PTS_system,_mannitol-specific_II_component	0	0	0	0	0	0	0	0	0	0	0	0	0	0	0	0	0	0	-1	-1	-1	-1	-1	-1	-1	-1	-1	-1	-1	-1	
M00275: PTS_system,_cellobiose-specific_II_component	0	0	0	0	0	0	0	0	0	1	0	0	0	0	0	0	0	0	-1	-1	-1	-1	-1	-1	-1	-1	-1	-1	-1	-1	
M00276: PTS_system,_mannose-specific_II_component	0	0	0	0	0	0	0.25	0	0	1	0	0.25	0.25	0	0	0	0	0.25	-1	-1	0	0	0	0	0	0	-1	-1	0	-1	
M00277: PTS_system,_N-acetylgalactosamine-specific_II_component	0	0	0	0	0	0	0	0	0	0	0	0	0	0	0	0	0	0	-1	-1	-1	-1	-1	-1	-1	-1	-1	-1	-1	-1	
M00279: PTS_system,_galactitol-specific_II_component	0	0	0	0	0	0	0	0	0	0.666667	0	0	0	0	0	0	0	0	-1	-1	-1	-1	-1	-1	-1	-1	-1	-1	-1	-1	
M00280: PTS_system,_glucitol/sorbitol-specific_II_component	0	0	0	0	0	0	0	0	0	0.333333	0	0	0.333333	0	0	0	0	0	-1	-1	-1	-1	-1	-1	-1	-1	-1	-1	-1	-1	
M00281: PTS_system,_lactose-specific_II_component	0	0	0	0	0	0	0	0	0	0	0	0	0	0	0	0	0	0	-1	-1	-1	-1	-1	-1	-1	-1	-1	-1	-1	-1	
M00282: PTS_system,_D-glucosamine-specific_II_component	0	0	0	0	0	0	0	0	0	0	0	0	0	0	0	0	0	0	-1	-1	-1	-1	-1	-1	-1	-1	-1	-1	-1	-1	
M00283: PTS_system,_ascorbate-specific_II_component	0.333333	0.333333	0.333333	0.333333	0.333333	0.333333	0.333333	0.333333	0.333333	0	0.333333	0.333333	0	0.333333	0.333333	0.333333	0.333333	0	0	-1	-1	-1	-1	-1	-1	-1	0	0	0	0	
M00287: PTS_system,_galactosamine-specific_II_component	0	0	0	0	0	0	0	0	0	0	0	0	0	0	0	0	0	0	-1	-1	-1	-1	-1	-1	-1	-1	-1	-1	-1	-1	
M00288: RPA_complex	0	0	0	0	0	0	0	0	0	0	0	0	0	0	0	0	0	0	-1	-1	-1	-1	-1	-1	-1	-1	-1	-1	-1	-1	
M00290: Holo-TFIIH_complex	0	0	0	0	0	0	0	0	0.1	0	0	0	0	0.1	0.1	0.1	0.1	0	-1	-1	-1	-1	-1	-1	-1	-1	-1	-1	-1	0	
M00295: BRCA1-associated_genome_surveillance_complex_(BASC)	0	0	0	0	0	0	0	0	0	0	0	0	0	0	0	0	0	0	-1	-1	-1	-1	-1	-1	-1	-1	-1	-1	-1	-1	
M00296: BER_complex	0	0	0	0	0	0	0	0	0	0	0	0	0	0	0	0	0	0	-1	-1	-1	-1	-1	-1	-1	-1	-1	-1	-1	-1	
M00298: Multidrug/hemolysin_transport_system	0	0	0	0	0	0	0	0	0	0	0	0	0	0	0	0	0	0	-1	-1	-1	-1	-1	-1	-1	-1	-1	-1	-1	-1	
M00299: Spermidine/putrescine_transport_system	0	0	0.25	0	0	0	1	0	0	1	1	1	1	0.25	0	0	0.25	1	0	1	1	1	1	1	1	1	-1	0	1	0	varied
M00300: Putrescine_transport_system	0	0	0	0	0	0	0	0	0	0	0	0.25	0	0	0	0	0	0	-1	-1	-1	0	1	-1	-1	-1	-1	-1	-1	-1	
M00301: Mannopine_transport_system	0	0	0	0	0	0	0	0	0	0	0	0	0	0	0	0	0	0	-1	-1	-1	-1	-1	-1	-1	-1	-1	-1	-1	-1	
M00302: 2-Aminoethylphosphonate_transport_system	0	0	0	0	0	0	0	0	0	0	0	0	0	0	0	0	0	0	-1	-1	-1	-1	-1	-1	-1	-1	-1	-1	-1	-1	
M00304: PTS_system,_fructose-specific_II_component	0	0	0	0	0	0	0	0	0	0	0	0	0	0	0	0	0	0	-1	-1	-1	-1	-1	-1	-1	-1	-1	-1	-1	-1	
M00305: PTS_system,_2-O-A-mannosyl-D-glycerate-specific_II_component	0	0	0	0	0	0	0	0	0	0	0	0	0	0	0	0	0	0	-1	-1	-1	-1	-1	-1	-1	-1	-1	-1	-1	-1	
M00306: PTS_system,_fructose-specific_II-like_component	0	0	0	0	0	0	0	0	0	0	0	0	0	0	0	0	0	0	-1	-1	-1	-1	-1	-1	-1	-1	-1	-1	-1	-1	
M00307: Pyruvate_oxidation,_pyruvate_=>_acetyl-CoA	0.5	0.5	0.5	0.5	0.5	0.5	0.5	0.5	0.5	1	0.6	0.9	1	0.5	0.5	0.5	0.5	1	0	0	0	1	0	0	0	0	0	0	0	0	
M00308: Semi-phosphorylative_Entner-Doudoroff_pathway,_gluconate_=>_glycerate-3P	0.571429	0.571429	0.571429	0.571429	0.571429	0.571429	0.571429	0.428571	0.428571	0.428571	0.285714	0.285714	0.571429	0.428571	0.428571	0.428571	0.428571	0.428571	0	0	0	0	0	0	0	0	0	0	0	0	
M00309: Non-phosphorylative_Entner-Doudoroff_pathway,_gluconate/galactonate_=>_glycerate	0	0	0	0	0	0	0	0.125	0.125	0.125	0.125	0.125	0.125	0.125	0.125	0.125	0.125	0.125	0	0	0	0	0	-1	-1	-1	-1	0	0	0	
M00314: Bacitracin_transport_system	0	0	0	0	0	0	0	0	0	0	0	0	0	0	0	0	0	0	-1	-1	-1	-1	-1	-1	-1	-1	-1	-1	-1	-1	
M00315: Uncharacterized_ABC_transport_system	0	0	0	0	0	0	0	0	0	0	0	0	0	0	0	0	0	0	-1	-1	-1	-1	-1	-1	-1	-1	-1	-1	-1	-1	
M00316: Manganese_transport_system	0	0	0	0	0	0	0	0	0	0	0	0	0	0	0	0	0	0	-1	-1	-1	-1	-1	-1	-1	-1	-1	-1	-1	-1	
M00317: Manganese/iron_transport_system	0	0	0	0	0	0	0	0	0	0	0	0	0	0	0	0	0	0	-1	-1	-1	-1	-1	-1	-1	-1	-1	-1	-1	-1	
M00318: Iron/zinc/copper_transport_system	0	0	0	0	0	0	0	0	0	0	0	0	0	0	0	0	0	0	-1	-1	-1	-1	-1	-1	-1	-1	-1	-1	-1	-1	
M00319: Manganese/zinc/iron_transport_system	0	0	0	0	0	0	0	0	0	0	0	0	0	0	0	0	0	0	-1	-1	0	-1	-1	-1	-1	-1	-1	-1	-1	-1	
M00320: Lipopolysaccharide_export_system	1	1	1	1	1	1	1	1	1	1	1	1	1	1	1	1	1	1	1	1	1	1	1	1	1	1	1	1	1	1	core
M00321: Bicarbonate_transport_system	0	0	0	0	0	0	0	0	0	0	0	0	0	0	0	0	0	0	-1	-1	-1	-1	-1	-1	-1	-1	-1	-1	-1	-1	
M00322: Neutral_amino_acid_transport_system	0	0	0	0	0	0	0	0	0	0	0	0	0	0	0	0	0	0	-1	-1	0	-1	-1	-1	-1	-1	-1	-1	-1	-1	
M00323: Urea_transport_system	1	1	1	1	1	1	1	1	0	0	1	1	0	1	1	0	1	0	0	-1	0	0	0	0	0	0	1	0	1	0	varied
M00324: Dipeptide_transport_system	0.2	0	0	0	0.2	0.2	0.2	0	0	0	0	0	0	0	0	0	0	0	-1	-1	-1	-1	-1	-1	-1	-1	0	-1	-1	-1	
M00325: alpha-Hemolysin/cyclolysin_transport_system	0.333333	0.333333	1	0.333333	0.333333	0.333333	0.333333	0.333333	0.333333	0.333333	0.333333	0.333333	0.333333	0.333333	0.333333	0.333333	0.333333	0.333333	0	0	0	0	0	0	0	0	0	0	0	0	
M00326: RTX_toxin_transport_system	0	0	0	0	0	0	0	0	0	0	0	0	0	0	0	0	0	0	-1	-1	-1	-1	-1	-1	-1	-1	-1	-1	-1	-1	
M00327: S-Layer_protein_transport_system	0	0	0	0	0	0	0	0	0	0	0	0	0	0	0	0	0	0	-1	-1	-1	-1	-1	-1	-1	-1	-1	-1	-1	-1	
M00328: Hemophore/metalloprotease_transport_system	0	0	0	0	0	0	0	0	0	0	0	0	0	0	0	0	0	0	-1	-1	-1	-1	-1	-1	-1	-1	-1	-1	-1	-1	
M00329: Multiple_protein_transport_system	0	0	0	0	0	0	0	0	0	0	0	0	0	0	0	0	0	0	-1	-1	-1	-1	-1	-1	-1	-1	-1	-1	-1	-1	
M00330: Adhesin_protein_transport_system	0	0	1	0	0	0	0	1	0	0	1	1	0	1	1	0	0	0	-1	-1	0	-1	-1	-1	-1	-1	-1	0	1	-1	varied
M00331: Type_II_general_secretion_system	0.764706	0.764706	0.764706	0.764706	0.764706	0.764706	0.764706	0.823529	0.882353	0.764706	0.823529	0.764706	0.764706	0.882353	0.882353	0.882353	0.882353	0.705882	0	0	0	0	0	0	0	0	0	0	0	0	
M00332: Type_III_secretion_system	0	0	0.611111	0	0	0	0	0	0	0	0	0	0	0	0	0	0	0	-1	-1	-1	-1	-1	-1	-1	-1	-1	-1	-1	-1	
M00333: Type_IV_secretion_system	0.833333	0.75	0.75	0.833333	0.833333	0.833333	0.833333	0.583333	0.666667	0.083333	0.083333	0.083333	0.083333	0.666667	0.666667	0.666667	0.666667	0.583333	0	0	0	0	0	0	0	0	0	0	-1	0	
M00334: Type_VI_secretion_system	0.117647	0.117647	0.764706	0.117647	0.294118	0.294118	0.705882	0.647059	0.117647	0.176471	0.764706	0.647059	0.117647	0.764706	0.588235	0	0.352941	0.058824	0	-1	0	0	0	-1	-1	-1	0	0	0	0	
M00335: Sec_(secretion)_system	0.923077	0.923077	0.846154	0.923077	0.923077	0.923077	0.923077	0.846154	0.846154	0.846154	0.846154	0.846154	0.846154	0.846154	0.846154	0.846154	0.923077	0.846154	0	0	0	0	0	1	1	0	1	0	0	0	
M00336: Twin-arginine_translocation_(Tat)_system	0.75	0.75	0.75	0.75	0.75	0.75	0.75	0.75	0.75	0.75	0.75	0.75	0.75	0.75	0.75	0.75	0.75	0.75	0	0	0	0	0	0	0	0	0	0	0	0	
M00338: Cysteine_biosynthesis,_homocysteine_+_serine_=>_cysteine	0	0	0	0	0	0	0	0	0	0	0	0	0.666667	0	0	0	0	0	0	0	0	0	-1	-1	-1	-1	-1	-1	-1	-1	
M00339: RaxAB-RaxC_type_I_secretion_system	0.333333	0.333333	0.333333	0.333333	0.333333	0.333333	0.333333	0	0	0	0.333333	0	0.333333	0	0.333333	0	0	0	0	0	0	0	0	0	0	0	0	-1	-1	-1	
M00342: Bacterial_proteasome	0	0	0	0	0	0	0	0	0	0	0	0	0	0	0	0	0	0	-1	-1	1	-1	-1	-1	-1	-1	-1	-1	-1	-1	varied
M00343: Archaeal_proteasome	0	0	0	0	0	0	0	0	0	0	0	0	0	0	0	0	0	0	-1	-1	0	-1	-1	-1	-1	-1	-1	-1	-1	-1	
M00344: Formaldehyde_assimilation,_xylulose_monophosphate_pathway	0.5	0.5	0.5	0.5	0.5	0.5	0.5	0.5	0.5	0.5	0.5	0.5	0.5	0.5	0.5	0.5	0.5	0.5	0	0	0	0	0	0	0	0	0	0	0	0	
M00345: Formaldehyde_assimilation,_ribulose_monophosphate_pathway	0.285714	0.285714	0.285714	0.142857	0.285714	0.285714	0.285714	0.285714	0.285714	0.285714	0.285714	0.285714	0.285714	0.285714	0.285714	0.285714	0.285714	0.285714	0	0	0	0	0	0	0	0	0	0	0	0	
M00346: Formaldehyde_assimilation,_serine_pathway	0.5	0.5	0.5	0.5	0.5	0.5	0.6	0.4	0.4	0.4	0.5	0.5	0.4	0.5	0.5	0.4	0.5	0.4	0	0	0	0	0	0	0	0	0	0	0	0	
M00348: Glutathione_transport_system	0	0	1	0	0	0	0	0	0	0	0	0	0	0	0	0	0	0	-1	-1	-1	-1	-1	-1	-1	-1	-1	-1	-1	-1	
M00349: Microcin_C_transport_system	1	1	1	1	1	1	1	0	1	0	0	1	1	1	1	1	1	1	0	-1	-1	-1	-1	0	-1	0	1	-1	1	1	varied
M00350: Capsaicin_biosynthesis,_L-Phenylalanine_=>_Capsaicin	0	0	0	0	0	0	0	0	0	0	0	0	0	0	0	0	0	0	-1	-1	-1	-1	-1	-1	-1	-1	-1	-1	-1	-1	
M00355: Spliceosome,_35S_U5-snRNP	0	0	0	0	0	0	0	0	0	0	0	0	0	0	0	0	0	0	-1	-1	-1	-1	-1	-1	-1	-1	-1	-1	-1	-1	
M00356: Methanogenesis,_methanol_=>_methane	0	0	0	0	0	0	0	0.222222	0	0.333333	0.333333	0	0.333333	0	0	0	0	0.333333	-1	-1	-1	-1	-1	-1	-1	-1	-1	0	-1	-1	
M00357: Methanogenesis,_acetate_=>_methane	0	0	0	0	0	0	0	0	0	0	0	0	0	0	0	0	0	0	-1	-1	-1	-1	-1	-1	-1	-1	-1	-1	-1	-1	
M00358: Coenzyme_M_biosynthesis	0	0	0.2	0	0	0	0	0	0	0	0	0	0.2	0	0	0	0	0	-1	0	0	0	0	-1	-1	-1	-1	-1	-1	-1	
M00359: Aminoacyl-tRNA_biosynthesis,_eukaryotes	0.863636	0.863636	0.863636	0.863636	0.863636	0.863636	0.863636	0.863636	0.863636	0.954545	0.954545	0.909091	0.954545	0.863636	0.863636	0.863636	0.909091	0.954545	1	1	1	1	1	0	0	0	0	1	1	0	
M00360: Aminoacyl-tRNA_biosynthesis,_prokaryotes	0.741935	0.741935	0.741935	0.741935	0.741935	0.741935	0.741935	0.677419	0.677419	0.774194	0.741935	0.741935	0.806452	0.677419	0.677419	0.677419	0.741935	0.806452	0	0	0	0	0	0	0	0	0	0	0	0	

Table S4. III

KEGG_Module	Habitat																																																																1 if median >= 90% for the samples per site; -1 if median <= 10% for the samples per site												Kind of Module inferred (Core or Varied)
	India_oil_refineries				Arctic												China_oil_refineries									Mangrove			Marine_sediments							Taiga_upper_active_layer				Taiga_bottom_active_layer				Taiga_permafrost_layer				Oil_sands_core							Oil_sands_tailings_pond_upper				Oil_sands_tailings_pond_median			Oil_sands_tailings_pond_deep																
	I1	I2	I3	I4	A1	A10	A11	A12	A2	A3	A4	A5	A6	A7	A8	A9	C1	C2	C3	C4	C5	C6	C7	C8	C9	M1	M2	M3	DWH1	DWH2	DWH3	DWH4	DWH5	DWH6	DWH7	Tu1	Tu2	Tu3	Tu4	Tb1	Tb2	Tb3	Tb4	Tp1	Tp2	Tp3	Tp4	OSC1	OSC12	OSC3	OSC4	OSC5	OSC7	OSC9	OSTPu1	OSTPu2	OSTPu4	OSTPu3	OSTPm2	OSTPm6	OSTPm4	OSTPd1	OSTPd2	OSTPd3	OSTPd4	India_oil_refineries	Arctic	China_oil_refineries	Mangrove	Marine_sediments	Taiga_upper_active_layer	Taiga_bottom_active_layer	Taiga_permafrost_layer	Oil_sands_core	Oil_sands_tailings_pond_upper	Oil_sands_tailings_pond_median	Oil_sands_tailings_pond_deep	
M00361: Nucleotide_sugar_biosynthesis,_eukaryotes	0.714286	0.571429	0.571429	0.571429	0.571429	0.571429	0.571429	0.571429	0.571429	0.714286	0.714286	0.571429	0.571429	0.571429	0.571429	0.571429	0.857143	0.857143	0.857143	0.857143	0.857143	0.857143	0.857143	0.857143	0.857143	0.857143	0.857143	0.857143	0.857143	0.714286	0.714286	0.714286	0.571429	0.714286	0.714286	0.714286	0.714286	0.714286	0.714286	0.714286	0.714286	0.714286	0.714286	0.714286	0.714286	0.714286	0.714286	0.714286	0.714286	0.714286	0.714286	0.714286	0.714286	0.714286	0.857143	0.857143	0.714286	0.714286	0.857143	0.857143	0.571429	0.714286	0.571429	0.857143	0.857143	0	0	0	0	0	0	0	0	0	0	0	0	
M00362: Nucleotide_sugar_biosynthesis,_prokaryotes	0.615385	0.615385	0.615385	0.615385	0.538462	0.615385	0.538462	0.538462	0.538462	0.615385	0.615385	0.538462	0.538462	0.538462	0.538462	0.538462	0.769231	0.769231	0.769231	0.769231	0.769231	0.692308	0.769231	0.769231	0.769231	0.692308	0.692308	0.769231	0.692308	0.538462	0.538462	0.538462	0.538462	0.615385	0.538462	0.615385	0.615385	0.615385	0.538462	0.538462	0.615385	0.615385	0.615385	0.615385	0.615385	0.538462	0.615385	0.538462	0.538462	0.538462	0.538462	0.538462	0.538462	0.615385	0.615385	0.615385	0.769231	0.692308	0.692308	0.846154	0.615385	0.538462	0.538462	0.615385	0.692308	0	0	0	0	0	0	0	0	0	0	0	0	
M00364: C10-C20_isoprenoid_biosynthesis,_bacteria	0.5	0.5	0.75	0.5	1	0.5	1	1	0.25	0.5	0.5	0.5	0.75	0.75	0.5	0.5	1	0.75	0.75	0.75	1	1	0.75	0.75	1	0.75	0.75	0.75	0.5	0.5	0.5	0.5	0.5	0.5	0.5	0.5	0.25	0.75	0.5	0.25	0.5	1	0.5	0.5	0.25	0.75	0.25	0.25	0.25	0.25	0.25	0.25	0.25	0.25	0.25	0.25	0.25	0.5	0.5	0.75	0.25	0.25	0.25	0.5	0.5	0	0	0	0	0	0	0	0	0	0	0	0	
M00365: C10-C20_isoprenoid_biosynthesis,_archaea	0	0	0.5	0	1	0	1	1	0	0.5	0	0	1	1	0	0	1	1	1	1	1	1	1	1	1	0.5	0.5	0.5	0	0	0	0	0	0	0	0	0	1	0	0	0	1	0	0	0	1	0	0	0	0	0	0	0	0	0	0	0	0	0	0.5	0	0	0	0	0	-1	0	1	0	-1	-1	-1	-1	-1	-1	-1	-1	varied
M00366: C10-C20_isoprenoid_biosynthesis,_plants	0.25	0.25	0.5	0.25	0.5	0.25	0.5	0.5	0.25	0.25	0.25	0.25	0.5	0.5	0.25	0.25	0.5	0.5	0.5	0.5	0.5	0.5	0.5	0.5	0.5	0.5	0.5	0.5	0.25	0.25	0.25	0.25	0.25	0.25	0.25	0.25	0	0.5	0.25	0	0.25	0.5	0.25	0.25	0	0.5	0	0	0	0	0	0	0	0	0	0	0.25	0.25	0.25	0.5	0	0	0	0.25	0.25	0	0	0	0	0	0	0	0	-1	0	0	0	
M00368: Ethylene_biosynthesis,_methionine_=>_ethylene	0	0	0	0	0	0	0	0	0	0	0	0	0	0	0	0	0	0	0.333333	0	0	0	0	0	0	0	0	0	0	0	0	0	0	0	0	0	0	0	0	0	0	0	0	0	0	0	0	0	0	0	0	0	0	0	0	0	0	0	0	0	0	0	0	0	0	-1	-1	-1	-1	-1	-1	-1	-1	-1	-1	-1	-1	
M00372: Abscisic_acid_biosynthesis,_beta-carotene_=>_abscisic_acid	0	0	0	0	0	0	0	0	0	0	0	0	0	0	0	0	0	0	0	0	0	0	0	0	0	0	0	0	0	0	0	0	0	0	0	0.142857	0.142857	0	0	0	0.142857	0	0.142857	0.142857	0.142857	0	0.142857	0	0	0	0	0	0	0	0	0	0	0	0	0	0	0	0	0	0	-1	-1	-1	-1	-1	-1	-1	0	-1	-1	-1	-1	
M00373: Ethylmalonyl_pathway	0.384615	0.461538	0.384615	0.461538	0.384615	0.461538	0.461538	0.384615	0.307692	0.307692	0.384615	0.384615	0.230769	0.230769	0.384615	0.307692	0.615385	0.384615	0.461538	0.307692	0.384615	0.384615	0.384615	0.307692	0.384615	0.384615	0.384615	0.384615	0.384615	0.384615	0.307692	0.384615	0.230769	0.384615	0.384615	0.538462	0.538462	0.538462	0.923077	0.615385	0.461538	0.461538	0.615385	0.461538	0.461538	0.307692	0.615385	0.384615	0.384615	0.384615	0.384615	0.384615	0.384615	0.461538	0.384615	0.384615	0.384615	0.461538	0.461538	0.384615	0.384615	0.384615	0.384615	0.461538	0.384615	0	0	0	0	0	0	0	0	0	0	0	0	
M00374: Dicarboxylate-hydroxybutyrate_cycle	0.416667	0.416667	0.416667	0.416667	0.416667	0.416667	0.416667	0.416667	0.375	0.5	0.458333	0.416667	0.375	0.375	0.416667	0.375	0.416667	0.458333	0.416667	0.625	0.541667	0.416667	0.5	0.416667	0.458333	0.666667	0.541667	0.666667	0.541667	0.416667	0.541667	0.416667	0.416667	0.375	0.416667	0.416667	0.375	0.416667	0.333333	0.375	0.416667	0.375	0.375	0.416667	0.416667	0.375	0.375	0.416667	0.416667	0.416667	0.416667	0.416667	0.416667	0.416667	0.416667	0.416667	0.708333	0.5	0.541667	0.708333	0.416667	0.416667	0.416667	0.416667	0.666667	0	0	0	0	0	0	0	0	0	0	0	0	
M00375: Hydroxypropionate-hydroxybutylate_cycle	0	0	0	0	0	0.210526	0.105263	0	0.210526	0	0	0.210526	0.210526	0	0	0.210526	0	0	0	0	0	0	0	0	0	0	0	0	0	0	0	0	0	0	0	0	0.105263	0	0	0	0	0	0.105263	0	0	0	0	0	0.052632	0	0	0	0	0.105263	0	0	0	0	0	0	0	0	0	0	0	-1	-1	-1	-1	-1	-1	-1	-1	-1	-1	-1	-1	
M00376: 3-Hydroxypropionate_bi-cycle	0.571429	0.571429	0.571429	0.571429	0.571429	0.571429	0.47619	0.52381	0.571429	0.571429	0.571429	0.571429	0.52381	0.52381	0.571429	0.52381	1	0.571429	0.619048	0.571429	0.571429	0.571429	0.571429	0.571429	0.571429	0.571429	0.571429	0.571429	0.571429	0.47619	0.52381	0.47619	0.428571	0.47619	0.47619	0.619048	0.52381	0.571429	0.571429	0.52381	0.571429	0.571429	0.571429	0.666667	0.571429	0.571429	0.571429	0.47619	0.47619	0.47619	0.47619	0.47619	0.47619	0.52381	0.428571	0.47619	0.571429	0.571429	0.47619	0.619048	0.47619	0.47619	0.47619	0.52381	0.571429	0	0	0	0	0	0	0	0	0	0	0	0	
M00377: Reductive_acetyl-CoA_pathway_(Wood-Ljungdahl_pathway)	0.3	0.2	0.2	0.2	0.6	0.6	0.2	0.5	0.6	0.6	0.6	0.3	0.6	0.5	0.6	0.3	0.4	0.4	0.4	0.5	0.5	0.4	0.4	0.4	0.4	0.6	0.6	0.6	0.6	0.5	0.6	0.6	0.6	0.3	0.3	0.3	0.2	0.3	0.3	0.2	0.2	0.4	0.2	0.3	0.2	0.4	0.2	0.2	0.2	0.2	0.2	0.2	0.2	0.2	0.2	0.2	0.9	0.5	0.5	0.9	0.3	0.2	0.2	0.2	0.9	0	0	0	0	0	0	0	0	0	0	0	0	
M00378: F420_biosynthesis	0	0	0	0	0.5	0	0.5	0.5	0	0	0	0	0.5	0.5	0	0	0.5	0.5	0.5	0.5	0.5	0.5	0.5	0	0.5	0	0	0	0	0	0	0	0	0	0	0	0	0.5	0	0	0	0.5	0	0	0	0.5	0	0	0	0	0	0	0	0	0	0	0	0	0	0	0	0	0	0	0	-1	-1	0	-1	-1	-1	-1	-1	-1	-1	-1	-1	
M00391: Exosome,_eukaryotes	0	0	0	0	0	0	0	0	0	0	0	0	0	0	0	0	0	0	0	0	0	0	0	0	0	0	0	0	0	0	0	0	0	0	0	0	0	0	0	0	0	0	0	0	0	0	0	0	0	0	0	0	0	0	0	0	0	0	0	0	0	0	0	0	0	-1	-1	-1	-1	-1	-1	-1	-1	-1	-1	-1	-1	
M00392: Ski_complex	0	0	0	0	0	0	0	0	0	0	0	0	0	0	0	0	0	0	0	0	0	0	0	0	0	0	0	0	0	0	0	0	0	0	0	0	0	0	0	0	0	0	0	0	0	0	0	0	0	0	0	0	0	0	0	0	0	0	0	0	0	0	0	0	0	-1	-1	-1	-1	-1	-1	-1	-1	-1	-1	-1	-1	
M00394: RNA_degradosome	0.75	0.75	0.75	0.75	0.75	0.75	0.75	0.75	0.75	0.75	0.75	0.75	0.75	0.75	0.75	0.75	0.75	0.75	0.75	0.75	0.75	1	0.75	0.75	0.75	1	1	1	0.75	1	1	1	1	0.75	1	0.75	0.75	0.75	0.75	0.75	0.75	0.75	0.75	0.75	0.75	0.75	0.75	1	1	0.75	1	1	1	1	0.75	0.75	0.75	0.75	1	1	0.75	0.75	0.75	1	1	0	0	0	1	1	0	0	0	1	0	1	0	
M00400: p97-Ufd1-Npl4_complex	0	0	0	0	0	0	0	0	0	0	0	0	0	0	0	0	0	0	0.333333	0	0	0	0	0	0	0	0	0	0	0	0	0	0	0	0	0.333333	0.333333	0.333333	0	0.333333	0.333333	0	0.333333	0.333333	0.333333	0	0.333333	0	0	0	0	0	0	0	0	0	0	0	0	0	0	0	0	0	0	-1	-1	-1	-1	-1	0	0	0	-1	-1	-1	-1	
M00403: HRD1/SEL1_ERAD_complex	0	0	0	0	0	0	0	0	0	0	0	0	0	0	0	0	0	0	0	0	0	0	0	0	0	0	0	0	0	0	0	0	0	0	0	0	0	0	0	0	0	0	0	0	0	0	0	0	0	0	0	0	0	0	0	0	0	0	0	0	0	0	0	0	0	-1	-1	-1	-1	-1	-1	-1	-1	-1	-1	-1	-1	
M00404: COPII_complex	0	0	0	0	0	0	0	0	0	0	0	0	0	0	0	0	0	0	0	0	0	0	0	0	0	0	0	0	0	0	0	0	0	0	0	0	0	0	0	0	0	0	0	0	0	0	0	0	0	0	0	0	0	0	0	0	0	0	0	0	0	0	0	0	0	-1	-1	-1	-1	-1	-1	-1	-1	-1	-1	-1	-1	
M00413: FA_core_complex	0	0	0	0	0	0	0	0	0	0	0	0	0	0	0	0	0	0	0	0	0	0	0	0	0	0	0	0	0	0	0	0	0	0	0	0	0	0	0	0	0	0	0	0	0	0	0	0	0	0	0	0	0	0	0	0	0	0	0	0	0	0	0	0	0	-1	-1	-1	-1	-1	-1	-1	-1	-1	-1	-1	-1	
M00416: Cytochrome_aa3-600_menaquinol_oxidase	0	0	0	0	0	0	0	0	0	0	0	0	0	0	0	0	0	0	0	0	0	0	0	0	0	0	0	0	0	0	0	0	0	0	0	0	0	0	0	0	0	0	0	0	0	0	0	0	0	0	0	0	0	0	0	0	0	0	0	0	0	0	0	0	0	-1	-1	-1	-1	-1	-1	-1	-1	-1	-1	-1	-1	
M00417: Cytochrome_o_ubiquinol_oxidase	1	1	0.25	1	1	1	1	1	0.25	0	0	0.25	0	0	0.25	1	0	0.25	0	0.25	0	0.25	0.25	0.25	0.25	0	0	0	1	0	0	0.25	0	0	0.5	1	0.75	0	0.25	1	1	0.5	1	1	0.75	0	1	1	1	1	1	1	1	1	0.25	1	0	0.25	0	0	1	1	1	1	0	1	0	0	-1	-1	0	1	0	1	0	-1	1	varied
M00418: Toluene_degradation,_anaerobic,_toluene_=>_benzoyl-CoA	0	0	0	0	0	0	0	0	0	0	0	0	0.111111	0.111111	0	0	0	0	0	0	0	0	0	0	0	0	0	0	0	0	0	0	0	0	0	0	0	0	0	0	0	0	0	0	0	0	0	0	0	0	0	0	0	0	0	0	0	0	0	0	0	0	0	0	0	-1	-1	-1	-1	-1	-1	-1	-1	-1	-1	-1	-1	
M00423: Molybdate/tungstate_transport_system	0	0	0	0	0	0	0	0	0	0	0	0	0	0	0	0	0	0	0	0.333333	0	0	0	0	0	0	0	0	0	0	0	0	0	0	0	0	0	0	0	0	0	0	0	0	0	0	0	0	0	0	0	0	0	0	0	0	0	0	0	0	0	0	0	0	0	-1	-1	-1	-1	-1	-1	-1	-1	-1	-1	-1	-1	
M00427: Nuclear_pore_complex	0	0	0	0	0	0	0	0	0	0	0	0	0	0	0	0	0	0	0	0	0	0	0	0	0	0	0	0	0	0	0	0	0	0	0	0	0	0	0	0	0	0	0	0	0	0	0	0	0	0	0	0	0	0	0	0	0	0	0	0	0	0	0	0	0	-1	-1	-1	-1	-1	-1	-1	-1	-1	-1	-1	-1	
M00428: eIF4F_complex	0	0	0	0	0	0	0	0	0	0	0	0	0	0	0	0	0	0	0	0	0	0	0	0	0	0	0	0	0	0	0	0	0	0	0	0	0	0	0	0	0	0	0	0	0	0	0	0	0	0	0	0	0	0	0	0	0	0	0	0	0	0	0	0	0	-1	-1	-1	-1	-1	-1	-1	-1	-1	-1	-1	-1	
M00429: Competence-related_DNA_transformation_transporter	0.133333	0.133333	0.2	0.2	0.2	0.2	0.133333	0.2	0.133333	0.2	0.2	0.133333	0.133333	0.133333	0.2	0.133333	0.2	0.2	0.2	0.2	0.2	0.2	0.2	0.2	0.2	0.2	0.2	0.2	0.2	0.2	0.2	0.2	0.2	0.133333	0.133333	0.133333	0.066667	0.133333	0.066667	0.066667	0.133333	0.133333	0.066667	0.133333	0.133333	0.133333	0.066667	0.133333	0.133333	0.133333	0.133333	0.133333	0.133333	0.133333	0.133333	0.133333	0.2	0.2	0.2	0.2	0.133333	0.133333	0.133333	0.2	0.2	0	0	0	0	0	0	0	0	0	0	0	0	
M00432: Leucine_biosynthesis,_2-oxoisovalerate_=>_2-oxoisocaproate	0.8	0.8	0.8	0.8	0.8	0.8	0.8	0.8	0.8	0.8	0.8	0.8	0.8	0.8	0.8	0.8	0.8	0.8	0.8	0.8	0.8	0.8	0.8	0.8	0.8	0.8	0.8	0.8	0.8	0.8	0.8	0.8	0.8	0.8	0.8	1	0.8	0.8	0.8	1	0.8	0.8	1	0.8	0.8	0.8	1	0.8	0.8	0.8	0.8	0.8	0.8	0.8	0.8	0.8	0.8	0.8	0.8	0.8	0.8	0.8	0.8	0.8	0.8	0	0	0	0	0	0	0	0	0	0	0	0	
M00433: Lysine_biosynthesis,_2-oxoglutarate_=>_2-oxoadipate	0	0	0	0	0	0	0	0	0	0	0	0	0	0	0	0	0.666667	0	0	0	0	0	0	0	0	0	0	0	0	0	0	0	0	0	0	0	0	0	0	0	0	0	0	0	0	0	0	0	0	0	0	0	0	0	0	0	0	0	0	0	0	0	0	0	0	-1	-1	-1	-1	-1	-1	-1	-1	-1	-1	-1	-1	
M00434: PhoR-PhoB_(phosphate_starvation_response)_two-component_regulatory_system	1	1	1	1	1	1	0.666667	1	1	1	1	1	1	0.666667	1	1	1	1	1	1	1	1	1	1	1	1	1	1	1	1	1	1	1	1	1	1	0.666667	1	0.666667	0.666667	1	1	0.666667	1	0.666667	1	0.666667	0.666667	0.666667	0.666667	0.666667	0.666667	0.666667	0.666667	1	0.666667	1	1	1	1	1	0.666667	0.666667	1	1	1	1	1	1	1	0	0	0	0	1	1	0	
M00435: Taurine_transport_system	0	0	0	0	0	0	0	0	0	0	0	0	0	0	0	0	0	0	0	0	0	0	0	0	0	0	0	0	0	0	0	0	0	0	0	0	0	0	0	0	0	0	0	0	0	0	0	0	0	0	0	0	0	0	0	0	0	0	0	0	0	0	0	0	0	-1	-1	-1	-1	-1	-1	-1	-1	-1	-1	-1	-1	
M00436: Sulfonate_transport_system	0.333333	0.333333	0	0.333333	0	0.333333	0.333333	0	0	0	0	0	0	0	0	0	1	1	1	1	1	1	1	0.333333	1	0.666667	0.666667	0.666667	0.333333	0.333333	0.333333	0.333333	0.333333	0.666667	0.333333	1	0.333333	1	1	1	1	1	0.333333	1	1	1	0.333333	1	1	1	1	1	1	1	0	0	1	1	0	0.666667	1	0	0	1	0.666667	0	-1	1	0	0	1	1	1	1	0	0	0	varied
M00438: Nitrate/nitrite_transport_system	0.25	0.75	0.75	0.75	0	0.75	0.25	0	0	0	0	0	0	0	0.25	0	0	0.5	0	0.5	0.75	0.25	0.5	0.5	0.75	0.5	0.25	0.5	0.25	0.25	0.25	0.25	0.25	0.5	0.25	0.75	0.75	0	0.25	0.75	0.75	0.5	0.75	0.75	0.75	0	0.75	0.75	0.75	0.75	0.75	0.75	0.75	0.75	0.75	0.75	0	0.75	0.75	0	0.75	0.75	0.75	0.75	0	0	-1	0	0	0	0	0	0	0	0	0	0	
M00439: Oligopeptide_transport_system	0.2	0	0	0	0.8	0.2	0.8	0.8	0.2	0.4	0.8	0.2	0.8	0.8	0.2	0	1	1	1	1	1	1	1	1	1	1	1	1	0.4	0.4	0.4	1	0.8	1	0.8	0.8	0	1	0	0.4	0.2	1	0	0.8	0	1	0	0.8	0.8	0.8	0.8	0.8	0.8	0.8	0.2	0	0.6	0.6	0.2	0.8	0.2	0	0	0.4	0.6	-1	0	1	1	0	0	0	0	0	0	0	0	varied
M00440: Nickel_transport_system	0	0	0	0	0	0	0	0	0	0	0	0	0	0	0	0	0	0	0	0	0	0	0	0	0	0	0	0	0	0	0	0	0	0	0	0	0	0	0	0	0	0	0	0	0	0	0	0	0	0	0	0	0	0	0	0	0	0	0	0	0	0	0	0	0	-1	-1	-1	-1	-1	-1	-1	-1	-1	-1	-1	-1	
M00442: Putative_hydroxymethylpyrimidine_transport_system	0	0	0	0	0	0	0	0	0	0	0	0	0	0	0	0	0.333333	0	0	0	0	0	0	0	0	0	0	0	0	0	0	0	0	0	0	0	0	0	0	0	0	0	0	0	0	0	0	0	0	0	0	0	0	0	0	0	0	0	0	0	0	0	0	0	0	-1	-1	-1	-1	-1	-1	-1	-1	-1	-1	-1	-1	
M00443: SenX3-RegX3_(phosphate_starvation_response)_two-component_regulatory_system	0	0	0	0	1	0	1	1	0	0	0	0	1	1	0	0	1	1	1	0	0.5	1	1	0	0.5	0	0	0	0	0	0	0	0	0	0	0	0	1	0	0	0	0.5	0	0	0	1	0	0	0	0	0	0	0	0	0	0	0	0	0	0	0	0	0	0	0	-1	-1	1	-1	-1	-1	-1	-1	-1	-1	-1	-1	varied
M00444: PhoQ-PhoP_(magnesium_transport)_two-component_regulatory_system	0.5	0.5	0	0	0	0	0	0	0	0	0	0	0	0	0	0.5	0	0	0	0	0	0	0	0	0	0	0	0	0	0	0	0	0	0	0	0	0	0	0	0	0	0	0	0	0	0	0	0	0	0	0	0	0	0	0	0	0	0	0	0	0	0	0	0	0	0	-1	-1	-1	-1	-1	-1	-1	-1	-1	-1	-1	
M00445: EnvZ-OmpR_(osmotic_stress_response)_two-component_regulatory_system	1	1	1	1	1	1	1	1	0	0	1	0.5	0	0	1	1	0.5	0	0	0	0	1	0	0	1	0.5	1	0.5	1	1	1	1	1	1	1	1	1	0	1	1	1	1	1	1	1	0	1	1	1	1	1	1	1	1	1	1	0.5	1	1	1	1	1	1	1	1	1	1	-1	0	1	1	1	1	1	1	1	1	varied
M00446: RstB-RstA_two-component_regulatory_system	0	0	0	0	0	0	0	0	0	0	0	0	0	0	0	0	0	0	0	0	0	1	0	0	0	0	0	0	0	0	0	0.5	0.5	0	0	0	0	0	0	0	0	0	0	0	0	0	0	1	1	0	1	1	1	1	0	0	0	0	0.5	0	0	0	0	0.5	0	-1	-1	-1	-1	-1	-1	-1	-1	1	-1	-1	-1	varied
M00447: CpxA-CpxR_(envelope_stress_response)_two-component_regulatory_system	1	1	0	0	0	0	0	0	1	0	0	0	0	0	0.5	0.5	0	0.5	0.5	1	1	0.5	0.5	1	0.5	0	0	0	0	0	0	0	0	0	0	0	0	0	0	0	0	0.5	0	0	0	0	0	0	0	0	0	0	0	0	0	0	0	0	0	0	0	0	0	0	0	0	-1	0	-1	-1	-1	-1	-1	-1	-1	-1	-1	
M00448: CssS-CssR_(secretion_stress_response)_two-component_regulatory_system	0	0	0	0	0	0	0	0	0	0	0	0	0	0	0	0	0	0	0	0	0	0	0	0	0	0	0	0	0	0	0	0	0	0	0	0	0	0	0	0	0	0	0	0	0	0	0	0	0	0	0	0	0	0	0	0	0	0	0	0	0	0	0	0	0	-1	-1	-1	-1	-1	-1	-1	-1	-1	-1	-1	-1	
M00449: CreC-CreB_(phosphate_regulation)_two-component_regulatory_system	0	0	0	0	0	0	0	0	0	0	0	0	0	0	0	0	0	0	0	0	0	0	0	0	0	0	0	0	0	0	0	0	0	0	0	0	0	0	0	0	0	0	0	0	0	0	0	0	0	0	0	0	0	0	1	0	0	1	1	0	1	0	0	0	0	-1	-1	-1	-1	-1	-1	-1	-1	-1	0	1	-1	varied
M00450: BaeS-BaeR_(envelope_stress_response)_two-component_regulatory_system	0	0	0.5	0	0	0	0	0	0	0	0	0	0	0	0	0	0.5	0.5	0.5	0	0	0	0	0	0	0	0	0	0	0	0	0	0	0	0	0	0	0	0	0	0	0	0	0	0	0	0	0	0	1	0	0	0	0.5	0	1	0	0	0.5	0.5	1	1	1	1	0.5	-1	-1	-1	-1	-1	-1	-1	-1	-1	-1	0	1	varied
M00451: BasS-BasR_(antimicrobial_peptide_resistance)_two-component_regulatory_system	0	0	0	0	0	0	0	0	0	0	0	0	0	0	0	0	0	0	0	0	0	0	0	0	0	0	0	0	0	0	0	0	0	0	0	0	0	0	0	0	0	0	0	0	0	0	0	0	0	0	0	0	0	0	0	0	0	0	0	0	0	0	0	0	0	-1	-1	-1	-1	-1	-1	-1	-1	-1	-1	-1	-1	
M00452: CusS-CusR_(copper_tolerance)_two-component_regulatory_system	1	1	1	1	0	0.5	0.5	0	0.5	0	0	1	0	0	0.5	1	0.5	1	0.5	1	1	1	1	1	1	0.5	0.5	0.5	1	1	1	1	1	1	1	1	0	0.5	0.5	0	1	0.5	0.5	1	1	0.5	1	1	1	1	1	1	1	1	1	1	1	1	1	1	1	1	1	1	1	1	0	1	0	1	0	0	1	1	1	1	1	
M00453: QseC-QseB_(quorum_sensing)_two-component_regulatory_system	0	0.5	0	1	0	0	0	0	0	0	0	0	0	0	0	0	0	0.5	0	1	0	1	0	1	1	0	0	0	1	1	1	1	1	1	1	0	0	0	0	0	0	0	0	0	1	0	0	1	1	0.5	1	1	1	1	1	1	0.5	1	1	0	1	1	0.5	1	0	0	-1	0	-1	1	-1	-1	-1	1	1	1	0	varied
M00454: KdpD-KdpE_(potassium_transport)_two-component_regulatory_system	1	1	1	1	1	1	1	1	1	1	1	1	1	1	1	1	1	1	1	1	1	1	1	1	1	1	1	1	1	0	0.5	1	0	0.5	1	1	1	1	1	1	1	1	1	1	1	1	1	1	1	1	1	1	1	1	1	1	1	1	1	1	1	1	1	1	1	1	1	1	1	0	1	1	1	1	1	1	1	
M00455: TorS-TorR_(TMAO_respiration)_two-component_regulatory_system	0	0	0	0	0	0	0	0	0	0	0	0	0	0	0	0	0	0	0	0	0	0	0	0	0	0	0	0	0	0	0	0	0	0	0	0	0	0	0	0	0	0	0	0	0	0	0	0	0	0	0	0	0	0	0	0	0	0	0	0	0	0	0	0	0	-1	-1	-1	-1	-1	-1	-1	-1	-1	-1	-1	-1	
M00456: ArcB-ArcA_(anoxic_redox_control)_two-component_regulatory_system	0	0	0	0	0	0	0	0	0	0	0	0	0	0	0	0	0	0	0	0	0	0	0	0	0	0	0	0	0	0	0	0	0	0	0	0	0	0	0	0	0	0	0	0	0	0	0	0	0	0	0	0	0	0	0	0	0	0	0	0	0	0	0	0	0	-1	-1	-1	-1	-1	-1	-1	-1	-1	-1	-1	-1	
M00457: TctE-TctD_(tricarboxylic_acid_transport)_two-component_regulatory_system	0.5	0.5	0.5	0.5	0.5	0.5	0.5	0.5	0	0	0	0	0	0	0.5	0.5	0	0	0	0	0	0.5	0.5	0	0.5	0	0	0	0	0	0	0	0	0	0	0.5	0	0	0	0	0.5	0	0	0.5	1	0	1	1	1	1	1	1	1	1	1	1	0.5	1	1	0.5	1	1	1	1	0	0	0	-1	-1	-1	-1	-1	0	1	1	1	1	varied
M00458: ResE-ResD_(aerobic_and_anaerobic_respiration)_two-component_regulatory_system	0	0	0	0	0	0	0	0	0	0	0	0	0	0	0	0	0	0	0	0	0	0	0	0	0	0	0	0	0	0	0	0	0	0	0	0	0	0	0	0	0	0	0	0	0	0	0	0	0	0	0	0	0	0	0	0	0	0	0	0	0	0	0	0	0	-1	-1	-1	-1	-1	-1	-1	-1	-1	-1	-1	-1	
M00459: VicK-VicR_(cell_wall_metabolism)_two-component_regulatory_system	0	0	0	0	0	0	0	0	0	0	0	0	0	0	0	0	0.5	0.5	0.5	0.5	0.5	0	0.5	0	0.5	0	0	0	0	0	0	0	0	0	0	0	0	0	0	0	0	0.5	0	0	0	0.5	0	0	0	0	0	0	0	0	0	0	0	0	0	0	0	0	0	0	0	-1	-1	0	-1	-1	-1	-1	-1	-1	-1	-1	-1	
M00460: MprB-MprA_(maintenance_of_persistent_infection)_two-component_regulatory_system	0	0.5	0	0	0	0	0	0	0	0	0	0	0	0	0	0	0.5	0.5	1	0.5	0.5	0.5	0.5	0.5	0.5	0.5	0.5	0.5	0	0	0	0	0	0.5	0	0	0	1	0	0	0	0.5	0	0	0	0.5	0	0	0	0	0	0	0	0	0	0	0	0	0	0	0	0	0	0	0	-1	-1	0	0	-1	-1	-1	-1	-1	-1	-1	-1	
M00461: MtrB-MtrA_(osmotic_stress_response)_two-component_regulatory_system	0	0	0	0	1	0	1	0	0	0	0	0	1	1	0	0	1	0.5	1	0.5	0.5	1	0.5	0	0.5	0	0	0	0	0	0	0	0	0	0	0	0	1	0	0	0	0.5	0	0	0	1	0	0	0	0	0	0	0	0	0	0	0	0	0	0	0	0	0	0	0	-1	-1	0	-1	-1	-1	-1	-1	-1	-1	-1	-1	
M00462: PrrB-PrrA_(intracellular_multiplication)_two-component_regulatory_system	0	0	0	0	0	0	0	0	0	0	0	0	0	0	0	0	0	0	0	0	0	0	0	0	0	0	0	0	0	0	0	0	0	0	0	0	0	0	0	0	0	0	0	0	0	0	0	0	0	0	0	0	0	0	0	0	0	0	0	0	0	0	0	0	0	-1	-1	-1	-1	-1	-1	-1	-1	-1	-1	-1	-1	
M00464: NrsS-NrsR_(nickel_tolerance)_two-component_regulatory_system	0	0	0	0	0	0	0	0	0	0	0	0	0	0	0	0	0	0	0	0	0	0	0	0	0	0	0	0	0	0	0	0	0	0	0	0	0	0	0	0	0	0	0	0	0	0	0	0	0	0	0	0	0	0	0	0	0	0	0	0	0	0	0	0	0	-1	-1	-1	-1	-1	-1	-1	-1	-1	-1	-1	-1	
M00465: ManS-ManR_(manganese_homeostasis)_two-component_regulatory_system	0	0	0	0	0	0	0	0	0	0	0	0	0	0	0	0	0	0	0	0	0	0	0	0	0	0	0	0	0	0	0	0	0	0	0	0	0	0	0	0	0	0	0	0	0	0	0	0	0	0	0	0	0	0	0	0	0	0	0	0	0	0	0	0	0	-1	-1	-1	-1	-1	-1	-1	-1	-1	-1	-1	-1	
M00466: NblS-NblR_(photosynthesis)_two-component_regulatory_system	0	0	0	0	0	0	0	0	0	0	0	0	0	0	0	0	0	0	0	0	0	0	0	0	0	0	0	0	0	0	0	0	0	0	0	0	0	0	0	0	0	0	0	0	0	0	0	0	0	0	0	0	0	0	0	0	0	0	0	0	0	0	0	0	0	-1	-1	-1	-1	-1	-1	-1	-1	-1	-1	-1	-1	
M00467: SasA-RpaAB_(circadian_timing_mediating)_two-component_regulatory_system	0	0	0	0	0	0	0	0	0	0	0	0	0	0	0	0	0.333333	0	0	0	0	0	0	0	0	0	0	0	0	0	0	0	0	0	0	0	0	0	0	0	0	0	0	0	0	0	0	0	0	0	0	0	0	0	0	0	0	0	0	0	0	0	0	0	0	-1	-1	-1	-1	-1	-1	-1	-1	-1	-1	-1	-1	
M00468: SaeS-SaeR_(staphylococcal_virulence_regulation)_two-component_regulatory_system	0	0	0	0	0	0	0	0	0	0	0	0	0	0	0	0	0	0	0	0	0	0	0	0	0	0	0	0	0	0	0	0	0	0	0	0	0	0	0	0	0	0	0	0	0	0	0	0	0	0	0	0	0	0	0	0	0	0	0	0	0	0	0	0	0	-1	-1	-1	-1	-1	-1	-1	-1	-1	-1	-1	-1	
M00469: BceS-BceR_(bacitracin_transport)_two-component_regulatory_system	0	0	0	0	0	0	0	0	0	0	0	0	0	0	0	0	0	0	0	0	0	0	0	0	0	0	0	0	0	0	0	0	0	0	0	0	0	0	0	0	0	0	0	0	0	0	0	0	0	0	0	0	0	0	0	0	0	0	0	0	0	0	0	0	0	-1	-1	-1	-1	-1	-1	-1	-1	-1	-1	-1	-1	
M00471: NarX-NarL_(nitrate_respiration)_two-component_regulatory_system	0	1	1	1	0	0	0	0	0	0	0	0	0	0	0	0	0	0	0.5	0	0	0	0	0	0	0	0	0	0	0	0	0	0	0	0	0	0	0	0	0	0	0	0	0	0.5	0	0	0.5	1	1	0.5	0.5	1	1	1	1	0	1	0.5	0	1	1	1	1	0	1	-1	-1	-1	-1	-1	-1	-1	1	1	0	1	varied
M00472: NarQ-NarP_(nitrate_respiration)_two-component_regulatory_system	0	0	0	0	0	0	0	0	0	0	0	0	0	0	0	0	0	0	0	0	0	0	0	0	0	0	0	0	0	0	0	0	0	0	0	0	0	0	0	0	0	0	0	0	0	0	0	0	0	0	0	0	0	0	0	0	0	0	0	0	0	0	0	0	0	-1	-1	-1	-1	-1	-1	-1	-1	-1	-1	-1	-1	
M00473: UhpB-UhpA_(hexose_phosphates_uptake)_two-component_regulatory_system	0.5	0.5	0	0.5	0	0.5	0	0	0	0	0	0	0	0	0	0	0	0	0	0	0	0	0	0	0	0	0	0	0	0	0	0	0	0	0	0	0	0	0	0	0	0	0	0	0	0	0	0	0	0	0	0	0	0	0.5	0	0	0.5	0.5	0	0.5	0.5	0	0	0	0	-1	-1	-1	-1	-1	-1	-1	-1	0	0	-1	
M00474: RcsC-RcsD-RcsB_(capsule_synthesis)_two-component_regulatory_system	0	0	0	0	0	0	0	0	0	0	0	0	0	0	0	0	0	0	0	0	0	0	0	0	0	0	0	0	0	0	0	0	0	0	0	0	0	0	0	0	0	0	0	0	0	0	0	0.666667	0.666667	0.666667	0.666667	0.333333	0.666667	0.666667	0	0	0	0	0	0	0	0	0	0	0	-1	-1	-1	-1	-1	-1	-1	-1	0	-1	-1	-1	
M00475: BarA-UvrY_(central_carbon_metabolism)_two-component_regulatory_system	0	0	0	0.5	0	0	0	0	0	0	0	0	0	0	0	0	0	0	0	0	0	0	0	0	0	0	0	0	0	0	0	0	0	0	0	0	0	0	0	0	0	0	0	0	0	0	0	0.5	0.5	0	0.5	0.5	0.5	0.5	0	0	0	0	0	0	0	0	0	0	0	-1	-1	-1	-1	-1	-1	-1	-1	0	-1	-1	-1	
M00476: ComP-ComA_(competence)_two-component_regulatory_system	0	0	0	0	0	0	0	0	0	0	0	0	0	0	0	0	0	0	0	0	0	0	0	0	0	0	0	0	0	0	0	0	0	0	0	0	0	0	0	0	0	0	0	0	0	0	0	0	0	0	0	0	0	0	0	0	0	0	0	0	0	0	0	0	0	-1	-1	-1	-1	-1	-1	-1	-1	-1	-1	-1	-1	
M00477: EvgS-EvgA_(acid_and_drug_tolerance)_two-component_regulatory_system	0	0	0	0	0	0	0	0	0	0	0	0	0.5	0.5	0	0	0	0	0	0	0	0	0	0.5	0	0	0	0	0	0	0	0	0	0	0	0	0	0	0	0	0	0	0	0	0	0	0	0	0	0.5	0	0	0.5	1	0	0	0	0	0	0	0	0	0	0	0	-1	-1	-1	-1	-1	-1	-1	-1	-1	-1	-1	-1	
M00478: DegS-DegU_(multicellular_behavior_control)_two-component_regulatory_system	0	0	0	0	0	0	0	0	0	0	0	0	0	0	0	0	0.5	0.5	0.5	0.5	0.5	0.5	0.5	0.5	0.5	0	0	0	0	0	0	0	0	0	0	0	0	0	0	0	0	0.5	0	0	0	0.5	0	0	0	0	0	0	0	0	0	0	0	0	0	0.5	0	0	0	0	0	-1	-1	0	-1	-1	-1	-1	-1	-1	-1	-1	-1	
M00479: DesK-DesR_(membrane_lipid_fluidity_regulation)_two-component_regulatory_system	1	1	0	0	1	1	1	1	1	1	1	1	1	1	1	1	1	1	1	0.5	0.5	1	1	0.5	0.5	0.5	0.5	0.5	0.5	0.5	0.5	0.5	0.5	0	0.5	0	0	1	0	0	0	1	0	0.5	0	1	0	0	0	0	0	0	0	0.5	0	0	0	0	0	0	0	0	0	0	0	0	1	1	0	0	-1	-1	0	-1	-1	-1	-1	varied
M00480: VraS-VraR_(cell-wall_peptidoglycan_synthesis)_two-component_regulatory_system	0	0	0	0	0	0	0	0	0	0	0	0	0	0	0	0	0	0	0	0	0	0	0	0	0	0	0	0	0	0	0	0	0	0	0	0	0	0	0	0	0	0	0	0	0	0	0	0	0	0	0	0	0	0	0	0	0	0	0	0	0	0	0	0	0	-1	-1	-1	-1	-1	-1	-1	-1	-1	-1	-1	-1	
M00481: LiaS-LiaR_(cell_wall_stress_response)_two-component_regulatory_system	0	0	0	0	0	0	0	0	0	0	0	0	0	0	0	0	0.5	0	0	0	0	0	0	0	0	0	0	0	0	0	0	0	0	0	0	0	0	0	0	0	0	0	0	0	0	0	0	0	0	0	0	0	0	0	0	0	0	0	0	0	0	0	0	0	0	-1	-1	-1	-1	-1	-1	-1	-1	-1	-1	-1	-1	
M00482: DevS-DevR_(redox_response)_two-component_regulatory_system	0	0	0	0	0	0	0	0	0	0	0	0	0	0	0	0	0	0	0	0	0	0	0	0	0	0	0	0	0	0	0	0	0	0	0	0	0	0	0	0	0	0	0	0	0	0	0	0	0	0	0	0	0	0	0	0	0	0	0	0	0	0	0	0	0	-1	-1	-1	-1	-1	-1	-1	-1	-1	-1	-1	-1	
M00483: NreB-NreC_(dissimilatory_nitrate/nitrite_reduction)_two-component_regulatory_system	0	0	0	0	0	0	0	0	0	0	0	0	0	0	0	0	0.5	0	0	0	0	0	0	0	0	0	0	0	0	0	0	0	0	0	0	0	0	0	0	0	0	0	0	0	0	0	0	0	0	0	0	0	0	0	0	0	0	0	0	0	0	0	0	0	0	-1	-1	-1	-1	-1	-1	-1	-1	-1	-1	-1	-1	
M00484: YdfH-YdfI_two-component_regulatory_system	0	0	0	0	0	0	0	0	0	0	0	0	0	0	0	0	0	0	0	0	0	0	0	0	0	0	0	0	0	0	0	0	0	0	0	0	0	0	0	0	0	0	0	0	0	0	0	0	0	0	0	0	0	0	0	0	0	0	0	0	0	0	0	0	0	-1	-1	-1	-1	-1	-1	-1	-1	-1	-1	-1	-1	
M00485: KinABCDE-Spo0FA_(sporulation_control)_two-component_regulatory_system	0	0	0	0	0	0	0	0	0	0	0	0	0	0	0	0	0	0	0	0	0	0	0	0	0	0	0	0	0	0	0	0	0	0	0	0	0	0	0	0	0	0	0	0	0	0	0	0	0	0	0	0	0	0	0	0	0	0	0	0	0	0	0	0	0	-1	-1	-1	-1	-1	-1	-1	-1	-1	-1	-1	-1	
M00486: CitA-CitB_(citrate_fermentation)_two-component_regulatory_system	0	0	0	0	0	0	0	0	0	0	0	0	0	0	0	0	0	0	0	0	0	0	0	0	0	0	0	0	0	0	0	0	0	0	0	0	0	0	0	0	0	0	0	0	0	0	0	0	0	0	0	0	0	0	0	0	0	0	0	0	0	0	0	0	0	-1	-1	-1	-1	-1	-1	-1	-1	-1	-1	-1	-1	
M00488: DcuS-DcuR_(C4-dicarboxylate_metabolism)_two-component_regulatory_system	0	0	0	0	0	0	0	0	0	0	0	0	0	0	0	0	0	0	0	0	0	0	0	0	0	0	0	0	0	0	0	0	0	0	0	0	0	0	0	0	0	0	0	0	0	0	0	0	0	0	0	0	0	0	0	0	0	0	0	0	0	0	0	0	0	-1	-1	-1	-1	-1	-1	-1	-1	-1	-1	-1	-1	
M00489: DctS-DctR_(C4-dicarboxylate_transport)_two-component_regulatory_system	0	0	0	0	0	0	0	0	0	0	0	0	0	0	0	0	0	0	0	0	0	0	0	0	0	0	0	0	0	0	0	0	0	0	0	0	0	0	0	0	0	0	0	0	0	0	0	0	0	0	0	0	0	0	0	0	0	0	0	0	0	0	0	0	0	-1	-1	-1	-1	-1	-1	-1	-1	-1	-1	-1	-1	
M00490: MalK-MalR_(malate_transport)_two-component_regulatory_system	0	0	0	0	0	0	0	0	0	0	0	0	0	0	0	0	0	0	0	0	0	0	0	0	0	0	0	0	0	0	0	0	0	0	0	0	0	0	0	0	0	0	0	0	0	0	0	0	0	0	0	0	0	0	0	0	0	0	0	0	0	0	0	0	0	-1	-1	-1	-1	-1	-1	-1	-1	-1	-1	-1	-1	
M00491: arabinogalactan_oligomer/maltooligosaccharide_transport_system	0.2	0.2	0.2	0.2	0.2	0.2	0.2	0.4	0.2	0.2	0.2	0.2	0.2	0.2	0.2	0.2	0.8	0.2	0.8	0.4	0.2	0.4	0.2	0.2	0.4	0.2	0.4	0.2	0.2	0.4	0.2	0.2	0.2	0.4	0.4	0.4	0.2	0.2	0.4	0.4	0.2	0.2	0.4	0.4	0.4	0.2	0.2	0.4	0.4	0.4	0.4	0.4	0.4	0.4	0.2	0	0.2	0.2	0.2	0.2	0.2	0	0	0.4	0.2	0	0	0	0	0	0	0	0	0	0	0	0	
M00492: LytS-LytR_two-component_regulatory_system	0.5	0.5	0	0	0	0	0	0	0.5	0	0	0	0	0	0	0	0	0.5	0	0.5	0.5	0	0	0.5	0	0	0	0	0	0	0	0	0	0	0	0	0	0	0	0	0	0	0	0	0	0	0	0	0	0	0	0	0	0	0	0	0	0	0	0	0	0	0	0	0.5	0	-1	-1	-1	-1	-1	-1	-1	-1	-1	-1	-1	
M00493: AlgZ-AlgR_(alginate_production)_two-component_regulatory_system	1	1	1	1	0	0.5	0	0	0	0	0	0	0	0	0	0	0	0	0	0	0	1	0	0	1	0	0	0	0	0	0	1	1	0	1	0.5	0	0	0	0	0.5	0	0.5	0.5	1	0	0.5	1	1	0.5	1	1	1	1	1	1	0	1	1	0	1	1	1	1	0	1	-1	-1	-1	-1	-1	0	0	1	1	1	1	varied
M00495: AgrC-AgrA_(exoprotein_synthesis)_two-component_regulatory_system	0	0	0	0	0	0	0	0	0	0	0	0	0	0	0	0	0	0	0	0	0	0	0	0	0	0	0	0	0	0	0	0	0	0	0	0	0	0	0	0	0	0	0	0	0	0	0	0	0	0	0	0	0	0	0	0	0	0	0	0	0	0	0	0	0	-1	-1	-1	-1	-1	-1	-1	-1	-1	-1	-1	-1	
M00497: GlnL-GlnG_(nitrogen_regulation)_two-component_regulatory_system	1	1	1	1	0	1	1	0	0	0	0	0	0	0	0	0	1	0.5	0.5	0.5	0.5	1	0.5	0.5	1	1	1	1	1	1	1	1	1	1	1	1	1	0	1	1	1	0	1	1	1	0.5	1	1	1	1	1	1	1	1	1	1	1	1	1	1	1	1	1	1	1	1	-1	0	1	1	1	1	1	1	1	1	1	varied
M00498: NtrY-NtrX_(nitrogen_regulation)_two-component_regulatory_system	0.5	0.5	0	0	1	0.5	0	1	1	1	1	0.5	1	0.5	1	0.5	0.5	1	0.5	1	1	0.5	1	1	0.5	1	1	1	1	1	1	1	1	1	1	1	1	0	1	1	1	1	1	1	1	0	1	1	1	0	1	1	1	1	0	0	1	0	1	1	0	0	0	1	1	0	1	1	1	1	1	1	1	1	-1	1	0	varied
M00499: HydH-HydG_(metal_tolerance)_two-component_regulatory_system	0.5	0.5	0.5	0.5	1	1	0.5	1	1	1	1	0.5	1	1	1	0.5	0.5	1	0.5	1	1	0.5	0.5	1	0.5	1	1	1	1	1	1	1	1	0.5	0.5	0	0	0	0	0	0.5	0.5	0	0	0	0.5	0	0	0	0	0	0	0	0	0.5	0.5	1	1	1	1	0.5	0	0	0.5	1	0	1	0	1	1	-1	0	-1	-1	0	1	0	varied
M00500: AtoS-AtoC_(cPHB_biosynthesis)_two-component_regulatory_system	0.5	0.5	0.5	0.5	0.5	0.5	0	0	0.5	0.5	0.5	0.5	0.5	0.5	0.5	0.5	0.5	0.5	0.5	0.5	0.5	0.5	0.5	0.5	0.5	0.5	0.5	0.5	0.5	0.5	0.5	0.5	0.5	0.5	0.5	0	0	0.5	0	0	0.5	0.5	0	0.5	0	0.5	0	0	0	0	0	0	0	0	0.5	0	1	0.5	0.5	0.5	0.5	0	0	0.5	1	0	0	0	0	0	-1	0	0	-1	0	0	0	
M00501: PilS-PilR_(type_4_fimbriae_synthesis)_two-component_regulatory_system	1	1	1	1	0.5	1	0.5	0.5	0.5	0.5	0.5	0.5	0.5	0.5	1	0.5	1	1	1	1	1	1	1	1	1	1	1	1	1	1	1	1	1	0.5	1	0	0	0	0	0	1	0	0	0.5	1	0	0	1	1	1	1	1	1	1	1	1	1	1	1	1	1	1	1	1	1	1	0	1	1	1	-1	-1	0	1	1	1	1	varied
M00502: GlrK-GlrR_(amino_sugar_metabolism)_two-component_regulatory_system	0	0	0.5	1	0	0	0	0	0	0	0	0	0	0	0	0	0	0	0	0	0	0	0	0	0	0	0	0	0	0	0	0	0	0	0	0	0	0	0	0	0	0	0	0	0	0	0	0	0	0	0	0	0	0	1	0.5	0	1	1	0	1	1	0.5	0	0	0	-1	-1	-1	-1	-1	-1	-1	-1	0	1	0	varied
M00504: DctB-DctD_(C4-dicarboxylate_transport)_two-component_regulatory_system	0	0	0	0	0	0	0	0	0	0	0	0	0	0	0	0	0	0	0	0	0	0	0	0	0	0	0	0	0	0.5	0	0.5	1	1	0.5	1	1	0	1	1	1	0	1	1	1	0	1	1	1	1	1	1	1	1	1	0	1	[illegible]	[illegible]	0	1	1	0	1	0	-1	-1	-1	-1	0	1	1	1	1	0	1	0	varied
M00505: KinB-AlgB_(alginate_production)_two-component_regulatory_system	0	0	0	0	0	0	0	0	0	0	0	0	0	0	0	0	0	0.5	0	0.5	0.5	0	0.5	0.5	0	0.5	0	0.5	0	0	0	0	0	0.5	0	0	0	0	0	0	0	0	0	0	0	0.5	0	0	0	0	0	0	0	0	0	0	0	0	0	0	0	0	0	0	0	-1	-1	0	0	-1	-1	-1	-1	-1	-1	-1	-1	
M00506: CheA-CheYBV_(chemotaxis)_two-component_regulatory_system	1	0.8	1	1	0.6	0.6	0.6	0.8	0.6	0.6	0.6	0.8	0.6	0.6	0.6	0.6	0.8	0.8	0.8	0.8	0.8	0.8	0.8	0.8	0.8	1	1	1	1	1	1	1	1	1	1	0.8	0.8	0.6	0.8	0.8	0.8	0.8	0.8	0.8	0.8	0.8	0.8	0.8	0.8	0.6	0.8	0.8	0.8	0.8	0.8	0.8	0.8	1	0.8	0.8	1	0.8	0.8	1	0.6	1	0	0	1	1	0	0	0	0	0	0	0	
M00507: ChpA-ChpB/PilGH_(chemosensory)_two-component_regulatory_system	0.6	0.6	0.6	0.6	0	0.6	0	0	0	0	0	0	0	0	0.2	0	0.6	0.6	0.6	0.6	0.6	0.6	0.6	0.6	0.6	0.6	0.6	0.6	0.6	0.6	0.6	0.6	0.6	0.2	0.6	0	0	0	0	0	0.2	0.2	0	0	0.6	0.4	0.2	0.6	0.6	0.6	0.6	0.6	0.6	0.6	0.6	0.8	0.6	0.6	0.6	0.4	0.8	0.8	0.8	0.8	0.6	0	-1	0	0	0	-1	0	0	0	0	0	0	

Table S4. IV

KEGG Module	Habitat																																																															1 if median >= 90% for the samples per site; -1 if median <= 10% for the samples per site												Kind of Module inferred (Core or Varied)
	India oil refineries				Arctic												China oil refineries									Mangrove			Marine sediments							Taiga upper active layer				Taiga bottom active layer				Taiga permafrost layer				Oil sands core							Oil sands tailings pond upper				Oil sands tailings pond median			Oil sands tailings pond deep																
	I1	I2	I3	I4	A1	A10	A11	A12	A2	A3	A4	A5	A6	A7	A8	A9	C1	C2	C3	C4	C5	C6	C7	C8	C9	M1	M2	M3	DWH1	DWH2	DWH3	DWH4	DWH5	DWH6	DWH7	Tu1	Tu2	Tu3	Tu4	Tb1	Tb2	Tb3	Tb4	Tp1	Tp2	Tp3	Tp4	OSC1	OSC12	OSC3	OSC4	OSC5	OSC7	OSC9	OSTPu1	OSTPu2	OSTPu4	OSTPu3	OSTPm2	OSTPm6	OSTPm4	OSTPd1	OSTPd2	OSTPd3	OSTPd4	India oil refineries	Arctic	China oil refineries	Mangrove	Marine sediments	Taiga upper active layer	Taiga bottom active layer	Taiga permafrost layer	Oil sands core	Oil sands tailings pond upper	Oil sands tailings pond median	Oil sands tailings pond deep	
M00508: PixL-PixGH_(positive_phototaxis)_two-component_regulatory_system	0	0	0	0	0	0	0	0	0	0	0	0	0	0	0	0	0	0	0	0	0	0	0	0	0	0	0	0	0	0	0	0	0	0	0	0	0	0	0	0	0	0	0	0	0	0	0	0	0	0	0	0	0	0	0	0	0	0	0	0	0	0	0	0	0	-1	-1	-1	-1	-1	-1	-1	-1	-1	-1	-1	-1	
M00509: WspE-WspRF_(chemosensory)_two-component_regulatory_system	0	0	0	0	0	0	0	0	0	0	0	0	0	0	0	0	0	0	0	0	0	0	0	0.666667	0	0	0	0	0	0	0	0	0	0	0	0	0	0	0	0	0	0	0	0	0	0	0	0	0	0	0	0	0	0	0	0	0	0	0	0	0	0	0	0	0	-1	-1	-1	-1	-1	-1	-1	-1	-1	-1	-1	-1	
M00510: Cph1-Rcp1_(light_response)_two-component_regulatory_system	0	0	0	0	0	0	0	0	0	0	0	0	0	0	0	0	0	0	0	0	0	0	0	0	0	0	0	0	0	0	0	0	0	0	0	0	0	0	0	0	0	0	0	0	0	0	0	0	0	0	0	0	0	0	0	0	0	0	0	0	0	0	0	0	0	-1	-1	-1	-1	-1	-1	-1	-1	-1	-1	-1	-1	
M00511: PleC-PleD_(cell_fate_control)_two-component_regulatory_system	0	0	0	0	0	0	0	0	0	0	0	0	0	0	0	0	0.2	0.4	0.2	0.4	0.4	0.2	0.4	0.4	0.2	0.4	0.4	0.4	0	0	0	0	0	0.4	0	1	0.8	0.2	0.8	1	0.8	0.6	1	0.8	0.4	0.2	1	0.4	0.4	0	0.4	0.4	0.4	0.4	0	0	0	0	0.2	0.4	0	0	0	0.4	0.2	-1	-1	0	0	-1	0	0	0	0	-1	0	0	
M00512: CckA-CtrA/CpdR_(cell_cycle_control)_two-component_regulatory_system	0	0	0	0	0	0	0	0	0	0	0	0	0	0	0	0	0	0.333333	0	0.333333	0.333333	0	0.333333	0.333333	0	0	0	0	0	0	0	0	0	0.333333	0	1	1	0	1	1	1	1	1	1	1	0	1	1	1	0	1	1	1	1	0	0	0	0	0	0	0	0	0	0	0	-1	-1	0	-1	-1	1	1	1	1	-1	-1	-1	varied
M00513: LuxQN/CqsS-LuxU-LuxO_(quorum_sensing)_two-component_regulatory_system	0	0	0	0	0	0	0	0	0	0	0	0	0	0	0	0	0	0	0	0	0	0	0	0	0	0	0	0	0	0	0	0	0	0	0	0	0	0	0	0	0	0	0	0	0	0	0	0	0	0.25	0	0	0	0	0	0	0	0	0	0	0	0	0	0	0	-1	-1	-1	-1	-1	-1	-1	-1	-1	-1	-1	-1	
M00514: TtrS-TtrR_(tetrathionate_respiration)_two-component_regulatory_system	0	0	0	0	0	0	0	0	0	0	0	0	0	0	0	0	0	0	0	0	0	0	0	0	0	0	0	0	0	0	0	0	0	0	0	0	0	0	0	0	0	0	0	0	0	0	0	0	0	0	0	0	0	0	0	0	0	0	0	0	0	0	0	0	0	-1	-1	-1	-1	-1	-1	-1	-1	-1	-1	-1	-1	
M00515: FlrB-FlrC_(polar_flagellar_synthesis)_two-component_regulatory_system	0	0	0.5	0	0	0	0	0	0	0.5	0	0	0	0	0	0	0	0	0	0	0	0	0	0	0	0.5	0.5	0.5	0	0.5	0.5	0.5	0.5	0	0.5	0.5	0	0	0	0	0.5	0	0.5	0.5	0.5	0	0.5	0	0	0	0	0	0	0	0	0	0	0	0.5	0.5	0	0	0	0	0.5	-1	-1	-1	0	0	-1	0	0	-1	-1	0	-1	
M00517: RpfC-RpfG_(cell-to-cell_signaling)_two-component_regulatory_system	0	0	0	0	0	0	0	0	0	0	0	0	0	0	0	0	0	0	0	0	0	0	0	0	0	0	0	0	0	0	0	0	0	0	0	0	0	0	0	0	0	0	0	0	0	0	0	0	0	0	0	0	0	0	0	0	0	0	0	0	0	0	0	0	0	-1	-1	-1	-1	-1	-1	-1	-1	-1	-1	-1	-1	
M00518: GlnK-GlnL_(glutamine_utilization)_two-component_regulatory_system	0	0	0	0	0	0	0	0	0	0	0	0	0	0	0	0	0	0	0	0	0	0	0	0	0	0	0	0	0	0	0	0	0	0	0	0	0	0	0	0	0	0	0	0	0	0	0	0	0	0	0	0	0	0	0	0	0	0	0	0	0	0	0	0	0	-1	-1	-1	-1	-1	-1	-1	-1	-1	-1	-1	-1	
M00519: YesM-YesN_two-component_regulatory_system	0	0	0	0	0	0	0	0	0	0	0	0	0	0	0	0	0	0	0	0	0	0	0	0	0	0.5	0	0.5	0	0	0	0	0	0	0	0	0	0	0	0	0	0	0	0	0	0	0	0	0	0	0	0	0	0	0	0	0.5	0	0	0	0	0	0	0	0	-1	-1	-1	0	-1	-1	-1	-1	-1	-1	-1	-1	
M00520: ChvG-ChvI_(acidity_sensing)_two-component_regulatory_system	0	0	0	0	0	0	0	0	0	0	0	0	0	0	0	0	0	0	0	0	0	0	0	0	0	0	0	0	0	0	0	0	0	0	0	1	1	0	1	1	1	1	1	1	1	0	1	1	1	0	1	1	1	1	0	0	0	0	0	0	0	0	0	0	0	-1	-1	-1	-1	-1	1	1	1	1	-1	-1	-1	varied
M00521: CiaH-CiaR_two-component_regulatory_system	0	0	0	0	0	0	0	0	0	0	0	0	0	0	0	0	0	0	0	0	0	0	0	0	0	0	0	0	0	0	0	0	0	0	0	0	0	0	0	0	0	0	0	0	0	0	0	0	0	0	0	0	0	0	0	0	0	0	0	0	0	0	0	0	0	-1	-1	-1	-1	-1	-1	-1	-1	-1	-1	-1	-1	
M00522: SalK-SalR_two-component_regulatory_system	0	0	0	0	0	0	0	0	0	0	0	0	0	0	0	0	0	0	0	0	0	0	0	0	0	0	0	0	0	0	0	0	0	0	0	0	0	0	0	0	0	0	0	0	0	0	0	0	0	0	0	0	0	0	0	0	0	0	0	0	0	0	0	0	0	-1	-1	-1	-1	-1	-1	-1	-1	-1	-1	-1	-1	
M00523: RegB-RegA_(redox_response)_two-component_regulatory_system	1	1	1	1	0	1	1	0	1	0	0	1	0	0	1	1	0	0	0	1	0	1	0	0	1	1	1	1	1	1	1	1	1	1	1	1	1	0	1	1	1	1	1	1	1	0	1	1	1	1	1	1	1	1	1	1	0	1	1	0	1	1	1	1	0	1	0	-1	1	1	1	1	1	1	1	1	1	varied
M00524: FixL-FixJ_(nitrogen_fixation)_two-component_regulatory_system	0	0	0	0	0.5	0	0	0	0	0.5	0.5	0	0	0	0	0	0	0	0	0	0	0	0	0	0	0	0	0	0	0	0	0	0	0	0	1	1	0	1	1	1	1	1	1	1	0	1	0	0	0	0	0	0	1	0	0	0	0	0	0	0	0	0	0	0	-1	-1	-1	-1	-1	1	1	1	-1	-1	-1	-1	varied
M00525: Lysine_biosynthesis,_acetyl-DAP_pathway,_aspartate_=>_lysine	0.666667	0.666667	0.666667	0.666667	0.666667	0.666667	0.666667	0.666667	0.666667	0.666667	0.666667	0.666667	0.666667	0.666667	0.666667	0.666667	0.666667	0.666667	0.666667	0.666667	0.666667	0.666667	0.666667	0.666667	0.666667	0.666667	0.666667	0.666667	0.666667	0.666667	0.666667	0.666667	0.666667	0.666667	0.666667	0.666667	0.666667	0.666667	0.666667	0.666667	0.666667	0.666667	0.666667	0.666667	0.666667	0.666667	0.666667	0.666667	0.666667	0.666667	0.666667	0.666667	0.666667	0.666667	0.666667	0.666667	0.666667	0.666667	0.666667	0.666667	0.666667	0.666667	0.666667	0.666667	0.666667	0	0	0	0	0	0	0	0	0	0	0	0	
M00526: Lysine_biosynthesis,_DAP_dehydrogenase_pathway,_aspartate_=>_lysine	0.5	0.5	0.5	0.5	0.5	0.5	0.5	0.5	0.5	0.5	0.5	0.5	0.5	0.5	0.5	0.5	0.5	0.5	0.5	0.5	0.5	0.5	0.5	0.5	0.5	0.6	0.5	0.6	0.5	0.5	0.5	0.5	0.5	0.5	0.5	0.5	0.5	0.5	0.5	0.5	0.5	0.5	0.5	0.5	0.5	0.5	0.5	0.5	0.5	0.5	0.5	0.5	0.5	0.5	0.5	0.6	0.5	0.5	0.5	0.6	0.6	0.6	0.6	0.6	0.5	0	0	0	0	0	0	0	0	0	0	0	0	
M00527: Lysine_biosynthesis,_DAP_aminotransferase_pathway,_aspartate_=>_lysine	0.545455	0.545455	0.545455	0.545455	0.545455	0.545455	0.545455	0.545455	0.545455	0.636364	0.636364	0.545455	0.545455	0.545455	0.545455	0.545455	0.636364	0.636364	0.636364	0.636364	0.636364	0.636364	0.636364	0.636364	0.636364	0.727273	0.636364	0.727273	0.636364	0.545455	0.636364	0.636364	0.545455	0.636364	0.545455	0.545455	0.545455	0.636364	0.545455	0.545455	0.545455	0.545455	0.545455	0.545455	0.545455	0.636364	0.545455	0.545455	0.545455	0.545455	0.545455	0.545455	0.545455	0.545455	0.545455	0.545455	0.636364	0.636364	0.636364	0.727273	0.545455	0.545455	0.545455	0.545455	0.636364	0	0	0	0	0	0	0	0	0	0	0	0	
M00528: Nitrification,_ammonia_=>_nitrite	0	0	0	0	0	0	0	0	0	0	0	0	0	0	0	0	0	0	0	0	0	0	0	0	0	0	0	0	0	0	0	0	0	0	0	0	0	0	0	0	0	0	0	0	0	0	0	0	0	0	0	0	0	0	0	0	0	0	0	0	0.25	0	0	0	0	-1	-1	-1	-1	-1	-1	-1	-1	-1	-1	-1	-1	
M00529: Denitrification,_nitrate_=>_nitrogen	0	0.083333	0.25	0.583333	0.083333	0.083333	0.083333	0.083333	0	0.083333	0.083333	0	0.083333	0	0.083333	0	0.166667	0.166667	0.416667	0.166667	0.166667	0.083333	0.166667	0.166667	0.166667	0.083333	0.083333	0.083333	0	0	0	0	0	0.083333	0	0	0	0.333333	0	0	0	0.083333	0	0	0.083333	0.333333	0	0.416667	0.416667	0.416667	0.416667	0.416667	0.416667	0.416667	0.25	0.666667	0	0.083333	0.25	0.083333	0.666667	0.666667	0.666667	0.666667	0.083333	0	-1	0	-1	-1	-1	-1	-1	0	0	0	0	
M00530: Dissimilatory_nitrate_reduction,_nitrate_=>_ammonia	0.2	0.2	0.2	0.6	0.3	0.3	0.3	0.3	0.2	0.3	0.3	0.2	0.3	0.2	0.3	0.2	0.2	0.3	0.6	0.4	0.3	0.2	0.3	0.4	0.2	0.3	0.2	0.2	0.2	0.2	0.2	0.2	0.2	0.2	0.2	0.2	0.2	0.6	0.2	0.2	0.2	0.2	0.2	0.2	0.2	0.5	0.2	0.6	0.6	0.6	0.6	0.6	0.6	0.6	0.2	0.6	0.2	0.2	0.3	0.2	0.6	0.6	0.6	0.6	0.3	0	0	0	0	0	0	0	0	0	0	0	0	
M00531: Assimilatory_nitrate_reduction,_nitrate_=>_ammonia	0.166667	0.166667	0.166667	0.166667	0.166667	0.166667	0.166667	0.166667	0.166667	0.166667	0.166667	0.166667	0.333333	0.333333	0.166667	0.166667	0.166667	0.333333	0.166667	0.333333	0.333333	0.166667	0.333333	0.333333	0.333333	0.166667	0.166667	0.166667	0.166667	0.166667	0.166667	0.166667	0.166667	0.333333	0.166667	0.166667	0	0.166667	0	0.166667	0.166667	0.166667	0.166667	0.166667	0.166667	0.333333	0.166667	0.166667	0.166667	0.166667	0.166667	0.166667	0.166667	0.166667	0	0.166667	0	0.166667	0.166667	0	0.166667	0.166667	0	0.166667	0.166667	0	0	0	0	0	-1	0	0	0	-1	0	0	
M00532: Photorespiration	0.466667	0.466667	0.533333	0.6	0.4	0.466667	0.4	0.4	0.4	0.4	0.4	0.4	0.4	0.4	0.4	0.4	0.466667	0.533333	0.533333	0.466667	0.533333	0.533333	0.533333	0.533333	0.466667	0.4	0.466667	0.4	0.466667	0.466667	0.4	0.4	0.4	0.466667	0.466667	0.466667	0.4	0.466667	0.4	0.533333	0.466667	0.4	0.466667	0.466667	0.466667	0.4	0.466667	0.4	0.4	0.533333	0.4	0.4	0.4	0.466667	0.466667	0.466667	0.466667	0.6	0.466667	0.4	0.6	0.466667	0.4	0.466667	0.4	0	0	0	0	0	0	0	0	0	0	0	0	
M00533: Homoprotocatechuate_degradation,_homoprotocatechuate_=>_2-oxohept-3-enedioate	0	0	0.2	0	0.2	0.2	0.2	0	0	0	0	0	0.2	0.2	0	0	0	0	0	0	0	0.2	0	0	0	0	0	0	0	0	0	0	0	0	0	0.2	0	0.2	0	0.2	0.2	0	0	0.2	0.2	0	0	0.2	0.2	0.2	0.2	0.2	0.2	0.2	0.2	0.2	0	0.4	0	0	0.4	0.2	0.2	0.2	0.2	-1	-1	-1	-1	-1	0	0	0	0	0	-1	0	
M00534: Naphthalene_degradation,_naphthalene_=>_salicylate	0	0	0	0	0	0	0	0	0	0	0	0	0	0	0	0	0	0	0	0	0	0	0	0	0	0	0	0	0	0	0	0	0	0	0	0	0	0	0	0	0	0	0	0	0	0	0	0	0	0	0	0	0	0	0	0	0	0	0	0	0	0	0	0	0	-1	-1	-1	-1	-1	-1	-1	-1	-1	-1	-1	-1	
M00535: Isoleucine_biosynthesis,_pyruvate_=>_2-oxobutanoate	0.75	0.75	0.75	0.75	0.75	0.75	0.75	0.75	0.75	0.75	0.75	0.75	0.75	0.75	0.75	0.75	0.75	0.75	0.75	0.75	0.75	0.75	0.75	0.75	0.75	0.75	0.75	0.75	0.75	0.75	0.75	0.75	0.75	0.75	0.75	0.75	0.75	0.75	0.75	0.75	0.75	0.75	0.75	0.75	0.75	0.75	0.75	0.75	0.75	0.75	0.75	0.75	0.75	0.75	0.75	0.75	0.75	0.75	0.75	0.75	0.75	0.75	0.75	0.75	0.75	0	0	0	0	0	0	0	0	0	0	0	0	
M00537: Xylene_degradation,_xylene_=>_methylbenzoate	0	0	0	0	0.25	0	0.25	0.25	0	0	0	0	0.25	0.25	0	0	0	0	0.25	0	0	0	0	0	0	0	0	0	0	0	0	0	0	0	0	0	0	0	0	0	0	0	0	0	0	0	0	0.25	0.25	0.25	0.25	0.25	0.25	0.5	0	0.25	0	0	0	0	0.25	0.25	0.25	0.25	0	-1	-1	-1	-1	-1	-1	-1	-1	0	-1	-1	0	
M00538: Toluene_degradation,_toluene_=>_benzoate	0	0	0	0	0.125	0.125	0.125	0.125	0	0	0	0	0.125	0.125	0	0	0	0	0.125	0	0	0	0	0	0	0	0	0	0	0	0	0	0	0	0	0	0	0	0	0	0	0	0	0	0	0	0	0.125	0.125	0.125	0.125	0.125	0.125	0.25	0	0.125	0	0	0	0	0.125	0.125	0.125	0.125	0	-1	-1	-1	-1	-1	-1	-1	-1	0	-1	-1	0	
M00539: Cumate_degradation,_p-cumate_=>_2-oxopent-4-enoate_+_2-methylpropanoate	0	0	0	0	0	0	0	0	0	0	0	0	0	0	0	0	0	0	0	0	0	0	0	0	0	0	0	0	0	0	0	0	0	0	0	0	0	0	0	0	0	0	0	0	0	0	0	0	0	0.125	0	0	0	0	0	0	0	0	0	0	0	0	0	0	0	-1	-1	-1	-1	-1	-1	-1	-1	-1	-1	-1	-1	
M00540: Benzoate_degradation,_cyclohexanecarboxylic_acid_=>pimeloyl-CoA	0	0	0	0	0	0	0	0	0	0	0	0	0	0	0	0	0	0	0	0	0	0	0	0	0	0	0	0	0	0	0	0	0	0	0	0	0	0.2	0	0	0	0	0	0	0	0	0	0	0	0	0	0	0	0	0	0	0	0	0	0	0	0	0	0	0	-1	-1	-1	-1	-1	-1	-1	-1	-1	-1	-1	-1	
M00541: Benzoyl-CoA_degradation,_benzoyl-CoA_=>_3-hydroxypimeloyl-CoA	0	0	0	0	0	0	0	0	0	0	0	0	0	0	0	0	0	0	0	0	0	0	0	0	0	0	0	0	0	0	0	0	0	0	0	0	0	0	0	0	0	0	0	0	0	0	0	0	0	0	0	0	0	0	0	0	0	0	0	0	0	0	0	0	0	-1	-1	-1	-1	-1	-1	-1	-1	-1	-1	-1	-1	
M00542: EHEC/EPEC_pathogenicity_signature,_T3SS_and_effectors	0	0	0	0	0	0	0	0	0	0	0	0	0	0	0	0	0	0	0	0	0	0	0	0	0	0	0	0	0	0	0	0	0	0	0	0	0	0	0	0	0	0	0	0	0	0	0	0	0	0.529412	0	0	0	0	0	0	0	0	0	0	0	0	0	0	0	-1	-1	-1	-1	-1	-1	-1	-1	-1	-1	-1	-1	
M00543: Biphenyl_degradation,_biphenyl_=>_2-oxopent-4-enoate_+_benzoate	0	0	0	0	0	0	0	0	0	0	0	0	0	0	0	0	0	0	0	0	0	0	0	0	0	0	0	0	0	0	0	0	0	0	0	0	0	0	0	0	0	0	0	0	0	0	0	0	0	0	0	0	0	0	0	0	0	0	0	0	0	0	0	0	0	-1	-1	-1	-1	-1	-1	-1	-1	-1	-1	-1	-1	
M00544: Carbazole_degradation,_carbazole_=>_2-oxopent-4-enoate_+_anthranilate	0	0	0	0	0	0	0	0	0	0	0	0	0	0	0	0	0	0	0	0	0	0	0	0	0	0	0	0	0	0	0	0	0	0	0	0	0	0	0	0	0	0	0	0	0	0	0	0	0	0	0	0	0	0	0	0	0	0	0	0	0	0	0	0	0	-1	-1	-1	-1	-1	-1	-1	-1	-1	-1	-1	-1	
M00545: Trans-cinnamate_degradation,_trans-cinnamate_=>_acetyl-CoA	0.181818	0	0.090909	0.090909	0.090909	0	0.272727	0.090909	0	0.090909	0	0.090909	0.090909	0.181818	0	0	0.545455	0.545455	0.545455	0.272727	0.272727	0.545455	0.363636	0.181818	0.545455	0.090909	0.090909	0.090909	0.181818	0.181818	0	0.181818	0.090909	0.090909	0.272727	0.545455	0.545455	0.545455	0.181818	0.272727	0.545455	0.272727	0.545455	0.545455	0.545455	0.545455	0.545455	0.181818	0.181818	0.454545	0.181818	0.181818	0.181818	0.272727	0.090909	0	0	0.090909	0	0.090909	0.090909	0.090909	0	0.181818	0	-1	-1	0	-1	0	0	0	0	0	-1	-1	-1	
M00546: Purine_degradation,_xanthine_=>_urea	0.2	0.2	0.05	0.2	0.05	0.05	0.15	0.05	0.2	0	0.05	0.2	0.2	0.2	0.2	0.2	0.2	0.2	0.25	0.15	0.15	0.5	0.2	0.15	0.4	0	0	0	0.2	0.05	0.05	0.2	0.05	0.15	0.2	0.35	0.2	0.3	0.15	0.35	0.3	0.15	0.35	0.3	0.3	0.15	0.35	0.55	0.5	0.4	0.55	0.5	0.5	0.55	0.15	0.15	0	0.15	0.05	0.05	0.15	0.15	0.15	0.2	0.15	0	0	0	-1	0	0	0	0	0	0	-1	0	
M00547: Benzene/toluene_degradation,_benzene_=>_catechol_/_toluene_=>_3-methylcatechol	0	0	0	0	0	0	0	0	0	0	0	0	0	0	0	0	0	0	0	0	0	0	0	0	0	0	0	0	0	0	0	0	0	0	0	0	0	0	0	0	0	0	0	0	0	0	0	0	0	0	0	0	0	0	0	0	0	0	0	0	0	0	0	0	0	-1	-1	-1	-1	-1	-1	-1	-1	-1	-1	-1	-1	
M00548: Benzene_degradation,_benzene_=>_catechol	0	0	0	0	0	0	0	0	0	0	0	0	0	0	0	0	0	0	0	0	0	0	0	0	0	0	0	0	0	0	0	0	0	0	0	0	0	0	0	0	0	0	0	0	0	0	0	0	0	0	0	0	0	0	0	0	0	0	0	0	0	0	0	0	0	-1	-1	-1	-1	-1	-1	-1	-1	-1	-1	-1	-1	
M00549: Nucleotide_sugar_biosynthesis,_glucose_=>_UDP-glucose	0.666667	0.666667	0.5	0.5	0.666667	0.5	0.666667	0.5	0.666667	0.5	0.5	0.666667	0.666667	0.666667	0.666667	0.666667	0.666667	0.666667	0.666667	0.666667	0.666667	0.666667	0.666667	0.666667	0.666667	0.5	0.5	0.5	0.5	0.5	0.5	0.5	0.5	0.5	0.5	0.5	0.333333	0.666667	0.333333	0.333333	0.5	0.5	0.333333	0.5	0.5	0.666667	0.333333	0.5	0.5	0.5	0.5	0.5	0.5	0.5	0.5	0.5	0.5	0.5	0.5	0.5	0.333333	0.5	0.5	0.5	0.5	0	0	0	0	0	0	0	0	0	0	0	0	
M00550: Ascorbate_degradation,_ascorbate_=>_D-xylulose-5P	0.285714	0.285714	0.142857	0.142857	0	0.142857	0.142857	0	0.142857	0	0	0	0	0	0	0	0	0	0	0	0	0	0	0	0	0	0	0	0	0	0	0	0	0	0	0	0	0.142857	0	0	0	0	0	0	0.142857	0	0	0.142857	0.142857	0.142857	0.142857	0.142857	0.142857	0.142857	0.142857	0.142857	0	0.142857	0.142857	0	0.142857	0.142857	0.142857	0.142857	0	0	-1	-1	-1	-1	-1	-1	-1	0	0	0	0	
M00551: Benzoate_degradation,_benzoate_=>_catechol_/_methylbenzoate_=>_methylcatechol	0.25	0	0	0	0	0	0	0	0	0	0	0	0	0	0	0	0	0	0	0	0	1	0	0	0.75	0	0	0	0	0	0	0.5	0.5	0	0.5	0.25	0	0	0	0	0.25	0	0.25	0.25	0.25	0	0.25	1	1	1	1	1	1	1	0	0	0	0	0	0	0	0	0	0.25	0	-1	-1	-1	-1	-1	-1	0	0	1	-1	-1	-1	varied
M00552: D-galactonate_degradation,_De_Ley-Doudoroff_pathway,_D-galactonate_=>_glycerate-3P	0.4	0.4	0.4	0.4	0.4	0.4	0.4	0.4	0.4	0.4	0.4	0.4	0.4	0.4	0.4	0.4	0.4	0.4	0.4	0.4	0.4	0.4	0.4	0.4	0.4	0.4	0.4	0.4	0.4	0.4	0.4	0.4	0.4	0.6	0.4	1	0.6	0.6	0.4	1	0.4	0.6	1	0.4	0.4	0.6	1	0.4	0.4	0.4	0.4	0.4	0.4	0.4	0.4	0.4	0.4	0.4	0.4	0.4	0.4	0.4	0.4	0.4	0.4	0	0	0	0	0	0	0	0	0	0	0	0	
M00554: Nucleotide_sugar_biosynthesis,_galactose_=>_UDP-galactose	1	1	1	1	1	0.5	1	1	1	1	1	1	1	1	1	1	1	1	1	1	1	1	1	1	1	1	0.5	0.5	0.5	0	0	0	0	0.5	0	1	1	1	1	0	1	1	1	1	1	1	1	0	0	0	0	0	0	1	0	0	0.5	0.5	0.5	1	0.5	0	0	0	0.5	1	1	1	0	-1	1	1	1	-1	0	0	-1	varied
M00555: Betaine_biosynthesis,_choline_=>_betaine	0.25	0.25	0.25	0.25	0.5	0.25	0.5	0.5	0.5	0.25	0.5	0.5	0.25	0.5	0.5	0.25	0.5	0.5	0.5	0.5	0.5	0.5	0.5	0.5	0.5	0.5	0.5	0.5	0.5	0.5	0.25	0.5	0.5	0.5	0.5	0.5	0.25	0.5	0.25	0.25	0.5	0.5	0.25	0.5	0.5	0.5	0.5	0.5	0.5	0.5	0.5	0.5	0.5	0.5	0.5	0.25	0.25	0.5	0.5	0.5	0.5	0.5	0.25	0.5	0.5	0	0	0	0	0	0	0	0	0	0	0	0	
M00565: Trehalose_biosynthesis,_D-glucose_1P_=>_trehalose	0.7	0.7	0.5	0.7	0.8	0.5	0.8	0.7	0.7	0.7	0.7	0.7	0.8	0.8	0.7	0.7	0.8	0.8	0.9	0.8	0.8	0.8	0.8	0.8	0.8	0.7	0.7	0.8	0.7	0.5	0.5	0.7	0.5	0.6	0.7	0.7	0	0.8	0.4	0.4	0.7	0.7	0.1	0.7	0.5	0.8	0.2	0.3	0.3	0.6	0.3	0.3	0.3	0.6	0.5	0.5	0.5	0.5	0.5	0.6	0.5	0.5	0.4	0.5	0.6	0	0	0	0	0	0	0	0	0	0	0	0	
M00566: Dipeptide_transport_system,_Firmicutes	0	0	0	0	0	0	0	0	0	0	0	0	0	0	0	0	0	0	0	0	0	0	0	0.25	0	0	0	0	0	0	0	0	0	0	0	0	0	0	0	0	0	0	0	0	0	0	0	0	0	0	0	0	0	0	0	0	0	0	0	0	0	0	0	0	0	-1	-1	-1	-1	-1	-1	-1	-1	-1	-1	-1	-1	
M00568: Catechol_ortho-cleavage,_catechol_=>_3-oxoadipate	0.2	0.2	0.2	0.2	0.4	0.2	0.4	0.4	0	0.2	0.2	0.2	0.4	0.4	0.2	0.2	0.2	0.4	0.8	0.2	0.2	0.8	0.2	0.2	0.8	0	0.2	0.2	0.2	0.2	0.2	0.2	0.2	0.2	0.2	0.4	0.4	0.6	0.2	0.2	0.4	0	0.4	0.4	0.4	0.4	0.6	1	0.8	0.8	0.8	0.8	1	1	0.2	0.8	0	0.2	0	0.2	0.8	0.8	0.8	0.8	0.4	0	0	0	0	0	0	0	0	0	0	0	0	
M00569: Catechol_meta-cleavage,_catechol_=>_acetyl-CoA_/_4-methylcatechol_=>_propanoyl-CoA	0.166667	0.416667	0.083333	0.166667	0.083333	0.416667	0.083333	0.083333	0.083333	0.083333	0.083333	0.083333	0.083333	0.083333	0.083333	0.083333	0.666667	0.583333	0.583333	0.25	0.333333	0.5	0.333333	0.25	0.583333	0.166667	0.166667	0.166667	0.166667	0.166667	0.083333	0.25	0.25	0.083333	0.25	0.333333	0.25	0.5	0.083333	0.083333	0.333333	0.166667	0.25	0.333333	0.333333	0.416667	0.333333	0.083333	0.083333	0.333333	0.083333	0.083333	0.083333	0.083333	0.083333	0.083333	0.083333	0.083333	0.083333	0.166667	0.083333	0.083333	0.083333	0.083333	0.083333	0	-1	0	0	0	0	0	0	-1	-1	-1	-1	
M00570: Isoleucine_biosynthesis,_threonine_=>_2-oxobutanoate_=>_isoleucine	0.75	0.75	0.75	0.75	0.75	0.75	0.75	0.75	0.75	0.75	0.75	0.75	0.75	0.75	0.75	0.75	0.75	0.75	0.75	0.75	0.75	0.75	0.75	0.75	0.75	0.75	0.75	0.75	0.75	0.75	0.75	0.75	0.75	0.75	0.75	0.75	0.75	0.75	0.75	0.75	0.75	0.75	0.75	0.75	0.75	0.75	0.75	0.75	0.75	0.75	0.75	0.75	0.75	0.75	0.75	0.75	0.75	0.75	0.75	0.75	0.75	0.75	0.75	0.75	0.75	0	0	0	0	0	0	0	0	0	0	0	0	
M00571: AlgE-type_Mannuronan_C-5-Epimerase_transport_system	0.333333	0.333333	0.333333	0.333333	0.333333	0.333333	0.333333	0.333333	0.333333	0.333333	0.333333	0.333333	0.333333	0	0.333333	0.333333	0.333333	0.333333	0.333333	0.333333	0.333333	0.333333	0.333333	0.333333	0.333333	0.333333	0.333333	0.333333	0.333333	0.333333	0.333333	0.333333	0.333333	0.333333	0.333333	0.333333	0.333333	0.333333	0.333333	0.333333	0.333333	0	0.333333	0.333333	0.333333	0	0.333333	0	0.333333	0.333333	0	0	0	0.333333	0	0	0	0.333333	0	0	0	0	0	0	0	0	0	0	0	0	0	0	0	-1	-1	-1	-1	
M00572: Pimeloyl-ACP_biosynthesis,_BioC-BioH_pathway,_malonyl-ACP_=>_pimeloyl-ACP	0.6	0.6	0.6	0.7	0.6	0.6	0.6	0.6	0.6	0.6	0.6	0.6	0.4	0.5	0.6	0.6	0.5	0.6	0.6	0.6	0.6	0.6	0.6	0.6	0.6	0.6	0.6	0.6	0.6	0.6	0.6	0.6	0.7	0.6	0.6	0.5	0.5	0.4	0.5	0.5	0.6	0.5	0.5	0.5	0.6	0.4	0.6	0.6	0.6	0.6	0.6	0.6	0.6	0.6	0.6	0.6	0.6	0.6	0.7	0.6	0.6	0.7	0.6	0.6	0.6	0	0	0	0	0	0	0	0	0	0	0	0	
M00573: Biotin_biosynthesis,_BioI_pathway,_long-chain-acyl-ACP_=>_pimeloyl-ACP_=>_biotin	0.666667	0.666667	0.666667	0.666667	0.666667	0.666667	0.666667	0.666667	0.666667	0.666667	0.666667	0.666667	0.666667	0.666667	0.666667	0.666667	0.666667	0.666667	0.666667	0.666667	0.666667	0.666667	0.666667	0.666667	0.666667	0.666667	0.666667	0.666667	0.666667	0.666667	0.666667	0.666667	0.666667	0.666667	0.666667	0.666667	0.333333	0.666667	0.666667	0.333333	0.666667	0.333333	0.333333	0.666667	0.666667	0.666667	0.5	0.666667	0.666667	0.666667	0.666667	0.666667	0.666667	0.666667	0.666667	0.666667	0.666667	0.666667	0.666667	0.666667	0.666667	0.666667	0.666667	0.666667	0.666667	0	0	0	0	0	0	0	0	0	0	0	0	
M00574: Pertussis_pathogenicity_signature_1,_pertussis_toxin	0	0	0	0	0	0	0	0	0	0	0	0	0	0	0	0	0	0	0	0	0	0	0	0	0	0	0	0	0	0	0	0	0	0	0	0	0	0	0	0	0	0	0	0	0	0	0	0	0	0	0	0	0	0	0	0	0	0	0	0	0	0	0	0	0	-1	-1	-1	-1	-1	-1	-1	-1	-1	-1	-1	-1	
M00575: Pertussis_pathogenicity_signature_2,_T1SS	0.2	0.2	0.2	0.2	0.2	0.2	0.2	0.2	0.2	0.2	0.2	0.2	0.2	0	0.2	0.2	0.2	0.2	0.2	0.2	0.2	0.2	0.2	0.2	0.2	0.2	0.2	0.2	0.2	0.2	0.2	0.2	0.2	0.2	0.2	0.2	0	0.2	0.2	0.2	0.2	0.2	0.2	0.2	0.2	0.2	0.2	0.2	0.2	0.8	0.2	0.2	0.2	0.2	0.2	0.2	0.2	0.2	0.2	0.2	0.2	0.2	0.2	0.2	0.2	0	0	0	0	0	0	0	0	0	0	0	0	
M00577: Biotin_biosynthesis,_BioW_pathway,_pimelate_=>_pimeloyl-CoA_=>_biotin	0.666667	0.666667	0.666667	0.666667	0.666667	0.666667	0.666667	0.666667	0.666667	0.666667	0.666667	0.666667	0.666667	0.666667	0.666667	0.666667	0.666667	0.666667	0.666667	0.666667	0.666667	0.666667	0.666667	0.666667	0.666667	0.666667	0.666667	0.666667	0.666667	0.666667	0.666667	0.666667	0.666667	0.666667	0.666667	0.666667	0.333333	0.666667	0.666667	0.333333	0.666667	0.333333	0.333333	0.666667	0.666667	0.666667	0.5	0.666667	0.666667	0.666667	0.666667	0.666667	0.666667	0.666667	0.666667	0.666667	0.666667	0.666667	0.666667	0.666667	0.666667	0.666667	0.666667	0.666667	0.666667	0	0	0	0	0	0	0	0	0	0	0	0	
M00579: Phosphate_acetyltransferase-acetate_kinase_pathway,_acetyl-CoA_=>_acetate	0.5	0.5	0.5	0.5	0.5	0.75	0.75	0.5	0.75	0.5	0.75	0.75	0.5	0.5	0.75	0.5	0.5	0.75	0.75	0.5	0.5	0.75	0.5	0.5	0.75	0.75	0.75	0.75	0.5	0.5	0.75	0.5	0.5	0.5	0.5	0.5	0	0.5	0.25	0.25	0.5	0.25	0.25	0.5	0.5	0.5	0.25	0.75	0.75	0.75	0.75	0.75	0.75	0.75	0.5	0.5	0.5	0.5	0.5	0.75	0.5	0.5	0.5	0.75	0.75	0	0	0	0	0	0	0	0	0	0	0	0	
M00580: Pentose_phosphate_pathway,_archaea,_fructose_6P_=>_ribose_5P	0.2	0.2	0.2	0.2	0.2	0.2	0.2	0.2	0	0	0.2	0	0.4	0.4	0.2	0.2	0.2	0.2	0.2	0.2	0.2	0.2	0.2	0.2	0.2	0.2	0.2	0.2	0.2	0.2	0.2	0.2	0.2	0.2	0.2	0.2	0	0.2	0.2	0.2	0.2	0.2	0.2	0.2	0.2	0.2	0.2	0.2	0.2	0.2	0.2	0.2	0.2	0.2	0.2	0.2	0.2	0.2	0.2	0.2	0.2	0.2	0.2	0.2	0.2	0	0	0	0	0	0	0	0	0	0	0	0	
M00581: Biotin_transport_system	0.333333	0.333333	0.333333	0.333333	1	0.333333	1	1	0.333333	0.333333	0.333333	1	1	1	0.333333	0.333333	0.333333	0.333333	1	0.333333	0.333333	0.333333	0.333333	0.333333	0.333333	0.333333	0.333333	0.333333	0	0	0	0	0	0	0	0.333333	0	1	0.333333	0.333333	0.333333	0.333333	0	0.333333	0.333333	0.333333	0.333333	0.333333	0.666667	0.333333	0.333333	0.333333	1	1	0.333333	1	0.333333	0.333333	0.333333	0.333333	1	1	1	1	0.333333	0	0	0	0	-1	0	0	0	0	0	0	1	varied
M00582: Energy-coupling_factor_transport_system	0.090909	0.090909	0.272727	0.090909	0.363636	0.272727	0.272727	0.272727	0.272727	0.272727	0.272727	0.272727	0.363636	0.363636	0.272727	0.272727	0.363636	0.363636	0.545455	0.272727	0.272727	0.272727	0.272727	0.272727	0.272727	0.272727	0.272727	0.272727	0.181818	0.181818	0.181818	0.181818	0.181818	0	0.181818	0.272727	0	0.454545	0.090909	0.090909	0.090909	0.272727	0	0.272727	0.090909	0.363636	0.090909	0.090909	0.090909	0.181818	0.090909	0.090909	0.090909	0.090909	0.272727	0.090909	0.363636	0.272727	0.272727	0.454545	0.272727	0.090909	0.090909	0.272727	0.272727	-1	0	0	0	0	0	-1	0	-1	0	0	0	
M00585: L-Cystine_transport_system	0	0	0	0	0	0	0	0	0	0	0	0	0	0	0	0	0	0	0	0	0	0	0	0	0	0	0	0	0	0	0	0	0	0	0	0	0	0	0	0	0	0	0	0	0	0	0	0	0	0	0	0	0	0	0	0	0	0	0	0	0	0	0	0	0	-1	-1	-1	-1	-1	-1	-1	-1	-1	-1	-1	-1	
M00586: Putative_amino-acid_transport_system	0	0	0	0	0	0	0	0	0	0	0	0	0	0	0	0	0	0	0	0	0	0	0	0	0	0	0	0	0	0	0	0	0	0	0	0	0	0	0	0	0	0	0	0	0	0	0	0	0	0	0	0	0	0	0	0	0	0	0	0	0	0	0	0	0	-1	-1	-1	-1	-1	-1	-1	-1	-1	-1	-1	-1	
M00587: Arginine/lysine/histidine/glutamine_transport_system	0	0	0	0	0	0	0	0	0	0	0	0	0	0	0	0	0	0	0	0	0	0	0	0	0	0	0	0	0	0	0	0	0	0	0	0	0	0	0	0	0	0	0	0	0	0	0	0	0	0	0	0	0	0	0	0	0	0	0	0	0	0	0	0	0	-1	-1	-1	-1	-1	-1	-1	-1	-1	-1	-1	-1	
M00589: Putative_lysine_transport_system	0	0	0	0	0	0	0	0	0	0	0	0	0	0	0	0	0	0	0	0	0	0	0	0	0	0	0	0	0	0	0	0	0	0	0	0	0	0	0	0	0	0	0	0	0	0	0	0	0	0	0	0	0	0	0	0	0	0	0	0	0	0	0	0	0	-1	-1	-1	-1	-1	-1	-1	-1	-1	-1	-1	-1	
M00590: Erythritol_transport_system	0	0	0	0	0	0	0	0	0	0	0	0	0	0	0	0	0	0	0	0	0	0	0	0	0	0	0	0	0	0	0	0	0	0	0	0	0	0	0	0	0	0	0	0	0	0	0	0.333333	0	0	0	0	0.333333	0	0	0	0	0	0	0	0	0	0	0	0	-1	-1	-1	-1	-1	-1	-1	-1	-1	-1	-1	-1	
M00591: Putative_xylitol_transport_system	0	0	0	0	0	0	0	0	0	0	0	0	0	0	0	0	0	0	0	0	0	0	0	0	0	0	0	0	0	0	0	0	0	0	0	0	0	0	0	0	0	0	0	0	0	0	0	0	0	0	0	0	0	0	0	0	0	0	0	0	0	0	0	0	0	-1	-1	-1	-1	-1	-1	-1	-1	-1	-1	-1	-1	
M00592: Inositol_transport_system	0	0	0	0	0	0	0	0	0	0	0	0	0	0	0	0	0	0	0	0	0	0	0	0	0	0	0	0	0	0	0	0	0	0	0	0	0	0	0	1	0	0	1	0	0	0	1	0	0	0	0	0	0	0	0	0	0	0	0	0	0	0	0	0	0	-1	-1	-1	-1	-1	-1	0	-1	-1	-1	-1	-1	
M00593: Inositol_transport_system	0	0	0	0	0	0	0	0	0	0	0	0	0	0	0	0	0	0	0	0	0	0	0	0	0	0	0	0	0	0	0	0	0	0	0	0	0	0	0	0	0	0	0	0	0	0	0	0	0	1	0	0	0	0	0	0	0	0	0	0	0	0	0	0	0	-1	-1	-1	-1	-1	-1	-1	-1	-1	-1	-1	-1	
M00595: Thiosulfate_oxidation_by_SOX_complex,_thiosulfate_=>_sulfate	0.285714	0.285714	0.428571	0.857143	0.142857	0.285714	0.142857	0.142857	0.142857	0.142857	0.142857	0.142857	0.142857	0.142857	0.142857	0.142857	0.142857	0.142857	0.142857	0.142857	0.142857	0.142857	0.142857	0.142857	0.142857	0.142857	0.142857	0.142857	0.142857	0.142857	0.142857	0.142857	0.142857	0.142857	0.142857	0.142857	0.142857	0.142857	0.285714	0.142857	0.142857	0.142857	0.142857	0.142857	0.142857	0	0.142857	0.142857	0.142857	0.857143	0.142857	0.142857	0.142857	0.142857	0.285714	0.285714	0.142857	0.285714	0.285714	0.142857	0.285714	0.285714	0.142857	0.285714	0.142857	0	0	0	0	0	0	0	0	0	0	0	0	
M00596: Dissimilatory_sulfate_reduction,_sulfate_=>_H2S	0.285714	0.285714	0.285714	0.428571	0.428571	0.428571	0.285714	0.428571	0.428571	0.285714	0.428571	0.285714	0.428571	0.428571	0.428571	0.285714	0.428571	0.428571	0.428571	0.428571	0.428571	0.428571	0.428571	0.428571	0.428571	0.285714	0.285714	0.428571	0.428571	0.428571	0.428571	0.428571	0.428571	0.428571	0.428571	0.142857	0.142857	0.428571	0.142857	0.142857	0.285714	0.285714	0.142857	0.285714	0.285714	0.285714	0.285714	0.285714	0.285714	0.285714	0.285714	0.285714	0.285714	0.285714	0.285714	0.285714	0.571429	0.428571	0.428571	0.857143	0.285714	0.285714	0.285714	0.428571	1	0	0	0	0	0	0	0	0	0	0	0	0	
M00597: Anoxygenic_photosystem_II	0	0	0	0	0	0	0	0	0	0	0	0	0	0	0	0	1	0	0	0	0	0	0	0	0	0	0	0	0	0	0	0	0	0	0	0	0	0	0	0	0	0	0	0	0	0	0	0	0	0	0	0	0	0	0	0	0	0	0	0	0	0	0	0	0	-1	-1	-1	-1	-1	-1	-1	-1	-1	-1	-1	-1	
M00599: Inositol-phosphate_transport_system	0	0	0	0	0	0	0	0	0	0	0	0	0	0	0	0	0	0	0	0	0	0	0	0	0	0	0	0	0	0	0	0	0	0	0	0	0	0	0	0	0	0	0	0	0	0	0	0	0	0	0	0	0	0	0	0	0	0	0	0	0	0	0	0	0	-1	-1	-1	-1	-1	-1	-1	-1	-1	-1	-1	-1	
M00600: alpha-1,4-Digalacturonate_transport_system	0	0	0	0	0	0	0	0	0	0	0	0	0	0	0	0	0	0	0	0	0	0	0	0	0	0	0	0	0	0	0	0	0	0	0	0	0	0	0	0	0	0	0	0	0	0	0	0	0	0	0	0	0	0	0	0	0	0	0	0	0	0	0	0	0	-1	-1	-1	-1	-1	-1	-1	-1	-1	-1	-1	-1	
M00601: Putative_chitobiose_transport_system	0	0	0	0	0	0	0	0	0	0	0	0	0	0	0	0	1	0	0	0	0	0	0	0	0	0	0	0	0	0	0	0	0	0	0	0	0	0	0	0	0	0	0	0	0	0	0	0	0	0	0	0	0	0	0	0	0	0	0	0	0	0	0	0	0	-1	-1	-1	-1	-1	-1	-1	-1	-1	-1	-1	-1	
M00602: Arabinosaccharide_transport_system	0	0	0	0	0.25	0.25	0.25	0.25	0	0	0	0.25	0.25	0	0.25	0	0.25	0.25	0.25	0.25	0.25	0.25	0.25	0.25	0.25	0.25	0.25	0.25	0.25	0.25	0.25	0.25	0.25	0.25	0.25	0	0	0	0.25	0	0.25	0	0	0	0	0	0	0	0.25	0.25	0	0	0.25	0	0.25	0	0.25	0.25	0.25	0.25	0.25	0	0	0.25	0	-1	0	0	0	0	-1	-1	-1	-1	0	0	-1	
M00603: Putative_aldouronate_transport_system	0	0	0	0	0	0	0	0	0	0	0	0	0	0	0	0	1	1	1	0	0.333333	1	1	0	0	0	0	0	0	0	0	0	0	0	0	0	0	1	0	0	0	0.666667	0	0	0	1	0	0	0	0	0	0	0	0	0	0	0	0	0	0	0	0	0	0	0	-1	-1	1	-1	-1	-1	-1	-1	-1	-1	-1	-1	varied
M00604: Trehalose_transport_system	0	0	0	0	0	0	0	0	0	0	0	0	0	0	0	0	0	0	0	0	0	0	0	0	0	0	0	0	0	0	0	0	0	0	0	0	0	0	0	0	0	0	0	0	0	0	0	0	0	0	0	0	0	0	0	0	0	0	0	0	0	0	0	0	0	-1	-1	-1	-1	-1	-1	-1	-1	-1	-1	-1	-1	
M00605: Glucose/mannose_transport_system	0.25	0.25	0.25	0.25	0.25	0.25	0.25	0.25	0.25	0.25	0.25	0.25	0.25	0.25	0.25	0.25	0.25	0.25	0.25	0.25	0.25	0.25	0.25	0.25	0.25	0.25	0.25	0.25	0.25	0.25	0.25	0.25	1	1	0.25	0.25	0	0.25	0.25	0.25	0.25	0.25	0.25	0.25	0.25	1	0	1	0.5	0.25	0.25	0.25	0.5	0.5	0.25	0	0.25	0.25	0.25	0.25	0.25	0	0	0.25	0.25	0	0	0	0	0	0	0	0	0	0	0	0	
M00606: N,N'-Diacetylchitobiose_transport_system	0.25	0	0	0.25	0.25	0.25	0.25	0.25	0.25	0.25	0.25	0.25	0.25	0.25	0.25	0.25	0.25	0.25	0.25	0.25	0.25	0.25	0.25	0.25	0.25	0.25	0.25	0.25	0.25	0	0.25	0	0.25	0	0	0.25	0	0.5	0.25	0.25	0.25	0.25	0.25	0.25	0.25	0.25	0	0	0.25	0	0	0	0	0.25	0	0	0	0	0	0	0.25	0	0	0	0	0	0	0	0	-1	0	0	0	-1	-1	-1	-1	
M00607: Glycerol_transport_system	0	0	0	0	0	0	0	0	0	0	0	0	0	0	0	0	0	0	0	0	0	0	0	0	0	0	0	0	0	0	0	0	0	1	0	0	0	0	0	0	0	0	0	0	0	0	0	0.8	1	0.8	0.6	0.8	0.8	1	0	1	0	0	0	0	1	1	1	1	0	-1	-1	-1	-1	-1	-1	-1	-1	0	-1	-1	1	varied
M00608: 2-Oxocarboxylic_acid_chain_extension,_2-oxoglutarate_=>_2-oxoadipate_=>_2-oxopimelate_=>_2-oxosuberate	0	0	0	0	0	0	0	0	0	0	0	0	0	0	0	0	0.5	0	0	0	0	0	0	0	0	0	0	0	0	0	0	0	0	0	0	0	0	0	0	0	0	0	0	0	0	0	0	0	0	0	0	0	0	0	0	0	0	0	0	0	0	0	0	0	0	-1	-1	-1	-1	-1	-1	-1	-1	-1	-1	-1	-1	
M00609: Cysteine_biosynthesis,_methionine_=>_cysteine	0.428571	0.428571	0.285714	0.285714	0.285714	0.285714	0.285714	0.285714	0.428571	0.285714	0.285714	0.285714	0.428571	0.428571	0.285714	0.285714	0.285714	0.285714	0.285714	0.285714	0.285714	0.285714	0.285714	0.285714	0.285714	0.285714	0.142857	0.285714	0.142857	0.142857	0.142857	0.142857	0.142857	0.142857	0.142857	0.285714	0.285714	0.285714	0.285714	0.285714	0.285714	0.285714	0.285714	0.285714	0.285714	0.285714	0.285714	0.285714	0.285714	0.285714	0.285714	0.285714	0.285714	0.285714	0.285714	0.285714	0.285714	0.285714	0.285714	0.285714	0.285714	0.285714	0.285714	0.285714	0.285714	0	0	0	0	0	0	0	0	0	0	0	0	
M00610: PTS_system,_D-glucosaminate-specific_II_component	0	0	0	0	0	0	0	0	0	0	0	0	0	0	0	0	0	0	0	0	0	0	0	0	0	0	0	0	0	0	0	0	0	0	0	0	0	0	0	0	0	0	0	0	0	0	0	0	0	0	0	0	0	0	0	0	0	0	0	0	0	0	0	0	0	-1	-1	-1	-1	-1	-1	-1	-1	-1	-1	-1	-1	
M00611: Oxygenic_photosynthesis_in_plants_and_cyanobacteria	0.333333	0.3	0.333333	0	0.266667	0.266667	0.266667	0.3	0.2	0.233333	0	0	0.233333	0	0.266667	0	0.366667	0.333333	0.4	0.3	0.4	0.3	0.4	0.433333	0.4	0.266667	0.366667	0.266667	0.3	0.266667	0.233333	0.266667	0.233333	0.266667	0.266667	0.333333	0	0.233333	0	0	0	0.3	0	0.333333	0	0	0	0.266667	0	0.233333	0	0	0	0.3	0.3	0.266667	0.3	0.366667	0.3	0.266667	0.333333	0	0.2	0.3	0.233333	0	0	0	0	0	0	-1	-1	-1	0	0	0	
M00612: Anoxygenic_photosynthesis_in_purple_bacteria	0.5	0.45	0.5	0.5	0.4	0.4	0.4	0.45	0.3	0.35	0.4	0.35	0.35	0.35	0.4	0.35	0.65	0.5	0.6	0.45	0.55	0.45	0.55	0.55	0.5	0.4	0.55	0.4	0.45	0.4	0.35	0.4	0.35	0.4	0.4	0.5	0.45	0.35	0.5	0.5	0.5	0.45	0.45	0.5	0.55	0.35	0.45	0.4	0.4	0.35	0.4	0.4	0.4	0.45	0.45	0.4	0.45	0.55	0.45	0.4	0.5	0.4	0.3	0.45	0.35	0	0	0	0	0	0	0	0	0	0	0	0	
M00613: Anoxygenic_photosynthesis_in_green_nonsulfur_bacteria	0.384615	0.410256	0.384615	0.410256	0.384615	0.435897	0.358974	0.384615	0.358974	0.384615	0.410256	0.384615	0.333333	0.333333	0.410256	0.333333	0.692308	0.435897	0.461538	0.461538	0.410256	0.384615	0.487179	0.410256	0.410256	0.487179	0.410256	0.487179	0.410256	0.358974	0.384615	0.358974	0.307692	0.333333	0.358974	0.435897	0.384615	0.410256	0.384615	0.384615	0.410256	0.384615	0.410256	0.461538	0.410256	0.358974	0.410256	0.358974	0.358974	0.384615	0.358974	0.358974	0.358974	0.384615	0.333333	0.358974	0.461538	0.487179	0.384615	0.487179	0.358974	0.358974	0.358974	0.410256	0.435897	0	0	0	0	0	0	0	0	0	0	0	0	
M00614: Anoxygenic_photosynthesis_in_green_sulfur_bacteria	0.428571	0.452381	0.47619	0.428571	0.452381	0.452381	0.404762	0.452381	0.404762	0.5	0.428571	0.428571	0.428571	0.428571	0.428571	0.380952	0.428571	0.5	0.452381	0.595238	0.547619	0.428571	0.547619	0.5	0.47619	0.690476	0.619048	0.690476	0.52381	0.452381	0.547619	0.47619	0.47619	0.380952	0.47619	0.404762	0.357143	0.404762	0.333333	0.404762	0.404762	0.357143	0.380952	0.404762	0.404762	0.380952	0.380952	0.380952	0.380952	0.380952	0.380952	0.380952	0.380952	0.380952	0.452381	0.380952	0.666667	0.547619	0.547619	0.619048	0.452381	0.380952	0.333333	0.452381	0.642857	0	0	0	0	0	0	0	0	0	0	0	0	
M00615: Nitrate_assimilation	0.3	0.5	0.5	0.5	0.2	0.5	0.3	0.2	0.2	0.2	0.2	0.2	0.3	0.3	0.3	0.2	0.2	0.5	0.2	0.5	0.6	0.3	0.5	0.5	0.6	0.4	0.3	0.4	0.3	0.3	0.3	0.3	0.3	0.5	0.3	0.5	0.5	0.2	0.3	0.5	0.5	0.4	0.5	0.5	0.5	0.3	0.5	0.5	0.5	0.5	0.5	0.5	0.5	0.5	0.5	0.5	0.1	0.5	0.5	0.1	0.5	0.5	0.5	0.5	0.2	0	0	0	0	0	0	0	0	0	0	0	0	
M00616: Sulfate-sulfur_assimilation	0.75	0.625	0.625	0.6875	0.4375	0.625	0.5625	0.5625	0.4375	0.25	0.5	0.4375	0.3125	0.3125	0.625	0.5	0.8125	0.8125	0.8125	0.8125	0.8125	0.8125	0.8125	0.8125	0.8125	0.6875	0.6875	0.75	0.6875	0.625	0.625	0.75	0.6875	0.75	0.75	0.5625	0.5625	0.6875	0.5625	0.5625	0.625	0.75	0.5625	0.6875	0.625	0.5625	0.625	0.625	0.625	0.6875	0.625	0.625	0.625	0.6875	0.5	0.5625	0.3125	0.625	0.625	0.5	0.5625	0.5625	0.5625	0.6875	0.625	0	0	0	0	0	0	0	0	0	0	0	0	
M00617: Methanogen	0.1	0.08	0.1	0.06	0.16	0.18	0.14	0.16	0.18	0.16	0.18	0.16	0.16	0.16	0.18	0.14	0.08	0.14	0.16	0.14	0.14	0.08	0.12	0.12	0.1	0.18	0.18	0.18	0.16	0.14	0.18	0.16	0.16	0.08	0.14	0.06	0.02	0.06	0.04	0.04	0.06	0.04	0.04	0.06	0.06	0.06	0.04	0.08	0.08	0.08	0.08	0.08	0.08	0.08	0.14	0.08	0.2	0.16	0.14	0.22	0.1	0.06	0.06	0.16	0.22	-1	0	0	0	0	-1	-1	-1	-1	0	0	0	
M00618: Acetogen	0.357143	0.285714	0.285714	0.285714	0.571429	0.642857	0.357143	0.5	0.642857	0.571429	0.642857	0.428571	0.571429	0.5	0.642857	0.357143	0.428571	0.5	0.5	0.5	0.5	0.5	0.428571	0.428571	0.5	0.642857	0.642857	0.642857	0.571429	0.5	0.642857	0.571429	0.571429	0.357143	0.357143	0.357143	0.142857	0.357143	0.285714	0.214286	0.285714	0.357143	0.214286	0.357143	0.285714	0.428571	0.214286	0.357143	0.357143	0.357143	0.357143	0.357143	0.357143	0.357143	0.285714	0.285714	0.785714	0.5	0.5	0.857143	0.357143	0.285714	0.285714	0.357143	0.857143	0	0	0	0	0	0	0	0	0	0	0	0	
M00620: Incomplete_reductive_citrate_cycle,_acetyl-CoA_=>_oxoglutarate	0.294118	0.352941	0.411765	0.294118	0.411765	0.411765	0.294118	0.411765	0.411765	0.647059	0.411765	0.411765	0.411765	0.411765	0.411765	0.411765	0.294118	0.470588	0.294118	0.705882	0.588235	0.294118	0.529412	0.470588	0.352941	0.823529	0.647059	0.823529	0.588235	0.411765	0.647059	0.470588	0.470588	0.294118	0.470588	0.294118	0.294118	0.294118	0.294118	0.352941	0.294118	0.294118	0.294118	0.294118	0.294118	0.294118	0.294118	0.176471	0.176471	0.176471	0.176471	0.176471	0.176471	0.176471	0.411765	0.294118	0.823529	0.529412	0.588235	0.823529	0.411765	0.294118	0.176471	0.411765	0.823529	0	0	0	0	0	0	0	0	0	0	0	0	
M00622: Nicotinate_degradation,_nicotinate_=>_fumarate	0	0	0	0	0	0	0	0	0	0	0	0	0	0	0	0	0	0	0	0	0	0	0	0	0	0	0	0	0	0	0	0	0	0.142857	0	0	0	0	0	0	0	0	0	0	0	0	0	0.142857	0.142857	0.571429	0.142857	0.142857	0.142857	0.285714	0	0	0	0	0	0	0	0	0	0	0	-1	-1	-1	-1	-1	-1	-1	-1	0	-1	-1	-1	
M00623: Phthalate_degradation,_phthalate_=>_protocatechuate	0	0	0	0	0	0	0	0	0	0	0	0	0	0	0	0	0	0	0	0	0	0	0	0	0	0	0	0	0	0	0	0	0	0	0	0	0	0	0	0	0	0	0	0	0	0	0	0	0	0	0	0	0	0	0	0	0	0	0	0	0	0	0	0	0	-1	-1	-1	-1	-1	-1	-1	-1	-1	-1	-1	-1	
M00624: Terephthalate_degradation,_terephthalate_=>_3,4-dihydroxybenzoate	0	0	0	0	0	0	0	0	0	0	0	0	0	0	0	0	0	0	0	0	0	0	0	0	0	0	0	0	0	0	0	0	0	0	0	0	0	0	0	0	0	0	0	0	0	0	0	0	0	0	0	0	0	0	0	0	0	0	0	0	0	0	0	0	0	-1	-1	-1	-1	-1	-1	-1	-1	-1	-1	-1	-1	

Table S4. V

KEGG Module	Habitat																																																																	1 if median >= 90% for the samples per site; -1 if median <= 10% for the samples per site												Kind of Module inferred (Core or Varied)
	India oil refineries				Arctic												China oil refineries									Mangrove			Marine sediments							Taiga upper active layer				Taiga bottom active layer				Taiga permafrost layer				Oil sands core							Oil sands tailings pond upper				Oil sands tailings pond median			Oil sands tailings pond deep																
	I1	I2	I3	I4	A1	A10	A11	A12	A2	A3	A4	A5	A6	A7	A8	A9	C1	C2	C3	C4	C5	C6	C7	C8	C9	M1	M2	M3	DWH1	DWH2	DWH3	DWH4	DWH5	DWH6	DWH7	Tu1	Tu2	Tu3	Tu4	Tb1	Tb2	Tb3	Tb4	Tp1	Tp2	Tp3	Tp4	OSC1	OSC12	OSC3	OSC4	OSC5	OSC7	OSC9	OSTPu1	OSTPu2	OSTPu4	OSTPu3	OSTPm2	OSTPm6	OSTPm4	OSTPd1	OSTPd2	OSTPd3	OSTPd4	India oil refineries	Arctic	China oil refineries	Mangrove	Marine sediments	Taiga upper active layer	Taiga bottom active layer	Taiga permafrost layer	Oil sands core	Oil sands tailings pond upper	Oil sands tailings pond median	Oil sands tailings pond deep	
M00625: Methicillin_resistance	0	0	0	0	0	0	0	0	0	0	0	0	0	0	0	0	0	0	0	0	0	0	0	0	0	0	0	0	0	0	0	0	0	0	0	0	0	0	0	0	0	0	0	0	0	0	0	0	0	0	0	0	0	0	0	0	0	0	0	0	0	0	0	0	0	-1	-1	-1	-1	-1	-1	-1	-1	-1	-1	-1	-1	
M00627: beta-Lactam_resistance,_Bla_system	0.25	0.25	0.25	0.25	0.25	0.25	0.25	0.25	0.25	0.25	0.25	0.25	0.25	0.25	0.25	0.25	0.25	0.25	0.25	0.25	0.25	0.25	0.25	0.25	0.25	0.25	0.25	0.25	0.25	0.25	0.25	0.25	0.25	0.25	0.25	0.25	0.25	0.25	0.25	0.5	0.25	0.25	0.5	0.25	0.25	0.25	0.5	0.25	0.25	0.25	0.25	0.25	0.25	0.25	0	0.25	0.25	0.25	0.25	0.25	0.25	0.25	0	0.25	0.25	0	0	0	0	0	0	0	0	0	0	0	0	
M00628: beta-Lactam_resistance,_AmpC_system	0.6	0.6	0.6	0.6	0.6	0.6	0.6	0.6	0.6	0.6	0.6	0.6	0.6	0.6	0.6	0.6	0.6	0.6	0.6	0.6	0.6	0.8	0.6	0.6	0.6	0.6	0.6	0.6	0.6	0.6	0.6	0.6	0.6	0.6	0.6	0.6	0.6	0.4	0.6	0.6	0.6	0.6	0.6	0.6	0.6	0.4	0.6	0.8	0.8	0.6	0.8	0.8	0.8	0.8	0.6	0.8	0.6	0.6	0.6	0.6	0.8	0.8	0.8	1	0.6	0	0	0	0	0	0	0	0	0	0	0	0	
M00631: Other_carbohydrate_metabolism|D-Galacturonate_degradation_(bacteria)	0.5	0.375	0.125	0.125	0.25	0.125	0.25	0.25	0.5	0.25	0.25	0.375	0.25	0.25	0.25	0.5	0.375	0.5	0.375	0.5	0.5	0.375	0.5	0.5	0.375	0.25	0.25	0.25	0.125	0.125	0.125	0.125	0.125	0.25	0.125	0.5	0.5	0.375	0.5	0.5	0.5	0.5	0.5	0.5	0.25	0.25	0.5	0.5	0.5	0.375	0.5	0.5	0.5	0.5	0.125	0.125	0.125	0	0	0.25	0.125	0.125	0.125	0.125	0.125	0	0	0	0	0	0	0	0	0	0	0	0	
M00632: Galactose_degradation,_Leloir_pathway,_galactose_=>_alpha-D-glucose-1P	1	1	1	1	1	0.75	1	1	1	1	1	1	1	1	1	1	1	1	1	1	1	1	1	1	1	1	0.75	0.75	0.75	0.5	0.5	0.5	0.5	0.75	0.5	1	1	1	1	0.5	1	1	1	1	1	1	1	0.5	0.5	0.5	0.5	0.5	0.5	1	0.25	0.25	0.5	0.5	0.5	0.75	0.5	0.25	0.25	0.5	0.5	1	1	1	0	0	1	1	1	0	0	0	0	
M00633: Semi-phosphorylative_Entner-Doudoroff_pathway,_gluconate/galactonate_=>_glycerate-3P	0	0	0	0	0	0	0	0	0	0	0	0	0	0	0	0	0	0	0	0.2	0	0	0	0	0	0	0	0	0	0	0	0	0	0	0	0	0	0	0	0	0	0	0	0	0	0.2	0	0	0	0	0	0	0	0	0	0	0	0	0	0	0	0	0	0	0	-1	-1	-1	-1	-1	-1	-1	-1	-1	-1	-1	-1	
M00634: Oleandomycin_transport_system	0	0	0	0	0	0	0	0	0	0	0	0	0	0	0	0	0	0	0	0	0	0	0	0	0	0	0	0	0	0	0	0	0	0	0	0	0	1	0	0	0	0	0	0	0	0	0	0	0	0	0	0	0	0	0	0	0	0	0	0	0	0	0	0	0	-1	-1	-1	-1	-1	-1	-1	-1	-1	-1	-1	-1	
M00635: Tetracycline_resistance,_TetAB(46)_transporter	0	0	0	0	0	0	0	0	0	0	0	0	0	0	0	0	0	0	0	0	0	0	0	0	0	0	0	0	0	0	0	0	0	0	0	0	0	0	0	0	0	0	0	0	0	0	0	0	0	0	0	0	0	0	0	0	0	0	0	0	0	0	0	0	0	-1	-1	-1	-1	-1	-1	-1	-1	-1	-1	-1	-1	
M00636: Phthalate_degradation,_phthalate_=>_protocatechuate	0	0	0	0	0	0	0	0	0	0	0	0	0	0	0	0	0	0	0	0	0	0	0	0	0	0	0	0	0	0	0	0	0	0	0	0	0	0	0	0	0	0	0	0	0	0	0	0	0	0	0	0	0	0	0	0	0	0	0	0	0	0	0	0	0	-1	-1	-1	-1	-1	-1	-1	-1	-1	-1	-1	-1	
M00637: Anthranilate_degradation,_anthranilate_=>_catechol	0	0	0	0	0	0	0	0	0	0	0	0	0	0	0	0	0	0	0	0	0	0	0	0	0	0	0	0	0	0	0	0	0	0	0	0	0	0	0	0	0	0	0	0	0	0	0.142857	0	0	0	0	0	0	0	0	0	0	0	0	0	0	0	0	0	0	-1	-1	-1	-1	-1	-1	-1	-1	-1	-1	-1	-1	
M00638: Salicylate_degradation,_salicylate_=>_gentisate	0	0	0	0	0	0	0	0	0	0	0	0	0	0	0	0	0	0	0	0	0	0	0	0	0	0	0	0	0	0	0	0	0	0	0	0	0	0	0	0	0	0	0	0	0	0	0	0	0	1	0	0	0	0	0	0	0	0	0	0	0	0	0	0	0	-1	-1	-1	-1	-1	-1	-1	-1	-1	-1	-1	-1	
M00639: Multidrug_resistance,_efflux_pump_MexCD-OprJ	0	0	0	0	0	0	0	0	0	0	0	0	0	0	0	0	0	0	0	0	0	0	0	0	0	0	0	0	0	0	0	0	0	0	0	0	0	0	0	0	0	0	0	0	0	0	0	0	0	0	0	0	0	0	0	0	0	0	0	0	0	0	0	0	0	-1	-1	-1	-1	-1	-1	-1	-1	-1	-1	-1	-1	
M00641: Multidrug_resistance,_efflux_pump_MexEF-OprN	0	0	0	0	0	0	0	0	0	0	0	0	0	0	0	0	0	0	0	0.5	0.5	0	0	0.5	0	0	0	0	0	0	0	0	0	0	0	0	0	0	0	0	0	0	0	0	0	0	0	0	0	0	0	0	0	0	0	0	0	0	0	0	0	0	0	0	0	-1	-1	-1	-1	-1	-1	-1	-1	-1	-1	-1	-1	
M00642: Multidrug_resistance,_efflux_pump_MexJK-OprM	0.25	0.25	0.25	0.25	0.25	0.25	0.25	0.25	0.25	0.25	0.25	0.25	0.25	0.25	0.25	0.25	0.25	0.25	0.25	0.25	0.25	0.25	0.25	0.25	0.25	0.25	0.25	0.25	0.25	0.25	0	0.25	0	0	0.25	0.5	0.5	0	0.25	0.25	0.5	0.25	0.5	0.5	0.5	0.25	0.5	0.25	0.25	0.5	0.25	0.25	0.25	0.25	0.25	0.25	0.25	0.25	0.5	0.25	0.25	0.25	0.25	0.25	0.25	0	0	0	0	0	0	0	0	0	0	0	0	
M00643: Multidrug_resistance,_efflux_pump_MexXY-OprM	0.25	0.25	0	0	0.25	0.25	0.25	0.25	0	0	0	0	0	0.25	0.25	0	0	0	0	0	0.25	0.25	0.25	0.25	0.25	0.25	0.25	0	0	0	0	0	0	0	0	0.25	0	0	0	0	0	0	0	0.25	0	0	0	0.25	0.25	0.25	0.25	0.25	0.25	0.25	0	0	0	0	0	0.25	0.25	0	0	0.25	0.25	0	0	0	0	-1	-1	-1	-1	0	-1	0	0	
M00644: Multidrug_resistance,_efflux_pump_MexHI-OprD	0	0	0	0	0	0	0	0	0	0	0	0	0	0	0	0	0	0	0	0	0	0	0	0	0	0	0	0	0	0	0	0	0	0	0	0	0	0	0	0	0	0	0	0	0	0	0	0	0	0	0	0	0	0	0	0	0	0	0	0	0	0	0	0	0	-1	-1	-1	-1	-1	-1	-1	-1	-1	-1	-1	-1	
M00646: Multidrug_resistance,_efflux_pump_AcrAD-TolC	0.4	0.4	0.6	0.4	0.4	0.4	0.4	0.4	0.4	0.4	0.4	0.4	0.4	0.2	0.4	0.4	0.6	0.6	0.6	0.4	0.4	0.4	0.4	0.4	0.4	0.4	0.4	0.4	0.4	0.4	0.4	0.4	0.4	0.4	0.4	0.4	0.4	0.4	0.4	0.4	0.4	0.4	0.4	0.4	0.4	0.4	0.4	0.4	0.4	0.8	0.4	0.4	0.4	0.6	0.4	0.8	0.4	0.4	0.6	0.6	0.8	0.8	0.8	0.8	0.6	0	0	0	0	0	0	0	0	0	0	0	0	
M00647: Multidrug_resistance,_efflux_pump_AcrAB-TolC/SmeDEF	0.363636	0.363636	0.363636	0.363636	0.363636	0.363636	0.363636	0.363636	0.363636	0.363636	0.363636	0.363636	0.363636	0.272727	0.363636	0.363636	0.363636	0.363636	0.363636	0.363636	0.363636	0.363636	0.363636	0.454545	0.363636	0.363636	0.363636	0.363636	0.363636	0.363636	0.363636	0.363636	0.363636	0.363636	0.363636	0.363636	0.363636	0.272727	0.363636	0.363636	0.363636	0.363636	0.363636	0.363636	0.454545	0.363636	0.363636	0.454545	0.454545	0.545455	0.454545	0.454545	0.454545	0.454545	0.454545	0.454545	0.363636	0.454545	0.454545	0.363636	0.454545	0.454545	0.454545	0.454545	0.363636	0	0	0	0	0	0	0	0	0	0	0	0	
M00648: Multidrug_resistance,_efflux_pump_MdtABC	0.6	0.6	0.2	0	0	0	0	0	0.6	0	0	0.6	0	0	0.6	0.6	0.8	0.8	0.6	0.6	0.6	0.6	0.6	0.6	0.6	0	0	0.6	0.6	0	0	0.4	0	0.2	0.6	0.6	0	0	0.4	0.2	0.6	0	0.2	0.6	0.6	0	0	0.2	0.6	1	0.2	0.4	0.6	0.8	0.2	0.4	0.2	0.6	0.4	0.2	1	0.6	0.4	0.6	0.2	0	-1	0	-1	0	0	0	0	0	0	0	0	
M00649: Multidrug_resistance,_efflux_pump_AdeABC	0	0	0	0	0	0	0	0	0	0	0	0	0	0	0	0	0	0	0	0	0	0	0	0	0	0	0	0	0	0	0	0	0	0	0	0	0	0	0	0	0	0	0	0	0	0	0	0	0	0.8	0	0	0	0	0	0	0	0	0	0	0	0	0	0	0	-1	-1	-1	-1	-1	-1	-1	-1	-1	-1	-1	-1	
M00651: Vancomycin_resistance,_D-Ala-D-Lac_type	0	0	0.090909	0	0.181818	0	0	0	0	0	0	0	0.272727	0.272727	0	0	0.090909	0.090909	0.090909	0.090909	0.090909	0.090909	0.181818	0.090909	0.181818	0.090909	0.090909	0.090909	0.090909	0	0	0	0	0.090909	0	0	0	0.545455	0	0	0	0	0	0	0	0	0	0	0	0	0	0	0	0	0	0	0.090909	0	0	0.090909	0	0	0	0	0	-1	-1	-1	-1	-1	-1	-1	-1	-1	-1	-1	-1	
M00652: Vancomycin_resistance,_D-Ala-D-Ser_type	0	0	0	0	0	0	0	0	0	0	0	0	0	0	0	0	0	0	0	0	0	0	0	0	0	0	0	0	0	0	0	0	0	0	0	0	0	0	0	0	0	0	0	0	0	0	0	0	0	0	0	0	0	0	0	0	0	0	0	0	0	0	0	0	0	-1	-1	-1	-1	-1	-1	-1	-1	-1	-1	-1	-1	
M00653: AauS-AauR_(acidic_amino_acids_utilization)_two-component_regulatory_system	0	0	0	0	0	0	0	0	0	0	0	0	0	0	0	0	0	0	0	0	0	0	0	0	0	0	0	0	0	0	0	0	0	0	0	0	0	0	0	0	0	0	0	0	0	0	0	0	0	0	0	0	0	0	0	0	0	0	0	0	0	0	0	0	0	-1	-1	-1	-1	-1	-1	-1	-1	-1	-1	-1	-1	
M00654: ParS-ParR_(polymyxin-adaptive_resistance)_two-component_regulatory_system	0	0	0	0	0	0	0	0	0	0	0	0	0	0	0	0	0	0	0	0	0	0	0	0	0	0	0	0	0	0	0	0	0	0	0	0	0	0	0	0	0	0	0	0	0	0	0	0	0	0	0	0	0	0	0	0	0	0	0	0	0	0	0	0	0	-1	-1	-1	-1	-1	-1	-1	-1	-1	-1	-1	-1	
M00655: AdeS-AdeR_two-component_regulatory_system	0	0	0	0	0	0	0	0	0	0	0	0	0	0	0	0	0	0	0	0	0	0	0	0	0	0	0	0	0	0	0	0	0	0	0	0	0	0	0	0	0	0	0	0	0	0	0	0	0	1	0	0	0	0	0	0	0	0	0	0	0	0	0	0	0	-1	-1	-1	-1	-1	-1	-1	-1	-1	-1	-1	-1	
M00656: VanS-VanR_(VanB_type_vancomycin_resistance)_two-component_regulatory_system	0	0	0	0	0	0	0	0	0	0	0	0	0	0	0	0	0	0	0	0	0	0	0	0	0	0	0	0	0	0	0	0	0	0	0	0	0	0	0	0	0	0	0	0	0	0	0	0	0	0	0	0	0	0	0	0	0	0	0	0	0	0	0	0	0	-1	-1	-1	-1	-1	-1	-1	-1	-1	-1	-1	-1	
M00657: VanS-VanR_(VanE_type_vancomycin_resistance)_two-component_regulatory_system	0	0	0	0	0	0	0	0	0	0	0	0	0	0	0	0	0	0	0	0	0	0	0	0	0	0	0	0	0	0	0	0	0	0	0	0	0	0	0	0	0	0	0	0	0	0	0	0	0	0	0	0	0	0	0	0	0	0	0	0	0	0	0	0	0	-1	-1	-1	-1	-1	-1	-1	-1	-1	-1	-1	-1	
M00658: VanS-VanR_(actinomycete_type_vancomycin_resistance)_two-component_regulatory_system	0	0	0	0	1	0	0	0	0	0	0	0	1	1	0	0	0	0	0	0	0	0	0	0	0	0	0	0	0	0	0	0	0	0	0	0	0	1	0	0	0	0	0	0	0	0	0	0	0	0	0	0	0	0	0	0	0	0	0	0	0	0	0	0	0	-1	-1	-1	-1	-1	-1	-1	-1	-1	-1	-1	-1	
M00660: Xanthomonas_spp_pathogenicity_signature,_T3SS_and_effectors	0	0	0	0	0	0	0	0	0	0	0	0	0	0	0	0	0	0	0	0	0	0	0	0	0	0	0	0	0	0	0	0	0	0	0	0	0	0	0	0	0	0	0	0	0	0	0	0	0	0.722222	0	0	0	0	0	0	0	0	0	0	0	0	0	0	0	-1	-1	-1	-1	-1	-1	-1	-1	-1	-1	-1	-1	
M00664: Nodulation	0	0	0	0	0	0	0	0	0	0	0	0	0	0	0	0	0	0	0	0	0	0	0	0	0	0	0	0	0	0	0	0	0	0	0	0	0	0	0	0	0	0	0	0	0	0	0	0	0	0	0	0	0	0	0	0	0	0	0	0	0	0	0	0	0	-1	-1	-1	-1	-1	-1	-1	-1	-1	-1	-1	-1	
M00667: Tetracycline_resistance,_efflux_pump	0	0	0	0	0	0	0	0	0	0	0	0	0	0	0	0	0	0	0	0	0	0	0	0	0	0	0	0	0	0	0	0	0	0	0	0	0	0	0	0	0	0	0	0	0	0	0	0	0	0	0	0	0	0	0	0	0	0	0	0	0	0	0	0	0	-1	-1	-1	-1	-1	-1	-1	-1	-1	-1	-1	-1	
M00668: Tetracycline_resistance,_TetA_transporter	0	0	0	0	0.5	0.5	0.5	0.5	0	0	0	0.5	0.5	0.5	0	0	0	0	0	0.5	0	0	0	0	0	0	0	0	0	0	0	0	0	0	0	0.5	0.5	0	0.5	0.5	0.5	0.5	0.5	0.5	0.5	0	0.5	0.5	0	0	0.5	0.5	0.5	0.5	0	0	0	0	0	0	0	0	0	0	0	-1	0	-1	-1	-1	0	0	0	0	-1	-1	-1	
M00669: gamma-Hexachlorocyclohexane_transport_system	1	1	1	1	1	0.75	1	1	0.75	0.75	0.75	0.75	0.75	0.75	0.75	0.75	0.75	0.75	0.75	0.75	0.75	1	0.75	0.75	0.75	0.75	1	1	1	1	1	1	1	0.75	1	1	1	0.75	1	0.75	1	0.75	1	1	1	0.75	1	1	1	1	1	1	1	1	1	1	0.75	1	1	0.75	1	1	1	1	0.75	1	0	0	1	1	1	0	1	1	1	1	1	
M00670: Mce_transport_system	0.75	0.75	0.75	0.75	0.75	0.75	0.75	0.75	0.75	0.75	0.75	0.75	0.75	0.75	0.75	0.75	1	1	1	0.75	0.75	1	1	0.75	1	0.75	0.75	0.75	0.75	0.75	0.75	0.75	0.75	0.75	0.75	0.75	0.75	1	0.75	0.75	0.75	0.75	0.75	0.75	0.75	1	0.75	0.75	0.75	0.75	0.75	0.75	0.75	0.75	0.75	0.75	0.75	0.75	0.75	0.75	0.75	0.75	0.75	0.75	0.75	0	0	1	0	0	0	0	0	0	0	0	0	
M00673: Cephamycin_C_biosynthesis,_aminoadipate_+_cycteine_+_valine_=>_cephamycin_C	0	0	0	0	0	0	0	0	0	0	0	0	0	0	0	0	0.125	0.125	0.125	0.125	0.125	0	0.125	0.125	0	0	0	0	0	0	0	0	0	0	0	0	0	0.125	0	0	0	0	0	0	0	0	0	0	0	0.25	0	0	0	0	0	0	0	0	0	0	0	0	0	0	0	-1	-1	0	-1	-1	-1	-1	-1	-1	-1	-1	-1	
M00694: cGMP_signaling	0	0	0	0	0	0	0	0	0	0	0	0	0	0	0	0	0	0	0	0	0	0	0	0	0	0	0	0	0	0	0	0	0	0	0	0	0	0	0	0	0	0	0	0	0	0	0	0	0	0	0	0	0	0	0	0	0	0	0	0	0	0	0	0	0	-1	-1	-1	-1	-1	-1	-1	-1	-1	-1	-1	-1	
M00695: cAMP_signaling	0.076923	0.076923	0.076923	0.076923	0.076923	0.076923	0.076923	0.076923	0.076923	0.076923	0.076923	0.076923	0.076923	0.076923	0.076923	0.076923	0.076923	0.076923	0.076923	0.076923	0.076923	0.076923	0.076923	0.076923	0.076923	0.076923	0.076923	0.076923	0.076923	0.076923	0.076923	0.076923	0.076923	0.076923	0.076923	0.076923	0.076923	0.076923	0.076923	0.076923	0.076923	0.076923	0.076923	0.076923	0.076923	0.076923	0.076923	0.076923	0.076923	0.076923	0.076923	0.076923	0.076923	0.076923	0.076923	0.076923	0.076923	0.076923	0.076923	0.076923	0.076923	0.076923	0.076923	0.076923	0.076923	-1	-1	-1	-1	-1	-1	-1	-1	-1	-1	-1	-1	
M00696: Multidrug_resistance,_efflux_pump_AcrEF-TolC	0	0	0	0	0	0	0	0	0	0	0	0	0	0	0	0	0	0	0	0	0	0	0	0	0	0	0	0	0	0	0	0	0	0	0	0	0	0	0	0	0	0	0	0	0	0	0	0.25	0.25	0	0.25	0.25	0.25	0.25	0	0	0	0	0	0	0	0	0	0	0	-1	-1	-1	-1	-1	-1	-1	-1	0	-1	-1	-1	
M00697: Multidrug_resistance,_efflux_pump_MdtEF-TolC	0.25	0.25	0.25	0.25	0.25	0.25	0.25	0.25	0.25	0.25	0.25	0.25	0.5	0.25	0.25	0.25	0.25	0.25	0.25	0.25	0.25	0.25	0.25	0.25	0.25	0.25	0.25	0.25	0.25	0	0.25	0.25	0.25	0.25	0.25	0.25	0.25	0.25	0	0.25	0.25	0.25	0.25	0.25	0.25	0.25	0.25	0.25	0.25	0.5	0.25	0.25	0.5	0.5	0.25	0.25	0.25	0.25	0.25	0.25	0.25	0.25	0.25	0.25	0.25	0	0	0	0	0	0	0	0	0	0	0	0	
M00698: Multidrug_resistance,_efflux_pump_BpeEF-OprC	0	0.5	0	0.5	0	0.5	0	0	0	0	0	0	0	0	0	0	0	0	0	0.5	0.5	0	0.5	0.5	0	0	0	0	0	0	0	0	0	0	0	0.75	0.75	0	0.25	0.75	0.75	0.5	0.75	0.75	0.75	0	0.75	0.75	0.75	1	0.75	0.75	0.75	0.75	0.75	0.75	0	0.75	0	0	0.75	0.75	0.75	0.75	0	0	-1	-1	-1	-1	0	0	0	0	0	-1	0	
M00699: Multidrug_resistance,_efflux_pump_AmeABC	0.5	0.5	0.5	0.5	0.5	0.5	0.5	0.5	0.5	0.5	0.5	0.5	0.5	0.5	0.5	0.5	0.5	0.5	0.5	0.5	0.5	0.5	0.5	0.5	0.5	0.5	0.5	0.5	0.5	0.5	0.5	0.5	0.5	0.5	0.5	0.5	0.5	0.5	0.5	0.5	0.5	0.5	0.5	0.5	0.5	0.5	0.5	0.5	0.5	0.5	0.5	0.5	0.5	0.5	0.5	0.5	0.5	0.5	0.5	0.5	0.5	0.5	0.5	0.5	0.5	0	0	0	0	0	0	0	0	0	0	0	0	
M00700: Multidrug_resistance,_efflux_pump_AbcA	0	0	0	0	0	0	0	0	0	0	0	0	0	0	0	0	0	0	0	0	0	0	0	0	0	0	0	0	0	0	0	0	0	0	0	0	0	0	0	0	0	0	0	0	0	0	0	0	0	0	0	0	0	0	0	0	0	0	0	0	0	0	0	0	0	-1	-1	-1	-1	-1	-1	-1	-1	-1	-1	-1	-1	
M00701: Multidrug_resistance,_efflux_pump_EmrAB	0.666667	0.666667	0.666667	0.666667	0.666667	0.666667	0.666667	0.666667	0.666667	0.666667	0.666667	0.666667	0.666667	0.333333	0.666667	0.666667	0.666667	0.666667	0.666667	0.666667	0.666667	0.666667	0.666667	0.666667	0.666667	0.666667	0.666667	0.666667	0.666667	0.666667	0.666667	0.666667	0.666667	0.666667	0.666667	0.666667	0.666667	0.666667	0.666667	0.666667	0.666667	0.666667	0.666667	0.666667	0.666667	0.666667	0.666667	0.666667	0.666667	0.666667	0.666667	0.666667	0.666667	0.666667	0.666667	0.666667	0.666667	0.666667	0.666667	0.666667	0.666667	0.666667	0.666667	0.666667	0.666667	0	0	0	0	0	0	0	0	0	0	0	0	
M00702: Multidrug_resistance,_efflux_pump_NorB	0	0	0	0	0	0	0	0	0	0	0	0	0	0	0	0	0	0	0	0	0	0	0	0	0	0	0	0	0	0	0	0	0	0	0	0	0	0	0	0	0	0	0	0	0	0	0	0	0	0	0	0	0	0	0	0	0	0	0	0	0	0	0	0	0	-1	-1	-1	-1	-1	-1	-1	-1	-1	-1	-1	-1	
M00704: Tetracycline_resistance,_efflux_pump_Tet38	0	0	0	0	0	0	0	0	0	0	0	0	0	0	0	0	0	0	0	0	0	0	0	0	0	0	0	0	0	0	0	0	0	0	0	0	0	0	0	0	0	0	0	0	0	0	0	0	0	0	0	0	0	0	0	0	0	0	0	0	0	0	0	0	0	-1	-1	-1	-1	-1	-1	-1	-1	-1	-1	-1	-1	
M00705: Multidrug_resistance,_efflux_pump_MepA	0	0	0	0	0	0	0	0	0	0	0	0	0	0	0	0	0	0	0	0	0	0	0	0	0	0	0	0	0	0	0	0	0	0	0	0	0	0	0	0	0	0	0	0	0	0	0	0	0	0	0	0	0	0	0	0	0	0	0	0	0	0	0	0	0	-1	-1	-1	-1	-1	-1	-1	-1	-1	-1	-1	-1	
M00706: Multidrug_resistance,_EfrAB_transporter	0	0	0	0	0	0	0	0	0	0	0	0	0	0	0	0	0	0	0	0	0	0	0	0	0	0	0	0	0	0	0	0	0	0	0	0	0	0	0	0	0	0	0	0	0	0	0	0	0	0	0	0	0	0	0	0	0	0	0	0	0	0	0	0	0	-1	-1	-1	-1	-1	-1	-1	-1	-1	-1	-1	-1	
M00707: Multidrug_resistance,_SmdAB/MdlAB_transporter	0	0	0	0	0	0	0	0	0	0	0	0	0	0	0	0	0	0	0	0	0	0	0	0	0	0	0	0	0	0	0	0	0	0	0	0	0	0	0	0	0	0	0	0	0	0	0	0	0	0	0	0	0	0	0	0	0	0	0	0	0	0	0	0	0	-1	-1	-1	-1	-1	-1	-1	-1	-1	-1	-1	-1	
M00708: Multidrug_resistance,_PatAB_transporter	0	0	0	0	0	0	0	0	0	0	0	0	0	0	0	0	0	0	0	0	0	0	0	0	0	0	0	0	0	0	0	0	0	0	0	0	0	0	0	0	0	0	0	0	0	0	0	0	0	0	0	0	0	0	0	0	0	0	0	0	0	0	0	0	0	-1	-1	-1	-1	-1	-1	-1	-1	-1	-1	-1	-1	
M00709: Macrolide_resistance,_MacAB-TolC_transporter	0.8	0.4	0.2	0.2	0.4	0.4	0.4	0.4	0.4	0.4	0.4	0.2	0.4	0.2	0.4	0.4	0.4	0.4	0.4	0.4	0.4	0.6	0.4	0.4	0.6	0.6	0.6	0.6	0.6	0.4	0.6	0.6	0.4	0.4	0.6	0.6	0.6	0.2	0.6	0.6	0.6	0.4	0.6	0.6	0.6	0.2	0.6	0.6	0.6	0.6	0.6	0.6	0.6	0.6	0.4	0.2	0.4	0.6	0.6	0.4	0.6	0.2	0.2	0.6	0.4	0	0	0	0	0	0	0	0	0	0	0	0	
M00711: Multidrug_resistance,_efflux_pump_MdtIJ	0	0	0	0	0	0	0	0	0	0	0	0	0	0	0	0	0	0	0	0	0	0	0	0	0	0	0	0	0	0	0	0	0	0	0	0	0	0	0	0	0	0	0	0	0	0	0	0	0	0	0	0	0	0	0	0	0	0	0	0	0	0	0	0	0	-1	-1	-1	-1	-1	-1	-1	-1	-1	-1	-1	-1	
M00712: Multidrug_resistance,_efflux_pump_YkkCD	0	0	0	0	0	0	0	0	0	0	0	0	0	0	0	0	0	0	0	0	0	0	0	0	0	0	0	0	0	0	0	0	0	0	0	0	0	0	0	0	0	0	0	0	0	0	0	0	0	0	0	0	0	0	0	0	0	0	0	0	0	0	0	0	0	-1	-1	-1	-1	-1	-1	-1	-1	-1	-1	-1	-1	
M00713: Fluoroquinolone_resistance,_efflux_pump_LfrA	0	0	0	0	0.5	0	0.5	0.5	0	0.5	0	0	0.5	0.5	0	0	0.5	0.5	0.5	0	0.5	0.5	0.5	0	0.5	0	0	0	0	0	0	0	0	0	0	0	0	0.5	0	0	0	0	0	0	0	0.5	0	0	0	0	0	0	0	0.5	0	0	0	0	0	0	0	0	0	0	0	-1	0	0	-1	-1	-1	-1	-1	-1	-1	-1	-1	
M00714: Multidrug_resistance,_efflux_pump_QacA	0	0	0	0	0.5	0	0.5	0.5	0	0.5	0	0	0.5	0.5	0	0	0.5	0.5	0.5	0	0.5	0.5	0.5	0	0.5	0	0	0	0	0	0	0	0	0	0	0	0	0.5	0	0	0	0	0	0	0	0.5	0	0	0	0	0	0	0	0.5	0	0	0	0	0	0	0	0	0	0	0	-1	0	0	-1	-1	-1	-1	-1	-1	-1	-1	-1	
M00715: Lincosamide_resistance,_efflux_pump_LmrB	0	0	0	0	0.5	0	0.5	0.5	0	0.5	0	0.5	0.5	0.5	0	0	0	0	0	0	0	0	0	0	0	0	0	0	0	0	0	0	0	0	0	0	0	0	0	0	0	0	0	0	0	0	0	0	0	0	0	0	0	0	0	0	0	0	0	0	0	0	0	0	0	-1	0	-1	-1	-1	-1	-1	-1	-1	-1	-1	-1	
M00716: ArlS-ArlR_(virulence_regulation)_two-component_regulatory_system	0	0	0	0	0	0	0	0	0	0	0	0	0	0	0	0	0	0	0	0	0	0	0	0	0	0	0	0	0	0	0	0	0	0	0	0	0	0	0	0	0	0	0	0	0	0	0	0	0	0	0	0	0	0	0	0	0	0	0	0	0	0	0	0	0	-1	-1	-1	-1	-1	-1	-1	-1	-1	-1	-1	-1	
M00717: Multidrug_resistance,_efflux_pump_NorA	0	0	0	0	0	0	0	0	0	0	0	0	0	0	0	0	0	0	0	0	0	0	0	0	0	0	0	0	0	0	0	0	0	0	0	0	0	0	0	0	0	0	0	0	0	0	0	0	0	0	0	0	0	0	0	0	0	0	0	0	0	0	0	0	0	-1	-1	-1	-1	-1	-1	-1	-1	-1	-1	-1	-1	
M00718: Multidrug_resistance,_efflux_pump_MexAB-OprM	0.75	0.75	0.75	0.75	0.75	0.75	0.75	0.75	0.75	0.75	0.75	0.75	0.75	0.75	0.75	0.75	0.75	0.75	0.75	0.75	0.75	0.75	0.75	0.75	0.75	0.75	0.75	0.75	0.75	0.75	0.75	0.75	0.75	0.75	0.75	0.75	0.75	0.5	0.75	0.75	0.75	0.75	0.75	0.75	0.75	0.75	0.75	0.75	0.75	0.75	0.75	0.75	0.75	0.75	0.75	0.75	0.75	0.75	0.75	0.75	0.75	0.75	0.75	0.75	0.75	0	0	0	0	0	0	0	0	0	0	0	0	
M00720: Multidrug_resistance,_efflux_pump_VexEF-TolC	0.333333	0.333333	0.333333	0.333333	0.333333	0.333333	0.333333	0.333333	0.333333	0.333333	0.333333	0.333333	0.333333	0	0.333333	0.333333	0.333333	0.333333	0.333333	0.333333	0.333333	0.333333	0.333333	0.333333	0.333333	0.333333	0.333333	0.333333	0.333333	0.333333	0.333333	1	1	1	1	0.333333	0.333333	0.333333	0.333333	0.333333	0.333333	0.333333	0.333333	0.333333	0.333333	0	0.333333	0	0.333333	0.333333	0.333333	0	0.333333	0.333333	0.333333	0.333333	0.333333	0.333333	0	0	0.333333	0.333333	0.333333	0.333333	0	0	0	0	0	1	0	0	0	0	0	-1	0	varied
M00721: Cationic_antimicrobial_peptide_(CAMP)_resistance,_arnBCADTEF_operon	0	0.076923	0	0	0	0	0	0	0	0.076923	0	0	0	0	0	0.076923	0	0	0	0	0	0	0	0.076923	0	0	0	0	0	0	0	0	0	0	0	0	0	0	0	0	0	0	0	0	0	0	0	0	0	0.076923	0	0	0	0	0	0	0	0	0.076923	0	0	0	0	0	0	-1	-1	-1	-1	-1	-1	-1	-1	-1	-1	-1	-1	
M00722: Cationic_antimicrobial_peptide_(CAMP)_resistance,_phosphoethanolamine_transferase_PmrC	0.333333	0.333333	0.333333	0.333333	0	0.333333	0.333333	0	0	0	0	0	0	0	0	0	0	0	0	0	0	0.333333	0	0	0.333333	0	0	0	0.333333	0	0	0.333333	0	0	0.333333	0	0	0	0	0	0	0	0	0	0.333333	0	0	0.333333	0.333333	0.333333	0.333333	0.333333	0.333333	0.333333	0.333333	0.333333	0.333333	0.333333	0.333333	0.333333	0.333333	0.333333	0.333333	0.333333	0.333333	0	-1	-1	-1	-1	-1	-1	-1	0	0	0	0	
M00723: Cationic_antimicrobial_peptide_(CAMP)_resistance,_phosphoethanolamine_transferase_EptB	0.25	0.25	0	0	0	0	0	0	0	0	0	0	0	0	0	0.25	0	0	0	0	0	0	0	0	0	0	0	0	0	0	0	0	0	0	0	0	0	0	0	0	0	0	0	0	0	0	0	0	0	0	0	0	0	0	0	0	0	0	0	0	0	0	0	0	0	0	-1	-1	-1	-1	-1	-1	-1	-1	-1	-1	-1	
M00724: Cationic_antimicrobial_peptide_(CAMP)_resistance,_palmitoyl_transferase_PagP	0.333333	0.333333	0	0	0	0	0	0	0	0	0	0	0	0	0	0.333333	0	0	0	0	0	0	0	0	0	0	0	0	0	0	0	0	0	0	0	0	0	0	0	0	0	0	0	0	0	0	0	0	0	0	0	0	0	0	0	0	0	0	0	0	0	0	0	0	0	0	-1	-1	-1	-1	-1	-1	-1	-1	-1	-1	-1	
M00725: Cationic_antimicrobial_peptide_(CAMP)_resistance,_dltABCD_operon	0	0	0	0	0	0	0	0	0	0	0	0	0	0	0	0	0	0	0	0	0	0	0	0.5	0	0	0	0	0	0	0	0	0	0	0	0	0	0	0	0	0	0	0	0	0	0	0	0	0	0	0	0	0	0	0	0	0	0	0	0	0	0	0	0	0	-1	-1	-1	-1	-1	-1	-1	-1	-1	-1	-1	-1	
M00726: Cationic_antimicrobial_peptide_(CAMP)_resistance,_lysyl-phosphatidylglycerol_(L-PG)_synthase_MprF	0.333333	0	0	0	0	0	0	0	0	0	0	0	0	0	0	0	0.333333	0	0	0	0	0	0	0	0	0	0	0	0	0	0	0	0	0	0	0.333333	0.333333	0	0.333333	0.333333	0.333333	0.333333	0.333333	0.333333	0.333333	0	0.333333	0	0	0.333333	0	0	0	0	0	0	0	0	0	0	0	0	0	0	0	-1	-1	-1	-1	-1	0	0	0	-1	-1	-1	-1	
M00727: Cationic_antimicrobial_peptide_(CAMP)_resistance,_N-acetylmuramoyl-L-alanine_amidase_AmiA_and_AmiC	1	1	0.333333	0.333333	0.333333	0.333333	0.333333	0.333333	1	0.333333	0.333333	0.333333	0.333333	0.333333	0.666667	0.666667	0.333333	0.666667	0.666667	1	1	0.666667	0.666667	1	0.666667	0.333333	0.333333	0.333333	0.333333	0.333333	0.333333	0.333333	0.333333	0.333333	0.333333	0.333333	0.333333	0.333333	0.333333	0.333333	0.333333	0.666667	0.333333	0.333333	0.333333	0.333333	0.333333	0.333333	0.333333	0.333333	0.333333	0.333333	0.333333	0.333333	0.333333	0.333333	0.333333	0.333333	0.333333	0.333333	0.333333	0.333333	0.333333	0.333333	0.333333	0	0	0	0	0	0	0	0	0	0	0	0	
M00728: Cationic_antimicrobial_peptide_(CAMP)_resistance,_envelope_protein_folding_and_degrading__factors_DegP_and_DsbA	1	1	0.5	0.5	0.25	0.5	0.25	0.25	0.75	0.25	0.25	0.25	0.25	0.25	0.5	0.5	0.25	0.5	0.5	0.75	0.75	0.75	0.5	0.75	0.75	0.25	0.5	0.5	0.5	0.5	0.5	0.5	0.5	0.25	0.5	0.25	0.25	0.25	0.25	0.25	0.25	0.5	0.25	0.25	0.5	0.25	0.25	0.5	0.5	0.5	0.5	0.5	0.5	0.5	0.5	0.5	0.25	0.5	0.5	0.25	0.5	0.5	0.5	0.5	0.5	0	0	0	0	0	0	0	0	0	0	0	0	
M00729: Fluoroquinolone_resistance,_gyrase-protecting_protein_Qnr	0.666667	0.666667	0.666667	0.666667	0.666667	0.666667	0.666667	0.666667	0.666667	0.666667	0.666667	0.666667	0.666667	0.666667	0.666667	0.666667	0.666667	0.666667	0.666667	0.666667	0.666667	0.666667	0.666667	0.666667	0.666667	0.666667	0.666667	0.666667	0.666667	0.666667	0.666667	0.666667	0.666667	0.666667	0.666667	0.666667	0.666667	0.666667	0.666667	0.666667	0.666667	0.666667	0.666667	0.666667	0.666667	0.666667	0.666667	0.666667	0.666667	0.666667	0.666667	0.666667	0.666667	0.666667	0.666667	0.666667	0.666667	0.666667	0.666667	0.666667	0.666667	0.666667	0.666667	0.666667	0.666667	0	0	0	0	0	0	0	0	0	0	0	0	
M00731: Bacitracin_transport_system	0	0	0	0	0	0	0	0	0	0	0	0	0	0	0	0	0	0	0	0	0	0	0	0	0	0	0	0	0	0	0	0	0	0	0	0	0	0	0	0	0	0	0	0	0	0	0	0	0	0	0	0	0	0	0	0	0	0	0	0	0	0	0	0	0	-1	-1	-1	-1	-1	-1	-1	-1	-1	-1	-1	-1	
M00734: BraS-BraR_(bacitracin_transport)_two-component_regulatory_system	0	0	0	0	0	0	0	0	0	0	0	0	0	0	0	0	0	0	0	0	0	0	0	0	0	0	0	0	0	0	0	0	0	0	0	0	0	0	0	0	0	0	0	0	0	0	0	0	0	0	0	0	0	0	0	0	0	0	0	0	0	0	0	0	0	-1	-1	-1	-1	-1	-1	-1	-1	-1	-1	-1	-1	
M00736: Nocardicin_A_biosynthesis,_L-pHPG_+_arginine_+_serine_=>_nocardicin_A	0	0	0	0	0	0	0	0	0	0	0	0	0	0	0	0	0.166667	0.166667	0.166667	0	0	0.166667	0.166667	0	0.166667	0	0	0	0	0	0	0	0	0	0	0	0	0.166667	0	0	0	0	0	0	0	0	0	0	0	0.166667	0	0	0	0.166667	0	0	0	0.166667	0	0	0.166667	0	0	0	0	-1	-1	0	-1	-1	-1	-1	-1	-1	-1	-1	-1	
M00737: Bacitracin_resistance,_VraDE_transporter	0	0	0	0	0	0	0	0	0	0	0	0	0	0	0	0	0	0	0	0	0	0	0	0	0	0	0	0	0	0	0	0	0	0	0	0	0	0	0	0	0	0	0	0	0	0	0	0	0	0	0	0	0	0	0	0	0	0	0	0	0	0	0	0	0	-1	-1	-1	-1	-1	-1	-1	-1	-1	-1	-1	-1	
M00738: Bacitracin_resistance,_BceAB_transporter	0	0	0	0	0	0	0	0	0	0	0	0	0	0	0	0	0	0	0	0	0	0	0	0	0	0	0	0	0	0	0	0	0	0	0	0	0	0	0	0	0	0	0	0	0	0	0	0	0	0	0	0	0	0	0	0	0	0	0	0	0	0	0	0	0	-1	-1	-1	-1	-1	-1	-1	-1	-1	-1	-1	-1	
M00739: Cationic_peptide_transport_system	0	0	0	0	0	0	0	0	0	0	0	0	0	0	0	0	0	0	0	0	0	0	0	0	0	0	0	0	0	0	0	0	0	0	0	0	0	0	0	0	0	0	0	0	0	0	0	0	0	0	0	0	0	0	0	0	0	0	0	0	0	0	0	0	0	-1	-1	-1	-1	-1	-1	-1	-1	-1	-1	-1	-1	
M00740: Methylaspartate_cycle	0.416667	0.416667	0.416667	0.416667	0.333333	0.416667	0.333333	0.333333	0.416667	0.333333	0.416667	0.416667	0.333333	0.333333	0.416667	0.416667	0.5	0.416667	0.416667	0.416667	0.416667	0.416667	0.416667	0.416667	0.416667	0.416667	0.416667	0.416667	0.416667	0.333333	0.333333	0.333333	0.333333	0.416667	0.333333	0.416667	0.333333	0.416667	0.416667	0.333333	0.416667	0.416667	0.333333	0.416667	0.333333	0.416667	0.333333	0.416667	0.416667	0.416667	0.333333	0.333333	0.416667	0.416667	0.416667	0.416667	0.333333	0.416667	0.416667	0.416667	0.416667	0.416667	0.416667	0.416667	0.416667	0	0	0	0	0	0	0	0	0	0	0	0	
M00741: Propanoyl-CoA_metabolism,_propanoyl-CoA_=>_succinyl-CoA	0.384615	0.461538	0.384615	0.461538	0.461538	0.615385	0.384615	0.461538	0.461538	0.538462	0.538462	0.461538	0.384615	0.384615	0.461538	0.307692	0.538462	0.538462	0.615385	0.538462	0.538462	0.461538	0.538462	0.538462	0.461538	0.461538	0.384615	0.461538	0.461538	0.307692	0.384615	0.307692	0.153846	0.307692	0.307692	0.384615	0.307692	0.615385	0.307692	0.307692	0.307692	0.538462	0.307692	0.461538	0.307692	0.538462	0.307692	0.230769	0.230769	0.307692	0.230769	0.230769	0.307692	0.384615	0.230769	0.230769	0.384615	0.461538	0.384615	0.461538	0.230769	0.230769	0.230769	0.384615	0.384615	0	0	0	0	0	0	0	0	0	0	0	0	
M00742: Aminoglycoside_resistance,_protease_FtsH	1	1	1	1	0.833333	1	0.833333	0.833333	0.5	0.5	0.833333	0.666667	0.5	0.5	0.833333	0.833333	0.666667	0.5	0.5	0.5	0.5	0.833333	0.5	0.5	0.833333	0.666667	1	0.833333	1	1	1	1	1	0.833333	1	0.833333	0.833333	0.166667	0.833333	0.833333	0.833333	0.5	0.833333	0.833333	1	0.166667	0.833333	1	1	1	0.833333	1	1	1	1	1	0.666667	1	1	0.833333	1	1	1	1	0.833333	1	0	0	0	1	0	0	0	1	1	1	1	
M00743: Aminoglycoside_resistance,_protease_HtpX	1	1	1	1	1	1	1	1	0.333333	0.333333	1	0.666667	0.333333	0.333333	1	1	0.666667	0.333333	0.333333	0.333333	0.333333	1	0.333333	0.333333	1	0.666667	1	0.666667	1	1	1	1	1	1	1	1	1	0.333333	1	1	1	1	1	1	1	0.333333	1	1	1	1	1	1	1	1	1	1	0.666667	1	1	1	1	1	1	1	1	1	1	0	0	1	1	1	1	1	1	1	1	
M00744: Cationic_antimicrobial_peptide_(CAMP)_resistance,_protease_PgtE	0.333333	0.333333	0	0	0	0	0	0	0	0	0	0	0	0	0	0.333333	0	0	0	0	0	0	0	0	0	0	0	0	0	0	0	0	0	0	0	0	0	0	0	0	0	0	0	0	0	0	0	0	0	0	0	0	0	0	0	0	0	0	0	0	0	0	0	0	0	0	-1	-1	-1	-1	-1	-1	-1	-1	-1	-1	-1	
M00745: Imipenem_resistance,_repression_of_porin_OprD	0.333333	0.333333	0.333333	0.333333	0	0.166667	0.166667	0	0.166667	0	0	0.333333	0	0	0.166667	0.333333	0.166667	0.333333	0.166667	0.333333	0.333333	0.333333	0.333333	0.333333	0.333333	0.166667	0.166667	0.166667	0.333333	0.333333	0.333333	0.333333	0.333333	0.333333	0.333333	0.333333	0	0.166667	0.166667	0	0.333333	0.166667	0.166667	0.333333	0.333333	0.166667	0.333333	0.333333	0.333333	0.333333	0.333333	0.333333	0.333333	0.333333	0.333333	0.333333	0.333333	0.333333	0.333333	0.333333	0.333333	0.333333	0.333333	0.333333	0.333333	0	-1	0	0	0	0	0	0	0	0	0	0	
M00746: Multidrug_resistance,_repression_of_porin_OmpF	0	0	0	0	0	0	0	0	0	0	0	0	0	0	0	0	0	0	0	0	0	0	0	0	0	0	0	0	0	0	0	0	0	0	0	0	0	0	0	0	0	0	0	0	0	0	0	0	0	0	0	0	0	0	0	0	0	0	0	0	0	0	0	0	0	-1	-1	-1	-1	-1	-1	-1	-1	-1	-1	-1	-1	
M00747: Bacitracin_transport_system	0	0	0	0	0	0	0	0	0	0	0	0	0	0	0	0	0	0	0	0	0	0	0	0	0	0	0	0	0	0	0	0	0	0	0	0	0	0	0	0	0	0	0	0	0	0	0	0	0	0	0	0	0	0	0	0	0	0	0	0	0	0	0	0	0	-1	-1	-1	-1	-1	-1	-1	-1	-1	-1	-1	-1	
M00754: Nisin_resistance,_phage_shock_protein_homolog_LiaH	0	0	0	0	0	0	0	0	0	0	0	0	0	0	0	0	0.25	0	0	0	0	0	0	0	0	0	0	0	0	0	0	0	0	0	0	0	0	0	0	0	0	0	0	0	0	0	0	0	0	0.25	0	0	0	0	0	0	0	0	0.25	0	0	0	0	0	0	-1	-1	-1	-1	-1	-1	-1	-1	-1	-1	-1	-1	
M00760: Erythromycin_resistance,_macrolide_2'-phosphotransferase_I_MphA	0	0	0	0	0	0	0	0	0	0	0	0	0	0	0	0	0	0	0	0	0	0	0	0	0	0	0	0	0	0	0	0	0	0	0	0	0	0	0	0	0	0	0	0	0	0	0	0	0	0	0	0	0	0	0	0	0	0	0	0	0	0	0	0	0	-1	-1	-1	-1	-1	-1	-1	-1	-1	-1	-1	-1	
M00761: Undecaprenylphosphate_alpha-L-Ara4N_biosynthesis,_UDP-GlcA_=>_undecaprenyl_phosphate_alpha-L-Ara4N	0	0	0	0	0	0	0	0	0	0	0	0	0	0	0	0	0	0	0	0	0	0	0	0	0	0	0	0	0	0	0	0	0	0	0	0	0	0	0	0	0	0	0	0	0	0	0	0	0	0	0	0	0	0	0	0	0	0	0	0	0	0	0	0	0	-1	-1	-1	-1	-1	-1	-1	-1	-1	-1	-1	-1	
M00762: Copper-processing_system	0	0	1	1	0	0	0	0	0	0	0	0	0	0	0	0	0.5	0.5	0	0.5	0.5	0.5	0.5	0.5	0.5	1	1	1	0	0	0	0	0	0	0	0.5	0	0	0	0	0	0	0	0	0	0	0	0.5	0.5	0.5	0.5	0.5	0.5	0.5	0.5	1	0	0	0.5	1	1	1	1	1	0	0	-1	0	1	-1	-1	-1	-1	0	0	1	1	varied
M00763: Ornithine_biosynthesis,_mediated_by_LysW,_glutamate_=>_ornithine	0.166667	0.166667	0	0	0	0	0	0	0	0	0	0	0	0	0	0	0.833333	0.833333	0.833333	0.833333	0.333333	0.333333	0.333333	0.166667	0.333333	0	0	0	0	0	0	0	0	0	0	0	0	0	0	0	0	0.833333	0	0	0	0.833333	0	0	0	0	0	0	0	0	0	0	0	0	0	0.166667	0	0	0	0	0	-1	-1	0	-1	-1	-1	-1	-1	-1	-1	-1	-1	
M00764: PTS_system,_fructoselysine_and_glucoselysine-specific_II_component	0	0	0	0	0	0	0	0	0	0	0	0	0	0	0	0	0	0	0	0	0	0	0	0	0	0	0	0	0	0	0	0	0	0	0	0	0	0	0	0	0	0	0	0	0	0	0	0	0	0	0	0	0	0	0	0	0	0	0	0	0	0	0	0	0	-1	-1	-1	-1	-1	-1	-1	-1	-1	-1	-1	-1	
M00766:Streptomycin_resistance,_deactivating_enzyme_StrAB	0	0	0	0	0	0	0	0	0	0	0	0	0	0	0	0	0	0	0.5	0	0	0	0	0	0	0	0	0	0	0	0	0	0	0	0	0	0	0.5	0	0	0	0	0	0	0	0.5	0	0	0	0	0	0	0	0	0	0	0	0	0	0	0	0	0	0	0	-1	-1	-1	-1	-1	-1	-1	-1	-1	-1	-1	-1	
M00767:Multidrug_resistance,_efflux_pump_OqxAB	0	0	0	0	0	0	0	0	0	0	0	0	0	0	0	0	0	0	0	0.25	0.25	0	0	0.25	0	0	0	0	0	0	0	0	0	0	0	0	0	0	0	0	0	0	0	0	0	0	0	0	0	0	0	0	0	0	0	0	0	0	0	0	0	0	0	0	0	-1	-1	-1	-1	-1	-1	-1	-1	-1	-1	-1	-1	
M00768: Multidrug_resistance,_efflux_pump_GesABC	0.25	0.25	0.25	0.25	0.25	0.25	0.25	0.25	0.25	0.25	0.25	0.25	0.25	0.25	0.25	0.25	0.25	0.25	0.25	0.25	0.25	0.25	0.25	0.25	0.25	0.25	0.25	0.25	0.25	0	0.25	0.25	0	0	0	0.25	0.25	0	0	0.25	0.25	0	0	0.25	0.25	0.25	0	0.25	0.25	0.25	0.25	0.25	0.25	0.25	0.25	1	0.25	0.25	0.25	0.25	0.25	1	1	1	0.25	0	0	0	0	-1	0	0	0	0	0	0	1	varied
M00769: Multidrug_resistance,_efflux_pump_MexPQ-OpmE	0	0	0	0	0	0	0	0	0	0	0	0	0	0	0	0	0	0	0	0	0	0	0	0	0	0	0	0	0	0	0	0	0	0	0	0	0	0	0	0	0	0	0	0	0	0	0	0	0	0	0	0	0	0	0	0.4	0	0	0	0	0.4	0.4	0.4	0.4	0	-1	-1	-1	-1	-1	-1	-1	-1	-1	-1	-1	0	
M00770: PfeS-PfeR_(enterobactin-dependent_iron_acquisition)_two-component_regulatory_system	0	0	0	0	0	0	0	0	0	0	0	0	0	0	0	0	0	0	0	0	0	0	0	0	0	0	0	0	0	0	0	0	0	0	0	0	0	0	0	0	0	0	0	0	0	0	0	0	0	0	0	0	0	0	0	0	0	0	0	0	0	0	0	0	0	-1	-1	-1	-1	-1	-1	-1	-1	-1	-1	-1	-1	
M00771: FitF-FitH_(insecticidal_toxin_regulation)_two-component_regulatory_system_	0.5	0	0	0	0	0	0	0	0	0	0	0	0	0	0	0	0	0	0	0	0	0	0	0	0	0	0	0	0	0	0	0	0	0	0	0	0	0	0	0	0	0	0	0	0	0	0	0	0	0	0	0	0	0	0	0	0	0	0	0	0	0	0	0	0	-1	-1	-1	-1	-1	-1	-1	-1	-1	-1	-1	-1	
M00772: HupT-HupR_(hydrogenase_synthesis_regulation)_two-component_regulatory_system_	0	0	0	0	0	0	0	0	0	0	0	0	0	0	0	0	0	0	0	0	0	0	0	0	0	0	0	0	0	0	0	0	0	0	0	0	0	0	0	0	0	0	0	0	0	0	0	0	0	0	0	0	0	0	0	0	0	0	0	0	0	0	0	0	0	-1	-1	-1	-1	-1	-1	-1	-1	-1	-1	-1	-1	
M00773: Tylosin_biosynthesis,_methylmalonyl-CoA_+_malonyl-CoA_=>_tylactone_=>_tylosin	0	0	0	0	0	0	0	0	0	0	0	0	0	0	0	0	0	0	0	0	0	0	0	0	0	0	0	0	0	0	0	0	0	0	0	0	0	0	0	0	0	0	0	0	0	0	0	0	0	0	0	0	0	0	0	0	0	0	0	0	0	0	0	0	0	-1	-1	-1	-1	-1	-1	-1	-1	-1	-1	-1	-1	
M00774: Erythromycin_biosynthesis,_propanoyl-CoA_+_methylmalonyl-CoA_=>_deoxyerythronolide_B_=>_erythromycin_A/B	0	0	0	0	0	0	0	0	0	0	0	0	0	0	0	0	0	0	0	0	0	0	0	0	0	0	0	0	0	0	0	0	0	0	0	0	0	0	0	0	0	0	0	0	0	0	0	0	0	0	0	0	0	0	0	0	0	0	0	0	0	0	0	0	0	-1	-1	-1	-1	-1	-1	-1	-1	-1	-1	-1	-1	
M00775: Oleandomycin_biosynthesis,_malonyl-CoA_+_methylmalonyl-CoA_=>_8,8a-deoxyoleandolide_=>_oleandomycin	0	0	0	0	0	0	0	0	0	0	0	0	0	0	0	0	0	0	0	0	0	0	0	0	0	0	0	0	0	0	0	0	0	0	0	0	0	0	0	0	0	0	0	0	0	0	0	0	0	0	0	0	0	0	0	0	0	0	0	0	0	0	0	0	0	-1	-1	-1	-1	-1	-1	-1	-1	-1	-1	-1	-1	
M00776: Pikromycin/methymycin/neomethymycin_biosynthesis,_methylmalonyl-CoA_+_malonyl-CoA_=>_narbonolide/10-deoxymethynolide_=>_pikromycin/methymycin/neomethymycin	0	0	0	0	0	0	0	0	0	0	0	0	0	0	0	0	0	0	0	0	0	0	0	0	0	0	0	0	0	0	0	0	0	0	0	0	0	0	0	0	0	0	0	0	0	0	0	0	0	0	0	0	0	0	0	0	0	0	0	0	0	0	0	0	0	-1	-1	-1	-1	-1	-1	-1	-1	-1	-1	-1	-1	
M00777: Avermectin_biosynthesis,_2-methylbutanoyl-CoA/isobutyryl-CoA_=>_6,8a-Seco-6,8a-deoxy-5-oxoavermectin_1a/1b_aglycone_=>_avermectin_A1a/B1a/A1b/B1b	0	0	0	0	0	0	0	0	0	0	0	0	0	0	0	0	0	0	0	0	0	0	0	0	0	0	0	0	0	0	0	0	0	0	0	0	0	0	0	0	0	0	0	0	0	0	0	0	0	0	0	0	0	0	0	0	0	0	0	0	0	0	0	0	0	-1	-1	-1	-1	-1	-1	-1	-1	-1	-1	-1	-1	
M00778: Type_II_polyketide_backbone_biosynthesis,_acyl-CoA_+_malonyl-CoA_=>_polyketide	0	0	0	0	0	0	0	0	0	0	0	0	0	0	0	0	0	0	0	0	0	0	0	0	0	0	0	0	0	0	0	0	0	0	0	0	0	0	0	0	0	0	0	0	0	0	0	0	0	0	0	0	0	0	0	0	0	0	0	0	0	0	0	0	0	-1	-1	-1	-1	-1	-1	-1	-1	-1	-1	-1	-1	
M00779: Dihydrokalafungin_biosynthesis,_octaketide_=>_dihydrokalafungin	0	0	0	0	0	0	0	0	0	0	0	0	0	0	0	0	0	0	0	0	0	0	0	0	0	0	0	0	0	0	0	0	0	0	0	0	0	0	0	0	0	0	0	0	0	0	0	0	0	0	0	0	0	0	0	0	0	0	0	0	0	0	0	0	0	-1	-1	-1	-1	-1	-1	-1	-1	-1	-1	-1	-1	
M00780: Tetracycline/oxytetracycline_biosynthesis,_pretetramide_=>_tetracycline/oxytetracycline	0	0	0	0	0	0	0	0	0	0	0	0	0	0	0	0	0	0	0	0	0	0	0	0	0	0	0	0	0	0	0	0	0	0	0	0	0	0	0	0	0	0	0	0	0	0	0	0	0	0	0	0	0	0	0	0	0	0	0	0	0	0	0	0	0	-1	-1	-1	-1	-1	-1	-1	-1	-1	-1	-1	-1	
M00781: Nogalavinone/aklavinone_biosynthesis,_deoxynogalonate/deoxyaklanonate_=>_nogalavinone/aklavinone	0	0	0	0	0	0	0	0	0	0	0	0	0	0	0	0	0	0	0	0	0	0	0	0	0	0	0	0	0	0	0	0	0	0	0	0	0	0	0	0	0	0	0	0	0	0	0	0	0	0	0	0	0	0	0	0	0	0	0	0	0	0	0	0	0	-1	-1	-1	-1	-1	-1	-1	-1	-1	-1	-1	-1	
M00782: Mithramycin_biosynthesis,_4-demethylpremithramycinone_=>_mithramycin	0	0	0	0	0	0	0	0	0	0	0	0	0	0	0	0	0	0	0	0	0	0	0	0	0	0	0	0	0	0	0	0	0	0	0	0	0	0	0	0	0	0	0	0	0	0	0	0	0	0	0	0	0	0	0	0	0	0	0	0	0	0	0	0	0	-1	-1	-1	-1	-1	-1	-1	-1	-1	-1	-1	-1	
M00783: Tetracenomycin_C/8-demethyltetracenomycin_C_biosynthesis,_tetracenomycin_F2_=>_tetracenomycin_C/8-demethyltetracenomycin_C	0	0	0	0	0	0	0	0	0	0	0	0	0	0	0	0	0	0	0	0	0	0	0	0	0	0	0	0	0	0	0	0	0	0	0	0	0	0	0	0	0	0	0	0	0	0	0	0	0	0	0	0	0	0	0	0	0	0	0	0	0	0	0	0	0	-1	-1	-1	-1	-1	-1	-1	-1	-1	-1	-1	-1	
M00784: Elloramycin_biosynthesis,_8-demethyltetracenomycin_C_=>_elloramycin_A	0	0	0	0	0	0	0	0	0	0	0	0	0	0	0	0	0	0	0	0	0	0	0	0	0	0	0	0	0	0	0	0	0	0	0	0	0	0	0	0	0	0	0	0	0	0	0	0	0	0	0	0	0	0	0	0	0	0	0	0	0	0	0	0	0	-1	-1	-1	-1	-1	-1	-1	-1	-1	-1	-1	-1	
M00785: Cycloserine_biosynthesis,_arginine/serine_=>_cycloserine	0	0	0	0	0	0	0	0	0	0	0	0	0	0	0	0	0	0	0	0	0	0	0	0	0	0	0	0	0	0	0	0	0	0	0	0	0	0	0	0	0	0	0	0	0	0	0	0	0	0	0	0	0	0	0	0	0	0	0	0	0	0	0	0	0	-1	-1	-1	-1	-1	-1	-1	-1	-1	-1	-1	-1	

Table S4. VI

Supplementary data. Table S5. Summary table showing differentially abundant bacterial clades identified by LEfSe. (Family level biomarkers are labelled in blue, genus level biomarkers in red and species level biomarkers in green; Rows with biomarkers mentioned in the text are filled in blue).

Differentially abundant Taxa	Habitat†	Taxonomy‡
Terriglobus	A	Bacteria|Acidobacteria|Acidobacteriia|Acidobacteriales|Acidobacteriaceae|Terriglobus
Solibacteres	A	Bacteria|Acidobacteria|Solibacteres
Solibacterales	A	Bacteria|Acidobacteria|Solibacteres|Solibacterales
Actinobacteria	A	Bacteria|Actinobacteria
Iamiaceae	A	Bacteria|Actinobacteria|Acidimicrobiia|Acidimicrobiales|Iamiaceae
Iamia	A	Bacteria|Actinobacteria|Acidimicrobiia|Acidimicrobiales|Iamiaceae|Iamia
Actinobacteria	A	Bacteria|Actinobacteria|Actinobacteria
Actinomycetales	A	Bacteria|Actinobacteria|Actinobacteria|Actinomycetales
Microbacteriaceae	A	Bacteria|Actinobacteria|Actinobacteria|Actinomycetales|Microbacteriaceae
Microbacterium	A	Bacteria|Actinobacteria|Actinobacteria|Actinomycetales|Microbacteriaceae|Microbacterium
Salinibacterium	A	Bacteria|Actinobacteria|Actinobacteria|Actinomycetales|Microbacteriaceae|Salinibacterium
amurskyense	A	Bacteria|Actinobacteria|Actinobacteria|Actinomycetales|Microbacteriaceae|Salinibacterium|amurskyense
Sphingobacteriia	A	Bacteria|Bacteroidetes|Sphingobacteriia
Sphingobacteriales	A	Bacteria|Bacteroidetes|Sphingobacteriia|Sphingobacteriales
Gemmatimonadetes	A	Bacteria|Gemmatimonadetes|Gemmatimonadetes
Gemmatimonadales	A	Bacteria|Gemmatimonadetes|Gemmatimonadetes|Gemmatimonadales
Acidobacteria 6	C	Bacteria|Acidobacteria|Acidobacteria 6
iii1 15	C	Bacteria|Acidobacteria|Acidobacteria 6|iii1 15
mb2424	C	Bacteria|Acidobacteria|Acidobacteria 6|iii1 15|mb2424
Chloracidobacteria	C	Bacteria|Acidobacteria|Chloracidobacteria
RB41	C	Bacteria|Acidobacteria|Chloracidobacteria|RB41
Ellin6075	C	Bacteria|Acidobacteria|Chloracidobacteria|RB41|Ellin6075
Dietziaceae	C	Bacteria|Actinobacteria|Actinobacteria|Actinomycetales|Dietziaceae
Dietzia	C	Bacteria|Actinobacteria|Actinobacteria|Actinomycetales|Dietziaceae|Dietzia
Geodermatophilaceae	C	Bacteria|Actinobacteria|Actinobacteria|Actinomycetales|Geodermatophilaceae
Arthrobacter	C	Bacteria|Actinobacteria|Actinobacteria|Actinomycetales|Micrococcaceae|Arthrobacter
psychrolactophilus	C	Bacteria|Actinobacteria|Actinobacteria|Actinomycetales|Micrococcaceae|Arthrobacter|psychrolactophilus

Micromonosporaceae	C	Bacteria|Actinobacteria|Actinobacteria|Actinomycetales|Micromonosporaceae
Mycobacteriaceae	C	Bacteria|Actinobacteria|Actinobacteria|Actinomycetales|Mycobacteriaceae
Mycobacterium	C	Bacteria|Actinobacteria|Actinobacteria|Actinomycetales|Mycobacteriaceae|Mycobacterium
Nocardiaceae	C	Bacteria|Actinobacteria|Actinobacteria|Actinomycetales|Nocardiaceae
Thermoleophilia	C	Bacteria|Actinobacteria|Thermoleophilia
Solirubrobacterales	C	Bacteria|Actinobacteria|Thermoleophilia|Solirubrobacterales
Solirubrobacteraceae	C	Bacteria|Actinobacteria|Thermoleophilia|Solirubrobacterales|Solirubrobacteraceae
Cytophagia	C	Bacteria|Bacteroidetes|Cytophagia
Cytophagales	C	Bacteria|Bacteroidetes|Cytophagia|Cytophagales
Cytophagaceae	C	Bacteria|Bacteroidetes|Cytophagia|Cytophagales|Cytophagaceae
Chloroflexi	C	Bacteria|Chloroflexi
Chloroflexi	C	Bacteria|Chloroflexi|Chloroflexi
Roseiflexales	C	Bacteria|Chloroflexi|Chloroflexi|Roseiflexales
Kouleothrixaceae	C	Bacteria|Chloroflexi|Chloroflexi|Roseiflexales|Kouleothrixaceae
Ellin6529	C	Bacteria|Chloroflexi|Ellin6529
JG30 KF AS9	C	Bacteria|Chloroflexi|Ktedonobacteria|JG30 KF AS9
S085	C	Bacteria|Chloroflexi|S085
Thermomicrobia	C	Bacteria|Chloroflexi|Thermomicrobia
JG30 KF CM45	C	Bacteria|Chloroflexi|Thermomicrobia|JG30 KF CM45
TK10	C	Bacteria|Chloroflexi|TK10
AKYG885	C	Bacteria|Chloroflexi|TK10|AKYG885
Dolo 23	C	Bacteria|Chloroflexi|TK10|AKYG885|Dolo 23
TK17	C	Bacteria|Chloroflexi|TK17
mle1 48	C	Bacteria|Chloroflexi|TK17|mle1 48
Cyanobacteria	C	Bacteria|Cyanobacteria
Oscillatoriophycideae	C	Bacteria|Cyanobacteria|Oscillatoriophycideae
Synechococcophycideae	C	Bacteria|Cyanobacteria|Synechococcophycideae
Bacillales	C	Bacteria|Firmicutes|Bacilli|Bacillales
Gemmatimonadetes	C	Bacteria|Gemmatimonadetes
Gemm 3	C	Bacteria|Gemmatimonadetes|Gemm 3
Gemm 5	C	Bacteria|Gemmatimonadetes|Gemm 5
Planctomycetes	C	Bacteria|Planctomycetes

Phycisphaerae	C	Bacteria|Planctomycetes|Phycisphaerae
Planctomycetia	C	Bacteria|Planctomycetes|Planctomycetia
Gemmatales	C	Bacteria|Planctomycetes|Planctomycetia|Gemmatales
Gemmataceae	C	Bacteria|Planctomycetes|Planctomycetia|Gemmatales|Gemmataceae
Pirellulales	C	Bacteria|Planctomycetes|Planctomycetia|Pirellulales
Pirellulaceae	C	Bacteria|Planctomycetes|Planctomycetia|Pirellulales|Pirellulaceae
Planctomycetales	C	Bacteria|Planctomycetes|Planctomycetia|Planctomycetales
Planctomycetaceae	C	Bacteria|Planctomycetes|Planctomycetia|Planctomycetales|Planctomycetaceae
Planctomyces	C	Bacteria|Planctomycetes|Planctomycetia|Planctomycetales|Planctomycetaceae|Planctomyces
Kaistobacter	C	Bacteria|Proteobacteria|Alphaproteobacteria|Sphingomonadales|Sphingomonadaceae|Kaistobacter
Janthinobacterium	C	Bacteria|Proteobacteria|Betaproteobacteria|Burkholderiales|Oxalobacteraceae|Janthinobacterium
Myxococcales	C	Bacteria|Proteobacteria|Deltaproteobacteria|Myxococcales
Myxococcaceae	C	Bacteria|Proteobacteria|Deltaproteobacteria|Myxococcales|Myxococcaceae
Anaeromyxobacter	C	Bacteria|Proteobacteria|Deltaproteobacteria|Myxococcales|Myxococcaceae|Anaeromyxobacter
Verrucomicrobia	C	Bacteria|Verrucomicrobia
Opitutae	C	Bacteria|Verrucomicrobia|Opitutae
Opitutales	C	Bacteria|Verrucomicrobia|Opitutae|Opitutales
Opitutaceae	C	Bacteria|Verrucomicrobia|Opitutae|Opitutales|Opitutaceae
Opitutus	C	Bacteria|Verrucomicrobia|Opitutae|Opitutales|Opitutaceae|Opitutus
Spartobacteria	C	Bacteria|Verrucomicrobia|Spartobacteria
Chthoniobacterales	C	Bacteria|Verrucomicrobia|Spartobacteria|Chthoniobacterales
Chthoniobacteraceae	C	Bacteria|Verrucomicrobia|Spartobacteria|Chthoniobacterales|Chthoniobacteraceae
Chthoniobacter	C	Bacteria|Verrucomicrobia|Spartobacteria|Chthoniobacterales|Chthoniobacteraceae|Chthoniobacter
DA101	C	Bacteria|Verrucomicrobia|Spartobacteria|Chthoniobacterales|Chthoniobacteraceae|DA101
Luteolibacter	C	Bacteria|Verrucomicrobia|Verrucomicrobiae|Verrucomicrobiales|Verrucomicrobiaceae|Luteolibacter
Bacteroidetes	DWH	Bacteria|Bacteroidetes
Flavobacteriia	DWH	Bacteria|Bacteroidetes|Flavobacteriia
Flavobacteriales	DWH	Bacteria|Bacteroidetes|Flavobacteriia|Flavobacteriales
Flavobacteriaceae	DWH	Bacteria|Bacteroidetes|Flavobacteriia|Flavobacteriales|Flavobacteriaceae
Weeksellaceae	DWH	Bacteria|Bacteroidetes|Flavobacteriia|Flavobacteriales|Weeksellaceae
Chryseobacterium	DWH	Bacteria|Bacteroidetes|Flavobacteriia|Flavobacteriales|Weeksellaceae|Chryseobacterium
BD7 3	DWH	Bacteria|Proteobacteria|Alphaproteobacteria|BD7 3

Rhodobacterales	DWH	Bacteria|Proteobacteria|Alphaproteobacteria|Rhodobacterales
Rhodobacteraceae	DWH	Bacteria|Proteobacteria|Alphaproteobacteria|Rhodobacterales|Rhodobacteraceae
Octadecabacter	DWH	Bacteria|Proteobacteria|Alphaproteobacteria|Rhodobacterales|Rhodobacteraceae|Octadecabacter
Phaeobacter	DWH	Bacteria|Proteobacteria|Alphaproteobacteria|Rhodobacterales|Rhodobacteraceae|Phaeobacter
Epsilonproteobacteria	DWH	Bacteria|Proteobacteria|Epsilonproteobacteria
Campylobacterales	DWH	Bacteria|Proteobacteria|Epsilonproteobacteria|Campylobacterales
Gammaproteobacteria	DWH	Bacteria|Proteobacteria|Gammaproteobacteria
Alteromonadales	DWH	Bacteria|Proteobacteria|Gammaproteobacteria|Alteromonadales
Alteromonadaceae	DWH	Bacteria|Proteobacteria|Gammaproteobacteria|Alteromonadales|Alteromonadaceae
HB2 32 21	DWH	Bacteria|Proteobacteria|Gammaproteobacteria|Alteromonadales|Alteromonadaceae|HB2 32 21
Colwelliaceae	DWH	Bacteria|Proteobacteria|Gammaproteobacteria|Alteromonadales|Colwelliaceae
Marinicellales	DWH	Bacteria|Proteobacteria|Gammaproteobacteria|Marinicellales
Marinicellaceae	DWH	Bacteria|Proteobacteria|Gammaproteobacteria|Marinicellales|Marinicellaceae
Xanthomonadales	DWH	Bacteria|Proteobacteria|Gammaproteobacteria|Xanthomonadales
Xanthomonadaceae	DWH	Bacteria|Proteobacteria|Gammaproteobacteria|Xanthomonadales|Xanthomonadaceae
Verrucomicrobiae	DWH	Bacteria|Verrucomicrobia|Verrucomicrobiae
Verrucomicrobiales	DWH	Bacteria|Verrucomicrobia|Verrucomicrobiae|Verrucomicrobiales
Verrucomicrobiaceae	DWH	Bacteria|Verrucomicrobia|Verrucomicrobiae|Verrucomicrobiales|Verrucomicrobiaceae
Persicirhabdus	DWH	Bacteria|Verrucomicrobia|Verrucomicrobiae|Verrucomicrobiales|Verrucomicrobiaceae|Persicirhabdus
Acidobacteria	I	Bacteria|Acidobacteria
Acidobacteriia	I	Bacteria|Acidobacteria|Acidobacteriia
Acidobacteriales	I	Bacteria|Acidobacteria|Acidobacteriia|Acidobacteriales
Acidobacteriaceae	I	Bacteria|Acidobacteria|Acidobacteriia|Acidobacteriales|Acidobacteriaceae
Edaphobacter	I	Bacteria|Acidobacteria|Acidobacteriia|Acidobacteriales|Acidobacteriaceae|Edaphobacter
modestum	I	Bacteria|Acidobacteria|Acidobacteriia|Acidobacteriales|Acidobacteriaceae|Edaphobacter|modestum
Koribacteraceae	I	Bacteria|Acidobacteria|Acidobacteriia|Acidobacteriales|Koribacteraceae
Candidatus Koribacter	I	Bacteria|Acidobacteria|Acidobacteriia|Acidobacteriales|Koribacteraceae|Candidatus Koribacter
Saprospirae	I	Bacteria|Bacteroidetes|Saprospirae
Saprospirales	I	Bacteria|Bacteroidetes|Saprospirae|Saprospirales
Chitinophagaceae	I	Bacteria|Bacteroidetes|Saprospirae|Saprospirales|Chitinophagaceae
Chlorobi	I	Bacteria|Chlorobi
Ignavibacteria	I	Bacteria|Chlorobi|Ignavibacteria

Ignavibacteriales	I	Bacteria|Chlorobi|Ignavibacteria|Ignavibacteriales
Ignavibacteriaceae	I	Bacteria|Chlorobi|Ignavibacteria|Ignavibacteriales|Ignavibacteriaceae
Rhodospirillales	I	Bacteria|Proteobacteria|Alphaproteobacteria|Rhodospirillales
Acetobacteraceae	I	Bacteria|Proteobacteria|Alphaproteobacteria|Rhodospirillales|Acetobacteraceae
Rhodospirillaceae	I	Bacteria|Proteobacteria|Alphaproteobacteria|Rhodospirillales|Rhodospirillaceae
Methylibium	I	Bacteria|Proteobacteria|Betaproteobacteria|Burkholderiales|Comamonadaceae|Methylibium
petroleiphilum	I	Bacteria|Proteobacteria|Betaproteobacteria|Burkholderiales|Comamonadaceae|Methylibium|petroleiphilum
Hydrogenophilales	I	Bacteria|Proteobacteria|Betaproteobacteria|Hydrogenophilales
Hydrogenophilaceae	I	Bacteria|Proteobacteria|Betaproteobacteria|Hydrogenophilales|Hydrogenophilaceae
Thiobacillus	I	Bacteria|Proteobacteria|Betaproteobacteria|Hydrogenophilales|Hydrogenophilaceae|Thiobacillus
Sinobacteraceae	I	Bacteria|Proteobacteria|Gammaproteobacteria|Xanthomonadales|Sinobacteraceae
Acidimicrobiia	M	Bacteria|Actinobacteria|Acidimicrobiia
Acidimicrobiales	M	Bacteria|Actinobacteria|Acidimicrobiia|Acidimicrobiales
Chloroplast	M	Bacteria|Cyanobacteria|Chloroplast
GN04	M	Bacteria|GN04
GN15	M	Bacteria|GN04|GN15
OP8	M	Bacteria|OP8
Phycisphaerales	M	Bacteria|Planctomycetes|Phycisphaerae|Phycisphaerales
Phycisphaeraceae	M	Bacteria|Planctomycetes|Phycisphaerae|Phycisphaerales|Phycisphaeraceae
Erythrobacteraceae	M	Bacteria|Proteobacteria|Alphaproteobacteria|Sphingomonadales|Erythrobacteraceae
Desulfobacterales	M	Bacteria|Proteobacteria|Deltaproteobacteria|Desulfobacterales
Desulfuromonadaceae	M	Bacteria|Proteobacteria|Deltaproteobacteria|Desulfuromonadales|Desulfuromonadaceae
Oceanospirillales	M	Bacteria|Proteobacteria|Gammaproteobacteria|Oceanospirillales
Spirochaetes	M	Bacteria|Spirochaetes
Spirochaetes	M	Bacteria|Spirochaetes|Spirochaetes
Spirochaetales	M	Bacteria|Spirochaetes|Spirochaetes|Spirochaetales
Spirochaetaceae	M	Bacteria|Spirochaetes|Spirochaetes|Spirochaetales|Spirochaetaceae
Pedosphaerae	M	Bacteria|Verrucomicrobia|Pedosphaerae
Pedosphaerales	M	Bacteria|Verrucomicrobia|Pedosphaerae|Pedosphaerales
Sediment 1	M	Bacteria|WS3|PRR 12|Sediment 1
Propionibacteriaceae	OSC	Bacteria|Actinobacteria|Actinobacteria|Actinomycetales|Propionibacteriaceae
Propionibacterium	OSC	Bacteria|Actinobacteria|Actinobacteria|Actinomycetales|Propionibacteriaceae|Propionibacterium

Sediminibacterium	OSC	Bacteria|Bacteroidetes|Saprospirae|Saprospirales|Chitinophagaceae|Sediminibacterium
Bacilli	OSC	Bacteria|Firmicutes|Bacilli
Proteobacteria	OSC	Bacteria|Proteobacteria
Rhizobiales	OSC	Bacteria|Proteobacteria|Alphaproteobacteria|Rhizobiales
Brucellaceae	OSC	Bacteria|Proteobacteria|Alphaproteobacteria|Rhizobiales|Brucellaceae
Ochrobactrum	OSC	Bacteria|Proteobacteria|Alphaproteobacteria|Rhizobiales|Brucellaceae|Ochrobactrum
intermedium	OSC	Bacteria|Proteobacteria|Alphaproteobacteria|Rhizobiales|Brucellaceae|Ochrobactrum|intermedium
Methylobacteriaceae	OSC	Bacteria|Proteobacteria|Alphaproteobacteria|Rhizobiales|Methylobacteriaceae
Methylobacterium	OSC	Bacteria|Proteobacteria|Alphaproteobacteria|Rhizobiales|Methylobacteriaceae|Methylobacterium
adhaesivum	OSC	Bacteria|Proteobacteria|Alphaproteobacteria|Rhizobiales|Methylobacteriaceae|Methylobacterium|adhaesivum
Delftia	OSC	Bacteria|Proteobacteria|Betaproteobacteria|Burkholderiales|Comamonadaceae|Delftia
Oxalobacteraceae	OSC	Bacteria|Proteobacteria|Betaproteobacteria|Burkholderiales|Oxalobacteraceae
Cupriavidus	OSC	Bacteria|Proteobacteria|Betaproteobacteria|Burkholderiales|Oxalobacteraceae|Cupriavidus
gilardii	OSC	Bacteria|Proteobacteria|Betaproteobacteria|Burkholderiales|Oxalobacteraceae|Cupriavidus|gilardii
Enterobacteriales	OSC	Bacteria|Proteobacteria|Gammaproteobacteria|Enterobacteriales
Enterobacteriaceae	OSC	Bacteria|Proteobacteria|Gammaproteobacteria|Enterobacteriales|Enterobacteriaceae
Pseudomonadales	OSC	Bacteria|Proteobacteria|Gammaproteobacteria|Pseudomonadales
Moraxellaceae	OSC	Bacteria|Proteobacteria|Gammaproteobacteria|Pseudomonadales|Moraxellaceae
Acinetobacter	OSC	Bacteria|Proteobacteria|Gammaproteobacteria|Pseudomonadales|Moraxellaceae|Acinetobacter
Caldiserica	OSTPd	Bacteria|Caldiserica
Caldisericia	OSTPd	Bacteria|Caldiserica|Caldisericia
Caldisericales	OSTPd	Bacteria|Caldiserica|Caldisericia|Caldisericales
Betaproteobacteria	OSTPd	Bacteria|Proteobacteria|Betaproteobacteria
Rhodocyclales	OSTPd	Bacteria|Proteobacteria|Betaproteobacteria|Rhodocyclales
Rhodocyclaceae	OSTPd	Bacteria|Proteobacteria|Betaproteobacteria|Rhodocyclales|Rhodocyclaceae
PYR10d3	OSTPd	Bacteria|Proteobacteria|Gammaproteobacteria|PYR10d3
Bacteroidia	OSTPm	Bacteria|Bacteroidetes|Bacteroidia
Bacteroidales	OSTPm	Bacteria|Bacteroidetes|Bacteroidia|Bacteroidales
Anaerolineae	OSTPm	Bacteria|Chloroflexi|Anaerolineae
Anaerolineales	OSTPm	Bacteria|Chloroflexi|Anaerolineae|Anaerolineales
Anaerolinaceae	OSTPm	Bacteria|Chloroflexi|Anaerolineae|Anaerolineales|Anaerolinaceae
SHD 231	OSTPm	Bacteria|Chloroflexi|Anaerolineae|Anaerolineales|Anaerolinaceae|SHD 231

T78	OSTPm	Bacteria|Chloroflexi|Anaerolineae|Anaerolineales|Anaerolinaceae|T78
Dehalococcoidetes	OSTPm	Bacteria|Chloroflexi|Dehalococcoidetes
Nitrospirae	OSTPm	Bacteria|Nitrospirae
Nitrospira	OSTPm	Bacteria|Nitrospirae|Nitrospira
Nitrospirales	OSTPm	Bacteria|Nitrospirae|Nitrospira|Nitrospirales
Thauera	OSTPm	Bacteria|Proteobacteria|Betaproteobacteria|Rhodocyclales|Rhodocyclaceae|Thauera
Deltaproteobacteria	OSTPm	Bacteria|Proteobacteria|Deltaproteobacteria
Desulfobulbaceae	OSTPm	Bacteria|Proteobacteria|Deltaproteobacteria|Desulfobacterales|Desulfobulbaceae
Desulfuromonadales	OSTPm	Bacteria|Proteobacteria|Deltaproteobacteria|Desulfuromonadales
Syntrophobacterales	OSTPm	Bacteria|Proteobacteria|Deltaproteobacteria|Syntrophobacterales
Syntrophaceae	OSTPm	Bacteria|Proteobacteria|Deltaproteobacteria|Syntrophobacterales|Syntrophaceae
Syntrophus	OSTPm	Bacteria|Proteobacteria|Deltaproteobacteria|Syntrophobacterales|Syntrophaceae|Syntrophus
Pseudomonadaceae	OSTPm	Bacteria|Proteobacteria|Gammaproteobacteria|Pseudomonadales|Pseudomonadaceae
Pseudomonas	OSTPm	Bacteria|Proteobacteria|Gammaproteobacteria|Pseudomonadales|Pseudomonadaceae|Pseudomonas
Treponema	OSTPm	Bacteria|Spirochaetes|Spirochaetes|Spirochaetales|Spirochaetaceae|Treponema
WS3	OSTPm	Bacteria|WS3
PRR 12	OSTPm	Bacteria|WS3|PRR 12
SSS58A	OSTPm	Bacteria|WS3|PRR 12|SSS58A
AC1	OSTPu	Bacteria|AC1
OPB41	OSTPu	Bacteria|Actinobacteria|OPB41
Firmicutes	OSTPu	Bacteria|Firmicutes
Clostridia	OSTPu	Bacteria|Firmicutes|Clostridia
Clostridiales	OSTPu	Bacteria|Firmicutes|Clostridia|Clostridiales
Peptococcaceae	OSTPu	Bacteria|Firmicutes|Clostridia|Clostridiales|Peptococcaceae
Pelotomaculum	OSTPu	Bacteria|Firmicutes|Clostridia|Clostridiales|Peptococcaceae|Pelotomaculum
Burkholderiales	OSTPu	Bacteria|Proteobacteria|Betaproteobacteria|Burkholderiales
Comamonadaceae	OSTPu	Bacteria|Proteobacteria|Betaproteobacteria|Burkholderiales|Comamonadaceae
Polaromonas	OSTPu	Bacteria|Proteobacteria|Betaproteobacteria|Burkholderiales|Comamonadaceae|Polaromonas
Rhodoferax	OSTPu	Bacteria|Proteobacteria|Betaproteobacteria|Burkholderiales|Comamonadaceae|Rhodoferax
Geobacteraceae	OSTPu	Bacteria|Proteobacteria|Deltaproteobacteria|Desulfuromonadales|Geobacteraceae
Geobacter	OSTPu	Bacteria|Proteobacteria|Deltaproteobacteria|Desulfuromonadales|Geobacteraceae|Geobacter
Syntrophorhabdaceae	OSTPu	Bacteria|Proteobacteria|Deltaproteobacteria|Syntrophobacterales|Syntrophorhabdaceae

WS6	OSTPu	Bacteria|WS6
SC72	OSTPu	Bacteria|WS6|SC72
Gaiellales	Tb	Bacteria|Actinobacteria|Thermoleophilia|Gaiellales
Gaiellaceae	Tb	Bacteria|Actinobacteria|Thermoleophilia|Gaiellales|Gaiellaceae
Alphaproteobacteria	Tb	Bacteria|Proteobacteria|Alphaproteobacteria
Caulobacterales	Tb	Bacteria|Proteobacteria|Alphaproteobacteria|Caulobacterales
Caulobacteraceae	Tb	Bacteria|Proteobacteria|Alphaproteobacteria|Caulobacterales|Caulobacteraceae
Arthrospira	Tb	Bacteria|Proteobacteria|Alphaproteobacteria|Caulobacterales|Caulobacteraceae|Arthrospira
fusiformis	Tb	Bacteria|Proteobacteria|Alphaproteobacteria|Caulobacterales|Caulobacteraceae|Arthrospira|fusiformis
Caulobacter	Tb	Bacteria|Proteobacteria|Alphaproteobacteria|Caulobacterales|Caulobacteraceae|Caulobacter
henricii	Tb	Bacteria|Proteobacteria|Alphaproteobacteria|Caulobacterales|Caulobacteraceae|Caulobacter|henricii
Bradyrhizobiaceae	Tb	Bacteria|Proteobacteria|Alphaproteobacteria|Rhizobiales|Bradyrhizobiaceae
Bradyrhizobium	Tb	Bacteria|Proteobacteria|Alphaproteobacteria|Rhizobiales|Bradyrhizobiaceae|Bradyrhizobium
Hyphomicrobiaceae	Tb	Bacteria|Proteobacteria|Alphaproteobacteria|Rhizobiales|Hyphomicrobiaceae
Burkholderia	Tb	Bacteria|Proteobacteria|Betaproteobacteria|Burkholderiales|Burkholderiaceae|Burkholderia
TM7	Tb	Bacteria|TM7
iii1 8	Tp	Bacteria|Acidobacteria|iii1 8
DS 18	Tp	Bacteria|Acidobacteria|iii1 8|DS 18
Sporichthyaceae	Tp	Bacteria|Actinobacteria|Actinobacteria|Actinomycetales|Sporichthyaceae
Ktedonobacteria	Tp	Bacteria|Chloroflexi|Ktedonobacteria
Thermogemmatisporales	Tp	Bacteria|Chloroflexi|Ktedonobacteria|Thermogemmatisporales
Thermogemmatisporaceae	Tp	Bacteria|Chloroflexi|Ktedonobacteria|Thermogemmatisporales|Thermogemmatisporaceae
Phenylobacterium	Tp	Bacteria|Proteobacteria|Alphaproteobacteria|Caulobacterales|Caulobacteraceae|Phenylobacterium
Sphingomonadales	Tp	Bacteria|Proteobacteria|Alphaproteobacteria|Sphingomonadales
Sphingomonadaceae	Tp	Bacteria|Proteobacteria|Alphaproteobacteria|Sphingomonadales|Sphingomonadaceae
Novosphingobium	Tp	Bacteria|Proteobacteria|Alphaproteobacteria|Sphingomonadales|Sphingomonadaceae|Novosphingobium
capsulatum	Tp	Bacteria|Proteobacteria|Alphaproteobacteria|Sphingomonadales|Sphingomonadaceae|Novosphingobium|capsulatum
Alcaligenaceae	Tp	Bacteria|Proteobacteria|Betaproteobacteria|Burkholderiales|Alcaligenaceae
Intrasporangiaceae	Tu	Bacteria|Actinobacteria|Actinobacteria|Actinomycetales|Intrasporangiaceae
Micrococcaceae	Tu	Bacteria|Actinobacteria|Actinobacteria|Actinomycetales|Micrococcaceae
Rhodococcus	Tu	Bacteria|Actinobacteria|Actinobacteria|Actinomycetales|Nocardiaceae|Rhodococcus
Nocardioidaceae	Tu	Bacteria|Actinobacteria|Actinobacteria|Actinomycetales|Nocardioidaceae

<table>
<tr><td>MB A2 108</td><td>Tu</td><td>Bacteria|Actinobacteria|MB A2 108</td></tr>
<tr><td>0319 7L14</td><td>Tu</td><td>Bacteria|Actinobacteria|MB A2 108|0319 7L14</td></tr>
<tr><td>Ellin329</td><td>Tu</td><td>Bacteria|Proteobacteria|Alphaproteobacteria|Ellin329</td></tr>
<tr><td>Burkholderiaceae</td><td>Tu</td><td>Bacteria|Proteobacteria|Betaproteobacteria|Burkholderiales|Burkholderiaceae</td></tr>
</table>

† Column labelled "Habitat" represents the petroleum contaminated environment in which the corresponding taxa (as presented in column labelled "Differentially abundant Taxa"), was found to be significantly differentially abundant by LEfSe using the one class, non-strict test (Please refer to Materials and methods, and Supplementary Table S2 for details). Acronyms represent the following habitats: A: Arctic, C: China oil refineries, I: India oil refineries, M: Mangrove, DWH: Marine sediments, OSC: Oil sands core, OSTPu: Oil sands tailings pond upper, OSTPm: Oil sands tailings pond median, OSTPd: Oil sands tailings pond deep, Tb: Taiga bottom active layer, Tu: Taiga upper active layer, Tp: Taiga permafrost layer.

‡ Taxonomy is described using the following hierarchy: Kingdom|Phylum|Class|Order|Family|Genus|species.

Supplementary data. Table S6. Summary table showing differentially abundant KEGG Modules identified by LEfSe. (Rows with biomarkers mentioned in the text are filled in blue).

Differentially abundant KEGG Modules	Habitat†	KEGG Module Hierarchy‡
M00006: Pentose phosphate pathway, oxidative phase, glucose 6P => ribulose 5P	A	Pathway module|Carbohydrate and lipid metabolism|Central carbohydrate metabolism|Pentose phosphate pathway, oxidative phase, glucose 6P => ribulose 5P M00006
M00050: Guanine ribonucleotide biosynthesis IMP => GDP,GTP	A	Pathway module|Nucleotide and amino acid metabolism|Purine metabolism|Guanine ribonucleotide biosynthesis IMP => GDP,GTP M00050
M00086: beta-Oxidation, acyl-CoA synthesis	A	Pathway module|Carbohydrate and lipid metabolism|Fatty acid metabolism|beta-Oxidation, acyl-CoA synthesis M00086
M00201: alpha-Glucoside transport system	A	Structural complex|Environmental information processing|Saccharide, polyol, and lipid transport system|alpha-Glucoside transport system M00201
M00206: Cellobiose transport system	A	Structural complex|Environmental information processing|Saccharide, polyol, and lipid transport system|Cellobiose transport system M00206
M00207: Putative multiple sugar transport system	A	Structural complex|Environmental information processing|Saccharide, polyol, and lipid transport system|Putative multiple sugar transport system M00207
M00209: Osmoprotectant transport system	A	Structural complex|Environmental information processing|Mineral and organic ion transport system|Osmoprotectant transport system M00209
M00216: Multiple sugar transport system	A	Structural complex|Environmental information processing|Saccharide, polyol, and lipid transport system|Multiple sugar transport system M00216
M00221: Putative simple sugar transport system	A	Structural complex|Environmental information processing|Saccharide, polyol, and lipid transport system|Putative simple sugar transport system M00221
M00227: Glutamine transport system	A	Structural complex|Environmental information processing|Phosphate and amino acid transport system|Glutamine transport system M00227
M00233: Glutamate transport system	A	Structural complex|Environmental information processing|Phosphate and amino acid transport system|Glutamate transport system M00233
M00240: Iron complex transport system	A	Structural complex|Environmental information processing|Metallic cation, iron-siderophore and vitamin B12 transport system|Iron complex transport system M00240
M00244: Putative zinc/manganese transport system	A	Structural complex|Environmental information processing|Metallic cation, iron-siderophore and vitamin B12 transport system|Putative zinc/manganese transport system M00244
M00271: PTS system, beta-glucosides-specific II component	A	Structural complex|Environmental information processing|Phosphotransferase system (PTS)|PTS system, beta-glucosides-specific II component M00271
M00274: PTS system, mannitol-specific II component	A	Structural complex|Environmental information processing|Phosphotransferase system (PTS)|PTS system, mannitol-specific II component M00274
M00290: Holo-TFIIH complex	A	Structural complex|Genetic information processing|Repair system|Holo-TFIIH complex M00290
M00358: Coenzyme M biosynthesis	A	Pathway module|Energy metabolism|Methane metabolism|Coenzyme M biosynthesis M00358
M00361: Nucleotide sugar biosynthesis, eukaryotes	A	Functional set|Metabolism|Nucleotide sugar|Nucleotide sugar biosynthesis, eukaryotes M00361
M00362: Nucleotide sugar biosynthesis, prokaryotes	A	Functional set|Metabolism|Nucleotide sugar|Nucleotide sugar biosynthesis, prokaryotes M00362
M00443: SenX3-RegX3 (phosphate starvation response) two-component regulatory system	A	Functional set|Environmental information processing|Two-component regulatory system|SenX3-RegX3 (phosphate starvation response) two-component regulatory system M00443
M00479: DesK-DesR (membrane lipid fluidity regulation) two-component regulatory system	A	Functional set|Environmental information processing|Two-component regulatory system|DesK-DesR (membrane lipid fluidity regulation) two-component regulatory system M00479
M00581: Biotin transport system	A	Structural complex|Environmental information processing|Metallic cation, iron-siderophore and vitamin B12 transport system|Biotin transport system M00581
M00582: Energy-coupling factor transport system	A	Structural complex|Environmental information processing|Metallic cation, iron-siderophore and vitamin B12 transport system|Energy-coupling factor transport system M00582

M00609: Cysteine biosynthesis, methionine => cysteine	A	Pathway module|Nucleotide and amino acid metabolism|Cysteine and methionine metabolism|Cysteine biosynthesis, methionine => cysteine M00609
M00617: Methanogen	A	Signature module|Gene set|Metabolic capacity|Methanogen M00617
M00632: Galactose degradation, Leloir pathway, galactose => alpha-D-glucose-1P	A	Pathway module|Carbohydrate and lipid metabolism|Other carbohydrate metabolism|Galactose degradation, Leloir pathway, galactose => alpha-D-glucose-1P M00632
M00651: Vancomycin resistance, D-Ala-D-Lac type	A	Signature module|Gene set|Drug resistance|Vancomycin resistance, D-Ala-D-Lac type M00651
M00658: VanS-VanR (actinomycete type vancomycin resistance) two-component regulatory system	A	Functional set|Environmental information processing|Two-component regulatory system|VanS-VanR (actinomycete type vancomycin resistance) two-component regulatory system M00658
M00713: Fluoroquinolone resistance, efflux pump LfrA	A	Functional set|Environmental information processing|Drug efflux transporter/pump|Fluoroquinolone resistance, efflux pump LfrA M00713
M00714: Multidrug resistance, efflux pump QacA	A	Signature module|Gene set|Drug resistance|Multidrug resistance, efflux pump QacA M00714
M00715: Lincosamide resistance, efflux pump LmrB	A	Functional set|Environmental information processing|Drug efflux transporter/pump|Lincosamide resistance, efflux pump LmrB M00715
M00005: PRPP biosynthesis, ribose 5P => PRPP	C	Pathway module|Carbohydrate and lipid metabolism|Central carbohydrate metabolism|PRPP biosynthesis, ribose 5P => PRPP M00005
M00014: Glucuronate pathway (uronate pathway)	C	Pathway module|Carbohydrate and lipid metabolism|Other carbohydrate metabolism|Glucuronate pathway (uronate pathway) M00014
M00031: Lysine biosynthesis, mediated by LysW, 2-aminoadipate => lysine	C	Pathway module|Nucleotide and amino acid metabolism|Lysine metabolism|Lysine biosynthesis, mediated by LysW, 2-aminoadipate => lysine M00031
M00076: Dermatan sulfate degradation	C	Pathway module|Carbohydrate and lipid metabolism|Glycosaminoglycan metabolism|Dermatan sulfate degradation M00076
M00089: Triacylglycerol biosynthesis	C	Pathway module|Carbohydrate and lipid metabolism|Lipid metabolism|Triacylglycerol biosynthesis M00089
M00112: Tocopherol biosynthesis	C	Pathway module|Nucleotide and amino acid metabolism|Cofactor and vitamin biosynthesis|Tocopherol biosynthesis M00112
M00116: Menaquinone biosynthesis, chorismate => menaquinone	C	Pathway module|Nucleotide and amino acid metabolism|Cofactor and vitamin biosynthesis|Menaquinone biosynthesis, chorismate => menaquinone M00116
M00145: NAD(P)H:quinone oxidoreductase, chloroplasts and cyanobacteria	C	Structural complex|Energy metabolism|ATP synthesis|NAD(P)H:quinone oxidoreductase, chloroplasts and cyanobacteria M00145
M00161: Photosystem II	C	Structural complex|Energy metabolism|Photosynthesis|Photosystem II M00161
M00162: Cytochrome b6f complex	C	Structural complex|Energy metabolism|ATP synthesis|Cytochrome b6f complex M00162
M00163: Photosystem I	C	Structural complex|Energy metabolism|Photosynthesis|Photosystem I M00163
M00196: Multiple sugar transport system	C	Structural complex|Environmental information processing|Saccharide, polyol, and lipid transport system|Multiple sugar transport system M00196
M00197: Putative fructooligosaccharide transport system	C	Structural complex|Environmental information processing|Saccharide, polyol, and lipid transport system|Putative fructooligosaccharide transport system M00197
M00212: Ribose transport system	C	Structural complex|Environmental information processing|Saccharide, polyol, and lipid transport system|Ribose transport system M00212
M00220: Rhamnose transport system	C	Structural complex|Environmental information processing|Saccharide, polyol, and lipid transport system|Rhamnose transport system M00220
M00239: Peptides/nickel transport system	C	Structural complex|Environmental information processing|Peptide and nickel transport system|Peptides/nickel transport system M00239
M00243: Manganese/iron transport system	C	Structural complex|Environmental information processing|Metallic cation, iron-siderophore and vitamin B12 transport system|Manganese/iron transport system M00243
M00253: Sodium transport system	C	Structural complex|Environmental information processing|ABC-2 type and other transport systems|Sodium transport system M00253
M00254: ABC-2 type transport system	C	Structural complex|Environmental information processing|ABC-2 type and other transport systems|ABC-2 type transport system M00254
M00319: Manganese/zinc/iron transport system	C	Structural complex|Environmental information processing|Metallic cation, iron-siderophore and vitamin B12 transport system|Manganese/zinc/iron transport system M00319
M00322: Neutral amino acid transport system	C	Structural complex|Environmental information processing|Phosphate and amino acid transport system|Neutral amino acid transport system M00322
M00336: Twin-arginine translocation (Tat) system	C	Structural complex|Environmental information processing|Bacterial secretion system|Twin-arginine translocation (Tat) system M00336
M00342: Bacterial proteasome	C	Structural complex|Genetic information processing|Proteasome|Bacterial proteasome M00342

M00343: Archaeal proteasome	C	Structural complex|Genetic information processing|Proteasome|Archaeal proteasome M00343
M00378: F420 biosynthesis	C	Pathway module|Energy metabolism|Methane metabolism|F420 biosynthesis M00378
M00439: Oligopeptide transport system	C	Structural complex|Environmental information processing|Peptide and nickel transport system|Oligopeptide transport system M00439
M00459: VicK-VicR (cell wall metabolism) two-component regulatory system	C	Functional set|Environmental information processing|Two-component regulatory system|VicK-VicR (cell wall metabolism) two-component regulatory system M00459
M00460: MprB-MprA (maintenance of persistent infection) two-component regulatory system	C	Functional set|Environmental information processing|Two-component regulatory system|MprB-MprA (maintenance of persistent infection) two-component regulatory system M00460
M00461: MtrB-MtrA (osmotic stress response) two-component regulatory system	C	Functional set|Environmental information processing|Two-component regulatory system|MtrB-MtrA (osmotic stress response) two-component regulatory system M00461
M00462: PrrB-PrrA (intracellular multiplication) two-component regulatory system	C	Functional set|Environmental information processing|Two-component regulatory system|PrrB-PrrA (intracellular multiplication) two-component regulatory system M00462
M00466: NblS-NblR (photosynthesis) two-component regulatory system	C	Functional set|Environmental information processing|Two-component regulatory system|NblS-NblR (photosynthesis) two-component regulatory system M00466
M00467: SasA-RpaAB (circadian timing mediating) two-component regulatory system	C	Functional set|Environmental information processing|Two-component regulatory system|SasA-RpaAB (circadian timing mediating) two-component regulatory system M00467
M00478: DegS-DegU (multicellular behavior control) two-component regulatory system	C	Functional set|Environmental information processing|Two-component regulatory system|DegS-DegU (multicellular behavior control) two-component regulatory system M00478
M00483: NreB-NreC (dissimilatory nitrate/nitrite reduction) two-component regulatory system	C	Functional set|Environmental information processing|Two-component regulatory system|NreB-NreC (dissimilatory nitrate/nitrite reduction) two-component regulatory system M00483
M00500: AtoS-AtoC (cPHB biosynthesis) two-component regulatory system	C	Functional set|Environmental information processing|Two-component regulatory system|AtoS-AtoC (cPHB biosynthesis) two-component regulatory system M00500
M00509: WspE-WspRF (chemosensory) two-component regulatory system	C	Functional set|Environmental information processing|Two-component regulatory system|WspE-WspRF (chemosensory) two-component regulatory system M00509
M00549: Nucleotide sugar biosynthesis, glucose => UDP-glucose	C	Pathway module|Carbohydrate and lipid metabolism|Other carbohydrate metabolism|Nucleotide sugar biosynthesis, glucose => UDP-glucose M00549
M00601: Putative chitobiose transport system	C	Structural complex|Environmental information processing|Saccharide, polyol, and lipid transport system|Putative chitobiose transport system M00601
M00633: Semi-phosphorylative Entner-Doudoroff pathway, gluconate/galactonate => glycerate-3P	C	Pathway module|Carbohydrate and lipid metabolism|Central carbohydrate metabolism|Semi-phosphorylative Entner-Doudoroff pathway, gluconate/galactonate => glycerate-3P M00633
M00695: cAMP signaling	C	Functional set|Cellular processes|Cell signaling|cAMP signaling M00695
M00725: Cationic antimicrobial peptide (CAMP) resistance, dltABCD operon	C	Signature module|Gene set|Drug resistance|Cationic antimicrobial peptide (CAMP) resistance, dltABCD operon M00725
M00728: Cationic antimicrobial peptide (CAMP) resistance, envelope protein folding and degrading factors DegP and DsbA	C	Functional set|Environmental information processing|Drug resistance|Cationic antimicrobial peptide (CAMP) resistance, envelope protein folding and degrading factors DegP and DsbA M00728
M00763: Ornithine biosynthesis, mediated by LysW, glutamate => ornithine	C	Pathway module|Nucleotide and amino acid metabolism|Arginine and proline metabolism|Ornithine biosynthesis, mediated by LysW, glutamate => ornithine M00763
M00767:Multidrug resistance, efflux pump OqxAB	C	Functional set|Environmental information processing|Drug efflux transporter/pump|Multidrug resistance, efflux pump OqxAB M00767
M00028: Ornithine biosynthesis, glutamate => ornithine	DWH	Pathway module|Nucleotide and amino acid metabolism|Arginine and proline metabolism|Ornithine biosynthesis, glutamate => ornithine M00028
M00030: Lysine biosynthesis, AAA pathway, 2-oxoglutarate => 2-aminoadipate => lysine	DWH	Pathway module|Nucleotide and amino acid metabolism|Lysine metabolism|Lysine biosynthesis, AAA pathway, 2-oxoglutarate => 2-aminoadipate => lysine M00030
M00060: Lipopolysaccharide biosynthesis, KDO2-lipid A	DWH	Pathway module|Carbohydrate and lipid metabolism|Lipopolysaccharide metabolism|Lipopolysaccharide biosynthesis, KDO2-lipid A M00060
M00123: Biotin biosynthesis, pimeloyl-ACP/CoA => biotin	DWH	Pathway module|Nucleotide and amino acid metabolism|Cofactor and vitamin biosynthesis|Biotin biosynthesis, pimeloyl-ACP/CoA => biotin M00123

M00136: GABA biosynthesis, prokaryotes, putrescine => GABA	DWH	Pathway module|Nucleotide and amino acid metabolism|Polyamine biosynthesis|GABA biosynthesis, prokaryotes, putrescine => GABA M00136
M00140: C1-unit interconversion, prokaryotes	DWH	Pathway module|Nucleotide and amino acid metabolism|Cofactor and vitamin biosynthesis|C1-unit interconversion, prokaryotes M00140
M00193: Putative spermidine/putrescine transport system	DWH	Structural complex|Environmental information processing|Mineral and organic ion transport system|Putative spermidine/putrescine transport system M00193
M00194: Maltose/maltodextrin transport system	DWH	Structural complex|Environmental information processing|Saccharide, polyol, and lipid transport system|Maltose/maltodextrin transport system M00194
M00200: Putative sorbitol/mannitol transport system	DWH	Structural complex|Environmental information processing|Saccharide, polyol, and lipid transport system|Putative sorbitol/mannitol transport system M00200
M00208: Glycine betaine/proline transport system	DWH	Structural complex|Environmental information processing|Mineral and organic ion transport system|Glycine betaine/proline transport system M00208
M00232: General L-amino acid transport system	DWH	Structural complex|Environmental information processing|Phosphate and amino acid transport system|General L-amino acid transport system M00232
M00235: Arginine/ornithine transport system	DWH	Structural complex|Environmental information processing|Phosphate and amino acid transport system|Arginine/ornithine transport system M00235
M00236: Putative polar amino acid transport system	DWH	Structural complex|Environmental information processing|Phosphate and amino acid transport system|Putative polar amino acid transport system M00236
M00250: Lipopolysaccharide transport system	DWH	Structural complex|Environmental information processing|ABC-2 type and other transport systems|Lipopolysaccharide transport system M00250
M00299: Spermidine/putrescine transport system	DWH	Structural complex|Environmental information processing|Mineral and organic ion transport system|Spermidine/putrescine transport system M00299
M00300: Putrescine transport system	DWH	Structural complex|Environmental information processing|Mineral and organic ion transport system|Putrescine transport system M00300
M00308: Semi-phosphorylative Entner-Doudoroff pathway, gluconate => glycerate-3P	DWH	Pathway module|Carbohydrate and lipid metabolism|Central carbohydrate metabolism|Semi-phosphorylative Entner-Doudoroff pathway, gluconate => glycerate-3P M00308
M00323: Urea transport system	DWH	Structural complex|Environmental information processing|Phosphate and amino acid transport system|Urea transport system M00323
M00334: Type VI secretion system	DWH	Structural complex|Environmental information processing|Bacterial secretion system|Type VI secretion system M00334
M00377: Reductive acetyl-CoA pathway (Wood-Ljungdahl pathway)	DWH	Pathway module|Energy metabolism|Carbon fixation|Reductive acetyl-CoA pathway (Wood-Ljungdahl pathway) M00377
M00442: Putative hydroxymethylpyrimidine transport system	DWH	Structural complex|Environmental information processing|Mineral and organic ion transport system|Putative hydroxymethylpyrimidine transport system M00442
M00491: arabinogalactan oligomer/maltooligosaccharide transport system	DWH	Structural complex|Environmental information processing|Saccharide, polyol, and lipid transport system|arabinogalactan oligomer/maltooligosaccharide transport system M00491
M00505: KinB-AlgB (alginate production) two-component regulatory system	DWH	Functional set|Environmental information processing|Two-component regulatory system|KinB-AlgB (alginate production) two-component regulatory system M00505
M00517: RpfC-RpfG (cell-to-cell signaling) two-component regulatory system	DWH	Functional set|Environmental information processing|Two-component regulatory system|RpfC-RpfG (cell-to-cell signaling) two-component regulatory system M00517
M00552: D-galactonate degradation, De Ley-Doudoroff pathway, D-galactonate => glycerate-3P	DWH	Pathway module|Carbohydrate and lipid metabolism|Other carbohydrate metabolism|D-galactonate degradation, De Ley-Doudoroff pathway, D-galactonate => glycerate-3P M00552
M00573: Biotin biosynthesis, BioI pathway, long-chain-acyl-ACP => pimeloyl-ACP => biotin	DWH	Pathway module|Nucleotide and amino acid metabolism|Cofactor and vitamin biosynthesis|Biotin biosynthesis, BioI pathway, long-chain-acyl-ACP => pimeloyl-ACP => biotin M00573
M00577: Biotin biosynthesis, BioW pathway, pimelate => pimeloyl-CoA => biotin	DWH	Pathway module|Nucleotide and amino acid metabolism|Cofactor and vitamin biosynthesis|Biotin biosynthesis, BioW pathway, pimelate => pimeloyl-CoA => biotin M00577
M00602: Arabinosaccharide transport system	DWH	Structural complex|Environmental information processing|Saccharide, polyol, and lipid transport system|Arabinosaccharide transport system M00602
M00605: Glucose/mannose transport system	DWH	Structural complex|Environmental information processing|Saccharide, polyol, and lipid transport system|Glucose/mannose transport system M00605
M00623: Phthalate degradation, phthalate => protocatechuate	DWH	Pathway module|Secondary metabolism|Aromatics degradation|Phthalate degradation, phthalate => protocatechuate M00623
M00637: Anthranilate degradation, anthranilate => catechol	DWH	Pathway module|Secondary metabolism|Aromatics degradation|Anthranilate degradation, anthranilate => catechol M00637
M00641: Multidrug resistance, efflux pump MexEF-OprN	DWH	Signature module|Gene set|Drug resistance|Multidrug resistance, efflux pump MexEF-OprN M00641

M00699: Multidrug resistance, efflux pump AmeABC	DWH	Functional set|Environmental information processing|Drug efflux transporter/pump|Multidrug resistance, efflux pump AmeABC M00699
M00718: Multidrug resistance, efflux pump MexAB-OprM	DWH	Signature module|Gene set|Drug resistance|Multidrug resistance, efflux pump MexAB-OprM M00718
M00721: Cationic antimicrobial peptide (CAMP) resistance, arnBCADTEF operon	DWH	Functional set|Environmental information processing|Drug resistance|Cationic antimicrobial peptide (CAMP) resistance, arnBCADTEF operon M00721
M00723: Cationic antimicrobial peptide (CAMP) resistance, phosphoethanolamine transferase EptB	DWH	Functional set|Environmental information processing|Drug resistance|Cationic antimicrobial peptide (CAMP) resistance, phosphoethanolamine transferase EptB M00723
M00724: Cationic antimicrobial peptide (CAMP) resistance, palmitoyl transferase PagP	DWH	Functional set|Environmental information processing|Drug resistance|Cationic antimicrobial peptide (CAMP) resistance, palmitoyl transferase PagP M00724
M00742: Aminoglycoside resistance, protease FtsH	DWH	Functional set|Environmental information processing|Drug resistance|Aminoglycoside resistance, protease FtsH M00742
M00744: Cationic antimicrobial peptide (CAMP) resistance, protease PgtE	DWH	Signature module|Gene set|Drug resistance|Cationic antimicrobial peptide (CAMP) resistance, protease PgtE M00744
M00042: Catecholamine biosynthesis, tyrosine => dopamine => noradrenaline => adrenaline	I	Pathway module|Nucleotide and amino acid metabolism|Aromatic amino acid metabolism|Catecholamine biosynthesis, tyrosine => dopamine => noradrenaline => adrenaline M00042
M00063: CMP-KDO biosynthesis	I	Pathway module|Carbohydrate and lipid metabolism|Lipopolysaccharide metabolism|CMP-KDO biosynthesis M00063
M00064: ADP-L-glycero-D-manno-heptose biosynthesis	I	Pathway module|Carbohydrate and lipid metabolism|Lipopolysaccharide metabolism|ADP-L-glycero-D-manno-heptose biosynthesis M00064
M00066: Lactosylceramide biosynthesis	I	Pathway module|Carbohydrate and lipid metabolism|Lipid metabolism|Lactosylceramide biosynthesis M00066
M00144: NADH:quinone oxidoreductase, prokaryotes	I	Structural complex|Energy metabolism|ATP synthesis|NADH:quinone oxidoreductase, prokaryotes M00144
M00155: Cytochrome c oxidase, prokaryotes	I	Structural complex|Energy metabolism|ATP synthesis|Cytochrome c oxidase, prokaryotes M00155
M00156: Cytochrome c oxidase, cbb3-type	I	Structural complex|Energy metabolism|ATP synthesis|Cytochrome c oxidase, cbb3-type M00156
M00166: Reductive pentose phosphate cycle, ribulose-5P => glyceraldehyde-3P	I	Pathway module|Energy metabolism|Carbon fixation|Reductive pentose phosphate cycle, ribulose-5P => glyceraldehyde-3P M00166
M00210: Phospholipid transport system	I	Structural complex|Environmental information processing|Saccharide, polyol, and lipid transport system|Phospholipid transport system M00210
M00211: Putative ABC transport system	I	Structural complex|Environmental information processing|Saccharide, polyol, and lipid transport system|Putative ABC transport system M00211
M00258: Putative ABC transport system	I	Structural complex|Environmental information processing|ABC-2 type and other transport systems|Putative ABC transport system M00258
M00339: RaxAB-RaxC type I secretion system	I	Structural complex|Environmental information processing|Bacterial secretion system|RaxAB-RaxC type I secretion system M00339
M00346: Formaldehyde assimilation, serine pathway	I	Pathway module|Energy metabolism|Methane metabolism|Formaldehyde assimilation, serine pathway M00346
M00444: PhoQ-PhoP (magnesium transport) two-component regulatory system	I	Functional set|Environmental information processing|Two-component regulatory system|PhoQ-PhoP (magnesium transport) two-component regulatory system M00444
M00447: CpxA-CpxR (envelope stress response) two-component regulatory system	I	Functional set|Environmental information processing|Two-component regulatory system|CpxA-CpxR (envelope stress response) two-component regulatory system M00447
M00471: NarX-NarL (nitrate respiration) two-component regulatory system	I	Functional set|Environmental information processing|Two-component regulatory system|NarX-NarL (nitrate respiration) two-component regulatory system M00471
M00473: UhpB-UhpA (hexose phosphates uptake) two-component regulatory system	I	Functional set|Environmental information processing|Two-component regulatory system|UhpB-UhpA (hexose phosphates uptake) two-component regulatory system M00473
M00492: LytS-LytR two-component regulatory system	I	Functional set|Environmental information processing|Two-component regulatory system|LytS-LytR two-component regulatory system M00492
M00532: Photorespiration	I	Pathway module|Carbohydrate and lipid metabolism|Other carbohydrate metabolism|Photorespiration M00532
M00550: Ascorbate degradation, ascorbate => D-xylulose-5P	I	Pathway module|Nucleotide and amino acid metabolism|Cofactor and vitamin biosynthesis|Ascorbate degradation, ascorbate => D-xylulose-5P M00550
M00571: AlgE-type Mannuronan C-5-Epimerase transport system	I	Structural complex|Environmental information processing|Bacterial secretion system|AlgE-type Mannuronan C-5-Epimerase transport system M00571

M00596: Dissimilatory sulfate reduction, sulfate => H2S	I	Pathway module|Energy metabolism|Sulfur metabolism|Dissimilatory sulfate reduction, sulfate => H2S M00596
M00720: Multidrug resistance, efflux pump VexEF-TolC	I	Structural complex|Environmental information processing|Drug efflux transporter/pump|Multidrug resistance, efflux pump VexEF-TolC M00720
M00727: Cationic antimicrobial peptide (CAMP) resistance, N-acetylmuramoyl-L-alanine amidase AmiA and AmiC	I	Functional set|Environmental information processing|Drug resistance|Cationic antimicrobial peptide (CAMP) resistance, N-acetylmuramoyl-L-alanine amidase AmiA and AmiC M00727
M00740: Methylaspartate cycle	I	Pathway module|Carbohydrate and lipid metabolism|Other carbohydrate metabolism|Methylaspartate cycle M00740
M00771: FitF-FitH (insecticidal toxin regulation) two-component regulatory system	I	Functional set|Environmental information processing|Two-component regulatory system|FitF-FitH (insecticidal toxin regulation) two-component regulatory system M00771
M00001: Glycolysis (Embden-Meyerhof pathway), glucose => pyruvate	M	Pathway module|Carbohydrate and lipid metabolism|Central carbohydrate metabolism|Glycolysis (Embden-Meyerhof pathway), glucose => pyruvate M00001
M00002: Glycolysis, core module involving three-carbon compounds	M	Pathway module|Carbohydrate and lipid metabolism|Central carbohydrate metabolism|Glycolysis, core module involving three-carbon compounds M00002
M00004: Pentose phosphate pathway (Pentose phosphate cycle)	M	Pathway module|Carbohydrate and lipid metabolism|Central carbohydrate metabolism|Pentose phosphate pathway (Pentose phosphate cycle) M00004
M00009: Citrate cycle (TCA cycle, Krebs cycle)	M	Pathway module|Carbohydrate and lipid metabolism|Central carbohydrate metabolism|Citrate cycle (TCA cycle, Krebs cycle) M00009
M00011: Citrate cycle, second carbon oxidation, 2-oxoglutarate => oxaloacetate	M	Pathway module|Carbohydrate and lipid metabolism|Central carbohydrate metabolism|Citrate cycle, second carbon oxidation, 2-oxoglutarate => oxaloacetate M00011
M00022: Shikimate pathway, phosphoenolpyruvate + erythrose-4P => chorismate	M	Pathway module|Nucleotide and amino acid metabolism|Aromatic amino acid metabolism|Shikimate pathway, phosphoenolpyruvate + erythrose-4P => chorismate M00022
M00023: Tryptophan biosynthesis, chorismate => tryptophan	M	Pathway module|Nucleotide and amino acid metabolism|Aromatic amino acid metabolism|Tryptophan biosynthesis, chorismate => tryptophan M00023
M00035: Methionine degradation	M	Pathway module|Nucleotide and amino acid metabolism|Cysteine and methionine metabolism|Methionine degradation M00035
M00051: Uridine monophosphate biosynthesis, glutamine (+ PRPP) => UMP	M	Pathway module|Nucleotide and amino acid metabolism|Pyrimidine metabolism|Uridine monophosphate biosynthesis, glutamine (+ PRPP) => UMP M00051
M00052: Pyrimidine ribonucleotide biosynthesis, UMP => UDP/UTP,CDP/CTP	M	Pathway module|Nucleotide and amino acid metabolism|Pyrimidine metabolism|Pyrimidine ribonucleotide biosynthesis, UMP => UDP/UTP,CDP/CTP M00052
M00080: Lipopolysaccharide biosynthesis, inner core => outer core => O-antigen	M	Pathway module|Carbohydrate and lipid metabolism|Lipopolysaccharide metabolism|Lipopolysaccharide biosynthesis, inner core => outer core => O-antigen M00080
M00115: NAD biosynthesis, aspartate => NAD	M	Pathway module|Nucleotide and amino acid metabolism|Cofactor and vitamin biosynthesis|NAD biosynthesis, aspartate => NAD M00115
M00120: Coenzyme A biosynthesis, pantothenate => CoA	M	Pathway module|Nucleotide and amino acid metabolism|Cofactor and vitamin biosynthesis|Coenzyme A biosynthesis, pantothenate => CoA M00120
M00159: V-type ATPase, prokaryotes	M	Structural complex|Energy metabolism|ATP synthesis|V-type ATPase, prokaryotes M00159
M00173: Reductive citrate cycle (Arnon-Buchanan cycle)	M	Pathway module|Energy metabolism|Carbon fixation|Reductive citrate cycle (Arnon-Buchanan cycle) M00173
M00178: Ribosome, bacteria	M	Structural complex|Genetic information processing|Ribosome|Ribosome, bacteria M00178
M00183: RNA polymerase, bacteria	M	Structural complex|Genetic information processing|RNA polymerase|RNA polymerase, bacteria M00183
M00222: Phosphate transport system	M	Structural complex|Environmental information processing|Phosphate and amino acid transport system|Phosphate transport system M00222
M00242: Zinc transport system	M	Structural complex|Environmental information processing|Metallic cation, iron-siderophore and vitamin B12 transport system|Zinc transport system M00242
M00255: Lipoprotein-releasing system	M	Structural complex|Environmental information processing|ABC-2 type and other transport systems|Lipoprotein-releasing system M00255
M00256: Cell division transport system	M	Structural complex|Environmental information processing|ABC-2 type and other transport systems|Cell division transport system M00256
M00260: DNA polymerase III complex, bacteria	M	Structural complex|Genetic information processing|DNA polymerase|DNA polymerase III complex, bacteria M00260
M00273: PTS system, fructose-specific II component	M	Structural complex|Environmental information processing|Phosphotransferase system (PTS)|PTS system, fructose-specific II component M00273
M00338: Cysteine biosynthesis, homocysteine + serine => cysteine	M	Pathway module|Nucleotide and amino acid metabolism|Cysteine and methionine metabolism|Cysteine biosynthesis, homocysteine + serine => cysteine M00338
M00359: Aminoacyl-tRNA biosynthesis, eukaryotes	M	Functional set|Metabolism|Aminoacyl tRNA|Aminoacyl-tRNA biosynthesis, eukaryotes M00359
M00360: Aminoacyl-tRNA biosynthesis, prokaryotes	M	Functional set|Metabolism|Aminoacyl tRNA|Aminoacyl-tRNA biosynthesis, prokaryotes M00360

M00364: C10-C20 isoprenoid biosynthesis, bacteria	M	Pathway module|Carbohydrate and lipid metabolism|Terpenoid backbone biosynthesis|C10-C20 isoprenoid biosynthesis, bacteria M00364
M00365: C10-C20 isoprenoid biosynthesis, archaea	M	Pathway module|Carbohydrate and lipid metabolism|Terpenoid backbone biosynthesis|C10-C20 isoprenoid biosynthesis, archaea M00365
M00366: C10-C20 isoprenoid biosynthesis, plants	M	Pathway module|Carbohydrate and lipid metabolism|Terpenoid backbone biosynthesis|C10-C20 isoprenoid biosynthesis, plants M00366
M00394: RNA degradosome	M	Structural complex|Genetic information processing|RNA processing|RNA degradosome M00394
M00429: Competence-related DNA transformation transporter	M	Structural complex|Environmental information processing|Bacterial secretion system|Competence-related DNA transformation transporter M00429
M00434: PhoR-PhoB (phosphate starvation response) two-component regulatory system	M	Functional set|Environmental information processing|Two-component regulatory system|PhoR-PhoB (phosphate starvation response) two-component regulatory system M00434
M00499: HydH-HydG (metal tolerance) two-component regulatory system	M	Functional set|Environmental information processing|Two-component regulatory system|HydH-HydG (metal tolerance) two-component regulatory system M00499
M00519: YesM-YesN two-component regulatory system	M	Functional set|Environmental information processing|Two-component regulatory system|YesM-YesN two-component regulatory system M00519
M00565: Trehalose biosynthesis, D-glucose 1P => trehalose	M	Pathway module|Carbohydrate and lipid metabolism|Other carbohydrate metabolism|Trehalose biosynthesis, D-glucose 1P => trehalose M00565
M00611: Oxygenic photosynthesis in plants and cyanobacteria	M	Signature module|Gene set|Metabolic capacity|Oxygenic photosynthesis in plants and cyanobacteria M00611
M00614: Anoxygenic photosynthesis in green sulfur bacteria	M	Signature module|Gene set|Metabolic capacity|Anoxygenic photosynthesis in green sulfur bacteria M00614
M00618: Acetogen	M	Signature module|Gene set|Metabolic capacity|Acetogen M00618
M00620: Incomplete reductive citrate cycle, acetyl-CoA => oxoglutarate	M	Pathway module|Energy metabolism|Carbon fixation|Incomplete reductive citrate cycle, acetyl-CoA => oxoglutarate M00620
M00729: Fluoroquinolone resistance, gyrase-protecting protein Qnr	M	Functional set|Environmental information processing|Drug resistance|Fluoroquinolone resistance, gyrase-protecting protein Qnr M00729
M00010: Citrate cycle, first carbon oxidation, oxaloacetate => 2-oxoglutarate	OSC	Pathway module|Carbohydrate and lipid metabolism|Central carbohydrate metabolism|Citrate cycle, first carbon oxidation, oxaloacetate => 2-oxoglutarate M00010
M00012: Glyoxylate cycle	OSC	Pathway module|Carbohydrate and lipid metabolism|Other carbohydrate metabolism|Glyoxylate cycle M00012
M00013: Malonate semialdehyde pathway, propanoyl-CoA => acetyl-CoA	OSC	Pathway module|Carbohydrate and lipid metabolism|Other carbohydrate metabolism|Malonate semialdehyde pathway, propanoyl-CoA => acetyl-CoA M00013
M00027: GABA (gamma-Aminobutyrate) shunt	OSC	Pathway module|Nucleotide and amino acid metabolism|Other amino acid metabolism|GABA (gamma-Aminobutyrate) shunt M00027
M00036: Leucine degradation, leucine => acetoacetate + acetyl-CoA	OSC	Pathway module|Nucleotide and amino acid metabolism|Branched-chain amino acid metabolism|Leucine degradation, leucine => acetoacetate + acetyl-CoA M00036
M00045: Histidine degradation, histidine => N-formiminoglutamate => glutamate	OSC	Pathway module|Nucleotide and amino acid metabolism|Histidine metabolism|Histidine degradation, histidine => N-formiminoglutamate => glutamate M00045
M00046: Pyrimidine degradation, uracil => beta-alanine, thymine => 3-aminoisobutanoate	OSC	Pathway module|Nucleotide and amino acid metabolism|Pyrimidine metabolism|Pyrimidine degradation, uracil => beta-alanine, thymine => 3-aminoisobutanoate M00046
M00088: Ketone body biosynthesis, acetyl-CoA => acetoacetate/3-hydroxybutyrate/acetone	OSC	Pathway module|Carbohydrate and lipid metabolism|Fatty acid metabolism|Ketone body biosynthesis, acetyl-CoA => acetoacetate/3-hydroxybutyrate/acetone M00088
M00098: Acylglycerol degradation	OSC	Pathway module|Carbohydrate and lipid metabolism|Lipid metabolism|Acylglycerol degradation M00098
M00118: Glutathione biosynthesis, glutamate => glutathione	OSC	Pathway module|Nucleotide and amino acid metabolism|Cofactor and vitamin biosynthesis|Glutathione biosynthesis, glutamate => glutathione M00118
M00124: Pyridoxal biosynthesis, erythrose-4P => pyridoxal-5P	OSC	Pathway module|Nucleotide and amino acid metabolism|Cofactor and vitamin biosynthesis|Pyridoxal biosynthesis, erythrose-4P => pyridoxal-5P M00124
M00130: Inositol phosphate metabolism, PI=> PIP2 => Ins(1,4,5)P3 => Ins(1,3,4,5)P4	OSC	Pathway module|Carbohydrate and lipid metabolism|Lipid metabolism|Inositol phosphate metabolism, PI=> PIP2 => Ins(1,4,5)P3 => Ins(1,3,4,5)P4 M00130
M00135: GABA biosynthesis, eukaryotes, putrescine => GABA	OSC	Pathway module|Nucleotide and amino acid metabolism|Polyamine biosynthesis|GABA biosynthesis, eukaryotes, putrescine => GABA M00135
M00185: Sulfate transport system	OSC	Structural complex|Environmental information processing|Mineral and organic ion transport system|Sulfate transport system M00185
M00188: NitT/TauT family transport system	OSC	Structural complex|Environmental information processing|Mineral and organic ion transport system|NitT/TauT family transport system M00188

M00190: Iron(III) transport system	OSC	Structural complex|Environmental information processing|Mineral and organic ion transport system|Iron(III) transport system M00190
M00191: Thiamine transport system	OSC	Structural complex|Environmental information processing|Mineral and organic ion transport system|Thiamine transport system M00191
M00204: Trehalose/maltose transport system	OSC	Structural complex|Environmental information processing|Saccharide, polyol, and lipid transport system|Trehalose/maltose transport system M00204
M00215: D-Xylose transport system	OSC	Structural complex|Environmental information processing|Saccharide, polyol, and lipid transport system|D-Xylose transport system M00215
M00230: Glutamate/aspartate transport system	OSC	Structural complex|Environmental information processing|Phosphate and amino acid transport system|Glutamate/aspartate transport system M00230
M00234: Cystine transport system	OSC	Structural complex|Environmental information processing|Phosphate and amino acid transport system|Cystine transport system M00234
M00238: D-Methionine transport system	OSC	Structural complex|Environmental information processing|Phosphate and amino acid transport system|D-Methionine transport system M00238
M00247: Putative ABC transport system	OSC	Structural complex|Environmental information processing|Metallic cation, iron-siderophore and vitamin B12 transport system|Putative ABC transport system M00247
M00269: PTS system, sucrose-specific II component	OSC	Structural complex|Environmental information processing|Phosphotransferase system (PTS)|PTS system, sucrose-specific II component M00269
M00317: Manganese/iron transport system	OSC	Structural complex|Environmental information processing|Metallic cation, iron-siderophore and vitamin B12 transport system|Manganese/iron transport system M00317
M00324: Dipeptide transport system	OSC	Structural complex|Environmental information processing|Peptide and nickel transport system|Dipeptide transport system M00324
M00328: Hemophore/metalloprotease transport system	OSC	Structural complex|Environmental information processing|Bacterial secretion system|Hemophore/metalloprotease transport system M00328
M00332: Type III secretion system	OSC	Structural complex|Environmental information processing|Bacterial secretion system|Type III secretion system M00332
M00348: Glutathione transport system	OSC	Structural complex|Environmental information processing|Peptide and nickel transport system|Glutathione transport system M00348
M00349: Microcin C transport system	OSC	Structural complex|Environmental information processing|Peptide and nickel transport system|Microcin C transport system M00349
M00417: Cytochrome o ubiquinol oxidase	OSC	Structural complex|Energy metabolism|ATP synthesis|Cytochrome o ubiquinol oxidase M00417
M00435: Taurine transport system	OSC	Structural complex|Environmental information processing|Mineral and organic ion transport system|Taurine transport system M00435
M00436: Sulfonate transport system	OSC	Structural complex|Environmental information processing|Mineral and organic ion transport system|Sulfonate transport system M00436
M00445: EnvZ-OmpR (osmotic stress response) two-component regulatory system	OSC	Functional set|Environmental information processing|Two-component regulatory system|EnvZ-OmpR (osmotic stress response) two-component regulatory system M00445
M00446: RstB-RstA two-component regulatory system	OSC	Functional set|Environmental information processing|Two-component regulatory system|RstB-RstA two-component regulatory system M00446
M00453: QseC-QseB (quorum sensing) two-component regulatory system	OSC	Functional set|Environmental information processing|Two-component regulatory system|QseC-QseB (quorum sensing) two-component regulatory system M00453
M00474: RcsC-RcsD-RcsB (capsule synthesis) two-component regulatory system	OSC	Functional set|Environmental information processing|Two-component regulatory system|RcsC-RcsD-RcsB (capsule synthesis) two-component regulatory system M00474
M00475: BarA-UvrY (central carbon metabolism) two-component regulatory system	OSC	Functional set|Environmental information processing|Two-component regulatory system|BarA-UvrY (central carbon metabolism) two-component regulatory system M00475
M00477: EvgS-EvgA (acid and drug tolerance) two-component regulatory system	OSC	Functional set|Environmental information processing|Two-component regulatory system|EvgS-EvgA (acid and drug tolerance) two-component regulatory system M00477
M00513: LuxQN/CqsS-LuxU-LuxO (quorum sensing) two-component regulatory system	OSC	Functional set|Environmental information processing|Two-component regulatory system|LuxQN/CqsS-LuxU-LuxO (quorum sensing) two-component regulatory system M00513
M00531: Assimilatory nitrate reduction, nitrate => ammonia	OSC	Pathway module|Energy metabolism|Nitrogen metabolism|Assimilatory nitrate reduction, nitrate => ammonia M00531
M00533: Homoprotocatechuate degradation, homoprotocatechuate => 2-oxohept-3-enedioate	OSC	Pathway module|Nucleotide and amino acid metabolism|Aromatic amino acid metabolism|Homoprotocatechuate degradation, homoprotocatechuate => 2-oxohept-3-enedioate M00533
M00537: Xylene degradation, xylene => methylbenzoate	OSC	Pathway module|Secondary metabolism|Aromatics degradation|Xylene degradation, xylene => methylbenzoate M00537
M00538: Toluene degradation, toluene => benzoate	OSC	Pathway module|Secondary metabolism|Aromatics degradation|Toluene degradation, toluene => benzoate M00538

M00539: Cumate degradation, p-cumate => 2-oxopent-4-enoate + 2-methylpropanoate	OSC	Pathway module|Secondary metabolism|Aromatics degradation|Cumate degradation, p-cumate => 2-oxopent-4-enoate + 2-methylpropanoate M00539
M00540: Benzoate degradation, cyclohexanecarboxylic acid =>pimeloyl-CoA	OSC	Pathway module|Secondary metabolism|Aromatics degradation|Benzoate degradation, cyclohexanecarboxylic acid =>pimeloyl-CoA M00540
M00542: EHEC/EPEC pathogenicity signature, T3SS and effectors	OSC	Signature module|Gene set|Pathogenicity |EHEC/EPEC pathogenicity signature, T3SS and effectors M00542
M00546: Purine degradation, xanthine => urea	OSC	Pathway module|Nucleotide and amino acid metabolism|Purine metabolism|Purine degradation, xanthine => urea M00546
M00551: Benzoate degradation, benzoate => catechol / methylbenzoate => methylcatechol	OSC	Pathway module|Secondary metabolism|Aromatics degradation|Benzoate degradation, benzoate => catechol / methylbenzoate => methylcatechol M00551
M00555: Betaine biosynthesis, choline => betaine	OSC	Pathway module|Nucleotide and amino acid metabolism|Serine and threonine metabolism|Betaine biosynthesis, choline => betaine M00555
M00568: Catechol ortho-cleavage, catechol => 3-oxoadipate	OSC	Pathway module|Secondary metabolism|Aromatics degradation|Catechol ortho-cleavage, catechol => 3-oxoadipate M00568
M00574: Pertussis pathogenicity signature 1, pertussis toxin	OSC	Signature module|Gene set|Pathogenicity |Pertussis pathogenicity signature 1, pertussis toxin M00574
M00575: Pertussis pathogenicity signature 2, T1SS	OSC	Signature module|Gene set|Pathogenicity |Pertussis pathogenicity signature 2, T1SS M00575
M00580: Pentose phosphate pathway, archaea, fructose 6P => ribose 5P	OSC	Pathway module|Carbohydrate and lipid metabolism|Central carbohydrate metabolism|Pentose phosphate pathway, archaea, fructose 6P => ribose 5P M00580
M00590: Erythritol transport system	OSC	Structural complex|Environmental information processing|Saccharide, polyol, and lipid transport system|Erythritol transport system M00590
M00593: Inositol transport system	OSC	Structural complex|Environmental information processing|Saccharide, polyol, and lipid transport system|Inositol transport system M00593
M00616: Sulfate-sulfur assimilation	OSC	Signature module|Gene set|Metabolic capacity|Sulfate-sulfur assimilation M00616
M00622: Nicotinate degradation, nicotinate => fumarate	OSC	Pathway module|Nucleotide and amino acid metabolism|Cofactor and vitamin biosynthesis|Nicotinate degradation, nicotinate => fumarate M00622
M00638: Salicylate degradation, salicylate => gentisate	OSC	Pathway module|Secondary metabolism|Aromatics degradation|Salicylate degradation, salicylate => gentisate M00638
M00643: Multidrug resistance, efflux pump MexXY-OprM	OSC	Signature module|Gene set|Drug resistance|Multidrug resistance, efflux pump MexXY-OprM M00643
M00649: Multidrug resistance, efflux pump AdeABC	OSC	Signature module|Gene set|Drug resistance|Multidrug resistance, efflux pump AdeABC M00649
M00655: AdeS-AdeR two-component regulatory system	OSC	Functional set|Environmental information processing|Two-component regulatory system|AdeS-AdeR two-component regulatory system M00655
M00660: Xanthomonas spp pathogenicity signature, T3SS and effectors	OSC	Signature module|Gene set|Plant pathogenicity|Xanthomonas spp| pathogenicity signature, T3SS and effectors M00660
M00664: Nodulation	OSC	Signature module|Gene set|Symbiosis|Nodulation M00664
M00696: Multidrug resistance, efflux pump AcrEF-TolC	OSC	Signature module|Gene set|Drug resistance|Multidrug resistance, efflux pump AcrEF-TolC M00696
M00697: Multidrug resistance, efflux pump MdtEF-TolC	OSC	Signature module|Gene set|Drug resistance|Multidrug resistance, efflux pump MdtEF-TolC M00697
M00709: Macrolide resistance, MacAB-TolC transporter	OSC	Functional set|Environmental information processing|Drug efflux transporter/pump|Macrolide resistance, MacAB-TolC transporter M00709
M00722: Cationic antimicrobial peptide (CAMP) resistance, phosphoethanolamine transferase PmrC	OSC	Functional set|Environmental information processing|Drug resistance|Cationic antimicrobial peptide (CAMP) resistance, phosphoethanolamine transferase PmrC M00722
M00736: Nocardicin A biosynthesis, L-pHPG + arginine + serine => nocardicin A	OSC	Pathway module|Secondary metabolism|Biosynthesis of secondary metabolites|Nocardicin A biosynthesis, L-pHPG + arginine + serine => nocardicin A M00736
M00743: Aminoglycoside resistance, protease HtpX	OSC	Functional set|Environmental information processing|Drug resistance|Aminoglycoside resistance, protease HtpX M00743
M00107: Steroid hormone biosynthesis, cholesterol => prognenolone => progesterone	OSTPd	Pathway module|Carbohydrate and lipid metabolism|Sterol biosynthesis|Steroid hormone biosynthesis, cholesterol => prognenolone => progesterone M00107
M00117: Ubiquinone biosynthesis, prokaryotes, chorismate => ubiquinone	OSTPd	Pathway module|Nucleotide and amino acid metabolism|Cofactor and vitamin biosynthesis|Ubiquinone biosynthesis, prokaryotes, chorismate => ubiquinone M00117

M00133: Polyamine biosynthesis, arginine => agmatine => putrescine => spermidine	OSTPd	Pathway module|Nucleotide and amino acid metabolism|Polyamine biosynthesis|Polyamine biosynthesis, arginine => agmatine => putrescine => spermidine M00133
M00169: CAM (Crassulacean acid metabolism), light	OSTPd	Pathway module|Energy metabolism|Carbon fixation|CAM (Crassulacean acid metabolism), light M00169
M00172: C4-dicarboxylic acid cycle, NADP - malic enzyme type	OSTPd	Pathway module|Energy metabolism|Carbon fixation|C4-dicarboxylic acid cycle, NADP - malic enzyme type M00172
M00198: Putative sn-glycerol-phosphate transport system	OSTPd	Structural complex|Environmental information processing|Saccharide, polyol, and lipid transport system|Putative sn-glycerol-phosphate transport system M00198
M00223: Phosphonate transport system	OSTPd	Structural complex|Environmental information processing|Phosphate and amino acid transport system|Phosphonate transport system M00223
M00237: Branched-chain amino acid transport system	OSTPd	Structural complex|Environmental information processing|Phosphate and amino acid transport system|Branched-chain amino acid transport system M00237
M00283: PTS system, ascorbate-specific II component	OSTPd	Structural complex|Environmental information processing|Phosphotransferase system (PTS)|PTS system, ascorbate-specific II component M00283
M00325: alpha-Hemolysin/cyclolysin transport system	OSTPd	Structural complex|Environmental information processing|Bacterial secretion system|alpha-Hemolysin/cyclolysin transport system M00325
M00423: Molybdate/tungstate transport system	OSTPd	Structural complex|Environmental information processing|Mineral and organic ion transport system|Molybdate/tungstate transport system M00423
M00438: Nitrate/nitrite transport system	OSTPd	Structural complex|Environmental information processing|Mineral and organic ion transport system|Nitrate/nitrite transport system M00438
M00452: CusS-CusR (copper tolerance) two-component regulatory system	OSTPd	Functional set|Environmental information processing|Two-component regulatory system|CusS-CusR (copper tolerance) two-component regulatory system M00452
M00493: AlgZ-AlgR (alginate production) two-component regulatory system	OSTPd	Functional set|Environmental information processing|Two-component regulatory system|AlgZ-AlgR (alginate production) two-component regulatory system M00493
M00497: GlnL-GlnG (nitrogen regulation) two-component regulatory system	OSTPd	Functional set|Environmental information processing|Two-component regulatory system|GlnL-GlnG (nitrogen regulation) two-component regulatory system M00497
M00501: PilS-PilR (type 4 fimbriae synthesis) two-component regulatory system	OSTPd	Functional set|Environmental information processing|Two-component regulatory system|PilS-PilR (type 4 fimbriae synthesis) two-component regulatory system M00501
M00507: ChpA-ChpB/PilGH (chemosensory) two-component regulatory system	OSTPd	Functional set|Environmental information processing|Two-component regulatory system|ChpA-ChpB/PilGH (chemosensory) two-component regulatory system M00507
M00529: Denitrification, nitrate => nitrogen	OSTPd	Pathway module|Energy metabolism|Nitrogen metabolism|Denitrification, nitrate => nitrogen M00529
M00530: Dissimilatory nitrate reduction, nitrate => ammonia	OSTPd	Pathway module|Energy metabolism|Nitrogen metabolism|Dissimilatory nitrate reduction, nitrate => ammonia M00530
M00548: Benzene degradation, benzene => catechol	OSTPd	Pathway module|Secondary metabolism|Aromatics degradation|Benzene degradation, benzene => catechol M00548
M00607: Glycerol transport system	OSTPd	Structural complex|Environmental information processing|Saccharide, polyol, and lipid transport system|Glycerol transport system M00607
M00642: Multidrug resistance, efflux pump MexJK-OprM	OSTPd	Signature module|Gene set|Drug resistance|Multidrug resistance, efflux pump MexJK-OprM M00642
M00646: Multidrug resistance, efflux pump AcrAD-TolC	OSTPd	Functional set|Environmental information processing|Drug efflux transporter/pump|Multidrug resistance, efflux pump AcrAD-TolC M00646
M00647: Multidrug resistance, efflux pump AcrAB-TolC/SmeDEF	OSTPd	Functional set|Environmental information processing|Drug efflux transporter/pump|Multidrug resistance, efflux pump AcrAB-TolC/SmeDEF M00647
M00669: gamma-Hexachlorocyclohexane transport system	OSTPd	Structural complex|Environmental information processing|Saccharide, polyol, and lipid transport system|gamma-Hexachlorocyclohexane transport system M00669
M00670: Mce transport system	OSTPd	Structural complex|Environmental information processing|Saccharide, polyol, and lipid transport system|Mce transport system M00670
M00745: Imipenem resistance, repression of porin OprD	OSTPd	Signature module|Gene set|Drug resistance|Imipenem resistance, repression of porin OprD M00745
M00761: Undecaprenylphosphate alpha-L-Ara4N biosynthesis, UDP-GlcA => undecaprenyl phosphate alpha-L-Ara4N	OSTPd	Pathway module|Carbohydrate and lipid metabolism|Other carbohydrate metabolism|Undecaprenylphosphate alpha-L-Ara4N biosynthesis, UDP-GlcA => undecaprenyl phosphate alpha-L-Ara4N M00761
M00762: Copper-processing system	OSTPd	Structural complex|Environmental information processing|ABC-2 type and other transport systems|Copper-processing system M00762
M00768: Multidrug resistance, efflux pump GesABC	OSTPd	Functional set|Environmental information processing|Drug efflux transporter/pump|Multidrug resistance, efflux pump GesABC M00768
M00769: Multidrug resistance, efflux pump MexPQ-OpmE	OSTPd	Signature module|Gene set|Drug resistance|Multidrug resistance, efflux pump MexPQ-OpmE M00769
M00003: Gluconeogenesis, oxaloacetate => fructose-6P	OSTPm	Pathway module|Carbohydrate and lipid metabolism|Central carbohydrate metabolism|Gluconeogenesis, oxaloacetate => fructose-6P M00003

M00189: Molybdate transport system	OSTPm	Structural complex|Environmental information processing|Mineral and organic ion transport system|Molybdate transport system M00189
M00224: Fluoroquinolone transport system	OSTPm	Structural complex|Environmental information processing|ABC-2 type and other transport systems|Fluoroquinolone transport system M00224
M00280: PTS system, glucitol/sorbitol-specific II component	OSTPm	Structural complex|Environmental information processing|Phosphotransferase system (PTS)|PTS system, glucitol/sorbitol-specific II component M00280
M00433: Lysine biosynthesis, 2-oxoglutarate => 2-oxoadipate	OSTPm	Pathway module|Nucleotide and amino acid metabolism|Lysine metabolism|Lysine biosynthesis, 2-oxoglutarate => 2-oxoadipate M00433
M00450: BaeS-BaeR (envelope stress response) two-component regulatory system	OSTPm	Functional set|Environmental information processing|Two-component regulatory system|BaeS-BaeR (envelope stress response) two-component regulatory system M00450
M00481: LiaS-LiaR (cell wall stress response) two-component regulatory system	OSTPm	Functional set|Environmental information processing|Two-component regulatory system|LiaS-LiaR (cell wall stress response) two-component regulatory system M00481
M00608: 2-Oxocarboxylic acid chain extension, 2-oxoglutarate => 2-oxoadipate => 2-oxopimelate => 2-oxosuberate	OSTPm	Pathway module|Energy metabolism|Methane metabolism|2-Oxocarboxylic acid chain extension, 2-oxoglutarate => 2-oxoadipate => 2-oxopimelate => 2-oxosuberate M00608
M00025: Tyrosine biosynthesis, chorismate => tyrosine	OSTPu	Pathway module|Nucleotide and amino acid metabolism|Aromatic amino acid metabolism|Tyrosine biosynthesis, chorismate => tyrosine M00025
M00034: Methionine salvage pathway	OSTPu	Pathway module|Nucleotide and amino acid metabolism|Cysteine and methionine metabolism|Methionine salvage pathway M00034
M00093: Phosphatidylethanolamine (PE) biosynthesis, PA => PS => PE	OSTPu	Pathway module|Carbohydrate and lipid metabolism|Lipid metabolism|Phosphatidylethanolamine (PE) biosynthesis, PA => PS => PE M00093
M00122: Cobalamin biosynthesis, cobinamide => cobalamin	OSTPu	Pathway module|Nucleotide and amino acid metabolism|Cofactor and vitamin biosynthesis|Cobalamin biosynthesis, cobinamide => cobalamin M00122
M00150: Fumarate reductase, prokaryotes	OSTPu	Structural complex|Energy metabolism|ATP synthesis|Fumarate reductase, prokaryotes M00150
M00175: Nitrogen fixation, nitrogen => ammonia	OSTPu	Pathway module|Energy metabolism|Nitrogen metabolism|Nitrogen fixation, nitrogen => ammonia M00175
M00186: Tungstate transport system	OSTPu	Structural complex|Environmental information processing|Mineral and organic ion transport system|Tungstate transport system M00186
M00245: Cobalt/nickel transport system	OSTPu	Structural complex|Environmental information processing|Metallic cation, iron-siderophore and vitamin B12 transport system|Cobalt/nickel transport system M00245
M00246: Nickel transport system	OSTPu	Structural complex|Environmental information processing|Metallic cation, iron-siderophore and vitamin B12 transport system|Nickel transport system M00246
M00252: Lipooligosaccharide transport system	OSTPu	Structural complex|Environmental information processing|ABC-2 type and other transport systems|Lipooligosaccharide transport system M00252
M00265: PTS system, glucose-specific II component	OSTPu	Structural complex|Environmental information processing|Phosphotransferase system (PTS)|PTS system, glucose-specific II component M00265
M00266: PTS system, maltose and glucose-specific II component	OSTPu	Structural complex|Environmental information processing|Phosphotransferase system (PTS)|PTS system, maltose and glucose-specific II component M00266
M00275: PTS system, cellobiose-specific II component	OSTPu	Structural complex|Environmental information processing|Phosphotransferase system (PTS)|PTS system, cellobiose-specific II component M00275
M00279: PTS system, galactitol-specific II component	OSTPu	Structural complex|Environmental information processing|Phosphotransferase system (PTS)|PTS system, galactitol-specific II component M00279
M00309: Non-phosphorylative Entner-Doudoroff pathway, gluconate/galactonate => glycerate	OSTPu	Pathway module|Carbohydrate and lipid metabolism|Central carbohydrate metabolism|Non-phosphorylative Entner-Doudoroff pathway, gluconate/galactonate => glycerate M00309
M00330: Adhesin protein transport system	OSTPu	Structural complex|Environmental information processing|Bacterial secretion system|Adhesin protein transport system M00330
M00331: Type II general secretion system	OSTPu	Structural complex|Environmental information processing|Bacterial secretion system|Type II general secretion system M00331
M00344: Formaldehyde assimilation, xylulose monophosphate pathway	OSTPu	Pathway module|Energy metabolism|Methane metabolism|Formaldehyde assimilation, xylulose monophosphate pathway M00344
M00345: Formaldehyde assimilation, ribulose monophosphate pathway	OSTPu	Pathway module|Energy metabolism|Methane metabolism|Formaldehyde assimilation, ribulose monophosphate pathway M00345
M00356: Methanogenesis, methanol => methane	OSTPu	Pathway module|Energy metabolism|Methane metabolism|Methanogenesis, methanol => methane M00356
M00449: CreC-CreB (phosphate regulation) two-component regulatory system	OSTPu	Functional set|Environmental information processing|Two-component regulatory system|CreC-CreB (phosphate regulation) two-component regulatory system M00449
M00457: TctE-TctD (tricarboxylic acid transport) two-component regulatory system	OSTPu	Functional set|Environmental information processing|Two-component regulatory system|TctE-TctD (tricarboxylic acid transport) two-component regulatory system M00457

M00458: ResE-ResD (aerobic and anaerobic respiration) two-component regulatory system	OSTPu	Functional set|Environmental information processing|Two-component regulatory system|ResE-ResD (aerobic and anaerobic respiration) two-component regulatory system M00458
M00485: KinABCDE-Spo0FA (sporulation control) two-component regulatory system	OSTPu	Functional set|Environmental information processing|Two-component regulatory system|KinABCDE-Spo0FA (sporulation control) two-component regulatory system M00485
M00502: GlrK-GlrR (amino sugar metabolism) two-component regulatory system	OSTPu	Functional set|Environmental information processing|Two-component regulatory system|GlrK-GlrR (amino sugar metabolism) two-component regulatory system M00502
M00506: CheA-CheYBV (chemotaxis) two-component regulatory system	OSTPu	Functional set|Environmental information processing|Two-component regulatory system|CheA-CheYBV (chemotaxis) two-component regulatory system M00506
M00528: Nitrification, ammonia => nitrite	OSTPu	Pathway module|Energy metabolism|Nitrogen metabolism|Nitrification, ammonia => nitrite M00528
M00579: Phosphate acetyltransferase-acetate kinase pathway, acetyl-CoA => acetate	OSTPu	Pathway module|Energy metabolism|Carbon fixation|Phosphate acetyltransferase-acetate kinase pathway, acetyl-CoA => acetate M00579
M00017: Methionine biosynthesis, apartate => homoserine => methionine	Tb	Pathway module|Nucleotide and amino acid metabolism|Cysteine and methionine metabolism|Methionine biosynthesis, apartate => homoserine => methionine M00017
M00018: Threonine biosynthesis, aspartate => homoserine => threonine	Tb	Pathway module|Nucleotide and amino acid metabolism|Serine and threonine metabolism|Threonine biosynthesis, aspartate => homoserine => threonine M00018
M00021: Cysteine biosynthesis, serine => cysteine	Tb	Pathway module|Nucleotide and amino acid metabolism|Cysteine and methionine metabolism|Cysteine biosynthesis, serine => cysteine M00021
M00049: Adenine ribonucleotide biosynthesis, IMP => ADP,ATP	Tb	Pathway module|Nucleotide and amino acid metabolism|Purine metabolism|Adenine ribonucleotide biosynthesis, IMP => ADP,ATP M00049
M00077: Chondroitin sulfate degradation	Tb	Pathway module|Carbohydrate and lipid metabolism|Glycosaminoglycan metabolism|Chondroitin sulfate degradation M00077
M00078: Heparan sulfate degradation	Tb	Pathway module|Carbohydrate and lipid metabolism|Glycosaminoglycan metabolism|Heparan sulfate degradation M00078
M00081: Pectin degradation	Tb	Pathway module|Carbohydrate and lipid metabolism|Other carbohydrate metabolism|Pectin degradation M00081
M00127: Thiamine biosynthesis, AIR => thiamine-P/thiamine-2P	Tb	Pathway module|Nucleotide and amino acid metabolism|Cofactor and vitamin biosynthesis|Thiamine biosynthesis, AIR => thiamine-P/thiamine-2P M00127
M00129: Ascorbate biosynthesis, animals, glucose-1P => ascorbate	Tb	Pathway module|Nucleotide and amino acid metabolism|Cofactor and vitamin biosynthesis|Ascorbate biosynthesis, animals, glucose-1P => ascorbate M00129
M00153: Cytochrome d ubiquinol oxidase	Tb	Structural complex|Energy metabolism|ATP synthesis|Cytochrome d ubiquinol oxidase M00153
M00157: F-type ATPase, prokaryotes and chloroplasts	Tb	Structural complex|Energy metabolism|ATP synthesis|F-type ATPase, prokaryotes and chloroplasts M00157
M00176: Assimilatory sulfate reduction, sulfate => H2S	Tb	Pathway module|Energy metabolism|Sulfur metabolism|Assimilatory sulfate reduction, sulfate => H2S M00176
M00202: Oligogalacturonide transport system	Tb	Structural complex|Environmental information processing|Saccharide, polyol, and lipid transport system|Oligogalacturonide transport system M00202
M00225: Lysine/arginine/ornithine transport system	Tb	Structural complex|Environmental information processing|Phosphate and amino acid transport system|Lysine/arginine/ornithine transport system M00225
M00259: Heme transport system	Tb	Structural complex|Environmental information processing|ABC-2 type and other transport systems|Heme transport system M00259
M00267: PTS system, N-acetylglucosamine-specific II component	Tb	Structural complex|Environmental information processing|Phosphotransferase system (PTS)|PTS system, N-acetylglucosamine-specific II component M00267
M00320: Lipopolysaccharide export system	Tb	Structural complex|Environmental information processing|ABC-2 type and other transport systems|Lipopolysaccharide export system M00320
M00333: Type IV secretion system	Tb	Structural complex|Environmental information processing|Bacterial secretion system|Type IV secretion system M00333
M00454: KdpD-KdpE (potassium transport) two-component regulatory system	Tb	Functional set|Environmental information processing|Two-component regulatory system|KdpD-KdpE (potassium transport) two-component regulatory system M00454
M00498: NtrY-NtrX (nitrogen regulation) two-component regulatory system	Tb	Functional set|Environmental information processing|Two-component regulatory system|NtrY-NtrX (nitrogen regulation) two-component regulatory system M00498
M00512: CckA-CtrA/CpdR (cell cycle control) two-component regulatory system	Tb	Functional set|Environmental information processing|Two-component regulatory system|CckA-CtrA/CpdR (cell cycle control) two-component regulatory system M00512
M00520: ChvG-ChvI (acidity sensing) two-component regulatory system	Tb	Functional set|Environmental information processing|Two-component regulatory system|ChvG-ChvI (acidity sensing) two-component regulatory system M00520
M00523: RegB-RegA (redox response) two-component regulatory system	Tb	Functional set|Environmental information processing|Two-component regulatory system|RegB-RegA (redox response) two-component regulatory system M00523

M00524: FixL-FixJ (nitrogen fixation) two-component regulatory system	Tb	Functional set|Environmental information processing|Two-component regulatory system|FixL-FixJ (nitrogen fixation) two-component regulatory system M00524
M00525: Lysine biosynthesis, acetyl-DAP pathway, aspartate => lysine	Tb	Pathway module|Nucleotide and amino acid metabolism|Lysine metabolism|Lysine biosynthesis, acetyl-DAP pathway, aspartate => lysine M00525
M00526: Lysine biosynthesis, DAP dehydrogenase pathway, aspartate => lysine	Tb	Pathway module|Nucleotide and amino acid metabolism|Lysine metabolism|Lysine biosynthesis, DAP dehydrogenase pathway, aspartate => lysine M00526
M00527: Lysine biosynthesis, DAP aminotransferase pathway, aspartate => lysine	Tb	Pathway module|Nucleotide and amino acid metabolism|Lysine metabolism|Lysine biosynthesis, DAP aminotransferase pathway, aspartate => lysine M00527
M00541: Benzoyl-CoA degradation, benzoyl-CoA => 3-hydroxypimeloyl-CoA	Tb	Pathway module|Secondary metabolism|Aromatics degradation|Benzoyl-CoA degradation, benzoyl-CoA => 3-hydroxypimeloyl-CoA M00541
M00592: Inositol transport system	Tb	Structural complex|Environmental information processing|Saccharide, polyol, and lipid transport system|Inositol transport system M00592
M00595: Thiosulfate oxidation by SOX complex, thiosulfate => sulfate	Tb	Pathway module|Energy metabolism|Sulfur metabolism|Thiosulfate oxidation by SOX complex, thiosulfate => sulfate M00595
M00597: Anoxygenic photosystem II	Tb	Structural complex|Energy metabolism|Photosynthesis|Anoxygenic photosystem II M00597
M00627: beta-Lactam resistance, Bla system	Tb	Signature module|Gene set|Drug resistance|beta-Lactam resistance, Bla system M00627
M00628: beta-Lactam resistance, AmpC system	Tb	Functional set|Environmental information processing|Drug resistance|beta-Lactam resistance, AmpC system M00628
M00631: D-Galacturonate degradation (bacteria)	Tb	Pathway module|Carbohydrate and lipid metabolism|Other carbohydrate metabolism|D-Galacturonate degradation (bacteria) M00631
M00644: Multidrug resistance, efflux pump MexHI-OprD	Tb	Functional set|Environmental information processing|Drug efflux transporter/pump|Multidrug resistance, efflux pump MexHI-OprD M00644
M00668: Tetracycline resistance, TetA transporter	Tb	Functional set|Environmental information processing|Drug efflux transporter/pump|Tetracycline resistance, TetA transporter M00668
M00698: Multidrug resistance, efflux pump BpeEF-OprC	Tb	Signature module|Gene set|Drug resistance|Multidrug resistance, efflux pump BpeEF-OprC M00698
M00701: Multidrug resistance, efflux pump EmrAB	Tb	Functional set|Environmental information processing|Drug efflux transporter/pump|Multidrug resistance, efflux pump EmrAB M00701
M00008: Entner-Doudoroff pathway, glucose-6P => glyceraldehyde-3P + pyruvate	Tp	Pathway module|Carbohydrate and lipid metabolism|Central carbohydrate metabolism|Entner-Doudoroff pathway, glucose-6P => glyceraldehyde-3P + pyruvate M00008
M00019: Valine/isoleucine biosynthesis, pyruvate => valine / 2-oxobutanoate => isoleucine	Tp	Pathway module|Nucleotide and amino acid metabolism|Branched-chain amino acid metabolism|Valine/isoleucine biosynthesis, pyruvate => valine / 2-oxobutanoate => isoleucine M00019
M00024: Phenylalanine biosynthesis, chorismate => phenylalanine	Tp	Pathway module|Nucleotide and amino acid metabolism|Aromatic amino acid metabolism|Phenylalanine biosynthesis, chorismate => phenylalanine M00024
M00026: Histidine biosynthesis, PRPP => histidine	Tp	Pathway module|Nucleotide and amino acid metabolism|Histidine metabolism|Histidine biosynthesis, PRPP => histidine M00026
M00029: Urea cycle	Tp	Pathway module|Nucleotide and amino acid metabolism|Arginine and proline metabolism|Urea cycle M00029
M00032: Lysine degradation, lysine => saccharopine => acetoacetyl-CoA	Tp	Pathway module|Nucleotide and amino acid metabolism|Lysine metabolism|Lysine degradation, lysine => saccharopine => acetoacetyl-CoA M00032
M00040: Tyrosine biosynthesis, prephanate => pretyrosine => tyrosine	Tp	Pathway module|Nucleotide and amino acid metabolism|Aromatic amino acid metabolism|Tyrosine biosynthesis, prephanate => pretyrosine => tyrosine M00040
M00044: Tyrosine degradation, tyrosine => homogentisate	Tp	Pathway module|Nucleotide and amino acid metabolism|Aromatic amino acid metabolism|Tyrosine degradation, tyrosine => homogentisate M00044
M00048: Inosine monophosphate biosynthesis, PRPP + glutamine => IMP	Tp	Pathway module|Nucleotide and amino acid metabolism|Purine metabolism|Inosine monophosphate biosynthesis, PRPP + glutamine => IMP M00048
M00053: Pyrimidine deoxyribonuleotide biosynthesis, CDP/CTP => dCDP/dCTP,dTDP/dTTP	Tp	Pathway module|Nucleotide and amino acid metabolism|Pyrimidine metabolism|Pyrimidine deoxyribonuleotide biosynthesis, CDP/CTP => dCDP/dCTP,dTDP/dTTP M00053
M00082: Fatty acid biosynthesis, initiation	Tp	Pathway module|Carbohydrate and lipid metabolism|Fatty acid metabolism|Fatty acid biosynthesis, initiation M00082
M00083: Fatty acid biosynthesis, elongation	Tp	Pathway module|Carbohydrate and lipid metabolism|Fatty acid metabolism|Fatty acid biosynthesis, elongation M00083
M00087: beta-Oxidation	Tp	Pathway module|Carbohydrate and lipid metabolism|Fatty acid metabolism|beta-Oxidation M00087
M00095: C5 isoprenoid biosynthesis, mevalonate pathway	Tp	Pathway module|Carbohydrate and lipid metabolism|Terpenoid backbone biosynthesis|C5 isoprenoid biosynthesis, mevalonate pathway M00095
M00097: beta-Carotene biosynthesis, GGAP => beta-carotene	Tp	Pathway module|Carbohydrate and lipid metabolism|Other terpenoid biosynthesis|beta-Carotene biosynthesis, GGAP => beta-carotene M00097

M00104: Bile acid biosynthesis, cholesterol => cholate	Tp	Pathway module|Carbohydrate and lipid metabolism|Sterol biosynthesis|Bile acid biosynthesis, cholesterol => cholate M00104
M00114: Ascorbate biosynthesis, plants, glucose-6P => ascorbate	Tp	Pathway module|Nucleotide and amino acid metabolism|Cofactor and vitamin biosynthesis|Ascorbate biosynthesis, plants, glucose-6P => ascorbate M00114
M00119: Pantothenate biosynthesis, valine/L-aspartate => pantothenate	Tp	Pathway module|Nucleotide and amino acid metabolism|Cofactor and vitamin biosynthesis|Pantothenate biosynthesis, valine/L-aspartate => pantothenate M00119
M00121: Heme biosynthesis, glutamate => protoheme/siroheme	Tp	Pathway module|Nucleotide and amino acid metabolism|Cofactor and vitamin biosynthesis|Heme biosynthesis, glutamate => protoheme/siroheme M00121
M00126: Tetrahydrofolate biosynthesis, GTP => THF	Tp	Pathway module|Nucleotide and amino acid metabolism|Cofactor and vitamin biosynthesis|Tetrahydrofolate biosynthesis, GTP => THF M00126
M00128: Ubiquinone biosynthesis, eukaryotes, 4-hydroxybenzoate => ubiquinone	Tp	Pathway module|Nucleotide and amino acid metabolism|Cofactor and vitamin biosynthesis|Ubiquinone biosynthesis, eukaryotes, 4-hydroxybenzoate => ubiquinone M00128
M00131: Inositol phosphate metabolism, Ins(1,3,4,5)P4 => Ins(1,3,4)P3 => myo-inositol	Tp	Pathway module|Carbohydrate and lipid metabolism|Lipid metabolism|Inositol phosphate metabolism, Ins(1,3,4,5)P4 => Ins(1,3,4)P3 => myo-inositol M00131
M00134: Polyamine biosynthesis, arginine => ornithine => putrescine	Tp	Pathway module|Nucleotide and amino acid metabolism|Polyamine biosynthesis|Polyamine biosynthesis, arginine => ornithine => putrescine M00134
M00154: Cytochrome c oxidase	Tp	Structural complex|Energy metabolism|ATP synthesis|Cytochrome c oxidase M00154
M00168: CAM (Crassulacean acid metabolism), dark	Tp	Pathway module|Energy metabolism|Carbon fixation|CAM (Crassulacean acid metabolism), dark M00168
M00170: C4-dicarboxylic acid cycle, phosphoenolpyruvate carboxykinase type	Tp	Pathway module|Energy metabolism|Carbon fixation|C4-dicarboxylic acid cycle, phosphoenolpyruvate carboxykinase type M00170
M00249: Capsular polysaccharide transport system	Tp	Structural complex|Environmental information processing|ABC-2 type and other transport systems|Capsular polysaccharide transport system M00249
M00276: PTS system, mannose-specific II component	Tp	Structural complex|Environmental information processing|Phosphotransferase system (PTS)|PTS system, mannose-specific II component M00276
M00307: Pyruvate oxidation, pyruvate => acetyl-CoA	Tp	Pathway module|Carbohydrate and lipid metabolism|Central carbohydrate metabolism|Pyruvate oxidation, pyruvate => acetyl-CoA M00307
M00372: Abscisic acid biosynthesis, beta-carotene => abscisic acid	Tp	Pathway module|Carbohydrate and lipid metabolism|Other terpenoid biosynthesis|Abscisic acid biosynthesis, beta-carotene => abscisic acid M00372
M00373: Ethylmalonyl pathway	Tp	Pathway module|Carbohydrate and lipid metabolism|Other carbohydrate metabolism|Ethylmalonyl pathway M00373
M00374: Dicarboxylate-hydroxybutyrate cycle	Tp	Pathway module|Energy metabolism|Carbon fixation|Dicarboxylate-hydroxybutyrate cycle M00374
M00376: 3-Hydroxypropionate bi-cycle	Tp	Pathway module|Energy metabolism|Carbon fixation|3-Hydroxypropionate bi-cycle M00376
M00400: p97-Ufd1-Npl4 complex	Tp	Structural complex|Genetic information processing|Protein processing|p97-Ufd1-Npl4 complex M00400
M00432: Leucine biosynthesis, 2-oxoisovalerate => 2-oxoisocaproate	Tp	Pathway module|Nucleotide and amino acid metabolism|Branched-chain amino acid metabolism|Leucine biosynthesis, 2-oxoisovalerate => 2-oxoisocaproate M00432
M00515: FlrB-FlrC (polar flagellar synthesis) two-component regulatory system	Tp	Functional set|Environmental information processing|Two-component regulatory system|FlrB-FlrC (polar flagellar synthesis) two-component regulatory system M00515
M00535: Isoleucine biosynthesis, pyruvate => 2-oxobutanoate	Tp	Pathway module|Nucleotide and amino acid metabolism|Branched-chain amino acid metabolism|Isoleucine biosynthesis, pyruvate => 2-oxobutanoate M00535
M00545: Trans-cinnamate degradation, trans-cinnamate => acetyl-CoA	Tp	Pathway module|Nucleotide and amino acid metabolism|Aromatic amino acid metabolism|Trans-cinnamate degradation, trans-cinnamate => acetyl-CoA M00545
M00554: Nucleotide sugar biosynthesis, galactose => UDP-galactose	Tp	Pathway module|Carbohydrate and lipid metabolism|Other carbohydrate metabolism|Nucleotide sugar biosynthesis, galactose => UDP-galactose M00554
M00569: Catechol meta-cleavage, catechol => acetyl-CoA / 4-methylcatechol => propanoyl-CoA	Tp	Pathway module|Secondary metabolism|Aromatics degradation|Catechol meta-cleavage, catechol => acetyl-CoA / 4-methylcatechol => propanoyl-CoA M00569
M00570: Isoleucine biosynthesis, threonine => 2-oxobutanoate => isoleucine	Tp	Pathway module|Nucleotide and amino acid metabolism|Branched-chain amino acid metabolism|Isoleucine biosynthesis, threonine => 2-oxobutanoate => isoleucine M00570
M00572: Pimeloyl-ACP biosynthesis, BioC-BioH pathway, malonyl-ACP => pimeloyl-ACP	Tp	Pathway module|Nucleotide and amino acid metabolism|Cofactor and vitamin biosynthesis|Pimeloyl-ACP biosynthesis, BioC-BioH pathway, malonyl-ACP => pimeloyl-ACP M00572
M00603: Putative aldouronate transport system	Tp	Structural complex|Environmental information processing|Saccharide, polyol, and lipid transport system|Putative aldouronate transport system M00603
M00613: Anoxygenic photosynthesis in green nonsulfur bacteria	Tp	Signature module|Gene set|Metabolic capacity|Anoxygenic photosynthesis in green nonsulfur bacteria M00613

M00726: Cationic antimicrobial peptide (CAMP) resistance, lysyl-phosphatidylglycerol (L-PG) synthase MprF	Tp	Signature module|Gene set|Drug resistance|Cationic antimicrobial peptide (CAMP) resistance, lysyl-phosphatidylglycerol (L-PG) synthase MprF M00726
M00741: Propanoyl-CoA metabolism, propanoyl-CoA => succinyl-CoA	Tp	Pathway module|Carbohydrate and lipid metabolism|Other carbohydrate metabolism|Propanoyl-CoA metabolism, propanoyl-CoA => succinyl-CoA M00741
M00061: D-Glucuronate degradation	Tu	Pathway module|Carbohydrate and lipid metabolism|Other carbohydrate metabolism|D-Glucuronate degradation M00061
M00091: Phosphatidylcholine (PC) biosynthesis, PE => PC	Tu	Pathway module|Carbohydrate and lipid metabolism|Lipid metabolism|Phosphatidylcholine (PC) biosynthesis, PE => PC M00091
M00149: Succinate dehydrogenase, prokaryotes	Tu	Structural complex|Energy metabolism|ATP synthesis|Succinate dehydrogenase, prokaryotes M00149
M00151: Cytochrome bc1 complex respiratory unit	Tu	Structural complex|Energy metabolism|ATP synthesis|Cytochrome bc1 complex respiratory unit M00151
M00205: N-Acetylglucosamine transport system	Tu	Structural complex|Environmental information processing|Saccharide, polyol, and lipid transport system|N-Acetylglucosamine transport system M00205
M00251: Teichoic acid transport system	Tu	Structural complex|Environmental information processing|ABC-2 type and other transport systems|Teichoic acid transport system M00251
M00316: Manganese transport system	Tu	Structural complex|Environmental information processing|Metallic cation, iron-siderophore and vitamin B12 transport system|Manganese transport system M00316
M00504: DctB-DctD (C4-dicarboxylate transport) two-component regulatory system	Tu	Functional set|Environmental information processing|Two-component regulatory system|DctB-DctD (C4-dicarboxylate transport) two-component regulatory system M00504
M00511: PleC-PleD (cell fate control) two-component regulatory system	Tu	Functional set|Environmental information processing|Two-component regulatory system|PleC-PleD (cell fate control) two-component regulatory system M00511
M00606: N,N'-Diacetylchitobiose transport system	Tu	Structural complex|Environmental information processing|Saccharide, polyol, and lipid transport system|N,N'-Diacetylchitobiose transport system M00606
M00634: Oleandomycin transport system	Tu	Structural complex|Environmental information processing|ABC-2 type and other transport systems|Oleandomycin transport system M00634
M00704: Tetracycline resistance, efflux pump Tet38	Tu	Signature module|Gene set|Drug resistance|Tetracycline resistance, efflux pump Tet38 M00704
M00766: Streptomycin resistance, deactivating enzyme StrAB	Tu	Functional set|Environmental information processing|Drug resistance|Streptomycin resistance, deactivating enzyme StrAB M00766

[†] Column labelled "Habitat" represents the petroleum contaminated environment in which the corresponding KEGG Modules (as presented in column labelled "Differentially abundant KEGG Modules"), was found to be significantly differentially abundant by LEfSe using the one class, non-strict test (Please refer to Materials and methods, and Supplementary Table S3 for details). Acronyms represent the following habitats: A: Arctic, C: China oil refineries, I: India oil refineries, M: Mangrove, DWH: Marine sediments, OSC: Oil sands core, OSTPu: Oil sands tailings pond upper, OSTPm: Oil sands tailings pond median, OSTPd: Oil sands tailings pond deep, Tb: Taiga bottom active layer, Tu: Taiga upper active layer, Tp: Taiga permafrost layer.

[‡] KEGG Modules Hierarchy is described using the KEGG Modules Hierarchy as described in the KEGG Modules database (http://www.kegg.jp/kegg/module.html).

Supplementary data. Table S7. p-values of PERMANOVA results for pairwise comparison of taxonomic composition of all habitats using Bray-Curtis similarities.

	India oil refineries	Arctic	China oil refineries	Mangrove	Marine sediments	Taiga upper active layer	Taiga bottom active layer	Taiga permafrost layer	Oil sands core	Oil sands tailings pond upper	Oil sands tailings pond median	Oil sands tailings pond deep
**India oil refineries (I)**												
**Arctic (A)**	0.0007											
**China oil refineries (C)**	0.0015	0.0002										
**Mangrove (M)**	0.0258	0.0018	0.0048									
**Marine sediments (DWH)**	0.0025	0.0001	0.0001	0.0105								
**Taiga upper active layer (Tu)**	0.0267	0.0005	0.0012	0.029	0.0038							
**Taiga bottom active layer (Tb)**	0.0277	0.0005	0.0014	0.0312	0.0023	0.9426‡						
**Taiga permafrost layer (Tp)**	0.0301	0.001	0.0013	0.0575‡	0.0023	0.719‡	0.6054‡					
**Oil sands core (OSC)**	0.003	0.0001	0.0002	0.0081	0.0012	0.0031	0.0035	0.003				
**Oil sands tailings pond upper (OSTPu)**	0.0259	0.0006	0.0011	0.0296	0.0015	0.0262	0.029	0.0293	0.0034			
**Oil sands tailings pond median (OSTPm)**	0.029	0.0021	0.0048	0.0994‡	0.0084	0.0276	0.0281	0.0543‡	0.0089	0.7089‡		
**Oil sands tailings pond deep (OSTPd)**	0.0284	0.0009	0.0015	0.03	0.0029	0.0272	0.0316	0.0287	0.0041	0.2565‡	0.5163‡	

‡ P-values greater than 0.05 for PERMANOVA tests using Bray-Curtis similarity scores derived from taxonomic compositions between habitats signifying non-significant dissimilarity.

Supplementary data. Table S8. Significant bacterial interactions in oil contaminated environments detected by SparCC (positive or negative correlation value > 0.6, p-value < 0.01). (Rows with interactions mentioned in the text are filled in blue).

Habitat[†]	OTU1 Classification[‡]	OTU2 Classification[‡]	Correlation	*p*-value
A	p__Acidobacteria|c__Acidobacteriia|o__Acidobacteriales|f__Koribacteraceae|g__Candidatus_Koribacter|s__	p__Proteobacteria|c__Alphaproteobacteria|o__Rhizobiales|f__Bradyrhizobiaceae|g__|s__	0.606438	0.004
A	p__Acidobacteria|c__Acidobacteriia|o__Acidobacteriales|f__|g__|s__	p__Bacteroidetes|c__|o__|f__|g__|s__	0.610808	0.0088
A	p__Actinobacteria|c__Actinobacteria|o__Actinomycetales|f__Nocardioidaceae|g__Nocardioides|s__plantarum	p__Bacteroidetes|c__Flavobacteriia|o__Flavobacteriales|f__Flavobacteriaceae|g__|s__	0.62121	0.0059
A	p__Acidobacteria|c__Acidobacteriia|o__Acidobacteriales|f__|g__|s__	p__Proteobacteria|c__Gammaproteobacteria|o__Xanthomonadales|f__Sinobacteraceae|g__|s__	0.62358	0.0067
A	p__Acidobacteria|c__DA052|o__Ellin6513|f__|g__|s__	p__Proteobacteria|c__Gammaproteobacteria|o__Xanthomonadales|f__Sinobacteraceae|g__|s__	0.629303	0.0046
A	p__Actinobacteria|c__Acidimicrobiia|o__Acidimicrobiales|f__Iamiaceae|g__Iamia|s__	p__Actinobacteria|c__Actinobacteria|o__Actinomycetales|f__Nocardioidaceae|g__Nocardioides|s__plantarum	0.631826	0.0022
A	p__Acidobacteria|c__Acidobacteriia|o__Acidobacteriales|f__Acidobacteriaceae|g__Terriglobus|s__	p__Proteobacteria|c__Alphaproteobacteria|o__Rhizobiales|f__Bradyrhizobiaceae|g__|s__	0.63512	0.0036
A	p__Actinobacteria|c__Actinobacteria|o__Actinomycetales|f__Nocardioidaceae|g__Nocardioides|s__plantarum	p__Actinobacteria|c__Actinobacteria|o__Actinomycetales|f__Nocardioidaceae|g__|s__	0.646635	0.0034
A	p__Acidobacteria|c__Acidobacteriia|o__Acidobacteriales|f__Koribacteraceae|g__Candidatus_Koribacter|s__	p__Acidobacteria|c__DA052|o__Ellin6513|f__|g__|s__	0.654059	0.0025
A	p__Bacteroidetes|c__|o__|f__|g__|s__	p__Proteobacteria|c__Gammaproteobacteria|o__Xanthomonadales|f__Sinobacteraceae|g__|s__	0.657003	0.0024
A	p__Actinobacteria|c__Acidimicrobiia|o__Acidimicrobiales|f__Iamiaceae|g__Iamia|s__	p__Bacteroidetes|c__Flavobacteriia|o__Flavobacteriales|f__Flavobacteriaceae|g__|s__	0.702216	0.0011
A	p__Acidobacteria|c__DA052|o__Ellin6513|f__|g__|s__	p__Proteobacteria|c__Alphaproteobacteria|o__Rhizobiales|f__Bradyrhizobiaceae|g__|s__	0.723816	0.0013
A	p__Acidobacteria|c__Acidobacteriia|o__Acidobacteriales|f__Koribacteraceae|g__Candidatus_Koribacter|s__	p__Acidobacteria|c__Acidobacteriia|o__Acidobacteriales|f__|g__|s__	0.726325	0.0001
A	p__Actinobacteria|c__Actinobacteria|o__Actinomycetales|f__Microbacteriaceae|g__Microbacterium|s__	p__Actinobacteria|c__Actinobacteria|o__Actinomycetales|f__Micrococcaceae|g__|s__	0.736769	0.0005
A	p__Bacteroidetes|c__Saprospirae|o__Saprospirales|f__Chitinophagaceae|g__|s__	p__Proteobacteria|c__Alphaproteobacteria|o__Rhizobiales|f__Bradyrhizobiaceae|g__|s__	0.742711	0.0048
A	p__Acidobacteria|c__Acidobacteriia|o__Acidobacteriales|f__|g__|s__	p__Proteobacteria|c__Alphaproteobacteria|o__Rhizobiales|f__Bradyrhizobiaceae|g__|s__	0.769795	0.0007
A	p__Acidobacteria|c__Acidobacteriia|o__Acidobacteriales|f__Acidobacteriaceae|g__Terriglobus|s__	p__Acidobacteria|c__DA052|o__Ellin6513|f__|g__|s__	0.824712	0.0001
A	p__Acidobacteria|c__Acidobacteriia|o__Acidobacteriales|f__Acidobacteriaceae|g__Terriglobus|s__	p__Acidobacteria|c__Acidobacteriia|o__Acidobacteriales|f__|g__|s__	0.843217	0.0001
A	p__Acidobacteria|c__Acidobacteriia|o__Acidobacteriales|f__|g__|s__	p__Acidobacteria|c__DA052|o__Ellin6513|f__|g__|s__	0.877044	0
C	p__Actinobacteria|c__Actinobacteria|o__Actinomycetales|f__Dietziaceae|g__Dietzia|s__	p__Verrucomicrobia|c__Spartobacteria|o__Chthoniobacterales|f__Chthoniobacteraceae|g__|s__	-0.73237	0.0006
C	p__Actinobacteria|c__Actinobacteria|o__Actinomycetales|f__Mycobacteriaceae|g__Mycobacterium|s__	p__Gemmatimonadetes|c__Gemm-3|o__|f__|g__|s__	-0.71167	0.0034
C	p__Actinobacteria|c__Actinobacteria|o__Actinomycetales|f__Intrasporangiaceae|g__|s__	p__Gemmatimonadetes|c__Gemm-3|o__|f__|g__|s__	-0.70465	0.0019
C	p__Gemmatimonadetes|c__Gemm-3|o__|f__|g__|s__	p__Proteobacteria|c__Gammaproteobacteria|o__Xanthomonadales|f__Sinobacteraceae|g__|s__	-0.68534	0.0039
C	p__Actinobacteria|c__Actinobacteria|o__Actinomycetales|f__Nocardioidaceae|g__|s__	p__Cyanobacteria|c__Oscillatoriophycideae|o__Oscillatoriales|f__Phormidiaceae|g__Phormidium|s__	-0.68273	0.0007
C	p__Acidobacteria|c__Acidobacteria-6|o__iii1-15|f__|g__|s__	p__Actinobacteria|c__Actinobacteria|o__Actinomycetales|f__Dietziaceae|g__Dietzia|s__	-0.67274	0.0024
C	p__Chloroflexi|c__Ellin6529|o__|f__|g__|s__	p__Cyanobacteria|c__|o__|f__|g__|s__	-0.67055	0.0005
C	p__Chloroflexi|c__S085|o__|f__|g__|s__	p__Chloroflexi|c__TK10|o__AKYG885|f__Dolo_23|g__|s__	-0.66641	0.0002
C	p__Actinobacteria|c__Actinobacteria|o__Actinomycetales|f__Dietziaceae|g__Dietzia|s__	p__Verrucomicrobia|c__Spartobacteria|o__Chthoniobacterales|f__Chthoniobacteraceae|g__Chthoniobacter|s__	-0.66151	0.0016
C	p__Actinobacteria|c__Actinobacteria|o__Actinomycetales|f__Micrococcaceae|g__Arthrobacter|s__psychrolactophilus	p__Gemmatimonadetes|c__Gemm-5|o__|f__|g__|s__	-0.65436	0.0027
C	p__Actinobacteria|c__Actinobacteria|o__Actinomycetales|f__Dietziaceae|g__Dietzia|s__	p__Planctomycetes|c__Planctomycetia|o__Pirellulales|f__Pirellulaceae|g__|s__	-0.65032	0.0024
C	p__Actinobacteria|c__Acidimicrobiia|o__Acidimicrobiales|f__|g__|s__	p__Gemmatimonadetes|c__Gemm-5|o__|f__|g__|s__	-0.64924	0.0025
C	p__Actinobacteria|c__Actinobacteria|o__Actinomycetales|f__Geodermatophilaceae|g__|s__	p__Verrucomicrobia|c__Verrucomicrobiae|o__Verrucomicrobiales|f__Verrucomicrobiaceae|g__Luteolibacter|s__	-0.64791	0.0017
C	p__Acidobacteria|c__Acidobacteria-6|o__iii1-15|f__mb2424|g__|s__	p__Actinobacteria|c__Actinobacteria|o__Actinomycetales|f__Dietziaceae|g__Dietzia|s__	-0.63946	0.0029
C	p__Actinobacteria|c__Actinobacteria|o__Actinomycetales|f__Intrasporangiaceae|g__|s__	p__Proteobacteria|c__Alphaproteobacteria|o__Sphingomonadales|f__Sphingomonadaceae|g__Kaistobacter|s__	-0.63305	0.0041
C	p__Actinobacteria|c__Actinobacteria|o__Actinomycetales|f__Micrococcaceae|g__Arthrobacter|s__psychrolactophilus	p__Verrucomicrobia|c__Verrucomicrobiae|o__Verrucomicrobiales|f__Verrucomicrobiaceae|g__Luteolibacter|s__	-0.62693	0.0071

C	p__Acidobacteria|c__Chloracidobacteria|o__RB41|f__Ellin6075|g__|s__	p__Chloroflexi|c__Ktedonobacteria|o__Thermogemmatisporales|f__Thermogemmatisporaceae|g__|s__	-0.62566	0.0011
C	p__Acidobacteria|c__Chloracidobacteria|o__RB41|f__Ellin6075|g__|s__	p__Actinobacteria|c__Actinobacteria|o__Actinomycetales|f__Nocardioidaceae|g__|s__	-0.62425	0.0047
C	p__Actinobacteria|c__Actinobacteria|o__Actinomycetales|f__Microbacteriaceae|g__Microbacterium|s__	p__Verrucomicrobia|c__Spartobacteria|o__Chthoniobacterales|f__Chthoniobacteraceae|g__|s__	-0.62039	0.0048
C	p__Actinobacteria|c__Actinobacteria|o__Actinomycetales|f__Geodermatophilaceae|g__|s__	p__Cyanobacteria|c__Oscillatoriophycideae|o__Oscillatoriales|f__Phormidiaceae|g__Phormidium|s__	-0.61876	0.0012
C	p__Actinobacteria|c__Actinobacteria|o__Actinomycetales|f__Micromonosporaceae|g__Solwaraspora|s__	p__Gemmatimonadetes|c__Gemm-3|o__|f__|g__|s__	-0.61726	0.0072
C	p__Actinobacteria|c__Acidimicrobiia|o__Acidimicrobiales|f__|g__|s__	p__Actinobacteria|c__Thermoleophilia|o__Gaiellales|f__Gaiellaceae|g__|s__	0.604518	0.0013
C	p__Actinobacteria|c__Thermoleophilia|o__Solirubrobacterales|f__Solirubrobacteraceae|g__|s__	p__Proteobacteria|c__Deltaproteobacteria|o__Myxococcales|f__Myxococcaceae|g__Anaeromyxobacter|s__	0.605995	0.0041
C	p__Actinobacteria|c__Actinobacteria|o__Actinomycetales|f__Mycobacteriaceae|g__Mycobacterium|s__	p__Proteobacteria|c__Deltaproteobacteria|o__Myxococcales|f__Myxococcaceae|g__Anaeromyxobacter|s__	0.620749	0.0035
C	p__Actinobacteria|c__Actinobacteria|o__Actinomycetales|f__Mycobacteriaceae|g__Mycobacterium|s__	p__Chloroflexi|c__Ktedonobacteria|o__JG30-KF-AS9|f__|g__|s__	0.623933	0.0002
C	p__Actinobacteria|c__Thermoleophilia|o__Gaiellales|f__Gaiellaceae|g__|s__	p__Chloroflexi|c__Ktedonobacteria|o__Thermogemmatisporales|f__Thermogemmatisporaceae|g__|s__	0.626391	0.0042
C	p__Actinobacteria|c__Actinobacteria|o__Actinomycetales|f__Micrococcaceae|g__Arthrobacter|s__psychrolactophilus	p__Actinobacteria|c__Actinobacteria|o__Actinomycetales|f__Nocardioidaceae|g__|s__	0.628133	0.0062
C	p__Chloroflexi|c__Ktedonobacteria|o__JG30-KF-AS9|f__|g__|s__	p__Proteobacteria|c__Gammaproteobacteria|o__Xanthomonadales|f__Sinobacteraceae|g__|s__	0.629345	0.0002
C	p__Actinobacteria|c__Actinobacteria|o__Actinomycetales|f__Nocardioidaceae|g__|s__	p__Proteobacteria|c__Gammaproteobacteria|o__Xanthomonadales|f__Sinobacteraceae|g__|s__	0.636663	0.0061
C	p__Actinobacteria|c__Actinobacteria|o__Actinomycetales|f__Intrasporangiaceae|g__|s__	p__Actinobacteria|c__Thermoleophilia|o__Solirubrobacterales|f__Solirubrobacteraceae|g__|s__	0.64268	0.004
C	p__Actinobacteria|c__Actinobacteria|o__Actinomycetales|f__Mycobacteriaceae|g__Mycobacterium|s__	p__Actinobacteria|c__Thermoleophilia|o__Solirubrobacterales|f__Solirubrobacteraceae|g__|s__	0.646094	0.0062
C	p__Actinobacteria|c__Actinobacteria|o__Actinomycetales|f__Microbacteriaceae|g__Microbacterium|s__	p__Proteobacteria|c__Deltaproteobacteria|o__Myxococcales|f__Myxococcaceae|g__Anaeromyxobacter|s__	0.652514	0.0039
C	p__Chloroflexi|c__Thermomicrobia|o__JG30-KF-CM45|f__|g__|s__	p__Proteobacteria|c__Gammaproteobacteria|o__Xanthomonadales|f__Sinobacteraceae|g__|s__	0.658582	0.0064
C	p__Chloroflexi|c__Thermomicrobia|o__JG30-KF-CM45|f__|g__|s__	p__Proteobacteria|c__Gammaproteobacteria|o__Pseudomonadales|f__Moraxellaceae|g__Alkanindiges|s__	0.663016	0.0043
C	p__Actinobacteria|c__Actinobacteria|o__Actinomycetales|f__Nocardioidaceae|g__|s__	p__Chloroflexi|c__Ktedonobacteria|o__Thermogemmatisporales|f__Thermogemmatisporaceae|g__|s__	0.670211	0.002
C	p__Actinobacteria|c__Actinobacteria|o__Actinomycetales|f__Microbacteriaceae|g__Microbacterium|s__	p__Chloroflexi|c__Chloroflexi|o__Roseiflexales|f__Kouleothrixaceae|g__|s__	0.672805	0.0049
C	p__Actinobacteria|c__Actinobacteria|o__Actinomycetales|f__Microbacteriaceae|g__Microbacterium|s__	p__Actinobacteria|c__Thermoleophilia|o__Gaiellales|f__Gaiellaceae|g__|s__	0.674267	0.0044
C	p__Actinobacteria|c__Actinobacteria|o__Actinomycetales|f__Geodermatophilaceae|g__|s__	p__Actinobacteria|c__Actinobacteria|o__Actinomycetales|f__Nocardioidaceae|g__|s__	0.677651	0.0014
C	p__Acidobacteria|c__Solibacteres|o__Solibacterales|f__|g__|s__	p__Actinobacteria|c__Actinobacteria|o__Actinomycetales|f__Intrasporangiaceae|g__|s__	0.678031	0.0035
C	p__Acidobacteria|c__Solibacteres|o__Solibacterales|f__|g__|s__	p__Actinobacteria|c__Actinobacteria|o__Actinomycetales|f__Mycobacteriaceae|g__Mycobacterium|s__	0.683276	0.0038
C	p__Actinobacteria|c__Actinobacteria|o__Actinomycetales|f__Geodermatophilaceae|g__|s__	p__Actinobacteria|c__Actinobacteria|o__Actinomycetales|f__Microbacteriaceae|g__Microbacterium|s__	0.688042	0.0009
C	p__Acidobacteria|c__Acidobacteria-6|o__iii1-15|f__mb2424|g__|s__	p__Planctomycetes|c__Planctomycetia|o__Planctomycetales|f__Planctomycetaceae|g__Planctomyces|s__	0.689099	0.009
C	p__Bacteroidetes|c__|o__|f__|g__|s__	p__Planctomycetes|c__Planctomycetia|o__Planctomycetales|f__Planctomycetaceae|g__Planctomyces|s__	0.690796	0.0059
C	p__Actinobacteria|c__Actinobacteria|o__Actinomycetales|f__Dietziaceae|g__Dietzia|s__	p__Proteobacteria|c__Gammaproteobacteria|o__Xanthomonadales|f__Sinobacteraceae|g__|s__	0.694573	0.0022
C	p__Actinobacteria|c__Actinobacteria|o__Actinomycetales|f__Mycobacteriaceae|g__Mycobacterium|s__	p__Chloroflexi|c__Ktedonobacteria|o__Thermogemmatisporales|f__Thermogemmatisporaceae|g__|s__	0.695917	0.0005
C	p__Actinobacteria|c__Actinobacteria|o__Actinomycetales|f__Micromonosporaceae|g__Solwaraspora|s__	p__Actinobacteria|c__Actinobacteria|o__Actinomycetales|f__Nocardioidaceae|g__|s__	0.696505	0.001
C	p__Acidobacteria|c__Solibacteres|o__Solibacterales|f__|g__|s__	p__Actinobacteria|c__Actinobacteria|o__Actinomycetales|f__Micromonosporaceae|g__Solwaraspora|s__	0.699261	0.0014
C	p__Actinobacteria|c__Actinobacteria|o__Actinomycetales|f__Intrasporangiaceae|g__|s__	p__Actinobacteria|c__Actinobacteria|o__Actinomycetales|f__Microbacteriaceae|g__Microbacterium|s__	0.704085	0.0013
C	p__Actinobacteria|c__Actinobacteria|o__Actinomycetales|f__Mycobacteriaceae|g__Mycobacterium|s__	p__Chloroflexi|c__Thermomicrobia|o__JG30-KF-CM45|f__|g__|s__	0.704785	0.0021
C	p__Actinobacteria|c__Actinobacteria|o__Actinomycetales|f__Dietziaceae|g__Dietzia|s__	p__Actinobacteria|c__Actinobacteria|o__Actinomycetales|f__Mycobacteriaceae|g__Mycobacterium|s__	0.705065	0.0032
C	p__Actinobacteria|c__Acidimicrobiia|o__Acidimicrobiales|f__AKIW874|g__|s__	p__Actinobacteria|c__Actinobacteria|o__Actinomycetales|f__Mycobacteriaceae|g__Mycobacterium|s__	0.708348	0.0053
C	p__Actinobacteria|c__Actinobacteria|o__Actinomycetales|f__Microbacteriaceae|g__Microbacterium|s__	p__Actinobacteria|c__Actinobacteria|o__Actinomycetales|f__Micromonosporaceae|g__Solwaraspora|s__	0.70866	0.0016
C	p__Chloroflexi|c__Chloroflexi|o__Roseiflexales|f__Kouleothrixaceae|g__|s__	p__Chloroflexi|c__Ktedonobacteria|o__Thermogemmatisporales|f__Thermogemmatisporaceae|g__|s__	0.708786	0.0003
C	p__Actinobacteria|c__Actinobacteria|o__Actinomycetales|f__Micromonosporaceae|g__Solwaraspora|s__	p__Actinobacteria|c__Thermoleophilia|o__Solirubrobacterales|f__Solirubrobacteraceae|g__|s__	0.709648	0.0005

<table>
<tr><td>C</td><td>p__Planctomycetes|c__Planctomycetia|o__Pirellulales|f__Pirellulaceae|g__|s__</td><td>p__Planctomycetes|c__Planctomycetia|o__Planctomycetales|f__Planctomycetaceae|g__Planctomyces|s__</td><td>0.711906</td><td>0.0066</td></tr>
<tr><td>C</td><td>p__Actinobacteria|c__Actinobacteria|o__Actinomycetales|f__Microbacteriaceae|g__Microbacterium|s__</td><td>p__Actinobacteria|c__Thermoleophilia|o__Solirubrobacterales|f__Solirubrobacteraceae|g__|s__</td><td>0.713551</td><td>0.0013</td></tr>
<tr><td>C</td><td>p__Acidobacteria|c__Chloracidobacteria|o__RB41|f__Ellin6075|g__|s__</td><td>p__Gemmatimonadetes|c__|o__|f__|g__|s__</td><td>0.715023</td><td>0.0052</td></tr>
<tr><td>C</td><td>p__Actinobacteria|c__Actinobacteria|o__Actinomycetales|f__Dietziaceae|g__Dietzia|s__</td><td>p__Proteobacteria|c__Gammaproteobacteria|o__Pseudomonadales|f__Moraxellaceae|g__Alkanindiges|s__</td><td>0.715562</td><td>0.0022</td></tr>
<tr><td>C</td><td>p__Cyanobacteria|c__Chloroplast|o__Streptophyta|f__|g__|s__</td><td>p__Gemmatimonadetes|c__Gemm-3|o__|f__|g__|s__</td><td>0.717833</td><td>0.0067</td></tr>
<tr><td>C</td><td>p__Actinobacteria|c__Thermoleophilia|o__Solirubrobacterales|f__Solirubrobacteraceae|g__|s__</td><td>p__Proteobacteria|c__Gammaproteobacteria|o__Xanthomonadales|f__Sinobacteraceae|g__|s__</td><td>0.726341</td><td>0.0011</td></tr>
<tr><td>C</td><td>p__Planctomycetes|c__Phycisphaerae|o__|f__|g__|s__</td><td>p__Verrucomicrobia|c__Spartobacteria|o__Chthoniobacterales|f__Chthoniobacteraceae|g__Chthoniobacter|s__</td><td>0.729392</td><td>0.0038</td></tr>
<tr><td>C</td><td>p__Gemmatimonadetes|c__Gemm-3|o__|f__|g__|s__</td><td>p__Gemmatimonadetes|c__|o__|f__|g__|s__</td><td>0.733753</td><td>0.0032</td></tr>
<tr><td>C</td><td>p__Acidobacteria|c__Chloracidobacteria|o__RB41|f__Ellin6075|g__|s__</td><td>p__Cyanobacteria|c__Oscillatoriophycideae|o__Oscillatoriales|f__Phormidiaceae|g__Phormidium|s__</td><td>0.734737</td><td>0.007</td></tr>
<tr><td>C</td><td>p__Proteobacteria|c__Deltaproteobacteria|o__Myxococcales|f__Myxococcaceae|g__Anaeromyxobacter|s__</td><td>p__Proteobacteria|c__Gammaproteobacteria|o__Xanthomonadales|f__Sinobacteraceae|g__|s__</td><td>0.735714</td><td>0</td></tr>
<tr><td>C</td><td>p__Acidobacteria|c__Acidobacteria-6|o__iii1-15|f__|g__|s__</td><td>p__Planctomycetes|c__Planctomycetia|o__Pirellulales|f__Pirellulaceae|g__|s__</td><td>0.739018</td><td>0.0085</td></tr>
<tr><td>C</td><td>p__Chloroflexi|c__Ktedonobacteria|o__Thermogemmatisporales|f__Thermogemmatisporaceae|g__|s__</td><td>p__Proteobacteria|c__Gammaproteobacteria|o__Xanthomonadales|f__Sinobacteraceae|g__|s__</td><td>0.741909</td><td>0.0002</td></tr>
<tr><td>C</td><td>p__Chloroflexi|c__Chloroflexi|o__Roseiflexales|f__Kouleothrixaceae|g__|s__</td><td>p__Proteobacteria|c__Gammaproteobacteria|o__Xanthomonadales|f__Sinobacteraceae|g__|s__</td><td>0.74492</td><td>0.0042</td></tr>
<tr><td>C</td><td>p__Actinobacteria|c__Actinobacteria|o__Actinomycetales|f__Microbacteriaceae|g__Microbacterium|s__</td><td>p__Actinobacteria|c__Actinobacteria|o__Actinomycetales|f__Mycobacteriaceae|g__Mycobacterium|s__</td><td>0.745595</td><td>0.0013</td></tr>
<tr><td>C</td><td>p__Acidobacteria|c__Solibacteres|o__Solibacterales|f__|g__|s__</td><td>p__Actinobacteria|c__Thermoleophilia|o__Solirubrobacterales|f__Solirubrobacteraceae|g__|s__</td><td>0.749175</td><td>0.0001</td></tr>
<tr><td>C</td><td>p__Actinobacteria|c__Actinobacteria|o__Actinomycetales|f__Micromonosporaceae|g__Solwaraspora|s__</td><td>p__Actinobacteria|c__Actinobacteria|o__Actinomycetales|f__Mycobacteriaceae|g__Mycobacterium|s__</td><td>0.754661</td><td>0.0009</td></tr>
<tr><td>C</td><td>p__Gemmatimonadetes|c__Gemm-3|o__|f__|g__|s__</td><td>p__Verrucomicrobia|c__Spartobacteria|o__Chthoniobacterales|f__Chthoniobacteraceae|g__|s__</td><td>0.755527</td><td>0.0062</td></tr>
<tr><td>C</td><td>p__Actinobacteria|c__Actinobacteria|o__Actinomycetales|f__Dietziaceae|g__Dietzia|s__</td><td>p__Actinobacteria|c__Actinobacteria|o__Actinomycetales|f__Microbacteriaceae|g__Microbacterium|s__</td><td>0.757977</td><td>0.0014</td></tr>
<tr><td>C</td><td>p__Planctomycetes|c__Planctomycetia|o__Pirellulales|f__Pirellulaceae|g__|s__</td><td>p__Verrucomicrobia|c__Spartobacteria|o__Chthoniobacterales|f__Chthoniobacteraceae|g__|s__</td><td>0.758399</td><td>0.0054</td></tr>
<tr><td>C</td><td>p__Actinobacteria|c__Actinobacteria|o__Actinomycetales|f__Intrasporangiaceae|g__|s__</td><td>p__Chloroflexi|c__Chloroflexi|o__Roseiflexales|f__Kouleothrixaceae|g__|s__</td><td>0.759195</td><td>0.0011</td></tr>
<tr><td>C</td><td>p__Acidobacteria|c__Chloracidobacteria|o__RB41|f__Ellin6075|g__|s__</td><td>p__Gemmatimonadetes|c__Gemm-3|o__|f__|g__|s__</td><td>0.759274</td><td>0.0048</td></tr>
<tr><td>C</td><td>p__Actinobacteria|c__Actinobacteria|o__Actinomycetales|f__Dietziaceae|g__Dietzia|s__</td><td>p__Chloroflexi|c__Thermomicrobia|o__JG30-KF-CM45|f__|g__|s__</td><td>0.765789</td><td>0.0012</td></tr>
<tr><td>C</td><td>p__Actinobacteria|c__Actinobacteria|o__Actinomycetales|f__Intrasporangiaceae|g__|s__</td><td>p__Actinobacteria|c__Actinobacteria|o__Actinomycetales|f__Mycobacteriaceae|g__Mycobacterium|s__</td><td>0.77922</td><td>0.001</td></tr>
<tr><td>C</td><td>p__Cyanobacteria|c__Synechococcophycideae|o__|f__|g__|s__</td><td>p__Cyanobacteria|c__|o__|f__|g__|s__</td><td>0.779928</td><td>0.0012</td></tr>
<tr><td>C</td><td>p__Actinobacteria|c__Actinobacteria|o__Actinomycetales|f__Intrasporangiaceae|g__|s__</td><td>p__Actinobacteria|c__Actinobacteria|o__Actinomycetales|f__Micromonosporaceae|g__Solwaraspora|s__</td><td>0.781555</td><td>0.0002</td></tr>
<tr><td>C</td><td>p__Acidobacteria|c__Solibacteres|o__Solibacterales|f__|g__|s__</td><td>p__Proteobacteria|c__Gammaproteobacteria|o__Xanthomonadales|f__Sinobacteraceae|g__|s__</td><td>0.788405</td><td>0.0005</td></tr>
<tr><td>C</td><td>p__Actinobacteria|c__Actinobacteria|o__Actinomycetales|f__Microbacteriaceae|g__Microbacterium|s__</td><td>p__Proteobacteria|c__Gammaproteobacteria|o__Xanthomonadales|f__Sinobacteraceae|g__|s__</td><td>0.796212</td><td>0.0001</td></tr>
<tr><td>C</td><td>p__Actinobacteria|c__Actinobacteria|o__Actinomycetales|f__Microbacteriaceae|g__Microbacterium|s__</td><td>p__Chloroflexi|c__Ktedonobacteria|o__Thermogemmatisporales|f__Thermogemmatisporaceae|g__|s__</td><td>0.797855</td><td>0.0001</td></tr>
<tr><td>C</td><td>p__Verrucomicrobia|c__Spartobacteria|o__Chthoniobacterales|f__Chthoniobacteraceae|g__Chthoniobacter|s__</td><td>p__Verrucomicrobia|c__Spartobacteria|o__Chthoniobacterales|f__Chthoniobacteraceae|g__|s__</td><td>0.802049</td><td>0.0008</td></tr>
<tr><td>C</td><td>p__Acidobacteria|c__Acidobacteria-6|o__iii1-15|f__mb2424|g__|s__</td><td>p__Acidobacteria|c__Acidobacteria-6|o__iii1-15|f__|g__|s__</td><td>0.81651</td><td>0.0014</td></tr>
<tr><td>C</td><td>p__Actinobacteria|c__Actinobacteria|o__Actinomycetales|f__Micromonosporaceae|g__Solwaraspora|s__</td><td>p__Proteobacteria|c__Gammaproteobacteria|o__Xanthomonadales|f__Sinobacteraceae|g__|s__</td><td>0.847377</td><td>0</td></tr>
<tr><td>C</td><td>p__Gemmatimonadetes|c__Gemm-5|o__|f__|g__|s__</td><td>p__Verrucomicrobia|c__Verrucomicrobiae|o__Verrucomicrobiales|f__Verrucomicrobiaceae|g__Luteolibacter|s__</td><td>0.862249</td><td>0.0002</td></tr>
<tr><td>C</td><td>p__Actinobacteria|c__Actinobacteria|o__Actinomycetales|f__Intrasporangiaceae|g__|s__</td><td>p__Proteobacteria|c__Gammaproteobacteria|o__Xanthomonadales|f__Sinobacteraceae|g__|s__</td><td>0.874985</td><td>0</td></tr>
<tr><td>C</td><td>p__Actinobacteria|c__Actinobacteria|o__Actinomycetales|f__Mycobacteriaceae|g__Mycobacterium|s__</td><td>p__Proteobacteria|c__Gammaproteobacteria|o__Xanthomonadales|f__Sinobacteraceae|g__|s__</td><td>0.893887</td><td>0</td></tr>
<tr><td>DWH</td><td>p__Bacteroidetes|c__Flavobacteriia|o__Flavobacteriales|f__Flavobacteriaceae|g__|s__</td><td>p__Proteobacteria|c__Deltaproteobacteria|o__Desulfuromonadales|f__Desulfuromonadaceae|g__|s__</td><td>-0.6902</td><td>0.0017</td></tr>
<tr><td>DWH</td><td>p__Proteobacteria|c__Deltaproteobacteria|o__Desulfobacterales|f__Desulfobulbaceae|g__|s__</td><td>p__Verrucomicrobia|c__Verrucomicrobiae|o__Verrucomicrobiales|f__Verrucomicrobiaceae|g__Luteolibacter|s__</td><td>-0.68639</td><td>0.0029</td></tr>
<tr><td>DWH</td><td>p__Proteobacteria|c__Alphaproteobacteria|o__Rhodobacterales|f__Rhodobacteraceae|g__Octadecabacter|s__</td><td>p__Proteobacteria|c__Deltaproteobacteria|o__NB1-j|f__NB1-i|g__|s__</td><td>-0.67135</td><td>0.0049</td></tr>
<tr><td>DWH</td><td>p__Proteobacteria|c__Gammaproteobacteria|o__Alteromonadales|f__Colwelliaceae|g__Colwellia|s__</td><td>p__Verrucomicrobia|c__Verrucomicrobiae|o__Verrucomicrobiales|f__Verrucomicrobiaceae|g__Luteolibacter|s__</td><td>-0.6596</td><td>0.0074</td></tr>
</table>

DWH	p__Bacteroidetes|c__Flavobacteriia|o__Flavobacteriales|f__Flavobacteriaceae|g__|s__	p__Proteobacteria|c__Deltaproteobacteria|o__Desulfobacterales|f__Desulfobulbaceae|g__Desulfocapsa|s__	-0.65319	0.0018
DWH	p__Spirochaetes|c__Spirochaetes|o__Spirochaetales|f__Spirochaetaceae|g__|s__	p__Verrucomicrobia|c__Verrucomicrobiae|o__Verrucomicrobiales|f__Verrucomicrobiaceae|g__Persicirhabdus|s__	-0.63334	0.0042
DWH	p__Proteobacteria|c__Alphaproteobacteria|o__Rhodobacterales|f__Rhodobacteraceae|g__Phaeobacter|s__	p__Proteobacteria|c__Deltaproteobacteria|o__Desulfobacterales|f__Desulfobulbaceae|g__|s__	-0.62926	0.0049
DWH	p__Planctomycetes|c__Planctomycetia|o__Planctomycetales|f__Planctomycetaceae|g__Planctomyces|s__	p__Verrucomicrobia|c__Verrucomicrobiae|o__Verrucomicrobiales|f__Verrucomicrobiaceae|g__Luteolibacter|s__	0.723493	0.01
DWH	p__Bacteroidetes|c__Flavobacteriia|o__Flavobacteriales|f__Flavobacteriaceae|g__Lutimonas|s__	p__Proteobacteria|c__Alphaproteobacteria|o__Rhodobacterales|f__Rhodobacteraceae|g__Phaeobacter|s__	0.736513	0.0073
DWH	p__Actinobacteria|c__Acidimicrobiia|o__Acidimicrobiales|f__koll13|g__|s__	p__Planctomycetes|c__Planctomycetia|o__Pirellulales|f__Pirellulaceae|g__|s__	0.755076	0.0014
DWH	p__Actinobacteria|c__Acidimicrobiia|o__Acidimicrobiales|f__koll13|g__|s__	p__Proteobacteria|c__Deltaproteobacteria|o__NB1-j|f__NB1-i|g__|s__	0.760916	0.0027
DWH	p__Planctomycetes|c__Planctomycetia|o__Pirellulales|f__Pirellulaceae|g__|s__	p__Proteobacteria|c__Gammaproteobacteria|o__Marinicellales|f__Marinicellaceae|g__|s__	0.762125	0.0083
DWH	p__Actinobacteria|c__Acidimicrobiia|o__Acidimicrobiales|f__koll13|g__|s__	p__Proteobacteria|c__Alphaproteobacteria|o__Rhizobiales|f__|g__|s__	0.793544	0.0007
DWH	p__Proteobacteria|c__Deltaproteobacteria|o__Desulfobacterales|f__Desulfobulbaceae|g__|s__	p__Proteobacteria|c__Gammaproteobacteria|o__Alteromonadales|f__Colwelliaceae|g__Colwellia|s__	0.808185	0.0005
DWH	p__Proteobacteria|c__Alphaproteobacteria|o__Rhizobiales|f__|g__|s__	p__Proteobacteria|c__Gammaproteobacteria|o__Marinicellales|f__Marinicellaceae|g__|s__	0.84479	0.0088
I	p__Proteobacteria|c__Alphaproteobacteria|o__Rhizobiales|f__Hyphomicrobiaceae|g__Parvibaculum|s__	p__Proteobacteria|c__Betaproteobacteria|o__MOB121|f__|g__|s__	-0.89107	0.0032
I	p__Chloroflexi|c__Anaerolineae|o__envOPS12|f__|g__|s__	p__Proteobacteria|c__Alphaproteobacteria|o__Rhodospirillales|f__Acetobacteraceae|g__Acidocella|s__	-0.87625	0.0025
I	p__Bacteroidetes|c__Flavobacteriia|o__Flavobacteriales|f__Weeksellaceae|g__Chryseobacterium|s__	p__Proteobacteria|c__Gammaproteobacteria|o__Xanthomonadales|f__Xanthomonadaceae|g__Dyella|s__ginsengisoli	-0.87494	0.0055
I	p__Chlorobi|c__Ignavibacteria|o__Ignavibacteriales|f__Ignavibacteriaceae|g__|s__	p__Proteobacteria|c__Alphaproteobacteria|o__Rhodospirillales|f__Acetobacteraceae|g__Acidocella|s__	-0.8733	0.0057
I	p__Acidobacteria|c__Acidobacteriia|o__Acidobacteriales|f__|g__|s__	p__Chloroflexi|c__Anaerolineae|o__envOPS12|f__|g__|s__	-0.86394	0.0081
I	p__Bacteroidetes|c__Saprospirae|o__Saprospirales|f__Chitinophagaceae|g__|s__	p__Proteobacteria|c__Alphaproteobacteria|o__Rhodospirillales|f__Acetobacteraceae|g__Acidocella|s__	-0.86366	0.0016
I	p__Proteobacteria|c__Betaproteobacteria|o__Burkholderiales|f__Comamonadaceae|g__Methylibium|s__petroleiphilum	p__Proteobacteria|c__Betaproteobacteria|o__MOB121|f__|g__|s__	-0.86204	0.0097
I	p__Cyanobacteria|c__4C0d-2|o__MLE1-12|f__|g__|s__	p__Proteobacteria|c__Alphaproteobacteria|o__Rhizobiales|f__Hyphomicrobiaceae|g__Parvibaculum|s__	-0.81942	0.0079
I	p__Chloroflexi|c__Anaerolineae|o__Anaerolineales|f__Anaerolinaceae|g__|s__	p__Proteobacteria|c__Alphaproteobacteria|o__Rhizobiales|f__Hyphomicrobiaceae|g__Parvibaculum|s__	-0.73563	0.0095
I	p__Proteobacteria|c__Alphaproteobacteria|o__Sphingomonadales|f__Sphingomonadaceae|g__Sphingobium|s__yanoikuyae	p__Proteobacteria|c__Alphaproteobacteria|o__Sphingomonadales|f__Sphingomonadaceae|g__Sphingobium|s__	0.924636	0.0095
I	p__Proteobacteria|c__Alphaproteobacteria|o__Rhizobiales|f__Hyphomicrobiaceae|g__Parvibaculum|s__	p__Proteobacteria|c__Betaproteobacteria|o__Burkholderiales|f__Comamonadaceae|g__Methylibium|s__petroleiphilum	0.945539	0.0089
OSC	p__Proteobacteria|c__Betaproteobacteria|o__Burkholderiales|f__Oxalobacteraceae|g__Cupriavidus|s__	p__Proteobacteria|c__Betaproteobacteria|o__Burkholderiales|f__Oxalobacteraceae|g__Herminiimonas|s__	-0.73902	0.0003
OSC	p__Proteobacteria|c__Betaproteobacteria|o__Burkholderiales|f__Oxalobacteraceae|g__Herminiimonas|s__	p__Proteobacteria|c__Gammaproteobacteria|o__Enterobacteriales|f__Enterobacteriaceae|g__|s__	0.681937	0.0096
OSC	p__Actinobacteria|c__Actinobacteria|o__Actinomycetales|f__Propionibacteriaceae|g__Propionibacterium|s__	p__Proteobacteria|c__Betaproteobacteria|o__Burkholderiales|f__Oxalobacteraceae|g__Ralstonia|s__	0.710158	0.0066
OSC	p__Actinobacteria|c__Actinobacteria|o__Actinomycetales|f__Propionibacteriaceae|g__Propionibacterium|s__	p__Proteobacteria|c__Betaproteobacteria|o__Burkholderiales|f__Oxalobacteraceae|g__Cupriavidus|s__	0.779666	0.0063
OSC	p__Proteobacteria|c__Betaproteobacteria|o__Burkholderiales|f__Oxalobacteraceae|g__Cupriavidus|s__	p__Proteobacteria|c__Betaproteobacteria|o__Burkholderiales|f__Oxalobacteraceae|g__Ralstonia|s__	0.796845	0.0024
OSC	p__Proteobacteria|c__Betaproteobacteria|o__Burkholderiales|f__Oxalobacteraceae|g__Cupriavidus|s__gilardii	p__Proteobacteria|c__Gammaproteobacteria|o__Pseudomonadales|f__Moraxellaceae|g__Acinetobacter|s__	1	0
OSC	p__Proteobacteria|c__Alphaproteobacteria|o__Rhizobiales|f__Brucellaceae|g__Ochrobactrum|s__intermedium	p__Proteobacteria|c__Gammaproteobacteria|o__Pseudomonadales|f__Moraxellaceae|g__Acinetobacter|s__	1	0
OSC	p__Proteobacteria|c__Alphaproteobacteria|o__Caulobacterales|f__Caulobacteraceae|g__Phenylobacterium|s__	p__Proteobacteria|c__Gammaproteobacteria|o__Pseudomonadales|f__Moraxellaceae|g__Acinetobacter|s__	1	0
OSC	p__Proteobacteria|c__Alphaproteobacteria|o__Rhizobiales|f__|g__|s__	p__Proteobacteria|c__Gammaproteobacteria|o__Pseudomonadales|f__Moraxellaceae|g__Acinetobacter|s__	1	0
OSTPd	p__Proteobacteria|c__Alphaproteobacteria|o__Rhizobiales|f__|g__|s__	p__Proteobacteria|c__Deltaproteobacteria|o__Desulfuromonadales|f__Pelobacteraceae|g__Pelobacter|s__	-0.82995	0.0081
OSTPd	p__Proteobacteria|c__Alphaproteobacteria|o__Rhizobiales|f__|g__|s__	p__Proteobacteria|c__Deltaproteobacteria|o__Desulfuromonadales|f__Geobacteraceae|g__Geobacter|s__	-0.69054	0.0035
OSTPd	p__Bacteroidetes|c__Bacteroidia|o__Bacteroidales|f__|g__|s__	p__Proteobacteria|c__Alphaproteobacteria|o__Rhizobiales|f__|g__|s__	-0.65548	0.0054
OSTPd	p__Proteobacteria|c__Betaproteobacteria|o__Burkholderiales|f__Oxalobacteraceae|g__Cupriavidus|s__gilardii	p__Proteobacteria|c__Deltaproteobacteria|o__Desulfuromonadales|f__Geobacteraceae|g__Geobacter|s__	-0.6231	0.0062
OSTPd	p__Proteobacteria|c__Alphaproteobacteria|o__Rhizobiales|f__Methylobacteriaceae|g__Methylobacterium|s__	p__Proteobacteria|c__Deltaproteobacteria|o__Desulfuromonadales|f__Geobacteraceae|g__Geobacter|s__	-0.60415	0.0055
OSTPd	p__Proteobacteria|c__Deltaproteobacteria|o__Desulfuromonadales|f__Geobacteraceae|g__Geobacter|s__	p__Proteobacteria|c__Deltaproteobacteria|o__Desulfuromonadales|f__Pelobacteraceae|g__Pelobacter|s__	0.920038	0.0084
OSTPu	p__Proteobacteria|c__Betaproteobacteria|o__Burkholderiales|f__Comamonadaceae|g__Polaromonas|s__	p__Thermotogae|c__Thermotogae|o__Thermotogales|f__Thermotogaceae|g__Thermotoga|s__	-0.89322	0.0006

OSTPu	p__AC1|c__TA06|o__|f__|g__|s__	p__Firmicutes|c__Clostridia|o__Clostridiales|f__Tissierellaceae|g__Tissierella_Soehngenia|s__	-0.80759	0.0004
OSTPu	p__Firmicutes|c__Clostridia|o__Clostridiales|f__Peptostreptococcaceae|g__|s__	p__Proteobacteria|c__Betaproteobacteria|o__Burkholderiales|f__Comamonadaceae|g__Polaromonas|s__	-0.7345	0.0087
OSTPu	p__Proteobacteria|c__Betaproteobacteria|o__Burkholderiales|f__Comamonadaceae|g__Hydrogenophaga|s__palleronii	p__Proteobacteria|c__Deltaproteobacteria|o__Syntrophobacterales|f__Syntrophaceae|g__Syntrophus|s__	-0.71111	0.0097
OSTPu	p__Firmicutes|c__Clostridia|o__Clostridiales|f__Peptococcaceae|g__Pelotomaculum|s__	p__Proteobacteria|c__Betaproteobacteria|o__Hydrogenophilales|f__Hydrogenophilaceae|g__Thiobacillus|s__	0.912775	0.01
OSTPu	p__Chloroflexi|c__Dehalococcoidetes|o__GIF9|f__|g__|s__	p__Proteobacteria|c__Betaproteobacteria|o__Hydrogenophilales|f__Hydrogenophilaceae|g__Thiobacillus|s__	0.925418	0.01
OSTPu	p__Chloroflexi|c__Dehalococcoidetes|o__GIF9|f__|g__|s__	p__Proteobacteria|c__Betaproteobacteria|o__Rhodocyclales|f__Rhodocyclaceae|g__|s__	0.941872	0.0064
OSTPu	p__Chloroflexi|c__Dehalococcoidetes|o__GIF9|f__|g__|s__	p__Proteobacteria|c__Betaproteobacteria|o__Rhodocyclales|f__Rhodocyclaceae|g__Sulfuritalea|s__	0.954342	0.0026
OSTPu	p__Chloroflexi|c__Dehalococcoidetes|o__GIF9|f__|g__|s__	p__Proteobacteria|c__Betaproteobacteria|o__Burkholderiales|f__Comamonadaceae|g__|s__	0.954959	0.008
OSTPu	p__Firmicutes|c__Clostridia|o__Clostridiales|f__Peptococcaceae|g__Pelotomaculum|s__	p__Proteobacteria|c__Betaproteobacteria|o__Burkholderiales|f__Comamonadaceae|g__|s__	0.958396	0.0058
OSTPu	p__Proteobacteria|c__Gammaproteobacteria|o__PYR10d3|f__|g__|s__	p__Proteobacteria|c__Gammaproteobacteria|o__Xanthomonadales|f__Xanthomonadaceae|g__|s__	0.977145	0.0087
OSTPu	p__Chloroflexi|c__Anaerolineae|o__Anaerolineales|f__Anaerolinaceae|g__SHD-231|s__	p__Proteobacteria|c__Betaproteobacteria|o__Burkholderiales|f__Comamonadaceae|g__Polaromonas|s__	0.979527	0.0055
OSTPu	p__Proteobacteria|c__Betaproteobacteria|o__Hydrogenophilales|f__Hydrogenophilaceae|g__Thiobacillus|s__	p__Proteobacteria|c__Betaproteobacteria|o__Rhodocyclales|f__Rhodocyclaceae|g__Sulfuritalea|s__	0.98091	0.0093
OSTPu	p__Proteobacteria|c__Betaproteobacteria|o__Rhodocyclales|f__Rhodocyclaceae|g__|s__	p__Proteobacteria|c__Deltaproteobacteria|o__Syntrophobacterales|f__Syntrophaceae|g__Syntrophus|s__	0.982277	0.01
OSTPu	p__Proteobacteria|c__Betaproteobacteria|o__Hydrogenophilales|f__Hydrogenophilaceae|g__Thiobacillus|s__	p__Proteobacteria|c__Gammaproteobacteria|o__Xanthomonadales|f__Xanthomonadaceae|g__|s__	0.984076	0.0048
OSTPu	p__Proteobacteria|c__Betaproteobacteria|o__Hydrogenophilales|f__Hydrogenophilaceae|g__Thiobacillus|s__	p__Proteobacteria|c__Gammaproteobacteria|o__PYR10d3|f__|g__|s__	0.986003	0.0052
OSTPu	p__Proteobacteria|c__Betaproteobacteria|o__Burkholderiales|f__Comamonadaceae|g__|s__	p__Proteobacteria|c__Betaproteobacteria|o__Hydrogenophilales|f__Hydrogenophilaceae|g__Thiobacillus|s__	0.989547	0.004
OSTPu	p__Proteobacteria|c__Betaproteobacteria|o__Hydrogenophilales|f__Hydrogenophilaceae|g__Thiobacillus|s__	p__Proteobacteria|c__Deltaproteobacteria|o__Syntrophobacterales|f__Syntrophaceae|g__Syntrophus|s__	0.999945	0.0084
OSTPu	p__Chloroflexi|c__Dehalococcoidetes|o__GIF9|f__|g__|s__	p__Proteobacteria|c__Deltaproteobacteria|o__Syntrophobacterales|f__Syntrophaceae|g__Syntrophus|s__	1	0
OSTPu	p__Proteobacteria|c__Betaproteobacteria|o__Burkholderiales|f__Comamonadaceae|g__|s__	p__Proteobacteria|c__Deltaproteobacteria|o__Syntrophobacterales|f__Syntrophaceae|g__Syntrophus|s__	1	0
OSTPu	p__Proteobacteria|c__Betaproteobacteria|o__Rhodocyclales|f__Rhodocyclaceae|g__Sulfuritalea|s__	p__Proteobacteria|c__Deltaproteobacteria|o__Syntrophobacterales|f__Syntrophaceae|g__Syntrophus|s__	1	0
Tb	p__Acidobacteria|c__Acidobacteriia|o__Acidobacteriales|f__Koribacteraceae|g__Candidatus_Koribacter|s__	p__Proteobacteria|c__Alphaproteobacteria|o__Rhizobiales|f__Hyphomicrobiaceae|g__Devosia|s__	-0.74831	0.0086
Tb	p__Acidobacteria|c__iii1-8|o__DS-18|f__|g__|s__	p__AD3|c__ABS-6|o__|f__|g__|s__	0.912588	0.0073
Tb	p__AD3|c__ABS-6|o__|f__|g__|s__	p__Chloroflexi|c__Ktedonobacteria|o__Thermogemmatisporales|f__Thermogemmatisporaceae|g__|s__	0.970769	0.0073
Tp	p__Acidobacteria|c__iii1-8|o__DS-18|f__|g__|s__	p__Actinobacteria|c__Actinobacteria|o__Actinomycetales|f__Microbacteriaceae|g__Microbacterium|s__	0.885098	0.0098
Tp	p__Actinobacteria|c__Actinobacteria|o__Actinomycetales|f__Microbacteriaceae|g__Microbacterium|s__	p__Chloroflexi|c__Ktedonobacteria|o__Thermogemmatisporales|f__Thermogemmatisporaceae|g__|s__	0.915172	0.0024
Tp	p__Actinobacteria|c__Actinobacteria|o__Actinomycetales|f__Micrococcaceae|g__|s__	p__Actinobacteria|c__MB-A2-108|o__0319-7L14|f__|g__|s__	0.91852	0.0078
Tp	p__Chloroflexi|c__Ktedonobacteria|o__Thermogemmatisporales|f__Thermogemmatisporaceae|g__|s__	p__Chloroflexi|c__S085|o__|f__|g__|s__	0.925952	0.0038
Tp	p__Actinobacteria|c__Actinobacteria|o__Actinomycetales|f__Micrococcaceae|g__|s__	p__Chloroflexi|c__Ktedonobacteria|o__Thermogemmatisporales|f__Thermogemmatisporaceae|g__|s__	0.945306	0.0091
Tp	p__Actinobacteria|c__Actinobacteria|o__Actinomycetales|f__Micrococcaceae|g__|s__	p__Actinobacteria|c__Thermoleophilia|o__Gaiellales|f__Gaiellaceae|g__|s__	0.951573	0.0073
Tp	p__Actinobacteria|c__Thermoleophilia|o__Gaiellales|f__Gaiellaceae|g__|s__	p__Chloroflexi|c__Ktedonobacteria|o__Thermogemmatisporales|f__Thermogemmatisporaceae|g__|s__	0.956107	0.0032
Tu	p__Actinobacteria|c__Actinobacteria|o__Actinomycetales|f__Pseudonocardiaceae|g__Pseudonocardia|s__	p__Proteobacteria|c__Alphaproteobacteria|o__Sphingomonadales|f__Sphingomonadaceae|g__Novosphingobium|s__capsulatum	-0.86427	0.0028
Tu	p__Actinobacteria|c__Actinobacteria|o__Actinomycetales|f__Nocardioidaceae|g__|s__	p__Proteobacteria|c__Alphaproteobacteria|o__Rhodospirillales|f__Rhodospirillaceae|g__|s__	-0.78114	0.0068
Tu	p__Actinobacteria|c__Actinobacteria|o__Actinomycetales|f__Pseudonocardiaceae|g__Pseudonocardia|s__	p__Proteobacteria|c__Alphaproteobacteria|o__Rhizobiales|f__Bradyrhizobiaceae|g__|s__	-0.75025	0.0088
Tu	p__Acidobacteria|c__Solibacteres|o__Solibacterales|f__|g__|s__	p__Proteobacteria|c__Alphaproteobacteria|o__Rhizobiales|f__Bradyrhizobiaceae|g__Bosea|s__genosp.	0.936646	0.0052
Tu	p__Proteobacteria|c__Alphaproteobacteria|o__Rhizobiales|f__Bradyrhizobiaceae|g__Bosea|s__genosp.	p__Proteobacteria|c__Alphaproteobacteria|o__Sphingomonadales|f__Sphingomonadaceae|g__Novosphingobium|s__	0.977588	0.0044
Tu	p__Proteobacteria|c__Alphaproteobacteria|o__Caulobacterales|f__Caulobacteraceae|g__Arthrospira|s__fusiformis	p__Proteobacteria|c__Alphaproteobacteria|o__Caulobacterales|f__Caulobacteraceae|g__Phenylobacterium|s__	0.993837	0.0067
Tu	p__Acidobacteria|c__Solibacteres|o__Solibacterales|f__|g__|s__	p__Proteobacteria|c__Alphaproteobacteria|o__Sphingomonadales|f__Sphingomonadaceae|g__Novosphingobium|s__	1	0

[†] Acronyms represent the following habitats: A: Arctic, C: China oil refineries, I: India oil refineries, DWH: Marine sediments, OSC: Oil sands core, OSTPu: Oil sands tailings pond upper, OSTPd: Oil sands tailings pond deep, Tb: Taiga bottom active layer, Tu: Taiga upper active layer, Tp: Taiga permafrost layer.

‡ OTUs classification using the following method: p__|c__|o__|f__|g__|s__ which is interpreted as Phylum|Class|Order|Family|Genus|species.

Supplementary data. Table S9. Relative abundances of bacterial families detected at a relative abundance of ≥ 2% in at least one habitat.

Bacterial Family	India oil refineries (I)	Arctic (A)	China oil refineries (C)	Mangrove (M)	Marine sediments (DWH)	Taiga upper active layer (Tu)	Taiga bottom active layer (Tb)	Taiga permafrost layer (Tp)	Oil sands core (OSC)	Oil sands tailings pond upper (OSTPu)	Oil sands tailings pond median (OSTPm)	Oil sands tailings pond deep (OSTPd)
Acidobacteriaceae	7.113177	1.788807	0.008862	0	0	1.367593	1.346817	1.253056	0	0	0	0
Alteromonadaceae	0	0	0.430089	3.029981	13.68729	0	0	0	0.049528	0.037497	0	0.444972
Anaerolinaceae	1.334966	0	0.274713	0.282269	0	0.083575	0.197837	0.059669	0.004953	2.941468	5.976871	2.163787
Bradyrhizobiaceae	0.229136	0.950304	0.150649	0.039694	0.003261	2.262227	4.371447	1.443252	0.702059	0	0.082553	0.05235
Brucellaceae	0	0	0	0	0	0	0.068482	0.156632	3.788889	0	0	0.314098
Caldithrixaceae	0	0	0	2.619809	0	0	0	0	0	0	0	0
Caulobacteraceae	0.252382	2.185078	0.336745	0.017642	0	17.32284	25.21287	16.29719	6.683799	0.041664	0.286185	1.500691
Chitinophagaceae	3.858782	1.485704	0.738477	0.017642	0.004348	0.372289	0.190228	0.11188	1.124285	0.049997	0.176114	0.540947
Chthoniobacteraceae	0	0.104347	4.546062	0.185239	0.313031	0.182346	0.205447	0.134256	0	0	0	0
Comamonadaceae	15.60449	10.53906	1.240641	0.079388	0.126082	2.324908	1.554166	2.938715	12.62963	32.59571	28.08194	28.94937
Desulfobulbaceae	0.252382	0.003727	0.011816	1.36724	1.693412	0	0	0.007459	0.014858	1.86654	2.878364	1.806064
Ectothiorhodospiraceae	5.907722	0	0.008862	0.013231	0	0	0	0	0.022288	0	0.016511	0
Ellin6075	0	0	3.385177	0	0	0	0	0.022376	0.029717	0	0	0
Enterobacteriaceae	0	0.017391	0.039245	0.008821	0	0	0.007609	0.007459	2.751632	0.008333	0.257095	0.575846
Flavobacteriaceae	0.011955	1.206451	0.284313	0.727725	3.799851	0.118525	0.017121	0.080554	0.023773	0.360392	0.044029	0.029665
Hydrogenophilaceae	2.636724	0	0.011816	0.008821	0	0	0	0.022376	0.009906	1.32491	1.001649	1.343642
Ignavibacteriaceae	2.928955	0	0.008862	1.878853	0.026086	0	0	0	0	0.024998	0	0.026175
Koribacteraceae	11.11807	0.447202	0.132926	0.026463	0	1.094074	4.770927	1.409688	0.014858	0	0	0
Kouleothrixaceae	0	0	3.042523	0	0	0.030391	0.022827	0.014917	0	0	0	0
Methylobacteriaceae	0.019925	0.003727	0.28579	0.013231	0	0.056983	0.005707	0.005594	3.714597	0	0.198128	0.975013
Microbacteriaceae	2.21166	18.98123	1.559663	0.595411	0.30651	0.900332	0.285343	0.570588	4.274263	0.162489	0.47881	0.183224
Micrococcaceae	0	1.337879	1.344323	0.006616	0	4.60309	0.528454	1.805743	0.092865	0	0.033021	0
Moraxellaceae	0	0	3.983343	0	0.006521	0	0	0.212572	26.88749	0.224985	2.228944	6.997407
Nocardioidaceae	0.066416	2.089426	0.80937	0.068362	0	2.359098	0.114137	1.708035	0.230305	0.008333	0.033021	0
OM60	0	0	0.008862	2.513958	0	0	0	0	0	0	0	0

Opitutaceae	0.099624	0	2.259738	0.158776	0.039129	0	0	0	0.029717	0	0	0
Oxalobacteraceae	0.026566	0.531673	1.163839	0	0	0.212737	0.205447	0.693656	10.98035	0.024998	0.484314	1.279077
Pelobacteraceae	0	0	0.008862	0	0	0	0	0	0	0.237484	3.219585	2.394998
Peptococcaceae	0.009962	0	0.200275	0	0	0	0.004565	0.004475	0.010401	5.499628	1.793061	1.159545
Rhodobacteraceae	0.413441	0.443475	0.37618	1.098203	9.310069	0.022793	0.039948	0.145444	0.434608	0.087494	0.057787	0.163593
Rhodocyclaceae	2.666611	0.034782	0.047263	0.013231	0	0.117765	1.012015	0.376663	0.339267	11.05342	7.677472	16.38109
Sinobacteraceae	6.804341	0.685709	1.174178	0	0	0.136759	0.182619	0.078316	0.586906	0	0	0.091612
Sphingomonadaceae	2.789481	1.967688	2.184414	0.052925	0.032607	17.86988	28.56849	39.52721	1.649281	0.049997	0.181618	0.35336
Syntrophaceae	0.514726	0	0.007385	0.136724	0	0.007598	0	0	0	5.291309	6.42266	1.583578
Syntrophobacteraceae	0.318798	0	0.354469	0.158776	0	0	0.022827	0	0	0.612459	0.264171	2.277211
Thermogemmatisporaceae	0.019925	0.029813	0.864018	0.317553	0.026086	1.527145	5.512818	10.86355	0	0.037497	0.082553	0
Verrucomicrobiaceae	0	0	2.454696	1.058509	5.438917	0.045586	0	0	0	0	0.033021	0.026175
Weeksellaceae	1.354891	0.119254	0.177234	0.079388	3.665073	0	0	0.246136	0.995512	0	0.23115	0.575846
Xanthomonadaceae	8.932981	5.704307	1.434122	6.351053	19.66879	0.163351	0.045655	0.231219	0.425941	1.024931	0.148596	0.418797
